# Performance Evaluation of Large Language Models in Cervical Cancer Management Based on a Standardized Questionnaire: Comparative Study

**DOI:** 10.2196/63626

**Published:** 2025-02-05

**Authors:** Warisijiang Kuerbanjiang, Shengzhe Peng, Yiershatijiang Jiamaliding, Yuexiong Yi

**Affiliations:** 1 Department of Gynecology Zhongnan Hospital of Wuhan University Wuhan, Hubei Province China

**Keywords:** large language model, cervical cancer, screening, artificial intelligence, model interpretability

## Abstract

**Background:**

Cervical cancer remains the fourth leading cause of death among women globally, with a particularly severe burden in low-resource settings. A comprehensive approach—from screening to diagnosis and treatment—is essential for effective prevention and management. Large language models (LLMs) have emerged as potential tools to support health care, though their specific role in cervical cancer management remains underexplored.

**Objective:**

This study aims to systematically evaluate the performance and interpretability of LLMs in cervical cancer management.

**Methods:**

Models were selected from the AlpacaEval leaderboard version 2.0 and based on the capabilities of our computer. The questions inputted into the models cover aspects of general knowledge, screening, diagnosis, and treatment, according to guidelines. The prompt was developed using the Context, Objective, Style, Tone, Audience, and Response (CO-STAR) framework. Responses were evaluated for accuracy, guideline compliance, clarity, and practicality, graded as A, B, C, and D with corresponding scores of 3, 2, 1, and 0. The effective rate was calculated as the ratio of A and B responses to the total number of designed questions. Local Interpretable Model-Agnostic Explanations (LIME) was used to explain and enhance physicians’ trust in model outputs within the medical context.

**Results:**

Nine models were included in this study, and a set of 100 standardized questions covering general information, screening, diagnosis, and treatment was designed based on international and national guidelines. Seven models (ChatGPT-4.0 Turbo, Claude 2, Gemini Pro, Mistral-7B-v0.2, Starling-LM-7B alpha, HuatuoGPT, and BioMedLM 2.7B) provided stable responses. Among all the models included, ChatGPT-4.0 Turbo ranked first with a mean score of 2.67 (95% CI 2.54-2.80; effective rate 94.00%) with a prompt and 2.52 (95% CI 2.37-2.67; effective rate 87.00%) without a prompt, outperforming the other 8 models (*P*<.001). Regardless of prompts, QiZhenGPT consistently ranked among the lowest-performing models, with *P*<.01 in comparisons against all models except BioMedLM. Interpretability analysis showed that prompts improved alignment with human annotations for proprietary models (median intersection over union 0.43), while medical-specialized models exhibited limited improvement.

**Conclusions:**

Proprietary LLMs, particularly ChatGPT-4.0 Turbo and Claude 2, show promise in clinical decision-making involving logical analysis. The use of prompts can enhance the accuracy of some models in cervical cancer management to varying degrees. Medical-specialized models, such as HuatuoGPT and BioMedLM, did not perform as well as expected in this study. By contrast, proprietary models, particularly those augmented with prompts, demonstrated notable accuracy and interpretability in medical tasks, such as cervical cancer management. However, this study underscores the need for further research to explore the practical application of LLMs in medical practice.

## Introduction

Cervical cancer is a significant global public health challenge, ranking fourth among all female cancers and remaining the leading cause of death in many low-income countries [1]. In 2020, approximately 604,127 new cases and 341,831 deaths from cervical cancer were reported worldwide [1]. Effective cervical cancer control necessitates an integrated approach that combines screening, accurate diagnosis, and personalized treatment to reduce morbidity and mortality. Despite a substantial decline in cervical cancer incidence in the United States since the introduction of screening [2], up to 25% of women remain inadequately treated [3], with even higher rates observed in resource-limited and developing countries [1]. Moreover, precise diagnosis and appropriate treatment are essential for addressing abnormalities detected through screening, particularly to prevent disease progression and improve survival outcomes [4]. Hence, strengthening these efforts is essential for reducing the global burden of cervical cancer and improving patient outcomes across diverse health care contexts.

Large language models (LLMs), as cutting-edge technologies in artificial intelligence, are trained on vast data sets and enable a wide range of applications, from text polishing to complex problem-solving, thanks to their unprecedented natural language understanding capabilities. In the health care domain, LLMs hold the potential to revolutionize medical practices, including decision-making, patient management, and clinical data interpretation [5,6]. Notably, OpenAI’s proprietary LLMs, ChatGPT-3.5 and ChatGPT-4.0, have demonstrated high performance on the United States Medical Licensing Examination (USMLE), with ChatGPT-4.0 achieving particularly impressive results [7,8]. Additionally, ChatGPT has shown competence across various medical fields, including surgery [9], cardiology [10], and plastic surgery [11]. Compared with generic language models, medical-specialized models—fine-tuned on domain-specific data sets and subjected to specialized adjustments—have achieved equivalent or superior performance [12].

To date, only a limited number of studies [13] have applied LLMs to questions related to cervical cancer, as well as explainability analyses on either closed- or open-source LLMs to assess transparency and interpretability. The management of abnormal cervical cancer screening results, diagnosis, and treatment is a complex task that requires careful interpretation and follow-up [14]. When deploying LLMs in cervical cancer management, it is crucial to evaluate their performance in managing abnormalities and to identify their strengths and limitations, particularly regarding model transparency and interpretability.

**Figure 1:**
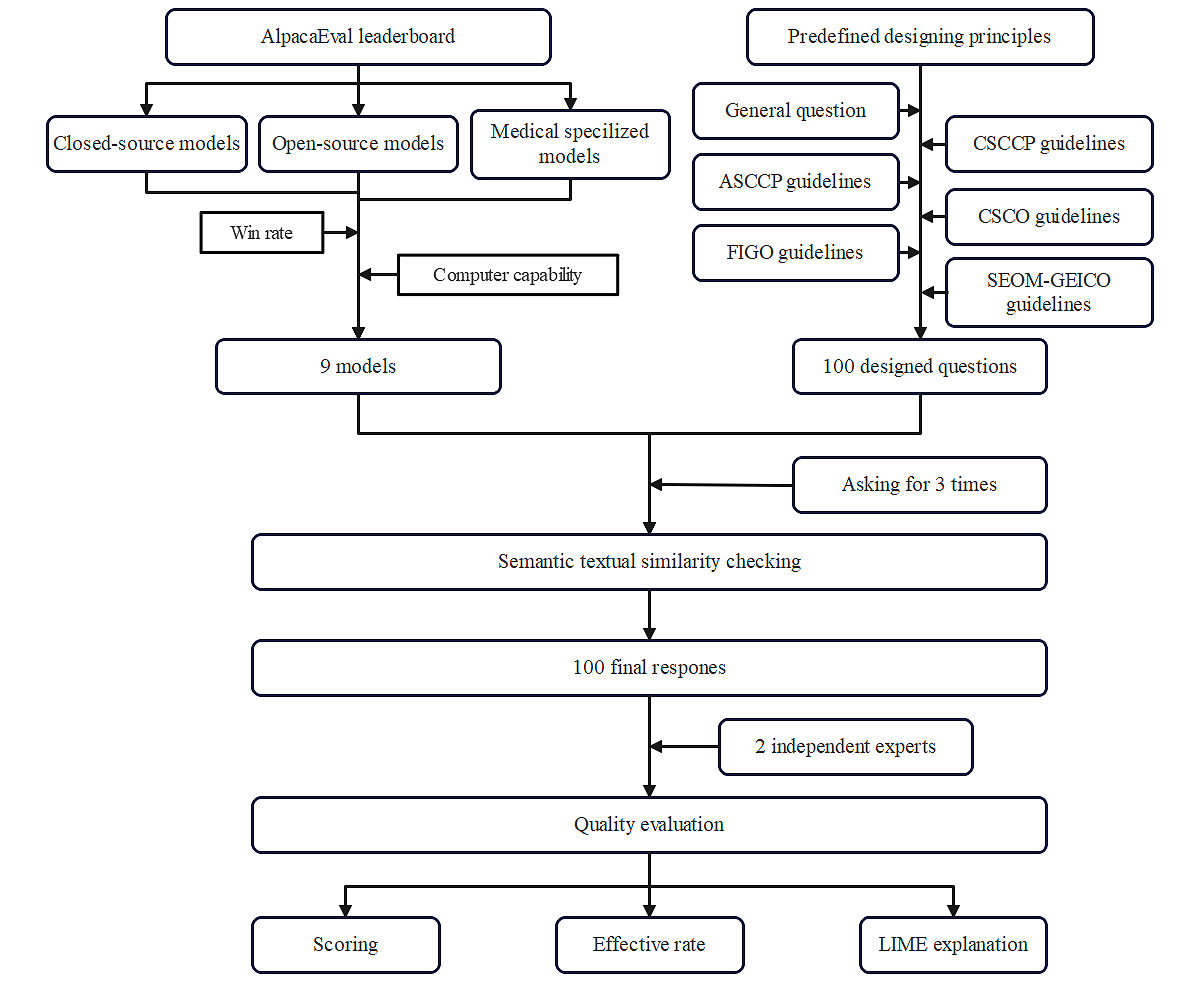
The flowchart of evaluation of LLMs' performance in cervical cancer management. Nine LLMs, including closed-source, open-source, and medical-specialized types, selected from AlpacaEval leaderboard were evaluated with 100 predefined questions derived from general inquiries and guidelines from ASCCP, CSCCP, FIGO, SEOM-GEICO, and CSCO guidelines. Responses were collected 3 times for each question, analyzed for semantic similarity, and reviewed by 2 experts for accuracy, effectiveness, and interpretability using LIME. ASCCP: American Society for Colposcopy and Cervical Pathology; CSCCP: Chinese Society for Colposcopy and Cervical Pathology of the China Healthy Birth Science Association; CSCO: Chinese Society of Clinical Oncology; FIGO: International Federation of Gynecology and Obstetrics; GEICO: Grupo Español de Investigación en Cáncer de Ovario; LIME: Local Interpretable Model-agnostic Explanations; LLM: large language model; SEOM: Sociedad Española de Oncología Médica.

In this study, we aim to compare the performance of current prevalent LLMs in cervical cancer management by evaluating their responses to a set of specifically designed questions (Figure 1). This research may provide valuable evidence to help clinicians manage screening results more effectively and accurately, particularly in regions with limited health care infrastructure.

## Methods

### Model Selection

The AlpacaEval leaderboard is an automated system designed to evaluate language models based on their adherence to instructions, ranking them by comparing their responses to reference answers from top-performing models such as GPT-4. It aims to reduce biases, such as those related to output length. Unlike other leaderboards that may focus on a single type, this leaderboard includes both open- and closed-source models. The selection of potential models—whether closed-source, open-source, or medically specialized—is determined by their win rates on version 2.0 of the leaderboard, updated on March 3, 2024.

For closed-source models, both free and paid versions are included, excluding those that are not publicly available or are in private beta. Open-source models are required to perform effectively on consumer-grade computers with standard configurations, given their potential use in resource-limited applications such as cervical cancer screening. The computer specifications for deploying these models are detailed in Multimedia Appendix 1, with a maximum model size capacity of approximately 7 billion trainable parameters. The selection of medical-specialized models, which are limited in number on leaderboards, is informed by a study [15] summarizing existing medical LLMs and their respective GitHub star counts. The performance of these medical LLMs is assessed based on the benchmark scores of their underlying models.

### Criteria for Question and Prompt Designing

#### Questions Designing

A comprehensive question set was developed to evaluate model performance, including general questions and those specifically focused on cervical cancer screening, diagnosis, and treatment. General questions were designed by our gynecological experts to address the most common queries about cervical cancer, covering essential, foundational information frequently encountered in clinical practice. Screening-related questions were crafted with reference to the Chinese Society for Colposcopy and Cervical Pathology of the China Healthy Birth Science Association (CSCCP) Consensus on cervical cancer screening and abnormal management in China [16]. To ensure relevance and keep our questions up to date, we also incorporate the 2019 American Society for Colposcopy and Cervical Pathology (ASCCP) Risk-Based Management Consensus Guidelines for Abnormal Cervical Cancer Screening Tests and Cancer Precursors [17]. The questions comprehensively address each clinical decision outlined in the CSCCP guideline flowcharts, as detailed in Multimedia Appendix 2. Additional screening questions were developed based on the Chinese Society of Clinical Oncology (CSCO) Guidelines for the Diagnosis and Treatment of Cervical Cancer (2023) [18]. The diagnosis and treatment questions were developed with reference to the Sociedad Española de Oncología Médica-Grupo Español de Investigación en Cáncer de Ovario (SEOM-GEICO) Clinical Guidelines on Cervical Cancer (2023) [19], the CSCO Guidelines for the Diagnosis and Treatment of Cervical Cancer [18], and The International Federation of Gynecology and Obstetrics (FIGO) 2018 Gynecologic Cancer Report – Interpretation of the Cervical Cancer Guidelines [20]. The design was guided by the principles outlined in Textbox 1.

**Principles guiding design.**

**1. Diverse complexity levels**

- A combination of basic and advanced questions was included to evaluate the model’s ability to address both routine and complex clinical scenarios.

**2. Strict guideline adherence**

- Questions were structured to prioritize guideline-based knowledge, minimizing reliance on outdated or nonevidence-based practices.

**3. Primarily closed-ended format**

- Predominantly closed-ended questions were used to reduce subjective bias, with a few open-ended questions included to assess the model’s capacity for divergent medical problem-solving.

**4. Definitive answers**

- Each question was designed to have a clear, definitive answer.

These questions aim to evaluate the models’ understanding of clinical guidelines, their decision-making processes, and their ability to provide clear, actionable advice.

#### Prompt Designing

The prompt was designed using the Context, Objective, Style, Tone, Audience, and Response (CO-STAR) framework, which was ranked as the top prompt in the inaugural GPT-4 Prompt Engineering Competition. This framework was applied to guide the LLM in generating expert-level responses in gynecology, with a clear focus on defining the context, objective, style, tone, audience, and response format.

### Questioning Method

**Figure 2:**
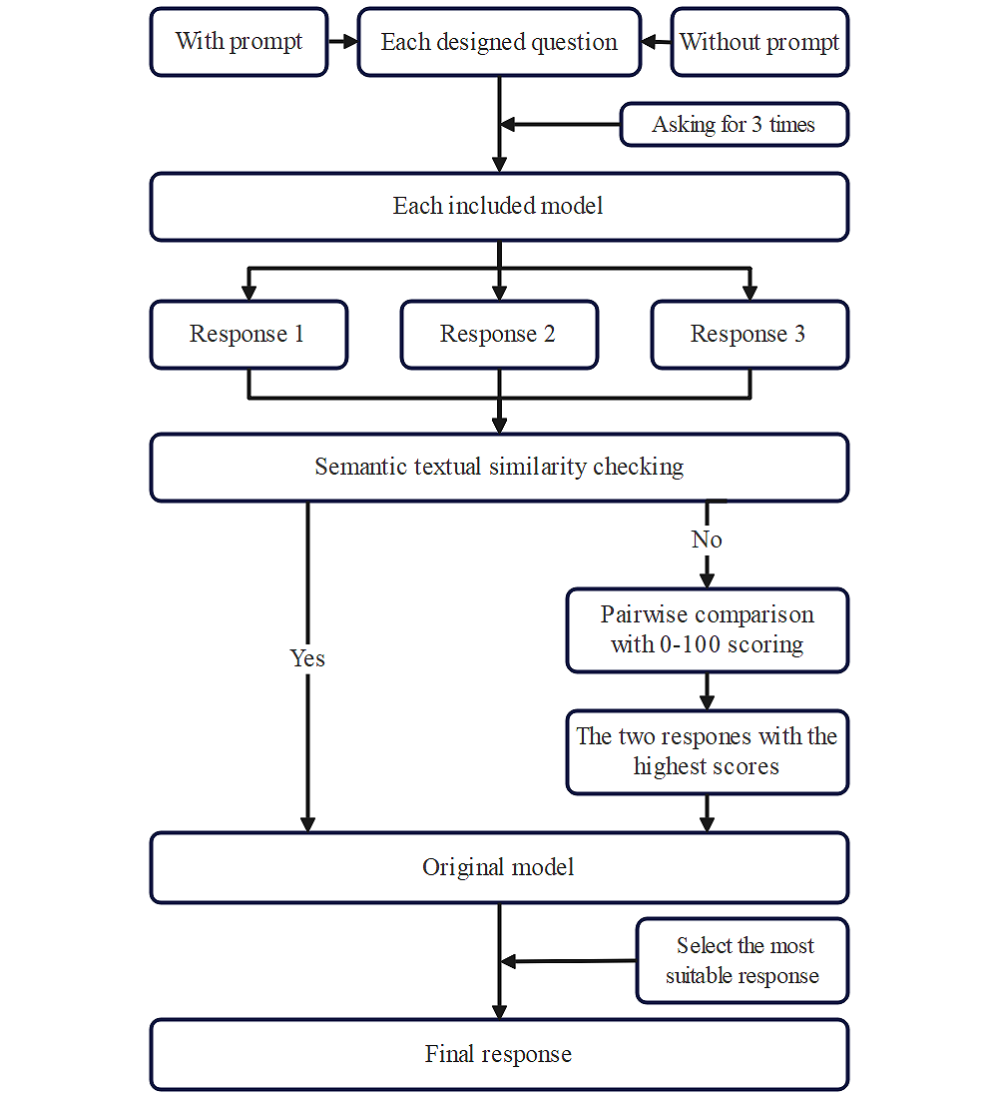
The flowchart of final response determination for LLMs. Each question was tested 3 times per model, with and without prompts, to assess consistency. Responses were analyzed using STS by ChatGPT-3.5 (not included as a test model). If all 3 responses were semantically identical, the model selected the most suitable answer. For discrepancies, a pairwise comparison scored responses from 0 (Not typical at all) to 100 (Extremely typical), and the 2 highest-scoring responses were returned to the model to determine the final response. LLM: large language model; STS: semantic textual similarity.

Each designed question was sequentially input 3 times for each model, both with and without the designed prompt, to test consistency. The coherence of the responses was evaluated using semantic textual similarity [21] by ChatGPT-3.5, the top-performing model on AlpacaEval, which was not used as a test model. If the semantics of all 3 responses are identical, they are sent back to the originating model to select the most suitable answer. In cases of discrepancies, a pairwise comparison is performed with scores ranging from 0 (not typical at all) to 100 (extremely typical) [22]. The 2 responses with the highest similarity scores are returned to their model, which then selects the most appropriate answer (Figure 2).

### Scoring Process and Criteria

Two gynecological experts independently and anonymously reviewed the responses to each question. If both experts agreed on a score, it was directly accepted; otherwise, they discussed it to determine the final score. Responses were evaluated based on accuracy, adherence to clinical guidelines, clarity of communication, and practicality. A scoring system, modified from a previous study [23], was used to categorize responses into 4 grades: A, B, C, and D, to minimize subjective bias. Grades A and B were considered effective, and the model’s effective rate was calculated as follows:

Effective rate = (*N_A_*+*N_B_*)/(*N_A_*+*N_B_*+*N_C_*+*N_D_*) × 100%

where *N* represents the number of each grade. Scores are weighted at 3, 2, 1, and 0 points for statistical analysis (Table 1).

**Table 1:** Criteria of scoring for response.

Grade	Description	Scores
A	Completely correct with comprehensive information	3
B	Mostly correct, but with missing information or minor errors	2
C	Contains major errors but with some correct content	1
D	Completely wrong or off-topic	0

### Model Explainability Analysis

**Figure 3:**
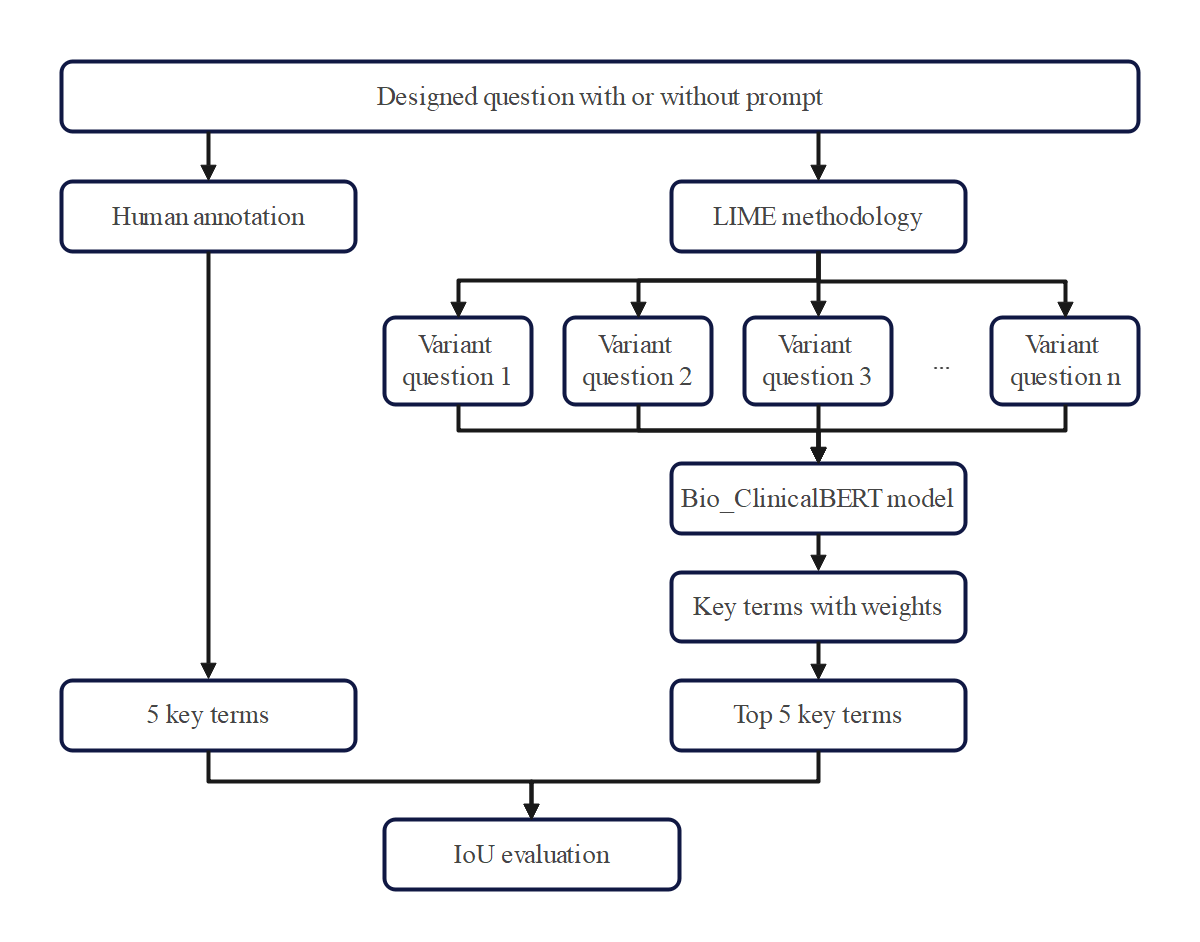
The flowchart of model explainability analysis by the Local Interpretable Model-agnostic Explanations (LIME) methodology. LIME was used to interpret large language model (LLM) outputs by analyzing each input question and generating variant questions with or without prompt. The number of samples was set to 10 times the token count of the input sentence. Key terms were extracted using the Bio_ClinicalBERT model, and the top 5 terms by weight were selected. Experts manually annotated 5 key terms per question for comparison. An intersection over union (IoU) analysis was conducted to evaluate the alignment between LIME-selected and expert-annotated key terms. BERT: Bidirectional Encoder Representations from Transformers.

Local Interpretable Model-Agnostic Explanations (LIME) is widely recognized for generating locally interpretable explanations of machine learning model predictions, including natural language processing models [24,25]. In this study, LIME was used to interpret LLM outputs by adapting methods previously successful in natural language processing. The primary LIME parameter, the number of samples, was set to 10 times the input sentence’s token count, based on preliminary experiments and prior applications of LIME to LLMs [26]. Each input question was analyzed to identify key terms with assigned weights, and the top 5 key terms by weight were selected. Our experts manually annotated 5 key terms per question for comparison. An intersection-over-union (IoU) analysis was performed between the LIME-selected key terms and the expert-annotated key terms to evaluate their alignment (Figure 3).

IoU(*x*1, *x*2) = (|*x*1∩*x*2|)/(| *x*1∪*x*2|)

### Ethical Considerations

This study did not involve human participants, identifiable patient data, or protected health information. The data utilized in this study comprised publicly available sources, including leaderboards, clinical guidelines, and secondary analyses of model-generated outputs. Therefore, an ethical review was not required under Zhongnan Hospital of Wuhan University’s secondary research policies. The study complied with the Declaration of Helsinki and institutional guidelines for secondary data use.

### Statistical Methods

Analyses were conducted using R version 4.3.1 (R Foundation) and RStudio 2023.12.1+402 (R Foundation). Differences across models were assessed using the chi-square test for categorical variables. For paired comparisons, data were first tested for normality. If normally distributed, a paired *t* test was applied, with results reported as mean and SD; otherwise, a paired Wilcoxon rank sum test was used, with outcomes presented as median and IQR. Effective rates were reported as mean values with 95% CIs. A *P* value of less than .05 was considered indicative of a significant difference.

## Results

### Model Selection

After screening for win rates and conducting tests on our computers, our study included 9 models. The proprietary models are ChatGPT-4.0 Turbo, Claude 2, and Gemini Pro, which are accessible through their official websites. The open-source LLMs include Mistral-7B-v0.2, Starling-LM-7B Alpha, and Microsoft Phi-2. The medical-specialized models are the Chinese models HuatuoGPT and QiZhenGPT, along with the English model BioMedLM 2.7B. The expected performance ranking of the selected models is as follows: ChatGPT-4.0 Turbo > Gemini Pro > Claude 2 > Mistral-7B-v0.2 > Starling-LM-7B Alpha > ChatGLM 6B (QiZhenGPT) > Phi-2 > Baichuan2-7B-Chat (HuatuoGPT). BioMedLM 2.7B is excluded from this ranking because it is not listed on the AlpacaEval Leaderboard. The characteristics of the included models are presented in Table 2.

**Table 2:** The characteristics of included models.

Model and access reference	AlpacaEval win rate	Description
ChatGPT-4.0 Turbo [27]	50%	Developed by OpenAI, ChatGPT-4.0 Turbo is an LLM^a^ that is currently the most powerful in terms of performance.
Claude 2 [28]	17.19%	Developed by Anthropic, it is built on the GPT-3 architecture. This model features a context window of 100,000 ultra-long tokens, enabling it to handle longer context inputs efficiently.
Mistral-7B-v0.2 [29]	14.72%	Mistral-7B-v0.2 is the strongest open-source model on the list that can be deployed on consumer computers. Furthermore, the popularity of this model is high, as it received more than 700,000 downloads in January 2024.
Starling-LM-7B alpha [30]	14.25%	A fine-tuned model that outperforms all models to date on MT-Bench except for OpenAI’s GPT-4 and GPT-4 Turbo.
Gemini Pro [31]	18.18%	Developed by Google DeepMind, the more advanced Gemini Ultra is not yet available to the public, so we used the Pro version.
HuatuoGPT 2-7B [32]	1.99% (base model)	Developed by the Shenzhen Institute of Big Data and The Chinese University of Hong Kong, this Chinese medical LLM is fine-tuned based on Baichuan2-7B. Uses deploying method. The online demo is available at [33].
QiZhenGPT [34]	3.01% (base model)	Released by Zhejiang University, the project includes 3 versions, each fine-tuned from the base models of ChatGLM-6B, Chinese-LLaMA-7B, and CaMA-13B.
Phi-2 [35]	2.34%	Released by Microsoft, this small language model has a data size of only 2.7 billion. Easy to deploy, even on consumer-grade computers, where it exhibits exceptionally fast response times.
BioMedLM 2.7B [36]	N/A^b^	Previously known as PubMedGPT 2.7B, this model was developed through pretraining.

^a^LLM: large language model.

^b^N/A: not applicable.

### Questions and Prompts for LLMs

The question set consisted of 100 questions designed to encompass a broad range of clinical scenarios commonly encountered in cervical cancer management. The first 22 questions focused on general knowledge, emphasizing foundational aspects frequently encountered in clinical gynecology. The next 40 questions addressed cervical cancer screening, aligning with the latest consensus guidelines and decision-making protocols. Subsequently, 6 and 32 questions covered diagnosis and treatment, respectively, offering a comprehensive evaluation of the models’ ability to interpret diagnostic criteria and recommend evidence-based treatment options. By including both routine and complex queries, the question set serves as a robust benchmark for assessing model performance, accuracy, and adherence to evidence-based medical practices. The complete list of questions is provided in Table 3.

**Table 3:** The 100 designed questions based on cervical cancer guidelines.

Category	Questions
Questions related to general knowledge	What are the risk factors that may necessitate cervical cancer screening? At what age or under what conditions is cervical cancer screening typically deemed unnecessary? What strategies are effective in reducing the risk of developing cervical cancer? What are the common clinical symptoms of cervical cancer? For individuals who have been vaccinated against HPV^a^, is it still necessary for them to undergo cervical cancer screening? Is cervical cancer screening still recommended for individuals who have had only 1 sexual partner or are not currently sexually active? What are the recommended intervals for cervical cancer screening, and do these intervals vary among different age groups? Is cervical cancer screening universally recommended for all age groups? If not, what are the reasons for excluding certain age groups from undergoing cervical cancer screening? How necessary is cervical cancer screening for women who have undergone total hysterectomy? What is the significance of cervical cancer screening? Why is the combined use of cytological screening (Papanicolaou test) and HPV testing not recommended for women aged 21-29 years? Is there an invariable link between HPV infection and the onset of cervical cancer? How should one interpret an abnormal result from a cervical cancer screening test? Does such a result definitively indicate the presence of cervical cancer? Why is yearly cervical cancer screening not recommended? What are the objectives of cervical cancer screening protocols? Is cervical cancer hereditary? If so, should individuals with a familial history of cervical cancer be subject to more frequent screening protocols? What’s the difference between a pelvic examination and a Pap test? Can individuals independently administer HPV tests, and if so, how accurate are these self-administered tests? Is it possible for cervical cancer to manifest within the interscreening interval, particularly between 2 consecutive cervical screening tests? Is cervical screening necessary for individuals who have reached menopause? Is it recommended to undergo cervical screening during pregnancy? What procedures are typically involved in cervical cancer screening?
Questions related to screening	What is the examination process for HPV-positive high-risk types? When the initial screening shows positive HPV with high-risk HPV types, emphasizing that HPV is not typed, what is the next step in the examination? When the initial screening shows positive HPV with high-risk HPV types, and the cytological examination result is negative, what does it indicate? Should regular check-ups follow? And if so, what should be the frequency? When the initial screening shows positive HPV with high-risk HPV types, and the cytological examination result is ≥ASC-US^b^, should the next step be a colposcopy? When the initial screening shows positive HPV with high-risk HPV types, typing identifies HPV16/18 positivity, should the next step be a colposcopy? When the initial screening shows positive HPV with high-risk HPV types, and upon typing, it shows neither HPV16/18 positive but 1 of the other 12 types, what is the most likely subsequent examination? When cytological examination indicates ASC-US as an abnormal initial screening result, what is the next step in the examination? When cytological examination indicates ASC-US as an abnormal initial screening result, and HPV is used for triage, if HPV is positive, should a colposcopy follow? When cytological examination indicates ASC-US as an abnormal initial screening result, and HPV is used for triage, if HPV is negative, what should be the subsequent examination? When cytological examination results show ASC-H^c^, LSIL^d^, HSIL^e^, is a colposcopy needed next? When cytological examination results show AGC^f^, what is the next examination required? If both cytological and high-risk HPV joint tests show negative results, what other examination should follow? If joint testing of cytology and high-risk HPV shows HPV negative and the cytological result is ASC-US, what should be done next? If joint testing of cytology and high-risk HPV shows HPV negative, and the cytological result is >ASC-US, what should be done next? Is a colposcopy required? In cervical cancer detection, if joint testing of cytology and high-risk HPV shows HPV positive and the cytological result is ≥ASC-US, what specific examinations should follow? In cervical cancer detection, if joint testing of cytology and high-risk HPV shows HPV positive and the cytological result is negative, what specific examinations should follow? In cervical cancer detection, if joint testing of cytology and high-risk HPV shows HPV positive and the cytological result is negative, and the HPV typing is HPV16/18 positive, what specific examinations should follow? In cervical cancer detection, if a patient has joint testing of cytology and high-risk HPV, with the results showing HPV positivity, a negative cytological result, and no typing for HPV16 or HPV18, are additional tests needed? In cervical cancer screening, if a patient has a histopathological confirmation (biopsy) of LSIL, cytology findings of LSIL or higher, and a TZ3^g^ classification, what follow-up steps should be conducted? In cervical cancer screening, if a patient has a histopathological confirmation (biopsy) of LSIL, cytology findings of LSIL or higher, and a TZ1/2 classification, what follow-up steps should be conducted? In cervical cancer screening, if a patient has a histopathological confirmation (biopsy) of LSIL and cytology findings of ASC-H or higher, what follow-up steps should be conducted? In cervical cancer screening, if a patient aged 21-24 years has a histopathological confirmation (biopsy) of LSIL and cytology findings of ASC-H or higher, what follow-up steps should be conducted? In cervical cancer screening, if a patient aged 21-24 years has a histopathological confirmation (biopsy) of LSIL, cytology findings of ASC-H or higher, and a TZ3 classification for colposcopy, what follow-up steps should be conducted? In cervical cancer screening, if a patient aged 21-24 years has a histopathological confirmation (biopsy) of LSIL, cytology findings of ASC-H or higher, and a TZ1/2 classification for colposcopy, what follow-up steps should be conducted? In cervical cancer screening, if a pregnant patient has a histopathological confirmation (biopsy) of LSIL and cytology findings of ASC-H or higher, what follow-up steps should be conducted? In cervical cancer screening, if a patient has a histopathological confirmation (biopsy) of HSIL and a TZ1/2 classification, what follow-up steps should be conducted? In cervical cancer screening, if a patient has a histopathological confirmation (biopsy) of HSIL and a TZ3 classification, what follow-up steps should be conducted? In cervical cancer screening, if a patient aged 21-24 years has a histopathological confirmation (biopsy) of CIN^h^ III/HSIL, what follow-up steps should be conducted? In cervical cancer screening, if a patient aged 21-24 years has a histopathological confirmation (biopsy) of HSIL and a TZ3 classification for colposcopy, what follow-up steps should be conducted? In cervical cancer screening, if a patient aged 21-24 years has a histopathological confirmation (biopsy) of CIN II/III/HSIL or CIN II/HSIL and a TZ1/2 classification for colposcopy, what follow-up steps should be conducted? In cervical cancer screening, if a pregnant patient has a histopathological confirmation (biopsy) of HSIL, what follow-up steps should be conducted? After diagnostic/therapeutic cervical conization for cervical cancer, what follow-up steps should be conducted? In cervical cancer screening, if a pregnant patient has a histopathological confirmation (biopsy) of HSIL without invasive cancer during pregnancy, what follow-up steps should be conducted after childbearing? What are the strategies for HPV vaccine use? What are the recommended vaccination programs for different age groups? Is HPV primary screening applicable in low-income countries? If so, why? Which cervical cancer screening methods are widely used in low-income countries, particularly in sub-Saharan Africa? What are the key indicators included in the expert consensus for quality control management of HPV testing? What is the difference in clinical management between ASC-US and ASC-H? Is 4-quadrant sampling still necessary for patients with no abnormalities on colposcopy? What are the differences in cervical cancer screening and management strategies for women during pregnancy?
Questions related to diagnosis	What is the preferred clinical diagnosis of cervical cancer? What tests should be conducted to make a pathologic diagnosis of cervical cancer? What tumor markers can be tested for laboratory diagnosis of cervical cancer? What is the significance of each tumor marker detected? In the diagnosis of cervical cancer, which imaging method should be preferred to evaluate cervical tumors? Which imaging method should be used to evaluate metastatic lesions? What are the recommended diagnostic tools for patients with FIGO^i^ stage IA1 cervical cancer? Is it necessary to consider lymphovascular infiltration? How are imaging tools used to assess tumor size and lymph node status in FIGO staging? What specific imaging tools are recommended?
Questions related to treatments	In cervical cancer treatment, if the patient does not wish to preserve fertility, and the stage is IA1 without lymphovascular space invasion, what treatment measures should be taken? In cervical cancer treatment, if the patient does not wish to preserve fertility, and the stage is IA1 with lymphovascular space invasion, what treatment measures should be taken? In cervical cancer treatment, if the patient does not wish to preserve fertility, and the stage is IA2, what treatment measures should be taken? In cervical cancer treatment, if the patient does not wish to preserve fertility, and the stage is IB1, IIA1, or IIB2, what treatment measures should be taken? In cervical cancer treatment, if the patient does not wish to preserve fertility, and the stage is IB3 or IIA2, what treatment measures should be taken? In cervical cancer treatment, if the patient wishes to preserve fertility, and the stage is IA1 without lymphovascular space invasion, what treatment measures should be taken? In cervical cancer treatment, if the patient wishes to preserve fertility, and the stage is IA1 with lymphovascular space invasion or IA2, what treatment measures should be taken? In cervical cancer treatment, if the patient wishes to preserve fertility, and the stage is IB1, what treatment measures should be taken? In cervical cancer treatment, if the patient wishes to preserve fertility, and the stage is IB2, what treatment measures should be taken? In cervical cancer treatment, if the stage is IIB, IIIA, or IIIB, what treatment measures should be taken? In cervical cancer treatment, if the stage is IIIC1, what treatment measures should be taken? In cervical cancer treatment, if the stage is IIIC2, what treatment measures should be taken? In cervical cancer treatment, if the stage is IVA without lymph node enlargement, what treatment measures should be taken? In cervical cancer treatment, if the stage is IVA with lymph node enlargement, what treatment measures should be taken? In cervical cancer treatment, if the stage is IVB, what treatment measures should be taken? After radical surgery for early cervical cancer, if the abdominal aortic lymph nodes are negative but high-risk factors are present, what treatment measures should be taken? After radical surgery for early cervical cancer, if the abdominal aortic lymph nodes are negative but intermediate-risk factors are present, what treatment measures should be taken? After radical surgery for early cervical cancer, if the abdominal aortic lymph nodes are positive but there is no distant metastasis, what treatment measures should be taken? What are the surgical treatment options for patients with FIGO stage IA1 cervical cancer? Are they suitable for patients with preserved fertility? In patients with FIGO stage IB2 and IIA1, what factors determine the choice between surgery and radiotherapy? What are the differences in outcomes between the 2 modalities? What is the recommended treatment of choice for FIGO stage IB3 cervical cancer? What is the difference between the different types of radical hysterectomy? For which patients is it indicated? What are the advantages of intensity-modulated radiation therapy in radiotherapy for cervical cancer? What are the common sites of recurrence in cervical cancer? How is the risk of recurrence monitored? What are the recommended treatment strategies for recurrent cervical cancer? Can surgery, radiotherapy, and chemotherapy be combined? What are the treatment strategies for metastatic cervical cancer? Are there specific treatments at different metastatic sites? What are the treatment strategies for patients with cervical cancer during pregnancy? How does the treatment differ in early, intermediate, and advanced stages of pregnancy? Are patients with cervical cancer in pregnancy suitable for surgery? At what stage of pregnancy should surgery be considered? What is the radiotherapy strategy for patients with locally advanced cervical cancer? When is a combination of chemotherapy recommended? When is image-guided brachytherapy necessary and how is it different from conventional radiotherapy? What are the methods for monitoring recurrence after treatment of cervical cancer? Is routine imaging recommended? What are the main goals of palliative care? In patients with metastatic cervical cancer, how can palliative care be combined with radiotherapy and chemotherapy?

^a^HPV: human papillomavirus.

^b^ASC-US: atypical squamous cells of undetermined significance.

^c^ASC-H: atypical squamous cells, cannot exclude high-grade squamous intraepithelial lesion

^d^LSIL: low-grade squamous intraepithelial lesion.

^e^HSIL: high-grade squamous intraepithelial lesion.

^f^AGC: atypical glandular cell.

^g^TZ3: Type 3 transformation zone.

^h^CIN: cervical intraepithelial neoplasia.

^i^FIGO: International Federation of Gynecology and Obstetrics.

Using the CO-STAR framework, the prompt was designed to guide the model in providing clinically relevant and detailed responses, meeting the standards necessary for accurate interpretation in cervical cancer management. The specific details of the prompt are presented in Table 4.

**Table 4:** Prompt designing based on the CO-STAR^a^ framework.

Prompt element	Content
# Context #	Now you are a gynecologist with over 20 years of experience in medicine and you are answering questions about the medical specialty of cervical cancer treatment, diagnosis, and screening.
# Objective #	Please answer the following questions correctly and in strict accordance with the latest guidelines for the screening, treatment, and diagnosis of cervical cancer.
# Style #	The information should be clear, concise, and medically accurate, using terminology appropriate for both health care professionals and patients.
# Tone #	The tone should be formal and professional, recognizing the sensitive nature of cancer-related discussions.
# Audience #	The primary audience includes health care professionals, researchers, and patients seeking information about cervical cancer management.
# Response #	Generate detailed responses to specific queries regarding cervical cancer. Assess the accuracy and relevance of the information provided.

^a^CO-STAR: Context, Objective, Style, Tone, Audience, and Response.

### Model Stability

Among the 9 models evaluated, 7 demonstrated good reproducibility with stable responses. However, the repeatability of Phi-2 and QiZhenGPT was unsatisfactory, as posing the same question 3 times often resulted in varying answers. For Phi-2, 61 out of 100 responses with the prompt and 68 responses without the prompt exhibited semantic differences across repetitions. Similarly, for QiZhenGPT, 60 responses with the prompt and 55 without the prompt varied. In both cases, pairwise comparisons were necessary to determine the final output (see Multimedia Appendix 2).

### Model Efficacy

**Figure 4:**
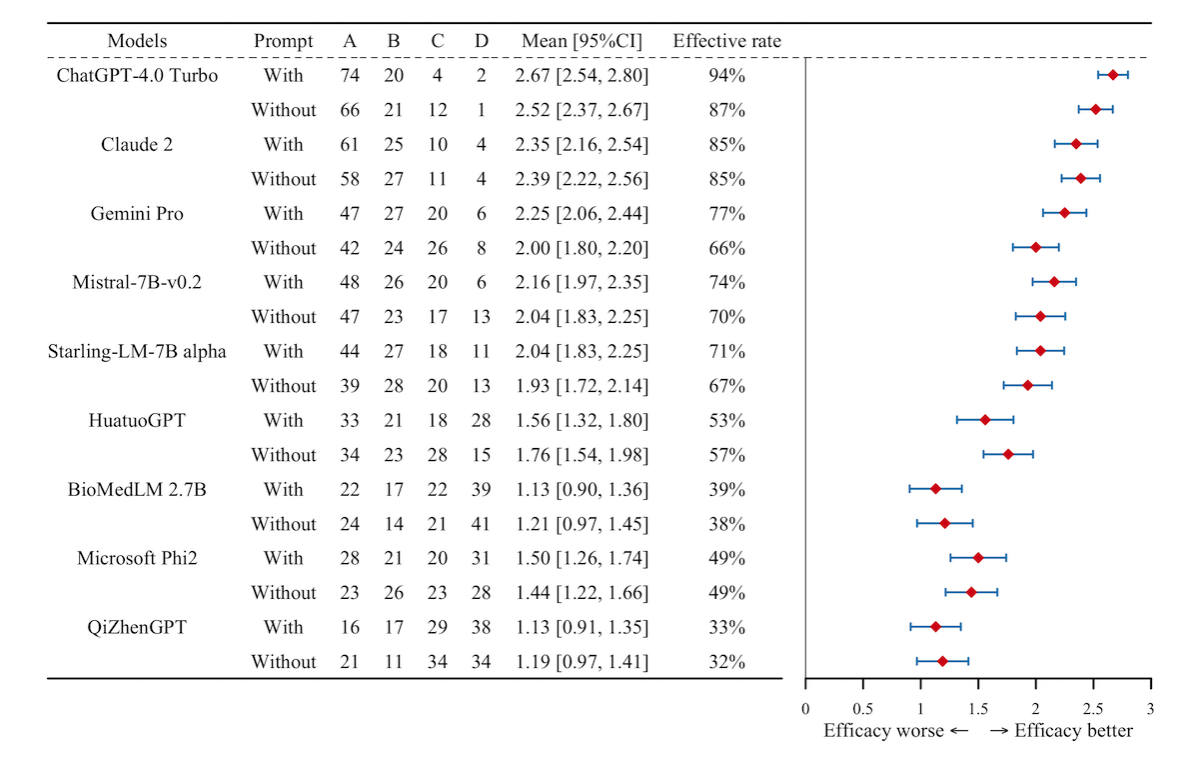
The efficacy assessment of each model with and without the prompt. The number in A, B, C and D represents the distribution of response quality in each grade. ChatGPT-4.0 Turbo achieved the highest effective rate (94% with a mean score of 2.67, 87% without at 2.52), while Claude 2 remained consistent at 85%. Gemini Pro improved from 66% to 77% with prompts. Among medically specialized models, HuatuoGPT slightly increased from 53% to 57% without prompts, BioMedLM stayed low (39% vs 38%), and QiZhenGPT had the lowest rates (33% vs 32%), showing minimal impact from prompts.

The evaluation results for each model, with and without the prompt, are presented in Figure 4. The top 3 performers were all proprietary models. ChatGPT-4.0 Turbo achieved the highest effective rate, at 94% (mean score 2.67, 95% CI 2.54-2.80) with the prompt and 87% (mean score 2.52, 95% CI 2.37-2.67) without it, highlighting the positive impact of the prompt on its performance. Claude 2 maintained an effective rate of 85% both with and without the prompt, with similar mean scores of 2.35 (95% CI 2.16-2.54) and 2.39 (95% CI 2.22-2.56), respectively. Gemini Pro showed moderate improvement, with its effective rate increasing from 66% (mean score 2.00, 95% CI 1.80-2.20) without the prompt to 77% (mean score 2.25, 95% CI 2.06-2.44) with the prompt.

By contrast, the 3 medically specialized models exhibited lower effective rates. HuatuoGPT achieved an effective rate of 53% (mean score 2.00, 95% CI 1.80-2.20) with the prompt, which unexpectedly increased to 57% (mean score 1.76, 95% CI 1.54-1.98) without it. BioMedLM showed minimal improvement, with an effective rate of 39% (mean score 1.13, 95% CI 0.90-1.36) with the prompt and 38% (mean score 1.76, 95% CI 1.54-1.98) without it. QiZhenGPT had the lowest performance, with an effective rate of 33% (mean score 1.13, 95% CI 0.91-1.35) with the prompt and 32% (mean score 1.19, 95% CI 0.97-1.41) without it, showing limited impact from the prompt on enhancing its responses. The STS testing results are provided in Multimedia Appendix 3. Detailed responses and original scoring are provided in Multimedia Appendix 4.

**Figure 5:**
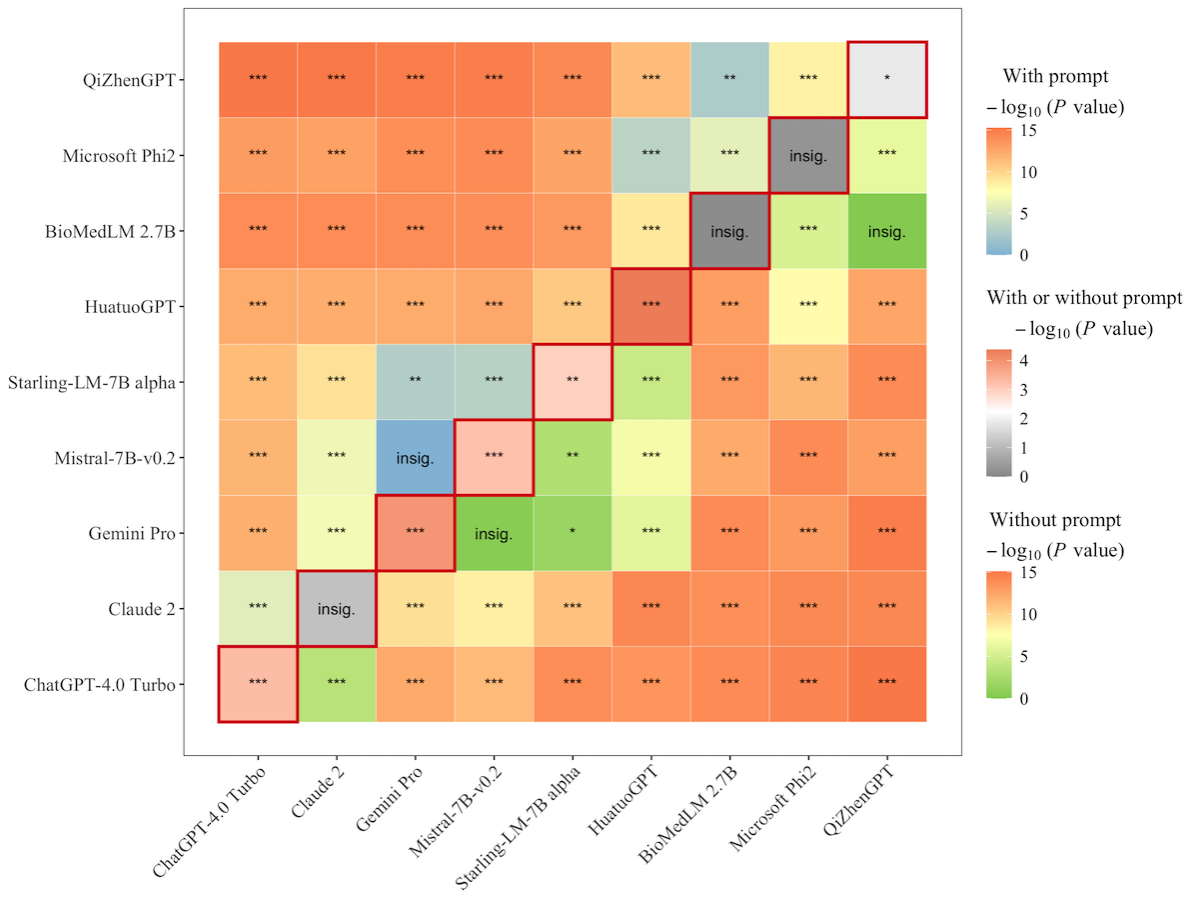
Pairwise significance comparison between models with and without the prompt. The upper triangle represents significance levels between models with the prompt, while the lower triangle displays significance levels without the prompt. The diagonal section shows significance of performance differences within each model between the prompted and unprompted conditions. The Wilcoxon rank sum test was performed as the data for each model did not follow a normal distribution. ChatGPT-4.0 Turbo and Claude 2 showed significant improvements (*P*<.001) with prompts, outperforming HuatuoGPT, BioMedLM, and QiZhenGPT. Without prompts, differences persisted but were less pronounced, especially between models such as Mistral-7B and Gemini Pro. insig.: insignificant; *P<.05; **P<.01; ***P<.001.

The chi-square test revealed significant differences across models (*P*=.001). As the data for each model did not follow a normal distribution (*P*<.01), the Wilcoxon rank sum test was applied. With the prompt, ChatGPT-4.0 Turbo and Claude 2 exhibited highly significant differences (*P*<.001) compared with most other models, indicating substantial performance enhancement when the prompt was used. This pattern remained consistent in comparisons with lower-performing models, such as HuatuoGPT, BioMedLM, and QiZhenGPT. Without the prompt, significant differences were still observed, particularly between high-performing models such as ChatGPT-4.0 Turbo (*P*<.001) and Claude 2 (*P*<.001) and lower-performing models. However, the absence of the prompt reduced significance in certain comparisons, such as between Mistral-7B and Gemini Pro (*P*=.30) or BioMedLM and QiZhenGPT (*P*=.64). When comparing performance with and without the prompt, ChatGPT-4.0 Turbo and Gemini Pro demonstrated statistically significant improvements with the prompt (*P*<.001), whereas Claude 2 showed no significant difference (*P*=.07). By contrast, models such as BioMedLM (*P*=.77), Phi-2 (*P*=.53), and QiZhenGPT (*P*=.01) exhibited minimal or insignificant changes (Figure 5).

### Model Explainability

**Figure 6:**
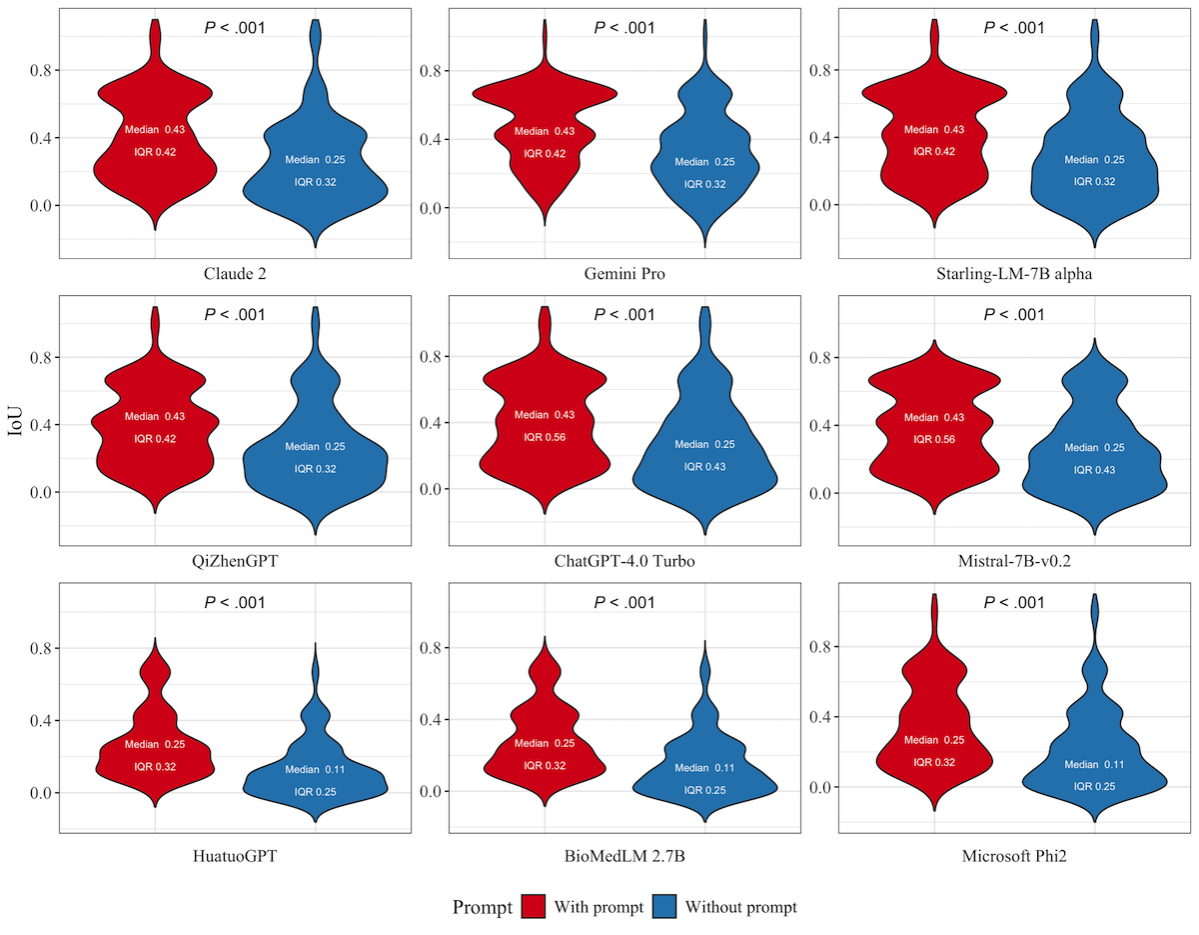
Model explainability analysis by intersection over union (IoU) for included models with or without the prompt. Data are expressed as median and IQR and the Wilcoxon rank sum test was applied due to nonnormal distribution within each model's data. Claude 2, Gemini Pro, Starling-LM-7B alpha, ChatGPT-4.0 Turbo, and Mistral-7B-v0.2 achieved a median IoU of 0.43 with prompts, dropping to 0.25 without prompt, with ChatGPT-4.0 Turbo showing the widest range (IQR 0.56). QiZhenGPT showed the most improvement among medical models with a median IoU of 0.43 with prompt. BioMedLM 2.7B and HuatuoGPT showed lower interpretability, with nonprompted IoUs of 0.11 (IQR 0.25).

Given the nonnormal distribution of IoU values for each model, the Wilcoxon rank sum test was used to assess differences. As shown in Figure 6, the inclusion of prompts significantly improved the alignment between model-generated explanations and human annotations, with all models exhibiting statistically significant differences between prompted and unprompted conditions (*P*<.001). Specifically, Claude 2, Gemini Pro, Starling-LM-7B Alpha, ChatGPT-4.0 Turbo, and Mistral-7B-v0.2 demonstrated a consistent median IoU of 0.43 with prompts. Among these, ChatGPT-4.0 Turbo had the widest IoU range (IQR 0.56). Without prompts, the median IoU for these models dropped to 0.25, with narrower IQRs ranging from 0.32 to 0.43, indicating reduced interpretability consistency. Among the medically specialized models, QiZhenGPT showed the most substantial improvement with prompts, achieving a median IoU of 0.43 (IQR 0.42), aligning it with the performance of proprietary models under similar conditions. By contrast, BioMedLM 2.7B and HuatuoGPT exhibited lower interpretability, with median IoUs of 0.29 and 0.25, respectively, and smaller IQRs in nonprompted conditions (median IoU of 0.11 and IQR of 0.25 for both).

## Discussion

### Principal Findings

This study systematically evaluated 9 LLMs for their performance, stability, and interpretability in cervical cancer management. The results revealed that proprietary models, such as ChatGPT-4.0 Turbo, Claude 2, and Gemini Pro, achieved superior response accuracy and interpretability, particularly with prompt guidance. By contrast, medically specialized models such as HuatuoGPT, QiZhenGPT, and BioMedLM demonstrated comparatively lower effectiveness, with limited improvement from prompt use. Notably, while proprietary models exhibited consistent reproducibility, certain open-source and specialized models, such as Phi-2 and QiZhenGPT, showed variable responses upon repeated questioning. Furthermore, the use of prompts significantly enhanced interpretability in models such as Claude 2, Gemini Pro, and Starling-LM-7B Alpha, highlighting the potential of structured input to improve alignment with clinical expectations.

### Comparison to Prior Work

In terms of average score ranking, proprietary models such as ChatGPT-4.0 Turbo, Claude 2, and Gemini Pro outperformed open-source models. This result aligns with traditional views on the superiority of proprietary systems [37]. However, without the prompt, Mistral-7B outperformed Gemini Pro. Among the open-source models, Mistral-7B-v0.2 and Starling-LM-7B Alpha outperformed HuatuoGPT and BioMedLM 2.7B. However, the repeatability of answers from Microsoft Phi-2 was poor, making it unsuitable for medical applications, while ChatGPT-4.0 Turbo and Claude 2 provided accurate and consistent responses. Our results indicated that the performance of the 3 medical models was average, challenging the prevailing belief that medical-specific models are superior for medical queries [38]. Previous studies [21,39] have shown that larger models, characterized by increased parameter counts, tend to perform better. Additionally, as the model scale increases, its generalization ability improves [40]. This may explain the relative underperformance of medical models compared with proprietary models, given the substantial disparity in parameter magnitude between them.

Recent advancements in algorithms have been shown to improve the performance of LLMs in the medical field [23,39], with research [41] indicating significant accuracy improvements using specific prompts. The integration of prompts has had a notable impact on the performance of several LLMs, emphasizing the value of structured input in guiding model responses within clinical contexts. Proprietary models, such as ChatGPT-4.0 Turbo and Gemini Pro, showed marked improvements in effective rate and response accuracy when guided by the CO-STAR prompt framework, suggesting that structured prompts help enhance focus on relevant clinical information and reduce ambiguity [42]. Conversely, models with specialized but limited training, such as BioMedLM, exhibited minimal sensitivity to prompts, likely due to architectural limitations in processing complex prompt structures [43]. Interestingly, HuatuoGPT experienced a decline in performance with the addition of prompts. This unexpected outcome suggests that the structured prompt for HuatuoGPT may have interfered with its response generation by introducing constraints that conflicted with its training data or underlying language patterns, potentially limiting its ability to accurately interpret open-ended clinical scenarios [44]. Additionally, smaller models often become confused when handling longer prompts [45]. The variation in prompt effectiveness across models underscores that, while structured prompts generally improve response precision, their impact is influenced by the model design and data scope.

The IoU serves as a robust indicator of alignment between model-generated explanations and human annotations, providing insights into the interpretability of LLMs in clinical contexts [46]. A higher IoU reflects greater consistency with human-provided explanations, suggesting enhanced model transparency and reliability in decision-making support. Our results demonstrate that a higher IoU corresponds to better alignment between model-generated explanations and human annotations, indicating improved interpretability. Proprietary models, particularly ChatGPT-4.0 Turbo and Claude-2, performed well in aligning with human explanations when prompts were used, highlighting their potential for generating clinically relevant interpretations. Interestingly, the rankings for model explainability based on IoU scores do not directly correlate with those based on effective rates. This discrepancy likely arises because improvements in model performance do not necessarily enhance explainability [47]. According to previous studies [48], as models become more accurate, their alignment with human-annotated explanations does not necessarily improve. This misalignment suggests that the factors driving a model’s effectiveness in task accuracy differ from those contributing to explainability. Higher-performing models may rely on complex, implicit patterns that are not fully captured by metrics such as IoU, which primarily assess agreement with human logic rather than the model’s actual reasoning process [49]. However, IoU alone may not fully capture explanation quality, as it can overlook aspects such as coherence and clinical relevance. Therefore, incorporating qualitative assessments alongside IoU could provide a more comprehensive measure of model explainability in clinical contexts.

### Ethical Issues

LLMs have performed well in the cervical cancer question-and-answer task, but ethical considerations, such as transparency, data privacy, and algorithmic bias, remain [50]. Tools such as LIME enhance transparency and simplify the explanation of AI decisions, with further progress expected [51]. Deployments adhere to strict data laws to ensure ongoing improvements in privacy, and technological advancements are anticipated to further safeguard patient privacy [52]. Bias issues are managed through explainable AI and methods such as training with multiple multiinstitutional or population data sets, as well as using generative adversarial networks to obtain more representative data [53]. While practical challenges remain in technology integration and staff training, LLMs are more easily adopted due to their application programming interfaces and their ability to act as personalized learning assistants, reducing the reliance on extensive medical staff training [54,55].

### Limitations

Our study also has limitations: (1) Because of the limited capabilities of our computers, we were unable to test all existing LLMs. It is possible that there are models with better performance than ChatGPT-4.0 Turbo in handling abnormal cervical screening results. (2) Our study did not include augmented algorithms or corpora that have been shown to improve LLM performance in other studies, as not all patients or physicians are familiar with these tools. The lack of these enhancements may limit the ability of LLMs to demonstrate their full potential in answering medical questions. This absence could have restricted the models from showcasing their full capabilities in medical query resolution, potentially affecting the generalizability of our results in more advanced settings. (3) The study conducted assessments under controlled, structured questions, which may not fully reflect the model’s performance in dynamic, real-world clinical settings. This controlled environment may limit our ability to assess the adaptability of LLMs in unpredictable or complex patient interactions.

### Conclusions

This study highlights the pivotal role of LLMs, particularly proprietary ones such as ChatGPT-4.0 Turbo, in enhancing clinical decision-making in cervical cancer screening. ChatGPT-4.0 Turbo outperforms both open-source and medical-specialized models in interpreting clinical guidelines and handling medical queries. Such findings are essential for improving the accuracy and efficiency of medical screenings and diagnoses, ultimately enhancing health care delivery and patient care. Further research is needed to assess the effectiveness of LLMs in medical applications, potentially leading to the development of models more tailored for medical practice and advancing overall health care.

## Data Availability

The 100 questions developed for model evaluation and all analyzed data in this study are included in the published manuscript and its multimedia appendices.

## Appendix

Multimedia Appendix 1 Computer specifications for deploying models.

Multimedia Appendix 2 Cervical cancer screening and abnormal result management process by the CSCCP (Chinese Society for Colposcopy and Cervical Pathology of the China Healthy Birth Science Association).

Multimedia Appendix 3 Semantic textual similarity testing for Phi-2 and QiZhenGPT model.

Multimedia Appendix 4 Responses from each model and quality assessments.

## Abbreviations

ASCCP: American Society for Colposcopy and Cervical Pathology
CO-STAR: Context, Objective, Style, Tone, Audience, and Response
CSCCP: Chinese Society for Colposcopy and Cervical Pathology of the China Healthy Birth Science Association
CSCO: Chinese Society of Clinical Oncology
FIGO: International Federation of Gynecology and Obstetrics
GEICO: Grupo Español de Investigación en Cáncer de Ovario
IoU: intersection over union
LIME: Local Interpretable Model-agnostic Explanations
LLM: large language model
SEOM: Sociedad Española de Oncología Médica
USMLE: United States Medical Licensing Examination
