# Supplementary material for: Performance Evaluation of Large Language Models in Cervical Cancer Management Based on a Standardized Questionnaire: Comparative Study
Source: J Med Internet Res. 2025 Feb 5;27:e63626. doi: 10.2196/63626 (PMC11840365; doi:10.2196/63626)
Supplement: Multimedia Appendix 1 [file jmir_v27i1e63626_app1.docx]

**Multimedia Appendix 1.** Computer specifications for deploying models

| **Component** | **Specification** |
| --- | --- |
| CPU | i7-11800H |
| RAM | 16GB DDR4 3200MHz |
| GPU | NVIDIA GeForce RTX 3080 Laptop (8GB) |
| System | Windows 10 Home 22H2 |
| Hard Drive | Micron 3400 MTFDKBA1T0TFH |
