# Supplementary material for: Performance Evaluation of Large Language Models in Cervical Cancer Management Based on a Standardized Questionnaire: Comparative Study
Source: J Med Internet Res. 2025 Feb 5;27:e63626. doi: 10.2196/63626 (PMC11840365; doi:10.2196/63626)
Supplement: Multimedia Appendix 2 [file jmir_v27i1e63626_app2.docx]

**
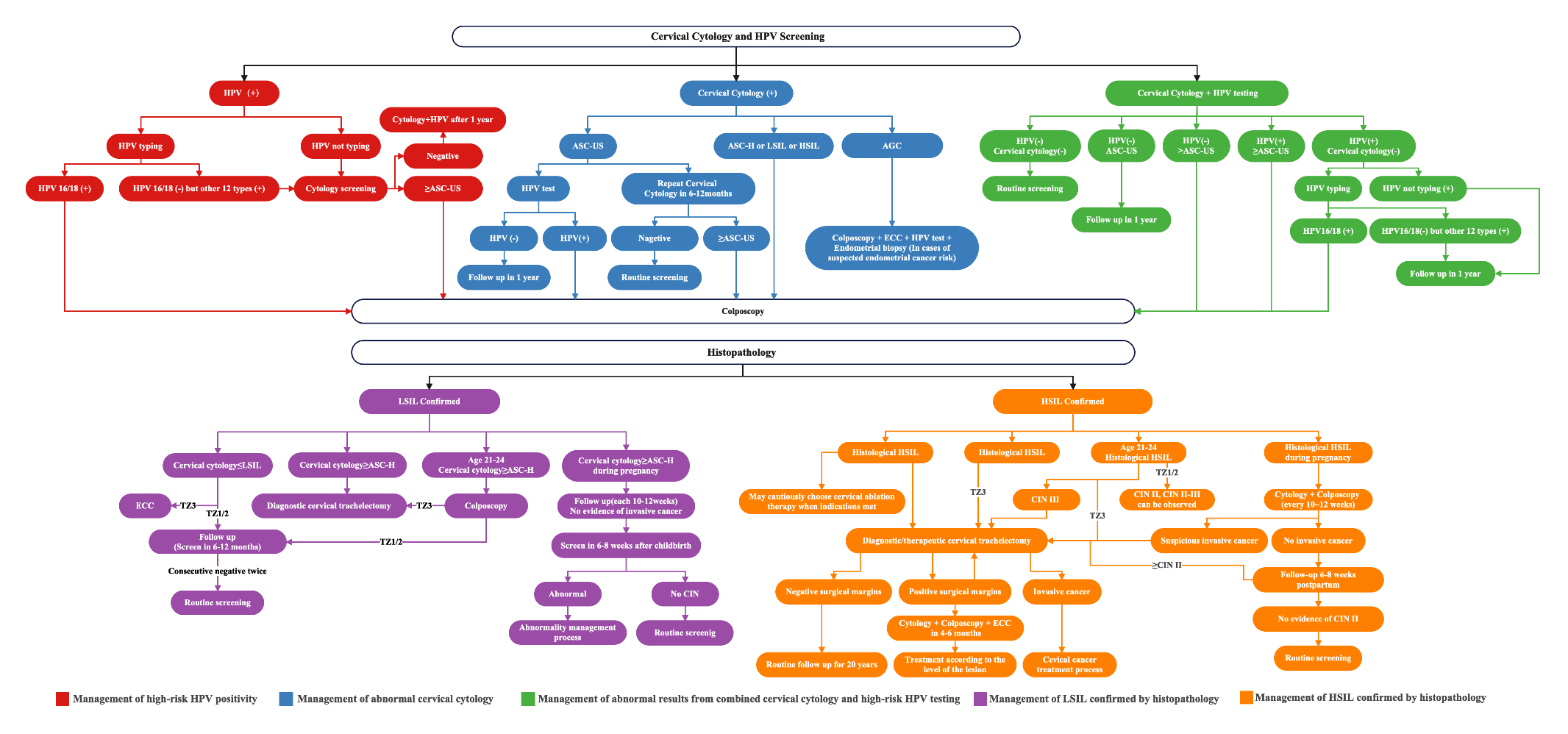
Multimedia Appendix 2.** Cervical Cancer Screening and Abnormal Result Management Process by CSCCP. HPV: Human Papillomavirus; ASC-US: Atypical Squamous Cells of Undetermined Significance; ECC: EndoCervical Curettage; LSIL: Low-grade Squamous Intraepithelial Lesion; HSIL: High-grade Squamous Intraepithelial Lesion; AGC: Atypical Glandular Cells; CIN: Cervical Intraepithelial Neoplasia; TZ: Transformation Zone.
