# Supplementary material for: Performance Evaluation of Large Language Models in Cervical Cancer Management Based on a Standardized Questionnaire: Comparative Study
Source: J Med Internet Res. 2025 Feb 5;27:e63626. doi: 10.2196/63626 (PMC11840365; doi:10.2196/63626)
Supplement: Multimedia Appendix 3 [file jmir_v27i1e63626_app3.docx]

**Multimedia Appendix 3.** Semantic textual similarity testing for Phi-2 and QiZhenGPT model

| **Model** | **Response** | | **Semantic textual similarity** | | | | | | **Final response** |
| --- | --- | --- | --- | --- | --- | --- | --- | --- | --- |
|  |  |  | **Response 1 vs 2** | **Response 1 vs 3** | | | | **Response 2 vs 3** |  |
| **1.What are the risk factors that may necessitate cervical cancer screening？** | | | | | | | | | |
| Phi-2  (with prompt) | **Response 1:** The risk factors that may necessitate cervical cancer screening include a history of human papillomavirus (HPV) infection, a weakened immune system, smoking, and a family history of cervical cancer.  **Response 2:** Risk factors that may necessitate cervical cancer screening include a history of human papillomavirus (HPV) infection, a weakened immune system, a history of sexually transmitted infections (STIs), a history of smoking, and a family history of cervical cancer.  **Response 3:**The risk factors that may necessitate cervical cancer screening include a history of sexually transmitted infections, a weakened immune system, and a family history of cervical cancer. | | 100 | 0 | | | | 0 | Response2 |
| Phi-2  (without promt) | The semantics of all three responses are identical | | - | - | | | | - | - |
| QiZhenGPT  (with prompt) | The semantics of all three responses are identical | | - | - | | | | - | - |
| QiZhenGPT  (without prompt) | The semantics of all three responses are identical | | - | - | | | | - | - |
| **2. At what age or under what conditions is cervical cancer screening typically deemed unnecessary?** | | | | | | | | | |
| Phi-2  (with prompt) | **Response 1:** Cervical cancer screening is typically deemed unnecessary for individuals who are over 65 years old, have had three consecutive negative Pap tests, and have had a hysterectomy with removal of the cervix.  **Response 2:** Cervical cancer screening is typically deemed unnecessary for individuals who are over the age of 65 and have had three consecutive normal Pap tests.  **Response 3:**Cervical cancer screening is typically deemed unnecessary for women who have had a hysterectomy, who are postmenopausal, or who have a history of cervical cancer that has been successfully treated. | | 100 | 85 | | | | 95 | Response2 |
| Phi-2  (without prompt) | **Response 1:** Cervical cancer screening is typically deemed unnecessary for individuals who are over the age of 65, have had a hysterectomy, or have had a negative Pap test in the past 10 years.  **Response 2:** Cervical cancer screening is typically deemed unnecessary for individuals who are over the age of 65 and have had three consecutive normal Pap tests, as well as for individuals who have been vaccinated against HPV and have had three consecutive normal Pap tests.  **Response 3:** Cervical cancer screening may be deemed unnecessary for individuals who are over the age of 65, have had a hysterectomy, or have had a negative Pap test in the past 10 years. | | 80 | 100 | | | | 70 | Response3 |
| QiZhenGPT  (with prompt) | **Response 1:** Cervical cancer screening is typically deemed unnecessary in:  * Women who have never been pregnant or have never had a menstrual period  * Women who have a low-risk profile (age 35 or older, no history of sexual violence or abuse, no history of cancer in a family member, no history of HIV infection)  * Women who have a history of sexual violence or abuse  * Women who have a history of cancer in a family member  * Women who are at high risk for developing cervical cancer due to their age, history of sexual violence or abuse, or a family history of cancer  **Response 2:** Cervical cancer screening is typically deemed unnecessary in:  * Women who have already been vaccinated against HPV  * Women who have had a previous colposcopy  * Women who have persistent with other nine types of high-risk human papillomavirus (hrHPV)  * Women who have high levels of human HPV DNA in their blood (high-risk HPV infection)  **Response 3:** Cervical cancer screening is typically considered unnecessary in the following situations:  * Age below 25 years,  * Condom use for 12 months or more,  * Pregnancy,  * Persistent or chronic infection with HPV-6 and HPV-11,  * Persistent or chronic infection with HPV-33, HPV-36, HPV-45, or HPV-51. | | 58 | 37 | | | | 45 | Response1 |
| QiZhenGPT  (without prompt) | **Response 1:** cervical cancer screening is considered unnecessary in the following situations:  * Age 16-19  * Pregnancy or planning to become pregnant  * Condom use history for more than 1 year  * Human Immunodeficiency Virus (HIV) infection  * Pregnancy or planning to become pregnant  * Age 50 or older  **Response 2:** cervical cancer screening is usually recommended for women who have a history ofdomdom use history,HIV infection, obesity, lack of physical activity, lack of education, poor diet, pregnancy, or age over 40 years old.  **Response 3:** cervical cancer screening is considered unnecessary in the following situations:  * Age 16-19  * Pregnancy or planning to become pregnant  * Condom use history for more than 1 year  * Human Immunodeficiency Virus (HIV) infection  * Pregnancy or planning to become pregnant  * Age 50 or older | | 0 | 100 | | | | 0 | Response1 |
| **3. What strategies are effective in reducing the risk of developing cervical cancer?** | | | | | | | | | |
| Phi-2  (with prompt) | The semantics of all three responses are identical | | - | - | | | | - | - |
| Phi-2  (without prompt) | The semantics of all three responses are identical | | - | - | | | | - | - |
| QiZhenGPT  (Without promt) | **Response 1:** Strategies to reduce the risk of developing cervical cancer include:  * Condom use  * Pregnancy prevention  * High-risk behavior prevention (such as sexual activity with a partner who has HIV or is HIV-RNA positive)  * Pregnancy prevention through early detection and prenatal care  * Pregnancy prevention through education and awareness campaigns  **Response 2:** Strategies that are effective in reducing the risk of developing cervical cancer include:  * Implementing a comprehensive HPV vaccination program that includes all women of reproductive age  * Implementing regular colposcopies in women who have already been vaccinated  * Improving access to healthcare for women in low-income countries  * Improving awareness and education about the risks and benefits of cervical cancer screening  **Response 3:** Strategies to reduce the risk of developing cervical cancer include:  * Implementing a comprehensive sexual health education program,  * Providing regular screening and testing for HPV infection,  * Implementing a policy of regular use of condoms,  * Providing access to affordable and effective treatment options for HPV-related cervical cancer. | | 70 | 80 | | | | 60 | Response1 |
| QiZhenGPT  (without promt) | **Response 1:** Strategies to reduce the risk of developing cervical cancer include:  * Condom use history for more than 1 year  * Human Immunodeficiency Virus (HIV) infection  * Pregnancy  * Age 16-19  * Family history of cervical cancer  **Response 2:** Strategies to reduce the risk of developing cervical cancer include:  * Using condoms consistently  * Avoiding risky behaviors such asdomdom use, smoking, and alcohol consumption  * Staying physically active  * Maintaining a healthy diet  * Avoiding pregnancy  **Response 3:** Strategies that can be effective in reducing the risk of developing cervical cancer include:  * Condom use: Condom use can reduce the likelihood of sexual intercourse, which is a risk factor for the development of cervical cancer.  * HPV vaccination: HPV vaccination can reduce the risk of developing cervical cancer by protecting against the virus that causes cervical cancer.  * Regular screening: Regular screening with a cervical test can detect pre-cancerous cells and prevent the development of cervical cancer. | | 0 | 100 | | | | 0 | Response1 |
| **4. What are the common clinical symptoms of cervical cancer?** | | | | | | | | | |
| Phi-2  (with promt) | The semantics of all three responses are identical | | - | - | | | | - | - |
| Phi-2  (without promt) | The semantics of all three responses are identical | | - | - | | | | - | - |
| QiZhenGPT  (with prompt) | **Response 1:** Common clinical symptoms of cervical cancer include:  * Persistent or recurrent menstrual bleeding  * Persistent or recurrent low-grade fever  * Persistent or recurrent cough  * Persistent or recurrent weight loss  **Response 2:** Common clinical symptoms of cervical cancer include:  * Cervical cancer symptoms can be subtle and may not be recognized until the disease is advanced  * Persistent or recurrent sore throat  * Persistent or recurrent cough  * Persistent or recurrent fever  * Persistent or recurrent weight loss  **Response 3:** Clinical symptoms of cervical cancer may include:  * A sore throat,  * Stoolstools with blood or white blood cells,  * Fatigue,  * Headache,  * Nausea and vomiting,  * Weakness,  * Dizziness,  * Difficulty breathing. | | 41 | 0 | | | | 26 | Response2 |
| QiZhenGPT  (without prompt) | **Response 1:** Common clinical symptoms of cervical cancer include:  * Cervical cancer can cause a low-grade fever  * A persistent, heavy menstrual flow  * Persistent, heavy bleeding  * Persistent, persistent, heavy spotting  * Persistent, persistent, persistent, heavy bleeding  **Response 2:** Clinical symptoms of cervical cancer include:  * Cervical cancer can cause a range of symptoms, including:  * A persistent, heavy menstrual flow  * A persistent, heavy, or light spotting or bleeding  * A low-grade fever  * A cough  * A sore throat  * A runny nose  **Response 3:** Common clinical symptoms of cervical cancer include:  * Cervical cancer can cause a range of symptoms, including:  * A persistent, heavy menstrual flow.  * A persistent, heavy, or light menstrual flow that does not stop.  * A persistent, heavy, or light bleeding after sexual intercourse.  * A persistent, heavy, or light bleeding that is accompanied by fever or other symptoms. | | 68 | 54 | | | | 100 | Response2 |
| **5. For individuals who have been vaccinated against HPV, is it still necessary for them to undergo cervical cancer screening?** | | | | | | | | | |
| Phi-2  (with prompt) | **Response 1:** For individuals who have been vaccinated against HPV, it is still necessary for them to undergo cervical cancer screening, as the vaccine does not provide 100% protection against all types of HPV that can cause cervical cancer.  **Response 2:** Even for individuals who have been vaccinated against HPV, it is still necessary for them to undergo cervical cancer screening as the vaccine does not provide 100% protection against all strains of HPV that can cause cervical cancer.  **Response 3:** Yes, cervical cancer screening is still recommended for individuals who have been vaccinated against HPV. While the vaccine can prevent certain types of HPV that cause cervical cancer, it does not protect against all types of HPV. Therefore, regular screening is still recommended to detect any potential abnormalities or changes in the cervix. | | 100 | 0 | | | | 0 | Response1 |
| Phi-2  (without prompt) | The semantics of all three responses are identical | | - | - | | | | - | - |
| QiZhenGPT  (with prompt) | **Response 1:** For individuals who have been vaccinated against HPV, it is generally not necessary for them to undergo cervical cancer screening. However, it is important to continue to use condoms and follow other risk-reducing strategies to prevent the development of HPV-related cancer.  **Response 2:** For individuals who have been vaccinated against HPV, it is generally not necessary for them to undergo cervical cancer screening. However, it is important for women who have not been vaccinated to continue to undergo regular colposcopies to monitor for persistent infections with other nine types of high-risk human papillomavirus (hrHPV) and to detect early-stage cervical cancer.  **Response 3:** For individuals who have been vaccinated against HPV, it is generally not necessary for them to undergo cervical cancer screening. However, it is important to follow the recommended schedule and interval for the vaccine to be administered, as well as to continue practicing good sexual health habits to reduce the risk of HPV-related cervical cancer. | | 0 | 100 | | | | 0 | Response3 |
| QiZhenGPT  (without prompt) | **Response 1:** For individuals who have been vaccinated against HPV, it is still necessary for them to undergo cervical cancer screening. HPV is a virus that causes cervical cancer. The vaccine does not prevent all cases of cervical cancer, but it can help reduce the risk of developing certain types of cancer by 50%. It is recommended that individuals who have been vaccinated against HPV undergo regular screening with cervical cancer tests to detect any changes in their cervical cells.  **Response 2:** For individuals who have been vaccinated against HPV, it is generally not necessary to undergo cervical cancer screening. HPV is a virus that causes many types of cancer in women, including cervical cancer. However, if you have already been vaccinated, it is important to continue using condoms consistently to protect against future infection of HPV.  **Response 3:** For individuals who have been vaccinated against HPV, it is still necessary for them to undergo cervical cancer screening, as the vaccine does not provide complete protection against cervical cancer. Regular screening with a cervical test can detect pre-cancerous cells and prevent the development of cervical cancer. | | 30 | 100 | | | | 70 | Response3 |
| **6.Is cervical cancer screening still recommended for individuals who have had only one sexual partner or are not currently sexually active?** | | | | | | | | | |
| Phi-2  (with prompt) | **Response 1:** Cervical cancer screening is still recommended for individuals who have had only one sexual partner or are not currently sexually active. This is because the risk of developing cervical cancer is not solely dependent on sexual activity, but also on other factors such as age, genetics, and exposure to certain viruses.  **Response 2:** Yes, cervical cancer screening is still recommended for individuals who have had only one sexual partner or are not currently sexually active, as they may still be at risk for developing cervical cancer due to other risk factors such as smoking or a weakened immune system.  **Response 3:** Yes, cervical cancer screening is still recommended for individuals who have had only one sexual partner or are not currently sexually active. While the risk of developing cervical cancer is lower in these individuals, regular screening is still recommended to detect any potential abnormalities or changes in the cervix. | | 100 | 71 | | | | 95 | Response2 |
| Phi-2  (without prompt) | **Response 1:** Cervical cancer screening is still recommended for individuals who have had only one sexual partner or are not currently sexually active.  **Response 2:** Yes, cervical cancer screening is still recommended for individuals who have had only one sexual partner or are not currently sexually active.  **Response 3:** No, cervical cancer screening is not recommended for individuals who have had only one sexual partner or are not currently sexually active. | | 100 | 0 | | | | 0 | Response1 |
| QiZhenGPT  (with prompt) | **Response 1:** Yes, cervical cancer screening is still recommended for individuals who have had only one sexual partner or are not currently sexually active.  **Response 2:** Yes, cervical cancer screening is still recommended for individuals who have had only one sexual partner or are not currently sexually active.  **Response 3:** Yes, cervical cancer screening is still recommended for individuals who have had only one sexual partner or are not currently sexually active. | | 80 | 0 | | | | 0 | Response1 |
| QiZhenGPT  (without prompt) | **Response 1:** Yes, cervical cancer screening is still recommended for individuals who have had only one sexual partner or are not currently sexually active.  **Response 2:** No, cervical cancer screening is not recommended for individuals who have had only one sexual partner or are not currently sexually active.  **Response 3:** Yes, cervical cancer screening is still recommended for individuals who have had only one sexual partner or are not currently sexually active. | | 0 | 100 | | | | 0 | Response3 |
| **7. What are the recommended intervals for cervical cancer screening, and do these intervals vary among different age groups?** | | | | | | | | | |
| Phi-2  (with prompt) | **Response 1:** The recommended intervals for cervical cancer screening vary among different age groups. For women aged 21-29, screening is recommended every three years. For women aged 30-65, screening is recommended every five years. For women aged 65 and older, screening is not recommended unless there are specific risk factors present.  **Response 2:** The recommended intervals for cervical cancer screening vary among different age groups. For individuals aged 21-29, screening is recommended every 3 years. For individuals aged 30-65, screening is recommended every 5 years. However, women with a history of abnormal Pap tests or high-risk HPV may require more frequent screening.  **Response 3:** The recommended intervals for cervical cancer screening vary among different age groups. For individuals aged 21-29, screening is recommended every 3 years. For individuals aged 30-65, screening is recommended every 5 years. However, it is important to note that these intervals may vary depending on individual risk factors and the results of previous screenings. | | 100 | 89 | | | | 80 | Response1 |
| Phi-2  (without prompt) | The semantics of all three responses are identical | | - | - | | | | - | - |
| QiZhenGPT  (with prompt) | **Response 1:** The recommended intervals for cervical cancer screening vary among different age groups. For example, the recommended interval for women aged 15-19 is 28-36 months, while for women aged 20-29, it is 36-48 months. These intervals should be adjusted based on individual risk factors and the stage of the disease.  **Response 2:** The recommended intervals for cervical cancer screening are 28-30 days, 6-12 months, and 12-24 months, respectively. These intervals do not vary among different age groups.  **Response 3:** The recommended intervals for cervical cancer screening vary among different age groups. For example, the recommended interval for women aged 15-19 is 28-36 months, while for women aged 20-29 it is 36-48 months. These intervals should be reviewed and updated periodically to reflect changes in population health and sexual behavior. | | 15 | 95 | | | | 20 | Response3 |
| QiZhenGPT  (without prompt) | The semantics of all three responses are identical | | - | - | | | | - | - |
| **8. Is cervical cancer screening universally recommended for all age groups? If not, what are the reasons for excluding certain age groups from undergoing cervical cancer screening?** | | | | | | | | | |
| Phi-2  (with prompt) | **Response 1:** Cervical cancer screening is not universally recommended for all age groups. Certain age groups, such as women over 65, may not require screening unless there are specific risk factors present. This is because the risk of developing cervical cancer decreases with age, and the benefits of screening may not outweigh the potential harms.  **Response 2:** Cervical cancer is a type of cancer that affects the cervix, which is the lower part of the uterus that connects to the vagina. It is caused by the human papillomavirus (HPV), which is a common sexually transmitted infection.  **Response 3:** No, cervical cancer screening is not universally recommended for all age groups. The American Cancer Society recommends that women aged 21 to 29 undergo cervical cancer screening every three years, while women aged 30 to 65 undergo screening every five years. Women aged 65 and older who have had regular screening in the past 10 years and have no history of cervical cancer or precancerous lesions may stop screening. The reasons for excluding certain age groups from undergoing cervical cancer screening include the low incidence of cervical cancer in younger women, the potential for false-positive results in younger women, and the potential for overdiagnosis and overtreatment in older women. | | 90 | 75 | | | | 80 | Response1 |
| Phi-2  (without prompt) | The semantics of all three responses are identical | | - | - | | | | - | - |
| QiZhenGPT  (with prompt) | The semantics of all three responses are identical | | - | - | | | | - | - |
| QiZhenGPT  (without prompt) | The semantics of all three responses are identical | | - | - | | | | - | - |
| **9. How necessary is cervical cancer screening for women who have undergone total hysterectomy?** | | | | | | | | | |
| Phi-2  (with prompt) | The semantics of all three responses are identical | | - | - | | | | - | - |
| Phi-2  (without prompt) | The semantics of all three responses are identical | | - | - | | | | - | - |
| QiZhenGPT  (with prompt) | **Response 1:** cervical cancer screening is necessary for women who have undergone total hysterectomy. These women may have difficulty accessing screening services due to their medical conditions.  **Response 2:** cervical cancer screening is necessary for women who have undergone total hysterectomy. The removal of the uterus does not remove the ability to detect early stages of cervical cancer, so screening is necessary to detect any remaining cancer cells.  **Response 3:** cervical cancer screening is necessary for women who have undergone total hysterectomy. Without screening, women with hysterectomy may develop cervical cancer at a later age, which can be difficult to detect and treat. | | 100 | 85 | | | | 80 | Response1 |
| QiZhenGPT  (without prompt) | **Response 1:** cervical cancer screening is necessary for women who have undergone total hysterectomy. Hysterectomy is a surgical procedure that removes the uterus, which is one of the two organs that are involved in the menstrual cycle. Women who have undergone total hysterectomy may not be able to have regular menstrual periods, which can increase their risk of developing cervical cancer. Therefore, screening for cervical cancer is recommended for women who have undergone total hysterectomy to help identify and treat any early-stage cases of the disease.  **Response 2:** cervical cancer screening is necessary for women who have undergone total hysterectomy. While women with total hysterectomy may no longer be able to have a Pap test or other screening tests, they may still need to be monitored for any changes in their health over time.  **Response 3:** cervical cancer screening is necessary for women who have undergone total hysterectomy. While total hysterectomy does not remove the ability to undergo cervical cancer screening, it may increase the risk of developing other cancers in the future. Women who have undergone hysterectomy may need to be rescreened every 5-10 years to ensure that they are still at risk of developing cervical cancer. | | 90 | 100 | | | | 80 | Response1 |
| **10.What is the significance of cervical cancer screening?** | | | | | | | | | |
| Phi-2  (with prompt) | **Response 1:** The significance of cervical cancer screening is to detect precancerous changes in the cervix before they develop into cancer. This allows for early intervention and treatment, which can improve outcomes and reduce the risk of death from cervical cancer.  **Response 2:** Women who have undergone total hysterectomy, which involves the removal of the uterus and cervix, are not recommended for cervical cancer screening. This is because the cervix is no longer present, and therefore cannot be affected by HPV.  **Response 3:** Cervical cancer screening is significant because it can detect precancerous lesions and cervical cancer at an early stage, when treatment is most effective. Early detection can also prevent the spread of cancer to other parts of the body and improve the chances of survival. | | 70 | 80 | | | | 90 | Response3 |
| Phi-2  (without promt) | **Response 1:** The significance of cervical cancer screening is to detect any abnormal changes in the cervix at an early stage, allowing for prompt treatment and prevention of cervical cancer.  **Response 2:** Cervical cancer screening is significant because it can help detect precancerous changes in the cervix before they develop into cancer. Early detection of cervical cancer can lead to more effective treatment and better outcomes for patients.  **Response 3:** Cervical cancer screening is significant as it can detect precancerous changes or early signs of cervical cancer, allowing for timely treatment and prevention of the disease. | | 60 | 80 | | | | 100 | Response2 |
| QiZhenGPT  (with prompt) | **Response 1:** The significance of cervical cancer screening is that it can help prevent the spread of the disease to the lungs and other parts of the body, as well as reduce the risk of developing advanced stages of the disease. Early detection and treatment can also improve the chances of survival for women with cervical cancer.  **Response 2:** The significance of cervical cancer screening is that it helps to prevent the spread of the disease to the rest of the body. Early detection of cervical cancer can also help to improve treatment outcomes and reduce the risk of death. Additionally, screening can help to identify women who may be at risk for developing cervical cancer due to their sexual history or other factors, which can help to improve their overall health and well-being.  **Response 3:** The significance of cervical cancer screening is that it helps to prevent the spread of the disease to the lungs and other parts of the body. Early detection of cervical cancer can also lead to prompt treatment, which can improve outcomes and reduce the risk of complications. Additionally, cervical cancer screening can help to identify women who may be at risk of developing the disease due to their sexual behavior or lifestyle, which can help to prevent the disease from developing in the first place. | | 90 | 95 | | | | 85 | Response3 |
| QiZhenGPT  (without prompt) | The semantics of all three responses are identical | | - | - | | | | - | - |
| **11. Why is the combined use of cytological screening (Pap smear) and Human Papillomavirus (HPV) testing not recommended for women aged 21 to 29?** | | | | | | | | | |
| Phi-2  (with prompt) | The semantics of all three responses are identical | | - | - | | | | - | - |
| Phi-2  (without prompt) | The semantics of all three responses are identical | | - | - | | | | - | - |
| QiZhenGPT  (with prompt) | The semantics of all three responses are identical | | - | - | | | | - | - |
| QiZhenGPT  (without prompt) | The semantics of all three responses are identical | | - | - | | | | - | - |
| **12. Is there an invariable link between HPV infection and the onset of cervical cancer?** | | | | | | | | | |
| Phi-2  (with prompt) | **Response 1:** There is an invariable link between HPV infection and the onset of cervical cancer, as HPV is the primary cause of cervical cancer. However, not all women with HPV infection will develop cervical cancer, and not all women with cervical cancer have HPV infection.  **Response 2:** The combined use of cytological screening (Pap smear) and HPV testing is not recommended for women aged 21 to 29 because the risk of false-positive results is high. This can lead to unnecessary follow-up tests and procedures, which can cause anxiety and discomfort for the patient.  **Response 3:** There is an invariable link between HPV infection and the onset of cervical cancer. HPV is a sexually transmitted infection that can cause changes in the cells of the cervix, leading to the development of cervical cancer. However, not all women who are infected with HPV will develop cervical cancer, and not all women who develop cervical cancer will have been infected with HPV. | | 0 | 84 | | | | 100 | Response2 |
| Phi-2  (without prompt) | The semantics of all three responses are identical | | - | - | | | | - | - |
| QiZhenGPT  (with prompt) | The semantics of all three responses are identical | | - | - | | | | - | - |
| QiZhenGPT  (without prompt) | The semantics of all three responses are identical | | - | - | | | | - | - |
| **13. How should one interpret an abnormal result from a cervical cancer screening test? Does such a result definitively indicate the presence of cervical cancer?** | | | | | | | | | |
| Phi-2  (with prompt) | **Response 1:** An abnormal result from a cervical cancer screening test does not definitively indicate the presence of cervical cancer. Such a result may indicate the presence of precancerous or cancerous cells, but further testing is required to confirm the diagnosis.  **Response 2:** An abnormal result from a cervical cancer screening test does not definitively indicate the presence of cervical cancer. It is important to remember that a Pap test is a screening test, and it is not a diagnostic test. An abnormal Pap test result may indicate the presence of precancerous changes in the cervix, but it does not necessarily mean that cancer is present. In order to confirm the presence of cervical cancer, further diagnostic tests, such as a biopsy, may be necessary.  **Response 3:** An abnormal result from a cervical cancer screening test does not definitively indicate the presence of cervical cancer. Such a result may indicate the presence of precancerous or cancerous cells, but further testing is required to confirm the diagnosis. | | 100 | 68 | | | | 88 | Response1 |
| Phi-2  (without prompt) |  | |  |  | | | |  |  |
| QiZhenGPT  (with prompt) | **Response 1:** When interpreting an abnormal result from a cervical cancer screening test, it is important to consider the number and type of abnormal cells, as well as the presence of any additional abnormalities. A abnormal result should be confirmed by a second test, such as a colposcopy or a DNA test, to ensure accurate diagnosis.  **Response 2:** When interpreting an abnormal result from a cervical cancer screening test, it is important to consider the number and type of abnormal cells, as well as the presence of other markers of cancer.  **Response 3:** When interpreting an abnormal result from a cervical cancer screening test, it is important to consider the number and type of abnormal cells, as well as the presence of any additional abnormalities. A single abnormal cell on a Pap smear does not definitively indicate the presence of cervical cancer. | | 94 | 88 | | | | 92 | Response2 |
| QiZhenGPT  (without prompt) | The semantics of all three responses are identical | | - | - | | | | - | - |
| **14. why yearly cervical cancer screening is not recommended?** | | | | | | | | | |
| Phi-2  (with prompt) | **Response 1:** Yearly cervical cancer screening is not recommended because the risk of developing cervical cancer is relatively low in most women, and the benefits of screening are outweighed by the potential harms, such as false positives and unnecessary follow-up procedures.  **Response 2:** Yearly cervical cancer screening is not recommended because the risk of developing cervical cancer is relatively low. The American Cancer Society recommends that women aged 21 to 65 years undergo cervical cancer screening every three years with a Pap test or every five years with a combination of a Pap test and an HPV test. Women aged 65 years or older who have had normal cervical cancer screening results in the past ten years may stop screening altogether.  **Response 3:** Yearly cervical cancer screening is not recommended because the risk of developing cervical cancer is relatively low, and the benefits of screening are not always clear. Additionally, screening can lead to false-positive results, which can cause unnecessary anxiety and lead to further testing and treatment. | | 78 | 56 | | | | 90 | Response3 |
| Phi-2  (without prompt) | **Response 1:** Yearly cervical cancer screening is not recommended because the risk of cervical cancer is relatively low during this age group. Additionally, the potential benefits of screening may not outweigh the potential harms, such as false-positive results and unnecessary interventions.  **Response 2:** Yearly cervical cancer screening is not recommended because the benefits of screening in this age group are not yet established. The decision to screen should be based on individual risk factors and the potential benefits and harms of screening.  **Response 3:** Yearly cervical cancer screening is not recommended because it can lead to unnecessary follow-up tests and procedures, such as colposcopy and biopsy, which may cause anxiety for the patient and result in false-positive findings. | | 0 | 0 | | | | 100 | Response2 |
| QiZhenGPT  (with prompt) | The semantics of all three responses are identical | | - | - | | | | - | - |
| QiZhenGPT  (without prompt) | The semantics of all three responses are identical | | - | - | | | | - | - |
| **15. What are the objectives of cervical cancer screening protocols?** | | | | | | | | | |
| Phi-2  (with prompt) | **Response 1:** The objectives of cervical cancer screening protocols are to detect precancerous lesions or early-stage cervical cancer, which can be treated effectively before they progress to more advanced stages.  **Response 2:** The objectives of cervical cancer screening protocols are to detect precancerous changes in the cervix and to diagnose cervical cancer at an early stage, when it is most treatable. Screening can help prevent the development of cervical cancer by identifying and treating precancerous changes before they become cancerous.  **Response 3:** The objectives of cervical cancer screening protocols are to detect precancerous or cancerous cells early, when they are most treatable, and to reduce the risk of developing cervical cancer. | | 100 | 0 | | | | 0 | Response1 |
| Phi-2  (without prompt) | **Response 1:** The objectives of cervical cancer screening protocols are to detect precancerous changes or abnormalities in the cervix early, when they are most treatable. By identifying these changes, healthcare providers can intervene and prevent the development of cervical cancer.  **Response 2:** The objectives of cervical cancer screening protocols are to detect precancerous changes or abnormalities in the cervix, identify women at high risk for developing cervical cancer, and provide timely treatment to prevent the progression of precancerous changes to cancer.  **Response 3:** The objectives of cervical cancer screening protocols are to detect and treat precancerous lesions before they progress to invasive cervical cancer, and to provide early detection and treatment of cervical cancer for those who are at high risk. | | 80 | 70 | | | | 75 | Response1 |
| QiZhenGPT  (with prompt) | **Response 1:** The objectives of cervical cancer screening protocols are to identify women at risk of developing cervical cancer, to prevent the development of precancerous changes in the cervix, and to detect and treat cervical cancer in early stages when it is more treatable. The goal of these protocols is to improve the health and quality of life of women with cervical cancer by reducing the risk of progression to advanced stages and improving the outcomes of treatment.  **Response 2:** The objectives of cervical cancer screening protocols are to identify women at risk of developing the disease, to prevent its onset, and to detect early stages of cancer so that treatment can be effective.  **Response 3:** The objectives of cervical cancer screening protocols are to detect and diagnose cervical cancer at an early stage, to prevent its development into advanced stages, and to reduce the burden of disease on healthcare systems. These goals can be achieved through a combination of cytological screening, HPV testing, and other forms of risk assessment and management. | | 76 | 63 | | | | 81 | Response3 |
| QiZhenGPT  (without prompt) | **Response 1:** The objectives of cervical cancer screening protocols are to reduce the risk of developing cervical cancer by identifying women who are at risk of the disease and providing them with regular screening and early detection. This can help to prevent the spread of the disease and reduce the number of cases that occur. The goal of these protocols is also to improve the quality of life for women with cervical cancer by providing them with support and treatment.  **Response 2:** The objectives of cervical cancer screening protocols are to identify women at high risk of developing cervical cancer, to prevent the development of this disease in those who are already infected with HPV, and to detect and treat early-stage cervical cancer in women who have already developed the disease. The goal of these protocols is to improve the lives of women with cervical cancer and to reduce the burden of this disease on healthcare systems.  **Response 3:** The objectives of cervical cancer screening protocols are to identify women at risk of developing the disease, to detect and treat the disease early, and to prevent its spread to other parts of the body. The goals of these protocols are to improve outcomes for women with cervical cancer and to reduce the burden of the disease on healthcare systems. | | 85 | 100 | | | | 75 | Response1 |
| **16.Is cervical cancer hereditary? If so, should individuals with a familial history of cervical cancer be subject to more frequent screening protocols?** | | | | | | | | | |
| Phi-2  (with prompt) | **Response 1:** Cervical cancer can be hereditary, particularly in women with a family history of the disease. Individuals with a familial history of cervical cancer may be subject to more frequent screening protocols to detect precancerous lesions or early-stage cervical cancer.  **Response 2:** Cervical cancer is not hereditary, but some individuals may have a familial history of cervical cancer that puts them at a higher risk of developing the disease. If an individual has a family history of cervical cancer, they may be advised to undergo more frequent screening protocols to detect the disease at an early stage.  **Response 3:** Yes, cervical cancer can be hereditary. Individuals with a familial history of cervical cancer may be at increased risk of developing the disease, and may benefit from more frequent screening protocols. | | 92 | 88 | | | | 85 | Response2 |
| Phi-2  (without prompt) | **Response 1:** Yes, cervical cancer can be hereditary. Individuals with a familial history of cervical cancer should be subject to more frequent screening protocols to detect any potential abnormalities at an early stage.  **Response 2:** Yes, cervical cancer can be hereditary. Individuals with a familial history of cervical cancer should be subject to more frequent screening protocols.  **Response 3:** Yes, cervical cancer can be hereditary, and individuals with a familial history of cervical cancer are at an increased risk of developing the disease. If an individual has a family history of cervical cancer, it is recommended that they undergo more frequent screening protocols, such as annual Pap tests or HPV testing. | | 100 | 85 | | | | 90 | Response2 |
| QiZhenGPT  (with prompt) | The semantics of all three responses are identical | | - | - | | | | - | - |
| QiZhenGPT  (without prompt) | The semantics of all three responses are identical | | - | - | | | | - | - |
| **17. What's the difference between a pelvic exam and a Pap test?** | | | | | | | | | |
| Phi-2  (with prompt) | **Response 1:** A pelvic exam is a physical examination of the female reproductive organs, including the uterus, ovaries, and vagina. A Pap test, on the other hand, is a screening test that detects abnormal cells on the cervix, which may indicate the presence of precancerous lesions or early-stage cervical cancer.  **Response 2:** A pelvic exam is a physical examination of the female reproductive system, including the cervix, uterus, and ovaries. A Pap test is a screening test that is used to detect changes in the cells of the cervix that may indicate the presence of precancerous changes or cervical cancer. During a Pap test, a sample of cells is collected from the cervix and examined under a microscope to look for abnormal changes.  **Response 3:** A pelvic exam is a physical examination of the female reproductive organs, including the cervix, uterus, and ovaries. A Pap test is a screening test that involves collecting cells from the cervix and examining them under a microscope for signs of precancerous or cancerous cells. | | 97 | 0 | | | | 0 | Response2 |
| Phi-2  (without prompt) | **Response 1:** A pelvic exam involves a physical examination of the female reproductive organs, including the cervix, vagina, and uterus. On the other hand, a Pap test specifically focuses on collecting cells from the cervix to detect any abnormal changes or signs of cervical cancer.  **Response 2:** A pelvic exam is a physical examination of the female reproductive organs, while a Pap test is a test that examines cells from the cervix for abnormalities.  **Response 3:** A pelvic exam involves a health care provider examining the external genitals, the vagina, and the internal reproductive organs through a pelvic examination. A Pap test involves collecting cells from the cervix to be examined for abnormalities that may indicate the presence of cervical cancer or other precancerous conditions. | | 76 | 86 | | | | 91 | Response3 |
| QiZhenGPT  (with prompt) | The semantics of all three responses are identical | | - | - | | | | - | - |
| QiZhenGPT  (without prompt) | **Response 1:** A pelvic exam is a physical examination that involves the examination of the reproductive organs, including the uterus, fallopian tubes, and ovaries. A Pap test, on the other hand, is a screening test that uses a small sample of cells from the skin to check for abnormal cells that may indicate the presence of cervical cancer.  **Response 2:** A pelvic exam is a physical examination that involves the examination of the reproductive organs, including the uterus, fallopian tubes, and ovaries. A Pap test, on the other hand, is a screening test that uses a small sample of tissue from the skin to check for abnormal cells that may indicate the presence of cervical cancer.  **Response 3:** A pelvic exam is a physical examination that involves the examination of the reproductive organs and the surrounding tissues. A Pap test, on the other hand, is a screening test that uses a small sample of cells from the skin to check for changes that may indicate the presence of cervical cancer. | | 89 | 100 | | | | 78 | Response1 |
| **18. Can individuals independently administer HPV tests, and if so, how accurate are these self-administered test?** | | | | | | | | | |
| Phi-2  (with prompt) | **Response 1:** Yes, individuals can independently administer HPV tests, and these self-administered tests are highly accurate.  **Response 2:** Yes, individuals can independently administer HPV tests, and the accuracy of these self-administered tests is comparable to that of professionally conducted tests.  **Response 3:** Yes, individuals can independently administer HPV tests, and these self-administered tests are highly accurate. | | 55 | 78 | | | | 68 | Response3 |
| Phi-2  (without prompt) | The semantics of all three responses are identical | | - | - | | | | - | - |
| QiZhenGPT  (with prompt) | The semantics of all three responses are identical | | - | - | | | | - | - |
| QiZhenGPT  (without prompt) | The semantics of all three responses are identical | | - | - | | | | - | - |
| **19. Is it possible for cervical cancer to manifest within the inter-screening interval, particularly between two consecutive cervical screening tests?** | | | | | | | | | |
| Phi-2  (with prompt) | **Response 1:** Yes, it is possible for cervical cancer to manifest within the inter-screening interval, particularly between two consecutive cervical screening tests.  **Response 2:** Yes, it is possible for cervical cancer to manifest within the inter-screening interval, particularly between two consecutive cervical screening tests.  **Response 3:** Yes, it is possible for cervical cancer to manifest within the inter-screening interval, particularly between two consecutive cervical screening tests. | | 60 | 80 | | | | 30 | Response1 |
| Phi-2  (without prompt) | The semantics of all three responses are identical | | - | - | | | | - | - |
| QiZhenGPT  (with prompt) | The semantics of all three responses are identical | | - | - | | | | - | - |
| QiZhenGPT  (without prompt) | The semantics of all three responses are identical | | - | - | | | | - | - |
| **20. Is cervical screening necessary for individuals who have reached menopause?** | | | | | | | | | |
| Phi-2  (with prompt) | **Response 1:** No, cervical screening is not necessary for individuals who have reached menopause.  **Response 2:** No, cervical screening is not necessary for individuals who have reached menopause, as this age group is at a very low risk of developing cervical cancer.  **Response 3:** No, cervical screening is not necessary for individuals who have reached menopause. | | 100 | 0 | | | | 0 | Response1 |
| Phi-2  (without prompt) | The semantics of all three responses are identical | | - | - | | | | - | - |
| QiZhenGPT  (with prompt) | The semantics of all three responses are identical | | - | - | | | | - | - |
| QiZhenGPT  (without prompt) | **Response 1:** Yes, cervical screening is necessary for individuals who have reached menopause. At this stage of life, the risk of developing cervical cancer decreases, but it is still important to undergo regular screening to detect any abnormal cells that may still be present.  **Response 2:** For individuals who have reached menopause, there is no need for cervical screening. However, it is important for these individuals to continue to follow up with a healthcare provider to ensure that they are taking care of their health and to detect any potential health issues early.  **Response 3:** cervical screening is necessary for individuals who have reached menopause. As the body begins to lose its ability to produce hormones, it is more likely to develop cervical cancer. Therefore, it is important for these individuals to undergo regular screening to detect the disease early and prevent its progression. | | 22 | 100 | | | | 56 | Response3 |
| **21. Is it recommended to undergo cervical screening during pregnancy?** | | | | | | | | | |
| Phi-2  (with prompt) | **Response 1:** No, cervical screening is not recommended during pregnancy.  **Response 2:** It is not recommended to undergo cervical screening during pregnancy, as this can interfere with the accuracy of the test and potentially harm the developing fetus.  **Response 3:** No, it is not recommended to undergo cervical screening during pregnancy. | | 100 | 0 | | | | 0 | Response1 |
| Phi-2  (without prompt) | The semantics of all three responses are identical | | - | - | | | | - | - |
| QiZhenGPT  (with prompt) | The semantics of all three responses are identical | | - | - | | | | - | - |
| QiZhenGPT  (without prompt) | The semantics of all three responses are identical | | - | - | | | | - | - |
| **22. What procedures are typically involved in cervical cancer screening?** | | | | | | | | | |
| Phi-2  (with prompt) | **Response 1:** Cervical cancer screening typically involves a Pap smear, which involves collecting cells from the cervix and examining them under a microscope for abnormalities. In some cases, additional tests such as HPV testing or colposcopy may be necessary.  **Response 2:** Cervical cancer screening typically involves a combination of Pap smears and HPV tests, as well as biopsies to determine the presence of abnormal cells.  **Response 3:** Cervical cancer screening typically involves a Pap smear, which involves collecting cells from the cervix and examining them for abnormalities. In some cases, HPV testing may also be performed. | | 92 | 0 | | | | 0 | Response2 |
| Phi-2  (without prompt) | **Response 1:** Cervical cancer screening typically involves a Pap smear, which involves collecting cells from the cervix for examination under a microscope.  **Response 2:** Cervical cancer screening typically involves a pelvic examination, a Pap smear, and an HPV test.  **Response 3:** Cervical screening typically involves a series of tests, including a visual inspection of the cervix using a speculum, followed by a pap smear to collect cells from the cervix for analysis. If any abnormal findings are detected, further investigations, such as colposcopy or biopsy, may be necessary. | | 80 | 70 | | | | 75 | Response1 |
| QiZhenGPT  (with prompt) | **Response 1:** The most common procedures involved in cervical cancer screening include:  a. Cervical screening tests: These tests are used to detect abnormal cells in the cervical mucus before pregnancy. They include the Papanville Test, the Human Immunodeficiency Virus (HIV) test, and the Human Immunodeficiency Virus (HIV) and Human Immunodeficiency Virus (HIV)-RNA test.  b. Cervical mucus analysis: This involves analyzing the cervical mucus to detect abnormal cells or abnormal DNA.  c. Biopsy: A biopsy is performed when the screening test is positive for cervical cancer. It involves taking a small sample of the cervical mucus and sending it to a laboratory for analysis.  d. Condom use: Condom use has been shown to reduce the risk of cervical cancer by 50%.  e. Follow-up screening: After the initial screening test, women should be followed up with a repeat screening test every 10 years or when they are 50 years old.  Note: These are the most common procedures involved in cervical cancer screening. The exact procedures may vary depending on the specific type of screening test and the location of the practice.  **Response 2:** The most common procedures involved in cervical cancer screening include:  a. Cervical scraping: This involves taking a small scraping of the Cervical cells from the surface of the uterus using a thin, flexible instrument called a uterine scraping.  b. Uterine  c. Biopsy: A biopsy is a diagnostic test that involves taking a small sample of the tissue or cells from the uterus for examination under a microscope. This is typically done to diagnose cervical cancer or to determine the stage of the disease.  d. Immunization: Immunization is a series of shots that are given to women to prevent the development of cervical cancer.  e. Follow-up: Follow-up screening is typically recommended every 3 years after the first negative screening test, and then every 5 years after that.  f. Pregnancy: Pregnancy does not increase the risk of developing cervical cancer, but it does increase the risk of developing other types of cancer in the future. Therefore, women who are planning to become pregnant should undergo regular screening to detect any early signs of cancer.  **Response 3:** The most common procedures involved in cervical cancer screening include:  a. Cervical screening tests: These tests are used to detect the presence of human cervical cancer cells in the uterus. They include the Papanville test, the Cervical Cancer screening test (CCT), and the Human Immunodeficiency Virus (HIV) test.  b. Biopsy: A biopsy is a procedure in which a small sample of tissue is taken for examination under a microscope. This is typically used to diagnose cervical cancer and to determine the stage of the disease.  c. Immunization: Immunizations are a series of shots that are given to women to prevent the transmission of cervical cancer.  d. Follow-up screening: Women who have undergone cervical screening tests and are found to have abnormal results may need to undergo additional screening tests over time to detect any changes in the status of the disease. | | 60 | 50 | | | | 70 | Response3 |
| QiZhenGPT  (without prompt) | **Response 1:** The recommended procedures for cervical cancer screening during pregnancy include:  * Cervical cancer screening tests: These are typically performed on a routine basis, such as every 12 weeks or every 36 weeks, depending on the pregnancy stage.  * Pre- Birth screening: This is a series of tests that are performed before pregnancy, including a Papanella test, a cervical culture, and a human growth hormone (GH) test.  * Mid-term screening: This is a series of tests that are performed at mid-term pregnancy, including a Papanella test, a cervical culture, and a human growth hormone (GH) test.  * Post- Birth screening: This is a series of tests that are performed after birth, including a Papanella test, a cervical culture, and a Human Immunodeficiency Virus (HIV) test.  It's important to note that these tests are not 100% effective in detecting cervical cancer, and a positive test result does not guarantee the presence of cancer. A healthcare provider will discuss the appropriate testing schedule and options with you. 1. Risk factors for cervical cancer include:  * Condom use history  * Human Immunodeficiency Virus (HIV) infection  * Pregnancy  * age  * family history of cervical cancer  **Response 2:** The recommended procedures for cervical cancer screening during pregnancy include:  * Cervical cancer screening tests: These are typically performed on a routine basis, usually at around 12-18 weeks of pregnancy. They include a simple blood test to check for the presence of human chorionic gonadotropin (hCG) and a colposcopy, which is a visual examination of the inside of the uterus using a light and a camera.  * colposcopy: This is the most common screening test used during pregnancy. It allows for the visualization of the inside of the uterus and can help identify any abnormalities or changes that may indicate the presence of cervical cancer.  * Human chorionic gonadotropin (hCG) test: This test is used to check for the presence of hCG, which is a hormone produced by the placenta during pregnancy. If hCG is detected, it can indicate that the pregnancy is ongoing and the woman should have a colposcopy as described above.  It is important to note that not all cervical cancer cases are detected during pregnancy, and early detection can help prevent the spread of the disease. Therefore, it is important for women to continue to follow up with cervical screening tests, even after the delivery of their baby.  **Response 3:** The recommended screening procedures for cervical cancer in pregnancy are:  * Cervical cancer screening in pregnancy: The first-line screening test for cervical cancer in pregnancy is the human Papillomavirus (Hpv) 13 test. This test is usually done at 12 weeks of pregnancy and is recommended every 2 years after the first trimester.  * Cervical cancer screening during pregnancy: The second-line screening test for cervical cancer in pregnancy is the human Papillomavirus (Hpv) 16 test. This test is usually done at 16 weeks of pregnancy and is recommended every 4 years after the first trimester.  * Cervical cancer screening in pregnancy: The third-line screening test for cervical cancer in pregnancy is the colposcopies. These tests are performed through a flexible, thin camera that is inserted into the uterus and can detect early stages of cervical cancer.  It's important to note that not all women will experience symptoms of cervical cancer during pregnancy, so it's important to continue screening even if you don't experience any symptoms. Additionally, it's important to talk to your healthcare provider about your options and to understand the risks and benefits of each screening test. | | 50 | 80 | | | | 100 | Response2 |
| **23.What is the examination process for HPVpositive highrisk types?** | | | | | | | | | |
| Phi-2  (with prompt) | The semantics of all three responses are identical | | - | - | | | | - | - |
| Phi-2  (without prompt) | **Response 1：**Examination Process for HPV-Positive High-Risk Types:  - Women who test positive for high-risk HPV types should be evaluated further since these types are associated with an increased risk of cervical cancer.  - The follow-up typically involves cytological testing (Pap smear) to detect any cellular abnormalities in the cervix.  **Response 2：**The examination process starts with HPV testing. If high-risk types are detected, further examination is warranted.  **Response 3：**The examination process for HPV-positive high-risk types involves a combination of cytological examination and HPV genotyping. | | 40 | 50 | | | | 30 | Response 3 |
| QiZhenGPT  (with prompt) | The semantics of all three responses are identical | | - | - | | | | - | - |
| QiZhenGPT  (without prompt) | The semantics of all three responses are identical | | - | - | | | | - | - |
| **24.When the initial screening shows positive HPV with highrisk HPV types emphasizing that HPV is not typed, what is the next step in the examination?** | | | | | | | | | |
| Phi-2  (with prompt) | **Response 1:** When the initial screening shows positive HPV with high-risk HPV types emphasizing that HPV is not typed, the next step in the examination is a colposcopy.  **Response 2:** If the initial screening shows positive HPV with high-risk HPV types and the cytological examination result is negative, it indicates that the patient is HPV-negative. Regular check-ups should follow, and the frequency should be determined based on the patient's age and risk factors.  **Response 3:** When the initial screening shows positive HPV with high-risk HPV types emphasizing that HPV is not typed, the next step in the examination is to perform a colposcopy. | | 36 | 100 | | | | 33 | Response3 |
| Phi-2  (without prompt) | **Response 1:** Once the specific type of HPV is identified through these tests, the healthcare provider will assess whether there are any signs of abnormal changes (such as cervical intraepithelial neoplasia grade 2 or higher) on the cervix. This assessment helps determine the need for further intervention or treatment. **Response 2:** When the initial screening shows positive HPV with high-risk HPV types, since HPV is not typed, the next step in the examination is to perform a follow-up colposcopy. This examination helps evaluate the abnormalities detected during the initial screening to determine if further actions are necessary, such as HPV DNA testing of cervical specimens. **Response 3:** The next step in the examination is to perform a colposcopy, which is a procedure that allows the doctor to examine the cervix in more detail. | | 40 | 10 | | | | 20 | Response 1 |
| QiZhenGPT  (with prompt) | **Response 1:** If the initial screening shows positive HPV with high-risk HPV types and HPV is not typed, the next step would be to perform a colposcopy to visualize the Cervical Cavity.  **Response 2:** If the initial screening shows positive HPV with high-risk HPV types and HPV is not typed, the next step would be to perform a colposcopy to visualize the Cervical Cavity.  **Response 3:** If the initial screening shows positive HPV with high-risk HPV types and HPV is not typed, the next step would be to perform a colposcopy to visualize the Cervical Cancer-related lesions. | | 100 | 78 | | | | 85 | Response 1 |
| QiZhenGPT  (without prompt) | The semantics of all three responses are identical | | - | - | | | | - | - |
| **25.When the initial screening shows positive HPV with highrisk HPV types and the cytological examination result is negative, what does it indicate? Should regular checkups follow and if so, what should be the frequency?** | | | | | | | | | |
| Phi-2  (with prompt) | The semantics of all three responses are identical | | - | - | | | | - | - |
| Phi-2  (without prompt) | **Response 1:** If the cytological examination result is negative, indicating no abnormalities, then regular check-ups can usually continue at the recommended intervals based on age, risk factors, and previous test results. However, it's important to note that even with a negative result, ongoing surveillance for potential changes remains crucial due to the long latency period associated with HPV infections.  **Response 2:** When the initial screening shows positive HPV with high-risk HPV types, and the cytological examination result is negative, it indicates that any abnormal cells found during the screening were not cancerous or precancerous. Regular check-ups are still necessary to monitor any potential changes in cervical health and to intervene if abnormalities occur in the future. **Response 3:** If the initial screening shows positive HPV with high-risk HPV types, and the cytological examination result is negative, it indicates that the patient is at a higher risk of developing cervical cancer. Regular check-ups should follow, and the frequency should be determined by the healthcare provider based on the patient's individual risk factors and medical history. | | 80 | 30 | | | | 20 | Response 1 |
| QiZhenGPT  (with prompt) | **Response 1:** If the initial screening shows positive HPV with high-risk HPV types and the cytological examination result is negative, it indicates that the sample is not suitable for analysis and regular check-ups should follow. The frequency of check-ups depends on the individual patient's risk factors and the stage of the disease.  **Response 2:** If the initial screening shows positive HPV with high-risk HPV types and the cytological examination result is negative, it indicates that the sample is not enough to detect any cervical cancer. Regular check-ups should follow, and the frequency depends on the individual's age and risk factors.  **Response 3:** If the initial screening shows positive HPV with high-risk HPV types and the cytological examination result is negative, it indicates that the screening process has been successful in identifying low-risk HPV types. Regular check-ups may be recommended, but the frequency would depend on the individual patient's risk factors and the presence of any symptoms. | | 92 | 82 | | | | 84 | Response1 |
| QiZhenGPT  (without prompt) | **Response 1:** If the initial screening shows HPV positivity and the cytology is negative, this may mean that the infection is mild or the viral load is low. Further testing is usually not needed, but regular follow-up is indicated. The frequency of follow-up depends on the circumstances and is usually every 6-12 months. **Response 2:** if the initial screen is positive for HPV and a high-risk type and the cytology is negative, this may mean that the HPV infection is mild or that cancer has not developed. Regular follow-up is usually needed, the frequency of which depends on the circumstances. **Response 3:** If the initial screening result is HPV-positive and in the high-risk category and the cytology is negative, this may indicate a mild infection or absence of lesions. However, regular follow-up is recommended to ensure that the risk of lesions is low. Cervical cytology is usually recommended every 6-12 months. | | 40 | 80 | | | | 70 | Response 3 |
| **26.When the initial screening shows positive HPV with highrisk HPV types and the cytological examination result is ≥ASCUS, is the next step to perform a colposcopy?** | | | | | | | | | |
| Phi-2  (with prompt) | The semantics of all three responses are identical | | - | - | | | | - | - |
| Phi-2  (without prompt) | **Response 1:** Yes, when the initial screening shows positive HPV with high-risk HPV types and the cytological examination result is ≥ASC-US, a colposcopy is often performed. A colposcopy is a procedure where a healthcare professional uses a magnifying instrument called a colposcope to examine the cervix more closely. It allows them to identify any areas of concern, such as precancerous lesions, and take appropriate measures accordingly. **Response 2:** When the initial screening shows positive HPV with high-risk HPV types, and the cytological examination result is ≥ASC-US, which stands for high-grade squamous intraepithelial lesion, the next step is typically to perform a colposcopy. The colposcopy allows for a more detailed examination of the cervix, collection of biopsy samples, and further evaluation to ensure accurate diagnosis and appropriate management. **Response 3:** Yes, the next step is to perform a colposcopy. | | 85 | 20 | | | | 10 | Response 1 |
| QiZhenGPT  (with prompt) | **Response 1:** If the cytological examination result is ≥ASC-US, it may indicate a high risk of developing cervical cancer and a colposcopy should be performed to visualize the Cervical Cavity.  **Response 2:** If the cytological examination result is ≥ASC-US, it may indicate a high risk of developing cervical cancer, and a colposcopy is recommended to confirm the diagnosis and identify any potential areas of risk.  **Response 3:** If the cytological examination result is ≥ASC-US, it may indicate that the patient is at high risk for cervical cancer and a colposcopy should be performed to confirm the diagnosis and identify any potential cancer sites. | | 100 | 86 | | | | 93 | Response1 |
| QiZhenGPT  (without prompt) | **Response 1:** if initial screening is HPV-positive and cytology is ASC-US (of uncertain significance), colposcopy (cervical biopsy) is usually needed to further assess the risk of lesions. **Response 2:** colposcopy is usually needed if the initial screen is HPV-positive and is a high-risk type and the cytology is ASC-US or higher.  **Response 3:** If the initial screening result is HPV-positive and high-risk, and the cytology result is ASC-US (of uncertain significance), further testing by colposcopy is usually needed. colposcopy is a visual test that can help your doctor more accurately assess the risk of lesions and take the appropriate therapeutic measures.  5. colposcopy should be performed to determine the extent and degree of the lesion if the initial screening result is HPV-positive and of a high-risk type, and the cytology result is HPV16/18 positive. In some cases, a cervical biopsy may be required to assess the extent of the lesion. | | 90 | 85 | | | | 95 | Response 3 |
| **27.When the initial screening shows positive HPV with high risk HPV types and upon typing it turns out to be HPV16/18 positive, is the next step to perform a colposcopy?** | | | | | | | | | |
| Phi-2  (with prompt) | The semantics of all three responses are identical | | - | - | | | | - | - |
| Phi-2  (without prompt) | The semantics of all three responses are identical | | - | - | | | | - | - |
| QiZhenGPT  (with prompt) | The semantics of all three responses are identical | | - | - | | | | - | - |
| QiZhenGPT  (without prompt) | **Response 1:** colposcopy should be performed if the initial screen is positive for HPV and the cytology result is positive for HPV16/18. If the result is another type of HPV infection, appropriate treatment and follow-up is also required.  **Response 2:** if the initial screening shows HPV positive and a high risk type and the HPV typing test result is HPV 16/18 positive, then colposcopy is usually the next step. **Response 3:** colposcopy should be performed to determine the extent and degree of the lesion if the initial screening result is HPV-positive and of a high-risk type, and the cytology result is HPV16/18 positive. In some cases, a cervical biopsy may be required to assess the extent of the lesion. | | 90 | 85 | | | | 95 | Response 3 |
| **28.When the initial screening shows positive HPV with highrisk HPV types and upon typing it shows neither HPV16/18 positive but one of the other twelve types, what is the most likely subsequent examination?** | | | | | | | | | |
| Phi-2  (with prompt) | **Response 1:** The initial screening shows positive HPV with high-risk HPV types and upon typing it shows neither HPV16/18 positive but one of the other twelve types, what is the most likely subsequent examination?  The most likely subsequent examination is a repeat HPV test.  **Response 2:** The most likely subsequent examination is a colposcopy.  **Response 3:** The initial screening shows positive HPV with high-risk HPV types and upon typing it shows neither HPV16/18 positive but one of the other twelve types, what is the most likely subsequent examination?  The most likely subsequent examination is a repeat HPV test. | | 0 | 100 | | | | 0 | Response3 |
| Phi-2  (without prompt) | **Response 1:** If the initial screening shows positive HPV with high-risk HPV types, and upon typing it reveals none of the HPV16/18 positive strains but one of the other twelve types, the most likely subsequent examination depends on various factors. While the absence of HPV16/18 doesn't eliminate the need for close monitoring, some low-risk HPV types have lower risks than others. Therefore, depending on the individual's specific situation, the healthcare provider may recommend either continued surveillance with regular check-ups or alternative management strategies tailored to the particular HPV type detected. **Response 2:** When the initial screening shows positive HPV with high-risk HPV types, and upon typing it shows neither HPV16/18 positive but one of the other twelve types, the most likely subsequent examination can vary depending on the specific types detected. The colposcopy may be necessary. It allows for a closer examination of the cervix for any abnormalities that could require further evaluation or intervention, such as biopsies or targeted treatment. The examination process for HPV-positive high-risk types involves conducting additional tests, including thorough medical evaluation, biopsies, and consultations with specialists. These procedures are necessary to identify any potential cancer and initiate appropriate treatment if required. **Response 3:** The most likely subsequent examination is a biopsy. | | 55 | 20 | | | | 30 | Response 1 |
| QiZhenGPT  (with prompt) | The semantics of all three responses are identical | | - | - | | | | - | - |
| QiZhenGPT  (without prompt) | **Response 1:** If the initial screen is positive for HPV and the cytology result is either one of the other twelve types (i.e., any of the other types of HPV infection), the most likely follow-up is cytology and histology (e.g., biopsy) to determine the nature of the lesion. **Response 2:** If the initial screen shows HPV-positive and high-risk types and the HPV typing test result is other than HPV16/18-positive, then the most likely follow-up tests are cytology and HPV typing to determine the severity and type of infection. **Response 3:** If the initial screening result shows HPV positivity and is of a high-risk type, and the cytology result is of 12 types other than HPV16/18 positivity, the most likely follow-up test is cytology and histology (e.g., cervical biopsy). This helps to determine the extent and type of lesion and to develop a treatment plan accordingly. | | 80 | 90 | | | | 70 | Response 3 |
| **29.When cytological examination indicates ASC-US as an abnormal initial screening result, what is the next step in the examination?** | | | | | | | | | |
| Phi-2  (with prompt) | The semantics of all three responses are identical | | - | - | | | | - | - |
| Phi-2  (without prompt) | **Response 1:** 1. If cytological examination indicates ASC-US as an abnormal initial screening result, further testing such as colposcopy or biopsy may be recommended depending on the patient's risk factors and clinical presentation. It is important for patients to discuss their results with their healthcare provider to determine the appropriate course of action. **Response 2:** If cytological examination indicates ASC-US as an abnormal initial screening result, subsequent examinations may include colposcopy, HPV DNA testing of the cervical specimen, and further evaluation by a specialist. The choice of examinations depends on various factors, including the individual's risk factors, previous medical history, and any other symptoms or concerns.  **Response 3:** If cytological examination indicates ASC-US as an abnormal initial screening result, the next step in the examination is to perform a colposcopy. | | 85 | 95 | | | | 80 | Response 1 |
| QiZhenGPT  (with prompt) | The semantics of all three responses are identical | | - | - | | | | - | - |
| QiZhenGPT  (without prompt) | **Response 1:** When cytology shows ASC-US as an abnormal initial screening result, what is the next step?  A. When cytology shows ASC-US as an abnormal initial screening result, further testing should be performed to determine the cause of the abnormality. This may include histologic testing, cytologic testing, HPV testing, etc. **Response 2:** What is the next step when cytology shows ASC-US as an abnormal initial screening result?  A. When cytology results show ASC-US as an abnormal initial screening result, further testing should be performed to determine the extent of the lesion. This may include histologic examination, cytologic examination, HPV testing, and others.  **Response 3:** When cytology shows ASC-US as an abnormal initial screening result, what is the next step?  A. When cytology indicates ASC-US as an abnormal initial screening result, further testing should be performed to determine the cause of the abnormality. This may include performing cervical cytology (C cytology), human papillomavirus (HPV) testing, cytology and histology (biopsy). | | 90 | 95 | | | | 85 | Response 3 |
| **30.When cytological examination indicates ASC-US as an abnormal initial screening result and HPV is used for triage, if HPV is positive, should a colposcopy follow?** | | | | | | | | |  |
| Phi-2  (with prompt) | The semantics of all three responses are identical | | - | - | | | | - | - |
| Phi-2  (without prompt) | **Response 1:** If cytological examination shows ASC-US as an abnormal initial screening result and HPV is used for triage, the patient will need a colposcopy to investigate further. **Response 2:** If cytological examination indicates ASC-US as an abnormal initial screening result, and HPV is used for triage, if HPV is positive, yes, a colposcopy should follow. HPV-positive results may suggest a higher chance of cervical abnormalities requiring further investigation and evaluation. **Response 3:** If cytological examination indicates ASC-US as an abnormal initial screening result, and HPV is used for triage, if HPV is positive, a colposcopy should be performed. | | 70 | 90 | | | | 80 | Response 1 |
| QiZhenGPT  (with prompt) | The semantics of all three responses are identical | | - | - | | | | - | - |
| QiZhenGPT  (without prompt) | **Response 1:** When cytology shows ASC-US as an abnormal initial screening result and HPV is used for sorting, is colposcopy required if HPV is positive?  A. When cytology indicates ASC-US as the initial screening result for an abnormality and sorting is performed using HPV, colposcopy is usually required to determine the cause of the abnormality if it is positive for HPV. **Response 2:** When cytology results show ASC-US as an abnormal initial screening result and HPV is used for triage, is colposcopy necessary if HPV is positive?  A. When cytology shows ASC-US as an abnormal initial screening result and HPV is used for triage, a colposcopy is usually needed if HPV is positive. A colposcopy helps the doctor examine the cervical epithelium in more detail in order to detect abnormalities and perform a histologic examination.  **Response 3:** When cytology indicates ASC-US as an abnormal initial screening result and HPV is used for sorting, is colposcopy required if HPV is positive?  A. When cytology shows ASC-US as an abnormal initial screening result and the test is positive for HPV, colposcopy is usually needed. colposcopy is a test used to check for abnormalities of the cervical epithelium, which is histologically examined by using special mirrors and techniques that allow for a clearer view of changes in the cervical epithelium. | | 90 | 85 | | | | 95 | Response 3 |
| **31.When cytological examination indicates ASC-US as an abnormal initial screening result and HPV is used for triage, if HPV is negative, what should be the subsequent examination?** | | | | | | | | | |
| Phi-2  (with prompt) | **Response 1:** When cytological examination indicates ASC-US as an abnormal initial screening result and HPV is used for triage, if HPV is negative, what should be the subsequent examination?  The subsequent examination should be a repeat HPV test.  **Response 2:** If HPV is negative, a colposcopy should not be performed.  **Response 3:** When cytological examination indicates ASC-US as an abnormal initial screening result and HPV is used for triage, if HPV is negative, what should be the subsequent examination?  The subsequent examination should be a repeat HPV test. | | 0 | 100 | | | | 0 | Response3 |
| Phi-2  (without prompt) | **Response 1:** If cytological examination shows ASC-US as an abnormal initial screening result and HPV is not used for triage, the patient will need additional tests or examinations depending on their risk factors and medical history. These may include repeat Pap test, HPV DNA testing, biopsy, etc. **Response 2:** If cytological examination indicates ASC-US as an abnormal initial screening result, and HPV is used for triage, if HPV is negative, subsequent examinations may focus on providing comprehensive education, regular screenings, and following up based on the patient's individual risk factors. This approach aims to ensure comprehensive monitoring and intervention, if necessary. **Response 3:** If cytological examination indicates ASC-US as an abnormal initial screening result, and HPV is used for triage, if HPV is negative, a repeat cytological examination should be performed. | | 40 | 70 | | | | 30 | Response 3 |
| QiZhenGPT  (with prompt) | The semantics of all three responses are identical | | - | - | | | | - | - |
| QiZhenGPT  (without prompt) | **Response 1:** When cytology indicates ASC-US as the initial screening result for an abnormality and sorting is performed using HPV, what is the next test if HPV is negative?  A. When cytology indicates ASC-US as the initial screening result for an abnormality and sorting is performed using HPV, and if HPV is negative, cytology or colposcopy is usually required to determine the cause of the abnormality. **Response 2:** When cytology shows ASC-US as an abnormal initial screening result and HPV is used for triage, what is the next test if HPV is negative?  A. When the cytology results show ASC-US as an abnormal initial screening result and HPV is used for triage, if HPV is negative, other tests may be required, such as the Cervical Cancer Screening Test (CCST) or ThinPrep test. Specific test requirements should be determined on an individual basis and based on the doctor's recommendation.  **Response 3:** When cytology shows ASC-US as an abnormal initial screening result and HPV is sorted, what is the next test if HPV is negative?  A. When cytology indicates ASC-US as the abnormal initial screening result and HPV testing is negative, additional testing may be needed to determine the cause of the abnormality. This may include performing cervical cytology (C cytology) or liquid-based cytology (Vaginal cytology), and performing cytology and histology (biopsy), among others. | | 70 | 80 | | | | 65 | Response 1 |
| **32.When cytological examination results show ASCH, LSIL, or HSIL, is a colposcopy needed next?** | | | | | | | | | |
| Phi-2  (with prompt) | The semantics of all three responses are identical | | - | - | | | | - | - |
| Phi-2  (without prompt) | The semantics of all three responses are identical | | - | - | | | | - | - |
| QiZhenGPT  (with prompt) | **Response 1:** If ASC-H, LSIL, or HSIL is detected, a colposcopy should be performed.  **Response 2:** If ASC-H, LSIL, or HSIL is detected, a colposcopy should be performed.  **Response 3:** If the cytological examination results show ASC-H, LSIL, or HSIL, a colposcopy would be needed next to determine the underlying cause of the abnormal result. | | 100 | 86 | | | | 91 | Response1 |
| QiZhenGPT  (without prompt) | **Response 1:** When cytology results indicate ASC-H, LSIL, HSIL, is colposcopy necessary?  A: When cytology results indicate ASC-H, LSIL, HSIL, colposcopy is usually required to determine the cause of the abnormality. **Response 2:** Is colposcopy required when the cytology results show ASC-H, LSIL, HSIL?  A: Colposcopy is usually required when the cytology result shows ASC-H, LSIL, HSIL. Colposcopy can help the doctor to examine the cervical epithelium in more detail so that abnormalities can be detected and histologic examination can be performed. However, specific examination requirements should be determined on an individual basis and based on the doctor's recommendation. **Response 3:** Is colposcopy necessary when the cytology results indicate ASC-H, LSIL, HSIL?  A. Colposcopy is usually needed when the cytology results indicate ASC-H, LSIL or HSIL. Colposcopy helps the doctor to see the changes in the cervical epithelium more clearly and to examine it histologically to determine the cause of the abnormality and to take appropriate treatment. | | 85 | 90 | | | | 95 | Response 3 |
| **33.When cytological examination results show AGC, what is the next examination required?** | | | | | | | | | |
| Phi-2  (with prompt) | The semantics of all three responses are identical | | - | - | | | | - | - |
| Phi-2  (without prompt) | **Response 1:** If cytological examination results show AGC (atypical glandular cells), the patient will likely require a colposcopy due to the increased risk of developing cervical cancer. **Response 2:** If cytological examination results show AGC (atypical glandular cell), there are several possible next examinations. They include colposcopy, HPV DNA testing of the cervical specimen, biopsy, or other appropriate investigations depending on the specific results and clinical findings. **Response 3:** If cytological examination results show AGC, a repeat cytological examination should be performed. | | 80 | 20 | | | | 10 | Response 1 |
| QiZhenGPT  (with prompt) | **Response 1:** When cytological examination results show AGC, what is the next examination required?  AGC means that there is a high risk of developing cervical cancer. The next examination required would be a colposcopy to visualize the internal cervical os and identify any potential cancer cells.  **Response 2:** When cytological examination results show AGC, what is the next examination required?  AGC stands for "Advanced Goal Scores," which are a measure of the risk of developing cervical cancer based on the results of a cytological examination. The next examination required would be a colposcopy to visualize the Cervical Cancer Nodes (CNs) and to determine the stage of the disease.  **Response 3:** When cytological examination results show AGC, what is the next examination required?  AGC means that there is a high risk of developing cervical cancer. The next examination required would be a colposcopy to visualize the internal cervical os and identify any potential cancer cells. | | 20 | 100 | | | | 30 | Response3 |
| QiZhenGPT  (without prompt) | **Response 1:** What is the next step when cytology shows AGC?  A. When cytology results indicate AGC, cytology, histology, and HPV testing are usually required to determine the cause of the abnormality. **Response 2:** When the cytology results show AGC, what is the next test?  A. When cytology results show AGC, further testing is usually required to determine the extent of the lesion. This may include histology, cytology, HPV testing, and others. Specific testing requirements should be determined on an individual basis and as recommended by your physician.  **Response 3:** When the cytologic findings indicate AGC, what is the next test?  A. When cytologic findings indicate AGC, further testing is usually necessary to determine the cause of the abnormality. This may include performing cervical cytology (C cytology), human papillomavirus (HPV) testing, cytology and histology (biopsy). Depending on the cause of the abnormality, your doctor may recommend different tests and treatments. | | 80 | 60 | | | | 70 | Response 1 |
| **34.If both cytological and highrisk HPV joint tests show negative results, what other examination should follow?** | | | | | | | | | |
| Phi-2  (with prompt) | The semantics of all three responses are identical | | - | - | | | | - | - |
| Phi-2  (without prompt) | **Response 1:** If both cytological and high-risk HPV joint tests show negative results, additional screening may not be necessary at that time, but patients should continue regular screenings according to their age and risk factors. **Response 2:** The next examination should be a colposcopy. **Response 3:** If both cytological and high-risk HPV joint tests show negative results, a colposcopy should follow. | | 0 | 100 | | | | 0 | Response 1 |
| QiZhenGPT  (with prompt) | The semantics of all three responses are identical | | - | - | | | | - | - |
| QiZhenGPT  (without prompt) | The semantics of all three responses are identical | | - | - | | | | - | - |
| **35.If joint testing of cytology and highrisk HPV shows HPVnegative and the cytological result is ASC-US, what should be done next?** | | | | | | | | | |
| Phi-2  (with prompt) | The semantics of all three responses are identical | | - | - | | | | - | - |
| Phi-2  (without prompt) | The semantics of all three responses are identical | | - | - | | | | - | - |
| QiZhenGPT  (with prompt) | **Response 1:** If joint testing of cytology and high-risk HPV shows HPV-negative and the cytological result is ASC-US, what should be done next?  If joint testing of cytology and high-risk HPV shows HPV-negative and the cytological result is ASC-US, it means that there is no evidence of cervical cancer or high-risk HPV infection. The next examination would be a colposcopy to visualize the internal cervical os and identify any potential cancer cells.  **Response 2:** If joint testing of cytology and high-risk HPV shows HPV-negative and the cytological result is ASC-US, what should be done next?  If joint testing of cytology and high-risk HPV shows HPV-negative and the cytological result is ASC-US, it is recommended that a follow-up test be performed to ensure that the results are accurate. This would be a colposcopy to visualize the CNs and to determine the stage of the disease.  **Response 3:** If joint testing of cytology and high-risk HPV shows HPV-negative and the cytological result is ASC-US, what should be done next?  If joint testing of cytology and high-risk HPV shows HPV-negative and the cytological result is ASC-US, it means that there is no evidence of cervical cancer or high-risk HPV infection. The next examination would be a colposcopy to visualize the internal cervical os and identify any potential cancer cells. | | 95 | 100 | | | | 88 | Response1 |
| QiZhenGPT  (without prompt) | **Response 1:** If the combined cytology and high-risk HPV test result shows HPV-negative and the cytology result is ASC-US, what should be done next?  A. If the combined cytology and high-risk HPV test result shows HPV-negative and the cytology result is ASC-US, colposcopy and HPV testing should be performed. **Response 2:** If the combined cytology and high-risk HPV test result shows HPV-negative and the cytology result is ASC-US, what should be done next?  A. If the combined cytology and high-risk HPV test result shows HPV-negative and the cytology result is ASC-US, a colposcopy should be performed. In colposcopy, the doctor can use special equipment to get a clearer view of the cervical epithelium and take samples for pathologic examination. If the pathology is positive, further treatment may be needed.  **Response 3:** If the combined cytology and high-risk HPV test result is HPV-negative and the cytological result is ASC-US, what should be done next?  A. If the combined cytology and high-risk HPV test result is HPV-negative and the cytological result is ASC-US, colposcopy should be performed. During colposcopy, the area of the lesion should be smeared with magenta acetate solution and the smear should be observed for the presence of abnormal cells. If abnormal cells are present in the smear, pathologic examination should be performed. | | 100 | 91 | | | | 85 | Response 1 |
| **36.If joint testing of cytology and highrisk HPV shows HPVnegative and the cytological result is >ASC-US, what should be done next? Is a colposcopy required?** | | | | | | | | | |
| Phi-2  (with prompt) | The semantics of all three responses are identical | | - | - | | | | - | - |
| Phi-2  (without prompt) | **Response 1:** If joint testing of cytology and high-risk HPV shows HPV-negative, and the cytological result is >ASC-US, colposcopy is still recommended to evaluate the abnormal cells and determine whether they are precancerous or benign. **Response 2:** If joint testing of cytology and high-risk HPV shows HPV-negative, and the cytological result is >ASC-US, no further examination will be necessary as negative HPV results are normal according to the ACOG guidelines. **Response 3:** If joint testing of cytology and high-risk HPV shows HPV-negative, and the cytological result is >ASC-US, a colposcopy is not required. | | 50 | 0 | | | | 0 | Response 1 |
| QiZhenGPT  (with prompt) | The semantics of all three responses are identical | | - | - | | | | - | - |
| QiZhenGPT  (without prompt) | **Response 1:** If the combined cytology and high-risk HPV test result shows HPV-negative and the cytology result is >ASC-US, what should be done next? Is colposcopy necessary?  A: If the combined cytology and high-risk HPV test result shows HPV-negative and the cytology result is >ASC-US, colposcopy should be performed. The need for further testing depends on the circumstances and physician judgment. **Response 2:** If the combined cytology and high-risk HPV test result shows HPV-negative and the cytology result is >ASC-US, what should be done next? Is colposcopy necessary?  A: If the combined cytology and high-risk HPV test result shows HPV-negative and the cytology result is >ASC-US, a colposcopy should be performed. In colposcopy, the doctor can use special equipment to visualize the cervical epithelium more clearly and take samples for pathological examination. If the pathology is positive, further treatment may be needed. The need for colposcopy depends on the individual case and should be decided on the advice of your doctor. **Response 3:** If the combined cytologic and high-risk HPV test result is HPV-negative and the cytologic result is >ASC-US, what should be done next?  A. If the combined cytology and high-risk HPV test result is HPV-negative and the cytological result is >ASC-US, one year of follow-up, including cytology and high-risk HPV testing, should be performed. Cytology and high-risk HPV testing should be performed regularly during the follow-up period. If abnormalities are detected during follow-up, further investigations and treatment should be performed. | | 90 | 55 | | | | 70 | Response 1 |
| **37.In cervical cancer detection, if joint testing of cytology and highrisk HPV shows HPVpositive and the cytological result is ≥ASC-US, what specific examinations should follow?** | | | | | | | | | |
| Phi-2  (with prompt) | The semantics of all three responses are identical | | - | - | | | | - | - |
| Phi-2  (without prompt) | **Response 1:** In cervical cancer detection, if joint testing of cytology and high-risk HPV shows HPV-positive, and the cytological result is ≥ASC-US, colposcopy followed by biopsy of the suspicious areas is typically performed to confirm the diagnosis. **Response 2:** If joint testing of cytology and high-risk HPV shows HPV-positive, and the cytological result is ≥ASC-US, the patient should undergo a cone biopsy or, if they have a normal PAP smear in recent history, a colposcopy.  **Response 3:** In cervical cancer detection, if joint testing of cytology and high-risk HPV shows HPV-positive, and the cytological result is ≥ASC-US, a colposcopy should be done, and a biopsy should be taken. | | 80 | 70 | | | | 90 | Response 1 |
| QiZhenGPT  (with prompt) | The semantics of all three responses are identical | | - | - | | | | - | - |
| QiZhenGPT  (without prompt) | **Response 1:** In cervical cancer screening, if the combined cytology and high-risk HPV test result shows HPV-positive and the cytology result is ≥ASC-US, what specific tests should be performed next?  A: If the combined cytology and high-risk HPV test result shows HPV-positive and the cytology result is ≥ASC-US, liquid-based cytology (LBC) and HPV testing should be performed. Human papillomavirus (HPV) typing may also be indicated if needed. **Response 2:** In cervical cancer screening, if the combined cytology and high-risk HPV test result shows HPV-positive and the cytology result is ≥ASC-US, what tests should be performed next?  A: If, in cervical cancer screening, the combined cytology and high-risk HPV test result shows HPV-positive and the cytology result is ≥ASC-US, the following tests should be performed: first, a v test should be performed to determine if one is infected with human papillomavirus (HPV). Second, cervical cytology and HPV testing should be performed to determine the presence of cervical intraepithelial neoplasia (CIN). If the CIN level is ≥CIN 2 or CIN 3, a cervical biopsy should be performed to determine if there is a risk of cervical cancer.  **Response 3:** In cervical cancer screening, if the combined cytology and high-risk HPV test result shows HPV-positive, with a cytological result of ≥ASC-US, what tests should be performed next?  A: If the combined cytology and high-risk HPV test result shows HPV-positive with a cytological result of ≥ASC-US during cervical cancer screening, colposcopy and pathology should be performed. Colposcopy can help physicians better visualize cervical lesions, while pathology can determine the nature and extent of the lesions. If the pathology result is malignant, further treatment should be carried out. | | 85 | 65 | | | | 60 | Response 2 |
| **38.In cervical cancer detection, if joint testing of cytology and highrisk HPV shows HPV-positive and the cytological result is negative, what specific examinations should follow?** | | | | | | | | | |
| Phi-2  (with prompt) | The semantics of all three responses are identical | | - | - | | | | - | - |
| Phi-2  (without prompt) | **Response 1:** In cervical cancer detection, if joint testing of cytology and high-risk HPV shows HPV-positive and the cytological result is negative, repeat testing within 3 months may be considered depending on the individual case and risk factors. **Response 2:** If joint testing of cytology and high-risk HPV shows HPV-positive and the cytological result is negative, no further investigation will be necessary as negative cytopathological reports are abnormal according to the ACOG guidelines.  **Response 3:** In cervical cancer detection, if joint testing of cytology and high-risk HPV shows HPV-positive and the cytological result is negative, a colposcopy should be done, and a biopsy should be taken. | | 60 | 20 | | | | 30 | Response 1 |
| QiZhenGPT  (with prompt) | The semantics of all three responses are identical | | - | - | | | | - | - |
| QiZhenGPT  (without prompt) | **Response 1:** During cervical cancer screening, if the combined cytology and high-risk HPV test result is HPV-positive and the cytology result is negative, what specific tests should be performed next?  A. In cervical cancer screening, if the combined cytology and high-risk HPV test result shows HPV-positive and the cytology result is negative, a repeat cytology test or a turn-around negative test should be performed. **Response 2:** In cervical cancer screening, if the combined cytology and high-risk HPV test result shows HPV-positive and the cytology result is negative, what tests should be performed?  A. If, in cervical cancer screening, the combined cytology and high-risk HPV test result shows HPV-positive and the cytology result is negative, the following tests should be performed: First, a TZB test should be performed to determine if you are infected with human papillomavirus (HPV). Then, cervical cytology and HPV testing should be performed to determine the presence of cervical intraepithelial neoplasia (CIN). If the CIN level is ≥CIN 2 or CIN 3, a cervical biopsy should be performed to determine if there is a risk of cervical cancer. **Response 3:** In cervical cancer screening, if the combined cytology and high-risk HPV test result shows HPV-positive and the cytological result is negative, what tests should be performed?  A. If the combined cytology and high-risk HPV test result shows HPV-positive and the cytological result is negative during cervical cancer screening, a one-year follow-up, including cytology and high-risk HPV testing, should be performed. Cytology and high-risk HPV testing should be performed regularly during the follow-up period. If abnormalities are detected during follow-up, further investigations and treatment should be performed. | | 55 | 70 | | | | 58 | Response 3 |
| **39.In cervical cancer detection, if joint testing of cytology and highrisk HPV shows HPVpositive and the cytological result is negative, and the HPV typing is HPV16/18 positive, what specific examinations should follow?** | | | | | | | | | |
| Phi-2  (with prompt) | The semantics of all three responses are identical | | - | - | | | | - | - |
| Phi-2  (without prompt) | **Response 1:** In cervical cancer detection, if joint testing of cytology and high-risk HPV shows HPV-positive and the cytological result is negative, HPV typing for HPV16 or HPV18 may be useful to identify specific subtypes associated with higher risk of developing invasive disease. **Response 2:** If a patient has a joint testing of cytology and high-risk HPV showing HPV-positive, the cytological result is negative, and there's no typing for HPV16 or HPV18, then a colposcopy is needed. **Response 3:** In cervical cancer detection, if joint testing of cytology and high-risk HPV shows HPV-positive and the cytological result is negative, and the HPV typing is HPV16/18 positive, a colposcopy should be done, and a biopsy should be taken. | | 50 | 80 | | | | 30 | Response 1 |
| QiZhenGPT  (with prompt) | The semantics of all three responses are identical | | - | - | | | | - | - |
| QiZhenGPT  (without prompt) | **Response 1:** In cervical cancer screening, if the combined cytology and high-risk HPV test result shows HPV-positive and the cytology result is negative, and the HPV typing is HPV16/18 positive, what specific test should be performed next?  A: In cervical cancer screening, if the combined cytology and high-risk HPV test result shows HPV-positive and negative cytology and HPV typing is HPV16/18 positive, HPV16/18 typing and cytology should be performed. Depending on the circumstances, other relevant tests may be required. **Response 2:** At cervical cancer screening, if the combined cytology and high-risk HPV test result shows HPV-positive, the cytology result is negative, and the HPV typing is HPV16/18 positive, what tests should be performed next?  A: If at cervical cancer screening, the combined cytology and high-risk HPV test result shows HPV-positive, the cytology result is negative, and the HPV typing is HPV16/18 positive, the following tests should be performed: First, a TZB test should be performed to determine whether or not one is infected with human papillomavirus (HPV). Then, cervical cytology and HPV testing should be performed to determine the presence of cervical intraepithelial neoplasia (CIN). If the CIN level is ≥CIN 2 or CIN 3, a cervical biopsy should be performed to determine if there is a risk of cervical cancer. In addition, HPV 16/18 genotype testing should be performed to assess the impact of HPV infection on cervical cancer risk. **Response 3:** During cervical cancer screening, if the combined cytology and high-risk HPV test result shows HPV-positive, the cytological result is negative, and the HPV typing is HPV16/18 positive, what test should be performed next?  A: If at cervical cancer screening, the combined cytology and high-risk HPV test result shows HPV-positive, the cytological result is negative, and the HPV typing is HPV16/18 positive, further testing and treatment should be performed. Specific tests include cervical biopsy and HPV 16/18 typing. Cervical biopsy can help the doctor better observe the cervical lesions, while HPV16/18 typing can help the doctor determine the specific type of HPV infection so that a more personalized treatment plan can be developed. If the pathology results are malignant, further treatment should be carried out. | | 75 | 95 | | | | 80 | Response 3 |
| **40.In cervical cancer detection, if a patient has joint testing of cytology and highrisk HPV showing HPVpositive, the cytological result is negative, and there's no typing for HPV16 or HPV18, are there any other tests needed?** | | | | | | | | | |
| Phi-2  (with prompt) | The semantics of all three responses are identical | | - | - | | | | - | - |
| Phi-2  (without prompt) | **Response 1:** n cervical cancer detection, if a patient has joint testing of cytology and high-risk HPV showing HPV-positive, the cytological result is negative, and there's no typing for HPV16 or HPV18, other diagnostic tests such as liquid-based cytology or Pap smear may be used to obtain further information about the abnormal cells. However, these tests have lower sensitivity than the combined test and may miss some cases of cervical abnormalities. Regular monitoring with follow-up exams is important to detect any changes in the cervix over time.  **Response 2:** No further tests are necessary if joint testing of cytology and high-risk HPV shows HPV-positive, the cytological result is negative, and there's no typing for HPV16 or HPV18. **Response 3:** If a patient has joint testing of cytology and high-risk HPV showing HPV-positive, the cytological result is negative, and there's no typing for HPV16 or HPV18, a colposcopy should be done, and a biopsy should be taken. | | 90 | 20 | | | | 10 | Response 1 |
| QiZhenGPT  (with prompt) | The semantics of all three responses are identical | | - | - | | | | - | - |
| QiZhenGPT  (without prompt) | **Response 1:** In cervical cancer screening, if a patient undergoes combined cytology and high-risk HPV testing that shows HPV-positive with negative cytology results and no HPV 16 or 18 typing detected, are additional tests necessary?  A: In this case, other relevant tests such as immunologic testing, cytology repeat testing, or trans-negative testing may be required. The specific testing needs depend on the patient's condition and the doctor's judgment.  **Response 2:** In cervical cancer screening, if a patient has a combination of a positive test result for a high-risk HPV type, a negative cytology result, and no HPV types 16 or 18 detected, is additional testing necessary?  A: If, at cervical cancer screening, a patient has a positive co-test for high-risk HPV, a negative cytology result, and no HPV types 16 or 18 are detected, then additional testing may be necessary. Specifically, a TZB test (cytology) should be performed to determine if infection with human papillomavirus (HPV) is present. Cervical cytology and HPV testing should then be performed to determine the presence of cervical intraepithelial neoplasia (CIN). If the CIN grade is ≥CIN 2 or CIN 3, a cervical biopsy should be performed to determine if there is a risk of cervical cancer. **Response 3:** In cervical cancer screening, if a patient has a combined test result showing HPV-positive, a negative cytological result, and no HPV16 or HPV18 type test result, what other tests are needed?  A: If a patient has a combined test result of HPV-positive, a negative cytological result, and no HPV16 or HPV18 type results at cervical cancer screening, additional testing, such as immunologic testing or genetic testing, may be needed. Immunologic testing can help the doctor determine if the patient is infected with other types of HPV. Genetic testing can help the doctor determine if there is an HPV16 or HPV18 genotype infection, which can lead to a more personalized treatment plan. If further testing is needed, it should be developed on a case-by-case basis. | | 90 | 78 | | | | 82 | Response 1 |
| **41.In cervical cancer screening, if a patient has a histopathological confirmation (biopsy) of LSIL cytology findings of LSIL or higher and a TZ3 classification, what followup steps should be conducted?** | | | | | | | | | |
| Phi-2  (with prompt) | The semantics of all three responses are identical | | - | - | | | | - | - |
| Phi-2  (without prompt) | **Response 1:** If a patient has a histopathological (biopsy) confirmation of low-grade squamous intraepithelial lesion (LSIL), cytopathology findings of LSIL or higher, and a tri-color zone 3 classification, it's recommended that they undergo repeat Pap smear testing every 6 months for 2 years after their initial diagnosis. After that, they can continue regular screenings based on local guidelines. **Response 2:** After a histopathological diagnosis of LSIL, no further follow-up steps are needed. You may commence treatment with antiviral medicines if you are HPV-positive. **Response 3:** If a patient has a histopathological confirmation(biopsy) of LSIL, cytology findings of LSIL or higher, and a TZ3 classification, the patient should be referred for colposcopy. | | 0 | 0 | | | | 0 | Response 1 |
| QiZhenGPT  (with prompt) | The semantics of all three responses are identical | | - | - | | | | - | - |
| QiZhenGPT  (without prompt) | **Response 1:** In cervical screening, if a patient has histopathologic confirmation of LSIL (biopsy), cervical cytology finding of LSIL or higher, and TZ3 classification, what follow-up steps should be performed?  A: If a patient has histopathologic confirmation of LSIL on cervical screening, cervical cytology finding of LSIL or higher, and TZ3 classification, the following follow-up steps should be performed:  * Cervical cytology and/or colposcopy every 6-12 months  * Cervical cytology and/or colposcopy every 1-2 years  * Consider cervical biopsy to evaluate for cancer if cervical cytology results are consistently LSIL or higher **Response 2:** In cervical cancer screening, what follow-up steps should be performed if a patient has pathologic confirmation of LSIL (biopsy), cervical cytology finding of LSIL or higher, and TZ3 classification?  A. If a patient has pathologic confirmation of LSIL (biopsy), cervical cytology finding of LSIL or higher, and TZ3 classification at cervical cancer screening, the following follow-up steps should be performed: close follow-up, including regular cervical cytology and HPV testing, cervical biopsy every 6-12 months, and regular physical examination. **Response 3:** 1. In cervical screening, what follow-up steps should be performed if a patient has pathologic confirmation of LSIL (biopsy), cervical cytology finding of LSIL or higher, and TZ3 classification?  A: If a patient has pathologic confirmation of LSIL, cervical cytology finding of LSIL or higher, and TZ3 classification at cervical screening, the following follow-up steps should be performed:  a) Cervical cytology (Pap smear) and/or cytology and histology (colposcopy) every 6-12 months.  b) If cervical cytology results are persistently LSIL or higher, a LEEP biopsy should be considered to evaluate for the presence of cervical intraepithelial neoplasia (CIN).  c) For persistent LSIL, regular follow-up, including cervical cytology and/or colposcopy, should be performed to monitor lesion progression. | | 90 | 78 | | | | 82 | Response 1 |
| **42. In cervical cancer screening, if a patient has a histopathological confirmation (biopsy) of LSIL, cytology findings of LSIL or higher, and a TZ1/2 classification, what follow-up steps should be conducted?** | | | | | | | | | |
| Phi-2  (with prompt) | The semantics of all three responses are identical | | - | - | | | | - | - |
| Phi-2  (without prompt) | **Response 1:** If a patient has a histopathological confirmation of LSIL, cytology findings of LSIL or lower, and a tri-color zone 1/2 classification, it's also recommended that they undergo repeat Pap smear testing every 6 months for 2 years after their initial diagnosis. Following that, they can continue regular screenings based on local guidelines. **Response 2:** After a histopathological confirmation of LSIL, even if cytology findings are LSIL or ASC-H grade, if a TZ1/2 semipersistent HSIL or worse is observed, colposcopy should be carried out as the cervical lesion has not reverted to LSIL.  **Response 3:** If a patient has a histopathological confirmation(biopsy) of LSIL, cytology findings of LSIL or lower, and a TZ1/2 classification, the patient should be referred for colposcopy. | | 65 | 75 | | | | 60 | Response 1 |
| QiZhenGPT  (with prompt) | The semantics of all three responses are identical | | - | - | | | | - | - |
| QiZhenGPT  (without prompt) | **Response 1:** In cervical screening, what follow-up steps should be performed if a patient has histopathologic confirmation of LSIL (biopsy), a cervical cytology finding of LSIL or lower, and a TZ1/2 classification?  A: If a patient has histopathologic confirmation of LSIL on cervical screening, cervical cytology finding of LSIL or lower, and TZ1/2 classification, the following follow-up steps should be performed:  * Cervical cytology and/or colposcopy every 6-12 months  * Cervical cytology and/or colposcopy every 1-2 years  * Consider cervical biopsy to evaluate for cancer if cervical cytology results are consistently LSIL or lower **Response 2:** In cervical cancer screening, what follow-up steps should be performed if a patient has pathologic confirmation of LSIL (biopsy), cervical cytology that reveals LSIL or lower, and TZ1/2 classification?  A. If a patient has pathologic confirmation of LSIL (biopsy), LSIL or lower on cervical cytology, and TZ1/2 classification at cervical cancer screening, the following follow-up steps should be performed: first, cervical cytology and HPV testing, cervical biopsy every 6-12 months, and regular physical exams. **Response 3:** In cervical screening, what follow-up steps should be performed if a patient has pathologic confirmation of LSIL, cervical cytology findings of LSIL or lower, and TZ1/2 classification?  A: If a patient has pathologic confirmation of LSIL, cervical cytology findings of LSIL or lower, and TZ1/2 classification on cervical screening, the following follow-up steps should be performed:  a) Cervical cytology (Pap smear) and/or cytology and histology (colposcopy) every 6-12 months.  b) If cervical cytology results are persistently LSIL or lower, a LEEP biopsy should be considered to evaluate for the presence of cervical intraepithelial neoplasia (CIN).  c) For persistent LSIL, regular follow-up, including cervical cytology and/or colposcopy, should be performed to monitor for lesion progression. | | 70 | 85 | | | | 65 | Response 3 |
| **43.In cervical cancer screening, if a patient has a histopathological confirmation (biopsy) of LSIL cytology findings of ASCH or higher, what followup steps should be conducted?** | | | | | | | | | |
| Phi-2  (with prompt) | **Response 1:** In cervical cancer screening, if a patient has a histopathological confirmation (biopsy) of LSIL cytology findings of ASC-H or higher, the patient should be referred to a gynecologic oncologist for further evaluation and management. The patient should undergo a colposcopy to assess the extent of the abnormal cells and determine the need for additional treatment, such as cryotherapy, loop electrosurgical excision procedure (LEEP), or conization. The patient should also be counseled on the risk of developing cervical cancer and the importance of regular follow-up screenings.  **Response 2:** If a patient has a histopathological confirmation (biopsy) of LSIL cytology findings of ASC-H or higher, the next step is to perform a colposcopy. If the colposcopy reveals no abnormalities, the patient can be rescreened in 3 years. If the colposcopy reveals abnormalities, the patient should undergo a cone biopsy.  **Response 3:** In cervical cancer screening, if a patient has a histopathological confirmation (biopsy) of LSIL cytology findings of ASC-H or higher, the patient should be referred to a gynecologic oncologist for further evaluation and management. The patient should undergo a repeat colposcopy with biopsy, and if the biopsy results are consistent with high-grade squamous intraepithelial lesion (HSIL) or cervical cancer, the patient should be referred to a gynecologic oncologist for further evaluation and management. | | 74 | 100 | | | | 69 | Response3 |
| Phi-2  (without prompt) | The semantics of all three responses are identical | | - | - | | | | - | - |
| QiZhenGPT  (with prompt) | **Response 1:** In cervical cancer screening, if a patient has a histopathological confirmation (biopsy) of LSIL cytology findings of ASC-H or higher, the next step would be to perform a colposcopy to evaluate the results.  **Response 2:** Follow-up steps should be conducted to evaluate the persistence or progression of the LSIL (low-gradeSIL)cythemia in the patient. This evaluation may include a repeatoscopy and/or a biopsy.  **Response 3:** Follow-up steps should be conducted to evaluate the persistence or progression of the LSIL (low-gradeSIL)cythemia in the patient. This may involve re-screening the patient in 6-12 months, or more frequently if the LSILcythemia persists or worsens. | | 78 | 66 | | | | 86 | Response3 |
| QiZhenGPT  (without prompt) | **Response 1:** at cervical screening, if a patient has histopathologic confirmation of LSIL (biopsy), a cervical cytology finding of ASC-H or higher, and a TZ3 classification, what follow-up steps should be performed?  A: If a patient has histopathologic confirmation of LSIL, cervical cytology finding of ASC-H or higher, and TZ3 classification on cervical screening, the following follow-up steps should be performed:  * Cervical cytology and/or colposcopy every 6-12 months  * Cervical cytology and/or colposcopy every 1-2 years  * Consider cervical biopsy to evaluate for cancer if cervical cytology results are consistently ASC-H or higher **Response 2:** In cervical cancer screening, if a patient has pathologic confirmation of LSIL (biopsy), and cervical cytology reveals ASC-H or higher, what follow-up steps should be performed?  A. If a patient has pathologic confirmation of LSIL (biopsy) and cervical cytology reveals ASC-H or higher at cervical cancer screening, the following follow-up steps should be performed: cervical cytology and HPV testing in the first instance, cervical biopsy every 6-12 months, and regular physical exams.  **Response 3:** In cervical screening, what follow-up steps should be performed if a patient has pathologic confirmation of LSIL, a cervical cytology finding of ASC-H or higher, and a TZ3 classification?  A: If a patient has pathologic confirmation of LSIL, cervical cytology findings of ASC-H or higher, and TZ3 classification on cervical screening, the following follow-up steps should be performed:  a) Cervical cytology (Pap smear) and/or cytology and histology (colposcopy) every 6-12 months.  b) If cervical cytology results are persistently ASC-H or higher, a LEEP biopsy should be considered to evaluate for the presence of cervical intraepithelial neoplasia (CIN).  c) For persistent LSIL, regular follow-up, including cervical cytology and/or colposcopy, should be performed to monitor the progression of the lesion. | | 77 | 89 | | | | 71 | Response 3 |
| **44.In cervical cancer screening, if a patient aged 21 to 24 years has a histopathological confirmation (biopsy) of LSIL cytology findings of ASCH or higher, what followup steps should be conducted?** | | | | | | | | | |
| Phi-2  (with prompt) | **Response 1:** In cervical cancer screening, if a patient aged 21 to 24 years has a histopathological confirmation (biopsy) of LSIL cytology findings of ASC-H or higher, the patient should be referred to a gynecologic oncologist for further evaluation and management. The patient should undergo a colposcopy to assess the extent of the abnormal cells and determine the need for additional treatment, such as cryotherapy, LEEP, or conization. The patient should also be counseled on the risk of developing cervical cancer and the importance of regular follow-up screenings.  **Response 2:** If a patient aged 21 to 24 years has a histopathological confirmation (biopsy) of LSIL cytology findings of ASC-H or higher, the next step is to perform a colposcopy. If the colposcopy reveals no abnormalities, the patient can be rescreened in 3 years. If the colposcopy reveals abnormalities, the patient should undergo a cone biopsy.  **Response 3:** In cervical cancer screening, if a patient aged 21 to 24 years has a histopathological confirmation (biopsy) of LSIL cytology findings of ASC-H or higher, the patient should be referred to a gynecologic oncologist for further evaluation and management. The patient should undergo a repeat colposcopy with biopsy, and if the biopsy results are consistent with high-grade squamous intraepithelial lesion (HSIL) or cervical cancer, the patient should be referred to a gynecologic oncologist for further evaluation and management. | | 75 | 85 | | | | 60 | Response3 |
| Phi-2  (without prompt) | **Response 1:** For patients aged 21 to 24 who have a histopathological confirmation of LSIL, cytology findings of ASC-H or higher, it's important to conduct a thorough evaluation. They may require additional tests such as HPV DNA testing or colposcopy to determine the appropriate course of action. The specific recommendations will depend on individual circumstances and the guidance provided by healthcare professionals.  **Response 2:** As long as the histopathology (biopsy) result of LSIL or ASCC-H is confirmed, and if cervical cytological findings are ASC-H or higher, they should be retested after 6 months; otherwise, colposcopy is not recommended.  **Response 3:** If a patient aged 21 to 24 years has a histopathological confirmation(biopsy) of LSIL, cytology findings of ASC-H or higher, and a TZ3 classification for colposcopy, the patient should be referred for colposcopy. | | 50 | 0 | | | | 0 | Response 1 |
| QiZhenGPT  (with prompt) | **Response 1:** In cervical cancer screening, if a patient aged 21 to 24 years has a histopathological confirmation (biopsy) of LSIL cytology findings of ASC-H or higher, the next step would be to perform a colposcopy to evaluate the results.  **Response 2:** Follow-up steps should be conducted to evaluate the patient for any potential complications or risks associated with the procedure. The patient should also be referred to a specialist for further evaluation and management.  **Response 3:** Follow-up steps should be conducted to evaluate the patient for any potential complications or risks associated with the LSILcythemia. This may include performing a colposcopy or other imaging studies to assess the site of thecythemia and to identify any potential complications such as bleeding or infection. | | 67 | 55 | | | | 73 | Response2 |
| QiZhenGPT  (without prompt) | **Response 1:** Blank **Response 2:** In cervical cancer screening, if a patient aged 21 to 24 years has pathologic confirmation of LSIL (biopsy) and cervical cytology reveals ASC-H or higher, what follow-up steps should be performed?  A. If a patient 21 to 24 years of age has pathologic confirmation of LSIL (biopsy) and cervical cytology reveals ASC-H or higher at cervical cancer screening, the following follow-up steps should be performed: cervical cytology and HPV testing first, cervical biopsy every 6 to 12 months, and periodic physical exams. **Response 3:** In cervical screening, what follow-up steps should be performed if a patient 21 to 24 years of age has pathologic confirmation of LSIL, a cervical cytology finding of ASC-H or higher, and a TZ3 classification?  A: If a patient 21 to 24 years of age has pathologic confirmation of LSIL, cervical cytology findings of ASC-H or higher, and TZ3 classification on cervical screening, the following follow-up steps should be performed:  a) Cervical cytology (Pap smear) and/or cytology and histology (colposcopy) every 6-12 months.  b) If cervical cytology results are persistently ASC-H or higher, a LEEP biopsy should be considered to evaluate for the presence of cervical intraepithelial neoplasia (CIN).  c) For persistent LSIL, regular follow-up, including cervical cytology and/or colposcopy, should be performed to monitor lesion progression. | | 70 | 55 | | | | 60 | Response 2 |
| **45.In cervical cancer screening, if a patient aged 21 to 24 years has a histopathological confirmation (biopsy) of LSIL cytology findings of ASCH or higher and a TZ3 classification for colposcopy, what followup steps should be conducted?** | | | | | | | | | |
| Phi-2  (with prompt) | **Response 1:** In cervical cancer screening, if a patient aged 21 to 24 years has a histopathological confirmation (biopsy) of LSIL cytology findings of ASC-H or higher and a TZ3 classification for colposcopy, the patient should be referred to a gynecologic oncologist for further evaluation and management. The patient should undergo a colposcopy to assess the extent of the abnormal cells and determine the need for additional treatment, such as cryotherapy, LEEP, or conization. The patient should also be counseled on the risk of developing cervical cancer and the importance of regular follow-up screenings.  **Response 2:** If a patient aged 21 to 24 years has a histopathological confirmation (biopsy) of LSIL cytology findings of ASC-H or higher and a TZ3 classification for colposcopy, the next step is to perform a colposcopy. If the colposcopy reveals no abnormalities, the patient can be rescreened in 3 years. If the colposcopy reveals abnormalities, the patient should undergo a cone biopsy.  **Response 3:** In cervical cancer screening, if a patient aged 21 to 24 years has a histopathological confirmation (biopsy) of LSIL cytology findings of ASC-H or higher and a TZ3 classification for colposcopy, the patient should be referred to a gynecologic oncologist for further evaluation and management. The patient should undergo a repeat colposcopy with biopsy, and if the biopsy results are consistent with high-grade squamous intraepithelial lesion (HSIL) or cervical cancer, the patient should be referred to a gynecologic oncologist for further evaluation and management. | | 78 | 94 | | | | 64 | Response3 |
| Phi-2  (without prompt) | **Response 1:** If a patient aged 21 to 24 has a histopathological confirmation of LSIL, cytology findings of ASC-H or higher, and a tri-color zone 3 classification for colposcopy, it's crucial to schedule a colposcopy examination. This procedure allows for a detailed visual inspection of the cervix under magnification, helping to identify any abnormalities that may warrant further investigation or treatment. **Response 2:** After the children and adolescent consultation is done, and if a pregnant patient with a diagnosis of LSIL, cytology findings of ASC-H or higher, and a TZ3 classification for colposcopy is detected, then colposcopy should be conducted as the cervical lesion has not reverted to LSIL. colposcopy should be considered as an initial scan with repeat investigation at 12 weeks during the scan, and then the result of the scan will be interpreted  **Response 3:** .If a patient aged 21 to 24 years has a histopathological confirmation(biopsy) of LSIL, cytology findings of ASC-H or higher, and a TZ1/2 classification for colposcopy, the patient should be referred for colposcopy. | | 70 | 80 | | | | 60 | Response 1 |
| QiZhenGPT  (with prompt) | **Response 1:** In cervical cancer screening, if a patient aged 21 to 24 years has a histopathological confirmation (biopsy) of LSIL cytology findings of ASC-H or higher and a TZ3 classification for colposcopy, the next step would be to perform a further colposcopy with a TZ4 classification.  **Response 2:** Follow-up steps should be conducted to evaluate the patient for any potential complications or risks associated with the procedure. The patient should also be referred to a specialist for further evaluation and management.  **Response 3:** Follow-up steps should be conducted to evaluate the patient for any potential complications or risks associated with the LSILcythemia. This may include performing a colposcopy or other imaging studies to assess the site of thecythemia and to identify any potential complications such as bleeding or infection. | | 71 | 54 | | | | 78 | Response3 |
| QiZhenGPT  (without prompt) | **Response 1:** in cervical screening, if a patient 21 to 24 years of age has histopathologic confirmation of LSIL (biopsy), a cervical cytology finding of ASC-H or higher, and a TZ3 classification, what follow-up steps should be performed?  A: If a patient 21 to 24 years of age has histopathologic confirmation of LSIL on cervical screening, ASC-H or higher on cervical cytology, and a TZ3 classification, the following follow-up steps should be performed:  * Cervical cytology and/or colposcopy every 6-12 months  * Cervical cytology and/or colposcopy every 1-2 years  * Consider cervical biopsy to evaluate for cancer if cervical cytology results are consistently ASC-H or higher **Response 2:** In cervical cancer screening, what follow-up steps should be performed if a patient 21 to 24 years of age has pathologic confirmation of LSIL (biopsy), a cervical cytology finding of ASC-H or higher, and a TZ3 classification for colposcopy?  A: If a patient 21 to 24 years of age has pathologic confirmation of LSIL on cervical cancer screening (biopsy), ASC-H or higher on cervical cytology, and TZ3 classification for colposcopy, the following follow-up steps should be performed: first perform cervical cytology and HPV testing, perform a cervical biopsy every 6 to 12 months, and Perform regular physical exams.  **Response 3:** In cervical screening, if a patient between 21 and 24 years of age has pathologically confirmed (biopsy) findings of LSIL, cytologic findings of ASC-H or higher, and a TZ3 classification by colposcopy, then the following follow-up steps should be performed:  - Cytology and colposcopy should be performed regularly in order to monitor the progression of the lesion.  - If cytology results are consistently ASC-H or higher, consider performing a LEEP biopsy to further evaluate the extent of the lesion.  - For TZ3 classification, more frequent follow-up may be necessary to allow timely detection of lesion progression. | | 60 | 80 | | | | 50 | Response 3 |
| **46.In cervical cancer screening, if a patient aged 21 to 24 years has a histopathological confirmation (biopsy) of LSIL cytology findings of ASCH or higher and a TZ1/2 classification for colposcopy, what followup steps should be conducted?** | | | | | | | | | |
| Phi-2  (with prompt) | **Response 1:** In cervical cancer screening, if a patient aged 21 to 24 years has a histopathological confirmation (biopsy) of LSIL cytology findings of ASC-H or higher and a TZ1/2 classification for colposcopy, the patient should be referred to a gynecologic oncologist for further evaluation and management. The patient should undergo a colposcopy to assess the extent of the abnormal cells and determine the need for additional treatment, such as cryotherapy, LEEP, or conization. The patient should also be counseled on the risk of developing cervical cancer and the importance of regular follow-up screenings.  **Response 2:** If a patient aged 21 to 24 years has a histopathological confirmation (biopsy) of LSIL cytology findings of ASC-H or higher and a TZ1/2 classification for colposcopy, the next step is to perform a colposcopy. If the colposcopy reveals no abnormalities, the patient can be rescreened in 3 years. If the colposcopy reveals abnormalities, the patient should undergo a cone biopsy.  **Response 3:** In cervical cancer screening, if a patient aged 21 to 24 years has a histopathological confirmation (biopsy) of LSIL cytology findings of ASC-H or higher and a TZ1/2 classification for colposcopy, the patient should be referred to a gynecologic oncologist for further evaluation and management. The patient should undergo a repeat colposcopy with biopsy, and if the biopsy results are consistent with high-grade squamous intraepithelial lesion (HSIL) or cervical cancer, the patient should be referred to a gynecologic oncologist for further evaluation and management. | | 83 | 97 | | | | 65 | Response1 |
| Phi-2  (without prompt) | **Response 1:** Similarly, if a patient aged 21 to 24 has a histopathological confirmation of LSIL, cytology findings of ASC-H or higher, and a tri-color zone 1/2 classification for colposcopy, scheduling a colposcopic examination becomes necessary. Colposcopy enables a comprehensive assessment of the cervix, aiding in determining whether any interventions are needed. **Response 2:** If the colposcopy of LSIL or other pathology background lesion shows a semipermanent HSIL or worse, treatments can be completed via antileukemic/cytotoxic drugs and local examination like punch and cone biopsy. **Response 3:** If a patient aged 21 to 24 years has a histopathological confirmation(biopsy) of LSIL, cytology findings of ASC-H or higher, and a TZ1/2 classification for colposcopy, the patient should be referred for colposcopy. | | 0 | 81 | | | | 7 | Response 1 |
| QiZhenGPT  (with prompt) | **Response 1:** In cervical cancer screening, if a patient aged 21 to 24 years has a histopathological confirmation (biopsy) of LSIL cytology findings of ASC-H or higher and a TZ1/2 classification for colposcopy, the next step would be to perform a further colposcopy with a TZ2 classification.  **Response 2:** Follow-up steps should be conducted to evaluate the patient for any potential complications or risks associated with the procedure. The patient should also be referred to a specialist for further evaluation and management.  **Response 3:** Follow-up steps should be conducted to evaluate the patient for any potential complications or risks associated with the LSILcythemia. This may include performing a colposcopy or other imaging studies to assess the site of thecythemia and to identify any potential complications such as bleeding or infection. | | 70 | 67 | | | | 89 | Response2 |
| QiZhenGPT  (without prompt) | **Response 1:** in cervical screening, if a patient aged 21 to 24 years has a histopathological confirmation (biopsy) of LSIL, cytology findings of ASC-H or higher, and a TZ3 classification for colposcopy, what follow-up steps should be conducted?  A: If a patient 21 to 24 years of age has histopathologic confirmation of LSIL on cervical screening, cytology findings of ASC-H or higher, and a TZ3 classification for colposcopy, the following follow-up steps should be conducted:  * Cervical cytology and/or colposcopy every 6-12 months  * Cervical cytology and/or colposcopy every 1-2 years  * Consider cervical biopsy to evaluate for cancer if cervical cytology results are consistently ASC-H or higher **Response 2:** In cervical cancer screening, what follow-up steps should be performed if a patient aged 21 to 24 years has pathologic confirmation of LSIL (biopsy), a cervical cytology finding of ASC-H or higher, and a TZ1/2 classification for colposcopy?  A: If a patient between the ages of 21 and 24 has pathologic confirmation of LSIL on cervical cancer screening (biopsy), ASC-H or higher on cervical cytology, and TZ1/2 classification for colposcopy, the following follow-up steps should be performed: first, cervical cytology and HPV testing, cervical biopsy every 6-12 months, and and a regular physical examination. **Response 3:** In cervical screening, if a patient 21 to 24 years of age has pathologically confirmed (biopsy) findings of LSIL, cytologic findings of ASC-H or higher, and a TZ1/2 classification on colposcopy, then the following follow-up steps should be performed:  - Cytology and colposcopy should be performed at regular intervals in order to monitor the progression of the lesion.  - If cytology results are consistently ASC-H or higher, consider a LEEP biopsy to further evaluate the extent of the lesion.  - For TZ1/2 classification, more frequent follow-up may be necessary to allow timely detection of lesion progression. | | 75 | 63 | | | | 78 | Response 3 |
| **47.In cervical cancer screening, if a pregnant patient has a histopathological confirmation (biopsy) of LSIL cytology findings of ASCH or higher, what followup steps should be conducted?** | | | | | | | | | |
| Phi-2  (with prompt) | **Response 1:** In cervical cancer screening, if a pregnant patient has a histopathological confirmation (biopsy) of LSIL cytology findings of ASC-H or higher, the patient should be referred to a gynecologic oncologist for further evaluation and management. The patient should undergo a colposcopy to assess the extent of the abnormal cells and determine the need for additional treatment, such as cryotherapy, LEEP, or conization. The patient should also be counseled on the risk of developing cervical cancer and the importance of regular follow-up screenings. The patient should be informed that the treatment options available during pregnancy may be different from those available to non-pregnant patients, and that the risks and benefits of each treatment option should be carefully considered. The patient should also be informed that the treatment options available during pregnancy may be different from those available to non-pregnant patients, and that the risks and benefits of each treatment option should be carefully considered.  **Response 2:** If a pregnant patient has a histopathological confirmation (biopsy) of LSIL cytology findings of ASC-H or higher, the next step is to perform a colposcopy. If the colposcopy reveals no abnormalities, the patient can be rescreened in 3 years. If the colposcopy reveals abnormalities, the patient should undergo a cone biopsy.  **Response 3:** In cervical cancer screening, if a pregnant patient has a histopathological confirmation (biopsy) of LSIL cytology findings of ASC-H or higher, the patient should be referred to a gynecologic oncologist for further evaluation and management. The patient should undergo a repeat colposcopy with biopsy, and if the biopsy results are consistent with high-grade squamous intraepithelial lesion (HSIL) or cervical cancer, the patient should be referred to a gynecologic oncologist for further evaluation and management. | | 75 | 85 | | | | 80 | Response1 |
| Phi-2  (without prompt) | **Response 1:** When it comes to pregnant patients who have a histopathological confirmation of LSIL, cytology findings of ASC-H or higher, there might not be immediate implications for pregnancy outcomes unless other complications arise. Regular prenatal care and monitoring would typically suffice. If any concerns arise during pregnancy, healthcare providers would evaluate them individually. **Response 2:** If you are pregnant and have a histopathological diagnosis(biopsy) of LSIL, then follow-up HSIL/ASCC-H examinations from colposcopy are recommended. **Response 3:** If a pregnant patient has a histopathological confirmation(biopsy) of LSIL, cytology findings of ASC-H or higher, the patient should be referred for colposcopy. | | 25 | 75 | | | | 0 | Response 1 |
| QiZhenGPT  (with prompt) | **Response 1:** In cervical cancer screening, if a pregnant patient has a histopathological confirmation (biopsy) of LSIL cytology findings of ASC-H or higher, the next step would be to perform a further colposcopy to evaluate the results and to determine the appropriate treatment plan.  **Response 2:** Follow-up steps should be conducted to evaluate the patient for any potential complications or risks associated with the procedure. The patient should also be referred to a specialist for further evaluation and management.  **Response 3:** Follow-up steps should be conducted to evaluate the patient for any potential complications or risks associated with the LSILcythemia. This may include performing a colposcopy or other imaging studies to assess the site of thecythemia and to identify any potential complications such as bleeding or infection. If the patient is pregnant, follow-up steps should be conducted to evaluate the potential risks and benefits of any treatment or intervention. | | 76 | 64 | | | | 74 | Response1 |
| QiZhenGPT  (without prompt) | **Response 1:** In cervical screening, if a pregnant woman has histopathologic confirmation of LSIL (biopsy), a cervical cytology finding of ASC-H or higher, and a TZ3 classification, what follow-up steps should be performed?  A: If a pregnant woman has histopathologic confirmation of LSIL on cervical screening, cervical cytology finding of ASC-H or higher, and TZ3 classification, the following follow-up steps should be performed:  * Cervical cytology and/or colposcopy every 6-12 months  * Cervical cytology and/or colposcopy every 1-2 years  * If cervical cytology results are consistently ASC-H or higher, a cervical biopsy should be considered to evaluate for carcinoma **Response 2:** In cervical cancer screening, if a pregnant woman has pathologic confirmation of LSIL (biopsy) and cervical cytology reveals ASC-H or higher, what follow-up steps should be performed?  A. If a pregnant woman has pathologically confirmed LSIL (biopsy) and cervical cytology reveals ASC-H or higher at cervical cancer screening, the following follow-up steps should be performed: cervical cytology and HPV testing first, cervical biopsy every 6-12 months, and regular physical examination. **Response 3:** in cervical screening, if a pregnant woman with pathologically confirmed (biopsy) findings of LSIL has cytologic findings of ASC-H or higher, then the following follow-up steps should be performed:  - Cytology and colposcopy should be performed regularly in order to monitor the progression of the lesion.  - If cytology results are consistently ASC-H or higher, consider a LEEP biopsy to further evaluate the extent of the lesion.  - More frequent follow-up visits may be necessary depending on the health status of the pregnant woman and the advice of her physician. | | 70 | 65 | | | | 80 | Response 3 |
| **48.In cervical cancer screening, if a patient has a histopathological confirmation (biopsy) of HSIL and a TZ1/2 classification, what followup steps should be conducted?** | | | | | | | | | |
| Phi-2  (with prompt) | **Response 1:** If a patient has a histopathological confirmation (biopsy) of HSIL and a TZ1/2 classification, the patient should be referred for colposcopy. If the colposcopy is normal, the patient should be re-screened in 3 years. If the colposcopy is abnormal, the patient should be referred for a cone biopsy.  **Response 2:** If a patient has a histopathological confirmation (biopsy) of HSIL and a TZ1/2 classification, the patient should undergo colposcopy. If the colposcopy is normal, the patient should be rescreened in 3 years. If the colposcopy is abnormal, the patient should undergo a repeat biopsy. If the repeat biopsy is normal, the patient should be rescreened in 3 years. If the repeat biopsy is abnormal, the patient should undergo treatment.  **Response 3:** If a patient has a histopathological confirmation (biopsy) of HSIL and a TZ1/2 classification, the patient should undergo colposcopy and be treated with cryotherapy or laser therapy. If the patient has a TZ3 classification, the patient should undergo colposcopy and be treated with cryotherapy or laser therapy. If the patient has a TZ3 classification and a biopsy shows invasive cancer, the patient should undergo radical hysterectomy and bilateral salpingo-oophorectomy. | | 89 | 66 | | | | 62 | Response1 |
| Phi-2  (without prompt) | **Response 1:** If a patient has a histopathological confirmation (biopsy) of high-grade squamous intraepithelial lesion (HSIL), which is classified as Tz1/2 in cervical cancer screening, then further examination and treatment are required. In general, patients who have received negative biopsies will undergo repeat cytology every 3 months until they reach 5 consecutive normal results; if there is any abnormality detected at this time point, it may need to be repeated immediately; If the result is abnormal again after one year, a repeat biopsy can be performed. After five consecutive normal results, annual testing is sufficient.  **Response 2:** If a patient has a histopathological confirmation of HSIL, and a TZ1/2 classification, follow-up steps should include a repeat Pap test in 6 months and a colposcopy in 1 year **Response 3:** If a patient has a histopathological confirmation(biopsy) of HSIL ,and a TZ1/2 classification, the follow-up steps should be conducted as follows.-The patient should be referred to a gynecologist for further evaluation and management.  -The patient should undergo colposcopy to examine the cervix in detail.  -If necessary, the patient may undergo a biopsy to obtain a tissue sample for further analysis.  -If the patient has a high-grade squamous intraepithelial lesion (HSIL), the patient should be referred to a gynecologic oncologist for further evaluation and management.  -The patient should undergo regular follow-up visits to monitor the condition of the cervix and to detect any changes or abnormalities. | | 85 | 65 | | | | 75 | Response 1 |
| QiZhenGPT  (with prompt) | **Response 1:** In cervical cancer screening, if a patient has a histopathological confirmation (biopsy) of HSIL and a TZ1/2 classification, the following follow-up steps should be conducted:  a. Follow-uposcopy: This involves performing a colposcopy or a standardoscopyoscopy on the patient to visualize the margins of the biopsy and to identify any potential cancer cells that may have been missed during the biopsy.  b. Follow-up DNA testing: If the histopathological confirmation is TZ1/2, the patient may need to undergo follow-up DNA testing to confirm the diagnosis and to determine the risk of developing cervical cancer in the future.  c. Follow-up treatment: If the patient is at high risk of developing cervical cancer, additional treatment may be recommended, such as radiation therapy or chemotherapy.  d. Follow-up care: The patient should continue to receive follow-up care, including regular check-ups with a gynecologist and any additional testing that may be needed to monitor the patient's health.  **Response 2:** In cervical cancer screening, if a patient has a histopathological confirmation (biopsy) of HSIL and a TZ1/2 classification, the following follow-up steps should be conducted:  a. Follow-uposcopy: This involves performing a colposcopy or a direct oscopyoscopy of the gastrointestinal tract to visualize any abnormalities or changes in the skin or internal organs.  b. Follow-up DNA testing: If the histopathological confirmation is HSIL, the patient may need to undergo DNA testing to determine if they have a risk of developing cervical cancer.  c. Follow-up treatment: If the patient has a TZ1/2 classification, they may need to undergo a treatment plan that includes radiation therapy or a combination of radiation therapy and chemotherapy.  d. Follow-up care: The patient should continue to receive follow-up care and monitoring for any changes in their health.  **Response 3:** In cervical cancer screening, if a patient has a histopathological confirmation (biopsy) of HSIL and a TZ1/2 classification, the following follow-up steps should be conducted:  a. Follow-uposcopy: This involves performing a colposcopy or a direct oscopyoscopy of the gastrointestinal tract to visualize any changes in the cervical cells.  b. Cervical cancer treatment: If the patient is found to have cervical cancer, the treatment plan will depend on the stage of the disease. The patient may receive a combination of surgery, radiation therapy, and chemotherapy.  c. Follow-up: The patient should be followed up regularly to monitor the progress of the disease and to detect any recurrence.  d. Follow-up: The patient should be followed up regularly to monitor the progress of the disease and to detect any recurrence. | | 73 | 43 | | | | 57 | Response1 |
| QiZhenGPT  (without prompt) | **Response 1:** If the patient has pathologic confirmation (biopsy) of HSIL on cervical screening and is classified as TZ1/2, the following follow-up steps should be performed:  * Perform cytologic follow-up, including cervical cytology (PAP smear) and/or liquid-based cytology (Vaginal cells cytology) every 3-6 months.  * If cytologic follow-up shows persistent abnormalities, further testing, such as a cervical biopsy, may be indicated. **Response 2:** If the patient has pathologically confirmed (biopsy) HSIL on cervical screening and is classified as TZ1/2, the following follow-up steps should be performed:  * Perform cytologic follow-up, including cervical cytology every 3-6 months (pap smear)  * Perform colposcopy, every 6-12 months  * Consider colposcopy every 12-18 months if two consecutive cytology follow-ups have normal results **Response 3:** If the patient has pathologic confirmation of HSIL on cervical screening (biopsy) and is categorized as TZ1/2, then the following follow-up steps should be performed:  * Perform cytologic follow-up, including cervical cytology (pap smear) every 3-6 months  * Perform colposcopy, every 6-12 months  * Consider colposcopy every 12-18 months if two consecutive cervical cytology tests are normal | | 73 | 96 | | | | 80 | Response 3 |
| **49.In cervical cancer screening, if a patient has a histopathological confirmation (biopsy) of HSIL and a TZ3 classification, what followup steps should be conducted?** | | | | | | | | | |
| Phi-2  (with prompt) | **Response 1:** If a patient has a histopathological confirmation (biopsy) of HSIL and a TZ3 classification, the patient should be referred for colposcopy. If the colposcopy is normal, the patient should be re-screened in 3 years. If the colposcopy is abnormal, the patient should be referred for a cone biopsy.  **Response 2:** If a patient has a histopathological confirmation (biopsy) of HSIL and a TZ3 classification, the patient should undergo colposcopy. If the colposcopy is normal, the patient should be rescreened in 3 years. If the colposcopy is abnormal, the patient should undergo a repeat biopsy. If the repeat biopsy is normal, the patient should be rescreened in 3 years. If the repeat biopsy is abnormal, the patient should undergo treatment.  **Response 3:** If a patient has a histopathological confirmation (biopsy) of HSIL and a TZ3 classification, the patient should undergo colposcopy and be treated with cryotherapy or laser therapy. If the patient has a TZ3 classification and a biopsy shows invasive cancer, the patient should undergo radical hysterectomy and bilateral salpingo-oophorectomy. | | 88 | 52 | | | | 55 | Response 1 |
| Phi-2  (without prompt) | **Response 1:** If a patient has a histopathological confirmation (biopsy) of high-grade squamous intraepithelial lesion (HSIL), which is classified as Tz3 in cervical cancer screening, then further examination and treatment are required. Similar to Tz1/2, patients who have received negative biopsies will undergo repeat cytology every 3 months until they reach 5 consecutive normal results; if there is any abnormality detected at this time point, it may need to be repeated immediately; If the result is abnormal again after one year, a repeat biopsy can be performed. After five consecutive normal results, annual testing is sufficient.  **Response 2:** If a patient has a histopathological confirmation of HSIL, and a TZ3 classification, follow-up steps should include a repeat Pap test in 6 months and a colposcopy in 1 year. **Response 3:** If a patient has a histopathological confirmation(biopsy) of HSIL, and a TZ3 classification, the follow-up steps should be conducted as follows:  -The patient should be referred to a gynecologist for further evaluation and management.  -The patient should undergo colposcopy to examine the cervix in detail.  -If necessary, the patient may undergo a biopsy to obtain a tissue sample for further analysis.  -If the patient has a high-grade squamous intraepithelial lesion (HSIL), the patient should be referred to a gynecologic oncologist for further evaluation and management.  -The patient should undergo regular follow-up visits to monitor the condition of the cervix and to detect any changes or abnormalities. | | 25 | 10 | | | | 15 | Response 1 |
| QiZhenGPT  (with prompt) | **Response 1:** In cervical cancer screening, if a patient has a histopathological confirmation (biopsy) of HSIL and a TZ3 classification, the following follow-up steps should be conducted:  a. Follow-uposcopy: This involves performing a colposcopy or a standardoscopyoscopy on the patient to visualize the margins of the biopsy and to identify any potential cancer cells that may have been missed during the biopsy.  b. Follow-up DNA testing: If the histopathological confirmation is TZ3, the patient may need to undergo follow-up DNA testing to confirm the diagnosis and to determine the risk of developing cervical cancer in the future.  c. Follow-up treatment: If the patient is at high risk of developing cervical cancer, additional treatment may be recommended, such as radiation therapy or chemotherapy.  d. Follow-up care: The patient should continue to receive follow-up care, including regular check-ups with a gynecologist and any additional testing that may be needed to monitor the patient's health.  **Response 2:** In cervical cancer screening, if a patient has a histopathological confirmation (biopsy) of HSIL and a TZ3 classification, the following follow-up steps should be conducted:  a. Follow-uposcopy: This involves performing a colposcopy or a direct oscopyoscopy of the gastrointestinal tract to visualize any abnormalities or changes in the skin or internal organs.  b. Follow-up DNA testing: If the histopathological confirmation is HSIL, the patient may need to undergo DNA testing to determine if they have a risk of developing cervical cancer.  c. Follow-up treatment: If the patient has a TZ3 classification, they may need to undergo a treatment plan that includes radiation therapy or a combination of radiation therapy and chemotherapy.  d. Follow-up care: The patient should continue to receive follow-up care and monitoring for any changes in their health.  **Response 3:** In cervical cancer screening, if a patient has a histopathological confirmation (biopsy) of HSIL and a TZ3 classification, the following follow-up steps should be conducted:  a. Follow-uposcopy: This involves performing a colposcopy or a direct oscopyoscopy of the gastrointestinal tract to visualize any changes in the cervical cells.  b. Cervical cancer treatment: If the patient is found to have cervical cancer, the treatment plan will depend on the stage of the disease. The patient may receive a combination of surgery, radiation therapy, and chemotherapy.  c. Follow-up: The patient should be followed up regularly to monitor the progress of the disease and to detect any recurrence.  d. Follow-up: The patient should be followed up regularly to monitor the progress of the disease and to detect any recurrence. | | 97 | 56 | | | | 64 | Response 1 |
| QiZhenGPT  (without prompt) | **Response 1:** if the patient has pathologically confirmed (biopsy) HSIL on cervical screening and is classified as TZ3, the following follow-up steps should be performed:  * Perform cytologic follow-up, including cervical cytology (PAP smear) and/or liquid-based cytology (Vaginal cells cytology) every 3-6 months.  * If cytology follow-up shows persistent abnormalities, further investigations such as cervical biopsy may be required. **Response 2:** if the patient has pathologically confirmed (biopsy) HSIL on cervical screening and is classified as TZ3, the following follow-up steps should be performed:  * Perform cytologic follow-up, including cervical cytology (pap smear) every 3-6 months  * Perform colposcopy, every 3-6 months  * Consider colposcopy every 12-18 months if three consecutive cytologic follow-ups are normal  **Response 3:** if the patient has pathologic confirmation of HSIL on cervical screening (biopsy) and is categorized as TZ3, then the following follow-up steps should be performed:  * Perform cytologic follow-up, including cervical cytology every 3-6 months (pap smear)  * Perform colposcopy, every 6-12 months  * Perform HPV viral testing  * Consider colposcopy every 12-18 months if two consecutive cervical cytology tests have normal results | | 85 | 80 | | | | 70 | Response 2 |
| **50.In cervical cancer screening, if a patient aged 21 to 24 years has a histopathological confirmation (biopsy) of CIN III/HSIL, what followup steps should be conducted?** | | | | | | | | | |
| Phi-2  (with prompt) | **Response 1:** If a patient aged 21 to 24 years has a histopathological confirmation (biopsy) of CIN III/HSIL, the patient should be referred for colposcopy. If the colposcopy is normal, the patient should be re-screened in 3 years. If the colposcopy is abnormal, the patient should be referred for a cone biopsy.  **Response 2:** If a patient aged 21 to 24 years has a histopathological confirmation (biopsy) of CIN III/HSIL, the patient should undergo colposcopy. If the colposcopy is normal, the patient should be rescreened in 3 years. If the colposcopy is abnormal, the patient should undergo a repeat biopsy. If the repeat biopsy is normal, the patient should be rescreened in 3 years. If the repeat biopsy is abnormal, the patient should undergo treatment.  **Response 3:** If a patient aged 21 to 24 years has a histopathological confirmation (biopsy) of CIN III/HSIL, the patient should undergo colposcopy and be treated with cryotherapy or laser therapy. If the patient has a TZ1/2 classification for colposcopy, the patient should undergo cryotherapy or laser therapy. If the patient has a TZ1/2 classification for colposcopy and a biopsy shows invasive cancer, the patient should undergo radical hysterectomy and bilateral salpingo-oophorectomy. | | 90 | 60 | | | | 70 | Response2 |
| Phi-2  (without prompt) | **Response 1:** If a patient aged between 21 and 24 years has a histopathological confirmation (biopsy) of low-grade squamous intraepithelial lesion (CIN III/HSIL), then regular surveillance through Pap smear is recommended once every three years. In addition, HPV DNA testing may also be used.  **Response 2:** If a patient aged 21 to 24 years has a histopathological confirmation of CIN III/HSIL, follow-up steps should include a repeat Pap test in 6 months and a colposcopy in 1 year. **Response 3:** If a patient aged 21 to 24 years has a histopathological confirmation(biopsy) of CIN III/HSIL, what follow-up steps should be conducted:  -The patient should be referred to a gynecologist for further evaluation and management.  -The patient should undergo colposcopy to examine the cervix in detail.  -If necessary, the patient may undergo a biopsy to obtain a tissue sample for further analysis.  -The patient should undergo regular follow-up visits to monitor the condition of the cervix and to detect any changes or abnormalities. | | 50 | 10 | | | | 40 | Response 1 |
| QiZhenGPT  (with prompt) | **Response 1:** In cervical cancer screening, if a patient aged 21 to 24 years has a histopathological confirmation (biopsy) of CIN III/HSIL, the following follow-up steps should be conducted:  a. Follow-uposcopy: This involves performing a colposcopy or a standardoscopyoscopy on the patient to visualize the margins of the biopsy and to identify any potential cancer cells that may have been missed during the biopsy.  b. Follow-up DNA testing: If the histopathological confirmation is CIN III/HSIL, the patient may need to undergo follow-up DNA testing to confirm the diagnosis and to determine the risk of developing cervical cancer in the future.  c. Follow-up treatment: If the patient is at high risk of developing cervical cancer, additional treatment may be recommended, such as radiation therapy or chemotherapy.  d. Follow-up care: The patient should continue to receive follow-up care, including regular check-ups with a gynecologist and any additional testing that may be needed to monitor the patient's health.  **Response 2:** In cervical cancer screening, if a patient aged 21 to 24 years has a histopathological confirmation (biopsy) of CIN III/HSIL, the following follow-up steps should be conducted:  a. Follow-uposcopy: This involves performing a colposcopy or a direct oscopyoscopy of the gastrointestinal tract to visualize any abnormalities or changes in the skin or internal organs.  b. Follow-up DNA testing: If the histopathological confirmation is CIN III/HSIL, the patient may need to undergo DNA testing to determine if they have a risk of developing cervical cancer.  c. Follow-up treatment: If the patient has a CIN III/HSIL classification, they may need to undergo a treatment plan that includes radiation therapy or a combination of radiation therapy and chemotherapy.  d. Follow-up care: The patient should continue to receive follow-up care and monitoring for any changes in their health.  **Response 3:** In cervical cancer screening, if a patient aged 21 to 24 years has a histopathological confirmation (biopsy) of CIN III/HSIL, the following follow-up steps should be conducted:  a. Follow-uposcopy: This involves performing a colposcopy or a direct oscopyoscopy of the gastrointestinal tract to visualize any changes in the cervical cells.  b. Cervical cancer treatment: If the patient is found to have cervical cancer, the treatment plan will depend on the stage of the disease. The patient may receive a combination of surgery, radiation therapy, and chemotherapy.  c. Follow-up: The patient should be followed up regularly to monitor the progress of the disease and to detect any recurrence.  d. Follow-up: The patient should be followed up regularly to monitor the progress of the disease and to detect any recurrence. | | 87 | 55 | | | | 60 | Response1 |
| QiZhenGPT  (without prompt) | The semantics of all three responses are identical | | - | - | | | | - | - |
| **51.In cervical cancer screening, if a patient aged 21 to 24 years has a histopathological confirmation (biopsy) of HSIL and a TZ3 classification for colposcopy, what followup steps should be conducted?** | | | | | | | | | |
| Phi-2  (with prompt) | **Response 1:** If a patient aged 21 to 24 years has a histopathological confirmation (biopsy) of HSIL and a TZ3 classification for colposcopy, the patient should be referred for colposcopy. If the colposcopy is normal, the patient should be re-screened in 3 years. If the colposcopy is abnormal, the patient should be referred for a cone biopsy.  **Response 2:** If a patient aged 21 to 24 years has a histopathological confirmation (biopsy) of HSIL and a TZ3 classification for colposcopy, the patient should undergo colposcopy. If the colposcopy is normal, the patient should be rescreened in 3 years. If the colposcopy is abnormal, the patient should undergo a repeat biopsy. If the repeat biopsy is normal, the patient should be rescreened in 3 years. If the repeat biopsy is abnormal, the patient should undergo treatment.  **Response 3:** If a patient aged 21 to 24 years has a histopathological confirmation (biopsy) of HSIL and a TZ3 classification for colposcopy, the patient should undergo colposcopy and be treated with cryotherapy or laser therapy. If the patient has a TZ3 classification for colposcopy and a biopsy shows invasive cancer, the patient should undergo radical hysterectomy and bilateral salpingo-oophorectomy. | | 86 | 58 | | | | 72 | Response2 |
| Phi-2  (without prompt) | **Response 1:** If a patient aged between 21 and 24 years has a histopathological confirmation (biopsy) of high-grade squamous intraepithelial lesion (HSIL), and a TZ3 classification for colposcopy, then regular surveillance through Pap smear is recommended once every three years. In addition, HPV DNA testing may also be used. **Response 2:** If a patient aged 21 to 24 years has a histopathological confirmation of HSIL, and a TZ3 classification for colposcopy, follow-up steps should include a repeat Pap test in 6 months and a colposcopy in 1 year. **Response 3:** In cervical cancer screening, if a patient aged 21 to 24 years has a histopathological confirmation(biopsy) of HSIL, and a TZ3 classification for colposcopy, what follow-up steps should be conducted:  -The patient should be referred to a gynecologist for further evaluation and management.  -The patient should undergo colposcopy to examine the cervix in detail.  -If necessary, the patient may undergo a biopsy to obtain a tissue sample for further analysis.  -The patient should undergo regular follow-up visits to monitor the condition of the cervix and to detect any changes or abnormalities. | | 85 | 60 | | | | 50 | Response 1 |
| QiZhenGPT  (with prompt) | **Response 1:** In cervical cancer screening, if a patient aged 21 to 24 years has a histopathological confirmation (biopsy) of HSIL and a TZ3 classification for colposcopy, the following follow-up steps should be conducted:  a. Follow-uposcopy: This involves performing a colposcopy on the patient to visualize the margins of the biopsy and to identify any potential cancer cells that may have been missed during the biopsy.  b. Follow-up DNA testing: If the histopathological confirmation is TZ3, the patient may need to undergo follow-up DNA testing to confirm the diagnosis and to determine the risk of developing cervical cancer in the future.  c. Follow-up treatment: If the patient is at high risk of developing cervical cancer, additional treatment may be recommended, such as radiation therapy or chemotherapy.  d. Follow-up care: The patient should continue to receive follow-up care, including regular check-ups with a gynecologist and any additional testing that may be needed to monitor the patient's health.  **Response 2:** In cervical cancer screening, if a patient aged 21 to 24 years has a histopathological confirmation (biopsy) of HSIL and a TZ3 classification for colposcopy, the following follow-up steps should be conducted:  a. Follow-uposcopy: This involves performing a colposcopy or a direct oscopyoscopy of the gastrointestinal tract to visualize any abnormalities or changes in the skin or internal organs.  b. Follow-up DNA testing: If the histopathological confirmation is HSIL, the patient may need to undergo DNA testing to determine if they have a risk of developing cervical cancer.  c. Follow-up treatment: If the patient has a TZ3 classification, they may need to undergo a treatment plan that includes radiation therapy or a combination of radiation therapy and chemotherapy.  d. Follow-up care: The patient should continue to receive follow-up care and monitoring for any changes in their health.  **Response 3:** In cervical cancer screening, if a patient aged 21 to 24 years has a histopathological confirmation (biopsy) of HSIL and a TZ3 classification for colposcopy, the following follow-up steps should be conducted:  a. Follow-uposcopy: This involves performing a colposcopy or a direct oscopyoscopy of the gastrointestinal tract to visualize any changes in the cervical cells.  b. Cervical cancer treatment: If the patient is found to have cervical cancer, the treatment plan will depend on the stage of the disease. The patient may receive a combination of surgery, radiation therapy, and chemotherapy.  c. Follow-up: The patient should be followed up regularly to monitor the progress of the disease and to detect any recurrence.  d. Follow-up: The patient should be followed up regularly to monitor the progress of the disease and to detect any recurrence. | | 67 | 46 | | | | 47 | Response1 |
| QiZhenGPT  (without prompt) | The semantics of all three responses are identical | | - | - | | | | - | - |
| **52.In cervical cancer screening, if a patient aged 21 to 24 years has a histopathological confirmation (biopsy) of CIN II/III/HSIL or CIN II/HSIL and a TZ1/2 classification for colposcopy, what followup steps should be conducted?** | | | | | | | | | |
| Phi-2  (with prompt) | **Response 1:** If a patient aged 21 to 24 years has a histopathological confirmation (biopsy) of CIN II/III/HSIL or CIN II/HSIL and a TZ1/2 classification for colposcopy, the patient should be referred for colposcopy. If the colposcopy is normal, the patient should be re-screened in 3 years. If the colposcopy is abnormal, the patient should be referred for a cone biopsy.  **Response 2:** If a patient aged 21 to 24 years has a histopathological confirmation (biopsy) of CIN II/III/HSIL or CIN II/HSIL and a TZ1/2 classification for colposcopy, the patient should undergo colposcopy. If the colposcopy is normal, the patient should be rescreened in 3 years. If the colposcopy is abnormal, the patient should undergo a repeat biopsy. If the repeat biopsy is normal, the patient should be rescreened in 3 years. If the repeat biopsy is abnormal, the patient should undergo treatment.  **Response 3:** If a patient aged 21 to 24 years has a histopathological confirmation (biopsy) of CIN II/III/HSIL or CIN II/HSIL and a TZ1/2 classification for colposcopy, the patient should undergo colposcopy and be treated with cryotherapy or laser therapy. If the patient has a TZ1/2 classification for colposcopy and a biopsy shows invasive cancer, the patient should undergo radical hysterectomy and bilateral salpingo-oophorectomy. | | 86 | 62 | | | | 56 | Response2 |
| Phi-2  (without prompt) | **Response 1:** If a patient aged between 21 and 24 years has a histopathological confirmation (biopsy) of low-grade squamous intraepithelial lesion (CIN Ⅱ/III/HSIL or CIN Ⅱ/HSIL), and a TZ1/2 classification for colposcopy, then regular surveillance through Pap smear is recommended once every three years. In addition, HPV DNA testing may also be used. **Response 2:** If a patient aged 21 to 24 years has a histopathological confirmation of CINⅡ/III/HSIL or CINⅡ/HSIL, and a TZ1/2 classification for colposcopy, follow-up steps should include a repeat Pap test in 6 months and a colposcopy in 1 year. **Response 3:** In cervical cancer screening, if a patient aged 21 to 24 years has a histopathological confirmation(biopsy) of CINⅡ/III/HSIL or CINⅡ/HSIL, and a TZ1/2 classification for colposcopy, what follow-up steps should be conducted:  -The patient should be referred to a gynecologist for further evaluation and management.  -The patient should undergo colposcopy to examine the cervix in detail.  -If necessary, the patient may undergo a biopsy to obtain a tissue sample for further analysis.  -The patient should undergo regular follow-up visits to monitor the condition of the cervix and to detect any changes or abnormalities. | | 50 | 0 | | | | 0 | Response 1 |
| QiZhenGPT  (with prompt) | **Response 1:** In cervical cancer screening, if a patient aged 21 to 24 years has a histopathological confirmation (biopsy) of CIN II/III/HSIL or CIN II/HSIL and a TZ1/2 classification for colposcopy, the following follow-up steps should be conducted:  a. Follow-uposcopy: This involves performing a colposcopy on the patient to visualize the margins of the biopsy and to identify any potential cancer cells that may have been missed during the biopsy.  b. Follow-up DNA testing: If the histopathological confirmation is TZ1/2, the patient may need to undergo follow-up DNA testing to confirm the diagnosis and to determine the risk of developing cervical cancer in the future.  c. Follow-up treatment: If the patient is at low risk of developing cervical cancer, additional treatment may be recommended, such as radiation therapy or chemotherapy.  d. Follow-up care: The patient should continue to receive follow-up care, including regular check-ups with a gynecologist and any additional testing that may be needed to monitor the patient's health.  **Response 2:** In cervical cancer screening, if a patient aged 21 to 24 years has a histopathological confirmation (biopsy) of CIN II/III/HSIL or CIN II/HSIL and a TZ1/2 classification for colposcopy, the following follow-up steps should be conducted:  a. Follow-uposcopy: This involves performing a colposcopy or a direct oscopyoscopy of the gastrointestinal tract to visualize any abnormalities or changes in the skin or internal organs.  b. Follow-up DNA testing: If the histopathological confirmation is CIN II/III/HSIL or CIN II/HSIL, the patient may need to undergo DNA testing to determine if they have a risk of developing cervical cancer.  c. Follow-up treatment: If the patient has a TZ1/2 classification, they may need to undergo a treatment plan that includes radiation therapy or a combination of radiation therapy and chemotherapy.  d. Follow-up care: The patient should continue to receive follow-up care and monitoring for any changes in their health.  **Response 3:** In cervical cancer screening, if a patient aged 21 to 24 years has a histopathological confirmation (biopsy) of CIN II/III/HSIL or CIN II/HSIL and a TZ1/2 classification for colposcopy, the following follow-up steps should be conducted:  a. Follow-uposcopy: This involves performing a colposcopy or a direct oscopyoscopy of the gastrointestinal tract to visualize any changes in the cervical cells.  b. Cervical cancer treatment: If the patient is found to have cervical cancer, the treatment plan will depend on the stage of the disease. The patient may receive a combination of surgery, radiation therapy, and chemotherapy.  c. Follow-up: The patient should be followed up regularly to monitor the progress of the disease and to detect any recurrence.  d. Follow-up: The patient should be followed up regularly to monitor the progress of the disease and to detect any recurrence. | | 85 | 60 | | | | 25 | Response2 |
| QiZhenGPT  (without prompt) | The semantics of all three responses are identical | | - | - | | | | - | - |
| **53.In cervical cancer screening, if a pregnant patient has a histopathological confirmation (biopsy) of HSIL, what followup steps should be conducted?** | | | | | | | | | |
| Phi-2  (with prompt) | **Response 1:** In cervical cancer screening, if a pregnant patient has a histopathological confirmation (biopsy) of HSIL, the patient should be referred to a gynecologic oncologist for further evaluation and management. The patient should undergo a repeat Pap smear and HPV testing at 6 months post-biopsy. If the results of the repeat Pap smear and HPV testing are negative, the patient can be followed up with a Pap smear every 3 years. If the results of the repeat Pap smear and HPV testing are positive, the patient should undergo further evaluation and management, which may include colposcopy, biopsy, and treatment.  **Response 2:** If a pregnant patient has a histopathological confirmation (biopsy) of HSIL, the follow-up steps should be as follows:  - The patient should be referred to a gynecologic oncologist for further evaluation and management.  - The patient should undergo a repeat Pap smear at 6-12 months after the initial biopsy.  - If the repeat Pap smear is negative, the patient should be monitored every 3-5 years for cervical cancer.  - If the repeat Pap smear is positive, the patient should undergo further diagnostic testing, such as colposcopy and biopsy, to determine the extent of the disease.  **Response 3:** In cervical cancer screening, if a pregnant patient has a histopathological confirmation (biopsy) of HSIL, the patient should be referred to a gynecologic oncologist for further evaluation and management. The patient should undergo a repeat Pap smear at 6-12 months after the initial biopsy to assess for any changes in the cervical cells. If the Pap smear shows persistent HSIL or invasive cancer, further treatment options such as cryotherapy, laser therapy, or conization may be considered. | | 94 | 81 | | | | 96 | Response1 |
| Phi-2  (without prompt) | **Response 1:** Pregnant women with a histopathological confirmation (biopsy) of HSIL do not require additional examinations or treatments unless other abnormalities are found on ultrasound imaging. Regular monitoring through routine prenatal care is usually sufficient. **Response 2:** If a pregnant patient has a histopathological confirmation of HSIL, follow-up steps should include a repeat Pap test in 6 months and a colposcopy in 1 year. **Response 3:** In cervical cancer screening, if a pregnant patient has a histopathological confirmation(biopsy) of HSIL, what follow-up steps should be conducted:  -The patient should be referred to a gynecologist for further evaluation and management.  -The patient should undergo colposcopy to examine the cervix in detail.  -If necessary, the patient may undergo a biopsy to obtain a tissue sample for further analysis.  -The patient should be closely monitored throughout the pregnancy to ensure the health of both the mother and the baby. | | 0 | 0 | | | | 0 | Response 1 |
| QiZhenGPT  (with prompt) | **Response 1:** In cervical cancer screening, if a pregnant patient has a histopathological confirmation (biopsy) of HSIL, what follow-up steps should be conducted?  Answer: Follow-up with a routine cervical cancer screening test (CCT) to detect any new cases of cervical cancer in the future.  Follow-up with a routine ASC-US CT to detect any new cases of ASC-US in the future.  Follow-up with a routine HSIL CT to detect any new cases of HSIL in the future.  **Response 2:** In cervical cancer screening, if a pregnant patient has a histopathological confirmation (biopsy) of HSIL, what follow-up steps should be conducted?  a. Follow-up with a routine cervical cancer screening test (CCT) to detect any changes in the status of the patient's cervical cancer screening results.  b. Follow-up with a routine cervical cancer screening test (CCT) to detect any changes in the status of the patient's cervical cancer screening results after pregnancy.  c. Follow-up with a routine cervical cancer screening test (CCT) to detect any changes in the status of the patient's cervical cancer screening results after childbearing.  d. Follow-up with a routine cervical cancer screening test (CCT) to detect any changes in the status of the patient's cervical cancer screening results after delivery.  e. Follow-up with a routine cervical cancer screening test (CCT) to detect any changes in the status of the patient's cervical cancer screening results after any subsequent pregnancy or childbirth.  **Response 3:** In cervical cancer screening, if a pregnant patient has a histopathological confirmation (biopsy) of HSIL, what follow-up steps should be conducted?  Answer: Follow-up with a routine cervical cancer screening test (CCT) to detect any new cases of cervical cancer in the future.  Follow-up with a routine ASCUS/HSIL screening test to detect any new cases of ASCUS/HSIL in the future.  Follow-up with a routine cervicalcancer test to detect any new cases of cervicalcancer in the future. | | 100 | 83 | | | | 0 | Response1 |
| QiZhenGPT  (without prompt) | **Response 1:** If a pregnant woman has pathologically confirmed (biopsy) HSIL on cervical screening, the following follow-up steps should be performed:  * Perform cytologic follow-up, including cervical cytology (PAP smear) every 3-6 months.  * If follow-up shows persistent abnormalities, further investigations, such as cervical biopsy, may be required. **Response 2:** If a pregnant woman has pathologically confirmed (biopsy) HSIL on cervical screening, the following follow-up steps should be performed:  * Perform cytologic follow-up, including cervical cytology (pap smear) every 3-6 months  * Perform colposcopy, every 6-12 months  * Perform breastfeedingsupport.com/cervical-cancer-screening-after-pregnancy follow-up, including colposcopy every 12-18 months **Response 3:** if a pregnant woman has pathologic confirmation of HSIL on cervical screening (biopsy), then the following follow-up steps should be performed:  * Perform cytologic follow-up, including cervical cytology every 3-6 months (Pap smear)  * Perform colposcopy, every 6-12 months  * Perform HPV viral testing  * Consider colposcopy every 12-18 months if two consecutive cervical cytology tests have normal results | | 80 | 90 | | | | 70 | Response 3 |
| **54.After diagnostic/therapeutic cervical conization for cervical cancer, what followup steps should be conducted?** | | | | | | | | | |
| Phi-2  (with prompt) | The semantics of all three responses are identical | | - | - | | | | - | - |
| Phi-2  (without prompt) | **Response 1:** After diagnostic or therapeutic coneal excision surgery for cervical cancer, postoperative care includes observation for bleeding, infection, and complications related to anesthesia. Patients should avoid strenuous activities such as lifting heavy objects, sexual intercourse, and douching for several weeks after the operation. Follow-up appointments with the doctor are necessary to monitor healing progress and detect any potential problems early. **Response 2:** After Diagnostic/Therapeutic Cervical Conization for cervical cancer, follow-up steps should include a repeat Pap test in 6 months and a colposcopy in 1 year. **Response 3:** After Diagnostic/Therapeutic Cervical Conization for cervical cancer, what follow-up steps should be conducted:  -The patient should be referred to a gynecologist for further evaluation and management.  -The patient should undergo regular follow-up visits to monitor the condition of the cervix and to detect any changes or abnormalities.  -The patient should be advised to undergo regular Pap smears to monitor the health of the cervix.  -If necessary, the patient may undergo additional procedures, such as colposcopy or biopsy, to further evaluate the condition of the cervix. | | 20 | 35 | | | | 40 | Response 3 |
| QiZhenGPT  (with prompt) | **Response 1:** After diagnostic/therapeutic cervical conization for cervical cancer, what follow-up steps should be conducted?  Answer: Follow-up with a routine cervical cancer screening test (CCT) to detect any new cases of cervical cancer in the future.  Follow-up with a routine ASC-US CT to detect any new cases of ASC-US in the future.  Follow-up with a routine HSIL CT to detect any new cases of HSIL in the future.  **Response 2:** After diagnostic/therapeutic cervical conization for cervical cancer, what follow-up steps should be conducted?  Answer: a. Follow-up with a routine cervical cancer screening test (CCT) to detect any changes in the status of the patient's cervical cancer screening results.  b. Follow-up with a routine cervical cancer screening test (CCT) to detect any changes in the status of the patient's cervical cancer screening results after pregnancy.  c. Follow-up with a routine cervical cancer screening test (CCT) to detect any changes in the status of the patient's cervical cancer screening results after childbearing.  d. Follow-up with a routine cervical cancer screening test (CCT) to detect any changes in the status of the patient's cervical cancer screening results after delivery.  e. Follow-up with a routine cervical cancer screening test (CCT) to detect any changes in the status of the patient's cervical cancer screening results after any subsequent pregnancy or childbirth.  **Response 3:** After diagnostic/therapeutic cervical conization for cervical cancer, what follow-up steps should be conducted?  Answer: Follow-up with a routine cervical cancer screening test (CCT) to detect any new cases of cervical cancer in the future.  Follow-up with a routine ASCUS/HSIL screening test to detect any new cases of ASCUS/HSIL in the future.  Follow-up with a routine cervical cancer test to detect any new cases of cervical cancer in the future. | | 88 | 60 | | | | 63 | Response1 |
| QiZhenGPT  (without prompt) | **Response 1:** Blank **Response 2:** the following follow-up steps should be performed after cervical conization (Diagnostic/Therapeutic Cervical Conization):  * Perform cytologic follow-up, including cervical cytology every 3-6 months (Pap smear)  * Perform colposcopy, every 6-12 months  * Consider colposcopy every 12-18 months if two consecutive cytologic follow-ups are normal **Response 3:** Following cervical conization (Diagnostic/Therapeutic Cervical Conization), patients should undergo the following follow-up steps:  * Perform cytologic follow-up, including cervical cytology every 3-6 months (Pap smear)  * Perform colposcopy, every 6-12 months  * Perform HPV viral testing  * If two consecutive cervical cytology tests are normal, colposcopy every 12-18 months may be considered | | 60 | 90 | | | | 70 | Response 3 |
| **55.In cervical cancer screening, if a pregnant patient has a histopathological confirmation (biopsy) of HSIL with no invasive cancer during pregnancy, what followup steps should be conducted after childbearing?** | | | | | | | | | |
| Phi-2  (with prompt) | **Response 1:** In cervical cancer screening, if a pregnant patient has a histopathological confirmation (biopsy) of HSIL with no invasive cancer during pregnancy, the patient should be referred to a gynecologic oncologist for further evaluation and management. The patient should undergo a repeat Pap smear and HPV testing at 6 months post-biopsy. If the results of the repeat Pap smear and HPV testing are negative, the patient can be followed up with a Pap smear every 3 years. If the results of the repeat Pap smear and HPV testing are positive, the patient should undergo further evaluation and management, which may include colposcopy, biopsy, and treatment.  the size, location, and extent of the metastatic lesions. CT can also be used to evaluate the surrounding tissues and organs for signs of metastasis.  **Response 2:** If a pregnant patient has a histopathological confirmation (biopsy) of HSIL with no invasive cancer during pregnancy, the follow-up steps should be as follows:  - The patient should be referred to a gynecologic oncologist for further evaluation and management.  - The patient should undergo a repeat Pap smear at 6-12 months after the initial biopsy.  - If the repeat Pap smear is negative, the patient should be monitored every 3-5 years for cervical cancer.  - If the repeat Pap smear is positive, the patient should undergo further diagnostic testing, such as colposcopy and biopsy, to determine the extent of the disease.  - The patient should also undergo regular pelvic examinations and imaging studies, such as CT scans and MRI, to monitor for recurrence or spread of the disease.  **Response 3:** In cervical cancer screening, if a pregnant patient has a histopathological confirmation (biopsy) of HSIL with no invasive cancer during pregnancy, the patient should be closely monitored for any changes in the cervical cells. Regular follow-up visits with a gynecologic oncologist are recommended to assess for any changes in the cervical cells and to provide appropriate supportive care. The patient should also be educated about the importance of continued cervical cancer screening after pregnancy to detect any potential changes in the cervical cells. | | 92 | 76 | | | | 84 | Response1 |
| Phi-2  (without prompt) | **Response 1:** For a pregnant patient who has a histopathological confirmation (biopsy) of HSIL without invasive cancer during pregnancy, and no history of preterm labor, delivery, or fetal loss, the following measures can be taken after childbirth: First of all, perform an endocervical curettage and hysterectomy within 6 months to remove any residual tissue that could lead to recurrence. Secondly, conduct a pelvic examination and cervical cytology test annually for 5 years after the procedure, and repeat the test every 2 years thereafter. Thirdly, vaccinate against human papillomavirus (HPV). Fourthly, maintain a healthy lifestyle by avoiding smoking and excessive alcohol consumption, eating a balanced diet, exercising regularly, getting enough sleep, reducing stress levels, and attending routine checkups. **Response 2:** If a pregnant patient has a histopathological confirmation of HSIL, and no invasive cancer during pregnancy, after childbearing, follow-up steps should include a repeat Pap test in 6 months and a colposcopy in 1 year. **Response 3:** ,In cervical cancer screening, if a pregnant patient has a histopathological confirmation(biopsy) of HSIL, no invasive cancer during pregnancy, after childbearing, what follow-up steps should be conducted?  -The patient should be referred to a gynecologist for further evaluation and management.  -The patient should undergo regular follow-up visits to monitor the condition of the cervix and to detect any changes or abnormalities.  -The patient should be advised to undergo regular Pap smears to monitor the health of the cervix.  -If necessary, the patient may undergo additional procedures, such as colposcopy or biopsy, to further evaluate the condition of the cervix. | | 50 | 15 | | | | 30 | Response 2 |
| QiZhenGPT  (with prompt) | **Response 1:** In cervical cancer screening, if a pregnant patient has a histopathological confirmation (biopsy) of HSIL with no invasive cancer during pregnancy, what follow-up steps should be conducted after childbearing?  Answer: Follow-up with a routine ASC-US CT to detect any new cases of ASC-US in the future.  Follow-up with a routine HSIL CT to detect any new cases of HSIL in the future.  Follow-up with a routine cervical cancer screening test (CCT) to detect any new cases of cervical cancer in the future.  **Response 2:** In cervical cancer screening, if a pregnant patient has a histopathological confirmation (biopsy) of HSIL with no invasive cancer during pregnancy, what follow-up steps should be conducted after childbearing?  Answer: Follow-up with a routine cervical cancer screening test (CCT) to detect any changes in the status of the patient's cervical cancer screening results.  a. Follow-up with a routine cervical cancer screening test (CCT) to detect any changes in the status of the patient's cervical cancer screening results after pregnancy.  b. Follow-up with a routine cervical cancer screening test (CCT) to detect any changes in the status of the patient's cervical cancer screening results after childbearing.  c. Follow-up with a routine cervical cancer screening test (CCT) to detect any changes in the status of the patient's cervical cancer screening results after delivery.  d. Follow-up with a routine cervical cancer screening test (CCT) to detect any changes in the status of the patient's cervical cancer screening results after any subsequent pregnancy or childbirth.  **Response 3:** In cervical cancer screening, if a pregnant patient has a histopathological confirmation (biopsy) of HSIL with no invasive cancer during pregnancy, what follow-up steps should be conducted after childbearing?  Answer: Follow-up with a routine cervical cancer screening test (CCT) to detect any new cases of cervical cancer in the future.  Follow-up with a routine ASCUS/HSIL screening test to detect any new cases of ASCUS/HSIL in the future.  Follow-up with a routine cervicalcancer test to detect any new cases of cervicalcancer in the future. | | 100 | 53 | | | | 42 | Response2 |
| QiZhenGPT  (without prompt) | **Response 1:** if a pregnant woman has pathologically confirmed (biopsy) HSIL during pregnancy testing and no invasive cancer was found during pregnancy and is followed up after delivery, the following follow-up steps should be performed:  * Perform cytologic follow-up, including cervical cytology (PAP smear) every 3-6 months.  * If follow-up shows persistent abnormalities, further investigations such as cervical biopsy may be required. **Response 2:** If a pregnant woman has pathologically confirmed (biopsy) HSIL on cervical screening during pregnancy and no invasive cancers developed during pregnancy and is followed up after delivery, the following follow-up steps should be performed:  * Perform cytologic follow-up, including cervical cytology (pap smear) every 3-6 months  * Perform colposcopy, every 6-12 months  * Consider colposcopy every 12-18 months if two consecutive cytologic follow-ups are normal **Response 3:** if a pregnant woman has pathologic confirmation of HSIL on cervical screening during pregnancy and no invasive cancer was found during pregnancy, the following follow-up steps should be performed after delivery:  * Perform cytologic follow-up, including cervical cytology (pap smear) every 3-6 months  * Perform colposcopy, including colposcopy every 6-12 months  * Perform HPV viral testing  * Consider colposcopy every 12-18 months if two consecutive cervical cytology results are normal | | 90 | 70 | | | | 85 | Response 1 |
| **56: What are the strategies for HPV vaccine use? What are the recommended vaccination programs for different age groups?** | | | | | | | | | |
| Phi-2  (with prompt) | **Response 1:** The strategies for HPV vaccine use include vaccination of both males and females, vaccination of high-risk populations, and vaccination of individuals who have not been vaccinated before. The recommended vaccination programs for different age groups include vaccination of girls and boys aged 9-14 years, vaccination of girls aged 15-26 years, and vaccination of women aged 27-45 years who have not been vaccinated before.  **Response 2:** The strategies for HPV vaccine use include vaccination of both males and females, vaccination of high-risk populations, and vaccination of individuals who have not yet been exposed to HPV. The recommended vaccination programs for different age groups vary depending on the vaccine used and the country's guidelines. For example, in the United States, the HPV vaccine is recommended for both males and females aged 11-12 years, with catch-up vaccination for those aged 13-26 years. In Europe, the HPV vaccine is recommended for both males and females aged 9-26 years.  **Response 3:** The strategies for HPV vaccine use include vaccination of girls and boys at the age of 11-12 years, vaccination of women aged 27-45 years, and vaccination of women aged 26-45 years with a booster dose. The recommended vaccination programs for different age groups are based on the age of vaccination and the type of vaccine used. | | 85 | 70 | | | | 75 | Response |
| Phi-2  (without prompt) | The semantics of all three responses are identical | | - | - | | | | - | - |
| QiZhenGPT  (with prompt) | **Response 1:** Strategies for HPV vaccine use:  * Recommendation: All children and adolescents aged 12-18 should be vaccinated with the HPV 16 and 18 vaccine.  * Recommendation for adults: HPV 6 and 11 vaccines should be recommended for those aged 26-65 years.  * Recommendation for high-risk HPV vaccines: HPV 16 and 18 vaccines should be recommended for individuals with a history of low-risk HPV infection or who have had a previous HPV vaccine series.  * Recommendation for HPV 31, 33, 35, 45, and 52 vaccines: These vaccines should be recommended for individuals aged 26-65 years with a history of low-risk HPV infection or who have had a previous HPV vaccine series.  **Response 2:** Strategies for HPV vaccine use:  * Recommendation for HPV vaccine: All children and adolescents aged 12-18 years should be vaccinated with two doses of HPV vaccine (6 and 11).  * Recommended vaccination programs for different age groups:  + Children aged 12-15 years: two doses (6 and 11)  + adolescents aged 16-18 years: two doses (6 and 11)  + adults aged 19-65 years: one dose (11)  **Response 3:** Strategies for HPV vaccine use:  * Recommendation: All children and adolescents should be vaccinated with the HPV 16 and 18 vaccine.  * Recommended vaccination programs:  + Age group: 12-18 years  + Recommendation: All girls and women should be vaccinated at least once before the age of 26 years. | | 79 | 91 | | | | 84 | Response1 |
| QiZhenGPT  (without prompt) | **Response 1:** Strategies for HPV vaccine use include providing education and counseling to increase vaccination rates, offering incentives for healthcare providers and community leaders, and implementing policies and laws that support vaccination. Recommended vaccination programs for different age groups include the primary series of 3 doses for children under 15 years,  **Response 2:** In the diagnosis of cervical cancer, a transvaginal ultrasound is often the preferred imaging method to evaluate cervical tumors. This allows for a more accurate diagnosis and treatment of cervical precancerous lesions. For evaluating metastatic lesions, a CT or MRI scan may be used.  **Response 3:** Strategies for HPV vaccine use include providing education and counseling to increase vaccination rates, offering incentives for healthcare providers and community leaders, and implementing policies and laws that support vaccination. Recommended vaccination programs for different age groups include the primary series of 3 doses for children under 15 years, | | 64 | 100 | | | | 67 | Responsse1 |
| **57: Is HPV primary screening applicable in lowincome countries? If so, why?** | | | | | | | | | |
| Phi-2  (with prompt) | **Response 1:** HPV primary screening is not applicable in low-income countries due to the lack of resources and infrastructure required for the screening program.  **Response 2:** HPV primary screening is not applicable in low-income countries due to the lack of resources and infrastructure required for the screening program. In addition, the cost of the screening program may be prohibitive for low-income countries.  **Response 3:** HPV primary screening is not applicable in low-income countries due to the lack of resources and infrastructure required for the screening. | | 100 | 95 | | | | 98 | Response1 |
| Phi-2  (without prompt) | The semantics of all three responses are identical | | - | - | | | | - | - |
| QiZhenGPT  (with prompt) | **Response 1:** Is HPV primary screening applicable in low-income countries? If so, why?  * Yes, HPV primary screening is applicable in low-income countries.  * The main reason for applying HPV primary screening in low-income countries is to reduce the incidence and progression of cervical cancer.  **Response 2:** Is HPV primary screening applicable in low-income countries? If so, why?  * HPV primary screening is not recommended in low-income countries due to the lack of resources and infrastructure for testing and treatment.  * However, HPV-based cervical cancer screening programs (e.g., Cervical Cancer Screening Program in South Africa) are implemented in low-income countries to improve access to cancer screening and treatment.  **Response 3:** Is HPV primary screening applicable in low-income countries? If so, why?  * Recommendation: HPV primary screening should be considered in low-income countries where access to advanced cervical cancer screening methods is limited.  * Reason: HPV primary screening can provide early detection of cervical cancer and improve survival rates. | | 40 | 80 | | | | 60 | Response3 |
| QiZhenGPT  (without prompt) | **Response 1:** doses for adults aged 15-65, and 3 doses for adults aged 65 or older. 2. HPV primary screening is not applicable in low-income countries because it is expensive and requires significant resources.  **Response 2:** Strategies for HPV vaccine use include providing access to the vaccine, ensuring proper storage and handling, and promoting vaccination through education and community outreach. Recommended vaccination programs for different age groups include catch-up schedules for older adults who have missed the initial doses.  **Response 3:** doses for adults aged 15-69 years, and 3 doses for adults aged 70 years and older. 2. HPV primary screening is not applicable in low-income countries due to lack of resources, infrastructure, and access to healthcare. | | 0 | 95 | | | | 90 | Responsse1 |
| **58: which cervical cancer screening methods are widely used in lowincome countries, particularly in subSaharan Africa?** | | | | | | | | | |
| Phi-2  (with prompt) | **Response 1:** The Pap smear is the most widely used cervical cancer screening method in low-income countries, particularly in sub-Saharan Africa.  **Response 2:** The key cervical cancer screening methods used in low-income countries, particularly in sub-Saharan Africa, include visual inspection with acetic acid (VIA) and liquid-based cytology (LBC). These methods are cost-effective and can be performed by trained health workers in primary care settings.  **Response 3:** The Pap smear is the most widely used cervical cancer screening method in low-income countries, particularly in sub-Saharan Africa. | | 76 | 100 | | | | 44 | Response3 |
| Phi-2  (without prompt) | The semantics of all three responses are identical | | - | - | | | | - | - |
| QiZhenGPT  (with prompt) | The semantics of all three responses are identical | | - | - | | | | - | - |
| QiZhenGPT  (without prompt) | The semantics of all three responses are identical | | - | - | | | | - | - |
| **59: what are the key indicators included in the expert consensus for quality control management of HPV testing?** | | | | | | | | | |
| Phi-2  (with prompt) | The semantics of all three responses are identical | | - | - | | | | - | - |
| Phi-2  (without prompt) | **Response 1:** The key indicators included in the expert consensus for quality control management of HPV testing are as follows:  **Response 2:** The key indicators included in the expert consensus for quality control management of HPV testing are as follows:  **Response 3:** The key indicators included in the expert consensus for quality control management of HPV testing are as follows: | | 61 | 41 | | | | 55 | Response2 |
| QiZhenGPT  (with prompt) | **Response 1:** What are the key indicators included in the expert consensus for quality control management of HPV testing?  * Accuracy: The accuracy of the HPV testing should be at least 90%.  * Efficiency: The efficiency of the HPV testing should be at least 80%.  * Timeliness: The timeliness of the HPV testing should be at least 72 hours.  * Transparency: The information provided by the HPV testing should be transparent and easy to understand.  **Response 2:** What are the key indicators included in the expert consensus for quality control management of HPV testing?  * Key indicators for quality control management of HPV testing include:  + Accessibility and convenience of testing  + Timeliness of results  + Accuracy of results  + Timeliness of follow-up care  + Availability of support services for patients  **Response 3:** What are the key indicators included in the expert consensus for quality control management of HPV testing?  * Key indicators:  + Accuracy of HPV testing  + Timeliness of HPV testing  + Availability of HPV testing materials  + Accessibility of HPV testing to all women of reproductive age  + Consistency of HPV testing results  + Timeliness of follow-up for women with positive HPV testing results | | 88 | 60 | | | | 78 | Response2 |
| QiZhenGPT  (without prompt) | **Response 1:** Key indicators for quality control management of HPV testing include test accuracy, specificity, and sensitivity, as well as operator training and quality control procedures.  **Response 2:** Cervical cancer screening methods that are widely used in low-income countries, particularly in sub-Saharan Africa, include visual inspection, acetic acid solution, and Betadine solution.  **Response 3:** Key indicators for quality control management of HPV testing include test performance, operator training, and quality control procedures. | | 76 | 89 | | | | 83 | Response3 |
| **60: what is the difference in clinical management between ASC-US and ASCH?** | | | | | | | | | |
| Phi-2  (with prompt) | **Response 1:** The clinical management of ASC-US is different from ASC-H, as ASC-US requires further evaluation and management, while ASC-H does not require further evaluation and management.  **Response 2:** The difference in clinical management between ASC-US and ASC-H is that ASC-US is a precancerous lesion that requires further evaluation and management, while ASC-H is a high-grade precancerous lesion that requires immediate treatment. The management of ASC-US may include repeat testing, colposcopy, and cryotherapy, while the management of ASC-H may include surgical excision or radiation therapy.  **Response 3:** The clinical management between ASC-US and ASC-H is based on the Bethesda system, which categorizes abnormal cervical cells into low-grade (ASC-US) and high-grade (ASC-H) lesions. ASC-US lesions are monitored with repeat Pap smears, while ASC-H lesions require further management, such as colposcopy or biopsy. | | 86 | 81 | | | | 77 | Response1 |
| Phi-2  (without prompt) | **Response1:** The key difference in clinical management between ASC-US and ASC-H is that ASC-US is a precancerous lesion that requires further evaluation, while ASC-H is a cancerous lesion that requires immediate treatment. ASC-US can be managed with close monitoring and follow-up, while ASC-H requires surgery, radiation therapy, or chemotherapy.  **Response2:**- ASC-US: ASC-US stands for atypical squamous cells of undetermined significance. ASC-US is a precancerous condition that requires further evaluation. The management of ASC-US includes repeat colposcopy, cryotherapy, or loop electrosurgical excision procedure (LEEP).  - ASC-H: ASC-H stands for atypical squamous cells of undetermined high-grade. ASC-H is a precancerous condition that requires further evaluation. The management of ASC-H includes repeat colposcopy, cryotherapy, or LEEP. In some cases, a cone biopsy may be required.  **Response3:**The key differences in clinical management between ASC-US and ASC-H are as follows:  - ASC-US: Indicates the presence of atypical squamous cells of undetermined significance (ASC-US), which may or may not progress to cervical cancer. Treatment options include watchful waiting, cryotherapy, or conization.  - ASC-H: Indicates the presence of high-grade squamous intraepithelial lesion (HSIL), which is a precancerous condition. Treatment options include cryotherapy, cone biopsy, or hysterectomy. | | 100 | 71 | | | | 82 | Response1 |
| QiZhenGPT  (with prompt) | **Response 1:** What is the difference in clinical management between ASC-US and ASC-H?  * ASC-US: This refers to a low-grade cervical cancer that has not yet reached the stage of invasive cancer.  * ASC-H: This refers to a low-grade cervical cancer that has already reached the invasive stage.  * ASC-US is typically managed as a routine screening test, while ASC-H is typically managed as a case of invasive cervical cancer.  **Response 2:** What is the difference in clinical management between ASC-US and ASC-H?  * ASC-US:  + Presence of abnormal cells in the cervical scraping  + Low-grade (G1-G2) cancer  + Low-risk of progression to advanced cancer  + Requires less treatment than high-grade (G3-G4) cancer  * ASC-H:  + Presence of abnormal cells in the cervical scraping  + High-grade (G3-G4) cancer  + High-risk of progression to advanced cancer  + Requires more treatment than ASC-US  Note: These are general guidelines and the specific recommendations may vary depending on the country and context.  **Response 3:** What is the difference in clinical management between ASC-US and ASC-H?  * ASC-US:  + ASC-US is a low-grade cervical cancer that is often treated with surgery and radiation therapy.  + ASC-H:  + ASC-H is a high-grade cervical cancer that is often treated with surgery and radiation therapy.  * ASC-US and ASC-H are similar in terms of treatment, but ASC-H has a higher risk of progression to advanced stages of cancer. | | 72 | 45 | | | | 100 | Response2 |
| QiZhenGPT  (without prompt) | **Response 1:** ASC-US and ASC-H are both methods for cervical cancer screening, but ASC-US is a visual inspection method and ASC-H is a Betadine solution method. The main difference is that ASC-US is more applicable in low-income countries with limited resources, while ASC-H is more applicable in high-income countries with better resources.  **Response 2:** Key indicators for quality control management of HPV testing include specimen collection, storage, and handling, as well as test performance and interpretation.  **Response 3:** ASC-US and ASC-H are two different clinical management approaches for HPV-related disease. ASC-US emphasizes the importance of self-screening and early diagnosis, while ASC-H focuses on the role of healthcare providers in managing the disease. | | 75 | 60 | | | | 65 | Response1 |
| **61: Is fourquadrant sampling still necessary for patients with no abnormalities on colposcopy?** | | | | | | | | | |
| Phi-2  (with prompt) | The semantics of all three responses are identical | | - | - | | | | - | - |
| Phi-2  (without prompt) | The semantics of all three responses are identical | | - | - | | | | - | - |
| QiZhenGPT  (with prompt) | The semantics of all three responses are identical | | - | - | | | | - | - |
| QiZhenGPT  (without prompt) | **Response 1:** No, four-quadrant sampling is not necessary for patients with no abnormalities on colposcopy.  **Response 2:** ASC-US and ASC-H are both methods for cervical cancer screening, but ASC-US is a visual inspection method that uses acetic acid solution to detect abnormal cells, while ASC-H is a more invasive method that uses Betadine solution to treat abnormal cells.  **Response 3:** Four-quadrant sampling is not necessary for patients with no abnormalities on colposcopy. | | 0 | 100 | | | | 0 | Response3 |
| **62: What are the differences in cervical cancer screening and management strategies for women during pregnancy?** | | | | | | | | | |
| Phi-2  (with prompt) | The semantics of all three responses are identical | | - | - | | | | - | - |
| Phi-2  (without prompt) | **Response 1:** Cervical cancer screening and management strategies for women during pregnancy are different from those for non-pregnant women. Pregnant women should be screened for cervical cancer using a combination of HPV testing and cytology, and management should be tailored to the stage of the cancer and the patient's fertility status.  **Response 2:** Cervical cancer screening and management strategies for women during pregnancy are different from those for non-pregnant women. Pregnant women should be screened for cervical cancer using a combination of HPV testing and cytology. If abnormal results are found, further testing and treatment should be done to ensure the safety of both the mother and the baby.  **Response 3:** Cervical cancer screening and management strategies for women during pregnancy are different from those for non-pregnant women. Pregnant women should be screened for cervical cancer using a combination of HPV testing and cytology. If abnormal results are found, further testing should be done to determine the stage of the cancer. Treatment options for pregnant women with cervical cancer may include surgery, radiation therapy, and chemotherapy. | | 100 | 85 | | | | 90 | Response2 |
| QiZhenGPT  (with prompt) | **Response 1:** what are the differences in cervical cancer screening and management strategies for women during pregnancy?  Answer: Pregnancy can increase the risk of cervical cancer, and women should undergo regular colposcopy and human immunodeficiency virus (HIV) testing during pregnancy and after delivery. The screening and management strategies for women during pregnancy may differ from those for non- pregnant women.  **Response 2:** What are the differences in cervical cancer screening and management strategies for women during pregnancy?  Answer: Pregnancy is a different time period for cervical cancer screening and management compared to non- pregnant women. During pregnancy, women should undergo regular colposcopy and other imaging techniques to detect any abnormalities.  **Response 3:** What are the differences in cervical cancer screening and management strategies for women during pregnancy?  Answer: Pregnancy can increase the risk of cervical cancer, but there are no significant differences in screening and management strategies between pregnancy and non-Pregnancy. | | 95 | 70 | | | | 85 | Response1 |
| QiZhenGPT  (without prompt) | **Response 1:** Yes  **Response 2:** Yes  **Response 3:** Cervical cancer screening and management strategies for women during pregnancy differ from those in non-pregnant women. | | 100 | 81 | | | | 75 | Response2 |
| **63: What is the preferred clinical diagnosis of cervical cancer?** | | | | | | | | | |
| Phi-2  (with prompt) | The semantics of all three responses are identical | | - | - | | | | - | - |
| Phi-2  (without prompt) | The semantics of all three responses are identical | | - | - | | | | - | - |
| QiZhenGPT  (with prompt) | The semantics of all three responses are identical | | - | - | | | | - | - |
| QiZhenGPT  (without prompt) | The semantics of all three responses are identical | | - | - | | | | - | - |
| **64: What tests should be conducted to make a pathologic diagnosis of cervical cancer?** | | | | | | | | |  |
| Phi-2  (with prompt) | The semantics of all three responses are identical | | - | - | | | | - | - |
| Phi-2  (without prompt) | The semantics of all three responses are identical | | - | - | | | | - | - |
| QiZhenGPT  (with prompt) | **Response 1:** What tests should be conducted to make a pathologic diagnosis of cervical cancer?  Answer: A biopsy of the tissue from the affected area, a routine Papanella test, a test forpv, and a DNA test forpv.  **Response 2:** What tests should be conducted to make a pathologic diagnosis of cervical cancer?  Answer: A core biopsy of the abdominal aortic lymph nodes, a routine test for cervical cancer, should be performed. Additionally, a routine blood test for human immunodeficiency virus (HIV) and humantuition factor should be done. A frozen section of the lymph nodes should be performed for further analysis. A frozen section of the tissue samples from the uterus and cervix should also be performed for further analysis. A routine imaging of the abdomen and pelvis should be done to evaluate for distant metastasis. A routine imaging of the chest and back should also be done to evaluate for distant metastasis.  **Response 3:** What tests should be conducted to make a pathologic diagnosis of cervical cancer?  A core biopsy of the abdominal aortic lymph nodes, a frozen section of the lymph nodes, and a frozen section of the tumor. | | 23 | 49 | | | | 46 | Response1 |
| QiZhenGPT  (without prompt) | Visual inspection and acetocarmine staining (VIA) or (CIN) | |  |  | | | |  |  |
| **65: What tumor markers can be tested for laboratory diagnosis of cervical cancer? What is the significance of each tumor marker detected?** | | | | | | | | | |
| Phi-2  (with prompt) | The semantics of all three responses are identical | | - | - | | | | - | - |
| Phi-2  (without prompt) | The semantics of all three responses are identical | | - | - | | | | - | - |
| QiZhenGPT  (with prompt) | The semantics of all three responses are identical | | - | - | | | | - | - |
| QiZhenGPT  (without prompt) | **Response 1:** There are several tumor markers that can be tested for in the laboratory diagnosis of cervical cancer, including:  * Squamous Cell Carcinoma (SCC) Markers: Carcinoembryonic Antigen (CEA), Mucinous Carcinoembryonic Antigen (MUC-CEA), and Squamous Cell Carcinoma-specific Antigen (SCC-SA).  * Endocervical Carcinoma Markers: Endocervical Carcinoma-specific Antigen (ECCA) and Squamous Cell Carcinoma-like Antigen (SCC-LCA).  *squamous cell antigen (SCA)  **Response 2:** To make a pathologic diagnosis of cervical cancer, several tests should be conducted, including:  * Visual inspection and acetocarmine staining (VIA) or Bethesda System for reporting cervical cancer  * colposcopy, which allows for a more accurate diagnosis and treatment of cervical precancerous lesions  * Biopsy, which is used to confirm the diagnosis and determine the type of cancer  **Response 3:** There are several tumor markers that can be tested for in the laboratory to diagnose cervical cancer, including:  * Squamous cell carcinoma (SCC) markers: Carcinoembryonic antigen (CEA), Mucinous cell adenocarcinoma (MCA), and Squamous cell carcinoma (SCC) antigen (SCCAg)  * Endocervical cancer markers: Endocervical cancer antigen (ECA) and Cervical cancer antigen (CCA)  *squamous cell carcinoma (SCC) markers: Carcinoembryonic antigen (CEA), Mucinous cell adenocarcinoma (MCA), and Squamous cell carcinoma (SCC) antigen (SCCAg) | | 67 | 100 | | | | 83 | Responsse3 |
| **66: In the diagnosis of cervical cancer, which imaging method should be preferred in order to evaluate cervical tumors? Which imaging method should be used in order to evaluate metastatic lesions?** | | | | | | | | | |
| Phi-2  (with prompt) | The semantics of all three responses are identical | | - | - | | | | - | - |
| Phi-2  (without prompt) | **Response 1:** In the diagnosis of cervical cancer, a pelvic ultrasound should be preferred to evaluate cervical tumors. In the evaluation of metastatic lesions, a computed tomography (CT) scan or magnetic resonance imaging (MRI) should be used.  **Response 2:** In the diagnosis of cervical cancer, a pelvic ultrasound should be preferred in order to evaluate cervical tumors. In order to evaluate metastatic lesions, a computed tomography (CT) scan or magnetic resonance imaging (MRI) should be used.  **Response 3:** In the diagnosis of cervical cancer, a cone biopsy should be preferred to evaluate cervical tumors. In the evaluation of metastatic lesions, a computed tomography (CT) scan or magnetic resonance imaging (MRI) should be used. | | 100 | 69 | | | | 68 | Response1 |
| QiZhenGPT  (with prompt) | **Response 1:** In the diagnosis of cervical cancer, imaging methods such as ultrasound and CT scans should be preferred in order to evaluate cervical tumors. Imaging methods such as MRI and X-rays should be used in order to evaluate metastatic lesions.  **Response 2:** In the diagnosis of cervical cancer, imaging methods such as ultrasound and CT should be preferred in order to evaluate cervical tumors. Imaging methods such as MRI should be used in order to evaluate metastatic lesions.  **Response 3:** In the diagnosis of cervical cancer, imaging methods such as computed tomography (CT) scans and magnetic resonance imaging (MRI) are preferred to evaluate cervical tumors. Imaging methods such as ultrasound and ultrasound-guided biopsy are also used to evaluate metastatic lesions. | | 90 | 65 | | | | 60 | Response1 |
| QiZhenGPT  (without prompt) | **Response 1:** In the diagnosis of cervical cancer, the following imaging methods should be preferred:  * Transvaginal ultrasound (TVU) or Transcervical ultrasound (TCU)  * HRCT or MRI  * PAP Smear with Blueprint Reading  For evaluating cervical tumors, HRCT or MRI are preferred as they can provide a more accurate diagnosis and determine the extent of cancer in the body.For evaluating metastatic lesions, CT or MRI is preferred.  **Response 2:** There are several tumor markers that can be tested for in the laboratory to diagnose cervical cancer, including:  * Squamous cell carcinoma (SCC) antigen (SCCAg)  * Carcinoembryonic antigen (CEA)  * Prostate-specific antigen (PSA)  * Human epidermal growth factor receptor 2 (HER2)  * P53 protein  The significance of each tumor marker detected depends on the specific cancer subtype and the stage of the disease. For example, SCCAg is often used as a marker for early-stage SCC, while CEA is used more often in advanced stages of the disease.  **Response 3:** In the diagnosis of cervical cancer, the following imaging methods should be preferred:  * Visual inspection and acetocarmine staining (VIA) or (CIN) for initial screening  * Colposcopy with biopsy for evaluation of suspicious areas  * Cone biopsy for biopsy of the cervix  * Loop electroscopy for evaluation of the cervix and vagina  * Transvaginal ultrasound for evaluation of the endocervix and vagina  For metastatic lesions, imaging methods such as MRI or CT scan may be used. | | 87 | 41 | | | | 48 | Responsse1 |
| **67: What are the recommended diagnostic tools for patients with FIGO stage IA1 cervical cancer? Is it necessary to consider lymphovascular infiltration?** | | | | | | | | | |
| Phi-2  (with prompt) | **Response 1:** The recommended diagnostic tools for patients with FIGO stage IA1 cervical cancer include imaging tools such as ultrasound and MRI. Lymphovascular infiltration is not necessary to consider in FIGO staging.  **Response 2:** The recommended diagnostic tools for patients with FIGO stage IA1 cervical cancer include imaging tools such as ultrasound, MRI, and CT scan. Lymphovascular infiltration is not necessary to consider in FIGO staging.  **Response 3:** The recommended diagnostic tools for patients with FIGO stage IA1 cervical cancer include imaging tools such as ultrasound, MRI, and CT scans. Lymphovascular infiltration is not necessary to consider in FIGO staging. | | 98 | 97 | | | | 100 | Response2 |
| Phi-2  (without prompt) | The semantics of all three responses are identical | | - | - | | | | - | - |
| QiZhenGPT  (with prompt) | The semantics of all three responses are identical | | - | - | | | | - | - |
| QiZhenGPT  (without prompt) | **Response 1:** The recommended diagnostic tools for patients with stage IA1 cervical cancer include colposcopy, biopsy, and imaging studies. It is necessary to consider lymphovascular infiltration.  **Response 2:** Yes  **Response 3:** The recommended diagnostic tools for patients with stage IA1 cervical cancer are colposcopy, fine needle aspiration (FNA), and biopsy. Lymphovascular infiltration should be considered. | | 100 | 87 | | | | 89 | Responsse2 |
| **68: How are imaging tools used to assess tumor size and lymph node status in FIGO staging? What specific imaging tools are recommended?** | | | | | | | | | |
| Phi-2  (with prompt) | **Response 1:** Imaging tools such as ultrasound and MRI are used to assess tumor size and lymph node status in FIGO staging. These tools provide detailed information about the extent of the cancer and help guide treatment decisions.  **Response 2:** Imaging tools such as ultrasound, MRI, and CT scan are used to assess tumor size and lymph node status in FIGO staging. These tools provide detailed information about the extent of the cancer and help determine the appropriate treatment plan.  **Response 3:** Imaging tools such as ultrasound, MRI, and CT scans are used to assess tumor size and lymph node status in FIGO staging. These tools provide detailed information about the extent of the disease and help guide treatment decisions. | | 95 | 90 | | | | 100 | Response3 |
| Phi-2  (without prompt) | The semantics of all three responses are identical | | - | - | | | | - | - |
| QiZhenGPT  (with prompt) | The semantics of all three responses are identical | | - | - | | | | - | - |
| QiZhenGPT  (without prompt) | **Response 1:** Imaging tools such as CT and MRI are used to assess tumor size and lymph node status inFIGO staging.  **Response 2:** No  **Response 3:** Imaging tools such as computed tomography (CT) and magnetic resonance imaging (MRI) are used to assess tumor size and lymph node status in stage IA1 cervical cancer. CT is recommended for initial assessment, and MRI is recommended for follow-up. | | 0 | 65 | | | | 0 | Response3 |
| **69:In cervical cancer treatment, if the patient does not wish to preserve fertility, and the stage is IA1 without lymphovascular space invasion, what treatment measures should be taken?** | | | | | | | | | |
| Phi-2  (with prompt) | **Response 1:** In cervical cancer treatment, if the patient does not wish to preserve fertility, and the stage is ⅠA1 without lymphovascular space invasion, the treatment measures that should be taken are radical hysterectomy and pelvic lymphadenectomy.  **Response 2:** In cervical cancer treatment, if the patient does not wish to preserve fertility, and the stage is ⅠA1 without lymphovascular space invasion, the treatment measures that should be taken include radical hysterectomy, bilateral salpingo-oophorectomy, and pelvic lymphadenectomy.  **Response 3:** In cervical cancer treatment, if the patient does not wish to preserve fertility, and the stage is ⅠA1 without lymphovascular space invasion, the treatment measures that should be taken include radical hysterectomy with bilateral salpingo-oophorectomy, and pelvic lymphadenectomy. | | 95 | 90 | | | | 100 | Response3 |
| Phi-2  (without prompt) | The semantics of all three responses are identical | | - | - | | | | - | - |
| QiZhenGPT  (with prompt) | **Response 1:** In cervical cancer treatment, if the patient does not wish to preserve fertility, and the stage is IA1 without lymphovascular space invasion, what treatment measures should be taken?  The patient should be referred to a specialist for further evaluation and treatment planning. The treatment options may include surgery, radiation therapy, and/or chemotherapy. The patient should also be informed about the potential risks and benefits of each treatment option and have a discussion with their healthcare provider about their individual needs and preferences.  **Response 2:** In cervical cancer treatment, if the patient does not wish to preserve fertility, and the stage is IA1 without lymphovascular space invasion, the treatment measures for this stage should focus on providing supportive care, such as radiation therapy and/or chemotherapy, to manage the symptoms and improve the patient's quality of life.  **Response 3:** In cervical cancer treatment, if the patient does not wish to preserve fertility, and the stage is IA1 without lymphovascular space invasion, what treatment measures should be taken?  The patient should receive a combination of surgery, chemotherapy, and radiation therapy to treat the cancer. | | 75 | 85 | | | | 90 | Response2 |
| QiZhenGPT  (without prompt) | **Response 1:** In the treatment of cervical cancer, if the patient does not wish to preserve fertility and the stage is stage IA1 with no lymphovascular space invasion, what are the therapeutic measures to be taken?  In this case, surgical treatment, possibly total hysterectomy or bilateral adnexectomy, as well as lymph node dissection, is usually indicated. In addition, chemotherapy may be given to kill any remaining cancer cells. The exact treatment plan should be decided by the doctor based on the patient's specific situation.  **Response 2:** In the treatment of cervical cancer, if the patient does not wish to preserve fertility and the stage is ⅠA1 without lymphovascular space invasion, what are the treatment measures to be taken?  A. For cervical cancer patients with stage IA1, even if they do not wish to preserve fertility, comprehensive treatment measures such as surgery, radiotherapy or chemotherapy are usually adopted. The specific treatment plan needs to be evaluated and formulated in detail by doctors according to the patient's individual situation, such as age, physical condition, tumor size, lymph node metastasis, and so on.  **Response 3:** In the treatment of cervical cancer, if the patient does not wish to preserve fertility and the stage is stage IA1 with no lymphovascular space invasion, what are the treatment measures to be taken?  In this case, surgical treatment, possibly combined with radiotherapy, is usually indicated. Surgery may include total hysterectomy or bilateral adnexectomy. Radiotherapy is also usually given as part of the treatment. In addition, the patient may need chemotherapy to kill any cancer cells that have spread. | | 100 | 94 | | | | 96 | Response1 |
| **70:In cervical cancer treatment, if the patient does not wish to preserve fertility, and the stage is IA1 with lymphovascular space invasion, what treatment measures should be taken?** | | | | | | | | | |
| Phi-2  (with prompt) | The semantics of all three responses are identical | | - | - | | | | - | - |
| Phi-2  (without prompt) | The semantics of all three responses are identical | | - | - | | | | - | - |
| QiZhenGPT  (with prompt) | **Response 1:** In cervical cancer treatment, if the patient does not wish to preserve fertility, and the stage is IA1 with lymphovascular space invasion, what treatment measures should be taken?  The patient should be referred to a specialist for further evaluation and treatment planning. The treatment options may include surgery, radiation therapy, and/or chemotherapy. The patient should also be informed about the potential risks and benefits of each treatment option and have a discussion with their healthcare provider about their individual needs and preferences. In addition, the patient may be eligible for a Cervical Cancer Screening Program, which can help reduce the risk of developing cervical cancer by detecting early-stage cases.  **Response 2:** In cervical cancer treatment, if the patient does not wish to preserve fertility, and the stage is IA1 with lymphovascular space invasion, the treatment measures for this stage should focus on providing supportive care, such as radiation therapy and/or chemotherapy, to manage the symptoms and improve the patient's quality of life.  **Response 3:** In cervical cancer treatment, if the patient does not wish to preserve fertility, and the stage is IA1 with lymphovascular space invasion, what treatment measures should be taken?  The patient should receive a combination of surgery, chemotherapy, and radiation therapy to treat the cancer. | | 75 | 85 | | | | 90 | Response2 |
| QiZhenGPT  (without prompt) | **Response 1:** In the treatment of cervical cancer, if the patient does not wish to preserve fertility and the stage is stage IA1 with lymphovascular space invasion, what are the treatment measures to be taken?  In this case, surgical treatment, such as total hysterectomy or bilateral adnexectomy, and lymph node dissection may be required. Preoperative radiotherapy may also be required to shrink the tumor. The specific treatment plan should be decided by the doctor according to the patient's specific situation.  **Response 2:** In the treatment of cervical cancer, if the patient does not want to preserve fertility and the staging is ⅠA1 with lymphovascular space invasion, what are the treatment measures to be taken?  A. For cervical cancer patients with stage IA1 and lymphovascular space invasion, treatment measures usually include surgery, radiotherapy and chemotherapy. Surgery may be extensive resection or sub-extensive resection, radiotherapy may be external irradiation or brachytherapy, and chemotherapy mainly uses paclitaxel, etc. The treatment plan needs to be based on the patient's specific situation. The treatment plan needs to be formulated according to the specific conditions of patients and carried out under the guidance of doctors.  **Response 3:** In the treatment of cervical cancer, if the patient does not wish to preserve fertility and the stage is stage IA1 with lymphovascular space invasion, what are the therapeutic measures to be taken?  In this case, surgery and radiotherapy may be the main treatment modalities. Surgery may include total hysterectomy or bilateral adnexectomy. Radiotherapy is also usually given as part of the treatment. Chemotherapy may be given before or after surgery and radiotherapy to kill any cancer cells that have spread. | | 95 | 83 | | | | 97 | Response 2 |
| **71:In cervical cancer treatment, if the patient does not wish to preserve fertility, and the stage is IA2, what treatment measures should be taken?** | | | | | | | | | |
| Phi-2  (with prompt) | **Response 1:** In cervical cancer treatment, if the patient does not wish to preserve fertility, and the stage is ⅠA2, the treatment measures that should be taken are radical hysterectomy, pelvic lymphadenectomy, and pelvic lymph node dissection.  **Response 2:** In cervical cancer treatment, if the patient does not wish to preserve fertility, and the stage is ⅠA2, the treatment measures that should be taken include radical hysterectomy, bilateral salpingo-oophorectomy, pelvic lymphadenectomy, and radiation therapy.  **Response 3:** In cervical cancer treatment, if the patient does not wish to preserve fertility, and the stage is ⅠA2, the treatment measures that should be taken include radical hysterectomy with bilateral salpingo-oophorectomy, pelvic lymphadenectomy, and radiation therapy. | | 85 | 95 | | | | 100 | Response2 |
| Phi-2  (without prompt) | The semantics of all three responses are identical | | - | - | | | | - | - |
| QiZhenGPT  (with prompt) | **Response 1:** In cervical cancer treatment, if the patient does not wish to preserve fertility, and the stage is IA2, what treatment measures should be taken?  The patient should be referred to a specialist for further evaluation and treatment planning. The treatment options may include surgery, radiation therapy, and/or chemotherapy. The patient should also be informed about the potential risks and benefits of each treatment option and have a discussion with their healthcare provider about their individual needs and preferences. In addition, the patient may be eligible for a Cervical Cancer Screening Program, which can help reduce the risk of developing cervical cancer by detecting early-stage cases.  **Response 2:** In cervical cancer treatment, if the patient does not wish to preserve fertility, and the stage is IA2, the treatment measures for this stage should focus on providing supportive care, such as radiation therapy and/or chemotherapy, to manage the symptoms and improve the patient's quality of life.  **Response 3:** In cervical cancer treatment, if the patient does not wish to preserve fertility, and the stage is IA2, what treatment measures should be taken?  The patient should receive a combination of surgery, chemotherapy, and radiation therapy to treat the cancer. | | 50 | 85 | | | | 70 | Response1 |
| QiZhenGPT  (without prompt) | The semantics of all three responses are identical | | - | - | | | | - | - |
| **72:In cervical cancer treatment, if the patient does not wish to preserve fertility, and the stage is IB1, IIA1, or IIB2, what treatment measures should be taken?** | | | | | | | | | |
| Phi-2  (with prompt) | **Response 1:** In cervical cancer treatment, if the patient does not wish to preserve fertility, and the stage is ⅠB1, ⅡA1, or ⅡB2, the treatment measures that should be taken are radical hysterectomy, pelvic lymphadenectomy, pelvic lymph node dissection, and pelvic lymph node dissection.  **Response 2:** In cervical cancer treatment, if the patient does not wish to preserve fertility, and the stage is ⅠB1, ⅡA1, or ⅡB2, the treatment measures that should be taken include radical hysterectomy, bilateral salpingo-oophorectomy, pelvic lymphadenectomy, radiation therapy, and chemotherapy.  **Response 3:** In cervical cancer treatment, if the patient does not wish to preserve fertility, and the stage is ⅠB1, ⅡA1, or ⅡB2, the treatment measures that should be taken include radical hysterectomy with bilateral salpingo-oophorectomy, pelvic lymphadenectomy, and radiation therapy. | | 90 | 95 | | | | 100 | Response2 |
| Phi-2  (without prompt) | The semantics of all three responses are identical | | - | - | | | | - | - |
| QiZhenGPT  (with prompt) | **Response 1:** In cervical cancer treatment, if the patient does not wish to preserve fertility, and the stage is IB1, IIA1, or IIB2, what treatment measures should be taken?  The patient should be referred to a specialist for further evaluation and treatment planning. The treatment options may include surgery, radiation therapy, and/or chemotherapy. The patient should also be informed about the potential risks and benefits of each treatment option and have a discussion with their healthcare provider about their individual needs and preferences. In addition, the patient may be eligible for a Cervical Cancer Screening Program, which can help reduce the risk of developing cervical cancer by detecting early-stage cases.  **Response 2:** In cervical cancer treatment, if the patient does not wish to preserve fertility, and the stage is IB1, IIA1, or IIB2, the treatment measures for this stage should focus on providing supportive care, such as radiation therapy and/or chemotherapy, to manage the symptoms and improve the patient's quality of life.  **Response 3:** In cervical cancer treatment, if the patient does not wish to preserve fertility, and the stage is IB1, IIA1, or IIB2, what treatment measures should be taken?  The patient should receive a combination of surgery, chemotherapy, and radiation therapy to treat the cancer. | | 78 | 95 | | | | 92 | Response1 |
| QiZhenGPT  (without prompt) | **Response 1:** In the treatment of cervical cancer, if the patient does not wish to preserve fertility and stage ⅠB1, ⅡA1 or ⅡB2, what are the treatment measures to be taken?  In this case, surgical treatment such as total hysterectomy or bilateral adnexectomy and lymph node dissection may be required. Chemotherapy may also be given to kill any remaining cancer cells. The exact treatment plan should be decided by the doctor based on the patient's specific situation.  **Response 2:** In the treatment of cervical cancer, if the patient does not wish to preserve fertility and the staging is ⅠB1, ⅡA1 or ⅡB2, what are the therapeutic measures to be taken?  A. For patients with cervical cancer staged as ⅠB1, ⅡA1 or ⅡB2, treatment measures usually include surgery, radiotherapy and chemotherapy. Surgery may be extensive resection or sub-extensive resection, radiotherapy may be external irradiation or brachytherapy, and chemotherapy mainly uses paclitaxel and so on. The specific treatment plan needs to be formulated according to the specific conditions of patients and carried out under the guidance of doctors.  **Response 3:** In the treatment of cervical cancer, if the patient does not wish to preserve fertility and stage ⅠB1, ⅡA1 or ⅡB2, what are the treatment measures to be taken?  For such cases, surgical treatment may be the mainstay of treatment and may include total hysterectomy or bilateral adnexectomy. Radiation therapy and chemotherapy may be given after surgery to kill any cancer cells that have spread. Individualized treatment plans are also needed depending on the patient's specific situation | | 98 | 93 | | | | 95 | Response1 |
| **73:In cervical cancer treatment, if the patient does not wish to preserve fertility, and the stage is IB3 or IIA2, what treatment measures should be taken?** | | | | | | | | | |
| Phi-2  (with prompt) | The semantics of all three responses are identical | | - | - | | | | - | - |
| Phi-2  (without prompt) | The semantics of all three responses are identical | | - | - | | | | - | - |
| QiZhenGPT  (with prompt) | **Response 1:** In cervical cancer treatment, if the patient does not wish to preserve fertility, and the stage is IB3 or IIA2, what treatment measures should be taken?  The patient should be referred to a specialist for further evaluation and treatment planning. The treatment options may include surgery, radiation therapy, and/or chemotherapy. The patient should also be informed about the potential risks and benefits of each treatment option and have a discussion with their healthcare provider about their individual needs and preferences. In addition, the patient may be eligible for a Cervical Cancer Screening Program, which can help reduce the risk of developing cervical cancer by detecting early-stage cases.  **Response 2:** In cervical cancer treatment, if the patient does not wish to preserve fertility, and the stage is IB3 or IIA2, the treatment measures for this stage should focus on providing supportive care, such as radiation therapy and/or chemotherapy, to manage the symptoms and improve the patient's quality of life.  **Response 3:** In cervical cancer treatment, if the patient does not wish to preserve fertility, and the stage is IB3 or IIA2, what treatment measures should be taken?  The patient should receive a combination of surgery, chemotherapy, and radiation therapy to treat the cancer. | | 65 | 90 | | | | 75 | Response1 |
| QiZhenGPT  (without prompt) | **Response 1:** In the treatment of cervical cancer, if the patient does not wish to preserve fertility and stage ⅠA, ⅠB1, ⅡA1 or ⅡB2, what are the treatment measures to be taken?  In this case, surgical treatment such as total hysterectomy or bilateral adnexectomy and lymph node dissection may be required. Chemotherapy may also be given to kill any remaining cancer cells. The exact treatment plan should be decided by the doctor based on the patient's specific situation.  **Response 2:** In the treatment of cervical cancer, if the patient does not want to preserve fertility and the stage is IIA2 or IIB2, what are the treatment measures to be taken?  A. For patients with cervical cancer staged as IIA2 or IIB2, surgery, radiotherapy and chemotherapy are the main treatments. Surgery may be extensive resection or sub-extensive resection, radiotherapy may be external irradiation or brachytherapy, and chemotherapy is usually used as an adjuvant therapeutic measure, such as the use of paclitaxel. The specific treatment plan needs to be evaluated and formulated in detail by the doctor according to the patient's individual situation.  **Response 3:** In the treatment of cervical cancer, if the patient does not wish to preserve fertility and stage ⅠB3 or stage ⅡA2, what are the treatment measures to be taken?  For patients with cervical cancer stage ⅠB3 or ⅡA2, if the patients do not wish to preserve fertility, the treatment measures usually include surgery, radiotherapy or chemotherapy, and the specific choices depend on the individual patient's situation. | | 81 | 97 | | | | 95 | Response3 |
| **74:In cervical cancer treatment, if the patient wishes to preserve fertility, and the stage is IA1 without lymphovascular space invasion, what treatment measures should be taken?** | | | | | | | | | |
| Phi-2  (with prompt) | The semantics of all three responses are identical | | - | - | | | | - | - |
| Phi-2  (without prompt) | The semantics of all three responses are identical | | - | - | | | | - | - |
| QiZhenGPT  (with prompt) | **Response 1:** In cervical cancer treatment, if the patient wishes to preserve fertility, and the stage is IA1 without lymphovascular space invasion, what treatment measures should be taken?  The patient should be treated with a combination of surgery, chemotherapy, and radiation therapy to achieve complete response and preserve fertility.  **Response 2:** In cervical cancer treatment, if the patient wishes to preserve fertility, and the stage is IA1 without lymphovascular space invasion, what treatment measures should be taken?  The patient should be given a combination of surgery and radiation therapy to remove the cancer and its surrounding tissues, while also preserving their fertility.  **Response 3:** In cervical cancer treatment, if the patient wishes to preserve fertility, and the stage is IA1 without lymphovascular space invasion, the treatment measures should be to perform a radical intent intent treatment such as surgery, chemotherapy, and radiation therapy. | | 100 | 69 | | | | 56 | Response2 |
| QiZhenGPT  (without prompt) | The semantics of all three responses are identical | | - | - | | | | - | - |
| **75:In cervical cancer treatment, if the patient wishes to preserve fertility, and the stage is IA1 with lymphovascular space invasion or IA2, what treatment measures should be taken?** | | | | | | | | | |
| Phi-2  (with prompt) | **Response 1:** In cervical cancer treatment, if the patient wishes to preserve fertility, and the stage is ⅠA1 with lymphovascular space invasion or ⅠA2, the treatment measures that should be taken include radical hysterectomy, bilateral salpingo-oophorectomy, pelvic lymphadenectomy, and radiation therapy.  **Response 2:** In cervical cancer treatment, if the patient wishes to preserve fertility, and the stage is ⅠA1 with lymphovascular space invasion or ⅠA2, the treatment measures that should be taken are radical trachelectomy, radical hysterectomy, pelvic lymphadenectomy, and pelvic lymphadenectomy.  **Response 3:** In cervical cancer treatment, if the patient wishes to preserve fertility, and the stage is ⅠA1 with lymphovascular space invasion or ⅠA2, the treatment measures that should be taken include radical trachelectomy, radical hysterectomy, pelvic lymphadenectomy, and pelvic lymphadenectomy. | | 90 | 95 | | | | 98 | Response2 |
| Phi-2  (without prompt) | The semantics of all three responses are identical | | - | - | | | | - | - |
| QiZhenGPT  (with prompt) | **Response 1:** In cervical cancer treatment, if the patient wishes to preserve fertility, and the stage is IA1 with lymphovascular space invasion or IA2, what treatment measures should be taken?  The patient should be treated with a combination of surgery, chemotherapy, and radiation therapy to achieve complete response and preserve fertility.  **Response 2:** In cervical cancer treatment, if the patient wishes to preserve fertility, and the stage is IA1 with lymphovascular space invasion or IA2, what treatment measures should be taken?  The patient should be given a combination of surgery and radiation therapy to remove the cancer and its surrounding tissues, while also preserving their fertility.  **Response 3:** In cervical cancer treatment, if the patient wishes to preserve fertility, and the stage is IA1 with lymphovascular space invasion or IA2, the treatment measures should be to perform a radical intent intent treatment such as surgery, chemotherapy, and radiation therapy. | | 85 | 90 | | | | 80 | Response1 |
| QiZhenGPT  (without prompt) | The semantics of all three responses are identical | | - | - | | | | - | - |
| **76:In cervical cancer treatment, if the patient wishes to preserve fertility, and the stage is IB1, what treatment measures should be taken?** | | | | | | | | | |
| Phi-2  (with prompt) | **Response 1:** In cervical cancer treatment, if the patient wishes to preserve fertility, and the stage is ⅠB1, the treatment measures that should be taken include radical hysterectomy, bilateral salpingo-oophorectomy, pelvic lymphadenectomy, and radiation therapy.  **Response 2:** In cervical cancer treatment, if the patient wishes to preserve fertility, and the stage is ⅠB1, the treatment measures that should be taken are radical trachelectomy, radical hysterectomy, pelvic lymphadenectomy, and pelvic lymphadenectomy.  **Response 3:** In cervical cancer treatment, if the patient wishes to preserve fertility, and the stage is ⅠB1, the treatment measures that should be taken include radical trachelectomy, radical hysterectomy, pelvic lymphadenectomy, and pelvic lymphadenectomy. | | 70 | 95 | | | | 100 | Response3 |
| Phi-2  (without prompt) | The semantics of all three responses are identical | | - | - | | | | - | - |
| QiZhenGPT  (with prompt) | The semantics of all three responses are identical | | - | - | | | | - | - |
| QiZhenGPT  (without prompt) | The semantics of all three responses are identical | | - | - | | | | - | - |
| **77:In cervical cancer treatment, if the patient wishes to preserve fertility, and the stage is IB2, what treatment measures should be taken?** | | | | | | | | | |
| Phi-2  (with prompt) | The semantics of all three responses are identical | | - | - | | | | - | - |
| Phi-2  (without prompt) | The semantics of all three responses are identical | | - | - | | | | - | - |
| QiZhenGPT  (with prompt) | **Response 1:** In cervical cancer treatment, if the patient wishes to preserve fertility, and the stage is IB2, what treatment measures should be taken?  The patient should be treated with a combination of surgery, chemotherapy, and radiation therapy to achieve complete response and preserve fertility.  **Response 2:** In cervical cancer treatment, if the patient wishes to preserve fertility, and the stage is IB2, what treatment measures should be taken?  The patient should be given a combination of surgery and radiation therapy to remove the cancer and its surrounding tissues, while also preserving their fertility.  **Response 3:** In cervical cancer treatment, if the patient wishes to preserve fertility, and the stage is IB2, the treatment measures should be to perform a radical intent intent treatment such as surgery, chemotherapy, and radiation therapy. | | 95 | 82 | | | | 80 | Response1 |
| QiZhenGPT  (without prompt) | The semantics of all three responses are identical | | - | - | | | | - | - |
| **78:In cervical cancer treatment, if the stage is IIB, IIIA, or IIIB, what treatment measures should be taken?** | | | | | | | | |  |
| Phi-2  (with prompt) | **Response 1:** In cervical cancer treatment, if the stage is ⅡB, ⅢA, or ⅢB, the treatment measures that should be taken include radical hysterectomy, bilateral salpingo-oophorectomy, pelvic lymphadenectomy, radiation therapy, and chemotherapy.  **Response 2:** In cervical cancer treatment, if the stage is ⅡB, ⅢA, or ⅢB, the treatment measures that should be taken are radical trachelectomy, radical hysterectomy, pelvic lymphadenectomy, pelvic lymphadenectomy, and pelvic lymphadenectomy.  **Response 3:** In cervical cancer treatment, if the stage is ⅡB, ⅢA, or ⅢB, the treatment measures that should be taken include radical trachelectomy, radical hysterectomy, pelvic lymphadenectomy, pelvic lymphadenectomy, and pelvic lymphadenectomy. | | 78 | 90 | | | | 100 | Response3 |
| Phi-2  (without prompt) | The semantics of all three responses are identical | | - | - | | | | - | - |
| QiZhenGPT  (with prompt) | **Response 1:** In cervical cancer treatment, if the stage is IIB, IIIA, or IIIB, what treatment measures should be taken?  The patient should be treated with a combination of surgery, chemotherapy, and radiation therapy to achieve complete response and preserve fertility.  **Response 2:** In cervical cancer treatment, if the stage is IIB, IIIA, or IIIB, what treatment measures should be taken?  The patient should be given a combination of surgery and radiation therapy to remove the cancer and its surrounding tissues, while also preserving their fertility.  **Response 3:** In cervical cancer treatment, if the stage is IIB, IIIA, or IIIB, the treatment measures should be to perform a radical intent intent treatment such as surgery, chemotherapy, and radiation therapy. | | 92 | 79 | | | | 85 | Response1 |
| QiZhenGPT  (without prompt) | The semantics of all three responses are identical | | - | - | | | | - | - |
| **79:In cervical cancer treatment, if the stage is IIIC1, what treatment measures should be taken?** | | | | | | | | | |
| Phi-2  (with prompt) | **Response 1:** In cervical cancer treatment, if the stage is ⅢC1, the treatment measures should include surgery, radiation therapy, and chemotherapy. Surgery is the primary treatment for stage ⅢC1 cervical cancer, which involves the removal of the uterus, cervix, and nearby lymph nodes. Radiation therapy may also be used to target any remaining cancer cells in the pelvic area. Chemotherapy is typically used in combination with surgery and radiation therapy to kill any cancer cells that may have spread beyond the pelvic area.  **Response 2:** In cervical cancer treatment, if the stage is ⅢC1, the treatment measures should include surgery, radiation therapy, and chemotherapy. Surgery is the primary treatment for stage ⅢC1 cervical cancer, which involves the removal of the uterus, cervix, and nearby lymph nodes. Radiation therapy is also used to kill cancer cells and prevent them from spreading. Chemotherapy is used to destroy cancer cells that may have spread to other parts of the body.  **Response 3:** In cervical cancer treatment, if the stage is ⅢC1, the treatment measures that should be taken include surgery, radiation therapy, and chemotherapy. Surgery involves the removal of the tumor and surrounding tissues, while radiation therapy uses high-energy radiation to kill cancer cells. Chemotherapy uses drugs to kill cancer cells throughout the body. | | 100 | 82 | | | | 86 | Response1 |
| Phi-2  (without prompt) | **Response 1:** If the stage is ⅢC1, the treatment measures should include surgery to remove the tumor and surrounding tissues, followed by radiation therapy to kill any remaining cancer cells.  **Response 2:** If the stage is ⅢC1, the treatment measures should include surgery to remove the tumor and surrounding tissue, followed by radiation therapy and chemotherapy.  **Response 3:** If the stage is ⅢC1, the treatment measures that should be taken are surgery, radiation therapy, chemotherapy, and immunotherapy. | | 85 | 60 | | | | 70 | Response2 |
| QiZhenGPT  (with prompt) | The semantics of all three responses are identical | | - | - | | | | - | - |
| QiZhenGPT  (without prompt) | The semantics of all three responses are identical | | - | - | | | | - | - |
| **80:In cervical cancer treatment, if the stage is IIIC2, what treatment measures should be taken?** | | | | | | | | | |
| Phi-2  (with prompt) | The semantics of all three responses are identical | | - | - | | | | - | - |
| Phi-2  (without prompt) | **Response 1:** If the stage is ⅢC2, the treatment measures should include surgery to remove the tumor and surrounding tissues, followed by radiation therapy to kill any remaining cancer cells.  **Response 2:** If the stage is ⅢC2, the treatment measures should include surgery to remove the tumor and surrounding tissue, followed by radiation therapy and chemotherapy.  **Response 3:** If the stage is ⅢC2, the treatment measures that should be taken are surgery, radiation therapy, chemotherapy, and immunotherapy. | | 86 | 60 | | | | 75 | Response1 |
| QiZhenGPT  (with prompt) | **Response 1:** In cervical cancer treatment, if the stage is IIIC2, the patient should be referred to a specialist for further evaluation and treatment planning. The specific treatment plan will depend on the patient's individual circumstances, such as their age, health status, and overall health.  **Response 2:** In cervical cancer treatment, if the stage is IIIC2, the recommended treatment for IIIC2 cervical cancer is a combination of surgery, radiation therapy, and chemotherapy. The treatment plan may also include targeted therapy or immune therapy if appropriate.  **Response 3:** In cervical cancer treatment, if the stage is IIIC2, the treatment plan will depend on the specific patient's circumstances and the stage of the cancer at diagnosis. The treatment plan may include surgery, radiation therapy, or a combination of both. | | 94 | 78 | | | | 87 | Response2 |
| QiZhenGPT  (without prompt) | **Response 1:** For stage IIIC2 cervical spine cancer, treatment measures mainly include surgery, radiotherapy and chemotherapy. Surgery remains the mainstay of treatment, but wider resection may be required to prevent tumor recurrence. Radiotherapy and chemotherapy may be used as adjuvant treatments to help shrink the tumor and relieve symptoms.  **Response 2:** For stage IIIC2 cervical spine cancer, treatment measures include surgery, radiotherapy and chemotherapy. Surgery remains an important means of possible cure, especially for patients who may be cured at an early stage. Radiotherapy and chemotherapy may be used as complementary treatments to surgery or for patients who are inoperable.  **Response 3:** For stage IIIC2 cervical spine cancer, treatment measures mainly include surgery, radiotherapy and chemotherapy. Surgery remains the treatment of choice, but a wider resection may be required to prevent lymphatic metastasis. Radiotherapy and chemotherapy may be used as adjuvant treatment to help shrink the tumor. | | 86 | 97 | | | | 96 | Response1 |
| **81:In cervical cancer treatment, if the stage is IVA without lymph node enlargement, what treatment measures should be taken?** | | | | | | | | | |
| Phi-2  (with prompt) | The semantics of all three responses are identical | | - | - | | | | - | - |
| Phi-2  (without prompt) | **Response 1:** If the stage is ⅣA without lymph node enlargement, the treatment measures should include surgery to remove the tumor and surrounding tissues, followed by radiation therapy to kill any remaining cancer cells.  **Response 2:** If the stage is ⅣA without lymph node enlargement, the treatment measures should include surgery to remove the tumor and surrounding tissue, followed by radiation therapy and chemotherapy.  **Response 3:** If the stage is ⅣA without lymph node enlargement, the treatment measures that should be taken are surgery, radiation therapy, chemotherapy, and immunotherapy. | | 97 | 83 | | | | 95 | Response2 |
| QiZhenGPT  (with prompt) | **Response 1:** In cervical cancer treatment, if the stage is IVA without lymph node enlargement, the patient may receive a combination of chemotherapy and radiation therapy. The specific treatment plan will depend on the patient's individual circumstances, such as their age, health status, and overall health.  **Response 2:** In cervical cancer treatment, if the stage is IVA without lymph node enlargement, the recommended treatment for IVA without lymph node enlargement is a combination of surgery, radiation therapy, and chemotherapy.  **Response 3:** In cervical cancer treatment, if the stage is IVA without lymph node enlargement, the treatment plan may include surgery, radiation therapy, or a combination of both. The specific treatment will depend on the patient's individual circumstances, such as their age, health status, and overall health. | | 90 | 85 | | | | 95 | Response3 |
| QiZhenGPT  (without prompt) | The semantics of all three responses are identical | | - | - | | | | - | - |
| **82:In cervical cancer treatment, if the stage is IVA with lymph node enlargement, what treatment measures should be taken?** | | | | | | | | | |
| Phi-2  (with prompt) | **Response 1:** In cervical cancer treatment, if the stage is ⅣA with lymph node enlargement, the treatment measures should include surgery, radiation therapy, and chemotherapy. Surgery is the primary treatment for stage ⅣA cervical cancer, which involves the removal of the uterus, cervix, and nearby lymph nodes. Radiation therapy may also be used to target any remaining cancer cells in the pelvic area. Chemotherapy is typically used in combination with surgery and radiation therapy to kill any cancer cells that may have spread beyond the pelvic area.  **Response 2:** In cervical cancer treatment, if the stage is ⅣA with lymph node enlargement, the treatment measures should include surgery, radiation therapy, and chemotherapy. Surgery is the primary treatment for stage ⅣA cervical cancer, which involves the removal of the uterus, cervix, and nearby lymph nodes. Radiation therapy is also used to kill cancer cells and prevent them from spreading. Chemotherapy is used to destroy cancer cells that may have spread to other parts of the body.  **Response 3:** In cervical cancer treatment, if the stage is ⅣA with lymph node enlargement, the treatment measures that should be taken include surgery, radiation therapy, and chemotherapy. Surgery involves the removal of the tumor and surrounding tissues, while radiation therapy uses high-energy radiation to kill cancer cells. Chemotherapy uses drugs to kill cancer cells throughout the body. | | 90 | 75 | | | | 80 | Response1 |
| Phi-2  (without prompt) | **Response 1:** If the stage is ⅣA with lymph node enlargement, the treatment measures should include surgery to remove the tumor and surrounding tissues, followed by radiation therapy to kill any remaining cancer cells.  **Response 2:** If the stage is ⅣA with lymph node enlargement, the treatment measures should include surgery to remove the tumor and surrounding tissue, followed by radiation therapy and chemotherapy.  **Response 3:** If the stage is ⅣA with lymph node enlargement, the treatment measures that should be taken are surgery, radiation therapy, chemotherapy, and immunotherapy. | | 90 | 70 | | | | 80 | Response2 |
| QiZhenGPT  (with prompt) | The semantics of all three responses are identical | | - | - | | | | - | - |
| QiZhenGPT  (without prompt) | **Response 1:** For stage IVA cervical spine cancer with enlarged lymph nodes, treatment usually includes surgery, radiotherapy and chemotherapy. Surgery remains the mainstay of treatment, and more extensive resection may be needed to prevent tumor recurrence. Radiotherapy and chemotherapy may be used as adjuvant treatment to help shrink the tumor and relieve symptoms.  **Response 2:** For stage IVA cervical spine cancer with lymph node enlargement, treatment measures usually include surgery, radiotherapy and chemotherapy. Surgery remains an important means of possible cure, especially for patients who may be cured at an early stage. Radiotherapy and chemotherapy may be used as complementary treatments to surgery or for patients who are inoperable.  **Response 3:** For stage IVA cervical spine cancer with lymph node enlargement, treatment usually includes surgery, radiotherapy and chemotherapy. Surgery remains the treatment of choice and may require more extensive resection to prevent lymphatic metastasis. Radiotherapy and chemotherapy may be used as adjuvant therapy to help shrink the tumor. | | 87 | 95 | | | | 93 | Response3 |
| **83:In cervical cancer treatment, if the stage is IVB, what treatment measures should be taken?** | | | | | | | | | |
| Phi-2  (with prompt) | **Response 1:** In cervical cancer treatment, if the stage is ⅣB, the treatment measures should include surgery, radiation therapy, and chemotherapy. Surgery is the primary treatment for stage ⅣB cervical cancer, which involves the removal of the uterus, cervix, and nearby lymph nodes. Radiation therapy may also be used to target any remaining cancer cells in the pelvic area. Chemotherapy is typically used in combination with surgery and radiation therapy to kill any cancer cells that may have spread beyond the pelvic area.  **Response 2:** In cervical cancer treatment, if the stage is ⅣB, the treatment measures should include surgery, radiation therapy, and chemotherapy. Surgery is the primary treatment for stage ⅣB cervical cancer, which involves the removal of the uterus, cervix, and nearby lymph nodes. Radiation therapy is also used to kill cancer cells and prevent them from spreading. Chemotherapy is used to destroy cancer cells that may have spread to other parts of the body.  **Response 3:** In cervical cancer treatment, if the stage is ⅣB, the treatment measures that should be taken include surgery, radiation therapy, and chemotherapy. Surgery involves the removal of the tumor and surrounding tissues, while radiation therapy uses high-energy radiation to kill cancer cells. Chemotherapy uses drugs to kill cancer cells throughout the body. | | 100 | 70 | | | | 75 | Response1 |
| Phi-2  (without prompt) | **Response 1:** If the stage is ⅣB, the treatment measures should include surgery to remove the tumor and surrounding tissues, followed by radiation therapy to kill any remaining cancer cells.  **Response 2:** If the stage is ⅣB, the treatment measures should include surgery to remove the tumor and surrounding tissue, followed by radiation therapy and chemotherapy.  **Response 3:** If the stage is ⅣB, the treatment measures that should be taken are surgery, radiation therapy, chemotherapy, and immunotherapy. | | 98 | 70 | | | | 65 | Response1 |
| QiZhenGPT  (with prompt) | The semantics of all three responses are identical | | - | - | | | | - | - |
| QiZhenGPT  (without prompt) | **Response 1:** For stage IVB cervical spine cancer, treatment measures usually include surgery, radiotherapy and chemotherapy. Surgery may be the primary treatment for complete removal of tumor tissue. Radiotherapy and chemotherapy may be used as adjuvant treatments to help shrink the tumor and relieve symptoms. In some cases, targeted therapy and immunotherapy may also be used to treat cervical spine cancer.  **Response 2:** For stage IVA cervical spine cancer with enlarged lymph nodes, treatment usually includes surgery, radiotherapy and chemotherapy. Surgery remains the mainstay of treatment, and more extensive resection may be needed to prevent tumor recurrence. Radiotherapy and chemotherapy may be used as adjuvant treatments to help shrink the tumor and relieve symptoms.  **Response 3:** For stage IVB cervical spine cancer, treatment measures usually include surgery, radiotherapy and chemotherapy. Surgery may be the treatment of choice because the tumor can be completely removed. Radiotherapy and chemotherapy may be used as adjuvant treatments to help shrink the tumor and relieve symptoms. In some cases, radiation or chemotherapy may also be needed to treat metastases. | | 97 | 96 | | | | 98 | Responsse2 |
| **84: After radical surgery for early cervical cancer, if the abdominal aortic lymph nodes are negative but highrisk factors are present, what treatment measures should be taken?** | | | | | | | | | |
| Phi-2  (with prompt) | **Response 1:** If the abdominal aortic lymph nodes are negative but high-risk factors are present, the patient should be treated with adjuvant chemotherapy.  **Response 2:** If the abdominal aortic lymph nodes are negative but high-risk factors are present, the patient should undergo adjuvant chemotherapy.  **Response 3:** If the abdominal aortic lymph nodes are negative but high-risk factors are present, the patient should be treated with adjuvant chemotherapy. | | 95 | 100 | | | | 90 | Response1 |
| Phi-2  (without prompt) | **Response 1:** If the abdominal aortic lymph nodes are negative but high-risk factors are present, the patient should receive adjuvant chemotherapy.  **Response 2:** If the abdominal aortic lymph nodes are negative but high-risk factors are present, the patient should receive adjuvant chemotherapy.  **Response 3:** If the abdominal aortic lymph nodes are negative but high-risk factors are present, the patient should undergo adjuvant chemotherapy. | | 100 | 82 | | | | 81 | Response1 |
| QiZhenGPT  (with prompt) | **Response 1:** After radical surgery for early cervical cancer, if the abdominal aortic lymph nodes are negative but high-risk factors are present, what treatment measures should be taken?  Answer: The treatment measures should be considered based on the presence of high-risk factors for recurrence and the stage of the disease.  **Response 2:** After radical surgery for early cervical cancer, if the abdominal aortic lymph nodes are negative but high-risk factors are present, what treatment measures should be taken?  Answer: The patient should be referred to a radiation oncologist for further treatment planning.  **Response 3:** After radical surgery for early cervical cancer, if the abdominal aortic lymph nodes are negative but high-risk factors are present, what treatment measures should be taken?  The preferred clinical diagnosis of cervical cancer is stage IA1. | | 0 | 0 | | | | 0 | Response1 |
| QiZhenGPT  (without prompt) | **Response 1:** In this case, ICRT can be used as an alternative or in addition to conventional radiotherapy. The main difference between ICRT and conventional radiotherapy is that ICRT uses images to guide the radiation dose, while conventional radiotherapy relies on the radiation oncologist's expertise to determine the dose.  **Response 2:** The main goals of palliative care are to improve the quality of life for patients with advanced cancer, and to manage symptoms and side effects. Palliative care can be combined with radiotherapy and chemotherapy in patients with metastatic cervical cancer by providing support and management of symptoms, and by helping to ensure that treatment is tailored to the individual needs of the patient.  **Response 3:** The main goals of palliative care are to improve the quality of life for patients with advanced cancer, and to manage symptoms and side effects. Palliative care can be combined with radiotherapy and chemotherapy in patients with metastatic cervical cancer by providing support and management of symptoms, and by helping to ensure that treatment is tailored to the individual needs of the patient. | | 0 | 56 | | | | 100 | Response3 |
| **85:After radical surgery for early cervical cancer, if the abdominal aortic lymph nodes are negative but intermediaterisk factors are present, what treatment measures should be taken?** | | | | | | | | | |
| Phi-2  (with prompt) | The semantics of all three responses are identical | | - | - | | | | - | - |
| Phi-2  (without prompt) | The semantics of all three responses are identical | | - | - | | | | - | - |
| QiZhenGPT  (with prompt) | **Response 1:** After radical surgery for early cervical cancer, if the abdominal aortic lymph nodes are negative but intermediate-risk factors are present, what treatment measures should be taken?  Answer: The treatment measures should be considered based on the presence of intermediate-risk factors for recurrence and the stage of the disease.  **Response 2:** After radical surgery for early cervical cancer, if the abdominal aortic lymph nodes are negative but intermediate-risk factors are present, what treatment measures should be taken?  Answer: The patient should be referred to a radiation oncologist for further treatment planning.  **Response 3:** After radical surgery for early cervical cancer, if the abdominal aortic lymph nodes are negative but intermediate-risk factors are present, what treatment measures should be taken?  The preferred clinical diagnosis of cervical cancer is stage IA2 | | 50 | 0 | | | | 0 | Response1 |
| QiZhenGPT  (without prompt) | **Response 1:** After radical surgery for early cervical cancer, if the abdominal aortic lymph nodes are negative but high-risk factors are present, treatment measures that may be considered include:  * Close surveillance and regular follow-up with the cancer team to monitor for recurrence  * Participation in clinical trials that focus on cervical cancer follow-up and treatment  * Additional therapies such as chemotherapy, radiation therapy, or brachytherapy may be recommended based on the individual patient's needs and circumstances  **Response 2:** For stage IVB cervical spine cancer, treatment measures usually include surgery, radiotherapy and chemotherapy. Surgery may be the treatment of choice, especially for patients who may be cured at an early stage. Radiotherapy and chemotherapy may be used as complementary treatments to surgery or for patients who are inoperable. In some cases, targeted therapy and immunotherapy may also be used to treat cervical spine cancer.  **Response 3:** After radical surgery for early cervical cancer, if the abdominal aortic lymph nodes are negative but high-risk factors are present, treatment measures that may be considered include: | | 43 | 38 | | | | 71 | Response2 |
| **86:After radical surgery for early cervical cancer, if the abdominal aortic lymph nodes are positive but there is no distant metastasis, what treatment measures should be taken?** | | | | | | | | | |
| Phi-2  (with prompt) | **Response 1:** If the abdominal aortic lymph nodes are positive but there is no distant metastasis, the patient should be treated with adjuvant chemotherapy.  **Response 2:** If the abdominal aortic lymph nodes are positive but there is no distant metastasis, the patient should undergo adjuvant chemotherapy.  **Response 3:** If the abdominal aortic lymph nodes are positive but there is no distant metastasis, the patient should be treated with adjuvant chemotherapy. | | 95 | 100 | | | | 85 | Response3 |
| Phi-2  (without prompt) | **Response 1:** If the abdominal aortic lymph nodes are positive but there is no distant metastasis, the patient should receive adjuvant chemotherapy.  **Response 2:** If the abdominal aortic lymph nodes are positive but there is no distant metastasis, the patient should receive adjuvant chemotherapy.  **Response 3:** If the abdominal aortic lymph nodes are positive but there is no distant metastasis, the patient should undergo adjuvant chemotherapy. | | 100 | 72 | | | | 75 | Response1 |
| QiZhenGPT  (with prompt) | **Response 1:** After radical surgery for early cervical cancer, if the abdominal aortic lymph nodes are positive but there is no distant metastasis, what treatment measures should be taken?  Answer: The treatment measures should be considered based on the stage of the disease and the presence of distant metastasis.  **Response 2:** After radical surgery for early cervical cancer, if the abdominal aortic lymph nodes are positive but there is no distant metastasis, what treatment measures should be taken?  Answer: The patient should be referred to a radiation oncologist for further treatment planning.  **Response 3:** After radical surgery for early cervical cancer, if the abdominal aortic lymph nodes are positive but there is no distant metastasis, what treatment measures should be taken?  The treatment for stage IA1 cervical cancer with positive abdominal aortic lymph nodes but no distant metastasis is radiation therapy. | | 100 | 0 | | | | 0 | Response1 |
| QiZhenGPT  (without prompt) | **Response 1:** After radical surgery for early cervical cancer, if the abdominal aortic lymph nodes are negative but high-risk factors are present, treatment measures that may be considered include:  * Close surveillance and regular follow-up with the cancer team to monitor for recurrence  * Participation in clinical trials that focus on cervical cancer follow-up and treatment  * Additional therapies such as chemotherapy, radiation therapy, or brachytherapy may be recommended based on the individual patient's needs and circumstances  **Response 2:** For stage IVB cervical spine cancer, treatment measures usually include surgery, radiotherapy and chemotherapy. Surgery may be the treatment of choice, especially for patients who may be cured at an early stage. Radiotherapy and chemotherapy may be used as complementary treatments to surgery or for patients who are inoperable. In some cases, targeted therapy and immunotherapy may also be used to treat cervical spine cancer.  **Response 3:** After radical surgery for early cervical cancer, if the abdominal aortic lymph nodes are negative but high-risk factors are present, treatment measures that may be considered include:  * Close surveillance and regular follow-up with the cancer team to monitor for any changes in the patient's condition.  * Additional lymph node sampling or biopsy to further evaluate the status of the lymph nodes.  * Consider adjuvant treatment such as chemotherapy, radiotherapy, or immunotherapy to reduce the risk of recurrence. | | 95 | 70 | | | | 72 | Response1 |
| **87: What are the surgical treatment options for patients with FIGO stage IA1 cervical cancer? Are they suitable for patients with preserved fertility?** | | | | | | | | | |
| Phi-2  (with prompt) | The semantics of all three responses are identical | | - | - | | | | - | - |
| Phi-2  (without prompt) | The semantics of all three responses are identical | | - | - | | | | - | - |
| QiZhenGPT  (with prompt) | The semantics of all three responses are identical | | - | - | | | | - | - |
| QiZhenGPT  (without prompt) | **Response 1:** The surgical treatment options for patients with stage IA1 cervical cancer include surgery alone or surgery in combination with radiation therapy or chemotherapy. They are suitable for patients with preserved fertility.  **Response 2:** No  **Response 3:** Surgical treatment options for stage IA1 cervical cancer are total laparoscopic surgery and laparoscopic nodeectomy. They are suitable for patients with preserved fertility. | | 100 | 74 | | | | 50 | Responsse1 |
| **88: In patients with FIGO stage IB2 and IIA1, what factors determine the choice between surgery and radiotherapy? What are the differences in outcomes between the two modalities?** | | | | | | | | | |
| Phi-2  (with prompt) | **Response 1:** The choice between surgery and radiotherapy in patients with FIGO stage IB2 and IIA1 is based on factors such as the patient's age, overall health, and the extent of the tumor. In some cases, surgery may be preferred if the tumor is small and localized, while radiotherapy may be recommended if the tumor is larger or if the patient is not a suitable candidate for surgery. The outcomes of surgery and radiotherapy are generally similar in terms of local control and overall survival, but surgery may have a higher risk of complications such as infection and bleeding.  **Response 2:** The choice between surgery and radiotherapy in patients with FIGO stage IB2 and IIA1 cervical cancer depends on various factors, including the patient's overall health, the extent of the tumor, and the presence of any metastases. In general, surgery is preferred for patients who are in good health and have a small tumor that is confined to the cervix. Radiotherapy is usually recommended for patients who are not suitable candidates for surgery or have a larger tumor that has spread to nearby lymph nodes. The outcomes of surgery and radiotherapy are similar in terms of overall survival and disease-free survival, but surgery may have a higher risk of complications such as infection and bleeding.  **Response 3:** In patients with FIGO stage IB2 and IIA1, the choice between surgery and radiotherapy is based on the patient's age, comorbidities, and overall health status. The decision is also influenced by the extent of the tumor and the presence of lymph node involvement. The outcomes of surgery and radiotherapy are similar in terms of overall survival and disease-free survival, but surgery may result in better cosmetic outcomes. | | 83 | 97 | | | | 92 | Response1 |
| Phi-2  (without prompt) | **Response 1:** The main factors that determine the choice between surgery and radiotherapy in patients with FIGO stage IB2 and IIA1 cervical cancer are the patient's age, comorbidities, and overall health status. The choice of treatment modality is also influenced by the patient's preference and the availability of resources.  **Response 2:** In patients with FIGO stage IB2 and IIA1, what factors determine the choice between surgery and radiotherapy? What are the differences in outcomes between the two modalities?  **Response 3:** The main factors that determine the choice between surgery and radiotherapy in patients with FIGO stage IB2 and IIA1 cervical cancer are the patient's age, overall health, and the extent of the tumor. In general, surgery is preferred for younger patients with smaller tumors, while radiotherapy is preferred for older patients or those with larger tumors. | | 95 | 90 | | | | 97 | Response3 |
| QiZhenGPT  (with prompt) | **Response 1:** In patients with FIGO stage IB2 and IIA1, what factors determine the choice between surgery and radiotherapy? What are the differences in outcomes between the two modalities?  Answer: Factors such as patient age, weight, and overall health, as well as the presence of any pre-existing medical conditions. The outcomes of surgery and radiotherapy are generally similar in terms of survival and recurrence rates, but they may differ in terms of the degree of tissue damage and the potential for complications.  **Response 2:** In patients with FIGO stage IB2 and IIA1, what factors determine the choice between surgery and radiotherapy? What are the differences in outcomes between the two modalities?  Answer: Factors such as patient age, stage, and overall health should be considered when making a decision between surgery and radiotherapy. The outcomes of the two modalities can vary, with surgery generally offering better preservation of tissue function and a lower risk of complications. Radiotherapy, on the other hand, can be more effective in treating advanced stages of cervical cancer and can be used to treat patients who are not candidates for surgery due to their poor health status.  **Response 3:** In patients with FIGO stage IB2 and IIA1, what factors determine the choice between surgery and radiotherapy? What are the differences in outcomes between the two modalities?  Answer: Factors such as patient age, stage, and overall health should be considered when making a decision between surgery and radiotherapy for patients with FIGO stage IB2 and IIA1 cervical cancer. The outcomes of the two modalities can vary, with surgery generally offering better outcomes in terms of survival and quality of life, while radiotherapy can provide better control over the spread of the cancer. | | 90 | 95 | | | | 85 | Response3 |
| QiZhenGPT  (without prompt) | **Response 1:** Factors determining the choice between surgery and radiotherapy in patients withFIGO stage IB2 and IIA1 include the patient's general health status, the presence or absence of other cancer, the extent of the cancer in the uterus, and the desire of the patient to have children. The outcomes between surgery and radiotherapy are generally similar, but surgery may have a slightly better outcome in some cases.  **Response 2:** Yes  **Response 3:** Factors determining the choice between surgery and radiotherapy in patients withFIGO stage IB2 and IIA1 include the patient's general health status, the presence or absence of evidence of disease in other parts of the body, and the expected outcomes of each modality. The choice between the two modalities is often based on a careful consideration of the patient's individual needs and preferences. The outcomes of surgery and radiotherapy are generally similar, but surgery may be preferred in cases where the cancer is localized and there is a good chance of a complete removal of the tumor. Radiotherapy may be preferred in cases where the cancer is not localized or there is a risk of recurrence. | | 90 | 85 | | | | 95 | Response2 |
| **89: What is the recommended treatment of choice for FIGO stage IB3 cervical cancer?** | | | | | | | | | |
| Phi-2  (with prompt) | **Response 1:** The recommended treatment of choice for FIGO stage IB3 cervical cancer is a combination of surgery and radiotherapy.  **Response 2:** The recommended treatment of choice for FIGO stage IB3 cervical cancer is a combination of surgery and radiotherapy. This approach is known as radical hysterectomy with pelvic lymphadenectomy and pelvic radiotherapy.  **Response 3:** The recommended treatment of choice for FIGO stage IB3 cervical cancer is radical hysterectomy with pelvic lymphadenectomy. | | 100 | 71 | | | | 77 | Response1 |
| Phi-2  (without prompt) | **Response 1:** The recommended treatment of choice for FIGO stage IB3 cervical cancer is radical hysterectomy with pelvic lymphadenectomy.  **Response 2:** What is the recommended treatment of choice for FIGO stage IB3 cervical cancer?  **Response 3:** The recommended treatment of choice for FIGO stage IB3 cervical cancer is a combination of surgery and radiotherapy. | | 100 | 39 | | | | 46 | Response2 |
| QiZhenGPT  (with prompt) | **Response 1:** What is the recommended treatment of choice for FIGO stage IB3 cervical cancer?  Answer: The recommended treatment of choice for FIGO stage IB3 cervical cancer is surgery. Radiotherapy may also be considered in some cases, particularly if the patient has a high risk of recurrence.  **Response 2:** What is the recommended treatment of choice for FIGO stage IB3 cervical cancer?  Answer: The recommended treatment of choice for FIGO stage IB3 cervical cancer is surgery. Radiotherapy can also be used as a treatment option, but it is not typically recommended due to its potential for complications and low effectiveness in treating early-stage cervical cancer.  **Response 3:** What is the recommended treatment of choice for FIGO stage IB3 cervical cancer?  Answer: The recommended treatment of choice for FIGO stage IB3 cervical cancer is surgery. Radiotherapy is also an option, but it is generally not recommended due to its potential for side effects and low survival rates. | | 92 | 84 | | | | 88 | Response1 |
| QiZhenGPT  (without prompt) | **Response 1:** The recommended treatment of choice forFIGO stage IB3 cervical cancer is surgery.  **Response 2:** Factors determining the choice between surgery and radiotherapy in patients withFIGO stage IB2 and IIA1 include the patient's general health, the presence or absence of lymph nodes, and the extent of the cancer in the uterus or cervix. The choice between the two modalities is often based on a combination of these factors, as well as the patient's preferences. The outcomes between the two modalities are generally similar, with surgery having a slightly higher five-year survival rate.  **Response 3:** The recommended treatment of choice for Figure stage IB3 cervical cancer is a combination of surgery and radiotherapy. This approach allows for a more comprehensive treatment of the cancer and can improve outcomes. | | 85 | 90 | | | | 95 | Response3 |
| **90: what is the difference between the different types of radical hysterectomy? For which patients is it indicated?** | | | | | | | | | |
| Phi-2  (with prompt) | **Response 1:** Radical hysterectomy can be performed in different ways, including total abdominal hysterectomy, subtotal hysterectomy, and radical hysterectomy with pelvic lymphadenectomy. The choice of procedure depends on factors such as the extent of the tumor, the patient's age and overall health, and the presence of other medical conditions. Radical hysterectomy is indicated for patients with FIGO stage IB3 cervical cancer, as it provides the best chance of achieving local control and preventing recurrence.  **Response 2:** There are several types of radical hysterectomy, including total hysterectomy, subtotal hysterectomy, and radical hysterectomy with lymphadenectomy. The choice of procedure depends on the extent of the tumor and the patient's overall health. Total hysterectomy involves the removal of the entire uterus, while subtotal hysterectomy involves the removal of part of the uterus. Radical hysterectomy with lymphadenectomy involves the removal of the uterus, cervix, and nearby lymph nodes. This procedure is indicated for patients with a large tumor that has spread to nearby lymph nodes.  **Response 3:** The different types of radical hysterectomy include total hysterectomy, subtotal hysterectomy, and radical hysterectomy with pelvic lymphadenectomy. Total hysterectomy is indicated for patients with small tumors and no lymph node involvement. Subtotal hysterectomy is indicated for patients with larger tumors and positive lymph nodes. Radical hysterectomy with pelvic lymphadenectomy is indicated for patients with large tumors and positive lymph nodes. | | 97 | 95 | | | | 98 | Response3 |
| Phi-2  (without prompt) | **Response 1:** Radical hysterectomy can be performed through different approaches, including abdominal, vaginal, and laparoscopic. The choice of approach depends on the patient's anatomy, comorbidities, and surgeon's preference. Radical hysterectomy is indicated for patients with FIGO stage IB3 cervical cancer.  **Response 2:** what is the difference between the different types of radical hysterectomy? For which patients is it indicated?  **Response 3:** The different types of radical hysterectomy include total hysterectomy, subtotal hysterectomy, and radical hysterectomy with pelvic lymphadenectomy. Total hysterectomy involves the removal of the entire uterus, while subtotal hysterectomy involves the removal of the cervix and part of the uterus. Radical hysterectomy with pelvic lymphadenectomy involves the removal of the uterus, cervix, and surrounding lymph nodes. Radical hysterectomy is indicated for patients with larger tumors or those who are not suitable candidates for radiotherapy. | | 74 | 67 | | | | 80 | Response3 |
| QiZhenGPT  (with prompt) | **Response 1:** what is the difference between the different types of radical hysterectomy? For which patients is it indicated?  Answer: radical hysterectomy involves the removal of all the tissue surrounding the uterus, including the vaginal wall, cervix, and any surrounding tissue. These procedures are typically indicated for patients with advanced or recurrent cervical cancer who are not candidates for surgery alone. The different types of radical hysterectomy include total hysterectomy, and partial hysterectomy with sphenoidectomy. These procedures are typically used in patients who have advanced or recurrent cervical cancer and are not candidates for surgery alone.  **Response 2:** what is the difference between the different types of radical hysterectomy? For which patients is it indicated?  Answer: radical hysterectomy refers to a surgical procedure that involves the removal of all the tissue surrounding a specific area of the body, such as the uterus, cervix, and vagina. There are several different types of radical hysterectomy, including total hysterectomy, partial hysterectomy, and vaginal hysterectomy. These procedures are typically indicated for patients with advanced stages of cervical cancer who are not candidates for surgery due to their poor health status.  **Response 3:** what is the difference between the different types of radical hysterectomy? For which patients is it indicated?  Answer: radical hysterectomy involves the removal of the entire uterus, including the cervix, in patients with FIGO stage IB3 cervical cancer. It is indicated for patients who have advanced or recurrent cervical cancer and are not candidates for surgery or radiotherapy. | | 89 | 63 | | | | 67 | Response2 |
| QiZhenGPT  (without prompt) | **Response 1:** There are three main types of radical hysterectomy: total hysterectomy, subtotal hysterectomy, and radical hysterectomy with lymph node dissection. The choice of which type of radical hysterectomy to perform depends on the patient's specific cancer and the preferences of the surgical team. For example, total hysterectomy may be indicated for patients with large cancer or deep infiltration into the uterus, while subtotal hysterectomy may be indicated for patients with smaller cancer or less infiltration. Radical hysterectomy with lymph node dissection may be indicated for patients with cancer that has spread to the lymph nodes.  **Response 2:** There are several types of radical hysterectomy, including total hysterectomy, subtotal hysterectomy, and Robotic-assisted hysterectomy. The choice of which type of radical hysterectomy to perform depends on the patient's specific needs and the extent of the cancer. For example, total hysterectomy is typically used for patients with a large cancer that involves the entire uterus, while subtotal hysterectomy is used for patients with cancer that involves only a portion of the uterus. Robotic-assisted hysterectomy is a newer type of radical hysterectomy that uses a robotic system to assist the surgeon in performing the procedure.  **Response 3:** There are several different types of radical hysterectomy, including total hysterectomy, subtotal hysterectomy, and radical hysterectomy with lymph node sampling. The choice of which type of hysterectomy to perform depends on the specific needs of the patient and the type and stage of cancer. For example, a total hysterectomy may be indicated for patients with a large cancer that is involving the entire uterus, while a subtotal hysterectomy may be indicated for patients with cancer that is primarily in the lower part of the uterus. Radical hysterectomy with lymph node sampling may be indicated for patients with cancer that is involving the lymph nodes in the pelvis. | | 89 | 95 | | | | 89 | Response1 |
| **91: What are the advantages of IMRT in radiotherapy for cervical cancer?** | | | | | | | | | |
| Phi-2  (with prompt) | **Response 1:** IMRT offers several advantages over conventional radiotherapy for cervical cancer. It allows for more precise targeting of the tumor, which can help to minimize damage to surrounding healthy tissue. This can lead to fewer side effects and a better quality of life for the patient. IMRT also allows for the delivery of higher doses of radiation to the tumor, which can improve the chances of achieving local control and preventing recurrence.  **Response 2:** IMRT is a type of radiotherapy that uses high-energy X-rays to target cancer cells while minimizing damage to surrounding healthy tissue. The advantages of IMRT in radiotherapy for cervical cancer include better tumor control, fewer side effects, and improved quality of life.  **Response 3:** The advantages of IMRT in radiotherapy for cervical cancer include better dose distribution, reduced toxicity, and improved cosmetic outcomes. | | 95 | 80 | | | | 90 | Response1 |
| Phi-2  (without prompt) | **Response 1:** IMRT is a type of radiotherapy that delivers high doses of radiation to the tumor while sparing the surrounding healthy tissues. The advantages of IMRT in radiotherapy for cervical cancer include better tumor control, reduced side effects, and improved quality of life.  **Response 2:** What are the advantages of IMRT in radiotherapy for cervical cancer?  **Response 3:** The advantages of IMRT in radiotherapy for cervical cancer include a more precise delivery of radiation, which can reduce the risk of damage to surrounding healthy tissue. IMRT can also be used to target tumors that are difficult to reach with traditional radiotherapy techniques. | | 78 | 74 | | | | 91 | Response2 |
| QiZhenGPT  (with prompt) | **Response 1:** What are the advantages of IMRT in radiotherapy for cervical cancer?  Answer: IMRT is a type of radiation therapy that uses high-energy photons to deliver precise radiation to the target area. It has several advantages over radiotherapy, including reduced side effects, improved image quality, and the ability to deliver radiation to specific areas of the body. IMRT may be more effective at reducing the risk of recurrence in patients with advanced or recurrent cervical cancer who are not candidates for surgery alone.  **Response 2:** What are the advantages of IMRT in radiotherapy for cervical cancer?  Answer: IMRT is a type of radiation therapy that uses high-energy photons to deliver radiation to the target area. It offers several advantages over traditional radiotherapy, including improved image guidance, reduced side effects, and improved patient comfort. IMRT can be used to treat early-stage cervical cancer and can be effective in reducing the risk of recurrence and improving the overall survival rate.  **Response 3:** What are the advantages of IMRT in radiotherapy for cervical cancer?  Answer: IMRT offers improved control over the dose of radiation to the cervix and can reduce the risk of side effects associated with radiotherapy. It is also more efficient than traditional radiotherapy and can be used to treat patients with advanced or recurrent cervical cancer. | | 92 | 78 | | | | 85 | Response2 |
| QiZhenGPT  (without prompt) | **Response 1:** IMRT (Intensity Modulated Radiation Therapy) in radiotherapy for cervical cancer has several advantages, including the ability to deliver a more precise and personalized treatment, the potential for improved outcome, and the ability to reduce side effects.  **Response 2:** The advantages of IMRT in radiotherapy for cervical cancer include its ability to deliver a high dose of radiation to the tumor with fewer side effects to the surrounding healthy tissue. This approach can help to improve outcomes and reduce the risk of recurrence compared to traditional radiation therapy.  **Response 3:** The advantages of intensity modulated radiation therapy (IMRT) in radiotherapy for cervical cancer include improved treatment accuracy and targeting, reduced side effects, and improved outcomes. | | 85 | 95 | | | | 90 | Response1 |
| **92: What are the common sites of recurrence in cervical cancer? How is the risk of recurrence monitored?** | | | | | | | | | |
| Phi-2  (with prompt) | **Response 1:** The common sites of recurrence in cervical cancer include the pelvis, vagina, and bladder. The risk of recurrence is monitored through regular follow-up appointments, which may include imaging studies such as CT scans or MRI scans. In some cases, additional treatments such as chemotherapy or radiation therapy may be recommended if a recurrence is detected.  **Response 2:** The common sites of recurrence in cervical cancer include the pelvis, vagina, and bladder. The risk of recurrence is monitored through regular follow-up appointments, imaging tests, and blood tests. Patients who are at high risk of recurrence may be recommended for additional treatments such as chemotherapy or radiation therapy.  **Response 3:** The common sites of recurrence in cervical cancer include the pelvis, vagina, and bladder. The risk of recurrence is monitored through regular follow-up imaging studies, such as CT scans and MRI. | | 85 | 95 | | | | 90 | Response3 |
| Phi-2  (without prompt) | The semantics of all three responses are identical | | - | - | | | | - | - |
| QiZhenGPT  (with prompt) | The semantics of all three responses are identical | | - | - | | | | - | - |
| QiZhenGPT  (without prompt) | **Response 1:** Common sites of recurrence in cervical cancer include the lung, bone, and soft tissue. The risk of recurrence can be monitored through imaging studies such as CT scans and PET scans.  **Response 2:** Common sites of recurrence in cervical cancer include the lung, bone, and liver. The risk of recurrence can be monitored through regular imaging and blood tests, as well as by periodically reviewing the patient's treatment plan.  **Response 3:** Common sites of recurrence in cervical cancer include the lymph nodes, the lung, and the bone. The risk of recurrence can be monitored through regular follow-up care, including imaging studies and blood tests. | | 80 | 70 | | | | 75 | Response1 |
| **93: What are the recommended treatment strategies for recurrent cervical cancer? Can surgery, radiotherapy, and chemotherapy be combined?** | | | | | | | | | |
| Phi-2  (with prompt) | The semantics of all three responses are identical | | - | - | | | | - | - |
| Phi-2  (without prompt) | The semantics of all three responses are identical | | - | - | | | | - | - |
| QiZhenGPT  (with prompt) | The semantics of all three responses are identical | | - | - | | | | - | - |
| QiZhenGPT  (without prompt) | The semantics of all three responses are identical | | - | - | | | | - | - |
| **94: What are the treatment strategies for metastatic cervical cancer? Are there specific treatments at different metastatic sites?** | | | | | | | | | |
| Phi-2  (with prompt) | **Response 1:** The treatment strategies for metastatic cervical cancer vary depending on the specific metastatic site. For example, patients with liver metastases may receive chemotherapy, while those with lung metastases may undergo radiotherapy.  **Response 2:** The treatment strategies for metastatic cervical cancer vary depending on the specific metastatic site. For example, patients with liver metastases may receive chemotherapy, while those with bone metastases may undergo radiotherapy.  **Response 3:** The treatment strategies for metastatic cervical cancer depend on the specific metastatic site. Different treatments may be recommended for different sites, such as the liver, lungs, bones, or brain. | | 100 | 77 | | | | 81 | Response2 |
| Phi-2  (without prompt) | The semantics of all three responses are identical | | - | - | | | | - | - |
| QiZhenGPT  (with prompt) | **Response 1:** For metastatic cervical cancer, the treatment strategies include surgery, radiotherapy, and chemotherapy. The specific treatments at different metastatic sites may vary depending on the patient's condition and the location of the cancer.  **Response 2:** The treatment strategies for metastatic cervical cancer vary depending on the specific metastatic site. For example, there are different treatments for metastatic nodes in the neck, lung, and bone.  **Response 3:** The treatment strategies for metastatic cervical cancer include specific treatments at different metastatic sites. | | 89 | 100 | | | | 86 | Response3 |
| QiZhenGPT  (without prompt) | The semantics of all three responses are identical | | - | - | | | | - | - |
| **95: What are the treatment strategies for patients with cervical cancer during pregnancy? How does the treatment differ in early, intermediate, and advanced stages of pregnancy?** | | | | | | | | | |
| Phi-2  (with prompt) | The semantics of all three responses are identical | | - | - | | | | - | - |
| Phi-2  (without prompt) | **Response 1:** The treatment strategies for patients with cervical cancer during pregnancy are tailored to the stage of pregnancy. In early pregnancy, surgery may be considered, while in intermediate and advanced stages, chemotherapy may be recommended.  **Response 2:** The treatment strategies for patients with cervical cancer during pregnancy are tailored to the stage of pregnancy. In early pregnancy, surgery may be considered, while in intermediate and advanced stages, radiotherapy and chemotherapy are typically used.  **Response 3:** The treatment strategies for patients with cervical cancer during pregnancy vary depending on the stage of pregnancy. In early pregnancy, surgery may be considered, while in intermediate and advanced stages, radiotherapy and chemotherapy may be used. | | 95 | 85 | | | | 90 | Response2 |
| QiZhenGPT  (with prompt) | **Response 1:** For patients with cervical cancer during pregnancy, the treatment strategies vary depending on the stage of pregnancy. In early pregnancy, surgery may be considered to remove the cancer before it causes any complications. In intermediate and advanced stages of pregnancy, the treatment may differ from that in early pregnancy, and it may involve surgery at a later stage.  **Response 2:** The treatment strategies for patients with cervical cancer during pregnancy vary depending on the stage of pregnancy. In early pregnancy, treatment may be recommended to prevent complications such as labor and delivery. In intermediate and advanced pregnancy stages, treatment may differ depending on the patient's overall health and the potential risks and benefits of the treatment.  **Response 3:** The treatment strategies for patients with cervical cancer during pregnancy vary depending on the stage of pregnancy. In early, intermediate, and advanced stages, treatment differs. | | 90 | 80 | | | | 95 | Response2 |
| QiZhenGPT  (without prompt) | The semantics of all three responses are identical | | - | - | | | | - | - |
| **96: Are patients with cervical cancer in pregnancy suitable for surgery? At what stage of pregnancy should surgery be considered?** | | | | | | | | | |
| Phi-2  (with prompt) | **Response 1:** Patients with cervical cancer in pregnancy can be considered for surgery, but it is important to assess the stage of pregnancy and the potential risks to the mother and fetus. Surgery should be performed at the appropriate stage to minimize any potential complications.  **Response 2:** Patients with cervical cancer in pregnancy can be considered for surgery, but it is typically recommended in the early stages of pregnancy. The timing of surgery depends on the individual case and the stage of pregnancy.  **Response 3:** Patients with cervical cancer in pregnancy can be considered for surgery, but it is important to assess the stage of pregnancy and the potential risks to the mother and fetus. Surgery should be performed at the appropriate stage to minimize risks. | | 95 | 100 | | | | 85 | Response1 |
| Phi-2  (without prompt) | The semantics of all three responses are identical | - | | | - | | - | | - |
| QiZhenGPT  (with prompt) | The semantics of all three responses are identical | | - | - | | - | | | - |
| QiZhenGPT  (without prompt) | The semantics of all three responses are identical | | - | - | | | | - | - |
| **97: What is the radiotherapy strategy for patients with locally advanced cervical cancer? When is a combination of chemotherapy recommended?** | | | | | | | | | |
| Phi-2  (with prompt) | The semantics of all three responses are identical | | - | - | | | | - | - |
| Phi-2  (without prompt) | The semantics of all three responses are identical | | - | - | | | | - | - |
| QiZhenGPT  (with prompt) | **Response 1:** For patients with locally advanced cervical cancer, radiotherapy may be recommended as a treatment option. The recommended timing for this treatment will depend on the patient's overall health and the location of the cancer. A combination of chemotherapy and radiotherapy may also be recommended to improve the effectiveness of the treatment.  **Response 2:** The radiotherapy strategy for patients with locally advanced cervical cancer is to use radiotherapy in combination with chemotherapy when the cancer has spread to the local area but not to other parts of the body. This strategy is recommended when the patient has no other options for treatment and the cancer is considered to be potentially resectable.  **Response 3:** radiotherapy for patients with locally advanced cervical cancer involves using chemotherapy as a part of the treatment plan. The recommended time for this combination of chemotherapy to be recommended is after the patient has completed surgery. | | 78 | 72 | | | | 86 | Response1 |
| QiZhenGPT  (without prompt) | The semantics of all three responses are identical | | - | - | | | | - | - |
| **98: When is imageguided brachytherapy (ICRT) necessary and how is it different from conventional radiotherapy?** | | | | | | | | | |
| Phi-2  (with prompt) | **Response 1:** Image-guided brachytherapy (ICRT) is necessary when the tumor is located in a difficult-to-reach area or when the tumor is too large for external beam radiation therapy. ICRT involves placing radioactive sources directly into the tumor using imaging techniques such as CT or MRI. This allows for more precise targeting of the tumor and can result in better treatment outcomes.  **Response 2:** Image-guided brachytherapy (ICRT) is necessary when the tumor is located in a difficult-to-reach area or when the tumor is too large for external beam radiation therapy. ICRT involves placing a radioactive source directly into the tumor using imaging guidance. This allows for a higher dose of radiation to be delivered to the tumor while minimizing exposure to surrounding healthy tissue.  **Response 3:** Image-guided brachytherapy (ICRT) is necessary when the tumor is located in a difficult-to-reach area or when the tumor is too large for conventional radiotherapy. ICRT is different from conventional radiotherapy because it delivers a higher dose of radiation directly to the tumor, while sparing the surrounding healthy tissue. | | 94 | 79 | | | | 89 | Response1 |
| Phi-2  (without prompt) | **Response 1:** Image-guided brachytherapy (ICRT) is necessary when the tumor is located in a difficult-to-reach area or when the tumor is too large for external beam radiation therapy. ICRT involves placing radioactive sources directly into the tumor using imaging guidance. This allows for a higher dose of radiation to be delivered to the tumor while minimizing exposure to surrounding healthy tissue.  **Response 2:** Image-guided brachytherapy (ICRT) is necessary in cases where the tumor is located in a difficult-to-reach area or when the tumor is too large for external beam radiation therapy. ICRT involves placing radioactive sources directly into the tumor using imaging techniques to guide the placement. This allows for a higher dose of radiation to be delivered to the tumor while minimizing exposure to surrounding healthy tissue.  **Response 3:** Image-guided brachytherapy (ICRT) is necessary in cases where the tumor is located in a difficult-to-reach area or when the tumor is too large for external beam radiation therapy. ICRT involves placing radioactive sources directly into the tumor using imaging techniques such as CT or MRI. This allows for more precise targeting of the tumor and can result in better treatment outcomes. | | 100 | 86 | | | | 93 | Response1 |
| QiZhenGPT  (with prompt) | **Response 1:** Image-guided brachytherapy (ICRT) is necessary when traditional radiotherapy is not effective in treating cervical cancer. ICRT uses high-energy radiation sources to deliver precise and targeted radiation to the tumor, while minimizing radiation to surrounding healthy tissue. This approach can improve the effectiveness and efficiency of radiotherapy in treating cervical cancer. However, ICRT is not typically used in routine treatment of cervical cancer, and it is recommended that patients undergo regular imaging studies to monitor their tumor progress.  **Response 2:** Image-guided brachytherapy (ICRT) is necessary when traditional radiotherapy is not effective in treating cervical cancer. ICRT involves using specialized equipment to guide the placement of radioactive seeds into the patient's body, which can be used to treat a variety of sites within the body. In contrast, traditional radiotherapy involves placing radioactive seeds directly into the patient's body, which can be less effective in some cases.  **Response 3:** Image-guided brachytherapy (ICRT) is typically used in patients with advanced stages of cervical cancer who have not responded to traditional radiotherapy. ICRT involves the use of image guidance to deliver radioactive seeds to specific locations within the body, which can help to reduce the risk of side effects and improve the overall effectiveness of the treatment. However, it is important to note that ICRT is not typically used in early-stage cervical cancer, as the disease is typically not advanced enough for this type of treatment. | | 85 | 70 | | | | 75 | Response1 |
| QiZhenGPT  (without prompt) | **Response 1:** When is image-guided brachytherapy (ICRT) necessary and how is it different from conventional radiotherapy? Image-guided brachytherapy (ICRT) is necessary when the tumor is in a location that makes it difficult to perform conventional radiotherapy, such as in the case of cervical cancer involving theWidget  **Response 2:** When is image-guided brachytherapy (ICRT) necessary and how is it different from conventional radiotherapy? Image-guided brachytherapy (ICRT) is necessary when the tumor is in a location that makes it difficult to perform conventional radiotherapy, such as in the case of cervical cancer involving theWidget Placeholder for.  **Response 3:** Image-guided brachytherapy (ICRT) is necessary when the tumor is in a critical position and conventional radiotherapy cannot be used due to the risk of severe side effects. ICRT is different from conventional radiotherapy in that it uses a combination of radiation and chemotherapy to treat the tumor, and it is guided by imaging. | | 100 | 0 | | | | 0 | Response2 |
| **99: What are the methods for monitoring recurrence after treatment of cervical cancer? Is routine imaging recommended:** | | | | | | | | | |
| Phi-2  (with prompt) | **Response 1:** The methods for monitoring recurrence after treatment of cervical cancer include regular imaging studies such as CT or MRI, as well as physical examinations and blood tests. Routine imaging is not typically recommended unless there are specific symptoms or risk factors for recurrence.  **Response 2:** The methods for monitoring recurrence after treatment of cervical cancer include imaging studies such as CT scans, MRI scans, and PET scans. Routine imaging is not recommended unless there are specific symptoms or signs of recurrence.  **Response 3:** The methods for monitoring recurrence after treatment of cervical cancer include physical examination, imaging tests (such as CT or MRI), and blood tests. Routine imaging is not recommended unless there are specific symptoms or risk factors for recurrence. | | 93 | 100 | | | | 89 | Response1 |
| Phi-2  (without prompt) | The semantics of all three responses are identical | | - | - | | | | - | - |
| QiZhenGPT  (with prompt) | **Response 1:** Methods for monitoring recurrence after treatment of cervical cancer include regular follow-up appointments with the patient and their healthcare provider, as well as imaging studies such as CT, MRI, or ultrasound. Routine imaging is important to ensure that the tumor has not recurrence and to identify any potential complications or side effects of treatment.  **Response 2:** Methods for monitoring recurrence after treatment of cervical cancer include regular follow-up appointments with the patient and their healthcare provider, as well as imaging studies such as CT scans, MRI scans, or X-rays. Routine imaging is recommended to ensure that the patient is receiving the best possible treatment and to identify any potential recurrence or new tumors.  **Response 3:** The methods for monitoring recurrence after treatment of cervical cancer include regular follow-up appointments with the patient and their healthcare provider, as well as imaging studies such as CT scans or MRI scans. Routine imaging is recommended to ensure that the patient is receiving the appropriate treatment and to identify any potential recurrence or new tumors that may need to be treated. | | 98 | 95 | | | | 100 | Response1 |
| QiZhenGPT  (without prompt) | **Response 1:** When is image-guided brachytherapy (ICRT) necessary and how is it different from conventional radiotherapy? Image-guided brachytherapy (ICRT) is necessary when the tumor is in a location that makes it difficult to perform conventional radiotherapy, such as in the case of cervical cancer involving theWidget  **Response 2:** Monitoring recurrence after treatment of cervical cancer includes regular imaging and blood tests.  **Response 3:** Monitoring recurrence after treatment of cervical cancer includes regular imaging and blood tests. | | 5 | 39 | | | | 100 | Response3 |
| **100:What are the main goals of palliative care? In patients with metastatic cervical cancer, how can palliative care be combined with radiotherapy and chemotherapy?** | | | | | | | | | |
| Phi-2  (with prompt) | **Response 1:** The main goals of palliative care are to improve the quality of life for patients with advanced cancer and to manage symptoms such as pain, nausea, and fatigue. In patients with metastatic cervical cancer, palliative care can be combined with radiotherapy and chemotherapy to provide symptom relief and improve overall quality of life.  **Response 2:** The main goals of palliative care are to relieve symptoms and improve quality of life for patients with advanced cancer. In patients with metastatic cervical cancer, palliative care can be combined with radiotherapy and chemotherapy to help manage symptoms such as pain, fatigue, and nausea.  **Response 3:** The main goals of palliative care are to relieve pain and other symptoms, improve quality of life, and provide emotional and spiritual support to patients with advanced cancer. In patients with metastatic cervical cancer, palliative care can be combined with radiotherapy and chemotherapy to improve symptom control and quality of life. | | 89 | 76 | | | | 82 | Response1 |
| Phi-2  (without prompt) | **Response 1:** The main goals of palliative care are to improve quality of life and manage symptoms in patients with advanced cancer. In patients with metastatic cervical cancer, palliative care can be combined with radiotherapy and chemotherapy to help manage symptoms such as pain, fatigue, and nausea. This can help improve overall quality of life and allow patients to continue with their daily activities.  **Response 2:** The main goals of palliative care are to relieve symptoms and improve quality of life for patients with advanced or metastatic cervical cancer. Palliative care can be combined with radiotherapy and chemotherapy to provide comprehensive care that addresses both the physical and emotional needs of the patient.  **Response 3:** The main goals of palliative care are to improve the quality of life for patients with advanced or metastatic cervical cancer. Palliative care can be combined with radiotherapy and chemotherapy to help manage symptoms such as pain, fatigue, and nausea. It can also provide emotional and psychological support for patients and their families. | | 94 | 83 | | | | 89 | Response1 |
| QiZhenGPT  (with prompt) | The semantics of all three responses are identical | | - | - | | | | - | - |
| QiZhenGPT  (without prompt) | **Response 1:** In this case, ICRT can be used as an alternative or in addition to conventional radiotherapy. The main difference between ICRT and conventional radiotherapy is that ICRT uses images to guide the radiation dose, while conventional radiotherapy relies on the radiation oncologist's expertise to determine the dose.  **Response 2:** The main goals of palliative care are to improve the quality of life for patients with advanced cancer, and to manage symptoms and side effects. Palliative care can be combined with radiotherapy and chemotherapy in patients with metastatic cervical cancer by providing support and management of symptoms, and by helping to ensure that treatment is tailored to the individual needs of the patient.  **Response 3:** The main goals of palliative care are to improve the quality of life for patients with advanced cancer, and to manage symptoms and side effects. Palliative care can be combined with radiotherapy and chemotherapy in patients with metastatic cervical cancer by providing support and management of symptoms, and by helping to ensure that treatment is tailored to the individual needs of the patient. | | 0 | 56 | | | | 100 | Response3 |
