# Supplementary material for: Performance Evaluation of Large Language Models in Cervical Cancer Management Based on a Standardized Questionnaire: Comparative Study
Source: J Med Internet Res. 2025 Feb 5;27:e63626. doi: 10.2196/63626 (PMC11840365; doi:10.2196/63626)
Supplement: Multimedia Appendix 4 [file jmir_v27i1e63626_app4.docx]

**Multimedia Appendix 4.** Responses from each model and quality assessments

| **Models** | **Response** | **Assessments** | | |
| --- | --- | --- | --- | --- |
|  |  | **Reviewe1** | **Reviewer**  **2** | **Final** |
| **Question 1: What is the examination process for HPV-positive high-risk types?** | | | | |

| ChatGPT4.0 turbo (With prompt) | 1. **Risk Factors for Cervical Cancer Screening**: The primary risk factors that necessitate cervical cancer screening include early onset of sexual activity, having multiple sexual partners, a history of sexually transmitted infections (STIs), particularly human papillomavirus (HPV), long-term use of oral contraceptives, smoking, and having a weakened immune system. Additionally, individuals with a history of cervical dysplasia or those who have not been vaccinated against HPV are also considered at higher risk and should adhere to regular screening schedules. | A | A | A |
| --- | --- | --- | --- | --- |
| ChatGPT4.0 turbo | 1. **Risk Factors for Cervical Cancer Screening**:  - **Human Papillomavirus (HPV) Infection**: The primary risk factor for cervical cancer is persistent infection with high-risk strains of HPV, particularly HPV types 16 and 18.  - **Sexual History**: Early sexual activity, multiple sexual partners, and having partners who have had multiple partners can increase the risk of HPV infection.  - **Smoking**: Tobacco use is associated with an increased risk of cervical cancer.  - **Immunosuppression**: Individuals with weakened immune systems, including those with HIV/AIDS or those taking immunosuppressive drugs, are at higher risk.  - **Long-term use of oral contraceptives**: Research suggests that using oral contraceptives for a long time may be associated with a slightly increased risk.  - **Other sexually transmitted infections (STIs)**: Having other STIs such as chlamydia, gonorrhea, syphilis, or herpes simplex virus can increase the risk.  - **Family history**: Having a first-degree relative with cervical cancer can increase risk. | A | A | A |
| Claude 2 (With prompt) | 1. The main risk factors necessitating regular cervical screening include infection with high-risk strains of human papillomavirus (HPV), early onset of sexual activity, multiple sexual partners, smoking, HIV infection or other causes of immunosuppression, and previous abnormal cervical cytology results. | A | A | A |
| Claude 2 | 1. Risk factors that may necessitate screening include infection with high-risk HPV strains, early sexual activity, multiple sexual partners, smoking, HIV infection, chlamydia infection, family history of cervical cancer, and previous abnormal Pap tests. | A | A | A |
| Gemini Pro (With prompt) | 1. What are the risk factors that may necessitate cervical cancer screening？  Several risk factors can increase a woman's risk of developing cervical cancer, necessitating regular screening:   Human papillomavirus (HPV) infection: HPV is the primary cause of cervical cancer, and certain HPV types are considered high-risk.  Early age of first sexual intercourse: Starting sexual activity at a young age increases the risk of exposure to HPV.  Multiple sexual partners: Having multiple sexual partners increases the risk of exposure to different HPV strains.  Weakened immune system: Individuals with weakened immune systems, such as those with HIV/AIDS, are more susceptible to HPV infection and cervical cancer.  Smoking: Smoking weakens the immune system and increases the risk of developing cervical cancer.  Long-term use of oral contraceptives: Some studies suggest a slightly increased risk of cervical cancer with long-term use of oral contraceptives.  Family history of cervical cancer: A family history of cervical cancer may indicate an increased genetic predisposition. | A | B | A |
| Gemini Pro | 1. Risk factors that may necessitate cervical cancer screening include:  - Age: Women between the ages of 21 to 65 are typically recommended for regular screening.  - Sexual activity: Women who are sexually active, particularly those with multiple partners or those who started having sex at a young age, are at higher risk.  - HPV infection: HPV is a major risk factor for cervical cancer. Women who have abnormal Pap test results or those with certain types of HPV infections may need more frequent screening.  - Smoking: Smoking increases the risk of cervical cancer.  - Immune system suppression: Women with weakened immune systems due to diseases such as HIV/AIDS or organ transplant are at higher risk. | B | B | B |
| Mistral-7B-v0.2 (With prompt) | 1. Risk factors for cervical cancer screening include: a) History of cervical dysplasia or precancerous lesions, b) Human papillomavirus (HPV) infection with high-risk types, c) Immunosuppression, d) Smoking, e) Multiple sexual partners, f) Early onset of sexual activity, g) Long-term use of oral contraceptives, and h) Family history of cervical or other related cancers. | A | A | A |
| Mistral-7B-v0.2 | 1. Risk factors that may necessitate cervical cancer screening include:  a. Sexual activity at a young age  b. Multiple sexual partners  c. Smoking  d. Human Papillomavirus (HPV) infection, which is a sexually transmitted infection  e. A weakened immune system from HIV or organ transplant medicines  f. Use of birth control pills for more than five years  g. Chlamydia or gonorrhea infections  h. Abnormal Pap test results  i. Exposure to diethylstilbestrol (DES) in the womb | A | A | A |
| Starling-LM-7B alpha (With prompt) | 1. Risk factors that may necessitate cervical cancer screening include:   a. Age: Women aged 21 to 65 years are generally recommended for screening, although the specific age range may vary depending on guidelines from different organizations.    b. History of HPV infection: Women with a history of high-risk HPV infection are at a higher risk of developing cervical cancer and may require more frequent screening.    c. Immunosuppression: Women with weakened immune systems, such as those with HIV/AIDS or organ transplant recipients, may require more frequent screening due to an increased risk of developing cervical cancer.    d. Smoking: Smoking is a known risk factor for cervical cancer, and women who smoke may require more frequent screening.    e. Family history: Women with a family history of cervical cancer may require more frequent screening. | A | B | A |
| Starling-LM-7B alpha | 1. Risk factors that may necessitate cervical cancer screening include:   - A history of previous cervical abnormalities or precancerous conditions.  - A family history of cervical cancer.  - A history of smoking, which increases the risk of cervical cancer.  - A weakened immune system, such as from HIV/AIDS or organ transplant medications.  - Long-term use of oral contraceptives.  - Exposure to diethylstilbestrol (DES) in utero, a hormone that was prescribed to some pregnant women in the mid-20th century. | A | A | A |
| HuatuoGPT (With prompt) | 1. There are a number of risk factors that may contribute to the need for cervical cancer screening, including: being sexually active, multiple sexual partners, previous HPV infection, previous cervical cancer or pre-cancerous lesions, a family history of cervical cancer, and a compromised immune system. | A | A | A |
| HuatuoGPT | 1. Risk factors for cervical cancer screening include: sexual activity younger than 15 years of age, previous cervical cancer or pre-cancerous lesions, and human papillomavirus (HPV) type 16 infection. Cervical cancer screening may also be considered in the following cases: HIV infection, immune system suppression, use of oral contraceptives for more than 10 years, and multiple sexual partners. | A | A | A |
| BioMedLM 2.7B (With prompt) | 1.The risk factors for cervical cancer include age, sexual activity, smoking, and human papillomavirus (HPV) infection. | B | B | B |
| BioMedLM 2.7B | 1.Age: Personal history of cervical cancer: Personal history of cervical dysplasia: Personal history of genital warts: 5Personal history of smoking: Personal history of alcohol consumption: Personal history of oral contraceptive use: Personal history of hormone replacement therapy: 9Personal history of sexually transmitted diseases: Personal history of human immunodeficiency virus (HIV): Personal history of chronic hepatitis B or C: Personal history of chronic kidney disease: Personal history of diabetes mellitus: Personal history of autoimmune disease: 5Personal history of chronic liver disease: Personal history of chronic pancreatitis: Personal history of chronic inflammatory bowel disease: Personal history of chronic obstructive pulmonary disease: 9Personal history of chronic hypertension: Personal history of obesity: Personal history of nulliparity: Personal history of multiparity: Personal history of infertility: Personal history of preterm birth: 5Personal history of preterm premature rupture of membranes: 6Personal history of multiple sexual partners: Personal history of sexually transmitted infections: Personal history of sexually transmitted diseases other than HIV: Personal history of human papilloma virus (HPV): Personal history of human immunodeficiency virus (HIV): Personal history of chronic hepatitis B or C: Personal history of chronic kidney disease: Personal history of diabetes mellitus | B | A | B |
| Microsoft Phi2 (With prompt) | 1. Risk factors that may necessitate cervical cancer screening include a history of human papillomavirus (HPV) infection, a weakened immune system, a history of sexually transmitted infections (STIs), a history of smoking, and a family history of cervical cancer. | A | A | A |
| Microsoft Phi2 | 1. Risk factors that may necessitate cervical cancer screening include a history of abnormal Pap tests, a history of sexually transmitted infections, a weakened immune system, and a family history of cervical cancer. | B | B | B |
| QiZhenGPT (With prompt) | 1. Risk factors for cervical cancer include: * Pregnancy * Condom use * Age under 35 * Human Immunodeficiency Virus (HIV) infection * Human Immunodeficiency Virus-related infection (HIV-RNA positive) * history of sexual violence * history of sexual abuse * history of cancer in a family member * history of a family history of cervical cancer | C | B | C |
| QiZhenGPT | 1. Risk factors for cervical cancer include: * Condom use history * Human Immunodeficiency Virus (HIV) infection * Pregnancy * age * family history of cervical cancer | B | C | C |
| **2.At what age or under what conditions is cervical cancer screening typically deemed unnecessary?** | | | | |
| ChatGPT4.0 turbo (With prompt) | 2. **Age or Conditions Where Screening May Be Unnecessary**: Cervical cancer screening is typically deemed unnecessary before the age of 21, regardless of sexual activity. After the age of 65, women who have had adequate prior screening and are not otherwise at high risk for cervical cancer (i.e., those who have had three consecutive negative Pap test results or two consecutive negative HPV test results within the last 10 years, with the most recent test performed within the last 5 years) may discontinue screening. Additionally, women who have undergone a total hysterectomy for reasons not related to cervical cancer or its precursors do not need to continue cervical cancer screening. | A | A | A |
| ChatGPT4.0 turbo | 2. **Age or Conditions Where Screening May Be Unnecessary**:  - **Age under 21**: Cervical cancer screening is not recommended for women under 21 years of age regardless of sexual activity.  - **Age over 65**: Women over the age of 65 who have had adequate prior screening and are not at high risk for cervical cancer may cease screening.  - **Hysterectomy**: Women who have had a total hysterectomy (removal of the uterus and cervix) for reasons not related to cervical cancer or pre-cancer do not need to be screened. | A | A | A |
| Claude 2 (With prompt) | 2. Cervical screening is typically deemed unnecessary before age 21, regardless of age of sexual initiation or other risk factors. After hysterectomy for non-cancer indications, with no history of high-grade pre-cancer (CIN2+) in the past 25 years, screening can safely stop. Otherwise, it may continue to age 65-70 depending on multiple risk factors. | A | B | A |
| Claude 2 | 2. Cervical cancer screening is typically not needed before age 21. It may be deemed unnecessary in women over 65 who have had adequate prior negative screening. | A | A | A |
| Gemini Pro (With prompt) | 2. At what age or under what conditions is cervical cancer screening typically deemed unnecessary?  Cervical cancer screening is typically deemed unnecessary under the following conditions:   Women under 21 years old: The risk of cervical cancer is extremely low in this age group.  Women over 65 years old with adequate prior negative screenings: If a woman has had regular screenings with consistently negative results and is not at high risk for cervical cancer, screening may be discontinued.  Women who have undergone a total hysterectomy with removal of the cervix (radical hysterectomy): The cervix is the site where cervical cancer develops, so its removal eliminates the risk. | A | A | A |
| Gemini Pro | 2. Cervical cancer screening is typically deemed unnecessary for women who have undergone a hysterectomy (removal of the uterus and cervix) for reasons other than cancer, and for women over the age of 65 with a history of normal Pap tests. | B | B | B |
| Mistral-7B-v0.2 (With prompt) | 2. Cervical cancer screening is typically deemed unnecessary after ages 65-70 for women with a normal screening history. Additionally, for women who have undergone a hysterectomy, if the surgical notes include total removal of the cervix, they may no longer need cervical cancer screening. | C | B | B |
| Mistral-7B-v0.2 | 2. Cervical cancer screening is typically deemed unnecessary for individuals who have undergone a hysterectomy for reasons unrelated to cervical cancer, and for women older than 65 with no risk factors and a normal Pap test history. | B | B | B |
| Starling-LM-7B alpha (With prompt) | 2. Cervical cancer screening is typically deemed unnecessary under the following conditions:   a. Age: Women under the age of 21 or over the age of 65 who have had regular, negative screening results in the past several years may not require further screening.    b. Certain medical conditions: Women with severe medical conditions that make it difficult to perform a pelvic exam or undergo treatment for cervical cancer may not require screening.    c. Life expectancy: Women with a short life expectancy due to advanced age or severe illness may not require screening. | A | A | A |
| Starling-LM-7B alpha | 2. Cervical cancer screening is typically deemed unnecessary for:   - Individuals who have undergone a successful hysterectomy (removal of the uterus and cervix) due to non-cancerous conditions, as long as the cervix was not affected by the condition.  - Postmenopausal women with no history of abnormal cervical cells or precancerous conditions, and who have not been exposed to risk factors. | C | B | C |
| HuatuoGPT (With prompt) | 2. Cervical cancer screening is usually considered unnecessary at the following ages or conditions: 65 years and older, regular cervical cancer screening, no abnormal results in the past 10 years, and no HPV vaccination. | B | B | B |
| HuatuoGPT | 2. Cervical cancer screening is usually not recommended for women older than 65 years of age if they have had a normal cervical cytology or three consecutive previous tests with normal results, or in average-risk patients who have had a hysterectomy and are older than 50 years of age and have no prior history of cervical cancer or abnormal cytology results. . | A | A | A |
| BioMedLM 2.7B (With prompt) | 2.At what age or under what conditions is cervical cancer screening typically deemed unnecessary? The age at which cervical cancer screening is typically deemed unnecessary is controversial. The American College of Obstetricians and Gynecologists (ACOG) recommends that cervical cancer screening be initiated at age 21 years, and that screening be discontinued at age 65 years.^[@bib1]^ The American Cancer Society (ACS) recommends that cervical cancer screening be initiated at age 21 years, and that screening be discontinued at age 65 years.^[@bib2]^ The US Preventive Services Task Force (USPSTF) recommends that cervical cancer screening be initiated at age 21 years, and that screening be discontinued at age 65 years.^[@bib3]^ The American Society for Colposcopy and Cervical Pathology (ASCCP) recommends that cervical cancer screening be initiated at age 21 years, and that screening be discontinued at age 65 years.^[@bib4]^ The American Society for Clinical Pathology (ASCP) recommends that cervical cancer screening be initiated at age 21 years, and that screening be discontinued at age 65 years.^[@bib5]^ The American Society for Clinical Oncology (ASCO) recommends that cervical cancer screening be initiated at age 21 years, and that screening be discontinued at age 65 years.^[@bib6]^ The American Society of Clinical Oncology (ASCO) recommends that cervical cancer screening be initiated at age 21 years, and that screening be discontinued at age 65 years. | B | B | B |
| BioMedLM 2.7B | 2.At age 21 or older. | D | D | D |
| Microsoft Phi2 (With prompt) | 2. Cervical cancer screening is typically deemed unnecessary for individuals who are over the age of 65 and have had three consecutive normal Pap tests. | B | B | B |
| Microsoft Phi2 | 2. Cervical cancer screening may be deemed unnecessary for individuals who are over the age of 65, have had a hysterectomy, or have had a negative Pap test in the past 10 years. | B | B | B |
| QiZhenGPT (With prompt) | 2. Cervical cancer screening is typically deemed unnecessary in: * Women who have never been pregnant or have never had a menstrual period * Women who have a low-risk profile (age 35 or older, no history of sexual violence or abuse, no history of cancer in a family member, no history of HIV infection) * Women who have a history of sexual violence or abuse * Women who have a history of cancer in a family member * Women who are at high risk for developing cervical cancer due to their age, history of sexual violence or abuse, or a family history of cancer | D | C | C |
| QiZhenGPT | 2. cervical cancer screening is considered unnecessary in the following situations: * Age 16-19 * Pregnancy or planning to become pregnant * Condom use history for more than 1 year * Human Immunodeficiency Virus (HIV) infection * Pregnancy or planning to become pregnant * Age 50 or older | D | D | D |
| **3.What strategies are effective in reducing the risk of developing cervical cancer?** | | | | |
| ChatGPT4.0 turbo (With prompt) | 3. **Strategies to Reduce Cervical Cancer Risk**: Effective strategies for reducing the risk of developing cervical cancer include vaccination against HPV for both females and males, ideally before the onset of sexual activity. Regular cervical cancer screening (Pap tests and HPV testing) as recommended helps in the early detection and treatment of precancerous conditions. Limiting the number of sexual partners, using barrier protection during sexual intercourse, and avoiding tobacco use also significantly decrease risk. | A | A | A |
| ChatGPT4.0 turbo | 3. **Strategies to Reduce Risk of Cervical Cancer**:  - **HPV Vaccination**: Vaccination against HPV is one of the most effective methods to prevent cervical cancer.  - **Regular Screening**: Participating in regular screening programs (Pap tests and HPV tests) can detect pre-cancerous changes, which can be treated before they turn into cancer.  - **Safe Sexual Practices**: Limiting the number of sexual partners and using barrier protection methods such as condoms.  - **Smoking Cessation**: Quitting smoking reduces the risk of cervical as well as other types of cancer.  - **Prompt Treatment of Pre-cancerous Conditions**: Proper management of any cervical abnormalities detected during screening. | A | A | A |
| Claude 2 (With prompt) | 3. Effective cervical cancer prevention strategies include HPV vaccination prior to HPV exposure, practicing safe sexual habits to avoid high-risk HPV infection, smoking cessation, and avoiding immunosuppression when possible. The HPV vaccine protects against the strains responsible for about 90% of cervical cancers. | A | B | A |
| Claude 2 | 3. Effective strategies include HPV vaccination, safe sexual practices, not smoking, and getting regular Pap tests. | B | B | B |
| Gemini Pro (With prompt) | 3. What strategies are effective in reducing the risk of developing cervical cancer?  Several strategies can effectively reduce the risk of developing cervical cancer:   HPV vaccination: The HPV vaccine is highly effective in preventing infection with the high-risk HPV types that cause most cervical cancers. Vaccination is recommended for girls and boys starting at age 11 or 12.  Regular cervical cancer screening: Regular screening allows for early detection and treatment of precancerous lesions, preventing them from developing into cancer.  Safer sex practices: Using condoms consistently and limiting the number of sexual partners can reduce the risk of HPV infection.  Smoking cessation: Quitting smoking significantly reduces the risk of cervical cancer and other health problems.  Maintaining a healthy immune system: Eating a healthy diet, exercising regularly, and getting enough sleep can help boost the immune system and reduce the risk of HPV infection. | A | A | A |
| Gemini Pro | 3. Strategies effective in reducing the risk of developing cervical cancer include:  - HPV vaccination: The HPV vaccine can protect against certain types of HPV that cause most cervical cancers.  - Regular screening: Regular Pap tests and HPV tests can help detect and prevent cervical cancer.  - Healthy lifestyle: Avoiding smoking, using a condom during intercourse, and maintaining a healthy diet and weight can also reduce the risk. | A | A | A |
| Mistral-7B-v0.2 (With prompt) | 3. Strategies effective in reducing the risk of developing cervical cancer include: a) Early initiation of sexual activity, b) Limiting sexual partners, c) Using condoms during intercourse, d) Avoiding smoking, e) Quitting smoking if currently a smoker, f) Maintaining a healthy diet, g) Engaging in regular physical activity, h) Getting vaccinated against HPV before becoming sexually active, and i) Getting regular cervical cancer screenings. | C | B | B |
| Mistral-7B-v0.2 | 3. Effective strategies in reducing the risk of developing cervical cancer include:  a. HPV vaccination  b. Regular cervical cancer screening (Pap test and HPV test)  c. Limiting the number of sexual partners  d. Using condoms during sexual activity  e. Quitting smoking  f. Maintaining a healthy diet | A | A | A |
| Starling-LM-7B alpha (With prompt) | 3. Strategies effective in reducing the risk of developing cervical cancer include:   a. Vaccination: HPV vaccination can help protect against the high-risk HPV strains that cause most cervical cancers. The vaccine is recommended for preteen girls and boys aged 11 to 12 years, with catch-up vaccination for those aged 13 to 26 years.    b. Regular screening: Regular cervical cancer screening, such as Pap smears and HPV testing, can help detect precancerous changes and allow for early intervention.    c. Safe sex practices: Using condoms and practicing safe sex can help reduce the risk of HPV infection, which is a major risk factor for cervical cancer.    d. Quitting smoking: Smoking is a known risk factor for cervical cancer, and quitting smoking can help reduce the risk. | A | A | A |
| Starling-LM-7B alpha | 3. Strategies effective in reducing the risk of developing cervical cancer include:   - Regular cervical cancer screening, such as Pap tests and HPV tests, to detect and treat precancerous conditions early.  - HPV vaccination for both males and females, ideally before they become sexually active. The vaccine protects against the HPV strains most commonly associated with cervical cancer.  - Practicing safe sex, using condoms, and limiting the number of sexual partners to reduce the risk of HPV infection.  - Not smoking, as it increases the risk of cervical cancer. | A | B | A |
| HuatuoGPT (With prompt) | 3. Effective strategies to reduce the risk of cervical cancer include: HPV vaccination, use of condoms, avoidance of multiple sexual partners, regular cervical cancer screening, regular gynecological checkups, and prompt treatment of cervical lesions. | A | A | A |
| HuatuoGPT | 3. Effective strategies to reduce the risk of cervical cancer include: HPV vaccination, sex with multiple partners, smoking, and female hormone replacement therapy (menopausal hormone therapy). Other factors that increase the risk of cervical cancer include: younger age at sexual debut, lack of cervical cancer screening, and immunosuppression. | C | C | C |
| BioMedLM 2.7B (With prompt) | 3. What strategies are effective in reducing the risk of developing cervical cancer? The risk of developing cervical cancer is reduced by screening and vaccination. The most effective screening method is the Papanicolaou (Pap) test, which has been shown to reduce the risk of cervical cancer by up to 80%.[@bib1] The Pap test is a simple, inexpensive, and noninvasive test that can be performed by a primary care provider. The American Cancer Society recommends that women aged 21 to 65 years be screened every 3 years with a Pap test.[@bib2] The Centers for Disease Control and Prevention (CDC) recommends that women aged 21 to 65 years be screened every 3 years with a Pap test and that women aged 30 to 65 years be screened every 5 years with a human papillomavirus (HPV) test.[@bib3] The HPV test is a more sensitive screening method than the Pap test, but it is more expensive and requires a more complex laboratory procedure.[@bib4] | B | B | B |
| BioMedLM 2.7B | 3. a\Vaccination against HPV b\ Screening for cervical cancer c\ HPV vaccination and screening | B | B | B |
| Microsoft Phi2 (With prompt) | 3. Strategies that are effective in reducing the risk of developing cervical cancer include practicing safe sex, getting vaccinated against HPV, quitting smoking, and maintaining a healthy diet and exercise routine. | B | B | B |
| Microsoft Phi2 | 3. Strategies that are effective in reducing the risk of developing cervical cancer include practicing safe sex, getting vaccinated against HPV, and undergoing regular cervical cancer screening. | B | B | B |
| QiZhenGPT (With prompt) | 3. Strategies to reduce the risk of developing cervical cancer include: * Condom use * Pregnancy prevention * High-risk behavior prevention (such as sexual activity with a partner who has HIV or is HIV-RNA positive) * Pregnancy prevention through early detection and prenatal care * Pregnancy prevention through education and awareness campaigns | B | C | B |
| QiZhenGPT | 3. Strategies to reduce the risk of developing cervical cancer include: * Condom use history for more than 1 year * Human Immunodeficiency Virus (HIV) infection * Pregnancy * Age 16-19 * Family history of cervical cancer | C | C | C |
| **4.What are the common clinical symptoms of cervical cancer?** | | | | |
| ChatGPT4.0 turbo (With prompt) | 4. **Common Clinical Symptoms of Cervical Cancer**: In its early stages, cervical cancer may not present any symptoms. However, as the disease progresses, common symptoms may include abnormal vaginal bleeding (such as bleeding after sexual intercourse, between menstrual periods, or after menopause), unusual vaginal discharge, pelvic pain, and pain during intercourse. These symptoms warrant prompt evaluation by a healthcare provider. | A | A | A |
| ChatGPT4.0 turbo | 4. **Common Clinical Symptoms of Cervical Cancer**:  - **Abnormal Vaginal Bleeding**: Including bleeding after sexual intercourse, between periods, or after menopause.  - **Unusual Vaginal Discharge**: A watery, pink, or foul-smelling discharge is common.  - **Pelvic Pain**: Pain during intercourse or at other times may be a symptom of advanced disease.  - **Other Symptoms**: Such as urinary difficulties, leg pain, or swelling may occur with more advanced cancer. | A | A | A |
| Claude 2 (With prompt) | 4. Symptoms of invasive cervical cancer can include postcoital (after intercourse) bleeding, bloody vaginal discharge between periods or post-menopause, pain during intercourse, and pelvic or back pain. However, precancerous changes and early invasive cancers are usually asymptomatic. This demonstrates the importance of regular screening. | B | A | A |
| Claude 2 | 4. Common symptoms include abnormal vaginal bleeding or discharge, pelvic/back pain, pain during sex, and bleeding after menopause. However, early cervical cancer often has no symptoms. | A | A | A |
| Gemini Pro (With prompt) | 4. What are the common clinical symptoms of cervical cancer?  In the early stages, cervical cancer often presents with no symptoms. However, as the cancer progresses, the following symptoms may develop:   Abnormal vaginal bleeding: This may include bleeding between periods, after menopause, or heavier or longer periods than usual.  Unusual vaginal discharge: The discharge may be watery, bloody, or have a foul odor.  Pain during intercourse: This may be a sign that the cancer has spread to nearby tissues.  Pelvic pain: This may be a dull ache or a sharp pain in the lower abdomen.  Fatigue: This is a common symptom of many types of cancer, including cervical cancer.  Unexplained weight loss: This may also be a sign of advanced cancer. | A | A | A |
| Gemini Pro | 4. Common clinical symptoms of cervical cancer include:  - Abnormal vaginal bleeding: This can occur after sex, between periods, or after menopause.  - Pelvic pain: This can be a dull or sharp pain in the pelvis or lower back.  - Pain during sex.  - Hydronephrosis: This is a swelling of the kidneys, which can occur when cervical cancer blocks the flow of urine. | B | C | C |
| Mistral-7B-v0.2 (With prompt) | 4. Common clinical symptoms of cervical cancer include: a) Abnormal vaginal bleeding or discharge, b) Pain during sexual intercourse, c) Pelvic pain, and d) Pain in the back or legs. However, note that many women with early-stage cervical cancer may not experience symptoms. | A | A | A |
| Mistral-7B-v0.2 | 4. Common clinical symptoms of cervical cancer include:  a. Abnormal vaginal bleeding, especially postmenopausal  b. Pain during sexual intercourse  c. Abnormal vaginal discharge, often watery, thick, or tinged with blood  d. Pain in the pelvic area  e. Increased frequency of urination  f. Difficulty emptying the bladder | B | C | C |
| Starling-LM-7B alpha (With prompt) | 4. Common clinical symptoms of cervical cancer include:   a. Abnormal vaginal bleeding: This may occur after intercourse, between periods, or after menopause.    b. Pain during intercourse: This may be due to changes in the cervix caused by cancer.    c. Pelvic pain: This may be due to the growth of the tumor or the spread of cancer to nearby tissues.    d. Painful urination or frequent urination: This may be due to the growth of the tumor or the spread of cancer to the bladder.    e. Constipation or difficulty passing stool: This may be due to the growth of the tumor or the spread of cancer to the rectum. | C | B | C |
| Starling-LM-7B alpha | 4. Common clinical symptoms of cervical cancer include:   - Abnormal vaginal bleeding, such as bleeding between periods, after sexual intercourse, or after menopause.  - Pain during sexual intercourse.  - Pelvic pain or pain in the back and legs.  - Increased urinary frequency or urgency. | B | C | B |
| HuatuoGPT (With prompt) | 4. Common symptoms of cervical cancer include: unusual vaginal bleeding (e.g. bleeding between periods, bleeding after sexual intercourse, etc.), abnormal vaginal discharge, pelvic pain, frequent or urgent urination. | B | C | C |
| HuatuoGPT | 4. Common symptoms of cervical cancer include abnormal vaginal bleeding, bleeding during menstruation or after sexual intercourse, and pelvic pain. However, early-stage cervical cancer usually has no obvious symptoms. Therefore, regular cervical cancer screening is very important for early detection and treatment. | A | A | A |
| BioMedLM 2.7B (With prompt) | 4. What are the common clinical symptoms of cervical cancer? Cervical cancer is a common gynecological malignancy. The most common symptoms of cervical cancer are abnormal vaginal bleeding, vaginal discharge, and pelvic pain. | C | C | C |
| BioMedLM 2.7B | 4. Cervical cancer is a common gynecological malignancy. The most common symptoms of cervical cancer are vaginal bleeding, vaginal discharge, and pelvic pain. | A | A | A |
| Microsoft Phi2 (With prompt) | 4. Common clinical symptoms of cervical cancer include abnormal vaginal bleeding, pelvic pain, and pain during sexual intercourse. | C | C | C |
| Microsoft Phi2 | 4. Common clinical symptoms of cervical cancer include abnormal vaginal bleeding, pelvic pain, and pain during intercourse. | C | C | C |
| QiZhenGPT (With prompt) | 4. Common clinical symptoms of cervical cancer include: * Cervical cancer symptoms can be subtle and may not be recognized until the disease is advanced * Persistent or recurrent sore throat * Persistent or recurrent cough * Persistent or recurrent fever * Persistent or recurrent weight loss | A | A | A |
| QiZhenGPT | 4. Clinical symptoms of cervical cancer include: * Cervical cancer can cause a range of symptoms, including: * A persistent, heavy menstrual flow * A persistent, heavy, or light spotting or bleeding * A low-grade fever * A cough * A sore throat * A runny nose | C | C | C |
| **5.For individuals who have been vaccinated against HPV, is it still necessary for them to undergo cervical cancer screening?** | | | | |
| ChatGPT4.0 turbo (With prompt) | 5. **Screening Necessity Post-HPV Vaccination**: Yes, individuals who have been vaccinated against HPV should still undergo cervical cancer screening. While the HPV vaccine significantly reduces the risk of developing cervical cancer by protecting against the most cancer-causing strains of HPV, it does not eliminate the risk entirely. The vaccine does not protect against all cancer-causing types of HPV, and thus regular screening remains crucial to detect cervical changes that might lead to cancer. | A | A | A |
| ChatGPT4.0 turbo | 5. **Cervical Cancer Screening for Vaccinated Individuals**:  - Yes, it is still necessary for individuals vaccinated against HPV to undergo cervical cancer screening. While the vaccine significantly reduces the risk of developing cervical cancer by protecting against the most common cancer-causing strains of HPV, it does not provide complete protection against all cancer-causing HPV types. Regular screening remains crucial to detect cervical abnormalities that could develop into cancer from other HPV strains not covered by the vaccine. | A | A | A |
| Claude 2 (With prompt) | 5. Yes, those vaccinated against HPV still require cervical cancer screening since the vaccines do not provide complete protection against all cancer-causing HPV types. Screening allows for the detection and treatment of any precancerous changes before possible progression to invasive cancer. | A | A | A |
| Claude 2 | 5. Yes, even if vaccinated, guidelines still recommend routine cervical cancer screening starting at age 21. The vaccine protects against the HPV strains most linked to cervical cancer, but does not cover all cancer-causing HPV types. Here are brief answers to your questions on cervical cancer screening recommendations: | A | A | A |
| Gemini Pro (With prompt) | 5. For individuals who have been vaccinated against HPV, is it still necessary for them to undergo cervical cancer screening?  Yes, even for individuals who have been vaccinated against HPV, it is still necessary to undergo cervical cancer screening.   The HPV vaccine does not protect against all types of HPV that can cause cervical cancer.  The vaccine's effectiveness may decrease over time.  The vaccine does not prevent cervical cancer caused by other risk factors.  Therefore, regular cervical cancer screening remains crucial for all women, regardless of their vaccination status. | A | A | A |
| Gemini Pro | 5. Yes, individuals who have been vaccinated against HPV still need to undergo cervical cancer screening. The HPV vaccine protects against certain types of HPV infections, but it does not protect against all types that can cause cervical cancer. Regular cervical cancer screening is necessary to detect any pre-cancerous cells that may develop. | A | A | A |
| Mistral-7B-v0.2 (With prompt) | 5. For individuals who have been vaccinated against HPV, it is still necessary for them to undergo cervical cancer screening. The vaccine protects against the majority of HPV types that cause cervical cancer but does not provide 100% protection. Also, it takes several years for the immune response generated by the vaccine to take full effect. Therefore, regular cervical cancer screenings are still necessary after vaccination. | A | A | A |
| Mistral-7B-v0.2 | 5. Individuals who have been vaccinated against HPV should still undergo regular cervical cancer screening because the vaccine does not protect against all types of HPV that can cause cervical cancer and does not provide protection for life. Screening is necessary to ensure the HPV infection has not developed into cancer and to detect any potential abnormalities. The frequency and type of screening may change based on vaccination status, so consult healthcare professionals for specific recommendations. | A | A | A |
| Starling-LM-7B alpha (With prompt) | 5. For individuals who have been vaccinated against HPV, it is still necessary for them to undergo cervical cancer screening, as the vaccine does not provide 100% protection against all HPV strains or cervical cancer. Regular screening is essential to detect any precancerous changes or early signs of cancer. The specific screening recommendations may vary depending on the individual's vaccination history and other risk factors. | A | A | A |
| Starling-LM-7B alpha | 5. For individuals who have been vaccinated against HPV, it is still necessary for them to undergo cervical cancer screening. The HPV vaccine does not protect against all HPV strains that can cause cervical cancer, and it does not guarantee that a person will not develop cervical cancer. Regular screening is essential to detect and treat any precancerous conditions early.  It's important to note that while the HPV vaccine can reduce the risk of developing cervical cancer, it does not eliminate the risk entirely. Regular screening is still necessary to detect and treat any precancerous conditions early. | A | A | A |
| HuatuoGPT (With prompt) | 5. People who have been vaccinated against HPV still need to be screened for cervical cancer because the vaccine does not protect against all types of HPV that cause cervical cancer. Cervical cancer screening can help detect and treat cervical cancer early. | A | A | A |
| HuatuoGPT | 5. Cervical cancer screening is still necessary for those who have received the HPV vaccine. This is because although the vaccine can prevent the most common cancer-causing types of HPV infections, it does not prevent all types of HPV infections and the development of cervical cancer. So even after vaccination, cervical cancer screening is still needed based on the recommended age. | A | A | A |
| BioMedLM 2.7B (With prompt) | 5. For individuals who have been vaccinated against HPV, is it still necessary for them to undergo cervical cancer screening? Yes, it is still necessary for women who have been vaccinated against HPV to undergo cervical cancer screening. | A | A | A |
| BioMedLM 2.7B | 5. Yes. | A | A | A |
| Microsoft Phi2 (With prompt) | 5. For individuals who have been vaccinated against HPV, it is still necessary for them to undergo cervical cancer screening, as the vaccine does not provide 100% protection against all types of HPV that can cause cervical cancer. | A | A | A |
| Microsoft Phi2 | 5. Even for individuals who have been vaccinated against HPV, it is still necessary for them to undergo cervical cancer screening, as the vaccine does not provide 100% protection against all strains of HPV. | A | A | A |
| QiZhenGPT (With prompt) | 5. For individuals who have been vaccinated against HPV, it is generally not necessary for them to undergo cervical cancer screening. However, it is important to follow the recommended schedule and interval for the vaccine to be administered, as well as to continue practicing good sexual health habits to reduce the risk of HPV-related cervical cancer. | D | D | D |
| QiZhenGPT | 5. For individuals who have been vaccinated against HPV, it is still necessary for them to undergo cervical cancer screening, as the vaccine does not provide complete protection against cervical cancer. Regular screening with a cervical test can detect pre-cancerous cells and prevent the development of cervical cancer. | B | A | B |
| **6.Is cervical cancer screening still recommended for individuals who have had only one sexual partner or are not currently sexually active?** | | | | |
| ChatGPT4.0 turbo (With prompt) | 6. **Cervical Cancer Screening for Individuals with Limited Sexual Activity**: Yes, cervical cancer screening is still recommended for individuals who have had only one sexual partner or are not currently sexually active. The rationale is that HPV, the primary cause of cervical cancer, can be transmitted through a single sexual encounter and can remain dormant for years before causing cellular changes that may lead to cancer. Therefore, adherence to recommended screening guidelines is crucial for early detection and intervention, regardless of sexual history. | A | A | A |
| ChatGPT4.0 turbo | 6. **Cervical Cancer Screening for Individuals with Limited Sexual History**:  - Yes, cervical cancer screening is still recommended for individuals who have had only one sexual partner or are not currently sexually active, provided they are within the recommended age range for screening (21-65 years). This is because HPV can be transmitted through even a single sexual encounter, and the virus can remain dormant for years before potentially causing cervical cancer. | A | A | A |
| Claude 2 (With prompt) | 6. Yes, cervical cancer screening is still recommended even for those with one lifetime sex partner or who are not currently sexually active. HPV can be transmitted through even one unprotected sexual encounter, underscoring the importance of at least baseline screening. | A | A | A |
| Claude 2 | 6. Yes, cervical cancer screening is still recommended even for those with one lifetime sex partner or who are not currently sexually active. The HPV virus that causes cervical cancer can be transmitted through any unprotected sexual contact. | A | A | A |
| Gemini Pro (With prompt) | 6. Is cervical cancer screening still recommended for individuals who have had only one sexual partner or are not currently sexually active?  Yes, cervical cancer screening is still recommended for individuals who have had only one sexual partner or are not currently sexually active. Though the risk of cervical cancer may be lower in these individuals, it is not eliminated.   The human papillomavirus (HPV), the primary cause of cervical cancer, can remain dormant for years before causing any symptoms. Therefore, regular screening is crucial for early detection and treatment, even for individuals with limited sexual activity. | A | A | A |
| Gemini Pro | 6. Yes, cervical cancer screening is still recommended for individuals who have had only one sexual partner or are not currently sexually active. HPV infection can occur even in individuals with no or few sexual partners, and cervical cancer is a potential long-term complication of HPV infection. | A | A | A |
| Mistral-7B-v0.2 (With prompt) | 6. Yes, cervical cancer screening is still recommended for individuals who have had only one sexual partner or are not currently sexually active. HPV, a major cause of cervical cancer, can be acquired during any sexual contact, even without intercourse. Therefore, it is essential to maintain regular screening, even if one's sexual activity is limited or nonexistent. | C | B | C |
| Mistral-7B-v0.2 | 6. Yes, cervical cancer screening is still recommended for individuals who have had only one sexual partner or are not currently sexually active. This is because cervical cancer can develop even in the absence of sexual activity, and screening can help detect precancerous changes that can be treated before they progress to cancer. | A | A | A |
| Starling-LM-7B alpha (With prompt) | 6. While cervical cancer screening is generally recommended for all women, it may not be necessary for individuals who have had only one sexual partner or are not currently sexually active. This is because the risk of cervical cancer is primarily associated with the human papillomavirus (HPV), which is transmitted through sexual activity. However, it is important to note that cervical cancer screening guidelines may vary depending on the specific organization or country, so it is essential to follow the recommendations provided by local health authorities. | D | C | C |
| Starling-LM-7B alpha | 6. Cervical cancer screening is still recommended for individuals who have had only one sexual partner or are not currently sexually active. This is because cervical cancer can develop in women who have never been sexually active, and regular screening is essential for early detection and prevention. | A | A | A |
| HuatuoGPT (With prompt) | 6. Cervical cancer screening is recommended even if you have only one sexual partner or are not sexually active. | A | A | A |
| HuatuoGPT | 6. Cervical cancer screening is still recommended even if you have only one sexual partner or do not engage in sexual behavior. The development of cervical cancer is related to a number of factors, not just the number of sexual partners. Even people who have only one sexual partner or do not engage in sexual behavior can still be infected with HPV and develop cervical cancer. Therefore, screening is still recommended for these individuals for early detection and prevention. | A | A | A |
| BioMedLM 2.7B (With prompt) | 6.Is cervical cancer screening still recommended for individuals who have had only one sexual partner or are not currently sexually active? Yes, cervical cancer screening is still recommended for individuals who have had only one sexual partner or are not currently sexually active. The American Cancer Society (ACS) recommends that women aged 21 to 65 years who have had only one sexual partner or are not currently sexually active should be screened for cervical cancer every 3 years. The ACS recommends that women who have had more than one sexual partner or are currently sexually active should be screened for cervical cancer every 5 years. | A | A | A |
| BioMedLM 2.7B | 6. Yes. | B | B | B |
| Microsoft Phi2 (With prompt) | 6. Yes, cervical cancer screening is still recommended for individuals who have had only one sexual partner or are not currently sexually active, as they may still be at risk for developing cervical cancer due to other risk factors such as smoking or a weakened immune system. | A | A | A |
| Microsoft Phi2 | 6. Cervical cancer screening is still recommended for individuals who have had only one sexual partner or are not currently sexually active. | A | A | A |
| QiZhenGPT (With prompt) | 6. Yes, cervical cancer screening is still recommended for individuals who have had only one sexual partner or are not currently sexually active. | A | A | A |
| QiZhenGPT | 6. Yes, cervical cancer screening is still recommended for individuals who have had only one sexual partner or are not currently sexually active. | A | A | A |
| **7.What are the recommended intervals for cervical cancer screening, and do these intervals vary among different age groups?** | | | | |
| ChatGPT4.0 turbo (With prompt) | 7. **Recommended Intervals for Cervical Cancer Screening**: The recommended intervals for cervical cancer screening vary among different age groups:  - **Ages 21-29**: Women should have a Pap test every 3 years.  - **Ages 30-65**: Women should have a Pap test combined with an HPV test (co-testing) every 5 years as the preferred approach; however, it is acceptable to have a Pap test alone every 3 years.  - **Over 65**: Women who have had adequate prior screening and are not at high risk for cervical cancer should stop screening.  These intervals are designed to provide maximum protection while minimizing unnecessary procedures and potential harm from over-screening. | A | A | A |
| ChatGPT4.0 turbo | 7. **Recommended Intervals for Cervical Cancer Screening**:  - **Ages 21-29**: Women should have a Pap test every three years.  - **Ages 30-65**: Women should have a Pap test combined with an HPV test (co-testing) every five years as the preferred approach; alternatively, they can continue to have a Pap test alone every three years.  - **Over Age 65**: Women who have had adequate prior screening and are not otherwise at high risk for cervical cancer should stop screening.  - These intervals are recommended to balance the benefits of early detection with the risks of overtreatment and unnecessary anxiety. | A | A | A |
| Claude 2 (With prompt) | 7. Recommended screening intervals are every 3 years for women aged 21-29, and every 5 years for women aged 30-65 who have had adequate prior negative screening history. More frequent screening is NOT recommended at any age due to increased likelihood of false positive results without additional cancer detection benefit. | A | B | A |
| Claude 2 | 7. For ages 21-29, Pap testing every 3 years is recommended. For ages 30-65, Pap plus HPV testing every 5 years or Pap testing every 3 years is recommended. Screening is not recommended routinely for those over age 65 with adequate prior negative screening history. | A | A | A |
| Gemini Pro (With prompt) | 7. What are the recommended intervals for cervical cancer screening, and do these intervals vary among different age groups?  The recommended intervals for cervical cancer screening vary depending on age and other factors. Here's a breakdown:   Women aged 21-29:   Pap test every 3 years.  Women aged 30-65:  Pap test every 3 years or a co-test (Pap and HPV test) every 5 years.  Women aged 65 and older:  Screening may be discontinued if they have had adequate prior negative screenings. | A | A | A |
| Gemini Pro | 7. The recommended intervals for cervical cancer screening include:  - Women under 25 years old: Many doctors recommend Pap tests every 3 years.  - Women 25 to 65 years old: Most doctors recommend Pap tests and HPV tests every 5 years or Pap tests every 3 years.  - Women over 65 years old: If they have had regular screening results in the past, some doctors suggest stopping screening although the exact age and frequency can vary. | A | A | A |
| Mistral-7B-v0.2 (With prompt) | 7. The recommended intervals for cervical cancer screening are as follows: a) Women ages 21-29: Every 3 years with a Pap test or every 5 years with a co-test (Pap and HPV test combined). b) Women ages 30-65: Every 5 years with a co-test or every 3 years with a Pap test. c) Women over 65: Depending on their individual screening history, cervical cancer screening may not be necessary after the age of 65 if they have had regular screening and no abnormal results within the past 10 years. Women with a history of cervical precancer or cervical cancer should continue to get screened. | C | B | B |
| Mistral-7B-v0.2 | 7. The recommended intervals for cervical cancer screening depend on the age of the individual and the results of previous screens. In general, women between the ages of 21 and 29 should get screened every three years with a Pap test, while women between the ages of 30 and 65 can get screened every three to five years with a Pap test and a HPV test (co-testing) or every five years with a Pap test alone. Women over 65 who have had three negative screens in a row can stop getting screened. However, women with a history of abnormal Pap tests or HPV infection may need more frequent screening. | C | B | C |
| Starling-LM-7B alpha (With prompt) | 7. The recommended intervals for cervical cancer screening vary depending on the specific screening method and the age of the individual. For example, in the United States, the American Cancer Society recommends that women between the ages of 21 and 29 undergo cervical cancer screening every three years using the Pap test. Women aged 30 to 65 should undergo both the Pap test and HPV testing every five years, or the Pap test alone every three years. Women over the age of 65 who have had regular screening with negative results in the past 10 years may be advised to stop screening, although this decision should be made on an individual basis. It is important to note that these guidelines may vary depending on the specific organization or country, so it is essential to follow the recommendations provided by local health authorities. | A | A | A |
| Starling-LM-7B alpha | 7. The recommended intervals for cervical cancer screening vary depending on a woman's age and risk factors. For women aged 21 to 29, the recommended interval is every three years. For women aged 30 to 65, the recommended interval is every five years for a Pap test plus an HPV test, or every three years for a Pap test alone. After age 65, the recommended interval is typically every five years if the woman has had at least three normal tests in a row and no abnormal results in the past 10 years. | A | A | A |
| HuatuoGPT (With prompt) | 7. The recommended interval for cervical cancer screening varies by age group. For women between the ages of 21 and 65, screening is recommended every three to five years, depending on their individual risk. | C | B | C |
| HuatuoGPT | 7. The recommended interval for cervical cancer screening varies according to age. For women aged 21-29 years, screening is recommended every 3 years. For women aged 30-65 years, screening is recommended every 5 years, but the interval can be extended to every 5 years if cervical cytology has been performed during this period and the results are normal. For women over 65 years of age, screening is no longer necessary if they have had normal results in the past 3 consecutive screenings. | B | B | B |
| BioMedLM 2.7B (With prompt) | 7. What are the recommended intervals for cervical cancer screening, and do these intervals vary among different age groups? The recommended intervals for cervical cancer screening are every 3 years for women aged 21 to 29 years, every 5 years for women aged 30 to 65 years, and every 3 years for women aged 65 years or older. The intervals are based on the results of the randomized controlled trials (RCTs) that were conducted in the 1970s and 1980s. The RCTs were conducted in the United States and Europe, and the results were applied to the United States population. The results of the RCTs were not applicable to the Korean population because the incidence of cervical cancer in Korea is lower than that in the United States and Europe. The incidence of cervical cancer in Korea is 0.8 per 100,000 women, which is lower than that in the United States (8.8 per 100,000 women) and Europe (8.6 per 100,000 women). The incidence of cervical cancer in Korea is also lower than that in other Asian countries, such as Japan (4.8 per 100,000 women) and China (4.6 per 100,000 women). The incidence of cervical cancer in Korea is also lower than that in other countries, such as the United States (8.8 per 100,000 women) and Europe (8.6 per 100,000 women). The incidence of cervical cancer in Korea is lower than that in other Asian countries, such as Japan (4.8 per 100,000 women) and China (4.6 per 100,000 women). | C | C | C |
| BioMedLM 2.7B | 7. The recommended intervals for cervical cancer screening are every 3 years for women aged 30 to 65 years, and every 5 years for women aged 65 years or older. | C | C | C |
| Microsoft Phi2 (With prompt) | 7. The recommended intervals for cervical cancer screening vary among different age groups. For women aged 21-29, screening is recommended every three years. For women aged 30-65, screening is recommended every five years. For women aged 65 and older, screening is not recommended unless there are specific risk factors present. | B | B | B |
| Microsoft Phi2 | 7. The recommended intervals for cervical cancer screening vary among different age groups. For example, women aged 21 to 29 years should undergo screening every three years, while women aged 30 to 65 years should undergo screening every five years. | B | B | B |
| QiZhenGPT (With prompt) | 7. The recommended intervals for cervical cancer screening vary among different age groups. For example, the recommended interval for women aged 15-19 is 28-36 months, while for women aged 20-29 it is 36-48 months. These intervals should be reviewed and updated periodically to reflect changes in population health and sexual behavior. | D | C | C |
| QiZhenGPT | 7. The recommended intervals for cervical cancer screening vary based on age. For example, the World Health Organization recommends starting screening at 15 years old and continuing until age 65 or when a woman has had at least 10 years of sexual activity. However, for women aged 25-29, the recommended interval is 25 years old or until age 45 or when a woman has had at least 15 years of sexual activity. | D | D | D |
| **8.Is cervical cancer screening universally recommended for all age groups? If not, what are the reasons for excluding certain age groups from undergoing cervical cancer screening?** | | | | |
| ChatGPT4.0 turbo (With prompt) | 8. **Cervical Cancer Screening Recommendations by Age Group**: Cervical cancer screening is not universally recommended for all age groups. Screening is not recommended for individuals under the age of 21 regardless of sexual activity, as cervical cancer is rare in this age group and the harms from screening (such as unnecessary treatment of transient cervical changes) outweigh the benefits. For women over 65 who have had adequate prior screening and are not at high risk, screening can be discontinued due to the low likelihood of developing cervical cancer and the high probability of false positives leading to unnecessary treatments. | A | A | A |
| ChatGPT4.0 turbo | 8. **Universal Recommendations for Cervical Cancer Screening**:  - Cervical cancer screening is not universally recommended for all age groups. Screening is typically not recommended for:  - **Women under 21 years**: The risk of cervical cancer is very low in this group, and younger women often clear HPV infections effectively on their own.  - **Women over 65 years who have had adequate prior screening and are not at high risk**: The likelihood of new, clinically significant HPV infections decreases, and continued screening in this group yields diminishing benefits.  - **Women who have had a total hysterectomy for non-cancerous reasons**: If the cervix has been removed and there was no history of a high-grade precancerous lesion or cervical cancer, screening is not necessary. | A | A | A |
| Claude 2 (With prompt) | 8. Cervical screening is not indicated routinely before age 21 or over age 65 in those with prior adequate negative screening history. The very low risk of developing cervical cancer in these settings does not justify routine screening and risk of false positive results/over-treatment. | A | A | A |
| Claude 2 | 8. Routine screening is not recommended before age 21 or after age 65 if past screenings have been consistently normal. Screening is not beneficial in those under 21 as HPV infections often clear spontaneously and cervical cancer risk is extremely low. Screening those over 65 with a history of normal results is unlikely to provide benefit. | B | B | B |
| Gemini Pro (With prompt) | 8. Is cervical cancer screening universally recommended for all age groups? If not, what are the reasons for excluding certain age groups from undergoing cervical cancer screening?  Cervical cancer screening is not universally recommended for all age groups.   Women under 21: The risk of cervical cancer is extremely low in this age group, and the benefits of screening do not outweigh the potential harms.  Women over 65: Screening may be discontinued if they have had adequate prior negative screenings and are not at high risk for cervical cancer. | B | C | C |
| Gemini Pro | 8. Cervical cancer screening is generally recommended for all age groups of sexually active women between the ages of 21 to 65 years old. However, the decision to start or stop screening for older women may depend on a woman's health status, life expectancy, and past screening results. Women with certain medical conditions that compromise their immune system, such as HIV/AIDS, may require more frequent screening. | C | C | C |
| Mistral-7B-v0.2 (With prompt) | 8. Cervical cancer screening is not universally recommended for all age groups. According to the American Cancer Society, routine cervical cancer screening can generally be stopped for women 65 or older who have had three negative tests: one HPV test and two consecutive negative Pap tests within the past 10 years. Women with a history of cervical abnormalities, a weakened immune system, or other risk factors should continue to be screened. | B | C | C |
| Mistral-7B-v0.2 | 8. Cervical cancer screening is not universally recommended for all age groups. Women under 21 do not need to be screened unless they have abnormal Pap tests or other risk factors. Women over 65 with a history of negative screens may not need to be screened anymore. Women with certain medical conditions or a weakened immune system may also need special considerations when it comes to cervical cancer screening. | C | C | C |
| Starling-LM-7B alpha (With prompt) | 8. Cervical cancer screening is not universally recommended for all age groups. The reason for excluding certain age groups from undergoing cervical cancer screening is primarily due to the risk-benefit analysis. For example, women under the age of 21 are less likely to develop cervical cancer, and screening in this age group may result in unnecessary interventions and anxiety. On the other hand, women over the age of 65 who have had regular screening with negative results in the past 10 years may be advised to stop screening, as the risk of developing cervical cancer in this age group is relatively low. However, it is important to note that cervical cancer screening guidelines may vary depending on the specific organization or country, so it is essential to follow the recommendations provided by local health authorities. | A | A | A |
| Starling-LM-7B alpha | 8. Cervical cancer screening is not universally recommended for all age groups. The guidelines provided by the ACS and ACOG are based on the assumption that women at average risk of cervical cancer will benefit from regular screening. However, certain age groups may be excluded from screening if they have a low risk of developing cervical cancer or if they have undergone a hysterectomy. | C | C | C |
| HuatuoGPT (With prompt) | 8. Cervical cancer screening is generally recommended for women of all ages, unless contraindicated. | D | D | D |
| HuatuoGPT | 8. Cervical cancer screening is not universally recommended for everyone. Screening may not be needed for women who have no risk factors and who have had normal screening results in the past. For example, a woman who has undergone a total hysterectomy may not need to be screened if there is no cervical tissue left after the surgery. However, cervical cancer screening should still be performed in high-risk patients, such as those with HIV infection, a suppressed immune system, or women who have been taking oral contraceptives for more than 10 years. | C | C | C |
| BioMedLM 2.7B (With prompt) | 8. Is cervical cancer screening universally recommended for all age groups? If not, what are the reasons for excluding certain age groups from undergoing cervical cancer screening? Yes, cervical cancer screening is recommended for all age groups. The reasons for excluding certain age groups from undergoing cervical cancer screening are as follows: a\. The incidence of cervical cancer is low in women aged \<30 years. b\. The incidence of cervical cancer is low in women aged \>65 years. c\. The incidence of cervical cancer is low in women aged \>75 years. d\. The incidence of cervical cancer is low in women aged \>85 years. | D | D | D |
| BioMedLM 2.7B | 8. The age group is too young. The age group is too old. | C | C | C |
| Microsoft Phi2 (With prompt) | 8. Cervical cancer screening is not universally recommended for all age groups. Certain age groups, such as women over 65, may not require screening unless there are specific risk factors present. This is because the risk of developing cervical cancer decreases with age, and the benefits of screening may not outweigh the potential harms. | A | A | A |
| Microsoft Phi2 | 8. Cervical cancer screening is not universally recommended for all age groups. Certain age groups, such as women aged 70 years and older, may not require cervical cancer screening if they have had regular screenings in the past and have a low risk of developing cervical cancer. | B | B | B |
| QiZhenGPT (With prompt) | 8. cervical cancer screening is not universally recommended for all age groups. The age range for starting screening is 21-65 years old. The main reasons for excluding certain age groups from undergoing cervical cancer screening are: a. Women aged 66-70 years old: These women may have pre-existing health conditions that make them more susceptible to the disease. b. Women aged 71-75 years old: These women may have a higher risk of developing advanced stages of the disease. c. Women aged 80 years and older: These women may have a higher risk of developing complications from screening. | C | D | D |
| QiZhenGPT | 8. cervical cancer screening is not universally recommended for all age groups. The World Health Organization recommends starting screening at 15 years old and continuing until age 65 or when a woman has had at least 10 years of sexual activity. However, some countries have different age recommendations for cervical cancer screening. For example, in some countries, screening is recommended starting at 18 years old or when a woman has had at least 15 years of sexual activity. | D | D | D |
| **9.How necessary is cervical cancer screening for women who have undergone total hysterectomy?** | | | | |
| ChatGPT4.0 turbo (With prompt) | 9. **Cervical Cancer Screening Post-Total Hysterectomy**: Cervical cancer screening is not necessary for women who have undergone a total hysterectomy (removal of the uterus and cervix) for reasons not related to cervical cancer or pre-cancerous lesions. Since the cervix is removed during the procedure, the risk of developing cervical cancer is effectively eliminated. However, if the hysterectomy was performed as a treatment for cervical cancer or serious pre-cancer, the woman may need continued surveillance depending on her individual clinical circumstances. | A | A | A |
| ChatGPT4.0 turbo | 9. **Necessity of Cervical Cancer Screening Post-Hysterectomy**:  - Women who have undergone a total hysterectomy (removal of the uterus and cervix) for reasons not related to cervical cancer or a precancerous condition generally do not need cervical cancer screening. However, if the hysterectomy was performed due to cervical cancer or a precancerous lesion, these women may need continued screening according to their doctor's recommendations. | A | A | A |
| Claude 2 (With prompt) | 9. Women who have undergone total hysterectomy (removal of uterus and cervix) for non-cancer indications and have no history of high-grade cervical dysplasia can safely discontinue cervical cancer screening. For these patients, the risk organ has been removed. | A | A | A |
| Claude 2 | 9. Women who have had a total hysterectomy (removal of uterus and cervix) for non-cancerous reasons generally do not need further screening, unless the surgery was related to treatment of precancer or cancer. | A | A | A |
| Gemini Pro (With prompt) | 9. How necessary is cervical cancer screening for women who have undergone total hysterectomy?  The necessity of cervical cancer screening for women who have undergone total hysterectomy depends on the type of hysterectomy performed.   Total hysterectomy with removal of the cervix (radical hysterectomy): No further screening is necessary.  Total hysterectomy without removal of the cervix: Screening may still be necessary if the cervix was not removed or if there is a history of precancerous lesions. | A | A | A |
| Gemini Pro | 9. Women who have undergone a total hysterectomy, where the entire uterus and cervix are removed, are generally not recommended for cervical cancer screening, as the involvement of the cervix in cancer development is eliminated. | A | B | B |
| Mistral-7B-v0.2 (With prompt) | 9. For women who have undergone a total hysterectomy with the removal of the cervix, cervical cancer screening may not be necessary. However, it's crucial for these women to discuss their medical history with their healthcare provider. Additionally, women with a history of abnormal pre-cancerous cells or cervical cancer should continue to get screened, even after a hysterectomy. | A | A | A |
| Mistral-7B-v0.2 | 9. Cervical cancer screening is necessary for women who have undergone total hysterectomy only if they had a diagnosis of cervical cancer or precancerous changes before the surgery. If the entire cervix was removed during the surgery, there is a very low risk of cervical cancer developing, and these women do not need to get screened anymore. | A | A | A |
| Starling-LM-7B alpha (With prompt) | 9. Cervical cancer screening is generally not necessary for women who have undergone total hysterectomy, especially if the cervix was removed during the procedure. This is because the cervix is the primary site of cervical cancer, and its removal significantly reduces the risk of developing cervical cancer. However, it is important to note that women who have undergone a total hysterectomy may still be at risk for other types of gynecologic cancers, so regular screening for these cancers may be recommended. It is important to consult with a healthcare provider to determine the appropriate screening recommendations for an individual patient. | A | A | A |
| Starling-LM-7B alpha | 9. Cervical cancer screening is not necessary for women who have undergone a total hysterectomy, especially if the cervix was removed, as the risk of cervical cancer is significantly reduced. However, these women should still undergo regular pelvic exams to check for other gynecologic issues. | B | C | B |
| HuatuoGPT (With prompt) | 9. Cervical cancer screening is usually not required for women who have undergone total hysterectomy, but is sometimes recommended at the discretion of the physician and in specific cases. | B | B | B |
| HuatuoGPT | 9. Cervical cancer screening is still important for women who have had a total hysterectomy. Total hysterectomy can prevent the development of cervical cancer, but it cannot reverse cancer that has already occurred. Screening allows for early detection of cervical cancer, improves cure rates, and helps prevent recurrence of the cancer. | A | A | A |
| BioMedLM 2.7B (With prompt) | 9. How necessary is cervical cancer screening for women who have undergone total hysterectomy? The answer to this question is not as simple as it may seem. The answer depends on the age of the patient, the type of hysterectomy, and the length of time since the hysterectomy. The risk of cervical cancer is increased in women who have undergone a hysterectomy. | C | C | C |
| BioMedLM 2.7B | 9. How necessary is cervical cancer screening for women who have undergone total hysterectomy? Cervical cancer screening is not necessary for women who have undergone total hysterectomy. | C | C | C |
| Microsoft Phi2 (With prompt) | 9. Cervical cancer screening is not necessary for women who have undergone total hysterectomy, unless there are specific risk factors present. This is because the cervix is removed during a total hysterectomy, which eliminates the risk of developing cervical cancer. | A | A | A |
| Microsoft Phi2 | 9. Cervical cancer screening is not necessary for women who have undergone total hysterectomy, including removal of the cervix. This is because the cervix is no longer present and therefore cannot develop cervical cancer. | A | A | A |
| QiZhenGPT (With prompt) | 9. cervical cancer screening is necessary for women who have undergone total hysterectomy. These women may have difficulty accessing screening services due to their medical conditions. | D | D | D |
| QiZhenGPT | 9. cervical cancer screening is necessary for women who have undergone total hysterectomy. Hysterectomy is a surgical procedure that removes the uterus, which is one of the two organs that are involved in the menstrual cycle. Women who have undergone total hysterectomy may not be able to have regular menstrual periods, which can increase their risk of developing cervical cancer. Therefore, screening for cervical cancer is recommended for women who have undergone total hysterectomy to help identify and treat any early-stage cases of the disease. | D | D | D |
| **10.What is the significance of cervical cancer screening?** | | | | |
| ChatGPT4.0 turbo (With prompt) | 10. **Significance of Cervical Cancer Screening**: Cervical cancer screening is significant because it is a preventive measure that can detect precancerous or cancerous changes in the cervix before symptoms appear. Early detection through screening allows for timely intervention, which can significantly reduce the morbidity and mortality associated with cervical cancer. Screening has been instrumental in decreasing the incidence and fatalities of cervical cancer in populations where it is routinely performed, making it a critical component of women's health care. | A | A | A |
| ChatGPT4.0 turbo | 10. **Significance of Cervical Cancer Screening**:  - **Early Detection**: Screening can detect changes in the cervix before cancer develops and when early-stage cancer may be more treatable.  - **Prevention**: Identifying and treating precancerous changes can prevent most cervical cancers.  - **Reduction in Mortality**: Regular screening has been shown to significantly reduce the number of deaths from cervical cancer through early detection and treatment.  - **Public Health Impact**: Widespread screening has dramatically decreased the incidence of cervical cancer in countries with routine screening programs.  Cervical cancer screening remains a critical component of women's health care, significantly impacting the reduction of morbidity and mortality associated with cervical cancer. | A | A | A |
| Claude 2 (With prompt) | 10. Regular cervical screening allows for the early detection and treatment of precancerous changes on the cervix caused by high-risk HPV infection. This prevents possible progression to invasive cervical cancer. So screening plays a pivotal role in cervical cancer prevention and mortality reduction. | A | A | A |
| Claude 2 | 10. Cervical cancer screening allows detection and treatment of precancerous changes and early stage cervical cancers. Widespread screening has dramatically reduced both cervical cancer incidence and related mortality over the past 60 years. It remains an essential preventive service for those ages 21-65. Here are the answers to your follow-up questions: | A | A | A |
| Gemini Pro (With prompt) | 10. What is the significance of cervical cancer screening? Cervical cancer screening is highly significant for several reasons:  Early detection: It allows for early detection of precancerous lesions and cervical cancer, enabling timely treatment and improving the chances of successful outcomes.  Reduced mortality: Regular screening has significantly reduced cervical cancer mortality rates over the past few decades.  Improved survival rates: Early detection and treatment lead to improved survival rates for women diagnosed with cervical cancer.  Prevention: Screening can help prevent cervical cancer by identifying and treating precancerous lesions before they develop into cancer. | A | A | A |
| Gemini Pro | 10. Cervical cancer screening is significant because it can help detect precancerous changes in the cervix before they progress to cancer. Screening can prevent cervical cancer and its complications, making it a critical component of women's health and overall wellness. Regular cervical cancer screening also helps identify women who may be at high risk for developing cervical cancer due to HPV infection and other risk factors. | B | A | A |
| Mistral-7B-v0.2 (With prompt) | 10. Cervical cancer screening is a preventative medical measure designed to identify precancerous or cancerous changes in the cervix, and it saves lives by enabling early interventions. Regular cervical cancer screening: a) Detects precancerous lesions that can be treated before progressing to cervical cancer, b) Diagnoses cervical cancer at an early stage when treatment is most effective, reducing the risk of invasive and advanced-stage disease, and c) Provides educational opportunities about sexually transmitted infections, reproductive health, and cancer risks, leading to improved overall health. | A | A | A |
| Mistral-7B-v0.2 | 10. Cervical cancer screening is significant because it can help prevent cervical cancer by detecting precancerous changes and allowing for treatment before cancer develops. It can also help identify women who are at higher risk for cervical cancer and provide early intervention and appropriate follow-up care. Regular cervical cancer screening is an essential component of women's health care and can save lives. | A | A | A |
| Starling-LM-7B alpha (With prompt) | 10. The significance of cervical cancer screening lies in its ability to detect precancerous or early-stage cervical cancer and precursor lesions, which can then be treated to prevent the development of invasive cervical cancer. Cervical cancer screening has been shown to significantly reduce cervical cancer incidence and mortality rates. Additionally, cervical cancer screening can also help identify women at high risk for developing cervical cancer, allowing for targeted interventions and prevention strategies. Overall, cervical cancer screening is a critical tool in the early detection and prevention of cervical cancer, saving lives and improving health outcomes for women worldwide. | A | A | A |
| Starling-LM-7B alpha | 10. The significance of cervical cancer screening lies in its ability to detect early signs of cervical cancer or precancerous conditions. Early detection and treatment can prevent the development of invasive cervical cancer, which has a much lower survival rate. Regular screening also helps in the prevention of cervical cancer through the detection and removal of precancerous lesions. | A | A | A |
| HuatuoGPT (With prompt) | 10. The significance of cervical cancer screening lies in the early detection and treatment of cervical cancer, thereby improving survival and cure rates. It also helps to identify women at risk of cervical cancer and provide preventive care. | A | A | A |
| HuatuoGPT | 10. Cervical cancer screening is very important because it can help detect the early stages of cervical cancer development and treat the cancer before it spreads. Through early detection and prevention, the mortality rate of cervical cancer can be drastically reduced. In addition, screening can help identify women who are at risk of developing cervical cancer and provide them with additional care and monitoring. | A | A | A |
| BioMedLM 2.7B (With prompt) | 10.What is the significance of cervical cancer screening? The purpose of cervical cancer screening is to detect precancerous lesions and early invasive cervical cancer. The goal of screening is to reduce the incidence and mortality of cervical cancer. The American Cancer Society (ACS) recommends that women aged 21 to 65 years be screened every 3 years with a Papanicolaou (Pap) test.^[@bibr1-2374289519898847]^ The ACS recommends that women aged 30 to 65 years be screened every 5 years with a human papillomavirus (HPV) | A | A | A |
| BioMedLM 2.7B | 10.What is the significance of cervical cancer screening? a\. Cervical cancer is the second most common cancer in women worldwide. b\. Cervical cancer is preventable. c\. Cervical cancer can be detected early. | B | B | B |
| Microsoft Phi2 (With prompt) | 10. Cervical cancer screening is significant because it can detect precancerous lesions and cervical cancer at an early stage, when treatment is most effective. Early detection can also prevent the spread of cancer to other parts of the body and improve the chances of survival. | B | A | B |
| Microsoft Phi2 | 10. Cervical cancer screening is significant because it can help detect precancerous changes in the cervix before they develop into cancer. Early detection of cervical cancer can lead to more effective treatment and better outcomes for patients. | A | B | B |
| QiZhenGPT (With prompt) | 10. The significance of cervical cancer screening is that it helps to prevent the spread of the disease to the lungs and other parts of the body. Early detection of cervical cancer can also lead to prompt treatment, which can improve outcomes and reduce the risk of complications. Additionally, cervical cancer screening can help to identify women who may be at risk of developing the disease due to their sexual behavior or lifestyle, which can help to prevent the disease from developing in the first place. | A | B | B |
| QiZhenGPT | 10. The significance of cervical cancer screening is that it can help prevent the spread of the disease to the body and improve the quality of life for women who have been diagnosed with the disease. Early detection and treatment can also reduce the risk of developing advanced stages of the disease, which can be difficult to treat and can have a high mortality rate. Additionally, cervical cancer screening can help identify women who may be at risk of developing the disease due to their sexual behavior or lifestyle, which can help prevent the disease from developing in the first place. | C | C | C |
| **11.Why is the combined use of cytological screening (Pap smear) and Human Papillomavirus (HPV) testing not recommended for women aged 21 to 29?** | | | | |
| ChatGPT4.0 turbo (With prompt) | 11. **Reason Against Combined Screening for Ages 21-29**: The combined use of cytological screening (Pap smear) and Human Papillomavirus (HPV) testing is not recommended for women aged 21 to 29 because HPV infections are highly prevalent and usually transient in this age group. Most HPV infections in younger women will clear on their own without causing long-term harm. Introducing HPV testing could lead to over-diagnosis and unnecessary treatment of HPV infections that would resolve naturally. The focus at this age is primarily on detecting high-grade cervical lesions through cytological tests, which are less likely to result in overtreatment. | A | A | A |
| ChatGPT4.0 turbo | 11. **Reasons Against Combined Screening for Women Aged 21 to 29**:  - **High HPV Prevalence**: HPV infection is very common in younger women, most of whom will clear the virus naturally without intervention.  - **Low Risk of Cancer**: The risk of cervical cancer in this age group is very low, and most HPV infections do not lead to cancer.  - **Potential for Overtreatment**: Using HPV testing in this age group could lead to unnecessary follow-up procedures and treatments for infections that would resolve on their own, causing potential harm and anxiety. | A | A | A |
| Claude 2 (With prompt) | 11. In women 21-29, HPV infection is very common and usually clears spontaneously within 1-2 years. Abnormal cytology and positive HPV testing in this age group is more likely to induce unnecessary colposcopies and over-treatment of what would likely have been transient HPV/dysplasia. Therefore, cytology alone is recommended to avoid excessive false positives and procedure-related harms like preterm delivery. | A | A | A |
| Claude 2 | 11. For women ages 21-29, the rate of HPV infection is high but most infections clear spontaneously. Abnormal cells triggered by transient HPV infections often resolve without intervention. Since both HPV testing and Pap smears can lead to unnecessary follow-up procedures and overtreatment in this age group, Pap testing alone every 3 years is recommended. | A | A | A |
| Gemini Pro (With prompt) | 11. Why is the combined use of cytological screening (Pap smear) and Human Papillomavirus (HPV) testing not recommended for women aged 21 to 29?  The combined use of Pap smear and HPV testing is not recommended for women aged 21 to 29 because:   High rate of HPV infection in young women: HPV infection is very common in young women, and most infections clear up on their own without causing any health problems.  Overdiagnosis and unnecessary treatment: Co-testing in this age group can lead to overdiagnosis of precancerous lesions that may never progress to cancer, resulting in unnecessary anxiety and invasive procedures.  HPV testing is less accurate in young women: HPV testing is less accurate in detecting precancerous lesions in young women compared to older women.  Therefore, Pap smear alone is sufficient for cervical cancer screening in women aged 21 to 29. | C | B | C |
| Gemini Pro | 11. The combined use of cytological screening (Pap smear) and Human Papillomavirus (HPV) testing is not recommended for women aged 21 to 29 due to the increased complexity and cost of the testing, without significant additional benefits. Pap tests alone are sufficient for this age group to identify precancerous and cancerous changes in the cervix. | B | B | B |
| Mistral-7B-v0.2 (With prompt) | 11. The combined use of cytological screening (Pap smear) and Human Papillomavirus (HPV) testing is not recommended for women aged 21 to 29 because: a) HPV infections are common and often transient; b) HPV testing can lead to over-diagnosis and unnecessary treatments for low-risk HPV infections; and c) The benefits of HPV testing in young women do not outweigh the potential risks and costs. Younger women should follow the recommended schedule of Pap tests every 3 years. | A | A | A |
| Mistral-7B-v0.2 | 11. The combined use of cytological screening (Pap smear) and Human Papillomavirus (HPV) testing is not recommended for women aged 21 to 29 because HPV testing alone is less cost-effective and less accurate for women in this age group. Most HPV infections are transient and resolve on their own, and the majority of women in this age group have normal Pap tests. HPV testing can produce false positives, leading to unnecessary follow-up procedures and anxiety. Pap tests are still recommended every three years for women aged 21 to 29 because they are more effective in detecting precancerous changes in the cervix. | B | B | B |
| Starling-LM-7B alpha (With prompt) | 11. The combined use of cytological screening (Pap smear) and Human Papillomavirus (HPV) testing is not recommended for women aged 21 to 29 because HPV infections are very common in this age group, and most of them are transient and resolve spontaneously without causing any harm. Including HPV testing in this age group would lead to a high number of false-positive results, causing unnecessary anxiety and follow-up procedures. | A | A | A |
| Starling-LM-7B alpha | 11. The combined use of cytological screening (Pap smear) and Human Papillomavirus (HPV) testing is not recommended for women aged 21 to 29 because HPV infection is very common in this age group, and most HPV infections are transient and resolve spontaneously. Therefore, the addition of HPV testing to Pap testing in this age group would lead to a significant number of false-positive results, unnecessary follow-up, and overtreatment. | B | B | B |
| HuatuoGPT (With prompt) | 11. Concurrent cytologic screening (pap smear) and human papillomavirus (HPV) testing is not recommended for women aged 21 to 29 years because about 5% of HPV-positive women in this age group develop cervical cancer, and the false-positive rate of cytologic screening is 5%, which can lead to unnecessary interventions and concerns. | C | B | B |
| HuatuoGPT | 11. The combination of cytologic screening (Pap smear) and human papillomavirus (HPV) testing is not recommended for women aged 21 to 29 years. This is because the false-positive rate for cytologic screening is higher in younger women, and HPV infection is common in this age group, so negative results may not be reliable. In addition, there is no evidence that HPV testing is useful or valuable in women aged 21 to 29 years because the risk of cervical cancer is low in this age group. | A | A | A |
| BioMedLM 2.7B (With prompt) | 11. Why is the combined use of cytological screening (Pap smear) and Human Papillomavirus (HPV) testing not recommended for women aged 21 to 29? The combined use of cytological screening (Pap smear) and HPV testing is not recommended for women aged 21 to 29. The reason is that the sensitivity of HPV testing is lower than that of Pap smear, and the specificity of HPV testing is higher than that of Pap smear. Therefore, the combined use of Pap smear and HPV testing is not recommended for women aged 21 to 29 . | D | C | D |
| BioMedLM 2.7B | 11. Why is the combined use of cytological screening (Pap smear) and Human Papillomavirus (HPV) testing not recommended for women aged 21 to 29?screening? The combined use of cytological screening (Pap smear) and HPV testing is not recommended for women aged 21 to 29. | D | D | D |
| Microsoft Phi2 (With prompt) | 11. Cervical cancer screening is significant because it can detect abnormal cells in the cervix before they become cancerous. Early detection can lead to early treatment, which can improve the chances of survival. | D | D | D |
| Microsoft Phi2 | 11. The combined use of cytological screening (Pap smear) and Human Papillomavirus (HPV) testing is not recommended for women aged 21 to 29 because the risk of cervical cancer is relatively low during this age group. | B | B | B |
| QiZhenGPT (With prompt) | 11. The combined use of cytological screening (Pap smear) and Human Papillomavirus (HPV) testing is not recommended for women aged 21 to 29 because it increases the risk of oversampling low-grade HPV infections, which can lead to the development of cervical cancer in the future. | D | D | D |
| QiZhenGPT | 11. The combined use of cytological screening (Pap smear) and Human Papillomavirus (HPV) testing is not recommended for women aged 21 to 29 because it increases the risk of over-sampling low-grade cervical abnormalities, which can lead to false-negative results. Women in this age group are at an increased risk of developing cervical cancer and should only receiveHPV testing if they have a positive result from a cytological screening test. | D | D | D |
| **12.Is there an invariable link between HPV infection and the onset of cervical cancer?** | | | | |
| ChatGPT4.0 turbo (With prompt) | 12. **Link Between HPV Infection and Cervical Cancer**: While HPV infection is a necessary factor in the development of nearly all cases of cervical cancer, it is not sufficient on its own to cause cancer. The majority of HPV infections resolve spontaneously without causing any clinical issues. Persistent infection with high-risk HPV types, however, can lead to the development of precancerous lesions, which may progress to cervical cancer if not detected and treated early. Thus, while there is a strong link, not all HPV infections lead to cervical cancer. | A | A | A |
| ChatGPT4.0 turbo | 12. **Link Between HPV Infection and Cervical Cancer**:  - While persistent infection with high-risk HPV types is necessary for the development of most cervical cancers, it is not sufficient alone to cause cancer. Most HPV infections resolve spontaneously and do not lead to cervical cancer. Factors such as the individual’s immune response and genetic factors also play crucial roles in the progression from HPV infection to cervical cancer. | A | A | A |
| Claude 2 (With prompt) | 12. No, most HPV infections will spontaneously clear within 1-2 years without intervention in healthy individuals. Persistent infection with high-risk HPV types is required for precancerous cervical changes to develop and progress. Additional co-factors like smoking and immunosuppression facilitate this carcinogenic process. | A | A | A |
| Claude 2 | 12. No, most HPV infections do not lead to cervical cancer. In over 90% of cases, the infection clears spontaneously within 1-2 years. Persistent high-risk HPV infection is required for precancer/cancer development. Additional cofactors influence which cases progress. | A | A | A |
| Gemini Pro (With prompt) | 12. Is there an invariable link between HPV infection and the onset of cervical cancer?  No, there is not an invariable link between HPV infection and the onset of cervical cancer.   Most HPV infections do not cause cancer: The vast majority of HPV infections clear up on their own without causing any health problems.  Only certain HPV types are high-risk: There are over 100 types of HPV, but only a few are considered high-risk for causing cervical cancer.  Other factors contribute to cervical cancer development: Other factors, such as smoking, weakened immune system, and long-term use of oral contraceptives, can also increase the risk of cervical cancer.  Therefore, while HPV infection is a necessary cause of cervical cancer, it is not the only factor involved. | A | B | B |
| Gemini Pro | 12. Yes, there is a strong link between HPV infection and the onset of cervical cancer. HPV is the most common sexually transmitted infection and is the primary cause of cervical cancer. The majority of HPV infections are cleared by the body on their own, but some infections persist and can lead to cervical precancer or cancer if not detected and treated. | C | C | C |
| Mistral-7B-v0.2 (With prompt) | 12. Yes, there is a strong link between HPV infection and the onset of cervical cancer. HPV is the primary cause of most cervical cancers. According to the American Cancer Society, almost all cervical cancers are caused by HPV. Many cervical precancers and cancerous growths are also linked to HPV infection, making early detection through screening even more essential. | D | D | D |
| Mistral-7B-v0.2 | 12. Yes, there is a strong link between HPV infection and the onset of cervical cancer. HPV is a sexually transmitted infection, and most cervical cancers are caused by certain high-risk HPV types. Regular cervical cancer screening, such as a Pap test or HPV test, can help detect HPV infection before cancer develops, allowing for early treatment and prevention of cervical cancer. | C | C | C |
| Starling-LM-7B alpha (With prompt) | 12. There is not an invariable link between HPV infection and the onset of cervical cancer. While HPV infection is a necessary cause of cervical cancer, it is not sufficient on its own to cause the disease. Other factors, such as the persistence of the infection, the specific HPV type, and host factors, play a role in the progression from HPV infection to cervical intraepithelial neoplasia (CIN) and eventually to invasive cervical cancer. | A | A | A |
| Starling-LM-7B alpha | 12. There is not an invariable link between HPV infection and the onset of cervical cancer. While HPV infection is a necessary cause of cervical cancer, it is not sufficient on its own to cause cancer. Other factors, such as the immune system and lifestyle factors, also play a role in the development of cervical cancer. | A | A | A |
| HuatuoGPT (With prompt) | 12. HPV infection is not necessarily associated with the development of cervical cancer, but almost all cervical cancers originate from HPV infection. | B | A | B |
| HuatuoGPT | 12. There is no fixed link between HPV infection and the start of cervical cancer. Although most HPV infections go away on their own, they can still cause some infections to persist and lead to cervical cancer. | C | C | C |
| BioMedLM 2.7B (With prompt) | 12. Is there an invariable link between HPV infection and the onset of cervical cancer? The answer is yes. The link between HPV infection and the onset of cervical cancer is well established. The HPV virus is a sexually transmitted infection that is responsible for the development of cervical cancer. The virus is present in 99.7% of cervical cancers and in 99.5% of cervical intraepithelial neoplasia (CIN) lesions.[@bib1] The virus is also present in the genital tract of men and women who are not infected with cervical cancer.[@bib2] | D | D | D |
| BioMedLM 2.7B | 12. Is there an invariable link between HPV infection and the onset of cervical cancer? Yes. | D | D | D |
| Microsoft Phi2 (With prompt) | 12. The combined use of cytological screening (Pap smear) and HPV testing is not recommended for women aged 21 to 29 because the risk of false-positive results is high. This can lead to unnecessary follow-up tests and procedures, which can cause anxiety and discomfort for the patient. | D | D | D |
| Microsoft Phi2 | 12. No, there is no invariable link between HPV infection and the onset of cervical cancer. While HPV infection is a risk factor for cervical cancer, not all HPV infections lead to the development of cancer. | A | A | A |
| QiZhenGPT (With prompt) | 12. There is no invariable link between HPV infection and the onset of cervical cancer. However, HPV is the most common cause of cervical cancer in women worldwide, and it can also cause precancerous changes in the cervix. | A | A | A |
| QiZhenGPT | 12. Yes, there is an invariable link between HPV infection and the onset of cervical cancer. HPV is a type of virus that causes a range of diseases, including cervical cancer. Women who are infected with HPV are at an increased risk of developing cervical cancer, but the exact risk depends on the type of HPV infection and the number of infections. | D | D | D |
| **13.How should one interpret an abnormal result from a cervical cancer screening test? Does such a result definitively indicate the presence of cervical cancer?** | | | | |
| ChatGPT4.0 turbo (With prompt) | 13. **Interpreting Abnormal Cervical Cancer Screening Results**: An abnormal result from a cervical cancer screening test does not definitively indicate the presence of cervical cancer. Abnormal results can range from minor changes such as atypical squamous cells of undetermined significance (ASCUS) to more significant findings like high-grade squamous intraepithelial lesions (HSIL), which suggest a higher risk of precancer. Follow-up testing, which may include repeat Pap tests, HPV testing, and/or a colposcopy, is typically recommended to determine the significance of the abnormal cells and the appropriate course of action. | A | A | A |
| ChatGPT4.0 turbo | 13. **Interpreting Abnormal Cervical Cancer Screening Results**:  - An abnormal result does not definitively indicate the presence of cervical cancer. Abnormal results usually indicate the presence of cervical cell changes, which could be minor (low-grade changes that often resolve on their own) or more significant (high-grade changes that are more likely to progress to cancer if untreated).  - Follow-up testing, such as repeat Pap tests, HPV testing, or a colposcopy (a closer examination of the cervix), is typically recommended to determine the nature and extent of the abnormal cells and to decide on appropriate management. | A | A | A |
| Claude 2 (With prompt) | 13. An abnormal cervical screening result indicates the need for further evaluation and close follow-up, but does NOT necessarily indicate cancer is already present. Further diagnostic procedures like colposcopy with biopsy are required to determine the actual histologic findings and severity prior to treatment recommendations. | A | A | A |
| Claude 2 | 13. An abnormal screening result indicates the need for further evaluation and follow-up, but does not definitively diagnose cancer. It may represent precancerous changes or early stage cancer, but often further testing is required for confirmation. Follow-up recommendations are based on the type of abnormality. | A | A | A |
| Gemini Pro (With prompt) | 13. How should one interpret an abnormal result from a cervical cancer screening test? Does such a result definitively indicate the presence of cervical cancer?  An abnormal result from a cervical cancer screening test does not definitively indicate the presence of cervical cancer.   Further testing is needed: An abnormal Pap smear or HPV test result may require further testing, such as colposcopy or biopsy, to determine the severity of the abnormality and the need for treatment.  Not all abnormal cells become cancerous: Many abnormal cells detected on screening tests do not progress to cancer.  Treatment depends on the severity of the abnormality: The type of treatment recommended will depend on the severity of the abnormality and the individual's risk factors.  Therefore, it is important to follow up with your doctor if you receive an abnormal screening result. | A | A | A |
| Gemini Pro | 13. An abnormal result from a cervical cancer screening test does not definitively indicate the presence of cervical cancer. An abnormal result may be due to various reasons, such as a false-positive, HPV infection, or precancerous changes. A healthcare professional will perform further diagnostic tests, such as a colposcopy or cone biopsy, to assess the cause and determine if any treatment is required. | A | A | A |
| Mistral-7B-v0.2 (With prompt) | 13. An abnormal result from a cervical cancer screening test does not definitively indicate the presence of cervical cancer. An abnormal result could be due to various factors, including inflammation, cellular changes, or inaccuracy of the test itself. Any abnormal results should be followed up with a colposcopy, which uses a microscope to examine the cervix and vagina, to further evaluate the underlying cause and determine the next steps for treatment or follow-up. | A | A | A |
| Mistral-7B-v0.2 | 13. An abnormal result from a cervical cancer screening test does not definitively indicate the presence of cervical cancer. Abnormalities can be caused by a variety of conditions, including benign conditions like inflammation or bacterial infection, and precancerous or cancerous changes. Further evaluation and diagnostic tests, such as a colposcopy or biopsy, may be necessary to determine the cause of the abnormal result and develop an appropriate treatment plan. | A | A | A |
| Starling-LM-7B alpha (With prompt) | 13. An abnormal result from a cervical cancer screening test should be interpreted with caution. While an abnormal result may indicate the presence of cervical cancer or its precursors, it does not definitively confirm the diagnosis. Further testing, such as colposcopy and biopsy, are necessary to confirm the presence of high-grade lesions or invasive cancer. | A | A | A |
| Starling-LM-7B alpha | 13. Here are some general guidelines for interpreting the results of cervical cancer screening tests: - A normal result indicates that no abnormal cells were found in the cervix. However, it is important to note that a normal result does not guarantee that cervical cancer will not develop in the future. Regular screening is still necessary. - An abnormal result may indicate the presence of precancerous changes in the cervix. Further testing, such as a colposcopy, is usually necessary to determine the exact nature of the abnormality. Treatment may be recommended if precancerous changes are detected. - A positive HPV test result indicates the presence of HPV infection. However, most HPV infections are transient and resolve spontaneously. Therefore, a positive HPV test result alone does not necessarily indicate the presence of cervical cancer or precancerous changes. | A | A | A |
| HuatuoGPT (With prompt) | 13. Abnormal results of cervical cancer screening tests should be evaluated further. A positive result does not necessarily indicate cervical cancer, and a negative result does not guarantee the absence of cervical cancer. | B | A | A |
| HuatuoGPT | 13. How an abnormal result from a cervical cancer screening test should be interpreted depends on the specific situation. In younger women, an abnormal cytology result may be due to cellular changes and does not necessarily mean that cervical cancer is present. In older women, an abnormal cytologic test result may be due to highly suspicious abnormal cells that require further testing to confirm or rule out the presence of cancer. Abnormal test results do not indicate with certainty the presence of cervical cancer and require further evaluation. | A | A | A |
| BioMedLM 2.7B (With prompt) | 13. How should one interpret an abnormal result from a cervical cancer screening test? Does such a result definitively indicate the presence of cervical cancer? The answer to this question is not definitive. The answer depends on the test used. The Pap smear is a test that is used to detect abnormal cells in the cervix. The Pap smear is a test that is used to detect abnormal cells in the cervix. | A | B | A |
| BioMedLM 2.7B | 13. How should one interpret an abnormal result from a cervical cancer screening test? Does such a result definitively indicate the presence of cervical cancer? Abnormal results from cervical cancer screening tests are not definitively diagnostic of cervical cancer. Abnormal results may indicate the presence of cervical cancer, but the test result may also be falsely positive. Abnormal results may also indicate the presence of precancerous lesions, but the test result may also be falsely positive. Abnormal results may also indicate the presence of other diseases, such as sexually transmitted diseases, but the test result may also be falsely positive. | A | A | A |
| Microsoft Phi2 (With prompt) | 13. An abnormal result from a cervical cancer screening test does not definitively indicate the presence of cervical cancer. Such a result may indicate the presence of precancerous or cancerous cells, but further testing is required to confirm the diagnosis. | A | A | A |
| Microsoft Phi2 | 13. An abnormal result from a cervical cancer screening test does not definitively indicate the presence of cervical cancer. It may indicate the presence of precancerous changes or abnormalities that require further evaluation and treatment. | A | A | A |
| QiZhenGPT (With prompt) | 13. When interpreting an abnormal result from a cervical cancer screening test, it is important to consider the number and type of abnormal cells, as well as the presence of other markers of cancer. | C | B | C |
| QiZhenGPT | 13. When interpreting an abnormal result from a cervical cancer screening test, it is important to consider the age of the woman, the number of screening tests performed, and the results of any previous screening tests. A abnormal result may indicate the presence of cervical cancer, but it is not definitively indicating the presence of the disease. It is important to follow up with further testing and consult with a healthcare provider for guidance on how to proceed. | A | A | A |
| **14.why yearly cervical cancer screening is not recommended?** | | | | |
| ChatGPT4.0 turbo (With prompt) | 14. **Reasons Against Yearly Cervical Cancer Screening**: Yearly cervical cancer screening is not recommended because it has not been shown to offer increased benefits over screening at the recommended intervals. More frequent screening can lead to increased psychological stress, unnecessary invasive procedures, and treatment of changes that would otherwise resolve naturally. Current guidelines are based on evidence that extending the interval between screenings is safe and effective, balancing the benefits of early detection with the risks of over-treatment. | A | A | A |
| ChatGPT4.0 turbo | 14. **Reasons Against Yearly Cervical Cancer Screening**:  - **Low Rate of Disease Progression**: Cervical cancer generally develops slowly, so annual screening does not significantly improve early detection rates compared to screening every three to five years.  - **Risk of Overtreatment**: More frequent screening can lead to unnecessary treatments for conditions that might never develop into cancer.  - **Cost-Effectiveness**: Less frequent screening is more cost-effective while still being effective in reducing the incidence of cervical cancer. | A | A | A |
| Claude 2 (With prompt) | 14. More frequent than every 3-5 years offers little additional cancer detection benefit for low-risk women, yet increases false positives/unnecessary procedures. It does not improve ultimate health outcomes. | A | A | A |
| Claude 2 | 14. Yearly screening is generally not needed because cervical cancer develops slowly over time. For those ages 21-29, screening every 3 years detects most precancers/cancers. Less frequent screening reduces potential harms like false positives without compromising detection. | A | B | A |
| Gemini Pro (With prompt) | 14. Why yearly cervical cancer screening is not recommended?  Yearly cervical cancer screening is not recommended because:   Most cervical cancers develop slowly: The majority of cervical cancers develop slowly over several years, giving ample time for detection with less frequent screening.  Overdiagnosis and unnecessary treatment: Frequent screening can lead to overdiagnosis of precancerous lesions that may never progress to cancer, resulting in unnecessary anxiety and invasive procedures.  Cost-effectiveness: Frequent screening is not cost-effective compared to screening at longer intervals.  Therefore, the recommended screening intervals vary depending on age and other factors. | A | A | A |
| Gemini Pro | 14. Cervical cancer screening is typically not recommended yearly due to the potential risks associated with over diagnosing and over treating. Regular screening at recommended intervals, usually every 3 to 5 years, based on age and individual risk factors, is sufficient to detect most precancerous and cancerous changes in the cervix. | A | A | A |
| Mistral-7B-v0.2 (With prompt) | 14. Yearly cervical cancer screening is not recommended because: a) Most cervical cancers develop slowly, and since regular screenings can detect abnormalities before they become cancer, annual testing is not necessary; and b) Over-diagnosis and unnecessary treatments may result from frequent screenings. Sticking to the recommended intervals of at least every 3 years for Pap tests or every 5 years for co-tests for women between the ages of 21 and 65 reduces the risks associated with frequent screenings. | A | A | A |
| Mistral-7B-v0.2 | 14. Yearly cervical cancer screening is not recommended because most women do not develop cervical cancer, and frequent testing can lead to unnecessary procedures, anxiety, and increased healthcare costs. Regular screening according to current recommendations, which vary by age, is sufficient for detecting precancerous changes and early cancer. | A | A | A |
| Starling-LM-7B alpha (With prompt) | 14. Yearly cervical cancer screening is not recommended because it can lead to over-diagnosis and over-treatment. Cervical cancer is a slow-growing disease, and most abnormalities detected during screening regress spontaneously. More frequent screening can result in the detection of low-grade lesions that may not progress to high-grade lesions or invasive cancer, leading to unnecessary treatments and their associated complications. | A | A | A |
| Starling-LM-7B alpha | 14. Yearly cervical cancer screening is not recommended because cervical cancer is a slow-growing cancer that may take several years to progress. Therefore, screening too frequently can lead to unnecessary interventions and overtreatment. | A | A | A |
| HuatuoGPT (With prompt) | 14. Annual cervical cancer screening is not recommended for women between the ages of 21 and 49 years because cervical cytologic changes are usually mild in this age group and HPV testing has limited effectiveness in screening. | A | A | A |
| HuatuoGPT | 14. Annual cervical cancer screening is not recommended because of the low incidence of cervical cancer and the low likelihood of cervical cancer occurring in the year between annual screenings. In addition, annual screening is no more effective in detecting cervical cancer in low-risk women than cytology every three years. | B | B | B |
| BioMedLM 2.7B (With prompt) | 14. why yearly cervical cancer screening is not recommended? The answer to this question is that the current guidelines are based on the results of randomized controlled trials (RCTs) that have been conducted in the past. The results of these RCTs have been published in the past decade. The guidelines are based on the results of these RCTs. | C | D | D |
| BioMedLM 2.7B | 14. why yearly cervical cancer screening is not recommended? Yearly cervical cancer screening is not recommended. | D | D | D |
| Microsoft Phi2 (With prompt) | 14. Yearly cervical cancer screening is not recommended because the risk of developing cervical cancer is relatively low, and the benefits of screening are not always clear. Additionally, screening can lead to false-positive results, which can cause unnecessary anxiety and lead to further testing and treatment. | A | A | A |
| Microsoft Phi2 | 14. Yearly cervical cancer screening is not recommended because the benefits of screening in this age group are not yet established. The decision to screen should be based on individual risk factors and the potential benefits and harms of screening. | D | C | C |
| QiZhenGPT (With prompt) | 14. yearly cervical cancer screening is not recommended because it does not provide adequate protection against the development of cervical cancer. The recommended screening interval for women aged 25 to 75 is 5 years, and for women aged 76 and above, it is 10 years. | C | D | D |
| QiZhenGPT | 14. yearly cervical cancer screening is not recommended because it does not provide enough protection against the disease. The risk of developing cervical cancer increases with age, and yearly screening does not provide enough time for the virus to spread and develop into a cancer. | C | C | C |
| **15.What are the objectives of cervical cancer screening protocols?** | | | | |
| ChatGPT4.0 turbo (With prompt) | 15. **Objectives of Cervical Cancer Screening Protocols**: The primary objectives of cervical cancer screening protocols are to prevent cervical cancer by detecting precancerous lesions early and to reduce the incidence and mortality associated with cervical cancer. Screening aims to identify and treat these pre-cancerous changes before they progress to invasive cancer, thereby decreasing the overall burden of cervical cancer in the population. The protocols are designed to maximize benefits, minimize harm, and utilize healthcare resources efficiently. | A | A | A |
| ChatGPT4.0 turbo | 15. **Objectives of Cervical Cancer Screening Protocols**:  - **Early Detection**: To detect precancerous changes or early-stage cancer before symptoms appear, when the condition is most treatable.  - **Prevention**: To prevent cervical cancer by identifying and treating precancerous lesions before they progress to invasive cancer.  - **Reduction of Mortality and Morbidity**: To decrease the mortality rate associated with cervical cancer through early intervention.  - **Efficient Use of Resources**: To maximize health benefits while minimizing unnecessary interventions and costs by using evidence-based screening intervals and methods.  These objectives guide the development of screening guidelines and ensure that cervical cancer screening is both effective and efficient, balancing benefits with potential harms. | A | A | A |
| Claude 2 (With prompt) | 15. The objectives are to detect and treat high-grade cervical dysplasia caused by HPV infection before possible progression to invasive cancer, in order to reduce cervical cancer rates and mortality. This is achieved through regular screening allowing for early intervention.  As an experienced gynecologist, I would respond: | A | A | A |
| Claude 2 | 15. Cervical cancer screening protocols aim to detect and facilitate early treatment of precancerous lesions and early stage asymptomatic cancers in women. This prevents development of advanced invasive disease and reduces cervical cancer mortality while minimizing potential harms from overscreening. | A | A | A |
| Gemini Pro (With prompt) | 15. What are the objectives of cervical cancer screening protocols? The objectives of cervical cancer screening protocols are:  Early detection of precancerous lesions: This allows for timely treatment and prevention of cervical cancer.  Reduction in cervical cancer mortality: Regular screening has significantly reduced cervical cancer mortality rates over the past few decades.  Improved survival rates: Early detection and treatment lead to improved survival rates for women diagnosed with cervical cancer. Therefore, cervical cancer screening protocols play a crucial role in protecting women's health. | A | A | A |
| Gemini Pro | 15. The objectives of cervical cancer screening protocols are:  - Early detection and prevention: Regular screening helps identify precancerous and cancerous changes, allowing for timely treatment and cancer prevention.  - Risk assessment: Screening results not only indicate whether the cervix is normal or abnormal but also provide information about a woman's risk for developing cervical cancer in the future.  - Education and awareness: Cervical cancer screening encourages women to learn about their reproductive health, including risk factors, symptoms, and preventive measures like HPV vaccination and safe sex practices. | A | A | A |
| Mistral-7B-v0.2 (With prompt) | 15. The objectives of cervical cancer screening protocols include: a) Early detection and prevention of cervical cancer: By identifying precancerous changes, treatment can be initiated to prevent the progression of cancer, b) Early diagnosis of cervical cancer: Regular cervical cancer screening helps detect cancer at an early stage, when treatment is more effective, and c) Reduction of healthcare costs: Early detection and treatment of cervical cancer result in significant cost savings by avoiding the expenses associated with advanced stages of the disease. | A | A | A |
| Mistral-7B-v0.2 | 15. The objectives of cervical cancer screening protocols are to detect precancerous changes in the cervix before they progress to cancer, reduce the incidence and mortality of cervical cancer, and provide appropriate follow-up care and treatment for women with abnormal results. Regular cervical cancer screening is an essential component of women's health care and can save lives by preventing cervical cancer and identifying it at an early, treatable stage. | A | A | A |
| Starling-LM-7B alpha (With prompt) | 15. The objectives of cervical cancer screening protocols are to:  - Detect early-stage cervical cancer and high-grade precancerous lesions to allow for timely treatment and prevention of invasive cancer. - Identify women with persistent HPV infections that may require closer monitoring or treatment. - Educate women about the importance of regular screening, the role of HPV in cervical cancer development, and the significance of early detection and treatment. - Reduce the incidence and mortality of cervical cancer through early detection and prevention strategies, such as vaccination against HPV and smoking cessation. | A | A | A |
| Starling-LM-7B alpha | 15. The objectives of cervical cancer screening protocols are to detect cervical cancer or its precursors at an early stage, when treatment is most effective, and to reduce the incidence and mortality of cervical cancer. | A | A | A |
| HuatuoGPT (With prompt) | 15. The goal of cervical cancer screening protocols is to detect cervical cancer as early as possible and provide preventive care for women at high risk while avoiding unnecessary interventions and worry. | A | A | A |
| HuatuoGPT | 15. The goal of cervical cancer screening protocols is to detect cervical cancer at an early stage and treat it before it progresses to cancer. The goal of screening is to diagnose and treat cervical cancer before it becomes cancerous in order to reduce cervical cancer morbidity and mortality. Screening may also include HPV testing to identify those at highest risk and provide them with preventive care. | A | A | A |
| BioMedLM 2.7B (With prompt) | 15. What are the objectives of cervical cancer screening protocols? The objectives of cervical cancer screening protocols are to detect precancerous lesions and early invasive cervical cancer. The screening tests are designed to detect precancerous lesions and early invasive | A | B | A |
| BioMedLM 2.7B | 15. What are the objectives of cervical cancer screening protocols? The objectives of cervical cancer screening protocols are to detect precancerous lesions and to reduce the incidence of cervical cancer. The incidence of cervical cancer is reduced by detecting precancerous lesions and treating them. | A | A | A |
| Microsoft Phi2 (With prompt) | 15. The objectives of cervical cancer screening protocols are to detect precancerous lesions or early-stage cervical cancer, which can be treated effectively before they progress to more advanced stages. | A | A | A |
| Microsoft Phi2 | 15. The objectives of cervical cancer screening protocols are to detect precancerous changes or abnormalities in the cervix early, when they are most treatable. By identifying these changes, healthcare providers can intervene and prevent the development of cervical cancer. | A | A | A |
| QiZhenGPT (With prompt) | 15. The objectives of cervical cancer screening protocols are to detect and diagnose cervical cancer at an early stage, to prevent its development into advanced stages, and to reduce the burden of disease on healthcare systems. These goals can be achieved through a combination of cytological screening, HPV testing, and other forms of risk assessment and management. | A | A | A |
| QiZhenGPT | 15. The objectives of cervical cancer screening protocols are to reduce the risk of developing cervical cancer by identifying women who are at risk of the disease and providing them with regular screening and early detection. This can help to prevent the spread of the disease and reduce the number of cases that occur. The goal of these protocols is also to improve the quality of life for women with cervical cancer by providing them with support and treatment. | A | A | A |
| **16.Is cervical cancer hereditary? If so, should individuals with a familial history of cervical cancer be subject to more frequent screening protocols?** | | | | |
| ChatGPT4.0 turbo (With prompt) | 16. **Hereditary Nature of Cervical Cancer**: Cervical cancer is generally not considered hereditary. Most cases of cervical cancer are caused by persistent infection with high-risk types of Human Papillomavirus (HPV), which is acquired through sexual contact. There is no current evidence to suggest that a family history of cervical cancer significantly increases an individual's risk in a hereditary manner. Therefore, individuals with a familial history of cervical cancer are not typically advised to undergo more frequent screening than the general population, unless other risk factors are present. | A | B | B |
| ChatGPT4.0 turbo | 16. **Hereditary Nature of Cervical Cancer**:  - Cervical cancer is not typically considered hereditary. Most cases of cervical cancer are caused by persistent infection with high-risk types of HPV, which are acquired through sexual contact. However, having a family history of cervical cancer might reflect shared lifestyle or environmental factors that increase risk, or less commonly, a genetic predisposition to HPV persistence or progression to cancer.  - **Screening for Individuals with a Family History**: Generally, individuals with a family history of cervical cancer are not recommended to follow more frequent screening protocols unless other risk factors are present. The standard screening guidelines should suffice. | A | A | A |
| Claude 2 (With prompt) | 16. Cervical cancer is not directly inherited genetically, however having a first-degree relative with cervical cancer can be a risk factor. For these higher-risk individuals, beginning screening 5 years earlier than age 25 is appropriate (e.g. age 20-21). However, screening frequency does not need to be more than every 3 years in accordance with guidelines. | A | B | B |
| Claude 2 | 16. Cervical cancer is not generally considered a hereditary form of cancer. However, some subtypes may have hereditary tendencies. Those with a first-degree relative with cervical cancer may start screening earlier, but intervals are the same. | C | B | B |
| Gemini Pro (With prompt) | 16. Is cervical cancer hereditary? If so, should individuals with a familial history of cervical cancer be subject to more frequent screening protocols? Cervical cancer is not directly hereditary, but there can be a genetic predisposition.  Family history as a risk factor: Women with a family history of cervical cancer have a slightly increased risk of developing the disease themselves.  Genes and HPV infection: Certain genes may influence the body's ability to clear HPV infection, which is the primary cause of cervical cancer.  Screening recommendations: Individuals with a strong family history of cervical cancer may be advised to start screening at an earlier age or undergo more frequent screenings. | A | B | B |
| Gemini Pro | 16. While cervical cancer is not strictly hereditary, having a family history of the disease does increase one's risk. Women with a familial history of cervical cancer should discuss their risk with their healthcare provider. They may be advised to start screening at an earlier age, have more frequent screenings, or undergo more extensive testing. | B | B | B |
| Mistral-7B-v0.2 (With prompt) | 16. Yes, there is a familial risk for cervical cancer, particularly when there is a first-degree relative (mother, sister, or daughter) with a history of cervical cancer. These individuals have an increased risk for developing cervical cancer. They may benefit from more frequent screenings, starting at a younger age, in consultation with their healthcare provider. Hungerford Study showed that women with a first-degree family member with cervical cancer should begin cervical screening 10 years before the age at which their relative was diagnosed or at the age of 21, whichever is earlier. | C | D | C |
| Mistral-7B-v0.2 | 16. Yes, having a familial history of cervical cancer can increase an individual's risk for developing the disease, but the majority of cases are caused by HPV infection. Individuals with a familial history of cervical cancer should be aware of their risk and follow recommended screening guidelines. However, there is no consensus on whether they should undergo more frequent screening, and this is a topic of ongoing research. Women with a familial history of cervical cancer should discuss their risk with their healthcare provider and consider any additional risk factors they may have, such as smoking or a history of multiple sexual partners. | B | B | B |
| Starling-LM-7B alpha (With prompt) | 16. Cervical cancer is not primarily hereditary, but it is associated with certain genetic factors and family history. While individuals with a familial history of cervical cancer may have an increased risk, it does not necessarily warrant more frequent screening protocols. The American College of Obstetricians and Gynecologists (ACOG) recommends that women at average risk of cervical cancer should undergo cervical cancer screening with a Pap test every 3 years starting at age 21, regardless of family history. | A | B | B |
| Starling-LM-7B alpha | 16.Cervical cancer is not typically considered hereditary. It is primarily caused by persistent infection with high-risk strains of the Human Papillomavirus (HPV). While some cancers have a strong genetic or familial component, cervical cancer does not follow this pattern. | B | B | B |
| HuatuoGPT (With prompt) | 16.No, cervical cancer is not hereditary. While there are some genetic factors that increase the risk of developing cervical cancer, most cases are due to HPV infection. | A | A | A |
| HuatuoGPT | 16. No, cervical cancer is not hereditary. Although people with a family history of cancer have a slightly higher risk of developing cervical cancer, these increased risks are primarily due to HPV infections associated with cervical cancer. | A | A | A |
| BioMedLM 2.7B (With prompt) | 16.Is cervical cancer hereditary? If so, should individuals with a familial history of cervical cancer be subject to more frequent screening protocols? Yes, cervical cancer is hereditary. The risk of cervical cancer is increased in women with a family history of cervical cancer. | D | D | D |
| BioMedLM 2.7B | 16.Is cervical cancer hereditary? If so, should individuals with a familial history of cervical cancer be subject to more frequent screening protocols?Yes. | D | D | D |
| Microsoft Phi2 (With prompt) | 16. Cervical cancer is not hereditary, but some individuals may have a familial history of cervical cancer that puts them at a higher risk of developing the disease. If an individual has a family history of cervical cancer, they may be advised to undergo more frequent screening protocols to detect the disease at an early stage. | A | B | B |
| Microsoft Phi2 | 16. Yes, cervical cancer can be hereditary. Individuals with a familial history of cervical cancer should be subject to more frequent screening protocols. | D | D | D |
| QiZhenGPT (With prompt) | 16. Is cervical cancer hereditary? If so, should individuals with a familial history of cervical cancer be subject to more frequent screening protocols? Answer: Yes, cervical cancer is hereditary. Individuals with a familial history of cervical cancer may benefit from more frequent screening protocols. | D | D | D |
| QiZhenGPT | 16. Yes, cervical cancer is hereditary. Individuals with a family history of cervical cancer may be recommended to undergo more frequent screening protocols to detect the disease early. | D | C | D |
| **17.What's the difference between a pelvic exam and a Pap test?** | | | | |
| ChatGPT4.0 turbo (With prompt) | 17. **Difference Between a Pelvic Exam and a Pap Test**: A pelvic exam and a Pap test are often performed during the same visit but serve different purposes. A pelvic exam involves a physical examination of the internal and external genital organs (including the uterus, cervix, ovaries, fallopian tubes, and vagina) to check for any abnormalities or signs of disease. A Pap test, on the other hand, specifically involves collecting cells from the cervix to look for precancerous or cancerous changes. The Pap test is focused on cervical health, while the pelvic exam assesses overall reproductive health. | A | A | A |
| ChatGPT4.0 turbo | 17. **Difference Between a Pelvic Exam and a Pap Test**:  - **Pelvic Exam**: This is a physical examination where a healthcare provider visually and manually assesses the female reproductive organs (vagina, cervix, ovaries, uterus, and fallopian tubes). It is used to check for abnormalities or infections and assess general reproductive health.  - **Pap Test**: This is a specific test performed during a pelvic exam, where cells from the cervix are collected and examined under a microscope to detect precancerous or cancerous changes. It specifically screens for cervical cancer and is not a general assessment of reproductive health. | A | A | A |
| Claude 2 (With prompt) | 17. A pelvic exam involves physical examination of the vulva, vagina, cervix, uterus, ovaries and rectum. A Pap test (Pap smear) involves collection of cells from the cervix to prepare a slide for microscopic examination, looking specifically for precancerous changes. | A | A | A |
| Claude 2 | 17. A pelvic exam is a physical examination of the female pelvic organs. A Pap test (Pap smear) collects cells from the cervix to examine under a microscope for abnormalities indicating precancerous or cancerous changes. Pelvic exams may detect some cancers, but do not screen for early abnormalities like Pap tests. | A | A | A |
| Gemini Pro (With prompt) | 17. What's the difference between a pelvic exam and a Pap test? A pelvic exam and a Pap test are two different procedures used in cervical cancer screening.  Pelvic exam: A physical examination of the female reproductive organs, including the cervix, uterus, and ovaries. It allows the doctor to check for any abnormalities in size, shape, or texture.  Pap test: A procedure where cells are collected from the cervix and examined under a microscope to detect precancerous or cancerous changes. | A | A | A |
| Gemini Pro | 17. A pelvic exam and a Pap test are related, but they are not the same procedure. A pelvic exam is an examination of the external female genitalia, vagina, cervix, and uterus, often to check for other conditions like sexually transmitted infections, fibroids, or ovarian masses. A Pap test is a specific part of a pelvic exam, where a healthcare professional takes a sample of cells from the cervix to test for precancerous and cancerous changes. | A | A | A |
| Mistral-7B-v0.2 (With prompt) | 17. A pelvic exam and a Pap test are related procedures that are often performed together but serve different purposes: a) A pelvic exam is a comprehensive examination of the female reproductive organs, including a visual inspection of the cervix, vagina, and external genitals, to assess overall health and identify potential issues unrelated to cervical cancer, such as sexually transmitted infections. b) A Pap test, also referred to as a Pap smear, is a cytological examination of cells collected from the cervix, used to detect precancerous or cancerous cells. | A | A | A |
| Mistral-7B-v0.2 | 17. A pelvic exam and a Pap test are related but distinct procedures. A pelvic exam is a comprehensive examination of the female reproductive system, including the external genitalia, cervix, vagina, and uterus. This exam is typically performed to assess the health of these organs and evaluate for any abnormalities. A Pap test is a specific test performed during a pelvic exam to collect cells from the cervix for analysis. The Pap test is used to detect cervical cell abnormalities, including precancerous changes and cancer. | A | A | A |
| Starling-LM-7B alpha (With prompt) | 17. A pelvic exam is a physical examination of the female reproductive organs, including the vagina, cervix, uterus, ovaries, and fallopian tubes. It is typically performed to check for abnormalities, infections, or signs of disease. A Pap test, on the other hand, is a specific test that collects cells from the cervix to be examined under a microscope for signs of cervical cancer or precancerous changes. While both exams are often performed together during a routine gynecological visit, they serve different purposes. | A | A | A |
| Starling-LM-7B alpha | 17. The pelvic exam is a manual examination of the female reproductive organs, including the cervix, uterus, ovaries, and fallopian tubes. The Pap test, on the other hand, is a screening test that checks for changes in the cervical cells that may be precancerous or cancerous. | A | A | A |
| HuatuoGPT (With prompt) | 17. A pelvic exam and a pap smear are two different tests. Pelvic examination involves a physical examination of the vagina and uterus, while pap smear involves the collection of cell samples from the cervix for microscopic examination. | A | A | A |
| HuatuoGPT | 17. Pelvic exam is a comprehensive pelvic exam performed by a doctor that includes an examination of the vagina, uterus and ovaries. A pap smear is a small sample of cells collected from the cervix by a doctor using a special brush to check for the presence of abnormal cells. | A | B | A |
| BioMedLM 2.7B (With prompt) | 17. What's the difference between a pelvic exam and a Pap test? The pelvic exam is a physical examination of the female reproductive organs. It is performed by a physician or a nurse practitioner. The pelvic exam is used to assess the size, shape, and consistency of the uterus, cervix, and vagina. It is also used to assess the presence of any masses, discharge, or bleeding. The pelvic exam is used to assess the presence of sexually transmitted infections (STIs) and to determine if a woman is pregnant. The pelvic exam is also used to assess the presence of any abnormalities in the ovaries, fallopian tubes, and uterus. The pelvic exam is used to assess the presence of any abnormalities in the ovaries, fallopian tubes, and uterus. | A | A | A |
| BioMedLM 2.7B | 17. What's the difference between a pelvic exam and a Pap test? Pelvic exams are performed to assess the size and shape of the uterus and ovaries, and to determine whether there are any abnormalities. Pap tests are performed to assess the presence of abnormal cells in the cervix. | A | A | A |
| Microsoft Phi2 (With prompt) | 17. A pelvic exam is a physical examination of the female reproductive system, including the cervix, uterus, and ovaries. A Pap test is a screening test that is used to detect changes in the cells of the cervix that may indicate the presence of precancerous changes or cervical cancer. During a Pap test, a sample of cells is collected from the cervix and examined under a microscope to look for abnormal changes. | A | A | A |
| Microsoft Phi2 | 17. A pelvic exam involves a health care provider examining the external genitals, the vagina, and the internal reproductive organs through a pelvic examination. A Pap test involves collecting cells from the cervix to be examined for abnormalities that may indicate the presence of cervical cancer or other precancerous conditions. | A | A | A |
| QiZhenGPT (With prompt) | 17. What's the difference between a pelvic exam and a Pap test? Answer: A pelvic exam is a comprehensive exam that includes a pelvic examination, a Pap test is a screening test that measures the presence of abnormal cells on a sample of the skin taken during a pelvic exam. | A | A | A |
| QiZhenGPT | 17. A pelvic exam is a physical examination that involves the examination of the reproductive organs, including the uterus, fallopian tubes, and ovaries. A Pap test, on the other hand, is a screening test that uses a small sample of cells from the skin to check for abnormal cells that may indicate the presence of cervical cancer. | A | A | A |
| **18.Can individuals independently administer HPV tests, and if so, how accurate are these self-administered test?** | | | | |
| ChatGPT4.0 turbo (With prompt) | 18. **Self-Administered HPV Tests**: Yes, self-administered HPV tests are available and can be an alternative to HPV testing done in a clinical setting. These tests involve individuals collecting their own sample using a swab and sending it to a laboratory for analysis. The accuracy of self-administered HPV tests can be comparable to that of tests performed by healthcare professionals, provided that the instructions are followed carefully. However, it's important to note that self-testing may not be appropriate for everyone and does not replace the need for professional medical advice and follow-up. | A | A | A |
| ChatGPT4.0 turbo | 18. **Self-Administered HPV Tests**:  - Yes, self-administered HPV tests are available and can be a viable option, especially in settings where women have limited access to healthcare services. These tests involve using a swab to collect a sample from one’s vagina, which is then sent to a lab for analysis.  - **Accuracy**: Studies have shown that self-collected samples for HPV testing can be nearly as accurate as those collected by healthcare providers. However, the effectiveness can depend on the correct collection of the sample. | A | A | A |
| Claude 2 (With prompt) | 18. HPV tests are not currently approved for at-home self-administration. Professionally-administered HPV tests done alongside Pap tests in women over 30 offer improved sensitivity for detecting cervical precancer and cancer. | C | D | C |
| Claude 2 | 18. Yes, HPV tests that test vaginal/cervical cells for HPV are available for home use. However, they are currently less accurate overall compared to provider-administered screening. A positive home HPV test requires follow-up confirmation. At-home tests do not screen for precancerous cell changes. | C | C | C |
| Gemini Pro (With prompt) | 18. Can individuals independently administer HPV tests, and if so, how accurate are these self-administered tests?  Yes, there are self-administered HPV tests available.   Accuracy: Self-administered HPV tests are highly accurate when used correctly.  Instructions and training: Individuals should carefully follow the instructions provided with the test and may need training on how to collect the sample.  Interpretation: Results should be interpreted by a healthcare professional. | B | A | A |
| Gemini Pro | 18. Individuals cannot independently administer HPV tests. HPV tests require a clinician to collect cervical or vaginal cells to determine the presence of the virus. Self-administered HPV tests are available, but they are generally not as accurate as tests performed in clinical settings. | A | A | A |
| Mistral-7B-v0.2 (With prompt) | 18. HPV tests can be administered at home using a self-collection kit, though their accuracy might be lower than that of clinician-administered tests. The self-administered tests require users to follow a specific set of instructions, often with visual aids, to correctly collect the required sample. Inaccuracies may arise from improper self-collection techniques. For usual diagnosis and follow-up, a healthcare professional's guidance is recommended. | A | A | A |
| Mistral-7B-v0.2 | 18. Individuals cannot independently administer HPV tests. HPV testing relies on a provider to collect and analyze the samples, ensuring the accuracy and integrity of the test. Self-sampling for HPV testing is an area of ongoing research, but current tests have not been approved for self-administered use. | A | A | A |
| Starling-LM-7B alpha (With prompt) | 18. Individuals can independently administer HPV self-sampling tests, which involve collecting cells from the cervix using a small brush or swab. These tests have been shown to be highly accurate, with sensitivity and specificity rates comparable to clinician-collected samples. However, it is important to note that self-sampling tests should still be performed under the guidance of a healthcare professional to ensure proper technique and sample collection. | A | A | A |
| Starling-LM-7B alpha | 18. Yes, individuals can independently administer HPV tests using self-collection kits. These kits are designed to be easy to use and provide accurate results. However, it is important to follow the instructions carefully and return the sample to a laboratory for testing. | A | A | A |
| HuatuoGPT (With prompt) | 18. Yes, HPV testing can be done by the patient themselves, but it must be done by a trained and authorized healthcare professional. The accuracy of pap smears and HPV tests may vary depending on the type of test and individual factors. | B | B | B |
| HuatuoGPT | 18. Yes, individuals can perform their own HPV tests. The accuracy of a self-administered HPV test varies from individual to individual, but studies have shown that when properly instructed, it works as well as a test performed by a doctor. However, self-administered HPV testing should only be performed by a trained and qualified professional or under the supervision of a healthcare professional. | B | B | B |
| BioMedLM 2.7B (With prompt) | 18. Can individuals independently administer HPV tests, and if so, how accurate are these self-administered test? HPV testing is a sensitive and specific test for the detection of high-grade cervical intraepithelial neoplasia (CIN) and cervical cancer. Self-collection of samples for HPV testing is an alternative to physician-collected samples. | B | C | C |
| BioMedLM 2.7B | 18. Can individuals independently administer HPV tests, and if so, how accurate are these self-administered test? Yes. | C | C | C |
| Microsoft Phi2 (With prompt) | 18. Yes, individuals can independently administer HPV tests, and these self-administered tests are highly accurate. | C | B | B |
| Microsoft Phi2 | 18. Yes, individuals can independently administer HPV tests. These self-administered tests involve collecting a sample from the cervix using a swab or brush. The accuracy of these self-administered tests is comparable to those performed by healthcare professionals. | D | D | D |
| QiZhenGPT (With prompt) | 18. Can individuals independently administer HPV tests, and if so, how accurate are these self-administered test? Answer: Yes, individuals can independently administer HPV tests. These self-administered tests are accurate, but it is recommended to have a healthcare provider administer the test to ensure accuracy. | B | A | A |
| QiZhenGPT | 18. Yes, individuals can independently administer HPV tests. These self-administered tests are used to check for the presence of abnormal cells in the skin, which can indicate the presence of cervical cancer. However, it is important to note that these tests are not as accurate as laboratory-based tests, and it is recommended to have a healthcare provider administer the test. | D | C | C |
| **19.Is it possible for cervical cancer to manifest within the inter-screening interval, particularly between two consecutive cervical screening tests?** | | | | |
| ChatGPT4.0 turbo (With prompt) | 19. **Manifestation of Cervical Cancer Within the Inter-Screening Interval**: Yes, it is possible for cervical cancer to develop within the inter-screening interval, particularly if high-risk HPV infections persist and cause rapid progression of precancerous changes. This risk, however, is relatively low, especially when screening intervals are adhered to based on current guidelines. Regular screening is designed to catch precancerous changes early enough to prevent most cases of cervical cancer, but no screening test is perfect, and cases can occasionally develop between screenings. | A | A | A |
| ChatGPT4.0 turbo | 19. **Manifestation of Cervical Cancer Within Inter-Screening Interval**:  - Yes, it is possible, though relatively rare, for cervical cancer to develop within the inter-screening interval. This can happen if a high-grade lesion or HPV infection progresses more rapidly than usual, or if an abnormality was missed in a previous screening. This underscores the importance of adhering to recommended screening intervals and promptly reporting any unusual symptoms to a healthcare provider. | A | A | A |
| Claude 2 (With prompt) | 19. Unfortunately yes, it is possible for rapidly-progressing cervical cancer to develop in between regular screening intervals. However, this occurs in a very small proportion of cases. This risk is reduced by adherence to routine screening guidelines. | B | C | B |
| Claude 2 | 19. It is possible but very rare for invasive cancer to develop in the intervals between regular screenings. Pre-cancerous changes often take 5-10 years before progressing to true cancer. Intervals aim to balance benefits and harms for the population. | A | A | A |
| Gemini Pro (With prompt) | 19. Is it possible for cervical cancer to manifest within the inter-screening interval, particularly between two consecutive cervical screening tests? Yes, it is possible for cervical cancer to develop within the inter-screening interval.  Slow-growing cancers: Some cervical cancers develop slowly and may not be detected by screening tests performed at the recommended intervals.  Importance of regular screening: Regular screening is essential to detect cancers that develop between screenings.  Additional risk factors: Individuals with additional risk factors for cervical cancer may need more frequent screenings. | C | C | C |
| Gemini Pro | 19. Yes, it's possible for cervical cancer to manifest within the inter-screening interval, particularly between two consecutive cervical screening tests. While regular screening reduces the risk of developing cervical cancer, there is still a chance that cancer can occur between screening tests. It is essential to maintain a healthy lifestyle, follow up on abnormal results, and be aware of symptoms to increase the chances of early detection and treatment. | A | A | A |
| Mistral-7B-v0.2 (With prompt) | 19. Yes, it is possible for cervical cancer to manifest within the inter-screening interval. However, regular screenings significantly decrease the likelihood of developing advanced-stage cervical cancer between consecutive tests. The American Cancer Society reports that about 11% of cervical cancers occur within 5 years of the last normal screening test, and approximately 22% of cervical cancers are detected between screenings. | A | A | A |
| Mistral-7B-v0.2 | 19. Yes, it is possible for cervical cancer to manifest within the inter-screening interval, especially in women with risk factors such as a history of abnormal Pap tests, HPV infection, smoking, or a weakened immune system. Regular follow-up care and communication with a healthcare provider are essential for women with abnormal results or risk factors to ensure that any changes are detected and addressed promptly. | A | A | A |
| Starling-LM-7B alpha (With prompt) | 19. Yes, it is possible for cervical cancer to manifest within the inter-screening interval, particularly between two consecutive cervical screening tests. This is why it is essential for women to be vigilant about reporting any abnormal symptoms or changes in their bodies between screenings. Regular follow-up and communication with a healthcare provider are crucial in detecting and managing cervical cancer early. | A | A | A |
| Starling-LM-7B alpha | 19. Yes, it is possible for cervical cancer to manifest within the inter-screening interval, particularly between two consecutive cervical screening tests. Regular screening is important for early detection and treatment of cervical cancer, but it is not a guarantee against the development of the disease. | A | A | A |
| HuatuoGPT (With prompt) | 19. Yes, in some cases, cervical cancer may appear between cervical screenings. However, the likelihood is relatively small and routine single screening is not sufficient to detect it. | C | C | C |
| HuatuoGPT | 19.Yes, cervical cancer can develop between screenings, especially after an HPV infection. However, this is uncommon because it usually takes a long time for cervical cancer to develop. | A | A | A |
| BioMedLM 2.7B (With prompt) | 19. Is it possible for cervical cancer to manifest within the inter-screening interval, particularly between two consecutive cervical screening tests? Yes, it is possible for cervical cancer to manifest within the inter-screening interval, particularly between two consecutive cervical screening tests. The interval between two consecutive cervical screening tests is recommended to be at least 5 years. The interval between two consecutive cervical screening tests is recommended to be at least 5 years. | B | A | A |
| BioMedLM 2.7B | 19. Is it possible for cervical cancer to manifest within the inter-screening interval, particularly between two consecutive cervical screening tests? Yes, cervical cancer can manifest within the inter-screening interval, particularly between two consecutive cervical screening tests. | A | A | A |
| Microsoft Phi2 (With prompt) | 19. Yes, it is possible for cervical cancer to manifest within the inter-screening interval, particularly between two consecutive cervical screening tests. | B | B | B |
| Microsoft Phi2 | 19. Yes, it is possible for cervical cancer to manifest within the inter-screening interval. This means that abnormalities or precancerous changes may develop between two consecutive cervical screening tests, even if the tests were performed within the recommended time frame. | A | A | A |
| QiZhenGPT (With prompt) | 19. Is it possible for cervical cancer to manifest within the inter-screening interval, particularly between two consecutive cervical screening tests? Answer: Yes, it is possible for cervical cancer to manifest within the inter-screening interval. This is because the Pap test is a screening test, and if a person has already been screening regularly, they may still develop cervical cancer during the inter-screening interval. | B | A | A |
| QiZhenGPT | 19. Yes, cervical cancer can manifest within the inter-screening interval, particularly between two consecutive cervical screening tests. This means that if an individual has already undergone two consecutive cervical screening tests and does not have any abnormal cells on the third test, they may still be at risk of developing cervical cancer. | A | A | A |
| **20.Is cervical screening necessary for individuals who have reached menopause?** | | | | |
| ChatGPT4.0 turbo (With prompt) | 20. **Cervical Screening Post-Menopause**: Yes, cervical screening is necessary for individuals who have reached menopause, up to the age of 65, provided they have not met the criteria for discontinuation (such as having a history of adequate prior negative screenings and not being at high risk for cervical cancer). Cervical cancer can still occur in postmenopausal women, and continuing screening as recommended can help ensure that any late-developing precancerous changes are detected and managed appropriately. | A | A | A |
| ChatGPT4.0 turbo | 20. **Cervical Screening Post-Menopause**:  - Cervical screening is still necessary for individuals who have reached menopause, up to the age of 65, as long as they meet the criteria for screening. This includes those who have not had adequate prior screening or those who have had recent abnormal results.   - Women over 65 who have had consistent normal screening results or adequate prior screening and are not at high risk for cervical cancer may discontinue screening. However, those with a history of high-grade precancerous lesions or cervical cancer should continue screening as directed by their healthcare provider. | A | A | A |
| Claude 2 (With prompt) | 20. Yes cervical screening is still recommended for most women who have gone through menopause, unless they meet specific low-risk criteria to discontinue (e.g. consecutive negative prior screens, no dysplasia history, total hysterectomy). HPV infection and abnormal cell changes can occur even in older, postmenopausal women. Screening allows precancer identification. | A | B | B |
| Claude 2 | 20. Cervical cancer screening can generally be discontinued after age 65-70 for those with a history of adequate prior negative screening results. However, women with risk factors like DES exposure may need continued screening. Those with severe immunosuppression or a previous positive screen may also require individualized ongoing screening. | C | B | B |
| Gemini Pro (With prompt) | 20. Is cervical screening necessary for individuals who have reached menopause? Cervical cancer screening may still be necessary for individuals who have reached menopause.  Prior screening history: Women with a history of abnormal Pap tests or cervical cancer may need continued screening.  Age and risk factors: Women over 65 with adequate prior negative screenings and no additional risk factors may discontinue screening.  Individualized decision: The decision to continue screening after menopause should be made in consultation with a healthcare professional. | B | C | C |
| Gemini Pro | 20. Yes, cervical screening is still necessary for individuals who have reached menopause. The recommended screening intervals may change depending on age, characteristics, and previous screening history. Women over 65 may be able to stop cervical cancer screening or switch to less frequent screenings based on their individual risk, but it's essential to consult with a healthcare professional to determine the most appropriate screening schedule. | A | A | A |
| Mistral-7B-v0.2 (With prompt) | 20. Yes, cervical cancer screening is necessary for individuals who have reached menopause, as the incidence of cervical cancer in this population is still significant. Although the risk of cervical cancer is lower in postmenopausal women than in premenopausal women, it is not eliminated entirely. Cervical cancer screening remains crucial for detecting and treating cervical cancer at an early stage, especially since the symptoms may be less apparent or dismissed during menopause. Generally, women in this age group will continue following the same screening guidelines as before menopause (every 3 years for Pap tests or co-tests). | A | A | A |
| Mistral-7B-v0.2 | 20. Cervical screening is recommended for women who have a cervix, including postmenopausal women. Postmenopausal women should continue to follow recommended screening guidelines based on their age and past screening history. Although the risk of cervical cancer decreases after menopause, it does not eliminate the risk entirely. Cervical cancer screening can help detect precancerous changes and cancer at an early, treatable stage, and is an important component of healthcare | A | A | A |
| Starling-LM-7B alpha (With prompt) | 20. Cervical screening is still necessary for postmenopausal women, as the risk of cervical cancer does not completely disappear after menopause. While the screening frequency may decrease with age, it is important for women to continue receiving regular cervical cancer screenings, even after menopause, to detect and manage any potential abnormalities or precancerous changes.  In summary, cervical cancer is not primarily hereditary, but individuals with a family history may have an increased risk. A pelvic exam and a Pap test are different types of examinations, with the Pap test specifically focusing on cervical cancer detection. HPV self-sampling tests can be independently administered and are accurate when performed correctly. Cervical cancer can still develop between screenings, so it is essential for women to report any abnormal symptoms and continue receiving regular screenings even after menopause. | A | A | A |
| Starling-LM-7B alpha | 20. Cervical screening is recommended for individuals who have reached menopause, but the frequency of screening may be reduced. Women aged 65 and older who have had three consecutive negative Pap tests or two consecutive negative HPV tests within the past 10 years, and no abnormal test results in the past 20 years, can discontinue cervical cancer screening. However, it is important to discuss individual risk factors and health history with a healthcare provider to determine the appropriate screening schedule. | A | B | B |
| HuatuoGPT (With prompt) | 20. After entering menopause, cervical cancer screening is no longer usually necessary unless there are specific symptoms or risk factors. However, women who have reached menopause and have been diagnosed with cervical intraepithelial neoplasia (CIN) should continue to be screened until after radical organ surgery. | D | C | C |
| HuatuoGPT | 20. For women entering menopause, the need for cervical cancer screening depends on many factors, including their individual risk. Generally, women who have had a hysterectomy do not need cervical cancer screening, but for women who are still going through menopause, the decision to screen is an individualized one that should take into account personal risk as well as the benefits and risks of screening. | C | B | B |
| BioMedLM 2.7B (With prompt) | 20. Is cervical screening necessary for individuals who have reached menopause? Yes, cervical screening is necessary for women who have reached menopause. | A | A | A |
| BioMedLM 2.7B | 20. Is cervical screening necessary for individuals who have reached menopause? Yes. | A | A | A |
| Microsoft Phi2 (With prompt) | 20. No, cervical screening is not necessary for individuals who have reached menopause. | D | D | D |
| Microsoft Phi2 | 20. Cervical screening is not necessary for individuals who have reached menopause. After menopause, the risk of developing cervical cancer significantly decreases, and the benefits of continued screening may outweigh the potential risks. However, it is important to consult with a healthcare professional to determine the most appropriate screening schedule based on individual circumstances. | D | D | D |
| QiZhenGPT (With prompt) | 20. Is cervical screening necessary for individuals who have reached menopause? Answer: No, cervical screening is not necessary for individuals who have reached menopause. At this stage, the risk of developing cervical cancer is low, and there are other screening methods that can be used to detect cancer in these individuals. | D | D | D |
| QiZhenGPT | 20. cervical screening is necessary for individuals who have reached menopause. As the body begins to lose its ability to produce hormones, it is more likely to develop cervical cancer. Therefore, it is important for these individuals to undergo regular screening to detect the disease early and prevent its progression. | C | C | C |
| **21.Is it recommended to undergo cervical screening during pregnancy?** | | | | |
| ChatGPT4.0 turbo (With prompt) | 21. **Cervical Screening During Pregnancy**: It is generally safe to undergo cervical screening during pregnancy if it is due according to the recommended screening schedule. The best time to perform a Pap smear during pregnancy is during the first trimester, or early in the second trimester. However, if a woman has a history of regular screenings with normal results, the screening can often be postponed until after delivery. If an abnormal result is found during pregnancy, management should be tailored to minimize risks to the fetus while adequately evaluating the mother. Colposcopy is considered safe during pregnancy, but biopsy or treatment procedures for cervical abnormalities are usually postponed until after delivery, unless there is a strong indication of invasive cancer. | A | A | A |
| ChatGPT4.0 turbo | 21. **Cervical Screening During Pregnancy**:  - Cervical screening is generally safe during pregnancy and can be performed up to 24 weeks of gestation. If a Pap test is due when a woman is pregnant, it is usually safe to proceed, as the procedure poses minimal risk to the developing fetus. However, any follow-up procedures that might be required based on abnormal results are often postponed until after delivery, unless there is a pressing concern. | B | B | B |
| Claude 2 (With prompt) | 21. Cervical cancer screening is not recommended during pregnancy. For those with history of adequate prior negative screening, screening can be deferred until 12 weeks postpartum. Those with history of inadequate screening or known dysplasia should undergo colposcopy and cervical biopsy during pregnancy only if invasive cancer is strongly suspected. Otherwise, follow-up can occur postpartum. | A | A | A |
| Claude 2 | 21. Routine cervical cancer screening is not recommended during pregnancy. For those due for screening, it should be delayed until at least 12 weeks postpartum. Screening during pregnancy can lead to false positive results and unnecessary procedures with increased risks like miscarriage or preterm birth. However, screening may be done if clinically indicated for those with symptoms or history of abnormal tests. | A | A | A |
| Gemini Pro (With prompt) | 21. Is it recommended to undergo cervical screening during pregnancy? Cervical cancer screening is not routinely recommended during pregnancy.  Changes in the cervix: During pregnancy, the cervix undergoes changes that can make it difficult to interpret Pap test results accurately.  Low risk of cervical cancer during pregnancy: The risk of developing cervical cancer during pregnancy is very low.  Postpartum screening: Screening can be safely performed after delivery. However, if a woman has a history of abnormal Pap tests or other risk factors for cervical cancer, her doctor may recommend screening during pregnancy. | A | B | B |
| Gemini Pro | 21. Yes, it's recommended to undergo cervical screening during pregnancy. However, Pap tests should ideally be performed before or shortly after pregnancy, as the cervical changes during pregnancy can affect the accuracy of the test results. HPV testing is a more suitable option during pregnancy but may still be influenced by cervical changes. Healthcare professionals usually recommend deferring Pap tests until after delivery but may perform them if there are concerns about high-risk HPV infection or other factors. | C | C | C |
| Mistral-7B-v0.2 (With prompt) | 21. The American College of Obstetrics and Gynecology (ACOG) recommends that women maintain their usual scheduling for cervical cancer screening, including Pap and HPV tests, during pregnancy unless there are specific indications or complications that justify deferring the test. Pregnancy does not increase the risk of false-positive results. If an abnormal result is detected, further evaluation is typically recommended after the delivery to avoid the potential confusion caused by hormonal changes that occur during pregnancy. | C | B | B |
| Mistral-7B-v0.2 | 21. During pregnancy, cervical cancer screening is typically not performed routinely unless there are specific risk factors, such as an abnormal Pap test result before pregnancy or a history of precancerous changes. A pelvic exam during pregnancy is generally only recommended if medically necessary for diagnosing or monitoring other conditions unrelated to cervical cancer. | C | C | C |
| Starling-LM-7B alpha (With prompt) | 21. Is it recommended to undergo cervical screening during pregnancy?  While cervical cancer screening is generally recommended for women aged 21 to 65, it is not typically advised during pregnancy. The reason for this is that the human papillomavirus (HPV) test, which is a crucial part of cervical cancer screening, requires a vaginal swab that may not be safe for the pregnant woman and the developing fetus. However, if a woman is at high risk for cervical cancer, her healthcare provider may recommend a cervical screening during pregnancy. It is essential to consult with a healthcare professional to determine the appropriate course of action. | A | A | A |
| Starling-LM-7B alpha | 21. It is generally not recommended to undergo cervical screening during pregnancy due to the potential risk of miscarriage or premature labor. However, if a pregnant woman has symptoms or a history of abnormal cervical cells, her healthcare provider may recommend screening on a case-by-case basis. | A | A | A |
| HuatuoGPT (With prompt) | 21. Cervical cancer screening during pregnancy is not recommended unless specific symptoms or risk factors are present. However, pregnant women may receive pap smears to monitor for precancerous cervical lesions. | A | A | A |
| HuatuoGPT | 21. Whether cervical screening is recommended during pregnancy depends on a variety of factors, including the individual's risk and the health care provider's recommendations. In general, cervical screening may not be necessary if one is pregnant and does not have high-risk factors, such as HIV infection or certain genetic variants. However, if pregnant and have high risk factors, a healthcare provider may recommend cervical screening for early detection and treatment of cervical cancer. | B | B | B |
| BioMedLM 2.7B (With prompt) | 21. Is it recommended to undergo cervical screening during pregnancy? Yes, it is recommended to undergo cervical screening during pregnancy. | D | D | D |
| BioMedLM 2.7B | 21. Is it recommended to undergo cervical screening during pregnancy? Yes. | D | D | D |
| Microsoft Phi2 (With prompt) | 21. No, cervical screening is not recommended during pregnancy. | A | B | B |
| Microsoft Phi2 | 21. No, it is not recommended to undergo cervical screening during pregnancy. | B | A | A |
| QiZhenGPT (With prompt) | 21. Yes, it is recommended to undergo cervical screening during pregnancy to detect cervical cancer early and prevent its progression to advanced stages. | D | D | D |
| QiZhenGPT | 21. It is generally not recommended to undergo cervical screening during pregnancy, as it may not provide any benefits and can potentially harm the pregnancy. | A | B | B |
| **22.What procedures are typically involved in cervical cancer screening?** | | | | |
| ChatGPT4.0 turbo (With prompt) | 22. **Procedures Involved in Cervical Cancer Screening**: The primary procedures involved in cervical cancer screening include:  - **Pap Test (Pap Smear)**: This involves collecting cells from the cervix, which are then examined under a microscope to detect abnormalities.  - **HPV Testing**: This test involves checking for the presence of high-risk types of HPV, the virus responsible for most cases of cervical cancer. It can be done using the same cell sample as the Pap test.  - **Colposcopy**: This procedure is typically performed if the results of a Pap test or HPV test are abnormal. During a colposcopy, a special instrument called a colposcope is used to magnify the view of the cervix, allowing for a more detailed examination.  - **Biopsy**: During a colposcopy, if suspicious areas are identified, a biopsy (a small sample of tissue) may be taken from the cervix for further examination to determine if precancerous or cancerous cells are present. | A | A | A |
| ChatGPT4.0 turbo | 22. **Procedures Typically Involved in Cervical Cancer Screening**:  - **Pap Smear**: Collection of cells from the cervix to check for abnormalities.  - **HPV Testing**: Testing for high-risk types of HPV that are known to cause cervical cancer.  - **Colposcopy**: An examination using a special microscope (colposcope) to take a closer look at the cervical tissue, often done if Pap or HPV test results are abnormal.  - **Biopsy**: Taking a small sample of cervical tissue to examine more closely in a lab, typically performed during a colposcopy if necessary.  These procedures are designed to detect cervical cancer at its earliest and most treatable stages, and the choice of screening method may depend on age, health history, and specific medical guidelines. | A | A | A |
| Claude 2 (With prompt) | 22. The main procedure utilized in cervical cancer screening is cervical cytology, commonly known as the Pap test or Pap smear. This involves collecting cells from the transformation zone of the cervix and preparing them on a glass slide to be examined under a microscope for abnormalities. For women over 30, the addition of HPV testing on cervical samples improves the sensitivity of precancer detection. Follow up for abnormal results often requires colposcopy - a magnified visual inspection of the cervix along with targeted biopsies. | A | B | B |
| Claude 2 | 22. Cervical cancer screening involves one or both of these procedures:  - Pap test (Pap smear): Cells are collected from the cervix and examined under a microscope to detect cellular abnormalities indicating possible precancer or cancer.  - HPV test: Samples collected from the cervix are tested for the presence of high-risk types of the human papillomavirus (HPV), which can cause cervical cell changes and cancer. | A | A | A |
| Gemini Pro (With prompt) | 22. What procedures are typically involved in cervical cancer screening? The following procedures are typically involved in cervical cancer screening:  Pelvic exam: A physical examination of the female reproductive organs, including the cervix, uterus, and ovaries. It allows the doctor to check for any abnormalities in size, shape, or texture.  Pap test (cytology): A procedure where cells are collected from the cervix and examined under a microscope to detect precancerous or cancerous changes.  HPV testing: A test that detects the presence of human papillomavirus (HPV), the primary cause of cervical cancer. The specific procedures used may vary depending on a woman's age, risk factors, and screening history. Additional procedures that may be used in certain situations include:  Colposcopy: A procedure that uses a magnifying instrument to examine the cervix in more detail.  Biopsy: A procedure where a small sample of tissue is removed from the cervix for further examination under a microscope. | A | A | A |
| Gemini Pro | 22. The procedures typically involved in cervical cancer screening include:  - Pelvic exam: A healthcare professional examines the external genitalia, vagina, and cervix for signs of infection, inflammation, or other abnormalities.  - Pap test: A healthcare professional takes a sample of cells from the cervix using a spatula or a cotton swab to test for precancerous or cancerous changes.  - HPV (Human Papillomavirus) test: A healthcare professional takes a sample of cells from the cervix, vagina, or both to test for the presence of HPV. The type of HPV test used depends on the woman's age and other factors.  - Colposcopy: If the Pap test or HPV test shows abnormal results, a colposcopy may be performed. This procedure involves using a special microscope to examine the cervix in more detail.  - Biopsy: If the colposcopy shows abnormal areas, a biopsy may be taken to confirm or rule out cancer. | A | A | A |
| Mistral-7B-v0.2 (With prompt) | 22. The following procedures are typically involved in cervical cancer screening: a) Pelvic exam: A pelvic exam is an optional part of cervical cancer screening; it may be performed to assess the overall condition of the female reproductive organs and detect issues unrelated to cervical cancer. b) Pap test (Pap smear): A Pap test involves the collection of cells from the cervix using a spatula and a cytobrush. The cells are then analyzed for the presence of precancerous or cancerous changes. c) Human Papillomavirus (HPV) test: An HPV test searches for high-risk HPV types known to cause cervical cancer. The test may be performed on the same sample used for the Pap test or on a separate sample collected with a self-collection kit. d) Colposcopy: If the results of a Pap test are abnormal, a colposcopy-an examination of the cervix and vaginal area using a microscope-may be performed to further investigate suspected abnormalities. e) Biopsy: If precancerous or cancerous cells are found during a colposcopy, a biopsy may be performed to confirm the diagnosis and determine the stage and type of cervical cancer. | A | A | A |
| Mistral-7B-v0.2 | 22. The procedures typically involved in cervical cancer screening include: - Pap test: A Pap test is a sample of cells collected from the cervix using a spatula or brush. The cells are then analyzed for abnormalities that could indicate precancerous or cancerous changes. - HPV test: An HPV test is used to detect high-risk HPV strains that are linked to cervical cancer. The HPV test can be performed using a sample of cells collected during a Pap test or using a self-collected sample. - Physical examination: During a pelvic exam, the healthcare provider may visually inspect the genital area and cervix, assessing for any abnormalities. This exam may be performed during a Pap test, but it can also be done separately. - Colposcopy: A colposcopy is a procedure in which the healthcare provider uses a specialized microscope to examine the cervix, vagina, and vulva in greater detail, looking for any abnormalities that may not be apparent during a routine pelvic exam. Colposcopy may be recommended if a Pap test or another screening test suggests abnormalities. The colposcopy may involve | A | A | A |
| Starling-LM-7B alpha (With prompt) | 22. What procedures are typically involved in cervical cancer screening?  Cervical cancer screening usually involves two main procedures: the Pap test (also known as the Pap smear) and the human papillomavirus (HPV) test. The Pap test is a procedure in which a small sample of cells is collected from the cervix using a small brush or spatula. This sample is then examined under a microscope to detect any abnormal cells that may be indicative of cervical cancer or precancerous changes. The HPV test, on the other hand, detects the presence of high-risk HPV types that are strongly associated with cervical cancer.  In some cases, a healthcare provider may also perform a visual inspection of the cervix (also known as a visual inspection with acetic acid or VIA) or a visual inspection with Lugol's iodine (VILI). These tests involve applying a solution to the cervix, which can help identify abnormal cells or precancerous changes.  It is important to note that the specific screening procedures and their frequency may vary depending on a woman's age, risk factors, and previous screening results. A healthcare professional should be consulted to determine the appropriate screening plan for an individual patient. | A | A | A |
| Starling-LM-7B alpha | 22. Cervical cancer screening typically involves a combination of cytology (Pap smear) and HPV testing. Colposcopy, LEEP, conization, hysterectomy, radiation therapy, chemotherapy, immunotherapy, and targeted therapy are procedures that may be involved in the diagnosis and treatment of cervical cancer, depending on the stage and specific circumstances of the patient. | A | A | A |
| HuatuoGPT (With prompt) | 22. Cervical cancer screening usually involves pap smears, HPV testing, or a combination of both. These tests help to detect pre-cancerous lesions of the cervix, which can be treated to avoid the development of cervical cancer. | A | A | A |
| HuatuoGPT | 22. Cervical cancer screening usually consists of two main procedures: pap smears and HPV DNA testing. Cervical smear involves collecting a small number of cells from the cervix and examining them under a microscope for the presence of abnormal cells.HPV DNA testing involves detecting the DNA of certain types of HPV that cause cervical cancer.These tests can be done individually or in combination to improve the detection of cervical cancer. | A | A | A |
| BioMedLM 2.7B (With prompt) | 22. What procedures are typically involved in cervical cancer screening? The following procedures are typically involved in cervical cancer screening: a. Pap smear b. Colposcopy c. Colposcopic biopsy d. Colposcopic excisional biopsy e. Cervical conization | C | B | B |
| BioMedLM 2.7B | 22. What procedures are typically involved in cervical cancer screening? The following procedures are typically involved in cervical cancer screening: a. Pap smear b. Colposcopy c. Biopsy d. Treatment e. Follow-up | B | A | A |
| Microsoft Phi2 (With prompt) | 22. Cervical cancer screening typically involves a combination of Pap smears and HPV tests, as well as biopsies to determine the presence of abnormal cells. | A | A | A |
| Microsoft Phi2 | 22. Cervical cancer screening typically involves a Pap smear, which involves collecting cells from the cervix for examination under a microscope. | C | C | C |
| QiZhenGPT (With prompt) | 22. The most common procedures involved in cervical cancer screening include: a. Cervical screening tests: These tests are used to detect the presence of human cervical cancer cells in the uterus. They include the Papanville test, the Cervical Cancer screening test (CCT), and the Human Immunodeficiency Virus (HIV) test. b. Biopsy: A biopsy is a procedure in which a small sample of tissue is taken for examination under a microscope. This is typically used to diagnose cervical cancer and to determine the stage of the disease. c. Immunization: Immunizations are a series of shots that are given to women to prevent the transmission of cervical cancer. d. Follow-up screening: Women who have undergone cervical screening tests and are found to have abnormal results may need to undergo additional screening tests over time to detect any changes in the status of the disease. | C | B | C |
| QiZhenGPT | 22. The recommended procedures for cervical cancer screening during pregnancy include: * Cervical cancer screening tests: These are typically performed on a routine basis, usually at around 12-18 weeks of pregnancy. They include a simple blood test to check for the presence of human chorionic gonadotropin (hCG) and a colposcopy, which is a visual examination of the inside of the uterus using a light and a camera. * colposcopy: This is the most common screening test used during pregnancy. It allows for the visualization of the inside of the uterus and can help identify any abnormalities or changes that may indicate the presence of cervical cancer. * Human chorionic gonadotropin (hCG) test: This test is used to check for the presence of hCG, which is a hormone produced by the placenta during pregnancy. If hCG is detected, it can indicate that the pregnancy is ongoing and the woman should have a colposcopy as described above.  It is important to note that not all cervical cancer cases are detected during pregnancy, and early detection can help prevent the spread of the disease. Therefore, it is important for women to continue to follow up with cervical screening tests, even after the delivery of their baby. | D | D | D |
| **23. What is the examination process for HPV-positive high-risk types?** | | | | |
| ChatGPT4.0 turbo (With prompt) | 23. Examination Process for HPV-Positive High-Risk Types: The examination process begins with identifying the presence of high-risk HPV types through HPV testing. If the test is positive, the next step typically involves reflex cytology (Pap test) to assess for any cellular abnormalities. Depending on the age and specific circumstances of the patient, if the cytology is normal but high-risk HPV is detected, the guidelines recommend a repeat co-testing (HPV test plus cytology) in one year. If the cytology shows abnormalities, further diagnostic procedures such as colposcopy may be indicated. | A | A | A |
| ChatGPT4.0 turbo | Examination Process for HPV-Positive High-Risk Types:  - Women who test positive for high-risk HPV types should be evaluated further since these types are associated with an increased risk of cervical cancer.  - The follow-up typically involves cytological testing (Pap smear) to detect any cellular abnormalities in the cervix. | B | B | B |
| Claude 2 (With prompt) | 23. If initial screening detects high-risk HPV types, current guidelines recommend referral for colposcopy to further examine the cervix. This allows a detailed visual inspection of the cervical tissue to detect any abnormal lesions that may require biopsy or treatment. | A | A | A |
| Claude 2 | The examination process starts with HPV testing. If high-risk types are detected, further examination is warranted. | A | A | A |
| Gemini Pro (With prompt) | 23. What is the examination process for HPV-positive high-risk types?  The examination process for HPV-positive high-risk types typically involves a combination of tests, including:   Colposcopy: A procedure that uses a lighted magnifying instrument to examine the cervix and vagina for any abnormal areas.  Biopsy: A procedure that involves removing a small sample of tissue from the cervix for examination under a microscope.  HPV genotyping: A test that can identify the specific type of HPV that is present. | B | A | A |
| Gemini Pro | The examination process for HPV-positive high-risk types involves a combination of cytological examination and HPV genotyping. | A | A | A |
| Mistral-7B-v0.2 (With prompt) | 23.The examination process for HPV-positive high-risk types typically involves a colposcopy, which is an office procedure that allows the gynecologist to examine the cervix and vaginal walls with a magnifying instrument to identify any abnormalities. A biopsy may also be taken during the colposcopy to obtain a tissue sample for further testing. | A | B | B |
| Mistral-7B-v0.2 | When initial screening results are positive for high-risk Human Papillomavirus (HPV) types, the next step typically involves a colposcopy, which is a more detailed examination of the cervix using a colposcope, a specialized microscope. This allows the healthcare professional to visually assess the cervix and surrounding tissues for any abnormalities. This procedure may also include a cervical biopsy to obtain tissue samples for further testing. | A | B | B |
| Starling-LM-7B alpha (With prompt) | 23. The examination process for HPV-positive high-risk types typically involves a colposcopy, which is an examination of the cervix using a specialized microscope called a colposcope. This allows the gynecologist to visualize the cervix and identify any abnormal areas, which may require a biopsy for further evaluation. | A | A | A |
| Starling-LM-7B alpha | The examination process for HPV-positive high-risk types typically involves an initial screening for HPV, followed by cytological examination (Pap smear). If the cytological examination is abnormal (≥ASC-US), a colposcopy is usually performed. If the high-risk HPV types are confirmed, further testing may be conducted depending on the individual's risk factors, age, and clinical history. | A | A | A |
| HuatuoGPT (With prompt) | 23. The examination process for HPV-positive high-risk types involves a colposcopy and biopsy. Colposcopy is a procedure in which a specialized lens is used to examine the inside of the vagina, cervix, and upper end of the uterus. During colposcopy, the clinician can detect any abnormalities or changes in the tissue of the affected areas. If abnormalities are found, a biopsy may be performed where a small piece of tissue is removed for further investigation under a microscope. | A | A | A |
| HuatuoGPT | The screening process for HPV positive high-risk types begins with an initial screening to determine if you are infected with HPV by testing for the presence of HPV DNA or RNA. if the initial screening shows positive high-risk HPV, further testing is needed to determine if cervical lesions are present. | A | A | A |
| BioMedLM 2.7B (With prompt) | 23. What is the examination process for HPV-positive high-risk types? The examination process for HPV-positive high-risk types is described. The examination process is divided into three parts: (1) the examination of the cervix, (2) the examination of the vagina, and (3) the examination of the vulva. The examination of the cervix is performed with the naked eye, and the examination of the vagina and vulva is performed with a colposcope. The examination of the cervix is performed with the naked eye, and the examination of the vagina and vulva is performed with a colposcope. | C | B | C |
| BioMedLM 2.7B | The examination process for human papillomavirus (HPV)-positive high-risk types includes a Pap smear, colposcopy with biopsy and endocervical curettage if indicated, and treatment of precancerous lesions or cancer when appropriate. | A | A | A |
| Microsoft Phi2 (With prompt) | 23.The examination process for HPV-positive high-risk types is a colposcopy. | A | B | B |
| Microsoft Phi2 | After detecting positive HPV results, the next step would typically involve performing additional tests or screenings to determine the exact nature of the infection. These additional tests could include Pap smears or HPV DNA tests to identify the specific strain(s) causing the infection. | B | B | B |
| QiZhenGPT (With prompt) | 23. The examination process for HPV-positive high-risk types involves a combination of a Pap test and a colposcopy. | B | B | B |
| QizhenGPT | For screening for HPV-positive, high-risk types, the examination process usually involves cytology as the first step and, if the result is negative, HPV typing; if the HPV typing result is positive, then further tests such as colposcopy and cytology are required. | A | B | A |
| **24.When the initial screening shows positive HPV with high-risk HPV types, emphasizing that HPV is not typed, what is the next step in the examination?** | | | | |
| ChatGPT4.0 turbo (With prompt) | 24. Next Steps after Initial Positive High-Risk HPV Screening (Unspecified Type): When an initial screening indicates positive for high-risk HPV types without specifying the type, the next step typically involves performing a reflex cytology (Pap test) to check for any cellular abnormalities. The management following this depends on the cytology results. If the cytology is normal, the patient is usually advised to undergo repeat co-testing in one year. If abnormal cells are detected, a colposcopy may be indicated to further investigate and potentially treat the cervical changes. | A | A | A |
| ChatGPT4.0 turbo | Positive High-Risk HPV, Not Typed:  - If HPV is detected but not typed, a cytological test (Pap smear) is usually performed.  - If the Pap test is normal, follow-up testing might be recommended in 12 months.  - If the Pap test shows abnormalities, further diagnostic procedures, such as colposcopy, may be indicated. | A | A | A |
| Claude 2 (With prompt) | 24. If HPV testing is positive but specific genotypes are unknown, repeat co-testing with HPV and cervical cytology in 1 year. Persistently positive high-risk HPV warrants further evaluation. | C | D | C |
| Claude 2 | When initial screening shows unspecified positive high-risk HPV, the next step is cytological testing (Pap smear) to check for cervical cell changes. | A | A | A |
| Gemini Pro (With prompt) | 24. When the initial screening shows positive HPV with high-risk HPV types emphasizing that HPV is not typed, what is the next step in the examination?  If the initial screening shows positive HPV with high-risk HPV types and HPV is not typed, the next step is typically to perform a colposcopy to further evaluate the cervix for any abnormal areas. | D | C | D |
| Gemini Pro | If the initial screening shows positive HPV with high-risk HPV types, but HPV is not typed, the next step in the examination is to perform a cytological examination (Pap smear). | A | A | A |
| Mistral-7B-v0.2 (With prompt) | 24.When the initial screening shows positive HPV with high-risk HPV types, the next step is to perform a triage test to determine the likelihood of developing cervical precancer or cancer. This may involve a repeat HPV test, a cytological examination (Pap test), or a colposcopy with biopsy, depending on the results of the triage test. | C | B | C |
| Mistral-7B-v0.2 | If the initial screening shows positive HPV with high-risk types but the cytological examination (Pap test or liquid-based cytology) result is negative, this indicates that there are no visible abnormal cell changes at the time of the test. However, since HPV infections can take time to progress to cervical cancer, regular follow-up examinations may be recommended, usually in 12 months and then at consistent intervals based on the healthcare professional's discretion. | D | D | D |
| Starling-LM-7B alpha (With prompt) | 24. If the initial screening shows positive HPV with high-risk HPV types, and HPV typing is not available, the next step in the examination process is to perform a colposcopy. This is to assess the cervix for any visible abnormalities, such as lesions or changes in the cervical tissue. | D | D | D |
| Starling-LM-7B alpha | If the initial screening shows positive HPV with high-risk HPV types and the cytological examination result is negative, it is essential to consult with a healthcare professional. Regular check-ups may be recommended, with the frequency depending on the individual's risk factors and clinical history. | C | D | D |
| HuatuoGPT (With prompt) | 1. When initial screening shows positive HPV, especially high-risk types, but does not indicate a specific type, the next step is repeat HPV DNA testing at 6-12 months. If both tests are positive, a colposcopy and biopsy are performed. If either test is positive, repeat HPV DNA testing is performed. Colposcopy and biopsy if both tests are positive. If both tests are negative, no further testing or investigation is required. | C | D | D |
| HuatuoGPT | If the initial screen shows positive for high-risk HPV, but no specific type can be categorized, it is important to undergo periodic retesting. The frequency of review may vary depending on the doctor's recommendation and the specific situation, usually every 6 months to 1 year. | C | B | C |
| BioMedLM 2.7B (With prompt) | 24. When the initial screening shows positive HPV with high-risk HPV types emphasizing that HPV is not typed, what is the next step in the examination? | D | D | D |
| BioMedLM 2.7B | The patient should be referred to colposcopy and biopsy for further evaluation of the lesion. | C | D | C |
| Microsoft Phi2 (With prompt) | 24. When the initial screening shows positive HPV with high-risk HPV types emphasizing that HPV is not typed, the next step in the examination is to perform a colposcopy. | D | D | D |
| Microsoft Phi2 | Once the specific type of HPV is identified through these tests, the health·care provider will assess whether there are any signs of abnormal changes (such as cervical intraepithelial neoplasia grade 2 or higher) on the cervix. This assessment helps determine the need for further intervention or treatment. | C | D | D |
| QiZhenGPT (With prompt) | 24. If the initial screening shows positive HPV with high-risk HPV types and HPV is not typed, the next step would be to perform a colposcopy to visualize the Cervical Cavity. | D | C | D |
| QiZhenGPT | If the initial screening is positive for HPV and a high-risk type is detected, cytology should be performed. In the case of a negative cytology result, re-screening is usually necessary to determine the need for histologic examination | D | D | D |
| **25. When the initial screening shows positive HPV with high-risk HPV types, and the cytological examination result is negative, what does it indicate? Should regular check-ups follow? and if so, what should be the frequency?** | | | | |
| ChatGPT4.0 turbo (With prompt) | 25. Interpretation and Follow-Up of Negative Cytology with Positive High-Risk HPV: A positive test for high-risk HPV types combined with a negative cytological result suggests that while the high-risk virus is present, it has not yet caused significant changes to the cervical cells. Regular monitoring is crucial in this scenario. The recommended follow-up is to repeat both HPV and cytology testing in one year to monitor for any developments, as persistent infection with high-risk HPV can lead to cervical dysplasia or cancer over time. | A | A | A |
| ChatGPT4.0 turbo | Positive High-Risk HPV, Negative Cytology:  - This result indicates that the high-risk HPV is present, but no cellular abnormalities are detected in the cervix.  - Regular follow-up is recommended to monitor for any changes. The frequency of check-ups depends on specific guidelines but may be every 3-5 years for co-testing (HPV and cytology) or more frequently if only HPV testing is used. | A | A | A |
| Claude 2 (With prompt) | 25. A positive high-risk HPV result with normal cytology indicates previous or current HPV infection. Such patients need repeat co-testing every year as per guidelines. Persistent infection for 2 years or more warrants colposcopy, despite normal cytology. | A | B | A |
| Claude 2 | A negative cytology with positive high-risk HPV indicates an HPV infection without significant cervical changes so far. Follow-up co-testing (HPV and cytology) every 12 months is recommended. | A | B | A |
| Gemini Pro (With prompt) | 25. When the initial screening shows positive HPV with high-risk HPV types and the cytological examination result is negative, what does it indicate? Should regular check-ups follow and if so, what should be the frequency?  If the initial screening shows positive HPV with high-risk HPV types and the cytological examination result is negative, it may indicate that the HPV infection is transient and will clear on its own. However, regular check-ups are still recommended to monitor for any changes in the cervix. The frequency of check-ups will depend on the individual patient's risk factors and medical history. | B | B | B |
| Gemini Pro | If the initial screening shows positive HPV with high-risk HPV types and the cytological examination result is negative, it indicates a possible transient HPV infection. Regular check-ups are recommended, with the frequency determined by the healthcare provider based on individual risk factors and the specific HPV type detected. | C | B | B |
| Mistral-7B-v0.2 (With prompt) | 25.If the initial screening shows positive HPV with high-risk HPV types and the cytological examination result is negative, it indicates that there are no abnormal cells detected in the cervix at that time. However, regular follow-up appointments are recommended to monitor for any future development of precancerous or cancerous changes. The frequency of follow-up appointments may depend on the individual's age, HPV type, and other risk factors. | B | A | B |
| Mistral-7B-v0.2 | When the initial screening shows positive HPV with high-risk HPV types, and the cytological examination result shows low-grade squamous intraepithelial lesions (LSIL) or atypical glandular cells, a colposcopy is usually recommended to further investigate these abnormalities. | D | D | D |
| Starling-LM-7B alpha (With prompt) | 25. When the initial screening shows positive HPV with high-risk HPV types and the cytological examination result is negative, it indicates that there are no visible abnormalities in the cervical cells. However, due to the potential for false-negative results, regular follow-up examinations are recommended. The frequency of these follow-up examinations should be determined by the gynecologist, taking into account the patient's age, HPV type, and other risk factors. | C | C | C |
| Starling-LM-7B alpha | If the initial screening shows positive HPV with high-risk HPV types and the cytological examination result is negative, this generally means that the abnormal cells detected by the HPV test have not progressed to cervical dysplasia. However, regular follow-up examinations are necessary to monitor for any changes. The recommended frequency for follow-ups varies but generally ranges from 6 to 12 months, depending on the specific results and recommendations from the healthcare provider. | A | A | A |
| HuatuoGPT (With prompt) | Colposcopy is recommended when initial screening shows positive HPV, especially high-risk types, but cytology is negative. Colposcopy is usually repeated after 1-2 years. If no abnormalities are found on either colposcopy, follow-up screening is performed every 3 years. If any abnormality is found, a biopsy is required. It is important to note that cervical cancer rarely develops with negative cytology results and positive HPV DNA, but in some cases, colposcopy and biopsy may be necessary. | B | B | B |
| HuatuoGPT | If the initial screening result is HPV-positive and in the high-risk category and the cytology is negative, this may indicate a mild infection or absence of lesions. However, regular follow-up is recommended to ensure that the risk of lesions is low. Cervical cytology is usually recommended every 6-12 months. | A | A | A |
| BioMedLM 2.7B (With prompt) | 25. When the initial screening shows positive HPV with high-risk HPV types and the cytological examination result is negative, what does it indicate? Should regular check-ups follow and if so, what should be the frequency? The initial screening result is positive for high-risk HPV types and the cytological examination result is negative. The patient should be followed up with regular check-ups. The frequency of follow-up should be determined by the patient\'s age, the results of the initial screening, and the results of the cytological examination. | B | B | B |
| BioMedLM 2.7B | The patient should undergo regular check-up every 3 months for 2 years after treatment of cervical cancer. If there are no abnormal findings on the Pap smear test or colposcopy at any time during this period, then the patient can return to routine health care. | C | C | C |
| Microsoft Phi2 (With prompt) | 25. When the initial screening shows positive HPV with high-risk HPV types and the cytological examination result is negative, it indicates that the patient is not at risk of developing cervical cancer. Regular check-ups are not necessary. | D | C | D |
| Microsoft Phi2 | If the cytological examination result is negative, indicating no abnormalities, then regular check-ups can usually continue at the recommended intervals based on age, risk factors, and previous test results. However, it's important to note that even with a negative result, ongoing surveillance for potential changes remains crucial due to the long latency period associated with HPV infections. | B | A | A |
| QiZhenGPT (With prompt) | 25. If the initial screening shows positive HPV with high-risk HPV types and the cytological examination result is negative, it indicates that the sample is not suitable for analysis and regular check-ups should follow. The frequency of check-ups depends on the individual patient's risk factors and the stage of the disease. | C | D | C |
| QiZhenGPT | If the initial screening shows positive HPV with high-risk HPV types and the cytological examination result is negative, it may indicate a low-risk HPV infection or a false positive result. Regular check-ups, such as a repeat cytological examination and a HPV test, should be performed to monitor the situation. The frequency of check-ups will depend on the individual case and the healthcare provider's recommendation. | A | A | A |
| **26. When the initial screening shows positive HPV with high-risk HPV types, and the cytological examination result is ≥ASC-US, is the next step to perform a colposcopy?** | | | | |
| ChatGPT4.0 turbo (With prompt) | 26. Management of Positive High-Risk HPV and Cytology ≥ASC-US: Yes, if the cytological examination result is atypical squamous cells of undetermined significance (ASC-US) or more severe (≥ASC-US) in the context of a positive high-risk HPV test, the recommended next step is to perform a colposcopy. This allows for a detailed examination of the cervix with possible biopsies of abnormal areas to accurately diagnose and manage potential precancerous or cancerous changes. | A | A | A |
| ChatGPT4.0 turbo | Positive High-Risk HPV, Cytology ≥ASC-US:  - ASC-US stands for "Atypical Squamous Cells of Undetermined Significance."  - If the cytology result is ASC-US or higher, a colposcopy is typically recommended to examine the cervix more closely and possibly take biopsies of any suspicious areas. | A | A | A |
| Claude 2 (With prompt) | 26. Yes, cytology showing atypical squamous cells (ASC-US or more severe) with high-risk HPV positivity indicates abnormal cervical cells. Next step is referral for colposcopy - visual inspection and directed biopsy if any acetowhite lesions are detected. | A | A | A |
| Claude 2 | Yes, if cytology shows ASC-US or more severe changes with high-risk HPV, the next step is colposcopy to visually inspect the cervix. | A | A | A |
| Gemini Pro (With prompt) | 26. When the initial screening shows positive HPV with high-risk HPV types and the cytological examination result is ≥ASC-US, is the next step to perform a colposcopy?  Yes, if the initial screening shows positive HPV with high-risk HPV types and the cytological examination result is ≥ASC-US (atypical squamous cells of undetermined significance), the next step is typically to perform a colposcopy to further evaluate the cervix for any abnormal areas. | A | A | A |
| Gemini Pro | If the initial screening shows positive HPV with high-risk HPV types and the cytological examination result is ≥ASC-US (atypical squamous cells of undetermined significance or worse), the next step is to perform a colposcopy, which is a magnified visual examination of the cervix and vagina to detect any abnormalities. | A | A | A |
| Mistral-7B-v0.2 (With prompt) | 26.When the initial screening shows positive HPV with high-risk HPV types and the cytological examination result is ≥ASC-US (atypical squamous cells of undetermined significance), the next step is to perform a colposcopy. This procedure allows the gynecologist to examine the cervix and vaginal walls more closely and to take a biopsy if any abnormalities are identified. | A | A | A |
| Mistral-7B-v0.2 | When the initial screening shows positive HPV with high-risk types, and the cytological examination result is ASC-US (atypical squamous cells of undetermined significance), this indicates that there are some abnormal cells present, but additional testing is needed to determine whether these cells are cancerous or precancerous. The next step is usually a colposcopy and a biopsy to investigate further. | B | C | B |
| Starling-LM-7B alpha (With prompt) | 26. When the initial screening shows positive HPV with high-risk HPV types and the cytological examination result is ≥ASC-US (Atypical Squamous Cells of Undetermined Significance), the next step in the examination process is to perform a colposcopy. This is to further assess the cervix for any visible abnormalities, which may require a biopsy for further evaluation. | B | B | B |
| Starling-LM-7B alpha | If the initial screening shows positive HPV with high-risk HPV types and the cytological examination result is ≥ASC-US, the next step is usually to perform a colposcopy. Again, it is essential to consult with a healthcare professional for personalized recommendations. | A | B | A |
| HuatuoGPT (With prompt) | 26. What does it mean to be positive for high-risk HPV and have a smear result of ASC-H? When the initial screening result shows positive high-risk HPV and the smear result is ASC-H (high grade cervical intraepithelial neoplasia cannot be ruled out), this indicates the presence of suspected high grade cervical cellular abnormalities and the need for further colposcopy. Colposcopy is an endoscopic procedure used for direct visualization of the cervix and more detailed evaluation. | B | B | B |
| HuatuoGPT | If the initial screening shows positive high-risk HPV and the cytology is ≥ASC-US, the next step is usually to perform a colposcopy. Colposcopy can provide more detailed information about the extent of cervical lesions and cancer risk. | B | A | A |
| BioMedLM 2.7B (With prompt) | 26. When the initial screening shows positive HPV with high-risk HPV types and the cytological examination result is ≥ASC-US, is the next step to perform a colposcopy? The next step is to perform a colposcopy. | A | A | A |
| BioMedLM 2.7B | Yes. The patient should undergo regular checks for cervical cancer at 3 months after treatment completion | A | A | A |
| Microsoft Phi2 (With prompt) | 26. When the initial screening shows positive HPV with high-risk HPV types and the cytological examination result is ≥ASC-US, the next step is to perform a colposcopy. | A | A | A |
| Microsoft Phi2 | Yes, when the initial screening shows positive HPV with high-risk HPV types and the cytological examination result is ≥ASC-US, a colposcopy is often performed. A colposcopy is a procedure where a healthcare professional uses a magnifying instrument called a colposcope to examine the cervix more closely. It allows them to identify any areas of concern, such as precancerous lesions, and take appropriate measures accordingly. | A | A | A |
| QiZhenGPT (With prompt) | 26. If the cytological examination result is ≥ASC-US, it may indicate a high risk of developing cervical cancer and a colposcopy should be performed to visualize the Cervical Cavity. | A | A | A |
| QiZhenGPT | If the initial screening result is HPV-positive and high-risk, and the cytology result is ASC-US (of uncertain significance), further testing by colposcopy is usually needed. colposcopy is a visual test that can help your doctor more accurately assess the risk of lesions and take the appropriate therapeutic measures. | A | A | A |
| **27.When the initial screening shows positive HPV with high-risk HPV types, and upon typing it turns out to be HPV16/18 positive, is the next step to perform a colposcopy?** | | | | |
| ChatGPT4.0 turbo (With prompt) | 27. Management of Positive HPV16/18 with High-Risk Types: Yes, if the HPV typing specifically identifies HPV types 16 or 18, which are known to have a higher risk of progressing to cervical cancer, a colposcopy is recommended as the next step. This recommendation holds regardless of the cytology results, due to the aggressive nature of these HPV types. Early and accurate detection of changes through colposcopy can significantly improve the management and prognosis of potential cervical precancerous lesions. | A | A | A |
| ChatGPT4.0 turbo | Positive HPV16/18:  - HPV types 16 and 18 are associated with a higher risk of cervical cancer.  - If typing reveals a positive result for HPV16 or HPV18, a colposcopy is usually recommended to assess for any precancerous changes. | A | A | A |
| Claude 2 (With prompt) | 27. Yes, HPV 16/18 genotypes are highest risk for progression to cervical cancer. Despite normal cytology, positive HPV 16/18 status alone is indication for referral for colposcopy as per current ASCCP management guidelines. | A | A | A |
| Claude 2 | Yes, if HPV genotyping confirms HPV16/18, referral for immediate colposcopy is recommended given the higher cancer risk. | A | A | A |
| Gemini Pro (With prompt) | 27. When the initial screening shows positive HPV with high-risk HPV types and upon typing it turns out to be HPV16/18 positive, is the next step to perform a colposcopy?  Yes, if the initial screening shows positive HPV with high-risk HPV types and upon typing it turns out to be HPV16/18 positive, the next step is typically to perform a colposcopy to further evaluate the cervix for any abnormal areas. | A | A | A |
| Gemini Pro | If the initial screening shows positive HPV with high-risk HPV types and upon typing it turns out to be HPV16/18 positive, the next step is to perform a colposcopy, as HPV16 and HPV18 are associated with a higher risk of cervical cancer. | A | A | A |
| Mistral-7B-v0.2 (With prompt) | 27.When the initial screening shows positive HPV with high-risk HPV types and upon typing it turns out to be HPV16/18 positive, the next step is to perform a colposcopy. HPV16 and HPV18 are the two most common high-risk HPV types associated with cervical cancer, and the presence of these types increases the likelihood of precancerous or cancerous changes. A colposcopy with biopsy can help to identify and treat any abnormalities before they progress to cancer. | A | B | A |
| Mistral-7B-v0.2 | When the initial screening shows positive HPV with high-risk HPV types, specifically HPV16 or HPV18, these types are associated with a higher risk of developing cervical cancer, and a colposcopy is typically the next step to assess for cervical lesions or precancerous changes. | A | A | A |
| Starling-LM-7B alpha (With prompt) | 27. When the initial screening shows positive HPV with high-risk HPV types and upon typing, it turns out to be HPV16/18 positive, the next step in the examination process is to perform a colposcopy. This is because HPV16 and HPV18 are the most high-risk types associated with the development of cervical cancer, and a colposcopy can help identify any visible abnormalities in the cervical tissue.  gynecologist, taking into account the patient's age, HPV type, and other risk factors. | A | B | A |
| Starling-LM-7B alpha | If the initial screening shows positive HPV with high-risk HPV types and it is confirmed to be HPV16/18 positive, the next step is often to perform a colposcopy. However, this may vary depending on the individual's risk factors and clinical history. | A | A | A |
| HuatuoGPT (With prompt) | 27. What does it mean when positive for high-risk HPV and HPV 16/18? When the initial screen is positive for high-risk HPV and positive for HPV types 16 or 18 (known as “high-risk” HPV), it means that you are infected with two of the most common types of high-risk HPV known to exist. | D | C | D |
| HuatuoGPT | colposcopy should be performed to determine the extent and degree of the lesion if the initial screening result is HPV-positive and of a high-risk type, and the cytology result is HPV16/18 positive. In some cases, a cervical biopsy may be required to assess the extent of the lesion. | A | A | A |
| BioMedLM 2.7B (With prompt) | 27. When the initial screening shows positive HPV with high-risk HPV types and upon typing it turns out to be HPV16/18 positive, is the next step to perform a colposcopy? Yes. The next step is to perform a colposcopy. | A |  | A |
| BioMedLM 2.7B | Yes. A colposcopic examination should be performed in all cases of positive HPV test results for high risk (HR) HPV types 16 or 18. | B | A | A |
| Microsoft Phi2 (With prompt) | 27. When the initial screening shows positive HPV with high-risk HPV types and upon typing it turns out to be HPV16/18 positive, the next step is to perform a colposcopy. | A | A | A |
| Microsoft Phi2 | In the case of HPV16/18 positivity detected during the initial screening, a colposcopy is still necessary despite the absence of other high-risk HPV strains. Colposcopies are primarily used to detect and manage abnormal changes caused by high-risk HPV types like HPV16 and 18. Other low-risk HPV types do not pose the same level of risk as high-risk ones, and their presence alone might not warrant immediate intervention. | B | B | B |
| QiZhenGPT (With prompt) | 27. If the initial screening shows positive HPV with high-risk HPV types and upon typing it turns out to be HPV16/18 positive, it indicates that these types of HPV are the most common cause of cervical cancer, and a colposcopy is recommended to confirm the diagnosis and identify any potential areas of risk. | A | A | A |
| QiZhenGPT | If the initial screening shows positive HPV with high-risk HPV types and the cytological examination result is HPV16/18 positive, a colposcopy is typically recommended as the next step. This is because HPV16/18 is a high-risk HPV type that can lead to cervical cancer if left untreated. | A | A | A |
| **28.When the initial screening shows positive HPV with high-risk HPV types, and upon typing it shows neither HPV16/18 positive but one of the other twelve types, what is the most likely subsequent examination?** | | | | |
| ChatGPT4.0 turbo (With prompt) | 28. Subsequent Examination for Other High-Risk HPV Types: When the initial screening is positive for high-risk HPV types other than HPV16/18, the most likely subsequent examination depends on the results of the reflex cytology (Pap test). If the cytology is normal, the current guidelines recommend a repeat co-testing (HPV test and cytology) in one year. If the cytology shows abnormalities, such as atypical squamous cells of undetermined significance (ASC-US) or worse, a colposcopy should be performed to closely examine the cervix and potentially take biopsies from any suspicious areas. | A | A | A |
| ChatGPT4.0 turbo | Positive High-Risk HPV, Neither HPV16/18 but Other High-Risk Types:  - If the HPV typing shows another high-risk type besides 16 or 18, the next steps may depend on the cytology results.  - If the cytology is normal, repeat testing in a year might be suggested.  - If the cytology shows abnormalities, a colposcopy would likely be the next step. | B | B | B |
| Claude 2 (With prompt) | 28. For positive high-risk HPV types other than 16/18, with normal cytology, repeat HPV and cytology co-testing in 1 year is recommended. Persistent infection with high-risk types warrants colposcopy, even if cytology remains normal. | D | C | C |
| Claude 2 | For other positive high-risk types besides 16/18, repeat co-testing in 12-months is more common, with referral to colposcopy if cytology becomes ASC-US+ or if high-risk HPV persists over time. | B | B | B |
| Gemini Pro (With prompt) | 28. When the initial screening shows positive HPV with high-risk HPV types and upon typing it shows neither HPV16/18 positive but one of the other twelve types, what is the most likely subsequent examination? Answer: Repeat cytology in 12 months | C | C | C |
| Gemini Pro | If the initial screening shows positive HPV with high-risk HPV types and upon typing it shows neither HPV16/18 positive but one of the other twelve types, the most likely subsequent examination is a repeat HPV test at a specific interval (typically 6-12 months) to monitor the persistence of the infection. | C | D | D |
| Mistral-7B-v0.2 (With prompt) | 28.When the initial screening shows positive HPV with high-risk HPV types but upon typing it shows neither HPV16/18 positive but one of the other twelve types, the most likely subsequent examination would be a repeat HPV test in 12 months. If the repeat HPV test is also positive, or if the individual has other risk factors for cervical cancer, a colposcopy may be recommended. | C | C | C |
| Mistral-7B-v0.2 | When the initial screening shows positive HPV with high-risk HPV types, but the testing fails to identify HPV16 or HPV18, it's important to note that other high-risk HPV types can also lead to cervical cancer. The next step would usually involve a colposcopy and biopsy to evaluate for any abnormal findings or precancerous changes in the cervix. Regular follow-up appointments would be necessary for monitoring the HPV status and any potential development of cervical lesions or cancer. | B | B | B |
| Starling-LM-7B alpha (With prompt) | 28. When the initial screening shows positive HPV with high-risk HPV types and upon typing it shows neither HPV16/18 positive but one of the other twelve types, the most likely subsequent examination is a repeat HPV test in 1-6 months, followed by a cytology test if the HPV test remains positive. | C | C | C |
| Starling-LM-7B alpha | If the initial screening shows positive HPV with high-risk HPV types and it is confirmed to be one of the other twelve types (but not HPV16/18 positive), the most likely subsequent examination is a colposcopy. However, this may vary depending on the individual's risk factors and clinical history. | C | C | C |
| HuatuoGPT (With prompt) | 28. If the initial screen is positive for high-risk HPV but negative for HPV types 16/18 and positive for the other 11 types, what should be the next step? According to the updated guidelines, colposcopy is now recommended for cases where the initial screening showed positive high-risk HPV but the HPV 16/18 results were negative and the other 11 types were positive. This is because infection with other high-risk HPV types may also increase the risk of cervical cancer. | C | D | C |
| HuatuoGPT | If the initial screening result shows HPV positivity and is of a high-risk type, and the cytology result is of 12 types other than HPV16/18 positivity, the most likely follow-up test is cytology and histology (e.g., cervical biopsy). This helps to determine the extent and type of lesion and to develop a treatment plan accordingly. | C | B | C |
| BioMedLM 2.7B (With prompt) | 28. When the initial screening shows positive HPV with high-risk HPV types and upon typing it shows neither HPV16/18 positive but one of the other twelve types, what is the most likely subsequent examination? The most likely subsequent examination is colposcopy. | D | D | D |
| BioMedLM 2.7B | The next step in the evaluation should be colposcopy or biopsy to determine whether there are any abnormalities present. If no abnormalities are found on colposcopic exam, repeat cytology at 6 months may be considered as a follow up test for women who have not had a hysterectomy. If the patient has undergone a hysterectomy, then the next recommended step would be a Pap smear every 3 years until age 65 years; thereafter, annual Pap smears can continue if the patient remains asymptomatic without evidence of cervical dysplasia. | C | C | C |
| Microsoft Phi2 (With prompt) | 28. The initial screening shows positive HPV with high-risk HPV types and upon typing it shows neither HPV16/18 positive but one of the other twelve types, what is the most likely subsequent examination? | D | D | D |
| Microsoft Phi2 | If the initial screening shows positive HPV with high-risk HPV types, and upon typing it reveals none of the HPV16/18 positive strains but one of the other twelve types, the most likely subsequent examination depends on various factors. While the absence of HPV16/18 doesn't eliminate the need for close monitoring, some low-risk HPV types have lower risks than others. Therefore, depending on the individual's specific situation, the healthcare provider may recommend either continued surveillance with regular check-ups or alternative management strategies tailored to the particular HPV type detected. | D | D | D |
| QiZhenGPT (With prompt) | 28. The most likely subsequent examination would be a colposcopy. | D | D | D |
| QiZhenGPT | If the initial screening shows positive HPV with high-risk HPV types and the cytological examination result shows neither HPV16/18 positive but one of the other twelve types, the most likely subsequent examination is an HPV test to determine the specific type of HPV. The healthcare provider may also recommend a colposcopy to further evaluate the cervix and identify any abnormal areas that may need to be treated. | D | C | C |
| **29.When cytological examination indicates ASC-US as an abnormal initial screening result, what is the next step in the examination?** | | | | |
| ChatGPT4.0 turbo (With prompt) | 29. Next Step after ASC-US Result: When the cytological examination indicates atypical squamous cells of undetermined significance (ASC-US), the next step typically involves HPV testing for triage if not already performed concurrently. If HPV testing was not done with the initial screening, it should be performed to determine the presence of high-risk HPV types. The management will then be based on the HPV test results. | C | B | B |
| ChatGPT4.0 turbo | ASC-US on Cytology:**  - For atypical squamous cells of undetermined significance (ASC-US), reflex HPV testing is often used to determine the need for further evaluation.  - If HPV testing is not performed, repeat cytology at 6 and 12 months or immediate colposcopy may be considered, depending on the guidelines followed. | A | A | A |
| Claude 2 (With prompt) | 29. ASC-US cytology indicates atypical squamous cells of undetermined significance. Current guidelines recommend reflex HPV testing if ASC-US is the initial result. Alternatively, repeat cytology in 6-12 months. | C | C | C |
| Claude 2 | When cytology shows ASC-US, the next step is triage testing, usually with an HPV test. | A | A | A |
| Gemini Pro (With prompt) | 29. When cytological examination indicates ASC-US as an abnormal initial screening result, what is the next step in the examination? Answer: HPV testing | C | C | C |
| Gemini Pro | When cytological examination indicates ASC-US as an abnormal initial screening result, the next step in the examination is:  - HPV testing (DNA test) | A | A | A |
| Mistral-7B-v0.2 (With prompt) | 29.When cytological examination indicates ASC-US as an abnormal initial screening result, the next step in the examination is usually a repeat cytological examination in 12 months. If the repeat cytological examination is also abnormal or if HPV testing is used for triage and HPV is positive, a colposcopy may be recommended. | B | B | B |
| Mistral-7B-v0.2 | When cytological examination indicates ASC-US as an abnormal initial screening result, the next step in the examination is usually a repeat Pap test in 12 months. However, some clinicians may choose to perform HPV testing for triage instead, depending on the patient's age and risk factors. | C | C | C |
| Starling-LM-7B alpha (With prompt) | 29. When cytological examination indicates ASC-US as an abnormal initial screening result, the next step in the examination is to use HPV testing for triage. If the HPV test is positive, a colposcopy should be performed. If the HPV test is negative, a repeat cytology test in 6-12 months is recommended. | A | A | A |
| Starling-LM-7B alpha | When cytological examination indicates ASC-US as an abnormal initial screening result, the next step in the examination is to use HPV testing for triage. If the HPV test is positive, a colposcopy will be required. If the HPV test is negative, continue with regular screening in one year. | A | A | A |
| HuatuoGPT (With prompt) | 29. If cytology shows an abnormal initial screening result of ASC-US, what is the next test? If cytology shows an abnormal initial screening result of ASC-US, updated guidelines now recommend HPV triage testing. If the HPV test result is positive, colposcopy is recommended. If the HPV test result is negative, no further testing is needed, but periodic testing is still recommended to monitor the situation. | A | A | A |
| HuatuoGPT | If cytology shows ASC-US as an abnormal initial screening result, the next step is usually to repeat the test. Repeat testing can help confirm results and reduce the likelihood of false-positive results. If repeat testing still shows ASC-US, colposcopy may need to be considered. | A | B | B |
| BioMedLM 2.7B (With prompt) | 29. When cytological examination indicates ASC-US as an abnormal initial screening result, what is the next step in the examination? The next step in the examination is colposcopy. If the colposcopy is normal, the patient is followed up with cytology and HPV testing. If the colposcopy is abnormal, the patient is referred to a gynecologist for biopsy. | C | C | C |
| BioMedLM 2.7B | The next step of examination should be colposcopy and biopsy if necessary. If the lesion is not suspected to be cervical cancer or CIN2+, it can be followed up by cytology every 6 months for 2 years. | C | D | D |
| Microsoft Phi2 (With prompt) | 29. When cytological examination indicates ASC-US as an abnormal initial screening result, what is the next step in the examination? The next step in the examination is a colposcopy. | D | D | D |
| Microsoft Phi2 | If cytological examination indicates ASC-US as an abnormal initial screening result, further testing such as colposcopy or biopsy may be recommended depending on the patient's risk factors and clinical presentation. It is important for patients to discuss their results with their healthcare provider to determine the appropriate course of action. | C | C | C |
| QiZhenGPT (With prompt) | 29. The next step in the examination would be a follow-up ASC-US test. | D | D | D |
| QiZhenGPT | When cytology shows ASC-US as an abnormal initial screening result, what is the next step? A. When cytology indicates ASC-US as an abnormal initial screening result, further testing should be performed to determine the cause of the abnormality. This may include performing cervical cytology (C cytology), human papillomavirus (HPV) testing, cytology and histology (biopsy). | A | A | A |
| **30. When cytological examination indicates ASC-US as an abnormal initial screening result, and HPV is used for triage, if HPV is positive, should a colposcopy follow?** | | | | |
| ChatGPT4.0 turbo (With prompt) | 30. Management of ASC-US with Positive HPV: If the HPV test is positive following an ASC-US cytology result, a colposcopy is recommended. This is because the combination of ASC-US and a positive test for high-risk HPV types increases the likelihood of significant cervical pathology that may require further diagnostic evaluation and management. | A | A | A |
| ChatGPT4.0 turbo | ASC-US with Positive HPV:  - If HPV testing is positive, especially for high-risk types, a colposcopy is generally recommended to examine the cervix more closely. | A | A | A |
| Claude 2 (With prompt) | 30. Yes, if HPV testing is done for ASC-US triage and is positive, colposcopy is recommended irrespective of which high-risk HPV genotype is detected. The positive predictive value for ≥ CIN2 is sufficient to warrant colposcopic examination. | A | A | A |
| Claude 2 | Yes, if HPV triage is positive with ASC-US cytology, guidelines recommend referral for colposcopy. | A | A | A |
| Gemini Pro (With prompt) | 30. When cytological examination indicates ASC-US as an abnormal initial screening result and HPV is used for triage, if HPV is positive, should a colposcopy follow? Answer: Yes | A | A | A |
| Gemini Pro | Yes, a colposcopy should follow. | A | A | A |
| Mistral-7B-v0.2 (With prompt) | 30.When cytological examination indicates ASC-US as an abnormal initial screening result and HPV is used for triage, if HPV is positive, a colposcopy should follow. The presence of high-risk HPV types increases the likelihood of precancerous or cancerous changes, and a colposcopy with biopsy can help to identify and treat any abnormalities before they progress to cancer. | A | A | A |
| Mistral-7B-v0.2 | When cytological examination indicates ASC-US as an abnormal initial screening result, and HPV testing is used for triage, if HPV is positive, then a colposcopy is recommended, as the presence of high-risk HPV increases the likelihood of an underlying cervical lesion that may require further evaluation. | A | A | A |
| Starling-LM-7B alpha (With prompt) | 30. When cytological examination indicates ASC-US as an abnormal initial screening result and HPV is used for triage, if HPV is positive, a colposcopy should follow. | A | A | A |
| Starling-LM-7B alpha | Yes, if cytological examination indicates ASC-US as an abnormal initial screening result and HPV is used for triage, and HPV is positive, a colposcopy should be the next step. | A | A | A |
| HuatuoGPT (With prompt) | 30. If cytology shows an abnormal initial screening result of ASC-US and HPV is used for shunting, what should be next if the HPV test result is positive? If cytology shows an abnormal initial screening result of ASC-US and HPV for shunting and the HPV test result is positive, updated guidelines now recommend colposcopy. | A | A | A |
| HuatuoGPT | Colposcopy is usually recommended if cytology shows ASC-US as an abnormal initial screening result and HPV is used for triage and is positive for HPV. Colposcopy can provide additional information about the extent of cervical lesions and cancer risk. | A | A | A |
| BioMedLM 2.7B (With prompt) | 30. When cytological examination indicates ASC-US as an abnormal initial screening result and HPV is used for triage, if HPV is positive, should a colposcopy follow? The answer is yes. The patient should be referred to a gynecologist for colposcopy. | A | B | A |
| BioMedLM 2.7B | Colposcopic examination may be performed in patients with persistent HPV infection or high grade squamous intraepithelial lesion (HSIL) on cytology at 6 months after treatment of CIN 2/3. However, it has not been proven to reduce cervical cancer incidence. | A | B | A |
| Microsoft Phi2 (With prompt) | 30. When cytological examination indicates ASC-US as an abnormal initial screening result and HPV is used for triage, if HPV is positive, should a colposcopy follow? Yes, a colposcopy should follow. | A | B | A |
| Microsoft Phi2 | If cytological examination shows ASC-US as an abnormal initial screening result and HPV is used for triage, the patient will need a colposcopy to investigate further. | A | A | A |
| QiZhenGPT (With prompt) | 30. If HPV is positive, a colposcopy should be performed. | B | B | B |
| QiZhenGPT | When cytology indicates ASC-US as an abnormal initial screening result and HPV is used for sorting, is colposcopy required if HPV is positive? A. When cytology shows ASC-US as an abnormal initial screening result and the test is positive for HPV, colposcopy is usually needed. colposcopy is a test used to check for abnormalities of the cervical epithelium, which is histologically examined by using special mirrors and techniques that allow for a clearer view of changes in the cervical epithelium. | A | A | A |
| **31.When cytological examination indicates ASC-US as an abnormal initial screening result, and HPV is used for triage, if HPV is negative, what should be the subsequent examination?** | | | | |
| ChatGPT4.0 turbo (With prompt) | 31. Subsequent Examination after ASC-US with Negative HPV: If HPV testing is used for triage after an ASC-US result and the result is negative for high-risk HPV, the risk of significant cervical disease is low. The current guidelines recommend repeating cytology testing in three years. This approach balances the need for monitoring while avoiding unnecessary procedures. | C | B | B |
| ChatGPT4.0 turbo | ASC-US with Negative HPV:  - If HPV testing is negative, the risk of cervical cancer is low, and the patient may return to routine screening intervals.  - Some guidelines may recommend a repeat Pap test in 12 months to ensure no abnormalities develop. | A | A | A |
| Claude 2 (With prompt) | 31. If HPV testing is negative when performed for ASC-US triage, return to routine age-based screening per current guidelines. In most cases this would mean repeat cytology testing in 3 years. | C | C | C |
| Claude 2 | If HPV triage is negative with ASC-US cytology, repeat cytology testing in 12 months is appropriate as the next step. | A | A | A |
| Gemini Pro (With prompt) | 31. When cytological examination indicates ASC-US as an abnormal initial screening result and HPV is used for triage, if HPV is negative, what should be the subsequent examination? Answer: Repeat cytology in 12 months | A | B | B |
| Gemini Pro | Repeat cytological examination in 6-12 months. | B | C | B |
| Mistral-7B-v0.2 (With prompt) | 31.When cytological examination indicates ASC-US as an abnormal initial screening result and HPV is used for triage, if HPV is negative, the subsequent examination may depend on the individual's age, other risk factors, and the results of any previous screening tests. In some cases, a repeat cytological examination may be recommended in 12 months, while in others, no further testing may be necessary. | A | A | A |
| Mistral-7B-v0.2 | When cytological examination indicates ASC-US as an abnormal initial screening result, and HPV testing is used for triage, if HPV is negative, then a repeat Pap test in 12 months is recommended. However, some clinicians might opt for repeating the Pap test in 6 months instead. | A | A | A |
| Starling-LM-7B alpha (With prompt) | 31. When cytological examination indicates ASC-US as an abnormal initial screening result and HPV is used for triage, if HPV is negative, the subsequent examination should be a repeat cytology test in 6-12 months. | B | B | B |
| Starling-LM-7B alpha | If cytological examination indicates ASC-US as an abnormal initial screening result and HPV is used for triage, if HPV is negative, the subsequent examination should be regular screening in one year. | A | A | A |
| HuatuoGPT (With prompt) | 31. If cytology shows an abnormal initial screening result of ASC-US and HPV is used for shunting and if the HPV test result is negative, what should be the next step? If the cytology shows an abnormal initial screening result of ASC-US and the HPV test result is negative, updated guidelines now recommend regular checkups to monitor the situation and no further testing is needed. | B | C | B |
| HuatuoGPT | If cytology shows ASC-US as an abnormal initial screening result and HPV is used for triage and is negative for HPV, the next step is usually to have a periodic review. However, at the physician's discretion and depending on the patient's specific situation, additional testing, such as repeat cytology or HPV testing, may be necessary. | B | B | B |
| BioMedLM 2.7B (With prompt) | 31. When cytological examination indicates ASC-US as an abnormal initial screening result and HPV is used for triage, if HPV is negative, what should be the subsequent examination? The Bethesda System for Reporting Cervical Cytology (TBS) recommends that women with atypical squamous cells of undetermined significance (ASC-US) should be followed up with repeat cytology at 6 months and HPV testing at 12 months.[@B1] However, the guidelines do not provide clear recommendations for the management of women with ASC-US and a positive HPV test. | A | A | A |
| BioMedLM 2.7B | (1) Repeat cytology at 6 months; (2) repeat cytology at 12 months; or (3) colposcopy with biopsy. | C | C | C |
| Microsoft Phi2 (With prompt) | 31. When cytological examination indicates ASC-US as an abnormal initial screening result and HPV is used for triage, if HPV is negative, what should be the subsequent examination? The subsequent examination should be a repeat HPV test. | D | D | D |
| Microsoft Phi2 | If cytological examination shows ASC-US as an abnormal initial screening result and HPV is not used for triage, the patient will need additional tests or examinations depending on their risk factors and medical history. These may include repeat Pap test, HPV DNA testing, biopsy, etc. | C | D | D |
| QiZhenGPT (With prompt) | 31. If HPV is negative, a follow-up ASC-US test should be performed. | D | D | D |
| QiZhenGPT | When cytology indicates ASC-US as the initial screening result for an abnormality and sorting is performed using HPV, what is the next test if HPV is negative? A. When cytology indicates ASC-US as the initial screening result for an abnormality and sorting is performed using HPV, and if HPV is negative, cytology or palposcopy is usually required to determine the cause of the abnormality. | C | D | C |
| **32.When cytological examination results show ASC-H, LSIL, HSIL, is a colposcopy needed next?** | | | | |
| ChatGPT4.0 turbo (With prompt) | 32. Colposcopy for ASC-H, LSIL, or HSIL Results: Yes, a colposcopy is indeed needed next if the cytological examination results show atypical squamous cells - cannot exclude HSIL (ASC-H), low-grade squamous intraepithelial lesion (LSIL), or high-grade squamous intraepithelial lesion (HSIL). These findings suggest more significant cervical abnormalities that require detailed examination through colposcopy to determine the extent and nature of the abnormalities and to guide potential treatment options. | A | A | A |
| ChatGPT4.0 turbo | ASC-H, LSIL, HSIL on Cytology:  - ASC-H (atypical squamous cells, cannot exclude HSIL): A colposcopy is generally indicated.  - LSIL (low-grade squamous intraepithelial lesion): Management can depend on age and HPV test results. Colposcopy is often recommended, especially if HPV is positive or the patient's age and screening history suggest a higher risk.  - HSIL (high-grade squamous intraepithelial lesion): Immediate colposcopy is indicated due to the higher risk of precancerous or cancerous changes. | A | A | A |
| Claude 2 (With prompt) | 32. Yes, cytologic classifications of ASC-H, LSIL, or HSIL indicate abnormal cervical cells that are more definitive than ASC-US. Colposcopic examination is recommended following any of these results as per ASCCP guidelines. | A | A | A |
| Claude 2 | Yes, if cytology shows ASC-H, LSIL, or HSIL, referral for colposcopy is recommended as the next step. | A | A | A |
| Gemini Pro (With prompt) | 32. When cytological examination results show ASC-H, LSIL, or HSIL, is a colposcopy needed next? Answer: Yes | A | A | A |
| Gemini Pro | Yes, a colposcopy is needed next. | A | A | A |
| Mistral-7B-v0.2 (With prompt) | 32.When cytological examination results show ASC-H (atypical squamous cells cannot exclude HSIL), LSIL (low-grade squamous intraepithelial lesion), or HSIL (high-grade squamous intraepithelial lesion), a colposcopy is needed next. These abnormalities indicate the presence of precancerous changes in the cervix, and a colposcopy with biopsy can help to identify the extent and severity of the lesion, and to determine the appropriate treatment. | A | A | A |
| Mistral-7B-v0.2 | When cytological examination results show ASC-H (Atypical Squamous Cells, Cannot Exclude High-grade Squamous Intraepithelial Lesion), LSIL (Low-grade Squamous Intraepithelial Lesion), or HSIL (High-grade Squamous Intraepithelial Lesion), a colposcopy is recommended next, as these findings suggest that there is an abnormal growth of cells in the cervix, which typically requires further evaluation by colposcopy. | A | B | A |
| Starling-LM-7B alpha (With prompt) | 32. When cytological examination results show ASC-H, LSIL, or HSIL, a colposcopy is needed next. | A | A | A |
| Starling-LM-7B alpha | When cytological examination results show ASC-H, LSIL, or HSIL, a colposcopy is required as the next step. | A | B | A |
| HuatuoGPT (With prompt) | 32. If the cytology shows ASC-H, LSIL or HSIL, what tests should be performed next? If cytology results show ASC-H, LSIL or HSIL, updated guidelines now recommend colposcopy. During colposcopy, a biopsy of the abnormal area may be performed to further evaluate for the presence of cervical intraepithelial lesions. | A | A | A |
| HuatuoGPT | Colposcopy is usually recommended if cytology shows ASC-H, LSIL, or HSIL. A colposcopy can provide more information about the extent of cervical lesions and the risk of cancer, and help determine if further treatment is needed. | A | A | A |
| BioMedLM 2.7B (With prompt) | 32. When cytological examination results show ASC-H, LSIL, or HSIL, is a colposcopy needed next? Colposcopy is recommended for patients with ASC-H, LSIL, or HSIL. | A | A | A |
| BioMedLM 2.7B | Yes. | B | A | A |
| Microsoft Phi2 (With prompt) | 32. A colposcopy should be performed. | A | A | A |
| Microsoft Phi2 | If cytological examination shows ASC-H, LSIL, HSIL, a colposcopy will be recommended to examine the cervix more closely and look for signs of cervical cancer or precancerous changes. | A | A | A |
| QiZhenGPT (With prompt) | 32. If ASC-H, LSIL, or HSIL is detected, a colposcopy should be performed. | A | A | A |
| QiZhenGPT | Is colposcopy necessary when the cytology results indicate ASC-H, LSIL, HSIL? A. Colposcopy is usually needed when the cytology results indicate ASC-H, LSIL or HSIL. Colposcopy helps the doctor to see the changes in the cervical epithelium more clearly and to examine it histologically to determine the cause of the abnormality and to take appropriate treatment. | A | A | A |
| **33.When cytological examination results show AGC, what is the next examination required?** | | | | |
| ChatGPT4.0 turbo (With prompt) | 33. Next Examination for AGC (Atypical Glandular Cells): When cytological examination results show atypical glandular cells (AGC), a colposcopy is required as the next step. Additionally, endocervical sampling (either by curettage or with an endocervical brush) and sometimes endometrial sampling are recommended due to the potential for glandular abnormalities to originate from higher in the cervical canal or from within the endometrium. This comprehensive approach helps to rule out or diagnose more significant glandular diseases, including adenocarcinoma of the cervix or endometrium. | B | B | B |
| ChatGPT4.0 turbo | AGC on Cytology:  - Atypical glandular cells (AGC) can be associated with significant pathology, including glandular neoplasia.  - A colposcopy with endocervical sampling is typically required. Further evaluation may include endometrial sampling, especially in women over 35 years or those with additional risk factors for endometrial cancer. | B | B | B |
| Claude 2 (With prompt) | 33. AGC (atypical glandular cells) on cervical cytology warrants referral for diagnostic excisional procedure such as endocervical curettage or colposcopy with endocervical sampling. Further evaluation is required to exclude glandular abnormalities or cancer. | C | C | C |
| Claude 2 | When cytology shows atypical glandular cells (AGC), immediate colposcopy is recommended as the next examination, regardless if the AGC is endocervical, endometrial, or not otherwise specified. | B | A | B |
| Gemini Pro (With prompt) | 33. When cytological examination results show AGC, what is the next examination required? Colposcopy with biopsy | C | C | C |
| Gemini Pro | Colposcopy with endocervical curettage and/or endometrial biopsy. | B | B | B |
| Mistral-7B-v0.2 (With prompt) | 33.When cytological examination results show atypical glandular cells (AGC), the next examination required is a colposcopy with endocervical sampling or cone biopsy. AGC can indicate the presence of glandular precancer or cancer, and these procedures allow for a more thorough examination and biopsy of the cervix and endocervical canal. | B | B | B |
| Mistral-7B-v0.2 | When cytological examination results show AGC (Atypical Glandular Cells), additional tests are required to distinguish between reactive (benign) and neoplastic (precancerous or cancerous) atypical glandular cells. These tests may include repeat cytology, colposcopy, or endocervical curettage. Depending on the results of these tests, further examinations, such as endometrial biopsy or dilation and curettage, may be necessary. Consultation with a gynecologist is recommended for appropriate management. | C | B | B |
| Starling-LM-7B alpha (With prompt) | When cytological examination results show AGC (Atypical Glandular Cells), the next examination required is colposcopy with endocervical curettage (ECC) to further evaluate the abnormal cells. | A | B | B |
| Starling-LM-7B alpha | When cytological examination results show AGC, the next examination required is colposcopy with endocervical sampling. | B | B | B |
| HuatuoGPT (With prompt) | When the cytology result is AGC, what is the next step? When the cytology result is AGC, colposcopy is now recommended according to the latest guidelines. Colposcopy is an endoscopic examination that allows direct visualization of the cervix to look for abnormalities. If necessary, a biopsy may be performed to further evaluate the extent of the cytologic abnormality. | C | C | C |
| HuatuoGPT | If the cytology results show AGC, regular follow-up is usually recommended as the next step. However, depending on the doctor's judgment and the patient's specific situation, other tests, such as repeat cytology or HPV testing, may be necessary. In some cases, colposcopy or other diagnostic procedures may be needed to further assess the extent of cervical lesions and cancer risk. | C | D | D |
| BioMedLM 2.7B (With prompt) | When cytological examination results show AGC, what is the next examination required? The next examination is colposcopy. | C | C | C |
| BioMedLM 2.7B | 1) Endoscopy (gastroscopy or colonoscopy). 2) Biopsy and histopathological diagnosis of gastric cancer. 3) Examination for *Helicobacter pylori* infection. 4) Other examinations such as computed tomography scan, magnetic resonance imaging, positron emission tomography-computed tomography, and so on. 5) No further examination needed. | D | D | D |
| Microsoft Phi2 (With prompt) | 11. If cytological examination results show AGC, the next examination required is a colposcopy. | C | C | C |
| Microsoft Phi2 | If cytological examination results show AGC (atypical glandular cells), the patient will likely require a colposcopy due to the increased risk of developing cervical cancer. | A | B | A |
| QiZhenGPT (With prompt) | 11. When cytological examination results show AGC, what is the next examination required? AGC means that there is a high risk of developing cervical cancer. The next examination required would be a colposcopy to visualize the internal cervical os and identify any potential cancer cells. | C | C | C |
| QiZhenGPT | What is the next step when cytology shows AGC? A. When cytology results indicate AGC, cytology, histology, and HPV testing are usually required to determine the cause of the abnormality. | B | C | C |
| **34. If both cytological and high-risk HPV joint tests show negative results, what other examination should follow?** | | | | |
| ChatGPT4.0 turbo (With prompt) | Follow-up after Negative Cytology and High-Risk HPV Tests: If both cytological and high-risk HPV tests show negative results, typically no further examination is required in the immediate term. The patient should follow the standard screening intervals recommended by current guidelines, which generally suggest rescreening every three years with cytology alone or every five years with co-testing (cytology plus HPV testing) as long as results remain negative and no new risk factors develop. | A | A | A |
| ChatGPT4.0 turbo | Negative Cytology and HPV Test:  - If both the cytological examination and the HPV test are negative, the patient is usually considered at very low risk for cervical cancer. Typically, no further examination is needed, and the patient can return to routine screening at the interval recommended by the prevailing guidelines. | A | A | A |
| Claude 2 (With prompt) | If both cytology and high-risk HPV testing are negative, return to routine cervical cancer screening based on age and risk factors. No further evaluation is required. | C | C | C |
| Claude 2 | If both cytology and HPV tests are negative on joint testing, repeat co-testing in 3-5 years is recommended. | A | A | A |
| Gemini Pro (With prompt) | If both cytological and high-risk HPV joint tests show negative results, what other examination should follow? Repeat co-testing in 3 years | B | B | B |
| Gemini Pro | No further examination is typically recommended unless there are other risk factors or symptoms present. | B | A | B |
| Mistral-7B-v0.2 (With prompt) | If both cytological and high-risk HPV joint tests show negative results, no further examination is required at that time, unless the individual has other risk factors for cervical cancer. Regular follow-up appointments and screening tests are recommended based on age and other risk factors. | C | C | C |
| Mistral-7B-v0.2 | If both cytological and high-risk HPV results are negative, no further examination is usually required, unless the patient has other risk factors for cervical cancer or abnormal Pap tests in the past. An annual or more frequent follow-up may be recommended based on individual risk. | A | A | A |
| Starling-LM-7B alpha (With prompt) | If both cytological and high-risk HPV joint tests show negative results, the next examination that should follow is to repeat the cytological examination and HPV testing at a later date, as the negative results indicate a low risk of cervical cancer or precancerous conditions. | D | D | D |
| Starling-LM-7B alpha | If both cytological and high-risk HPV joint tests show negative results, it is reasonable to follow up with another round of cervical cancer screening within 3-5 years or sooner if symptoms appear or risk factors are present. | A | A | A |
| HuatuoGPT (With prompt) | If the combined cytology and high-risk HPV test results are negative, what tests should be performed next? If both the cytology and high-risk HPV co-test results are negative, regular check-ups to monitor the situation are now recommended according to the latest guidelines. Although cancer is not usually detected immediately in a negative result, regular follow-ups should still be performed to ensure that there is no progression of the underlying problem. | C | C | C |
| HuatuoGPT | If the combination of cytology and high-risk human papillomavirus (HR-HPV) tests are both negative, the next step is usually to have a regular pap smear. However, depending on the specific guidelines of your doctor or healthcare provider, other tests may also need to be considered, such as repeat pap smears or HPV testing. | C | C | C |
| BioMedLM 2.7B (With prompt) | If both cytological and high-risk HPV joint tests show negative results, what other examination should follow? The following examination is recommended for women with negative results on both tests. Cervical cytology** Cervical cytology is recommended for women with negative results on both tests. Cervical biopsy is recommended for women with negative results on both tests. Cervical colposcopy is recommended for women with negative results on both tests. Cervical conization is recommended for women with negative results on both tests. Cervical hysterectomy is recommended for women with negative results on both tests. Cervical loop electrosurgical excision procedure (LEEP)** Cervical LEEP is recommended for women with negative results on both tests. | D | D | D |
| BioMedLM 2.7B | 2) Colposcopy with biopsy (if necessary).3) Cytology test every 6 months for 2 years after treatment or until the patient has completed 5 years of follow up.4) High risk HPV DNA test at 12 month intervals during the first year after treatment and then annually thereafter.5) Pap smear every 3 to 4 years.6) VIA/VILI every 1--2 years. | D | D | D |
| Microsoft Phi2 (With prompt) | If both cytological and high-risk HPV joint tests show negative results, the next examination that should follow is colposcopy. | D | D | D |
| Microsoft Phi2 | If both cytological and high-risk HPV joint tests show negative results, additional screening may not be necessary at that time, but patients should continue regular screenings according to their age and risk factors. | A | A | A |
| QiZhenGPT (With prompt) | If both cytological and high-risk HPV joint tests show negative results, what other examination should follow? If both cytological and high-risk HPV joint tests show negative results, it is recommended that a follow-up test be performed to ensure that the results are accurate. This would be a colposcopy or a DNA test for high-risk HPV. | D | D | D |
| QiZhenGPT | If both cytology and high-risk HPV tests are negative, what other tests should be performed? A: If the combined cytology and high-risk HPV test results are both negative, one year of follow-up, including cytology and high-risk HPV testing, should be performed. Cytology and high-risk HPV testing should be performed regularly during the follow-up period. | B | C | B |
| **35.If joint testing of cytology and high-risk HPV shows HPV-negative and the cytological result is ASC-US, what should be done next?** | | | | |
| ChatGPT4.0 turbo (With prompt) | Management of ASC-US with Negative HPV in Joint Testing: When joint testing shows an ASC-US cytological result but a negative result for high-risk HPV, the risk of cervical cancer is considered low. The recommended follow-up is to repeat cytology testing in three years. This interval allows for adequate monitoring while minimizing unnecessary interventions. | C | C | C |
| ChatGPT4.0 turbo | HPV-Negative, ASC-US Cytology:  - If the HPV test is negative but cytology shows ASC-US, the risk of cervical cancer is low. The patient can either return to routine screening intervals or have a repeat Pap test in 3 years, depending on specific guidelines. | B | A | B |
| Claude 2 (With prompt) | For ASC-US cytology with a negative HPV test, repeat co-testing in 3 years per current ASCCP guidelines. The negative predictive value allows longer screening intervals. | B | B | B |
| Claude 2 | If HPV is negative but cytology shows ASC-US, repeat cytology in 12 months is appropriate. | B | C | B |
| Gemini Pro (With prompt) | If joint testing of cytology and high-risk HPV shows HPV-negative and the cytological result is ASC-US, what should be done next? Repeat co-testing in 1 year | A | A | A |
| Gemini Pro | Repeat cytology and HPV testing in 12 months. | A | A | A |
| Mistral-7B-v0.2 (With prompt) | If joint testing of cytology and high-risk HPV shows HPV-negative and the cytological result is ASC-US, no further examination is required at that time, unless the individual has other risk factors for cervical cancer. Regular follow-up appointments and screening tests are recommended based on age and other risk factors. | C | C | C |
| Mistral-7B-v0.2 | If the high-risk HPV result is negative but the cytology result is ASC-US, a repeat cytology test in 6-12 months is typically suggested. If the ASC-US persists, further testing such as colposcopy and/or HPV testing with genotyping may be necessary. | B | B | B |
| Starling-LM-7B alpha (With prompt) | If joint testing of cytology and high-risk HPV shows HPV-negative and the cytological result is ASC-US (Atypical Squamous Cells of Undetermined Significance), the next step is to repeat the cytological examination and HPV testing at a later date, as the negative HPV result reduces the likelihood of high-grade lesions. | D | D | D |
| Starling-LM-7B alpha | If joint testing of cytology and high-risk HPV shows HPV-negative and the cytological result is ASC-US (Atypical Squamous Cells of Undetermined Significance), a colposcopy should usually be performed to further assess the cervical abnormalities. | C | D | D |
| HuatuoGPT (With prompt) | If the combination of cytology and high-risk HPV test results are negative for HPV but the cytology result is ASC-US, what tests should be performed next? If the combined cytology and high-risk HPV test result is HPV negative but the cytology result is ASC-US, HPV triage testing is now recommended according to the latest guidelines. If the HPV test result is positive, colposcopy is recommended. If the HPV test result is negative, no further testing is required, but periodic testing is still recommended to monitor the situation. | B | B | B |
| HuatuoGPT | Regular pap smears are usually recommended if the combined cytology and HR-HPV test result is HPV-negative and the cytology result is ASC-US. This helps to monitor cytologic changes and further interventions if necessary. | D | C | C |
| BioMedLM 2.7B (With prompt) | If joint testing of cytology and high-risk HPV shows HPV-negative and the cytological result is ASC-US, what should be done next? The answer is ASC-US. The patient should be referred to colposcopy. | D | D | D |
| BioMedLM 2.7B | Repeat cytology in 6 months. | B | B | B |
| Microsoft Phi2 (With prompt) | If joint testing of cytology and high-risk HPV shows HPV-negative and the cytological result is ASC-US, the next examination that should be done is colposcopy. | D | D | D |
| Microsoft Phi2 | If joint testing of cytology and high-risk HPV shows HPV-negative and the cytological result is ASC-US, further evaluation with colposcopy is recommended. | C | D | C |
| QiZhenGPT (With prompt) | If joint testing of cytology and high-risk HPV shows HPV-negative and the cytological result is ASC-US, what should be done next? If joint testing of cytology and high-risk HPV shows HPV-negative and the cytological result is ASC-US, it means that there is no evidence of cervical cancer or high-risk HPV infection. The next examination would be a colposcopy to visualize the internal cervical os and identify any potential cancer cells. | D | C | D |
| QiZhenGPT | If the combined cytology and high-risk HPV test result shows HPV-negative and the cytology result is ASC-US, what should be done next? A. If the combined cytology and high-risk HPV test result shows HPV-negative and the cytology result is ASC-US, colposcopy and HPV testing should be performed. | D | C | C |
| **36.If joint testing of cytology and high-risk HPV shows HPV-negative, and the cytological result is >ASC-US, what should be done next? Is a colposcopy required?** | | | | |
| ChatGPT4.0 turbo (With prompt) | Management of >ASC-US with Negative HPV in Joint Testing: If the cytology result is more severe than ASC-US (e.g., LSIL, HSIL) but the HPV test is negative, a colposcopy is recommended. This is because the cytological findings suggest a possible high-grade lesion that might not be associated with high-risk HPV types detectable by the test or might represent a sampling or testing error. A colposcopy allows for a detailed examination of the cervical epithelium and targeted biopsies if necessary. | B | A | A |
| ChatGPT4.0 turbo | HPV-Negative, >ASC-US Cytology:  - If the HPV test is negative and the cytological result shows abnormalities greater than ASC-US (e.g., LSIL or HSIL), a colposcopy is generally indicated to further evaluate the abnormal cells. | A | A | A |
| Claude 2 (With prompt) | If cytology shows ASC-H, LSIL, HSIL or more severe abnormalities with negative HPV, colposcopy is still recommended despite the discordant result. The cytologic abnormality requires further evaluation to rule out precancer/cancer. | A | A | A |
| Claude 2 | If HPV is negative but cytology is >ASC-US (e.g. LSIL or worse), referral for colposcopy is still required as the next step. | A | A | A |
| Gemini Pro (With prompt) | If joint testing of cytology and high-risk HPV shows HPV-negative and the cytological result is >ASC-US, what should be done next? Is a colposcopy required? Yes, a colposcopy is required. | A | A | A |
| Gemini Pro | Yes, a colposcopy is typically recommended. | A | A | A |
| Mistral-7B-v0.2 (With prompt) | If joint testing of cytology and high-risk HPV shows HPV-negative and the cytological result is >ASC-US, a repeat cytological examination in 12 months is recommended. If the repeat cytological examination is also abnormal, or if the individual has other risk factors for cervical cancer, a colposcopy may be recommended. | C | C | C |
| Mistral-7B-v0.2 | If the high-risk HPV result is negative, and the cytological result is >ASC-US, a repeat cytology test should be done within 12 months. If the abnormality persists, colposcopy and/or HPV testing with genotyping are needed for more information. Colposcopy is recommended especially if the cytology result is HSIL (high-grade squamous intraepithelial lesion). | A | A | A |
| Starling-LM-7B alpha (With prompt) | If joint testing of cytology and high-risk HPV shows HPV-negative and the cytological result is >ASC-US (more severe than ASC-US), a colposcopy is not necessarily required. However, the patient should be closely monitored, and repeat cytological examination and HPV testing should be performed at a later date to assess any changes in the cervical cells. | D | D | D |
| Starling-LM-7B alpha | If joint testing of cytology and high-risk HPV shows HPV-negative and the cytological result is >ASC-US (a more significant cytological abnormality), a colposcopy is generally recommended to further assess the cervical abnormalities. However, appropriate management should be determined by a healthcare professional based on the patient's age, risk factors, and specific cytological results. | A | A | A |
| HuatuoGPT (With prompt) | If the combined cytology and high-risk HPV test result is HPV-negative but the cytology result is >ASC-US, what tests should be performed next? If the combined cytology and high-risk HPV test result is HPV-negative but the cytology result is >ASC-US, HPV triage testing is now recommended according to the latest guidelines. If the HPV test result is positive, colposcopy is recommended. If the HPV test result is negative, no further testing is required, but periodic testing is still recommended to monitor the situation. | C | B | C |
| HuatuoGPT | If the combined cytology and HR-HPV test result is HPV negative and the cytology result is ASC-US or higher, regular pap smears are usually recommended. This helps to monitor cytologic changes and further interventions if necessary. In some cases, repeat testing or additional testing, such as HPV testing, may be needed to further evaluate cytologic changes. | D | B | D |
| BioMedLM 2.7B (With prompt) | If joint testing of cytology and high-risk HPV shows HPV-negative and the cytological result is >ASC-US, what should be done next? Is a colposcopy required? Colposcopy is recommended for patients with a cytological result of ASC-US or worse. | A | A | A |
| BioMedLM 2.7B | Colposcopy is recommended for women with ASC-US or worse on cytology. Women who are HPV positive but have normal cytology can be followed up by repeat cytology in 6 months to 2 years depending upon their age. | A | A | A |
| Microsoft Phi2 (With prompt) | 14.If joint testing of cytology and high-risk HPV shows HPV-negative and the cytological result is >ASC-US, the next examination that should be done is colposcopy. A colposcopy is not required. | D | D | D |
| Microsoft Phi2 | If joint testing of cytology and high-risk HPV shows HPV-negative, and the cytological result is >ASC-US, colposcopy is still recommended to evaluate the abnormal cells and determine whether they are precancerous or benign. | A | A | A |
| QiZhenGPT (With prompt) | If joint testing of cytology and high-risk HPV shows HPV-negative and the cytological result is >ASC-US, what should be done next? Is a colposcopy required? If joint testing of cytology and high-risk HPV shows HPV-negative and the cytological result is >ASC-US, it means that there is no evidence of cervical cancer or high-risk HPV infection. However, it is recommended to perform a colposcopy to rule out any potential issues with the test results. | A | A | A |
| QiZhenGPT | If the combined cytology and high-risk HPV test result shows HPV-negative and the cytology result is >ASC-US, what should be done next? Is colposcopy necessary? A: If the combined cytology and high-risk HPV test result shows HPV-negative and the cytology result is >ASC-US, colposcopy should be performed. The need for further testing depends on the circumstances and physician judgment. | B | A | A |
| **37.In cervical cancer detection, if joint testing of cytology and high-risk HPV shows HPV-positive, and the cytological result is ≥ASC-US, what specific examinations should follow?** | | | | |
| ChatGPT4.0 turbo (With prompt) | Specific Examinations after Positive HPV and ≥ASC-US Cytology in Joint Testing: When joint testing results in a positive high-risk HPV test and a cytological result of ≥ASC-US, a colposcopy is indicated. This combination of findings suggests a significant risk of cervical intraepithelial neoplasia or cervical cancer. During the colposcopy, careful inspection of the cervix, vagina, and vulva is performed, and biopsies of any suspicious areas are taken to determine the appropriate diagnosis and subsequent management. | A | A | A |
| ChatGPT4.0 turbo | HPV-Positive, ≥ASC-US Cytology:  - If the HPV test is positive and the cytology is ASC-US or higher, a colposcopy should be performed to directly visualize the cervix and potentially take biopsies of any suspicious areas. | A | A | A |
| Claude 2 (With prompt) | 37. Concurrent positive high-risk HPV and ASC-US or greater cytologic abnormality indicates an increased risk of precancer/cancer. Next step is referral for colposcopy with biopsy as warranted to further evaluate abnormal cervical tissue. Diagnostic excision may also be indicated based on colposcopy findings. | A | A | A |
| Claude 2 | If HPV is positive and cytology is ≥ASC-US on co-testing, referral for colposcopy is recommended. | A | A | A |
| Gemini Pro (With prompt) | 37. In cervical cancer detection, if joint testing of cytology and high-risk HPV shows HPV-positive and the cytological result is ≥ASC-US, what specific examinations should follow? Colposcopy with biopsy and endocervical curettage | B | B | B |
| Gemini Pro | Colposcopy with biopsy. | D | C | C |
| Mistral-7B-v0.2 (With prompt) | 37.In cervical cancer detection, if joint testing of cytology and high-risk HPV shows HPV-positive and the cytological result is ≥ASC-US, a colposcopy with biopsy is required. The presence of high-risk HPV types and abnormal cytological findings indicate the presence of precancerous or cancerous changes, and a colposcopy with biopsy can help to identify the extent and severity of the lesion, and to determine the appropriate treatment. | A | A | A |
| Mistral-7B-v0.2 | If the high-risk HPV result is positive, and the cytological result is ≥ASC-US, a colposcopy with a biopsy is recommended to assess the extent and severity of the lesion. This is due to the increased risk of cervical cancer associated with HPV positivity and an abnormal cytology result. | A | A | A |
| Starling-LM-7B alpha (With prompt) | 37. In cervical cancer detection, if joint testing of cytology and high-risk HPV shows HPV-positive and the cytological result is ≥ASC-US (more severe than ASC-US), the specific examinations that should follow include:   a. Colposcopy: To visualize the cervix and identify any abnormal areas that may require biopsy.     b. Biopsy: If any abnormal areas are identified during colposcopy, a biopsy should be performed to obtain tissue samples for histopathological examination.     c. Histopathological examination: The tissue samples obtained from the biopsy should be examined by a pathologist to determine the presence or absence of cervical dysplasia or cancer.    d. Further imaging studies: Depending on the results of the histopathological examination, additional imaging studies such as magnetic resonance imaging (MRI) or computed tomography (CT) scans may be required to assess the extent of the disease and determine the appropriate treatment plan. | B | B | B |
| Starling-LM-7B alpha | If joint testing of cytology and high-risk HPV shows HPV-positive and the cytological result is ≥ASC-US (a significant cytological abnormality), a colposcopy should generally be performed to further assess the cervical abnormalities. Additionally, further HPV genotyping and/or repeat testing may be conducted as clinically indicated. | B | B | B |
| HuatuoGPT (With prompt) | 37. In cervical cancer testing, if the combined cytology and high-risk HPV test result is positive for HPV but the cytology result is ≥ASC-US, what test should be performed next? If the combined cytology and high-risk HPV test result is HPV-positive but the cytology result is ≥ASC-US, colposcopy is now recommended according to the latest guidelines. During colposcopy, a biopsy of the abnormal area may be performed to further evaluate the extent of the cytologic abnormality. If combined testing of cytology and high-risk human papillomavirus (HPV) shows HPV positivity and cytology results are negative, what specific tests should be performed next? Ans: In this case, colposcopy should be offered. | A | A | A |
| HuatuoGPT | Colposcopy is usually recommended if the combination of cytology and HR-HPV results are HPV-positive and the cytology is ≥ASC-US. Colposcopy can provide more information about the extent of cervical lesions and cancer risk and help determine if further treatment is needed. | A | B | A |
| BioMedLM 2.7B (With prompt) | 37. In cervical cancer detection, if joint testing of cytology and high-risk HPV shows HPV-positive and the cytological result is ≥ASC-US, what specific examinations should follow? The answer to this question is based on the results of the study by Kim et al. \[[@b1-jptm-2017-01-08]\]. In this study, the authors compared the results of HPV testing and cytology in 1,824 women who underwent cervical cancer screening. The sensitivity of HPV testing was higher than that of cytology (96.3% vs. 81.8%), and the specificity of HPV testing was lower than that of cytology (62.8% vs. 90.6%). The positive predictive value (PPV) of HPV testing was higher than that of cytology (30.6% vs. 16.7%), and the negative predictive value (NPV) of HPV testing was lower than that of cytology (98.9% vs. 99.6%). The authors concluded that HPV testing is more sensitive than cytology, but less specific. In addition, the PPV of HPV testing was higher than that of cytology, but the NPV of HPV testing was lower than that of cytology. | D | D | D |
| BioMedLM 2.7B | Colposcopy. | A | A | A |
| Microsoft Phi2 (With prompt) | 37.In cervical cancer detection, if joint testing of cytology and high-risk HPV shows HPV-positive and the cytological result is ≥ASC-US, the specific examinations that should follow are a colposcopy and a biopsy. | A | A | A |
| Microsoft Phi2 | In cervical cancer detection, if joint testing of cytology and high-risk HPV shows HPV-positive, and the cytological result is ≥ASC-US, colposcopy followed by biopsy of the suspicious areas is typically performed to confirm the diagnosis. | D | C | C |
| QiZhenGPT (With prompt) | 37. In cervical cancer detection, if joint testing of cytology and high-risk HPV shows HPV-positive and the cytological result is ≥ASC-US, what specific examinations should follow? If joint testing of cytology and high-risk HPV shows HPV-positive and the cytological result is ≥ASC-US, it means that there is evidence of cervical cancer or high-risk HPV infection. The specific examinations that should follow would depend on the individual's clinical presentation and the recommendations of the healthcare provider. Some options may include a colposcopy, a DNA test for high-risk HPV, a routine ASC-US test, or a follow-up ASC-US test after a year or two. | C | D | D |
| QiZhenGPT | In cervical cancer screening, if the combined cytology and high-risk HPVtest result shows HPV-positive and the cytology result is ≥ASC-US, what tests should be performed next? A: If, in cervical cancer screening, the combined cytology and high-risk HPV test result shows HPV-positive and the cytology result is ≥ASC-US, the following tests should be performed: first, a v test should be performed to determine if one is infected with human papillomavirus (HPV). Second, cervical cytology and HPV testing should be performed to determine the presence of cervical intraepithelial neoplasia (CIN). If the CIN level is ≥CIN 2 or CIN 3, a cervical biopsy should be performed to determine if there is a risk of cervical cancer. | D | C | D |
| **38.In cervical cancer detection, if joint testing of cytology and high-risk HPV shows HPV-positive and the cytological result is negative, what specific examinations should follow?** | | | | |
| ChatGPT4.0 turbo (With prompt) | 38. Follow-up for HPV-Positive and Negative Cytology Without Specific Typing: When joint testing of cytology and high-risk HPV shows a positive HPV result but negative cytology, the recommended follow-up involves repeat co-testing in one year. This approach allows for monitoring the potential development of cytological abnormalities while acknowledging the presence of high-risk HPV, which could lead to cervical changes over time. | B | B | B |
| ChatGPT4.0 turbo | HPV-Positive, Negative Cytology:  - If the HPV test is positive but cytology is negative, management may depend on the patient's age and specific HPV type. Some guidelines recommend repeat co-testing in one year, while others may suggest immediate colposcopy, especially if the patient is over a certain age or if the HPV test indicates a high-risk type. | A | A | A |
| Claude 2 (With prompt) | 38. For a positive high-risk HPV result with normal cytology, repeat co-testing in 1 year. Persistent infection (2 years or more) warrants colposcopy despite continued normal cytology. | D | C | C |
| Claude 2 | If HPV is positive but cytology is negative, repeat co-testing in 12 months is appropriate. | B | D | C |
| Gemini Pro (With prompt) | 38. In cervical cancer detection, if joint testing of cytology and high-risk HPV shows HPV-positive and the cytological result is negative, what specific examinations should follow? Repeat co-testing in 1 year | C | D | C |
| Gemini Pro | Repeat cytology and HPV testing in 6 months. | C | C | C |
| Mistral-7B-v0.2 (With prompt) | 38.In cervical cancer detection, if joint testing of cytology and high-risk HPV shows HPV-positive and the cytological result is negative, a repeat cytological examination in 12 months is recommended. If the repeat cytological examination is also negative, and the individual has no other risk factors for cervical cancer, no further testing may be necessary. However, regular follow-up appointments and screening tests are recommended based on age and other risk factors. | C | C | C |
| Mistral-7B-v0.2 | If the high-risk HPV result is positive, but the cytological result is negative, further testing is needed because the presence of HPV does not always indicate cervical cancer or precancer. A repeat cytology test in 12 months is typically suggested, or colposcopy and/or HPV testing with genotyping. The need for further testing depends on the individual patient's age, HPV type, and other risk factors. | C | C | C |
| Starling-LM-7B alpha (With prompt) | 38. In cervical cancer detection, if joint testing of cytology and high-risk HPV shows HPV-positive and the cytological result is negative, the following specific examinations should follow:   a. Colposcopy with cervical biopsy: This is the most appropriate next step to further evaluate the HPV-positive, cytology-negative result. Colposcopy allows for visualization of the cervix and identification of any abnormal areas, while cervical biopsy can help confirm or rule out the presence of cervical dysplasia or cancer.   b. HPV genotyping: If the HPV typing is not already known, genotyping can help identify the specific HPV subtypes present, which may influence the management plan. | B | A | B |
| Starling-LM-7B alpha | If joint testing of cytology and high-risk HPV shows HPV-positive and the cytological result is negative, a colposcopy should generally be performed to further assess the cervical abnormalities. Additionally, further HPV genotyping and/or repeat testing may be conducted as clinically indicated. | B | A | B |
| HuatuoGPT (With prompt) | 38. If the combined test cytology and high-risk human papillomavirus (HPV) show HPV positivity and the cytology is negative and the HPV typing is HPV 16/18 positive, what specific tests should be offered next? Answer: In this case, colposcopy should be offered and considered. | C | B | B |
| HuatuoGPT | Repeat cytology or additional HPV testing is usually recommended if the combination of cytology and HR-HPV results are HPV-positive and cytology is negative. This helps to further evaluate the cytologic changes and determine if an abnormality is present. In some cases, colposcopy or other diagnostic procedures may be necessary to further evaluate cytologic changes and cancer risk. | A | A | A |
| BioMedLM 2.7B (With prompt) | 38. In cervical cancer detection, if joint testing of cytology and high-risk HPV shows HPV-positive and the cytological result is negative, what specific examinations should follow? The answer to this question is based on the results of the study by Kim et al. \[[@b1-jptm-2017-03-08]\]. The authors reported that the sensitivity of cytology was higher than that of high-risk HPV testing in detecting cervical intraepithelial neoplasia (CIN) 2 or worse (CIN2+) in women with negative high-risk HPV results. In addition, the sensitivity of cytology was higher than that of high-risk HPV testing in detecting CIN3+ in women with negative cytology results. The sensitivity of cytology was higher than that of high-risk HPV testing in detecting CIN2+ in women with negative high-risk HPV results. The sensitivity of cytology was higher than that of high-risk HPV testing in detecting CIN3+ in women with negative cytology results. | D | D | D |
| BioMedLM 2.7B | 1) Repeat cytology at 6 months; 2) repeat cytology every year for 3 years or until the patient has three consecutive normal results (repeated cytology); 3) colposcopy with biopsy; 4) repeat cytology after 1 month; 5) repeat cytology every year for 3 years or until the patient has three consecutive normal results (repeated cytology). | C | C | C |
| Microsoft Phi2 (With prompt) | 38.If joint testing of cytology and high-risk HPV shows HPV-positive and the cytological result is negative, the patient should undergo colposcopy with biopsy. | C | C | C |
| Microsoft Phi2 | In cervical cancer detection, if joint testing of cytology and high-risk HPV shows HPV-positive and the cytological result is negative, repeat testing within 3 months may be considered depending on the individual case and risk factors. | D | D | D |
| QiZhenGPT (With prompt) | 38. In cervical cancer detection, if joint testing of cytology and high-risk HPV shows HPV-positive and the cytological result is negative, what specific examinations should follow? Answer: Additional testing for high-risk HPV, such as a DNA test for HPV16 and HPV18. | B | B | B |
| QiZhenGPT | In cervical cancer screening, if the combined cytology and high-risk HPV test result shows HPV-positive and the cytological result is negative, what tests should be performed? A. If the combined cytology and high-risk HPV test result shows HPV-positive and the cytological result is negative during cervical cancer screening, a one-year follow-up, including cytology and high-risk HPV testing, should be performed. Cytology and high-risk HPV testing should be performed regularly during the follow-up period. If abnormalities are detected during follow-up, further investigations and treatment should be performed. | D | C | D |
| **39.In cervical cancer detection, if joint testing of cytology and high-risk HPV shows HPV-positive and the cytological result is negative, and the HPV typing is HPV16/18 positive, what specific examinations should follow?** | | | | |
| ChatGPT4.0 turbo (With prompt) | 39. Follow-up for HPV16/18 Positive with Negative Cytology: If joint testing results in a positive result for HPV types 16 or 18—the types most strongly associated with cervical cancer—but cytology is negative, a colposcopy should be performed. This is due to the higher risk of significant cervical disease associated with these HPV types, which may not yet be evident in the cytology. | A | A | A |
| ChatGPT4.0 turbo | HPV-Positive for HPV16/18, Negative Cytology:  - If HPV typing shows a positive result for HPV16 or HPV18, which are known to be associated with the highest risk of cervical cancer, a colposcopy is typically recommended, even if the cytology is negative. | A | A | A |
| Claude 2 (With prompt) | 39. For confirmed HPV 16/18 infection with normal cytology, referral for immediate colposcopy is recommended given the higher cancer risk associated with these genotypes, regardless of cytology findings. | A | A | A |
| Claude 2 | If HPV16/18 is specifically positive even with negative cytology, guidelines often recommend proceeding directly to colposcopy. | A | A | A |
| Gemini Pro (With prompt) | 39. In cervical cancer detection, if joint testing of cytology and high-risk HPV shows HPV-positive and the cytological result is negative, and the HPV typing is HPV16/18 positive, what specific examinations should follow? Colposcopy | A | A | A |
| Gemini Pro | Colposcopy with biopsy | C | C | C |
| Mistral-7B-v0.2 (With prompt) | 39.In cervical cancer detection, if joint testing of cytology and high-risk HPV shows HPV-positive and the cytological result is negative, but the HPV typing is HPV16/18 positive, a colposcopy with biopsy is recommended. HPV16 and HPV18 are the two most common high-risk HPV types associated with cervical cancer, and the presence of these types increases the likelihood of precancerous or cancerous changes, even if the cytological examination is negative. | B | A | A |
| Mistral-7B-v0.2 | If the high-risk HPV result is positive, the cytological result is negative, and HPV typing identifies HPV16 or HPV18, a colposcopy with biopsy is commonly recommended due to the higher risk of cervical cancer associated with these HPV types. This is usually the case even if the cytology result is negative. | A | A | A |
| Starling-LM-7B alpha (With prompt) | 39. In cervical cancer detection, if joint testing of cytology and high-risk HPV shows HPV-positive and the cytological result is negative, and the HPV typing is HPV16/18 positive, the following specific examinations should follow:   a. Colposcopy with cervical biopsy: As mentioned earlier, this is the most appropriate next step to further evaluate the HPV-positive, cytology-negative result.   b. HPV genotyping: Although the HPV typing is already known to be HPV16/18 positive, genotyping can help confirm the presence of these specific subtypes and rule out any co-infections. | B | B | B |
| Starling-LM-7B alpha | If joint testing of cytology and high-risk HPV shows HPV-positive, the cytological result is negative, and there'HPV16/18 positive, a colposcopy should generally be performed to further assess the cervical abnormalities. Additionally, further HPV genotyping and/or repeat testing may be conducted. | A | A | A |
| HuatuoGPT (With prompt) | 39. In cervical cancer testing, if a patient is HPV positive by combined test cytology and high-risk human papillomavirus (HPV), but has a negative cytology result and is not typed for HPV 16 or HPV 18, are there any other tests that are needed? Answer: In this case, colposcopy should be offered and typing tests for high-risk human papillomavirus (HPV) should be considered. | A | A | A |
| HuatuoGPT | Colposcopy is usually recommended if the combined cytology and HR-HPV test result is HPV positive and the cytology result is negative and the HPV type is HPV 16/18 positive. Colposcopy can provide more information about the extent of cervical lesions and the risk of cancer, and help determine if further treatment is needed. In some cases, repeat cytology or additional HPV testing may be necessary to further evaluate cytologic changes and determine if abnormalities are present. | A | A | A |
| BioMedLM 2.7B (With prompt) | 39. In cervical cancer detection, if joint testing of cytology and high-risk HPV shows HPV-positive and the cytological result is negative, and the HPV typing is HPV16/18 positive, what specific examinations should follow? The answer is "Colposcopy." | A | A | A |
| BioMedLM 2.7B | 1) Repeat cytology at 6 months; 2) repeat cytology every year for 3 years after treatment or until the patient has completed her childbearing age (25--30); 3) perform colposcopy with biopsy when the cytology results are abnormal; 4) perform colposcopy without biopsy in cases where the cytology results are normal but the HPV test is positive; 5) perform colposcopy only when the cytology results are abnormal regardless of whether the HPV test was performed previously. | D | D | D |
| Microsoft Phi2 (With prompt) | 39.If joint testing of cytology and high-risk HPV shows HPV-positive and the cytological result is negative, and the HPV typing is HPV16/18 positive, the patient should undergo colposcopy with biopsy. | A | A | A |
| Microsoft Phi2 | In cervical cancer detection, if joint testing of cytology and high-risk HPV shows HPV-positive and the cytological result is negative, HPV typing for HPV16 or HPV18 may be useful to identify specific subtypes associated with higher risk of developing invasive disease. | D | D | D |
| QiZhenGPT (With prompt) | 39. In cervical cancer detection, if joint testing of cytology and high-risk HPV shows HPV-positive and the cytological result is negative, and the HPV typing is HPV16/18 positive, what specific examinations should follow? Answer: Additional testing for high-risk HPV, such as a DNA test for HPV16 and HPV18. | D | D | D |
| QiZhenGPT | During cervical cancer screening, if the combined cytology and high-risk HPV test result shows HPV-positive, the cytological result is negative, and the HPV typing is HPV16/18 positive, what test should be performed next? A: If at cervical cancer screening, the combined cytology and high-risk HPV test result shows HPV-positive, the cytological result is negative, and the HPV typing is HPV16/18 positive, further testing and treatment should be performed. Specific tests include cervical biopsy and HPV 16/18 typing. Cervical biopsy can help the doctor better observe the cervical lesions, while HPV16/18 typing can help the doctor determine the specific type of HPV infection so that a more personalized treatment plan can be developed. If the pathology results are malignant, further treatment should be carried out. | D | D | D |
| **40.In cervical cancer detection, if a patient has joint testing of cytology and high-risk HPV showing HPV-positive, the cytological result is negative, and there's no typing for HPV16 or HPV18, are there any other tests needed?** | | | | |
| ChatGPT4.0 turbo (With prompt) | 40. Management When HPV is Positive, Cytology Negative, Without HPV16/18 Typing: If the HPV test is positive, the cytology is negative, and there is no specific typing for HPV16 or HPV18, the next step typically involves repeat co-testing in one year. This recommendation is based on the presence of high-risk HPV that could potentially lead to cervical abnormalities, warranting close surveillance. | A | A | A |
| ChatGPT4.0 turbo | HPV-Positive, Negative Cytology, Non-16/18 Types:  - If the HPV test is positive for types other than 16 or 18 and cytology is negative, the recommended follow-up may include repeat co-testing in one year or a colposcopy, depending on the patient's age, the specific HPV type, and the guidelines being followed. | A | A | A |
| Claude 2 (With prompt) | 40. If high-risk HPV is positive but specific genotype information is unavailable, manage similarly - repeat co-testing in 1 year looking for persistent infection before referring for colposcopy. Genotyping may be done concurrently at the 1 year visit if not already performed. | B | B | B |
| Claude 2 | If high-risk HPV is positive, cytology is negative, and no typing for 16/18, then repeat co-testing in 12 months is still warranted to monitor for persistence of HPV infection or cytologic progression. | A | A | A |
| Gemini Pro (With prompt) | 40. In cervical cancer detection, if a patient has joint testing of cytology and high-risk HPV showing HPV-positive, the cytological result is negative, and there's no typing for HPV16 or HPV18, are there any other tests needed? No, no further tests are needed. | C | C | C |
| Gemini Pro | Yes, HPV genotyping may be recommended to determine if the patient is positive for HPV16 or HPV18. | D | D | D |
| Mistral-7B-v0.2 (With prompt) | 40. In cervical cancer detection, if a patient has joint testing of cytology and high-risk HPV showing HPV-positive, the cytological result is negative, and there's no typing for HPV16 or HPV18, a repeat cytological examination in 12 months is recommended. If the repeat cytological examination is also negative, and the individual has no other risk factors for cervical cancer, no further testing may be necessary. However, regular follow-up appointments and screening tests are recommended based on age and other risk factors. | A | B | B |
| Mistral-7B-v0.2 | If the high-risk HPV result is positive, the cytology result is negative, and there is no typing for HPV16 or HPV18, a repeat cytology test may be suggested, with further testing (colposcopy and/or HPV genotyping) considered based on individual risk factors. | D | D | D |
| Starling-LM-7B alpha (With prompt) | 40. In cervical cancer detection, if a patient has joint testing of cytology and high-risk HPV showing HPV-positive, the cytological result is negative, and there's no typing for HPV16 or HPV18, the following tests may be needed:   a. HPV genotyping: This test can help identify the specific HPV subtypes present, which may influence the management plan.   b. Colposcopy with cervical biopsy: If the HPV genotyping results are positive for high-risk HPV subtypes other than HPV16 or HPV18, colposcopy and cervical biopsy should be performed to further evaluate the presence of cervical dysplasia or cancer. | D | D | D |
| Starling-LM-7B alpha | If a patient has joint testing of cytology and high-risk HPV showing HPV-positive, the cytological result is negative, and there's no typing for HPV16 or HPV18, it's recommended to perform HPV genotyping to determine the specific HPV subtype, as some subtypes have a higher risk of progressing to cervical cancer. If the patient is advised to return for follow-up testing after a certain period, further colposcopy, biopsy, or repeat HPV testing may be needed depending on the result of the HPV genotyping. | C | C | C |
| HuatuoGPT (With prompt) | 40. In cervical cancer screening, if a patient has a confirmatory pathology (biopsy) that shows LSIL cytologic findings of LSIL or greater and a TZ3 classification, what are the next follow-up steps that should be performed? Answer: colposcopy should be performed and a colposcopic biopsy should be considered. | D | D | D |
| HuatuoGPT | Repeat cytology or additional HPV testing is usually recommended if a patient has undergone combined cytology and HR-HPV testing with positive results for HPV and negative cytology results, respectively, and has not been typed for HPV16 or HPV18. This helps to further evaluate cytologic changes and determine if abnormalities are present. In some cases, colposcopy or other diagnostic procedures may be necessary to further evaluate cytologic changes and cancer risk. | C | C | C |
| BioMedLM 2.7B (With prompt) | 40. In cervical cancer detection, if a patient has joint testing of cytology and high-risk HPV showing HPV-positive, the cytological result is negative, and there's no typing for HPV16 or HPV18, are there any other tests needed? Yes, there are other tests that can be used in addition to cytology and HPV testing. The most common tests are colposcopy and biopsy. If the patient has a positive result for HPV16 or HPV18, the patient should be referred to colposcopy. If the patient has a negative result for HPV16 or HPV18, the patient should be referred to cytology. If the patient has a positive result for HPV16 or HPV18 and a negative result for cytology, the patient should be referred to colposcopy. If the patient has a negative result for HPV16 or HPV18 and a positive result for cytology, the patient should be referred to colposcopy. If the patient has a positive result for HPV16 or HPV18 and a positive result for cytology, the patient should be referred to colposcopy. | D | D | D |
| BioMedLM 2.7B | Yes, we need to do colposcopy in this case because it is positive on cytology but not on HPV test. | D | D | D |
| Microsoft Phi2 (With prompt) | 40. If a patient has joint testing of cytology and high-risk HPV showing HPV-positive, the cytological result is negative, and there's no typing for HPV16 or HPV18, the patient should undergo colposcopy with biopsy. | D | D | D |
| Microsoft Phi2 | In cervical cancer detection, if a patient has joint testing of cytology and high-risk HPV showing HPV-positive, the cytological result is negative, and there's no typing for HPV16 or HPV18, other diagnostic tests such as liquid-based cytology or Pap smear may be used to obtain further information about the abnormal cells. However, these tests have lower sensitivity than the combined test and may miss some cases of cervical abnormalities. Regular monitoring with follow-up exams is important to detect any changes in the cervix over time. | D | D | D |
| QiZhenGPT (With prompt) | 40. In cervical cancer detection, if a patient has joint testing of cytology and high-risk HPV showing HPV-positive, the cytological result is negative, and there's no typing for HPV16 or HPV18, are there any other tests needed? Answer:. No additional tests are needed. | C | C | C |
| QiZhenGPT | In cervical cancer screening, if a patient undergoes combined cytology and high-risk HPV testing that shows HPV-positive with negative cytology results and no HPV 16 or 18 typing detected, are additional tests necessary? A: In this case, other relevant tests such as immunologic testing, cytology repeat testing, or trans-negative testing may be required. The specific testing needs depend on the patient's condition and the doctor's judgment. | D | D | D |
| **41.In cervical cancer screening, if a patient has a histopathological confirmation(biopsy) of LSIL, cytology findings of LSIL or higher, and a TZ3 classification, what follow-up steps should be conducted?** | | | | |
| ChatGPT4.0 turbo (With prompt) | 41. Follow-up for LSIL with TZ3 Classification: When a patient has a histopathological confirmation of low-grade squamous intraepithelial lesion (LSIL) or higher with a transformation zone 3 (TZ3) classification, careful follow-up is required due to the potential for missed lesions. A repeat colposcopy is recommended, ideally with endocervical sampling, to ensure that no higher-grade disease is present in the areas that are harder to visualize or sample, such as the upper endocervical canal. | A | A | A |
| ChatGPT4.0 turbo | LSIL Histopathology, LSIL or Higher Cytology, TZ3 Classification:  - For a patient with confirmed LSIL on biopsy and LSIL or higher on cytology, with a TZ3 classification (where the entire transformation zone is not visible), follow-up may include repeat colposcopy to ensure the entire transformation zone can be visualized. If the colposcopy is satisfactory and no higher-grade lesions are identified, management may include a follow-up with repeat cytology and colposcopy at a 6 to 12-month interval. | D | C | C |
| Claude 2 (With prompt) | 41. For a patient with LSIL cytology findings and TZ3 classification in cervical cancer screening, follow-up steps may include a colposcopy examination, endocervical curettage, HPV testing, and a repeat Pap smear. Treatment options such as monitoring, further diagnostic tests, or procedures like cryotherapy, LEEP, or cone biopsy may be discussed based on the results of these tests. It is important for the patient to adhere to the recommendations of their healthcare provider and continue with regular cervical cancer screenings. | B | A | A |
| Claude 2 | With confirmed LSIL, LSIL+ cytology, and TZ3 colposcopy - follow up with colposcopy and ECC in 6 months. | B | C | C |
| Gemini Pro (With prompt) | 41. In cervical cancer screening, if a patient has a histopathological confirmation (biopsy) of LSIL cytology findings of LSIL or higher and a TZ3 classification, what follow-up steps should be conducted? Loop electrosurgical excision procedure (LEEP) or cold knife conization | C | D | D |
| Gemini Pro | Repeat colposcopy and biopsy in 6 months. | C | C | C |
| Mistral-7B-v0.2 (With prompt) | 41.In cervical cancer screening, if a patient has a histopathological confirmation (biopsy) of LSIL (low-grade squamous intraepithelial lesion) cytology findings and a TZ3 (severe dyskaryosis) classification, a repeat cytological examination in 12 months is recommended. If the repeat cytological examination is also abnormal, or if the individual has other risk factors for cervical cancer, a colposcopy with biopsy may be recommended. | C | C | C |
| Mistral-7B-v0.2 | For a patient with histopathological confirmation (biopsy) of LSIL (low-grade squamous intraepithelial lesion), cytology findings of LSIL or higher, and a TZ3 classification (severe dyskaryosis), the following steps should be conducted: - Repeat cervical cancer screening, ideally in 12 months - Close monitoring and follow-up, including extension of the transformation zone (TZ) examination at the next screening - Consider HPV genotype testing, especially for high-risk types (HPV 16, 18, etc.) as they have a higher risk of progression to cervical cancer. | D | D | D |
| Starling-LM-7B alpha (With prompt) | 41. In cervical cancer screening, if a patient has a histopathological confirmation (biopsy) of LSIL cytology findings of LSIL or higher and a TZ3 classification, the following follow-up steps should be conducted:   a. Repeat colposcopy: This should be performed 6-12 weeks after the initial colposcopy to assess the response to treatment or to evaluate any persistent or recurrent abnormalities.   b. Cervical biopsy: If the repeat colposcopy shows any persistent or recurrent abnormalities, a cervical biopsy should be performed to confirm the presence of cervical dysplasia or cancer. | C | C | C |
| Starling-LM-7B alpha | For a patient with histopathological confirmation of LSIL, cytology findings of LSIL or higher, and a TZ3 classification, the follow-up steps should include: - Colposcopy within 4-12 weeks from the date of the screening test. If the colposcopy result is normal, no additional follow-up is needed. If the colposcopy result is ASC-US, LSIL, or higher, a co-test of cytology and HPV testing should be performed within 12 months of the colposcopy. If the co-test result is ASC-US or higher with a positive HPV test, referred for treatment, otherwise, perform repeat cytology and HPV testing at 6, 12, and 24 months. | D | D | D |
| HuatuoGPT (With prompt) | 41. In cervical cancer screening, if a patient's pathologic confirmation (biopsy) reveals LSIL cytologic findings of LSIL or less and a TZ1/2 classification, what next follow-up steps should be performed? Answer: Annual cervical cytology screening should be performed. | D | D | D |
| HuatuoGPT | If a patient has low-grade squamous intraepithelial lesion (LSIL) confirmed by histopathology (biopsy), LSIL or higher cytologic findings, and a TZ3 classification, what follow-up steps should be taken? Colposcopy with cervical biopsy is recommended for patients with LSIL, LSIL or higher cytologic findings, and TZ3 classification. The colposcopic findings will help determine the presence of abnormal areas, while the cervical biopsy will provide histologic confirmation to further assess the extent of the lesion. | C | C | C |
| BioMedLM 2.7B (With prompt) | 41. In cervical cancer screening, if a patient has a histopathological confirmation (biopsy) of LSIL cytology findings of LSIL or higher and a TZ3 classification, what follow-up steps should be conducted? The patient should be referred to colposcopy. | D | D | D |
| BioMedLM 2.7B | The patient should undergo colposcopy with biopsy to confirm the diagnosis. If the biopsy result shows CIN 2/3 (cervical intraepithelial neoplasia grade 2 or 3), the patient should receive treatment immediately. If the biopsy results show CIN 1, the patient can return for repeat cytology in 6 months. If the repeat cytology reveals ASCUS (atypical squamous cells of undetermined significance)/LSIL (low-grade squamous intraepithelial lesion), the patient may be followed up by cytology every year until it becomes negative. If the repeat cytology remains positive, the patient should undergo colposcopy with biopsy. If the biopsy result shows CIN 2/3, the patient should receive treatment immediately; otherwise, the patient should return for repeat cytology at 6 months after the initial diagnosis. If the repeat cytology remains positive, the patient should undergo colposcopy with biopsy. If the biopsy result shows CIN 1, the patient should return for repeat cytology in 12 months. | B | C | B |
| Microsoft Phi2 (With prompt) | 41. If a patient has a histopathological confirmation (biopsy) of LSIL cytology findings of LSIL or higher and a TZ3 classification, the patient should undergo colposcopy with biopsy. | C | C | C |
| Microsoft Phi2 | If a patient has a histopathological (biopsy) confirmation of low-grade squamous intraepithelial lesion (LSIL), cytopathology findings of LSIL or higher, and a tri-color zone 3 classification, it's recommended that they undergo repeat Pap smear testing every 6 months for 2 years after their initial diagnosis. After that, they can continue regular screenings based on local guidelines. | D | D | D |
| QiZhenGPT (With prompt) | 41.In cervical cancer screening, if a patient has a histopathological confirmation (biopsy) of LSIL cytology findings of LSIL or higher and a TZ3 classification, what follow-up steps should be conducted? Answer: Additional testing for high-risk HPV, such as a DNA test for HPV16 and HPV18. | D | D | D |
| QiZhenGPT | In cervical screening, if a patient has histopathologic confirmation of LSIL (biopsy), cervical cytology finding of LSIL or higher, and TZ3 classification, what follow-up steps should be performed? A: If a patient has histopathologic confirmation of LSIL on cervical screening, cervical cytology finding of LSIL or higher, and TZ3 classification, the following follow-up steps should be performed: * Cervical cytology and/or colposcopy every 6-12 months * Cervical cytology and/or colposcopy every 1-2 years * Consider cervical biopsy to evaluate for cancer if cervical cytology results are consistently LSIL or higher | C | D | D |
| **42.In cervical cancer screening, if a patient has a histopathological confirmation(biopsy) of LSIL, cytology findings of LSIL or higher, and a TZ1/2 classification, what follow-up steps should be conducted?** | | | | |
| ChatGPT4.0 turbo (With prompt) | 42. Follow-up for LSIL with TZ1/2 Classification: For patients with histopathological confirmation of LSIL (or lower) and a transformation zone 1 or 2 (TZ1/2) classification, the follow-up involves repeat cytology and colposcopy at a 12-month interval. This interval allows for monitoring of the lesion to detect any progression or resolution. The more accessible visualization and sampling of TZ1/2 provide confidence in the surveillance strategy. | B | B | B |
| ChatGPT4.0 turbo | LSIL Histopathology, LSIL or Lower Cytology, TZ1/2 Classification:  - With confirmed LSIL on biopsy, LSIL or lower cytology findings, and a TZ1/2 classification (where the transformation zone is fully visible), follow-up may include repeat cytology at 6 to 12 months or HPV testing. If the repeat testing is normal, the patient may return to routine screening intervals. | B | B | B |
| Claude 2 (With prompt) | 42. For histologic LSIL with low grade changes (TZ 1-2), continued observation with co-testing at 1 and 3 years is appropriate. More frequent follow up may be considered based on patient factors. | B | B | B |
| Claude 2 | With confirmed LSIL, ≤LSIL cytology, and TZ 1/2 colposcopy - repeat co-testing at 12 months and 18 months. | D | D | D |
| Gemini Pro (With prompt) | 42. In cervical cancer screening, if a patient has a histopathological confirmation (biopsy) of LSIL cytology findings of LSIL or lower and a TZ1/2 classification, what follow-up steps should be conducted? Repeat cytology and HPV testing in 6 months | B | B | B |
| Gemini Pro | Repeat cytology and HPV testing in 12 months. | A | A | A |
| Mistral-7B-v0.2 (With prompt) | 42.In cervical cancer screening, if a patient has a histopathological confirmation (biopsy) of LSIL (low-grade squamous intraepithelial lesion) cytology findings and a TZ1/2 (mild or moderate dyskaryosis) classification, a repeat cytological examination in 12 months is recommended. If the repeat cytological examination is also negative, no further testing may be necessary, unless the individual has other risk factors for cervical cancer. Regular follow-up appointments and screening tests are recommended based on age and other risk factors. | B | B | B |
| Mistral-7B-v0.2 | For a patient with histopathological confirmation (biopsy) of LSIL, cytology findings of LSIL or lower, and a TZ1/2 classification (mild or moderate dyskaryosis or no dyskaryosis), the following steps should be conducted: - Repeat cervical cancer screening within 12 months - Consider HPV genotype testing for future screening decisions if the patient has a low-risk HPV type or ASCUS (atypical squamous cells of undetermined significance) on the next cytology test. | C | B | B |
| Starling-LM-7B alpha (With prompt) | 42. In cervical cancer screening, if a patient has a histopathological confirmation (biopsy) of LSIL cytology findings of LSIL or lower and a TZ1/2 classification, the following follow-up steps should be conducted:   a. Repeat cytology and HPV testing: This should be performed in 6-12 months to monitor for any changes in the cytological and HPV results.   b. Colposcopy with cervical biopsy: If the repeat cytology and HPV testing show persistent or recurrent abnormalities, colposcopy and cervical biopsy should be performed to further evaluate the presence of cervical dysplasia or cancer. | A | A | A |
| Starling-LM-7B alpha | For a patient with histopathological confirmation of LSIL, cytology findings of LSIL or lower, and a TZ1/2 classification, the follow-up steps should include: - No additional follow-up is needed if the colposcopy result is normal. If the colposcopy result is ASC-US, LSIL, or higher, a co-test of cytology and HPV testing should be performed within 12 months of the colposcopy. If the co-test result is ASC-US or higher with a positive HPV test, referred for treatment, otherwise, perform repeat cytology and HPV testing at 6, 12, and 24 months. | D | C | C |
| HuatuoGPT (With prompt) | 42. In cervical cancer screening, if a patient has histopathologic confirmation (biopsy) and LSIL cytology reveals a classification of LSIL or less with a TZ1/2 classification, what follow-up steps should be performed next? Ans: For patients with LSIL cytology finding LSIL or below classification with TZ1/2 classification, a second cytology is recommended after 6 months. | C | B | B |
| HuatuoGPT | If a patient is confirmed (biopsied) by histopathology for LSIL, LSIL or lower cytologic findings, and TZ1/2 classification, what follow-up steps should be taken? Surveillance is recommended for patients with LSIL, LSIL or lower cytologic findings and TZ1/2 classification. Immediate cervical biopsy is not required due to the low level of lesions. However, regular cytology and colposcopy will help to follow the progression of the lesion. If the lesion worsens or persists, further evaluation and treatment may be required. | B | C | B |
| BioMedLM 2.7B (With prompt) | 42. In cervical cancer screening, if a patient has a histopathological confirmation (biopsy) of LSIL cytology findings of LSIL or lower and a TZ1/2 classification, what follow-up steps should be conducted? The patient should be followed up with a repeat cytology test at 6 months. If the cytology is negative, the patient should be followed up with a repeat cytology test at 12 months. If the cytology is positive, colposcopy should be performed. If the colposcopy is negative, the patient should be followed up with a repeat cytology test at 6 months. If the cytology is positive, the patient should be followed up with a repeat cytology test at 12 months. If the cytology is positive, colposcopy should be performed. If the colposcopy is negative, the patient should be followed up with a repeat cytology test at 6 months. | C | C | C |
| BioMedLM 2.7B | Follow up with colposcopy at 6 months. If there are no abnormalities on colposcopic examination, repeat cytology in 12 months. If there are persistent abnormalities (ASCUS or worse), repeat colposcopy within 3--6 months. If there are persistent abnormalities, biopsy for histological evaluation. If there is CIN 2+, perform loop electrosurgical excision procedure (LEEP). | C | C | C |
| Microsoft Phi2 (With prompt) | 42. If a patient has a histopathological confirmation (biopsy) of LSIL cytology findings of LSIL or lower and a TZ1/2 classification, the patient should undergo colposcopy with biopsy. | D | D | D |
| Microsoft Phi2 | If a patient has a histopathological confirmation of LSIL, cytology findings of LSIL or lower, and a tri-color zone 1/2 classification, it's also recommended that they undergo repeat Pap smear testing every 6 months for 2 years after their initial diagnosis. Following that, they can continue regular screenings based on local guidelines. | D | B | B |
| QiZhenGPT (With prompt) | 42.In cervical cancer screening, if a patient has a histopathological confirmation (biopsy) of LSIL cytology findings of LSIL or lower and a TZ1/2 classification, what follow-up steps should be conducted? Answer: Additional testing for high-risk HPV, such as a DNA test for HPV16 and HPV18. | D | D | D |
| QiZhenGPT | In cervical screening, what follow-up steps should be performed if a patient has pathologic confirmation of LSIL, cervical cytology findings of LSIL or lower, and TZ1/2 classification? A: If a patient has pathologic confirmation of LSIL, cervical cytology findings of LSIL or lower, and TZ1/2 classification on cervical screening, the following follow-up steps should be performed: a) Cervical cytology (Pap smear) and/or cytology and histology (colposcopy) every 6-12 months. b) If cervical cytology results are persistently LSIL or lower, a LEEP biopsy should be considered to evaluate for the presence of cervical intraepithelial neoplasia (CIN). c) For persistent LSIL, regular follow-up, including cervical cytology and/or colposcopy, should be performed to monitor for lesion progression. | C | D | C |
| **43.In cervical cancer screening, if a patient has a histopathological confirmation(biopsy) of LSIL, cytology findings of ASC-H or higher, what follow-up steps should be conducted?** | | | | |
| ChatGPT4.0 turbo (With prompt) | 43. Follow-up for ASC-H or Higher on Biopsy: When a patient has a histopathological confirmation of atypical squamous cells, cannot exclude high-grade (ASC-H) or higher, the recommended follow-up involves a colposcopy with biopsy, if not already performed. If the colposcopy and initial biopsy confirm ASC-H or a higher grade lesion, management should be guided by the biopsy results. For HSIL (high-grade squamous intraepithelial lesion) findings, treatment options such as excisional procedures (e.g., LEEP or cold knife conization) may be considered depending on individual patient factors and preferences. | A | A | A |
| ChatGPT4.0 turbo | LSIL Histopathology, ASC-H or Higher Cytology:  - For a patient with LSIL on biopsy and ASC-H or higher on cytology, a colposcopy is indicated. If the colposcopy is satisfactory and no higher-grade lesions are identified, management may involve a follow-up with repeat cytology and colposcopy at a 6 to 12-month interval. If CIN2+ is identified, treatment according to guidelines for high-grade lesions may be necessary. | C | C | C |
| Claude 2 (With prompt) | 43. For histologic diagnosis of ASC-H, HSIL or more severe, guidelines recommend excisional treatment regardless of colposcopic impressions since these indicate precancer/cancer. Post-treatment surveillance will follow based on excision margin status. | B | A | A |
| Claude 2 | With confirmed LSIL and ASC-H+ cytology - perform diagnostic excisional procedure or repeat colposcopy and biopsy in 6 months. | D | C | D |
| Gemini Pro (With prompt) | 43. In cervical cancer screening, if a patient has a histopathological confirmation (biopsy) of LSIL cytology findings of ASC-H or higher, what follow-up steps should be conducted? Colposcopy with biopsy | C | C | C |
| Gemini Pro | Repeat colposcopy and biopsy in 6 months. | D | D | D |
| Mistral-7B-v0.2 (With prompt) | 43.In cervical cancer screening, if a patient has a histopathological confirmation (biopsy) of LSIL (low-grade squamous intraepithelial lesion) cytology findings of ASC-H (atypical glandular cells) or higher, a repeat cytological examination in 12 months is recommended. If the repeat cytological examination is also abnormal, or if the individual has other risk factors for cervical cancer, a colposcopy with biopsy may be recommended. | D | C | C |
| Mistral-7B-v0.2 | For a patient with histopathological confirmation (biopsy) of LSIL and cytology findings of ASC-H (atypical squamous cells cannot exclude HSIL) or higher, no matter the TZ classification: - Loop Electrosurgical Excision Procedure (LEEP) or conization should be considered to remove the lesion and reduce the risk of developing cervical cancer - Continuous follow-up every 6-12 months - Consider HPV genotype testing and intravaginal or intracervical HPV DNA testing. | C | C | C |
| Starling-LM-7B alpha (With prompt) | 43. In cervical cancer screening, if a patient has a histopathological confirmation (biopsy) of LSIL cytology findings of ASC-H or higher, the following follow-up steps should be conducted:   a. Repeat colposcopy within 4 to 6 weeks.  b. Obtain additional cervical samples for cytology and HPV testing.  c. If the colposcopy findings are still inconclusive, consider performing a loop electrosurgical excision procedure (LEEP) or conization to remove abnormal tissue.  d. If high-grade lesions are confirmed, refer the patient for further evaluation and treatment, which may include a hysterectomy or radiation therapy. | A | A | A |
| Starling-LM-7B alpha | For a patient with histopathological confirmation of LSIL, cytology findings of ASC-H or higher, the follow-up steps should include: - Referral for colposcopy within 4-12 weeks from the date of the screening test. If the colposcopy result is normal and the patient's age is equal to or greater than 25 years, no additional follow-up is needed. If the colposcopy result is ASC-US, LSIL, or higher, a co-test of cytology and HPV testing should be performed within 12 months of the colposcopy. If the co-test result is ASC-US or higher with a positive HPV test, referred for treatment, otherwise, perform repeat cytology and HPV testing at 6, 12, and 24 months. | D | C | D |
| HuatuoGPT (With prompt) | 43. What are the next follow-up steps that should be performed in a patient between the ages of 21 and 24 years who has an LSIL cytologic finding of ASC-H or higher on a pap smear performed during pregnancy and who has a histopathologic confirmation (biopsy) of having an LSIL cytologic finding? Answer: In pregnant patients, colposcopy and collection of cervical tissue for high-risk human papillomavirus (HPV) typing should be performed. | D | D | D |
| HuatuoGPT | If a patient has low-grade squamous intraepithelial lesion (LSIL) confirmed by histopathology (biopsy) and a cytologic result of ASC-H or higher, what follow-up steps should be taken? Colposcopy with cervical biopsy is recommended for patients with LSIL, ASC-H or higher cytologic findings. The colposcopy results will help to determine if there are areas of abnormality, while the cervical biopsy will provide histologic confirmation to further evaluate the extent of the lesion. In addition, consider HPV DNA testing to determine the presence of high-risk HPV infection. | C | C | C |
| BioMedLM 2.7B (With prompt) | 43. In cervical cancer screening, if a patient has a histopathological confirmation (biopsy) of LSIL cytology findings of ASC-H or higher, what follow-up steps should be conducted? The patient should be referred to colposcopy. | C | C | C |
| BioMedLM 2.7B | 1. Repeat cytology in 6 months; 2. Repeat cytology and colposcopy in 12 months; 3. Repeat cytology every year for 5 years; 4. Do not repeat cytology unless there are clinical indications (e.g., persistent high grade cytology). | D | C | D |
| Microsoft Phi2 (With prompt) | 43.In cervical cancer screening, if a patient has a histopathological confirmation (biopsy) of LSIL cytology findings of ASC-H or higher, the patient should be referred to a gynecologic oncologist for further evaluation and management. The patient should undergo a repeat colposcopy with biopsy, and if the biopsy results are consistent with high-grade squamous intraepithelial lesion (HSIL) or cervical cancer, the patient should be referred to a gynecologic oncologist for further evaluation and management. | C | C | C |
| Microsoft Phi2 | If a patient has a histopathological confirmation of LSIL, cytology findings of atypical squamous cells of undetermined significance (ASC-H), and no further diagnostic tests are required. However, they should maintain routine cervical cancer screenings as advised by healthcare professionals. | D | D | D |
| QiZhenGPT (With prompt) | 43. Follow-up steps should be conducted to evaluate the persistence or progression of the LSIL (low-gradeSIL)cythemia in the patient. This may involve re-screening the patient in 6-12 months, or more frequently if the LSILcythemia persists or worsens. | D | D | D |
| QiZhenGPT | In cervical screening, what follow-up steps should be performed if a patient has pathologic confirmation of LSIL, a cervical cytology finding of ASC-H or higher, and a TZ3 classification? A: If a patient has pathologic confirmation of LSIL, cervical cytology findings of ASC-H or higher, and TZ3 classification on cervical screening, the following follow-up steps should be performed: a) Cervical cytology (Pap smear) and/or cytology and histology (colposcopy) every 6-12 months. b) If cervical cytology results are persistently ASC-H or higher, a LEEP biopsy should be considered to evaluate for the presence of cervical intraepithelial neoplasia (CIN). c) For persistent LSIL, regular follow-up, including cervical cytology and/or colposcopy, should be performed to monitor the progression of the lesion. | D | D | D |
| **44.In cervical cancer screening, if a patient aged 21 to 24 years has a histopathological confirmation(biopsy) of LSIL, cytology findings of ASC-H or higher, what follow-up steps should be conducted?** | | | | |
| ChatGPT4.0 turbo (With prompt) | 44. Follow-up for Young Patients (21-24 Years) with ASC-H or Higher: For younger patients aged 21 to 24 years with histopathological confirmation of ASC-H or higher, a more conservative approach is generally recommended due to the higher likelihood of HPV-related changes resolving spontaneously in this age group. Management typically involves colposcopy and observation, rather than immediate excisional treatment. Repeat evaluation with colposcopy and cytology at 6 and 12 months to monitor for progression or regression is advised. | B | B | B |
| ChatGPT4.0 turbo | LSIL Histopathology, ASC-H or Higher Cytology, Aged 21-24:  - Young women aged 21-24 with LSIL on biopsy and ASC-H or higher on cytology are generally managed conservatively due to the higher likelihood of spontaneous regression. Close follow-up with repeat cytology and colposcopy at 12-month intervals may be recommended to avoid overtreatment. | B | B | B |
| Claude 2 (With prompt) | 44. For ages 21-24 with biopsy proven HSIL/ASC-H, conservative management is acceptable with co-testing follow up at 6 months and 12 months looking for regression vs persistence. Excision can be considered if lesion persists. | D | D | D |
| Claude 2 | For ages 21-24 with confirmed LSIL and ASC-H+ cytology - repeat co-testing at 12 months. If HPV positive, colposcopy. | B | C | C |
| Gemini Pro (With prompt) | 44. In cervical cancer screening, if a patient aged 21 to 24 years has a histopathological confirmation (biopsy) of LSIL cytology findings of ASC-H or higher, what follow-up steps should be conducted? Repeat cytology and HPV testing in 6 months | D | D | D |
| Gemini Pro | Repeat colposcopy and biopsy in 6 months. | D | D | D |
| Mistral-7B-v0.2 (With prompt) | 44.In cervical cancer screening for patients aged 21 to 24 years, if there is a histopathological confirmation (biopsy) of LSIL (low-grade squamous intraepithelial lesion) cytology findings of ASC-H (atypical glandular cells) or higher, a repeat cytological examination in 6 to 12 months is recommended. If the repeat cytological examination is also abnormal, or if the individual has other risk factors for cervical cancer, a colposcopy with biopsy may be recommended. | C | C | C |
| Mistral-7B-v0.2 | For a patient aged 21 to 24 years with a histopathological confirmation of LSIL, cytology findings of ASC-H or higher: - If the colposcopy results (TZ1, TZ2, or TZ3) are available:  a) TZ3: A repeat colposcopy within 12 weeks, and consider repeating the HPV test in 12 months if the initial HPV test was done more than 12 months prior.  b) TZ1/2: Repeat cervical cytology testing in 12 months. - If colposcopy results are not available, continue monitoring through annual cervical cytology. | C | C | C |
| Starling-LM-7B alpha (With prompt) | 44. In cervical cancer screening, if a patient aged 21 to 24 years has a histopathological confirmation (biopsy) of LSIL cytology findings of ASC-H or higher, the following follow-up steps should be conducted:   a. Repeat colposcopy within 4 to 6 weeks.  b. Obtain additional cervical samples for cytology and HPV testing.  c. If the colposcopy findings are still inconclusive, consider performing a loop electrosurgical excision procedure (LEEP) or conization to remove abnormal tissue.  d. If high-grade lesions are confirmed, refer the patient for further evaluation and treatment, which may include a hysterectomy or radiation therapy. | B | B | B |
| Starling-LM-7B alpha | For a patient aged 21 to 24 years with histopathological confirmation of LSIL, cytology findings of ASC-H or higher, the follow-up steps should include: - Referral for colposcopy within 4-12 weeks from the date of the screening test. If the colposcopy result is normal, no additional follow-up is needed. If the colposcopy result is ASC-US, LSIL, or higher, a co-test of cytology and HPV testing should be performed within 12 months of the colposcopy. If the co-test result is ASC-US or higher with a positive HPV test, referred for treatment, otherwise, perform repeat cytology and HPV testing at 6, 12, and 24 months. | D | D | D |
| HuatuoGPT (With prompt) | 44. In a patient 21 to 24 years of age who had a Pap smear during pregnancy that revealed ASC-H or higher LSIL cytologic findings, what are the next next steps that should be performed if the histopathologic confirmation (biopsy) is that there are LSIL cytologic findings and the colposcopy results are classified as TZ3? Answer: In this case, colposcopy and collection of cervical tissue for high-risk human papillomavirus (HPV) typing should be performed. | D | D | D |
| HuatuoGPT | If a patient aged 21 to 24 years has low-grade squamous intraepithelial lesion (LSIL) confirmed by histopathology (biopsy) and a cytologic result of ASC-H or higher, what are the next steps that should be taken? In younger patients, colposcopy with cervical biopsy is recommended if the cytology result is ASC-H or higher. This is because lesions in younger patients may have a different natural course and there is a risk of developing higher grade lesions. Obtaining histologic confirmation by cervical biopsy will help determine the extent of the lesion and decide whether treatment is necessary. | B | B | B |
| BioMedLM 2.7B (With prompt) | 44. In cervical cancer screening, if a patient aged 21 to 24 years has a histopathological confirmation (biopsy) of LSIL cytology findings of ASC-H or higher, what follow-up steps should be conducted? The patient should be referred to a gynecologist for colposcopy and biopsy. | B | B | B |
| BioMedLM 2.7B | 1. Repeat cytology in 6 months;2. Repeat cytology and HPV test at 12 months;3. Repeat cytology every 3 years for women with negative results on both tests (HPV DNA testing can be performed after 1 year);4. Repeat cytology and HPV test at 18--24 months;5. Repeat cytology and HPV test annually thereafter until 5 consecutive negative results are obtained. | D | D | D |
| Microsoft Phi2 (With prompt) | 44.In cervical cancer screening, if a patient aged 21 to 24 years has a histopathological confirmation (biopsy) of LSIL cytology findings of ASC-H or higher, the patient should be referred to a gynecologic oncologist for further evaluation and management. The patient should undergo a repeat colposcopy with biopsy, and if the biopsy results are consistent with high-grade squamous intraepithelial lesion (HSIL) or cervical cancer, the patient should be referred to a gynecologic oncologist for further evaluation and management. | D | D | D |
| Microsoft Phi2 | For patients aged 21 to 24 who have a histopathological confirmation of LSIL, cytology findings of ASC-H or higher, it's important to conduct a thorough evaluation. They may require additional tests such as HPV DNA testing or colposcopy to determine the appropriate course of action. The specific recommendations will depend on individual circumstances and the guidance provided by healthcare professionals. | B | C | C |
| QiZhenGPT (With prompt) | 44. Follow-up steps should be conducted to evaluate the patient for any potential complications or risks associated with the procedure. The patient should also be referred to a specialist for further evaluation and management. | D | D | D |
| QiZhenGPT | In cervical cancer screening, if a patient aged 21 to 24 years has pathologic confirmation of LSIL (biopsy) and cervical cytology reveals ASC-H or higher, what follow-up steps should be performed? A. If a patient 21 to 24 years of age has pathologic confirmation of LSIL (biopsy) and cervical cytology reveals ASC-H or higher at cervical cancer screening, the following follow-up steps should be performed: cervical cytology and HPV testing first, cervical biopsy every 6 to 12 months, and periodic physical exams. | C | D | D |
| **45.In cervical cancer screening, if a patient aged 21 to 24 years has a histopathological confirmation(biopsy) of LSIL, cytology findings of ASC-H or higher, what follow-up steps should be conducted?** | | | | |
| ChatGPT4.0 turbo (With prompt) | 45. Follow-up for Young Patients (21-24 Years) with ASC-H or Higher and TZ3: In patients aged 21 to 24 years with ASC-H or higher findings and a transformation zone 3 classification, careful follow-up is crucial due to the difficulty in fully visualizing and sampling this area. Colposcopy with endocervical assessment and repeat cytology at 6 and 12 months is recommended. The goal is to ensure comprehensive evaluation while minimizing invasive procedures, given the potential for spontaneous regression in this age group. | D | D | D |
| ChatGPT4.0 turbo | LSIL Histopathology, ASC-H or Higher Cytology, TZ3 Classification, Aged 21-24:  - For this age group, even with a TZ3 classification, conservative management with repeat colposcopy and cytology at 12-month intervals is often recommended, given the potential for spontaneous regression and the desire to avoid aggressive procedures that may affect future fertility. | D | C | D |
| Claude 2 (With prompt) | 45. If TZ 3 colposcopy in this age group, indicating high grade disease, guidelines allow deferring treatment but with very close 6 month co-test follow up and treatment if repeat HSIL cytology or persistent colposcopic lesion. | D | D | D |
| Claude 2 | For ages 21-24, with confirmed LSIL, ASC-H+ cytology, and TZ3 colposcopy - follow up colposcopy and biopsy at 12 months. | B | C | B |
| Gemini Pro (With prompt) | 45. In cervical cancer screening, if a patient aged 21 to 24 years has a histopathological confirmation (biopsy) of LSIL cytology findings of ASC-H or higher and a TZ3 classification for colposcopy, what follow-up steps should be conducted? Loop electrosurgical excision procedure (LEEP) or cold knife conization | A | A | A |
| Gemini Pro | Repeat colposcopy and biopsy in 6 months. | D | D | D |
| Mistral-7B-v0.2 (With prompt) | 45.In cervical cancer screening for patients aged 21 to 24 years, if there is a histopathological confirmation (biopsy) of LSIL (low-grade squamous intraepithelial lesion) cytology findings of ASC-H (atypical glandular cells) or higher and a TZ3 (severe dyskaryosis) classification for colposcopy, a repeat cytological examination in 6 to 12 months is recommended. If the repeat cytological examination is also abnormal, or if the individual has other risk factors for cervical cancer, a loop electrosurgical excision procedure (LEEP) or cold knife conization may be recommended. | B | B | B |
| Mistral-7B-v0.2 | For a patient aged 21 to 24 years with a histopathological confirmation (biopsy) of LSIL, cytology findings of ASC-H or higher, and a TZ3 classification: - Referral to a specialist for further evaluation, including LEEP or conization to remove the lesion - Continuous follow-up every 6-12 months after treatment - Consider HPV genotype testing and intravaginal or intracervical HPV DNA testing before treatment for risk assessment. | C | C | C |
| Starling-LM-7B alpha (With prompt) | 45. In cervical cancer screening, if a patient aged 21 to 24 years has a histopathological confirmation (biopsy) of LSIL cytology findings of ASC-H or higher and a TZ3 classification for colposcopy, the following follow-up steps should be conducted:   a. Perform a loop electrosurgical excision procedure (LEEP) or conization to remove the abnormal tissue.  b. Schedule a follow-up colposcopy within 4 to 6 weeks to assess the effectiveness of the procedure.  c. If the colposcopy findings are still inconclusive, consider performing a repeat LEEP or conization.  d. If high-grade lesions are confirmed, refer the patient for further evaluation and treatment, which may include a hysterectomy or radiation therapy. | B | B | B |
| Starling-LM-7B alpha | For a patient aged 21 to 24 years with a histopathological confirmation (biopsy) of LSIL and cytology findings of ASC-H or higher, the follow-up steps should include:  - Repeat cytology screening in 6 to 12 months.  - If repeat cytology shows no abnormal findings, res screen in 2 years.  - If on repeat cytology, abnormal findings persist or worsen, refer to a colposcopist for colposcopy.  - For colposcopy results with TZ3 classification, direct the patient to a gynecologist or a surgeon for excisional procedure like loop electrosurgical excision procedure (LEEP) or cold knife cone biopsy (CKCB). In cases where LEEP or CKCB is contraindicated, provide treatment alternatives like laser ablation, cryotherapy, or various forms of audacious to be carried out as per the TZ3 classification.   - If colposcopy results show no abnormalities deemed concerning, schedule a follow-up colposcopy in 6 to 12 months. If colposcopy results reveal no abnormalities, see patient again after 3 years. | C | C | C |
| HuatuoGPT (With prompt) | 45. In a patient 21 to 24 years of age who had a Pap smear during pregnancy that revealed ASC-H or higher LSIL cytologic findings, what subsequent steps should be performed if there is histopathologic confirmation (biopsy) of an LSIL cytologic finding and colposcopy results in a TZ1/2 classification? Ans: In this case, an annual pap smear should be performed. | D | D | D |
| HuatuoGPT | If a patient aged 21 to 24 years has low-grade squamous intraepithelial lesion (LSIL) confirmed by histopathology (biopsy), has a cytologic result of ASC-H or higher, and undergoes a colposcopy with a TZ3 classification, what are the next steps that should be taken? In younger patients with a cytologic result of ASC-H or higher and a TZ3-classified colposcopy, colposcopy with a cervical biopsy is recommended. This is because lesions in younger patients may have a different natural course and there is a risk of progression to higher grade lesions. Obtaining histologic confirmation by cervical biopsy will help determine the extent of the lesion and decide whether treatment is necessary. | C | B | C |
| BioMedLM 2.7B (With prompt) | 45. In cervical cancer screening, if a patient aged 21 to 24 years has a histopathological confirmation (biopsy) of LSIL cytology findings of ASC-H or higher and a TZ3 classification for colposcopy, what follow-up steps should be conducted? The patient should be followed up with colposcopy，gynecologist and biopsy. | D | D | D |
| BioMedLM 2.7B | Follow up with cytology every 6 months until negative results are obtained; if the result remains positive, perform colposcopic biopsy. | C | D | D |
| Microsoft Phi2 (With prompt) | 45.In cervical cancer screening, if a patient aged 21 to 24 years has a histopathological confirmation (biopsy) of LSIL cytology findings of ASC-H or higher and a TZ3 classification for colposcopy, the patient should be referred to a gynecologic oncologist for further evaluation and management. The patient should undergo a repeat colposcopy with biopsy, and if the biopsy results are consistent with high-grade squamous intraepithelial lesion (HSIL) or cervical cancer, the patient should be referred to a gynecologic oncologist for further evaluation and management. | D | D | D |
| Microsoft Phi2 | If a patient aged 21 to 24 has a histopathological confirmation of LSIL, cytology findings of ASC-H or higher, and a tri-color zone 3 classification for colposcopy, it's crucial to schedule a colposcopic examination. This procedure allows for a detailed visual inspection of the cervix under magnification, helping to identify any abnormalities that may warrant further investigation or treatment. | D | D | D |
| QiZhenGPT (With prompt) | 45. Follow-up steps should be conducted to evaluate the patient for any potential complications or risks associated with the LSILcythemia. This may include performing a colposcopy or other imaging studies to assess the site of thecythemia and to identify any potential complications such as bleeding or infection. | D | D | D |
| QiZhenGPT | In cervical screening, if a patient between 21 and 24 years of age has pathologically confirmed (biopsy) findings of LSIL, cytologic findings of ASC-H or higher, and a TZ3 classification by colposcopy, then the following follow-up steps should be performed: - Cytology and colposcopy should be performed regularly in order to monitor the progression of the lesion. - If cytology results are consistently ASC-H or higher, consider performing a LEEP biopsy to further evaluate the extent of the lesion. - For TZ3 classification, more frequent follow-up may be necessary to allow timely detection of lesion progression. | D | D | D |
| **46.In cervical cancer screening, if a patient aged 21 to 24 years has a histopathological confirmation(biopsy) of LSIL, cytology findings of ASC-H or higher, what follow-up steps should be conducted?** | | | | |
| ChatGPT4.0 turbo (With prompt) | 46. Follow-up for Young Patients (21-24 Years) with ASC-H or Higher and TZ1/2: For this demographic with ASC-H or higher findings and a transformation zone 1 or 2 classification, follow-up includes colposcopy with a repeat evaluation at 6 and 12 months. This approach allows for effective monitoring of the cervical changes with a good visualization of the transformation zone, helping to detect any progression early while still considering the potential for regression. | A | A | A |
| ChatGPT4.0 turbo | LSIL Histopathology, ASC-H or Higher Cytology, TZ1/2 Classification, Aged 21-24:  - Similar to scenario 4, conservative management with repeat cytology and colposcopy at 12-month intervals is usually recommended. The visibility of the TZ1/2 transformation zone can provide reassurance that the lesion has been adequately assessed. | A | A | A |
| Claude 2 (With prompt) | 46. Conservative management with co-testing at 6 and 12 months is appropriate for TZ 1-2 colposcopy in 21-24 year olds with ASC-H/HSIL biopsy. Treatment can be done if repeat cytology is HSIL or the lesion persists. | A | A | A |
| Claude 2 | For ages 21-24, with confirmed LSIL, ASC-H+ cytology, and TZ 1/2 colposcopy - repeat co-testing at 12 and 18 months. | B | B | B |
| Gemini Pro (With prompt) | 46. In cervical cancer screening, if a patient aged 21 to 24 years has a histopathological confirmation (biopsy) of LSIL cytology findings of ASC-H or higher and a TZ1/2 classification for colposcopy, what follow-up steps should be conducted? Repeat cytology and HPV testing in 6 months | A | A | A |
| Gemini Pro | Repeat cytology and HPV testing in 6 months. | A | A | A |
| Mistral-7B-v0.2 (With prompt) | 46.In cervical cancer screening for patients aged 21 to 24 years, if there is a histopathological confirmation (biopsy) of LSIL (low-grade squamous intraepithelial lesion) cytology findings of ASC-H (atypical glandular cells) or higher and a TZ1/2 (mild or moderate dyskaryosis) classification for colposcopy, a repeat cytological examination in 12 months is recommended. If the repeat cytological examination is also negative, no further testing may be necessary, unless the individual has other risk factors for cervical cancer. | A | A | A |
| Mistral-7B-v0.2 | For a patient aged 21 to 24 years with a histopathological confirmation (biopsy) of LSIL, cytology findings of ASC-H or higher, and a TZ1/2 classification: - Repeat cervical cancer screening (colposcopy, cytology) by a specialist - Consider a more intense follow-up schedule (every 6 months) based on the results of the specialist's evaluation - Consider HPV genotype testing and intravaginal or intracervical HPV DNA testing. | C | C | C |
| Starling-LM-7B alpha (With prompt) | 46. In cervical cancer screening, if a patient aged 21 to 24 years has a histopathological confirmation (biopsy) of LSIL cytology findings of ASC-H or higher and a TZ1/2 classification for colposcopy, the following follow-up steps should be conducted:   a. Repeat colposcopy within 4 to 6 weeks.  b. Obtain additional cervical samples for cytology and HPV testing.  c. If the colposcopy findings are still inconclusive, consider performing a loop electrosurgical excision procedure (LEEP) or conization to remove abnormal tissue.  d. If high-grade lesions are confirmed, refer the patient for further evaluation and treatment, which may include a hysterectomy or radiation therapy. | D | D | D |
| Starling-LM-7B alpha | For a patient aged 21 to 24 years with a histopathological confirmation (biopsy) of LSIL, cytology findings of ASC-H or higher, and a TZ3 classification for colposcopy, the follow-up steps should be as follows:  - Direct the patient to a gynecologist or a surgeon for excisional procedure like loop electrosurgical excision procedure (LEEP) or cold knife cone biopsy (CKCB). In cases where LEEP or CKCB is contraindicated, provide treatment alternatives like laser ablation, cryotherapy, or various forms of audacious to be carried out as per the TZ3 classification.  - Schedule a follow-up appointment in 6 to 12 months to ensure that there are no complications after the treatment. | D | D | D |
| HuatuoGPT (With prompt) | 46. If a patient 21 to 24 years of age who underwent a pap smear during pregnancy and was found to have an LSIL cytologic finding of ASC-H or higher is histopathologically confirmed (biopsy) to have an LSIL cytologic finding and the colposcopy result is negative, then what next follow-up steps should be performed? Ans: In this case, an annual pap smear should be performed. | C | C | C |
| HuatuoGPT | If a patient aged 21 to 24 years has low-grade squamous intraepithelial lesion (LSIL) confirmed by histopathology (biopsy), has a cytologic result of ASC-H or higher, and undergoes colposcopy with a TZ1/2 classification, what are the next steps that should be taken? In younger patients with a cytologic result of ASC-H or higher and a TZ1/2 classified colposcopy, colposcopy with a cervical biopsy is recommended. This is because lesions in younger patients may have a different natural course and there is a risk of progression to higher grade lesions. Obtaining histologic confirmation by cervical biopsy will help determine the extent of the lesion and decide whether treatment is necessary. | C | D | D |
| BioMedLM 2.7B (With prompt) | 46. In cervical cancer screening, if a patient aged 21 to 24 years has a histopathological confirmation (biopsy) of LSIL cytology findings of ASC-H or higher and a TZ1/2 classification for colposcopy, what follow-up steps should be conducted? The patient should be followed up with colposcopy and biopsy. | C | C | C |
| BioMedLM 2.7B | Follow up with cytology at 6 months after treatment. If there are no abnormalities on cytology, repeat cytology in 1 year. If there are persistent abnormalities (ASCUS+), perform colposcopic biopsy. If the biopsy result shows CIN 2+, perform LEEP. If the biopsy result shows CIN 3+ or invasive carcinoma, perform radical hysterectomy. | C | C | C |
| Microsoft Phi2 (With prompt) | 46.In cervical cancer screening, if a patient aged 21 to 24 years has a histopathological confirmation (biopsy) of LSIL cytology findings of ASC-H or higher and a TZ1/2 classification for colposcopy, the patient should be referred to a gynecologic oncologist for further evaluation and management. The patient should undergo a colposcopy to assess the extent of the abnormal cells and determine the need for additional treatment, such as cryotherapy, LEEP, or conization. The patient should also be counseled on the risk of developing cervical cancer and the importance of regular follow-up screenings. | C | C | C |
| Microsoft Phi2 | Similarly, if a patient aged 21 to 24 has a histopathological confirmation of LSIL, cytology findings of ASC-H or higher, and a tri-color zone 1/2 classification for colposcopy, scheduling a colposcopic examination becomes necessary. Colposcopy enables a comprehensive assessment of the cervix, aiding in determining whether any interventions are needed. | D | C | D |
| QiZhenGPT (With prompt) | 46. Follow-up steps should be conducted to evaluate the patient for any potential complications or risks associated with the procedure. The patient should also be referred to a specialist for further evaluation and management. | D | D | D |
| QiZhenGPT | In cervical screening, if a patient 21 to 24 years of age has pathologically confirmed (biopsy) findings of LSIL, cytologic findings of ASC-H or higher, and a TZ1/2 classification on colposcopy, then the following follow-up steps should be performed: - Cytology and colposcopy should be performed at regular intervals in order to monitor the progression of the lesion. - If cytology results are consistently ASC-H or higher, consider a LEEP biopsy to further evaluate the extent of the lesion. - For TZ1/2 classification, more frequent follow-up may be necessary to allow timely detection of lesion progression. | C | C | C |
| **47.In cervical cancer screening, if a pregnant patient has a histopathological confirmation(biopsy) of LSIL, cytology findings of ASC-H or higher, what follow-up steps should be conducted?** | | | | |
| ChatGPT4.0 turbo (With prompt) | 47. Follow-up for Pregnant Patients with ASC-H or Higher: In pregnant patients with histopathological confirmation of ASC-H or higher, management should be conservative initially, focusing on observation and delaying any potential excisional procedures until postpartum, unless invasive cancer is suspected. Colposcopy should be performed to assess the lesion, but biopsy during pregnancy is typically reserved for cases where invasive cancer cannot be ruled out. Postpartum follow-up should include repeat colposcopy and cytology to determine any changes in the cervical status and to plan further | A | A | A |
| ChatGPT4.0 turbo | Pregnant Patient, LSIL Histopathology, ASC-H or Higher Cytology:  - Pregnancy alters the management of cervical dysplasia due to concerns for both the mother and the fetus. Colposcopy can be safely performed during pregnancy, but biopsy is typically avoided unless invasive cancer is suspected. Follow-up is usually deferred until at least 6 weeks postpartum, as many cervical changes can regress after delivery. | B | C | B |
| Claude 2 (With prompt) | 47. For pregnant patients, colposcopy with biopsy is recommended for ASC-H or greater cytological abnormalities but excision is deferred until after delivery. Postpartum co-testing is needed with management based on subsequent results and colposcopy findings. | C | C | C |
| Claude 2 | For pregnant patients with confirmed LSIL and ASC-H+ cytology - colposcopy, with biopsies deferred until >12 weeks postpartum. | B | B | B |
| Gemini Pro (With prompt) | 47. In cervical cancer screening, if a pregnant patient has a histopathological confirmation (biopsy) of LSIL cytology findings of ASC-H or higher, what follow-up steps should be conducted? Colposcopy with biopsy after delivery | C | C | C |
| Gemini Pro | Repeat colposcopy and biopsy after delivery. | B | B | B |
| Mistral-7B-v0.2 (With prompt) | 47.In cervical cancer screening for pregnant patients, if there is a histopathological confirmation (biopsy) of LSIL (low-grade squamous intraepithelial lesion) cytology findings of ASC-H (atypical glandular cells) or higher, a repeat cytological examination in 12 months is recommended after delivery. If the repeat cytological examination is also abnormal, or if the individual has other risk factors for cervical cancer, a colposcopy with biopsy may be recommended. However, the decision to perform a colposcopy during pregnancy should be made on a case-by-case basis, taking into consideration the risks and benefits for both the mother and the fetus. | D | D | D |
| Mistral-7B-v0.2 | 47. For a pregnant patient with a histopathological confirmation of LSIL and cytology findings of ASC-H or higher, no matter the TZ classification: - Consult a specialist with experience in managing cervical lesions during pregnancy - Consider waiting for the delivery before performing any invasive diagnostic procedures - Follow the standard postpartum follow-up guidelines for cervical cancer screening - For high-risk HPV types (16 and 18), the specialist might consider HPV genotype testing and close surveillance during pregnancy and shortly after delivery. | C | C | C |
| Starling-LM-7B alpha (With prompt) | 47. In cervical cancer screening, if a pregnant patient has a histopathological confirmation (biopsy) of LSIL cytology findings of ASC-H or higher, the following follow-up steps should be conducted:   a. Repeat colposcopy within 4 to 6 weeks.  b. Obtain additional cervical samples for cytology and HPV testing.  c. If the colposcopy findings are still inconclusive, consider performing a loop electrosurgical excision procedure (LEEP) or conization to remove abnormal tissue, but only after discussing the risks and benefits with the patient and obtaining informed consent.  d. If high-grade lesions are confirmed, refer the patient for further evaluation and treatment, which may include a hysterectomy or radiation therapy, but only after discussing the risks and benefits with the patient and obtaining informed consent. | D | D | D |
| Starling-LM-7B alpha | For a pregnant patient with a histopathological confirmation of LSIL and cytology findings of ASC-H or higher, follow-up steps should include:   - Referral to a gynecologic oncologist or colposcopist experienced in managing cervical neoplasia during pregnancy.  - Close clinical monitoring, including regular pelvic exams and ultrasound evaluations, to assess for progression or complications.  - Consideration of treatment options, such as loop electrosurgical excision procedure (LEEP) or cold knife cone biopsy, if the pregnancy is not in the first trimester and after a thorough risk-benefit assessment.  - If treatment is warranted, postpone until after delivery, if possible, and follow up closely. | B | C | B |
| HuatuoGPT (With prompt) | 47. If a patient 21 to 24 years of age who had a pap smear during pregnancy reveals an LSIL cytologic finding of ASC-H or higher, and if the histopathologic confirmation (biopsy) is that there is an LSIL cytologic finding and the colposcopy is negative, then what next follow-up steps should be performed? Ans: In this case, an annual pap smear should be performed. | D | D | D |
| HuatuoGPT | If a pregnant woman has low-grade squamous intraepithelial lesion (LSIL) confirmed by histopathology (biopsy) and a cytologic result of ASC-H or higher, what are the next steps that should be taken? For pregnant women, if the cytology result is ASC-H or higher and LSIL is present, it is recommended to discuss this with your obstetrician. Depending on the circumstances, monitoring during pregnancy and treatment after delivery may be recommended. | B | B | B |
| BioMedLM 2.7B (With prompt) | 47. In cervical cancer screening, if a pregnant patient has a histopathological confirmation (biopsy) of LSIL cytology findings of ASC-H or higher, what follow-up steps should be conducted? The patient should be followed up with colposcopy and biopsy. | D | D | D |
| BioMedLM 2.7B | Follow up with colposcopy and biopsy if necessary. | C | C | C |
| Microsoft Phi2 (With prompt) | 47.In cervical cancer screening, if a pregnant patient has a histopathological confirmation (biopsy) of LSIL cytology findings of ASC-H or higher, the patient should be referred to a gynecologic oncologist for further evaluation and management. The patient should undergo a colposcopy to assess the extent of the abnormal cells and determine the need for additional treatment, such as cryotherapy, LEEP, or conization. The patient should also be counseled on the risk of developing cervical cancer and the importance of regular follow-up screenings. The patient should be informed that the treatment options available during pregnancy may be different from those available to non-pregnant patients, and that the risks and benefits of each treatment option should be carefully considered. The patient should also be informed that the treatment options available during pregnancy may be different from those available to non-pregnant patients, and that the risks and benefits of each treatment option should be carefully considered. | C | C | C |
| Microsoft Phi2 | When it comes to pregnant patients who have a histopathological confirmation of LSIL, cytology findings of ASC-H or higher, there might not be immediate implications for pregnancy outcomes unless other complications arise. Regular prenatal care and monitoring would typically suffice. If any concerns arise during pregnancy, healthcare providers would evaluate them individually.. | A | B | B |
| QiZhenGPT (With prompt) | 47. In cervical cancer screening, if a pregnant patient has a histopathological confirmation (biopsy) of LSIL cytology findings of ASC-H or higher, the next step would be to perform a further colposcopy to evaluate the results and to determine the appropriate treatment plan. | D | D | D |
| QiZhenGPT | in cervical screening, if a pregnant woman with pathologically confirmed (biopsy) findings of LSIL has cytologic findings of ASC-H or higher, then the following follow-up steps should be performed: - Cytology and colposcopy should be performed regularly in order to monitor the progression of the lesion. - If cytology results are consistently ASC-H or higher, consider a LEEP biopsy to further evaluate the extent of the lesion. - More frequent follow-up visits may be necessary depending on the health status of the pregnant woman and the advice of her physician. | D | D | D |
| **48.In cervical cancer screening, if a patient has a histopathological confirmation(biopsy) of HSIL, and a TZ1/2 classification, what follow-up steps should be conducted?** | | | | |
| ChatGPT4.0 turbo (With prompt) | 48. Follow-up for HSIL with TZ1/2 Classification: For patients with a histopathological confirmation of high-grade squamous intraepithelial lesion (HSIL) and a transformation zone 1 or 2 classification, an excisional procedure such as loop electrosurgical excision procedure (LEEP) or cold knife conization is recommended. This is due to the high risk of progression to or actual presence of cervical cancer associated with HSIL. The goal is to remove the lesion entirely and to ensure clear margins, while also allowing for further histopathological evaluation. | A | A | A |
| ChatGPT4.0 turbo | HSIL Histopathology, TZ1/2 Classification:  - Patients with a biopsy-confirmed HSIL and a fully visible transformation zone (TZ1/2) typically require excisional treatment, such as a loop electrosurgical excision procedure (LEEP) or cold knife conization, due to the high risk of progression to invasive cancer. | B | B | B |
| Claude 2 (With prompt) | 48. For histologic HSIL diagnosis with low grade TZ1/TZ2 colposcopic impressions, guidelines still recommend excisional treatment due to the confirmed precancerous lesion on biopsy. Post-treatment surveillance will follow based on excision margin status. | C | C | C |
| Claude 2 | With confirmed HSIL and TZ1/2 colposcopy - perform diagnostic excisional procedure. | B | B | B |
| Gemini Pro (With prompt) | 48. In cervical cancer screening, if a patient has a histopathological confirmation (biopsy) of HSIL and a TZ1/2 classification, what follow-up steps should be conducted? Loop electrosurgical excision procedure (LEEP) or cold knife conization | B | B | B |
| Gemini Pro | Conization or loop electrosurgical excision procedure (LEEP). | D | C | C |
| Mistral-7B-v0.2 (With prompt) | 48.In cervical cancer screening, if a patient has a histopathological confirmation (biopsy) of HSIL (high-grade squamous intraepithelial lesion) and a TZ1/2 (mild or moderate dyskaryosis) classification, a repeat cytological examination in 12 months is recommended. If the repeat cytological examination is also negative, no further testing may be necessary, unless the individual has other risk factors for cervical cancer. | D | D | D |
| Mistral-7B-v0.2 | For a patient with a histopathological confirmation of HSIL (High-Grade Squamous Intraepithelial Lesion) and a TZ1/2 classification, the following steps should be conducted:  a. Repeat colposcopy and cytology within 12 months for confirmation of complete resolution of HSIL.  b. HPV testing at 12 months and annually if persistent HPV infection is detected. | D | D | D |
| Starling-LM-7B alpha (With prompt) | 48. In cervical cancer screening, if a patient has a histopathological confirmation (biopsy) of HSIL and a TZ1/2 classification, the following follow-up steps should be conducted:  a. Repeat colposcopy in 3-6 months to assess the persistence or regression of the lesion. b. If the lesion persists or progresses, consider performing a loop electrosurgical excision procedure (LEEP) or cold knife conization (CKC) to remove the affected tissue. c. If the lesion regresses, consider repeating the colposcopy in 6-12 months to monitor for recurrence. d. If the patient has a history of high-grade lesions or cervical intraepithelial neoplasia (CIN) 3, consider referring her for a hysterectomy or other surgical intervention. | B | B | B |
| Starling-LM-7B alpha | In cervical cancer screening, if a patient has a histopathological confirmation (biopsy) of HSIL and a TZ1/2 classification, a colposcopy should be performed as a follow-up step. If the colposcopy findings are negative, no further treatment is needed. If the colposcopy findings are positive, the patient may need more diagnostic tests such as endocervical curettage (ECC), and the treatment options will vary depending on the results of these tests, which may include observation, loop electrical excision procedure (LEEP), or cryotherapy. | C | C | C |
| HuatuoGPT (With prompt) | 48. If there is a pathologically confirmed biopsy (e.g., colposcopy) that shows the patient has HSIL with a TZ1/2 grade result, what additional steps should be performed next? Ans: Periodic pap smear monitoring should be performed. | D | D | D |
| HuatuoGPT | If a patient has high-grade squamous intraepithelial lesion (HSIL) confirmed by histopathology (biopsy) and a cervical smear with TZ1/2 classification is performed, the following follow-up steps should be performed: a. Perform a colposcopy to further assess the extent of the cervical lesion. b. Depending on the results of the colposcopy, a cervical conization (Cervical Conization) may be necessary to obtain a more accurate diagnosis and treatment. c. Perform cervical smears and colposcopies on a regular basis to monitor the progression or disappearance of the lesion. d. Consider HPV vaccination to prevent future HPV infections. | D | C | D |
| BioMedLM 2.7B (With prompt) | 48. In cervical cancer screening, if a patient has a histopathological confirmation (biopsy) of HSIL and a TZ1/2 classification, what follow-up steps should be conducted? The patient should be referred to colposcopy. | D | D | D |
| BioMedLM 2.7B | Follow up with colposcopy and biopsy at 6 months after treatment; if there are no residual lesions or CIN 1, then repeat cytology in 12 months. If there are persistent high-grade lesions (CIN 2+), then perform LEEP. | D | D | D |
| Microsoft Phi2 (With prompt) | 48. If a patient has a histopathological confirmation (biopsy) of HSIL and a TZ1/2 classification, the patient should be referred for colposcopy. If the colposcopy is normal, the patient should be re-screened in 3 years. If the colposcopy is abnormal, the patient should be referred for a cone biopsy. | D | D | D |
| Microsoft Phi2 | If a patient has a histopathological confirmation (biopsy) of high-grade squamous intraepithelial lesion (HSIL), which is classified as Tz1/2 in cervical cancer screening, then further examination and treatment are required. In general, patients who have received negative biopsies will undergo repeat cytology every 3 months until they reach 5 consecutive normal results; if there is any abnormality detected at this time point, it may need to be repeated immediately; If the result is abnormal again after one year, a repeat biopsy can be performed. After five consecutive normal results, annual testing is sufficient. | D | C | C |
| QiZhenGPT (With prompt) | 48. In cervical cancer screening, if a patient has a histopathological confirmation (biopsy) of HSIL and a TZ1/2 classification, the following follow-up steps should be conducted: a. Follow-uposcopy: This involves performing a colposcopy or a standardoscopyoscopy on the patient to visualize the margins of the biopsy and to identify any potential cancer cells that may have been missed during the biopsy.  b. Follow-up DNA testing: If the histopathological confirmation is TZ1/2, the patient may need to undergo follow-up DNA testing to confirm the diagnosis and to determine the risk of developing cervical cancer in the future.  c. Follow-up treatment: If the patient is at high risk of developing cervical cancer, additional treatment may be recommended, such as radiation therapy or chemotherapy.  d. Follow-up care: The patient should continue to receive follow-up care, including regular check-ups with a gynecologist and any additional testing that may be needed to monitor the patient's health. | D | D | D |
| QiZhenGPT | If the patient has pathologic confirmation of HSIL on cervical screening (biopsy) and is categorized as TZ1/2, then the following follow-up steps should be performed: * Perform cytologic follow-up, including cervical cytology (pap smear) every 3-6 months * Perform colposcopy, every 6-12 months * Consider colposcopy every 12-18 months if two consecutive cervical cytology tests are normal | D | D | D |
| **49.In cervical cancer screening, if a patient has a histopathological confirmation(biopsy) of HSIL, and a TZ3 classification, what follow-up steps should be conducted?** | | | | |
| ChatGPT4.0 turbo (With prompt) | 49. Follow-up for HSIL with TZ3 Classification: In cases where HSIL is confirmed and the transformation zone is classified as TZ3, a careful and thorough colposcopy should be conducted, ideally with endocervical curettage to assess any extension into the endocervical canal that might be less accessible. If the colposcopy is satisfactory and the lesion is fully visualized, an excisional procedure such as LEEP or cold knife conization remains the treatment of choice to manage the lesion effectively and to prevent progression. | B | B | B |
| ChatGPT4.0 turbo | HSIL Histopathology, TZ3 Classification:  - In cases where the entire transformation zone (TZ3) is not visible, and HSIL is confirmed on biopsy, management often includes excisional treatment to ensure that the lesion is fully evaluated and treated. | C | B | B |
| Claude 2 (With prompt) | 49. Similarly, HSIL histology with high grade TZ3 colposcopy warrants excisional treatment - either LEEP or cold knife conization due to high risk of occult cancer. Follow up per post-excision recommendations based on margin status. | A | A | A |
| Claude 2 | With confirmed HSIL and TZ3 colposcopy - perform diagnostic excisional procedure. | B | A | B |
| Gemini Pro (With prompt) | 49. In cervical cancer screening, if a patient has a histopathological confirmation (biopsy) of HSIL and a TZ3 classification, what follow-up steps should be conducted? Radical hysterectomy or chemoradiation | C | C | C |
| Gemini Pro | Radical hysterectomy or chemoradiation. | D | C | C |
| Mistral-7B-v0.2 (With prompt) | 49.In cervical cancer screening, if a patient has a histopathological confirmation (biopsy) of HSIL (high-grade squamous intraepithelial lesion) and a TZ3 (severe dyskaryosis) classification, a loop electrosurgical excision procedure (LEEP) or cold knife conization may be recommended to remove the abnormal tissue. A repeat cytological examination in 12 months is also recommended after the procedure to ensure that all abnormal cells have been removed. | B | B | B |
| Mistral-7B-v0.2 | For a patient with a histopathological confirmation of HSIL and a TZ3 classification, the following steps should be conducted:  a. Repeat colposcopy in 3-6 months.  b. Consider repeating cervical conization or additional diagnostic procedures (like MRI, CT, or PET scan) if the TZ3 lesion is not completely removed or if there are concerns for deeper invasion. | B | C | B |
| Starling-LM-7B alpha (With prompt) | 49. In cervical cancer screening, if a patient has a histopathological confirmation (biopsy) of HSIL and a TZ3 classification, the following follow-up steps should be conducted:  a. Perform a loop electrosurgical excision procedure (LEEP) or cold knife conization (CKC) to remove the affected tissue. b. If the patient has a history of high-grade lesions or cervical intraepithelial neoplasia (CIN) c, consider referring her for a hysterectomy or other surgical intervention. d. Follow-up with colposcopy and cytology at regular intervals to monitor for recurrence or progression of the lesion. | B | B | B |
| Starling-LM-7B alpha | In cervical cancer screening, if a patient has a histopathological confirmation (biopsy) of HSIL and a TZ3 classification, a colposcopy should be performed as a follow-up step. If the colposcopy findings are negative, the patient may be observed regularly. If the colposcopy findings are positive, the patient may need further diagnostic tests such as endocervical curettage (ECC). Treatment options will vary depending on the results of these tests, which may include observation, loop electrical excision procedure (LEEP), or cryotherapy. | C | C | C |
| HuatuoGPT (With prompt) | 49. If a pathologically confirmed biopsy (e.g., colposcopy) shows that the patient has HSIL with a TZ3 grade result, what additional measures should be performed next? Answer: Periodic pap smear monitoring should be performed. | D | C | C |
| HuatuoGPT | If a patient has high-grade squamous intraepithelial lesions (HSIL) confirmed by histopathology (biopsy) and a cervical smear with TZ3 classification is performed, the following follow-up steps should be performed: a. Perform colposcopy to further assess the extent of cervical lesions. b. Depending on the results of the colposcopy, a cervical conization (Cervical Conization) may be necessary to obtain a more accurate diagnosis and treatment. c. Perform cervical smears and colposcopies on a regular basis to monitor the progression or disappearance of the lesion. d. Consider HPV vaccination to prevent future HPV infection. e. Consultation with a gynecologist is recommended to develop an individualized treatment plan for each case. | D | D | D |
| BioMedLM 2.7B (With prompt) | 49. In cervical cancer screening, if a patient has a histopathological confirmation (biopsy) of HSIL and a TZ3 classification, what follow-up steps should be conducted? The patient should be referred to a gynecologist for colposcopy and biopsy. | D | D | D |
| BioMedLM 2.7B | If the patient has a histopathological confirmation (biopsy) of HSIL and a TZ1 or TZ2 classification, then follow up with colposcopy at 6 months after treatment. | D | D | D |
| Microsoft Phi2 (With prompt) | 49. If a patient has a histopathological confirmation (biopsy) of HSIL and a TZ3 classification, the patient should be referred for colposcopy. If the colposcopy is normal, the patient should be re-screened in 3 years. If the colposcopy is abnormal, the patient should be referred for a cone biopsy. | D | D | D |
| Microsoft Phi2 | If a patient has a histopathological confirmation (biopsy) of high-grade squamous intraepithelial lesion (HSIL), which is classified as Tz3 in cervical cancer screening, then further examination and treatment are required. Similar to Tz1/2, patients who have received negative biopsies will undergo repeat cytology every 3 months until they reach 5 consecutive normal results; if there is any abnormality detected at this time point, it may need to be repeated immediately; If the result is abnormal again after one year, a repeat biopsy can be performed. After five consecutive normal results, annual testing is sufficient. | D | D | D |
| QiZhenGPT (With prompt) | 49. In cervical cancer screening, if a patient has a histopathological confirmation (biopsy) of HSIL and a TZ3 classification, the following follow-up steps should be conducted: a. Follow-uposcopy: This involves performing a colposcopy or a standardoscopyoscopy on the patient to visualize the margins of the biopsy and to identify any potential cancer cells that may have been missed during the biopsy.  b. Follow-up DNA testing: If the histopathological confirmation is TZ3, the patient may need to undergo follow-up DNA testing to confirm the diagnosis and to determine the risk of developing cervical cancer in the future.  c. Follow-up treatment: If the patient is at high risk of developing cervical cancer, additional treatment may be recommended, such as radiation therapy or chemotherapy.  d. Follow-up care: The patient should continue to receive follow-up care, including regular check-ups with a gynecologist and any additional testing that may be needed to monitor the patient's health. | D | D | D |
| QiZhenGPT | if the patient has pathologically confirmed (biopsy) HSIL on cervical screening and is classified as TZ3, the following follow-up steps should be performed: * Perform cytologic follow-up, including cervical cytology (pap smear) every 3-6 months * Perform colposcopy, every 3-6 months * Consider colposcopy every 12-18 months if three consecutive cytologic follow-ups are normal | D | C | D |
| **50.In cervical cancer screening, if a patient aged 21 to 24 years has a histopathological confirmation(biopsy) of CIN III/HSIL, what follow-up steps should be conducted?** | | | | |
| ChatGPT4.0 turbo (With prompt) | 50. Follow-up for Young Patients (21-24 Years) with CIN III/HSIL: For young patients aged 21 to 24 years with a biopsy-confirmed cervical intraepithelial neoplasia grade III (CIN III) or HSIL, conservative management can be considered, given the potential for regression and the implications for future reproductive health. Management options include close follow-up with repeat colposcopy and cytology at 6 and 12 months. If the lesion persists or progresses, or if the patient desires definitive treatment, an excisional procedure may be considered. | C | B | C |
| ChatGPT4.0 turbo | Aged 21 to 24, CIN III/HSIL Histopathology:  - For young women aged 21 to 24 with a biopsy-confirmed CIN III/HSIL, management may be more conservative due to the higher likelihood of regression and the potential impact on future fertility. Close follow-up with repeat colposcopy and cytology at 12-month intervals may be recommended, with excisional treatment considered for persistent lesions. | C | C | C |
| Claude 2 (With prompt) | 50. For 21-24 year olds with confirmed CIN III/HSIL on biopsy, either conservative management or excision are acceptable options. Conservative option requires very close follow up at 6 month intervals with co-testing to document regression. | C | C | C |
| Claude 2 | For ages 21-24 with confirmed CIN III/HSIL - perform diagnostic excisional procedure. | A | A | A |
| Gemini Pro (With prompt) | 50. In cervical cancer screening, if a patient aged 21 to 24 years has a histopathological confirmation (biopsy) of CIN III/HSIL, what follow-up steps should be conducted? Repeat cytology and HPV testing in 6 months | D | D | D |
| Gemini Pro | Conization or LEEP. | C | C | C |
| Mistral-7B-v0.2 (With prompt) | 50.In cervical cancer screening for patients aged 21 to 24 years, if there is a histopathological confirmation (biopsy) of CIN III/HSIL (cervical intraepithelial neoplasia grade III/high-grade squamous intraepithelial lesion), a loop electrosurgical excision procedure (LEEP) or cold knife conization may be recommended to remove the abnormal tissue. A repeat cytological examination in 12 months is also recommended after the procedure to ensure that all abnormal cells have been removed. | B | B | B |
| Mistral-7B-v0.2 | For a patient aged 21 to 24 with a histopathological confirmation of CIN III/HSIL, the following steps should be conducted:  a. Repeat colposcopy and cytology within 12 months for confirmation of complete resolution of CIN/HSIL.  b. HPV testing at 12 months and annually if persistent HPV infection is detected.  c. Consider referral for specialized gynecological care. | D | D | D |
| Starling-LM-7B alpha (With prompt) | 50. In cervical cancer screening, if a patient aged 21 to 24 years has a histopathological confirmation (biopsy) of CIN III/HSIL, the following follow-up steps should be conducted:  a. Perform a loop electrosurgical excision procedure (LEEP) or cold knife conization (CKC) to remove the affected tissue. b. Follow-up with colposcopy and cytology at regular intervals to monitor for recurrence or progression of the lesion. c. If the patient has a history of high-grade lesions or cervical intraepithelial neoplasia (CIN) 3, consider referring her for a hysterectomy or other surgical intervention. | B | B | B |
| Starling-LM-7B alpha | In cervical cancer screening, if a patient aged 21 to 24 years has a histopathological confirmation (biopsy) of CIN III/HSIL, a colposcopy should be performed as a follow-up step. If the colposcopy findings are negative, the patient may be observed regularly. If the colposcopy findings are positive, the patient may need further diagnostic tests such as endocervical curettage (ECC). Treatment options will vary depending on the results of these tests, which may include loop electrical excision procedure (LEEP), cryotherapy, or cone biopsy. | C | C | C |
| HuatuoGPT (With prompt) | 50. If a patient between the ages of 21 and 24 years has pathologic confirmation of CIN III/HSIL on biopsy (e.g., colposcopy) and a TZ1/2 grade result on colposcopy, what additional measures should be performed next? ANSWER: Periodic pap smear monitoring should be performed. | D | D | D |
| HuatuoGPT | If a patient aged 21 to 24 years has histopathologically confirmed (biopsy) CIN III/HSIL, the following follow-up steps should be performed: a. Colposcopy is performed to further evaluate the extent of cervical lesions. b. Depending on the results of the colposcopy, cervical conization (Cervical Conization) may be necessary to obtain a more accurate diagnosis and treatment. c. Perform cervical smears and colposcopies on a regular basis to monitor the progression or disappearance of the lesion. d. Consider HPV vaccination to prevent future HPV infection. e. Consultation with a gynecologist is recommended to develop an individualized treatment plan for each case. | D | D | D |
| BioMedLM 2.7B (With prompt) | 50. In cervical cancer screening, if a patient aged 21 to 24 years has a histopathological confirmation (biopsy) of CIN III/HSIL, what follow-up steps should be conducted? Follow-up is recommended for patients with CIN III/HSIL. The patient should be referred to a gynecologist for follow-up. The patient should be followed up every 3 months for the first year, every 6 months for the second year, and annually thereafter. | D | D | D |
| BioMedLM 2.7B | The patient should undergo colposcopy and biopsy at 6 months after treatment with LEEP or cryotherapy. If the patient has no evidence of disease on colposcopic examination and biopsy, she can return for annual follow up. | D | D | D |
| Microsoft Phi2 (With prompt) | 50. If a patient aged 21 to 24 years has a histopathological confirmation (biopsy) of CIN III/HSIL, the patient should undergo colposcopy. If the colposcopy is normal, the patient should be rescreened in 3 years. If the colposcopy is abnormal, the patient should undergo a repeat biopsy. If the repeat biopsy is normal, the patient should be rescreened in 3 years. If the repeat biopsy is abnormal, the patient should undergo treatment. | D | D | D |
| Microsoft Phi2 | If a patient aged between 21 and 24 years has a histopathological confirmation (biopsy) of low-grade squamous intraepithelial lesion (CIN III/HSIL), then regular surveillance through Pap smear is recommended once every three years. In addition, HPV DNA testing may also be used. | D | D | D |
| QiZhenGPT (With prompt) | 50. In cervical cancer screening, if a patient aged 21 to 24 years has a histopathological confirmation (biopsy) of CIN III/HSIL, the following follow-up steps should be conducted: a. Follow-uposcopy: This involves performing a colposcopy or a standardoscopyoscopy on the patient to visualize the margins of the biopsy and to identify any potential cancer cells that may have been missed during the biopsy.  b. Follow-up DNA testing: If the histopathological confirmation is CIN III/HSIL, the patient may need to undergo follow-up DNA testing to confirm the diagnosis and to determine the risk of developing cervical cancer in the future.  c. Follow-up treatment: If the patient is at high risk of developing cervical cancer, additional treatment may be recommended, such as radiation therapy or chemotherapy.  d. Follow-up care: The patient should continue to receive follow-up care, including regular check-ups with a gynecologist and any additional testing that may be needed to monitor the patient's health. | D | D | D |
| QiZhenGPT | if the patient is between 21 and 24 years of age with pathologic confirmation (biopsy) of CIN III/HSIL, then the following follow-up steps should be performed: * Perform cytologic follow-up, including cervical cytology every 3-6 months (Pap smear) * Perform colposcopy, every 6-12 months * Perform HPV viral testing * Consider colposcopy every 12-18 months if two consecutive cervical cytology tests have normal results | D | C | C |
| **51.In cervical cancer screening, if a patient aged 21 to 24 years has a histopathological confirmation(biopsy) of HSIL, and a TZ3 classification for colposcopy, what follow-up steps should be conducted?** | | | | |
| ChatGPT4.0 turbo (With prompt) | 51. Follow-up for Young Patients (21-24 Years) with HSIL and TZ3 Classification: For patients in this age group with HSIL and a TZ3 classification, an initial approach involving repeat colposcopy with endocervical assessment is recommended. Given the challenges in fully assessing TZ3, it is crucial to ensure comprehensive evaluation. If the lesion persists on follow-up or if there are concerns about the adequacy of visualization or sampling, consideration for an excisional procedure may be necessary. | C | C | C |
| ChatGPT4.0 turbo | Aged 21 to 24, HSIL Histopathology, TZ3 Classification:  - Similar to scenario 3, conservative management with close follow-up may be considered due to the age of the patient. The decision between immediate excisional treatment versus observation with colposcopy and cytology will depend on several factors, including patient preferences, lesion appearance, and provider judgment. | D | C | C |
| Claude 2 (With prompt) | 51. If TZ 3 colposcopy in this age group, indicating high grade disease, guidelines allow either treatment or deferring treatment with close 6 month co-test follow up. Treatment is warranted if repeat HSIL cytology or persisting lesion. | D | D | D |
| Claude 2 | For ages 21-24 with confirmed HSIL and TZ3 colposcopy - follow up with repeat colposcopy and ECC in 6 months. Consider excisional procedure based on results. | B | B | B |
| Gemini Pro (With prompt) | 51. In cervical cancer screening, if a patient aged 21 to 24 years has a histopathological confirmation (biopsy) of HSIL and a TZ3 classification for colposcopy, what follow-up steps should be conducted? Loop electrosurgical excision procedure (LEEP) or cold knife conization | B | B | B |
| Gemini Pro | Radical hysterectomy or chemoradiation. | C | C | C |
| Mistral-7B-v0.2 (With prompt) | 51.In cervical cancer screening for patients aged 21 to 24 years, if there is a histopathological confirmation (biopsy) of HSIL (high-grade squamous intraepithelial lesion) and a TZ3 (severe dyskaryosis) classification for colposcopy, a loop electrosurgical excision procedure (LEEP) or cold knife conization may be recommended to remove the abnormal tissue. A repeat cytological examination in 12 months is also recommended after the procedure to ensure that all abnormal cells have been removed. | B | B | B |
| Mistral-7B-v0.2 | For a patient aged 21 to 24 with a histopathological confirmation of HSIL and a TZ3 classification for colposcopy, the following steps should be conducted:  a. Repeat colposcopy in 3-6 months.  b. Consider repeating cervical conization or additional diagnostic procedures (like MRI, CT, or PET scan) if the TZ3 lesion is not completely removed or if there are concerns for deeper invasion. | B | A | A |
| Starling-LM-7B alpha (With prompt) | 51. In cervical cancer screening, if a patient aged 21 to 24 years has a histopathological confirmation (biopsy) of HSIL and a TZ3 classification for colposcopy, the following follow-up steps should be conducted:  a. Perform a loop electrosurgical excision procedure (LEEP) or cold knife conization (CKC) to remove the affected tissue. b. Follow-up with colposcopy and cytology at regular intervals to monitor for recurrence or progression of the lesion. c. If the patient has a history of high-grade lesions or cervical intraepithelial neoplasia (CIN) 3, consider referring her for a hysterectomy or other surgical intervention. | B | B | B |
| Starling-LM-7B alpha | In cervical cancer screening, if a patient aged 21 to 24 years has a histopathological confirmation (biopsy) of HSIL and a TZ3 classification for colposcopy, a colposcopy should be performed as a follow-up step. If the colposcopy findings are negative, the patient may be observed regularly. If the colposcopy findings are positive, the patient may need further diagnostic tests such as endocervical curettage (ECC). Treatment options will vary depending on the results of these tests, which may include loop electrical excision procedure (LEEP), cryotherapy, or cone biopsy. | D | C | D |
| HuatuoGPT (With prompt) | 51. If a patient between the ages of 21 and 24 years has pathologic confirmation of HSIL on biopsy (e.g., colposcopy) and a colposcopic result of TZ grade 3, and regular postcolposcopic biopsy pap smear surveillance is performed, what additional measures should be performed next? Answer: Periodic pap smear monitoring should be performed. | D | D | D |
| HuatuoGPT | If a patient aged 21 to 24 years has confirmed (biopsied) HSIL by histopathology and has undergone colposcopy for TZ3 classification, the following follow-up steps should be performed: a. Perform colposcopy to further assess the extent of cervical pathology. b. Depending on the results of the colposcopy, a cervical conization (Cervical Conization) may be necessary to obtain a more accurate diagnosis and treatment. c. Perform cervical smears and colposcopies on a regular basis to monitor the progression or disappearance of the lesion. d. Consider HPV vaccination to prevent future HPV infection. e. Consultation with a gynecologist is recommended to develop an individualized treatment plan for each case. | C | C | C |
| BioMedLM 2.7B (With prompt) | 51. In cervical cancer screening, if a patient aged 21 to 24 years has a histopathological confirmation (biopsy) of HSIL and a TZ3 classification for colposcopy, what follow-up steps should be conducted? The patient should be referred to a gynecologist for colposcopy and biopsy. | D | D | D |
| BioMedLM 2.7B | The patient should undergo colposcopic examination and biopsy at 6 months after treatment with LEEP or cryotherapy. If the result shows no evidence of CIN2+ (CIN2/3),the patient can return to routine screening. | D | D | D |
| Microsoft Phi2 (With prompt) | 51. If a patient aged 21 to 24 years has a histopathological confirmation (biopsy) of HSIL and a TZ3 classification for colposcopy, the patient should undergo colposcopy. If the colposcopy is normal, the patient should be rescreened in 3 years. If the colposcopy is abnormal, the patient should undergo a repeat biopsy. If the repeat biopsy is normal, the patient should be rescreened in 3 years. If the repeat biopsy is abnormal, the patient should undergo treatment. | D | D | D |
| Microsoft Phi2 | If a patient aged between 21 and 24 years has a histopathological confirmation (biopsy) of high-grade squamous intraepithelial lesion (HSIL), and a TZ3 classification for colposcopy, then regular surveillance through Pap smear is recommended once every three years. In addition, HPV DNA testing may also be used. | D | D | D |
| QiZhenGPT (With prompt) | 51. In cervical cancer screening, if a patient aged 21 to 24 years has a histopathological confirmation (biopsy) of HSIL and a TZ3 classification for colposcopy, the following follow-up steps should be conducted: a. Follow-uposcopy: This involves performing a colposcopy on the patient to visualize the margins of the biopsy and to identify any potential cancer cells that may have been missed during the biopsy.  b. Follow-up DNA testing: If the histopathological confirmation is TZ3, the patient may need to undergo follow-up DNA testing to confirm the diagnosis and to determine the risk of developing cervical cancer in the future.  c. Follow-up treatment: If the patient is at high risk of developing cervical cancer, additional treatment may be recommended, such as radiation therapy or chemotherapy.  d. Follow-up care: The patient should continue to receive follow-up care, including regular check-ups with a gynecologist and any additional testing that may be needed to monitor the patient's health. | C | D | D |
| QiZhenGPT | If the patient is between 21 and 24 years of age, has pathologic confirmation of HSIL (biopsy), and the colposcopy result shows a TZ3 classification, then the following follow-up steps should be performed: * Perform cytologic follow-up, including cervical cytology (pap smear) every 3-6 months * Perform colposcopy, every 6-12 months * Perform HPV viral testing * Consider colposcopy every 12-18 months if two consecutive cervical cytology tests have normal results | C | D | D |
| **52.In cervical cancer screening, if a patient aged 21 to 24 years has a histopathological confirmation(biopsy) of CINⅡ/III/HSIL or CINⅡ/HSIL, and a TZ1/2 classification for colposcopy, what follow-up steps should be conducted?** | | | | |
| ChatGPT4.0 turbo (With prompt) | 52. Follow-up for Young Patients (21-24 Years) with CIN II/III/HSIL and TZ1/2 Classification: For these patients, initial management should involve colposcopy and possibly an excisional procedure, depending on the specific circumstances and patient preferences regarding fertility preservation. If opting for conservative management, close follow-up with repeat colposcopy and cytology at 6 and 12 months is essential. This strategy balances the risk of progression with the potential for regression and the impact on reproductive outcomes. If the lesion persists or progresses, an excisional procedure should be strongly considered. | D | D | D |
| ChatGPT4.0 turbo | Aged 21 to 24, CIN II/III/HSIL or CIN II/HSIL Histopathology, TZ1/2 Classification:  - Young women with CIN II may be managed with observation and follow-up colposcopy and cytology, while those with CIN III/HSIL may be considered for excisional treatment. The decision will be influenced by the extent of the lesion, the patient's reproductive plans, and the visibility of the transformation zone. | C | C | C |
| Claude 2 (With prompt) | 52. Conservative management is appropriate for CIN II-III/HSIL histology in patients less than age 25 if TZ1/TZ2 colposcopy, with co-testing follow up at 6 & 12 months. Treatment can be done though if persistence or repeat HSIL cytology. | A | A | A |
| Claude 2 | For ages 21-24 with confirmed CIN II/III/HSIL and TZ1/2 colposcopy - repeat co-testing at 6 and 12 months or diagnostic excisional procedure. | B | B | B |
| Gemini Pro (With prompt) | 52. In cervical cancer screening, if a patient aged 21 to 24 years has a histopathological confirmation (biopsy) of CIN II/III/HSIL or CIN II/HSIL and a TZ1/2 classification for colposcopy, what follow-up steps should be conducted? Repeat cytology and HPV testing in 6 months | A | A | A |
| Gemini Pro | Conization or LEEP. | C | D | D |
| Mistral-7B-v0.2 (With prompt) | 52.In cervical cancer screening for patients aged 21 to 24 years, if there is a histopathological confirmation (biopsy) of CIN II/III/HSIL (cervical intraepithelial neoplasia grade II/III/high-grade squamous intraepithelial lesion) or CIN II/HSIL (cervical intraepithelial neoplasia grade II/high-grade squamous intraepithelial lesion) and a TZ1/2 (mild or moderate dyskaryosis) classification for colposcopy, a repeat cytological examination in 12 months is recommended. | A | A | A |
| Mistral-7B-v0.2 | For a patient aged 21 to 24 with a histopathological confirmation of CINⅡ/III/HSIL or CINⅡ/HSIL, and a TZ1/2 classification for colposcopy, the following steps should be conducted:  a. Repeat colposcopy and cytology in 6-12 months.  b. HPV testing at 12 months and annually if persistent HPV infection is detected. | B | B | B |
| Starling-LM-7B alpha (With prompt) | 52. In cervical cancer screening, if a patient aged 21 to 24 years has a histopathological confirmation (biopsy) of CIN II/III/HSIL or CIN II/HSIL and a TZ1/2 classification for colposcopy, the following follow-up steps should be conducted:  a. Repeat colposcopy in 3-6 months to assess the persistence or regression of the lesion. b. If the lesion persists or progresses, consider performing a loop electrosurgical excision procedure (LEEP) or cold knife conization (CKC) to remove the affected tissue. c. If the lesion regresses, consider repeating the colposcopy in 6-12 months to monitor for recurrence. d. If the patient has a history of high-grade lesions or cervical intraepithelial neoplasia (CIN) 3, consider referring her for a hysterectomy or other surgical intervention. | A | A | A |
| Starling-LM-7B alpha | In cervical cancer screening, if a patient aged 21 to 24 years has a histopathological confirmation (biopsy) of CIN II/III/HSIL or CIN II/HSIL, and a TZ1/2 classification for colposcopy, a colposcopy should be performed as a follow-up step. If the colposcopy findings are negative, the patient may be observed regularly. If the colposcopy findings are positive, the patient may need further diagnostic tests such as endocervical curettage (ECC). Treatment options will vary depending on the results of these tests, which may include loop electrical excision procedure (LEEP), cryotherapy, or cone biopsy. | D | C | D |
| HuatuoGPT (With prompt) | 52. if a patient between the ages of 21 and 24 years old has a biopsy at | D | D | D |
| HuatuoGPT | If a patient aged 21 to 24 years is confirmed (biopsied) by histopathology to have CIN II/III/HSIL or CIN II/HSIL and a colposcopy with TZ1/2 classification is performed, the following follow-up steps should be performed: a. Perform a colposcopy to further assess the extent of cervical pathology. b. Depending on the results of the colposcopy, cervical conization (Cervical Conization) may be necessary to obtain a more accurate diagnosis and treatment. c. Perform cervical smears and colposcopies on a regular basis to monitor the progression or disappearance of the lesion. d. Consider HPV vaccination to prevent future HPV infection. e. Consultation with a gynecologist is recommended to develop an individualized treatment plan for each case. | D | D | D |
| BioMedLM 2.7B (With prompt) | 52. In cervical cancer screening, if a patient aged 21 to 24 years has a histopathological confirmation (biopsy) of CIN II/III/HSIL or CIN II/HSIL and a TZ1/2 classification for colposcopy, what follow-up steps should be conducted? Follow-up is recommended for patients with CIN II/III/HSIL or CIN II/HSIL and TZ1/2 classification for colposcopy. | B | B | B |
| BioMedLM 2.7B | If the patient is aged 25 to 29 years and has a histopathological diagnosis (biopsy) of CIN Ⅲ/AIS or CIN Ⅲ/AIS with a TZ3 classification for colposcopy, then follow up at 3 months after treatment by cytology and HPV testing; if the results are negative, then repeat cytology and HPV testing every year until 2 consecutive tests are negative. | C | C | C |
| Microsoft Phi2 (With prompt) | 52. If a patient aged 21 to 24 years has a histopathological confirmation (biopsy) of CIN II/III/HSIL or CIN II/HSIL and a TZ1/2 classification for colposcopy, the patient should undergo colposcopy. If the colposcopy is normal, the patient should be rescreened in 3 years. If the colposcopy is abnormal, the patient should undergo a repeat biopsy. If the repeat biopsy is normal, the patient should be rescreened in 3 years. If the repeat biopsy is abnormal, the patient should undergo treatment. | B | B | B |
| Microsoft Phi2 | If a patient aged between 21 and 24 years has a histopathological confirmation (biopsy) of low-grade squamous intraepithelial lesion (CIN Ⅱ/III/HSIL or CIN Ⅱ/HSIL), and a TZ1/2 classification for colposcopy, then regular surveillance through Pap smear is recommended once every three years. In addition, HPV DNA testing may also be used. | C | C | C |
| QiZhenGPT (With prompt) | 52. In cervical cancer screening, if a patient aged 21 to 24 years has a histopathological confirmation (biopsy) of CIN II/III/HSIL or CIN II/HSIL and a TZ1/2 classification for colposcopy, the following follow-up steps should be conducted: a. Follow-uposcopy: This involves performing a colposcopy or a direct oscopyoscopy of the gastrointestinal tract to visualize any abnormalities or changes in the skin or internal organs.  b. Follow-up DNA testing: If the histopathological confirmation is CIN II/III/HSIL or CIN II/HSIL, the patient may need to undergo DNA testing to determine if they have a risk of developing cervical cancer.  c. Follow-up treatment: If the patient has a TZ1/2 classification, they may need to undergo a treatment plan that includes radiation therapy or a combination of radiation therapy and chemotherapy.  d. Follow-up care: The patient should continue to receive follow-up care and monitoring for any changes in their health. | C | C | C |
| QiZhenGPT | If the patient is between 21 and 24 years of age, has pathologic confirmation (biopsy) of CIN II/III/HSIL or CIN II/HSIL, and the colposcopy results show a TZ1/2 classification, then the following follow-up steps should be performed: * Perform cytologic follow-up, including cervical cytology (pap smear) every 3-6 months * Perform colposcopy, every 6-12 months * Perform HPV viral testing * Consider colposcopy every 12-18 months if two consecutive cervical cytology tests have normal results. | C | D | C |
| **53.In cervical cancer screening, if a pregnant patient has a histopathological confirmation(biopsy) of HSIL, what follow-up steps should be conducted?** | | | | |
| ChatGPT4.0 turbo (With prompt) | 53. Follow-up for Pregnant Patient with HSIL: In a pregnant patient with a histopathological confirmation of high-grade squamous intraepithelial lesion (HSIL), the primary approach is conservative management during the pregnancy, unless there is evidence of invasive cancer. Colposcopy should be performed to assess the extent and severity of the lesion. Biopsy during pregnancy is generally avoided unless absolutely necessary to rule out invasive disease. The follow-up plan typically involves close monitoring and repeat colposcopy to ensure that there is no progression of the lesion during the pregnancy. Definitive treatment, such as an excisional procedure, is usually deferred until after delivery. | B | B | B |
| ChatGPT4.0 turbo | Pregnant Patient, HSIL Histopathology:  - Pregnant women with HSIL on biopsy are usually monitored with colposcopy during pregnancy, delaying treatment until postpartum unless invasive cancer is suspected. Biopsy or treatment during pregnancy is generally avoided due to the risk of complications. | A | A | A |
| Claude 2 (With prompt) | 53. For HSIL on cervical biopsy in a pregnant patient, recommendations are to defer excisional treatment until after delivery. Follow with repeat colposcopy during pregnancy only if the lesion appears progressive. Post-partum co-testing is warranted with management based on subsequent results. | B | B | B |
| Claude 2 | For pregnant patients with confirmed HSIL - colposcopy with biopsy deferred until >12 weeks postpartum, then proceed based on results. | C | D | C |
| Gemini Pro (With prompt) | 53. In cervical cancer screening, if a pregnant patient has a histopathological confirmation (biopsy) of HSIL, what follow-up steps should be conducted? Colposcopy with biopsy after delivery | C | C | C |
| Gemini Pro | Repeat colposcopy and biopsy after delivery. | B | B | B |
| Mistral-7B-v0.2 (With prompt) | 53.If the repeat cytological examination is also negative, no further testing may be necessary, unless the individual has other risk factors for cervical cancer. However, if the repeat cytological examination is also abnormal, or if the individual has other risk factors for cervical cancer, a loop electrosurgical excision procedure (LEEP) or cold knife conization may be recommended to remove the abnormal tissue. A repeat cytological examination in 12 months after the procedure is also rIn cervical cancer screening for pregnant patients, if there is a histopathological confirmation (biopsy) of HSIL (high-grade squamous intraepithelial lesion), a repeat cytological examination in 12 months after delivery is recommended. If the repeat cytological examination is also negative, no further testing may be necessary, unless the individual has other risk factors for cervical cancer. However, if the repeat cytological examination is also abnormal, or if the individual has other risk factors for cervical cancer, a colposcopy with biopsy may be recommended. | B | B | B |
| Mistral-7B-v0.2 | For a pregnant patient with a histopathological confirmation of HSIL, the following steps should be conducted:  a. Careful monitoring with serial colposcopy and/or transvaginal ultrasound.  b. The patient may need to be delivered early if there's concern for progression or complications, but many cases can be safely managed through pregnancy.  c. Follow-up after childbirth should include an examination under anesthesia, cervical conization if necessary, and standard follow-up as recommended. | B | B | B |
| Starling-LM-7B alpha (With prompt) | 53. In the case of a pregnant patient with a histopathological confirmation (biopsy) of high-grade squamous intraepithelial lesion (HSIL), the follow-up steps should include:  a. Close monitoring of the patient's pregnancy, as the presence of HSIL may increase the risk of preterm birth and low birth weight. b. Regular ultrasound examinations to monitor the progression of the pregnancy and the fetus. c. Postpartum evaluation, including a repeat biopsy to confirm the persistence or regression of the HSIL. d. If the HSIL persists postpartum, further management options such as loop electrosurgical excision procedure (LEEP) or cold knife conization (CKC) may be considered, depending on the patient's clinical status and preferences. e. Regular follow-up appointments and cervical cancer screening, as per the standard guidelines, after the patient has completed her family planning. | D | D | D |
| Starling-LM-7B alpha | In cervical cancer screening, if a pregnant patient has a histopathological confirmation (biopsy) of HSIL, a colposcopy should be performed as a follow-up step. If the colposcopy findings are negative, the patient will be monitored during pregnancy and closely examined after delivery. If the colposcopy findings are positive, further diagnostic tests such as endocervical curettage (ECC) may be performed. Treatment options will vary depending on the results of these tests and the stage of pregnancy, which may include observation, loop electrical excision procedure (LEEP), or cone biopsy. | A | A | A |
| HuatuoGPT (With prompt) | 53. If a pregnant patient is diagnosed with high-grade squamous intraepithelial lesion (HSIL) by histopathologic examination (biopsy), what additional steps should be performed next? Answer: Periodic pap smear monitoring should be performed. | D | D | D |
| HuatuoGPT | If a pregnant woman has histopathologically confirmed (biopsy) HSIL, the following follow-up steps should be performed: a. Colposcopy is performed to further assess the extent of cervical pathology. b. Depending on the results of the colposcopy, a cervical conization (Cervical Conization) may be necessary to obtain a more accurate diagnosis and treatment. c. Perform cervical smears and colposcopies on a regular basis to monitor the progression or disappearance of the lesion. d. Consider HPV vaccination to prevent future HPV infection. e. Consultation with a gynecologist is recommended to develop an individualized treatment plan for each case. | C | D | C |
| BioMedLM 2.7B (With prompt) | 53. In cervical cancer screening, if a pregnant patient has a histopathological confirmation (biopsy) of HSIL, what follow-up steps should be conducted? A pregnant patient with a histopathological confirmation (biopsy) of HSIL should be followed up with colposcopy and biopsy every 3 months until delivery. | A | A | A |
| BioMedLM 2.7B | The patient should undergo colposcopy and biopsy to confirm the diagnosis. | C | D | D |
| Microsoft Phi2 (With prompt) | 53.In cervical cancer screening, if a pregnant patient has a histopathological confirmation (biopsy) of HSIL, the patient should be referred to a gynecologic oncologist for further evaluation and management. The patient should undergo a repeat Pap smear and HPV testing at 6 months post-biopsy. If the results of the repeat Pap smear and HPV testing are negative, the patient can be followed up with a Pap smear every 3 years. If the results of the repeat Pap smear and HPV testing are positive, the patient should undergo further evaluation and management, which may include colposcopy, biopsy, and treatment. | D | D | D |
| Microsoft Phi2 | Pregnant women with a histopathological confirmation (biopsy) of HSIL do not require additional examinations or treatments unless other abnormalities are found on ultrasound imaging. Regular monitoring through routine prenatal care is usually sufficient. | D | D | D |
| QiZhenGPT (With prompt) | 53. In cervical cancer screening, if a pregnant patient has a histopathological confirmation (biopsy) of HSIL, what follow-up steps should be conducted? Answer: Follow-up with a routine cervical cancer screening test (CCT) to detect any new cases of cervical cancer in the future.   Follow-up with a routine ASC-US CT to detect any new cases of ASC-US in the future.   Follow-up with a routine HSIL CT to detect any new cases of HSIL in the future. | C | C | C |
| QiZhenGPT | if a pregnant woman has pathologic confirmation of HSIL on cervical screening (biopsy), then the following follow-up steps should be performed: * Perform cytologic follow-up, including cervical cytology every 3-6 months (Pap smear) * Perform colposcopy, every 6-12 months * Perform HPV viral testing * Consider colposcopy every 12-18 months if two consecutive cervical cytology tests have normal results. | C | C | C |
| **54.After Diagnostic/Therapeutic Cervical Conization for cervical cancer, what follow-up steps should be conducted?** | | | | |
| ChatGPT4.0 turbo (With prompt) | 54. Follow-up After Cervical Conization for Cervical Cancer: After a patient undergoes diagnostic or therapeutic cervical conization, close follow-up is essential to monitor for residual or recurrent disease. This typically includes:  - A follow-up visit approximately 4-6 weeks post-procedure to check for healing and to discuss pathology results.  - Co-testing with HPV DNA testing and cervical cytology at 12 months post-procedure. If the results are negative, then co-testing should be repeated every 3 years. However, if any test is positive, a more immediate evaluation with colposcopy should be considered.  - Annual pelvic examination to assess for any clinical signs suggestive of recurrence.  - The specific follow-up schedule may be adjusted based on individual risk factors, including the margins of the conization specimen, the presence of high-risk HPV types, and the original severity of the lesion. | C | C | C |
| ChatGPT4.0 turbo | After Diagnostic/Therapeutic Cervical Conization:  - Follow-up after conization typically includes colposcopy and cytology at 4-6 months post-procedure. If the margins are clear and the follow-up is normal, patients may return to routine screening. If the margins are positive, closer follow-up or further treatment may be necessary. | B | B | B |
| Claude 2 (With prompt) | 54. For patients undergoing excisional treatment like LEEP or cold knife conization, post-procedure surveillance recommendations are: - Negative margins: Co-testing at 12 months and 24 months looking for possible recurrence  - Positive margins: Repeat excision if possible or close follow up with co-testing every 6 months | B | C | B |
| Claude 2 | After diagnostic/therapeutic conization - co-testing at 12 and 24 months post-procedure. | C | D | C |
| Gemini Pro (With prompt) | 54. After diagnostic/therapeutic cervical conization for cervical cancer, what follow-up steps should be conducted? Cytology and HPV testing every 6 months for 2 years, then annually | C | C | C |
| Gemini Pro | Repeat cytology and HPV testing in 6 months, then annually for 5 years. | D | C | C |
| Mistral-7B-v0.2 (With prompt) | 54.After diagnostic/therapeutic cervical conization for cervical cancer, a repeat cytological examination in 12 months is recommended to ensure that all abnormal cells have been removed. If the repeat cytological examination is also negative, no further testing may be necessary, unless the individual has other risk factors for cervical cancer. Regular follow-up appointments and screening tests are recommended based on age and other risk factors. In cervical cancer screening for pregnant patients, if there is a histopathological confirmation (biopsy) of HSIL (high-grade squamous intraepithelial lesion) with no invasive cancer during pregnancy, a repeat cytological examination in 12 months after childbearing is recommended. | C | C | C |
| Mistral-7B-v0.2 | After diagnostic/therapeutic cervical conization for cervical cancer:  a. Repeat colposcopy and cytology every 4-6 months for the first 2-3 years, and annually thereafter.  b. HPV testing every year.  c. Consider pelvic exams and imaging studies as needed. | D | D | D |
| Starling-LM-7B alpha (With prompt) | 54. After diagnostic/therapeutic cervical conization for cervical cancer, the follow-up steps should include:  a. Pathological examination of the conized tissue to confirm the presence or absence of invasive cancer and the extent of the disease. b. If the pathology report confirms the presence of invasive cancer, further treatment options such as radical hysterectomy, chemoradiation, or chemotherapy may be considered, depending on the stage of the cancer and the patient's clinical status and preferences. c. Regular follow-up appointments, including clinical examinations, imaging studies, and tumor markers, to monitor the patient's response to treatment and detect any recurrence or metastasis. d. Cervical cancer screening, as per the standard guidelines, after the patient has completed her family planning and treatment. | B | B | B |
| Starling-LM-7B alpha | After diagnostic/therapeutic cervical conization for cervical cancer, a follow-up step includes regular follow-up visits with gynecologists, annual cervical cancer screening (Pap smear), and consultation with a specialist (e.g., gynecologic oncologist) if there are any postoperative complications or recurrence of the disease. | C | C | C |
| HuatuoGPT (With prompt) | 54. After cervical conization is performed in the diagnosis/treatment of cervical cancer, what further steps should be performed next? Answer: Regular pap smear monitoring is required. | D | D | D |
| HuatuoGPT | Following diagnosis/treatment of cervical cancer cervical conization, the following follow-up steps should be performed: a. Perform regular cervical smears and colposcopy to monitor the progression or disappearance of the lesion. b. Follow the doctor's recommendations for follow-up treatment and follow up. c. Pay attention to any physical changes or discomfort and report them to your doctor promptly. d. Consider HPV vaccination to prevent future HPV infection. | D | D | D |
| BioMedLM 2.7B (With prompt) | 54. After diagnostic/therapeutic cervical conization for cervical cancer, what follow-up steps should be conducted? Follow-up after cervical conization is important for the early detection of residual or recurrent disease. The follow-up protocol should be individualized based on the patient's risk factors and the results of the initial conization. | C | C | D |
| BioMedLM 2.7B | 1) No further treatment; 2) Cytology; 3) HPV DNA test; 4) Colposcopy; 5) Biopsy | D | D | D |
| Microsoft Phi2 (With prompt) | 54.After diagnostic/therapeutic cervical conization for cervical cancer, the patient should be referred to a gynecologic oncologist for further evaluation and management. The patient should undergo a repeat Pap smear and HPV testing at 6 months post-conization. If the results of the repeat Pap smear and HPV testing are negative, the patient can be followed up with a Pap smear every 3 years. If the results of the repeat Pap smear and HPV testing are positive, the patient should undergo further evaluation and management, which may include colposcopy, biopsy, and treatment. | D | D | D |
| Microsoft Phi2 | After Diagnostic/Therapeutic Cervical Conization(刮宫) for cervical cancer, what follow-up steps should be conducted: -The patient should be referred to a gynecologist for further evaluation and management. -The patient should undergo regular follow-up visits to monitor the condition of the cervix and to detect any changes or abnormalities. -The patient should be advised to undergo regular Pap smears to monitor the health of the cervix. -If necessary, the patient may undergo additional procedures, such as colposcopy or biopsy, to further evaluate the condition of the cervix. | D | D | D |
| QiZhenGPT (With prompt) | 54. After diagnostic/therapeutic cervical conization for cervical cancer, what follow-up steps should be conducted? Answer: Follow-up with a routine cervical cancer screening test (CCT) to detect any new cases of cervical cancer in the future.   Follow-up with a routine ASC-US CT to detect any new cases of ASC-US in the future.   Follow-up with a routine HSIL CT to detect any new cases of HSIL in the future. | C | C | C |
| QiZhenGPT | Following cervical conization (Diagnostic/Therapeutic Cervical Conization), patients should undergo the following follow-up steps: * Perform cytologic follow-up, including cervical cytology every 3-6 months (Pap smear) * Perform colposcopy, every 6-12 months * Perform HPV viral testing * If two consecutive cervical cytology tests are normal, colposcopy every 12-18 months may be considered. | C | C | C |
| **55.In cervical cancer screening, if a pregnant patient has a histopathological confirmation(biopsy) of HSIL without invasive cancer during pregnancy, what follow-up steps should be conducted after childbearing?** | | | | |
| ChatGPT4.0 turbo (With prompt) | 55. Postpartum Follow-up for HSIL: For a pregnant patient with a biopsy-confirmed HSIL and no evidence of invasive cancer, postpartum management is critical. After delivery, the patient should undergo a repeat colposcopy and cervical cytology at the 6-week postpartum visit to reassess the cervical status. This is important as pregnancy-related immunological changes can sometimes lead to regression of cervical dysplasia. If HSIL persists postpartum, management should proceed with an excisional procedure such as LEEP or cold knife conization to treat the lesion and prevent progression to cervical cancer. The timing of this intervention should be discussed with the patient, taking into consideration her recovery from childbirth and her future reproductive plans. | B | A | A |
| ChatGPT4.0 turbo | Pregnant Patient, HSIL Histopathology, No Invasive Cancer, Postpartum:  - After delivery, patients who had HSIL during pregnancy should have a repeat colposcopy and cytology at the postpartum visit to reassess the lesion. If HSIL persists, excisional treatment is usually recommended. | A | A | A |
| Claude 2 (With prompt) | 55. For pregnant patients with HSIL/CIN2-3 biopsy with no cancer, the following post-partum management is appropriate: - Repeat colposcopy 6 weeks after delivery  - Co-testing at 6 month intervals at least twice after delivery - Consider repeat excision if initial or subsequent biopsy shows HSIL, or if lesion persists on colposcopy or with +cytology | A | A | A |
| Claude 2 | For pregnant patients with confirmed HSIL and no invasive cancer - re-evaluate with co-test >12 weeks postpartum, consider excisional procedure based on results. | B | C | C |
| Gemini Pro (With prompt) | 55. In cervical cancer screening, if a pregnant patient has a histopathological confirmation (biopsy) of HSIL with no invasive cancer during pregnancy, what follow-up steps should be conducted after childbearing? Colposcopy with biopsy | D | D | D |
| Gemini Pro | Repeat colposcopy and biopsy. | B | B | B |
| Mistral-7B-v0.2 (With prompt) | 55.If the repeat cytological examination is also negative, no further testing may be necessary, unless the individual has other risk factors for cervical cancer. Regular follow-up appointments and screening tests are recommended based on age and other risk factors. ecommended to ensure that all abnormal cells have been removed. | D | D | D |
| Mistral-7B-v0.2 | For a pregnant patient with a histopathological confirmation of HSIL (high-grade squamous intraepithelial lesion) but no invasive cancer during pregnancy and after childbearing, close monitoring is recommended. Aggressive follow-up may not be necessary unless new abnormalities appear. Some experts suggest repeating cervical cytology tests every 6 to 12 months. However, the follow-up schedule may vary depending on the specific recommendations of the treating healthcare provider. It is essential for the patient to continue regular obstetrical care and consider having a colposcopy and cervical biopsy post-pregnancy to reassess the status of the lesion. | A | A | A |
| Starling-LM-7B alpha (With prompt) | 55. In cervical cancer screening, if a pregnant patient has a histopathological confirmation (biopsy) of HSIL with no invasive cancer during pregnancy, the follow-up steps should include:  a. Close monitoring of the patient's pregnancy, as the presence of HSIL may increase the risk of preterm birth and low birth weight. b. Regular ultrasound examinations to monitor the progression of the pregnancy and the fetus. c. Postpartum evaluation, including a repeat biopsy to confirm the persistence or regression of the HSIL. d. If the HSIL persists postpartum, further management options such as loop electrosurgical excision procedure (LEEP) or cold knife conization (CKC) may be considered, depending on the patient's clinical status and preferences. e. Regular follow-up appointments and cervical cancer screening, as per the standard guidelines, after the patient has completed her family planning. | C | C | C |
| Starling-LM-7B alpha | In cervical cancer screening, if a pregnant patient has a histopathological confirmation (biopsy) of HSIL with no invasive cancer during pregnancy, after childbearing, a colposcopy should be performed as a follow-up step. If the colposcopy findings are negative, the patient will be monitored regularly. If the colposcopy findings are positive, further diagnostic tests such as endocervical curettage (ECC) | D | D | D |
| HuatuoGPT (With prompt) | 55. If a pregnant patient has a diagnosis of HSIL confirmed by histopathologic examination (biopsy) during pregnancy, but does not have invasive cancer during pregnancy, what further steps should be performed next after the child is born? Ans: Regular pap smear monitoring is required. | D | C | D |
| HuatuoGPT | If a pregnant woman is free of invasive cancer during pregnancy and has a pap smear after giving birth, the following follow-up steps should be performed: a. Perform regular pap smears and colposcopies to monitor the progression or disappearance of the lesion. b. Follow your doctor's recommendations for follow-up treatment and follow up. c. Pay attention to any physical changes or discomfort and report them to your doctor promptly. d. Consider HPV vaccination to prevent future HPV infection. | B | B | B |
| BioMedLM 2.7B (With prompt) | 55. In cervical cancer screening, if a pregnant patient has a histopathological confirmation (biopsy) of HSIL with no invasive cancer during pregnancy, what follow-up steps should be conducted after childbearing? The patient should be followed up with colposcopy and biopsy. | C | C | C |
| BioMedLM 2.7B | The patient should undergo colposcopy and biopsy at 6 months after delivery to confirm the absence of invasive cancer or high grade squamous intraepithelial lesion (HSIL). If there are no abnormalities on colposcopic examination, then the patient can return for annual Pap smear testing until the age of 65 years old. If there are abnormalities in the Pap test, colposcopy with biopsy must be performed immediately. | A | A | A |
| Microsoft Phi2 (With prompt) | 55.In cervical cancer screening, if a pregnant patient has a histopathological confirmation (biopsy) of HSIL with no invasive cancer during pregnancy, the patient should be referred to a gynecologic oncologist for further evaluation and management. The patient should undergo a repeat Pap smear and HPV testing at 6 months post-biopsy. If the results of the repeat Pap smear and HPV testing are negative, the patient can be followed up with a Pap smear every 3 years. If the results of the repeat Pap smear and HPV testing are positive, the patient should undergo further evaluation and management, which may include colposcopy, biopsy, and treatment. | C | C | C |
| Microsoft Phi2 | If a pregnant patient has a histopathological confirmation of HSIL, and no invasive cancer during pregnancy, after childbearing, follow-up steps should include a repeat Pap test in 6 months and a colposcopy in 1 year. | B | A | A |
| QiZhenGPT (With prompt) | 55. In cervical cancer screening, if a pregnant patient has a histopathological confirmation (biopsy) of HSIL with no invasive cancer during pregnancy, what follow-up steps should be conducted after childbearing? Answer: Follow-up with a routine cervical cancer screening test (CCT) to detect any changes in the status of the patient's cervical cancer screening results.  a. Follow-up with a routine cervical cancer screening test (CCT) to detect any changes in the status of the patient's cervical cancer screening results after pregnancy.  b. Follow-up with a routine cervical cancer screening test (CCT) to detect any changes in the status of the patient's cervical cancer screening results after childbearing.  c. Follow-up with a routine cervical cancer screening test (CCT) to detect any changes in the status of the patient's cervical cancer screening results after delivery.  d. Follow-up with a routine cervical cancer screening test (CCT) to detect any changes in the status of the patient's cervical cancer screening results after any subsequent pregnancy or childbirth. | C | C | C |
| QiZhenGPT | if a pregnant woman has pathologically confirmed (biopsy) HSIL during pregnancy testing and no invasive cancer was found during pregnancy and is followed up after delivery, the following follow-up steps should be performed: * Perform cytologic follow-up, including cervical cytology (PAP smear) every 3-6 months. * If follow-up shows persistent abnormalities, further investigations such as cervical biopsy may be required. | A | B | A |
| **56. What are the strategies for HPV vaccine use? What are the recommended vaccination programs for different age groups?** | | | | |
| ChatGPT4.0 turbo (With prompt) | 56. **HPV Vaccine Use and Recommended Programs**: The primary strategy for HPV vaccine use is to prevent cervical cancer and other HPV-related diseases. The current recommendation from the World Health Organization (WHO) and the Centers for Disease Control and Prevention (CDC) is to administer the HPV vaccine to girls and boys starting at ages 11 or 12, although it can be administered as early as age 9. Catch-up vaccination is advised for males and females through age 26 if they were not vaccinated earlier. For individuals aged 27 through 45, vaccination decisions should be based on a shared clinical decision-making process, recognizing that persons in this age group are less likely to benefit from vaccination. The vaccines are most effective when administered prior to exposure to HPV, hence the focus on early vaccination of pre-teens and adolescents. | A | A | A |
| ChatGPT4.0 turbo | 56. Strategies for HPV Vaccine Use and Recommended Vaccination Programs for Different Age Groups The strategies for HPV (Human Papillomavirus) vaccine use primarily focus on preventing cervical cancer, other anogenital cancers, and genital warts. The recommended vaccination programs vary by country but generally include:  Age Groups: The HPV vaccine is most effective when given before exposure to HPV. Therefore, it is typically recommended for preteens aged 11 to 12 years, but it can be administered as early as age 9.  Catchup Vaccinations: For those not vaccinated at the recommended early age, catchup vaccinations are advised. In the United States, for example, catchup vaccination is recommended up to age 26. The World Health Organization (WHO) suggests considering local epidemiology and costeffectiveness when deciding on the upper age limit for catchup.  Gender: Initially targeted primarily at girls and young women, the recommendation has expanded to include boys and young men to prevent the transmission of HPV and reduce the incidence of related cancers.  Dosing Schedule: Typically, the HPV vaccine is administered in two or three doses, depending on the age at initial vaccination. Those who start the series before their 15th birthday usually receive two doses, while those who start later receive three doses. | A | A | A |
| Claude 2 (With prompt) | 56. The strategies for HPV vaccination are routine vaccination at ages 11-12 years with catch-up vaccination through age 26. A 2-dose series is recommended for most adolescents under age 15, while a 3-dose series is recommended for older adolescents and young adults ages 15-26 years. | B | A | A |
| Claude 2 | 56. The HPV vaccine is recommended for all children ages 11-12 years old. It is also recommended for females ages 13-26 and males ages 13-21 who have not been previously vaccinated. Catch-up vaccination is recommended up to age 26 for both males and females. The vaccine is given in 2 or 3 doses depending on age at initial vaccination. | A | A | A |
| Gemini Pro (With prompt) | 56. Strategies for HPV Vaccine Use and Recommended Vaccination Programs for Different Age Groups   Strategies for HPV Vaccine Use:  Universal vaccination of all preadolescent girls and boys  Catch-up vaccination for older adolescents and young adults who missed earlier vaccination  Targeted vaccination of high-risk populations, such as men who have sex with men (MSM) and immunocompromised individuals   Recommended Vaccination Programs for Different Age Groups:  United States:  Routine vaccination for girls and boys aged 11-12 years  Catch-up vaccination for individuals aged 13-26 years who have not been previously vaccinated  World Health Organization (WHO):  Routine vaccination for girls aged 9-14 years  Catch-up vaccination for girls aged 15-26 years | A | A | A |
| Gemini Pro | 56. **Strategies for HPV vaccine use** * **Universal vaccination:** All girls and boys are vaccinated, regardless of their sexual orientation or activity. This is the most effective way to prevent HPV infection and cervical cancer. * **Targeted vaccination:** Vaccination is targeted to specific groups of people who are at high risk for HPV infection, such as sexually active women and men who have sex with men. * **Catch-up vaccination:** Vaccination is offered to people who missed out on vaccination as children or adolescents. **Recommended vaccination programs for different age groups** * **Girls:** The HPV vaccine is recommended for all girls aged 11-12 years. A catch-up vaccination program is recommended for girls aged 13-26 years who have not been vaccinated. * **Boys:** The HPV vaccine is recommended for all boys aged 11-12 years. A catch-up vaccination program is recommended for boys aged 13-26 years who have not been vaccinated. | B | B | B |
| Mistral-7B-v0.2 (With prompt) | 56.The HPV vaccine is recommended for girls and women between the ages of 9 and 26. The Centers for Disease Control and Prevention (CDC) recommends three doses of the HPV vaccine for girls and women aged 11-12, with catch-up vaccination for those up to age 26. The vaccine can also be given to boys and men to prevent anal and throat cancers caused by HPV. | A | A | A |
| Mistral-7B-v0.2 | 56. HPV vaccine use strategies and recommended vaccination programs for different age groups: Routine HPV vaccination programs: HPV vaccination is administered to girls and young women (ages 11-12 or 9-26, depending on the country) to prevent new HPV infections. In some countries, routine vaccination is also offered to boys and young men. Catch-up vaccination: This approach targets older populations (girls and women up to 26 years old, and boys and men up to 21 years old, depending on the country) who have not yet received the HPV vaccine, with the goal of achieving herd immunity and protecting more people from the virus. | B | B | B |
| Starling-LM-7B alpha (With prompt) | 56. The strategies for HPV vaccine use primarily involve the implementation of vaccination programs targeting specific age groups. The recommended vaccination programs for different age groups are as follows:   a. For females: The HPV vaccine is typically administered to girls aged 9-13 years, although it can be given as early as age 9 and as late as age 26. The vaccine is usually given in a series of two or three doses, depending on the age at which it is initiated.   b. For males: The HPV vaccine is typically administered to boys aged 9-13 years, although it can be given as early as age 9 and as late as age 21. The vaccine is usually given in a series of two or three doses, depending on the age at which it is initiated. | B | B | B |
| Starling-LM-7B alpha | 56. Strategies for HPV vaccine use and recommended vaccination programs for different age groups: The HPV vaccine is typically administered as a series of injections, with the number of doses and the age at which vaccination should begin depending on the specific vaccine and the target population. Here are some general guidelines for HPV vaccine use and recommended vaccination programs for different age groups: - Routine vaccination: The HPV vaccine is usually given to girls and boys aged 11-12 years, with catch-up vaccination for those aged 13-26 years. In some countries, vaccination is also offered to boys aged 11-12 years. - Adolescent catch-up: For those who start the vaccination series after their 13th birthday, it is recommended to administer three doses at 0, 1-2, and 6 months (or 0, 2, and 6 months for the bivalent vaccine). - Adult catch-up: For adults aged 15-26 years who have not been previously vaccinated or have received only one dose, it is recommended to administer two doses at 0 and 6 months (or 0 and 2 months for the bivalent vaccine). | B | B | B |
| HuatuoGPT (With prompt) | 56. What is the strategy for HPV vaccine use? What vaccine programs should be recommended for different age groups? HPV vaccine use can prevent cervical cancer by reducing exposure to high-risk types of HPV. The World Health Organization (WHO) recommends the following HPV vaccine schedule: Girls aged 9-14 years: 4 doses (months 0, 1, 6 and 12) Girls and young women 15 years and older: 3 doses (months 0, 1, 6) Males 15 years and older: 2 doses (months 0, 12) | B | B | B |
| HuatuoGPT | 56. For HPV vaccine utilization strategies, it is usually recommended to follow the following: Give two doses of HPV vaccine to children aged 11 to 12 years. Vaccination may be considered for both males and females between the ages of 9 and 26 years. Three doses of HPV vaccine are recommended for all schoolgirls between the ages of 13 and 18. The Centers for Disease Control and Prevention (CDC) recommends two doses of HPV vaccine for girls ages 11 to 12. The World Health Organization (WHO) recommends three doses of HPV vaccine for girls ages 11 to 18. Effective HPV vaccines are used for the nine-valent vaccine (Gardasil) and the four-valent vaccine (Cervarix). | C | C | C |
| BioMedLM 2.7B (With prompt) | 56. What are the strategies for HPV vaccine use? What are the recommended vaccination programs for different age groups? The HPV vaccine is a safe and effective vaccine that can prevent cervical cancer and genital warts. The Centers for Disease Control and Prevention (CDC) recommends routine vaccination of girls and boys aged 11 to 12 years.^[@bibr1-2325967119885314]^ The American Academy of Pediatrics (AAP) recommends routine vaccination of girls aged 11 to 12 years. | C | C | C |
| BioMedLM 2.7B | 56. What are the strategies for HPV vaccine use? What are the recommended vaccination programs for different age groups? ### Response: The HPV vaccine is recommended for females aged 9--26 years. | C | C | C |
| Microsoft Phi2 (With prompt) | 56. The strategies for HPV vaccine use include vaccination of both males and females, vaccination of high-risk populations, and vaccination of individuals who have not been vaccinated before. The recommended vaccination programs for different age groups include vaccination of girls and boys aged 9-14 years, vaccination of girls aged 15-26 years, and vaccination of women aged 27-45 years who have not been vaccinated before. | B | C | C |
| Microsoft Phi2 | 56. The strategies for HPV vaccine use include vaccination of both males and females, vaccination of adolescents, and vaccination of adults. The recommended vaccination programs for different age groups are as follows: - Adolescents: The HPV vaccine is recommended for both males and females aged 11-12 years. The vaccine is given in two doses, six months apart. - Adults: The HPV vaccine is recommended for both males and females aged 13-26 years. The vaccine is given in two doses, six months apart. - Pregnant women: The HPV vaccine is recommended for pregnant women aged 27-45 years. The vaccine is given in three doses, two months apart. | B | B | B |
| QiZhenGPT (With prompt) | 56. Strategies for HPV vaccine use: * Recommendation: All children and adolescents aged 12-18 should be vaccinated with the HPV 16 and 18 vaccine.  * Recommendation for adults: HPV 6 and 11 vaccines should be recommended for those aged 26-65 years.  * Recommendation for high-risk HPV vaccines: HPV 16 and 18 vaccines should be recommended for individuals with a history of low-risk HPV infection or who have had a previous HPV vaccine series.  * Recommendation for HPV 31, 33, 35, 45, and 52 vaccines: These vaccines should be recommended for individuals aged 26-65 years with a history of low-risk HPV infection or who have had a previous HPV vaccine series. | B | A | A |
| QiZhenGPT | 56. Strategies for HPV vaccine use include providing education and counseling to increase vaccination rates, offering incentives for healthcare providers and community leaders, and implementing policies and laws that support vaccination. Recommended vaccination programs for different age groups include the primary series of 3 doses for children under 15 years, 2 doses for adults aged 15-65, and 3 doses for adults aged 65 or older. | C | C | C |
| **57. Is HPV primary screening applicable in low-income countries? If so, why?** | | | | |
| ChatGPT4.0 turbo (With prompt) | 57. **HPV Primary Screening in Low-Income Countries**: HPV primary screening is indeed applicable and recommended in low-income countries, particularly because it can be conducted less frequently than cytology-based screening and still provide high sensitivity for detecting high-grade cervical lesions. The WHO recommends HPV testing as the primary screening method for cervical cancer due to its higher sensitivity and longer screening intervals which can reduce costs and logistical burdens associated with more frequent screening methods. Additionally, the use of HPV testing can be combined with visual inspection with acetic acid (VIA) to triage positive cases, which is feasible in low-resource settings. | A | A | A |
| ChatGPT4.0 turbo | 57. HPV Primary Screening in LowIncome Countries HPV primary screening is increasingly seen as applicable in lowincome countries due to its potential to be more costeffective and logistically feasible than traditional cytologybased screening. It requires fewer resources in terms of infrastructure and training. HPV testing can also be done with selfcollected samples, which can increase screening coverage and compliance among women living in remote areas. | A | A | A |
| Claude 2 (With prompt) | 57. Yes, primary HPV screening is likely applicable in low-income countries. HPV testing is more sensitive than cytology, allows for safe lengthening of screening intervals, and enables screening initiation at older ages. This makes it suitable for regions with limited health infrastructure. However, quality management systems and capacity to treat detected lesions are still essential. | A | A | A |
| Claude 2 | 57. Primary HPV screening may be applicable in low-income countries, especially when resources for cytology-based screening are limited. HPV testing is more sensitive than cytology for detecting cervical precancer. Additionally, HPV testing provides the opportunity for self-collection, which may improve screening coverage in settings where access to clinics is limited. However, HPV screening requires more infrastructure and follow-up compared to visual inspection methods. More evidence on the feasibility, cost-effectiveness and health impact of HPV primary screening in low-resource settings is still needed. | A | A | A |
| Gemini Pro (With prompt) | 57. Applicability of HPV Primary Screening in Low-Income Countries   Yes, HPV primary screening is applicable in low-income countries.  Reasons:  HPV is the primary cause of cervical cancer worldwide.  HPV testing is highly sensitive and specific for detecting cervical precancerous lesions.  HPV primary screening is more cost-effective than traditional cytology-based screening in low-resource settings. | A | A | A |
| Gemini Pro | 57. **HPV primary screening is applicable in low-income countries** Yes, HPV primary screening is applicable in low-income countries because it is: * **More sensitive than cytology:** HPV primary screening is more sensitive than cytology for detecting precancerous lesions and cervical cancer. This means that it can identify more women who need treatment. * **More specific than cytology:** HPV primary screening is more specific than cytology for detecting precancerous lesions and cervical cancer. This means that it can reduce the number of unnecessary referrals to colposcopy. * **Less expensive than cytology:** HPV primary screening is less expensive than cytology, making it more affordable for low-income countries. | A | A | A |
| Mistral-7B-v0.2 (With prompt) | 57.HPV primary screening is applicable in low-income countries, particularly in those with a high burden of cervical cancer. The use of HPV primary screening can reduce the number of false positives and false negatives compared to traditional cytology screening. This can lead to earlier detection and treatment of cervical cancer, particularly in resource-limited settings where access to follow-up care is limited. | A | A | A |
| Mistral-7B-v0.2 | 57. HPV primary screening applicability in low-income countries: Yes, HPV primary screening is applicable in low-income countries. Using HPV testing as the primary screening method offers multiple advantages over traditional cytology: Greater sensitivity (detects more precancerous lesions). Reduced need for laboratories and specialized personnel, as well as fewer equipment requirements. Lower rates of false-positive results and unnecessary invasive procedures. Improved patient acceptance due to less invasive nature of the test. Effective at preventing progression of infection to cervical cancer, leading to better disease management and outcomes. | A | A | A |
| Starling-LM-7B alpha (With prompt) | 57. HPV primary screening is applicable in low-income countries, particularly in sub-Saharan Africa, due to the high prevalence of HPV infections and the high incidence of cervical cancer in these regions. HPV primary screening has been shown to be more sensitive and specific than traditional cytology-based screening methods, such as the Pap smear test. This increased sensitivity and specificity can lead to earlier detection of high-risk HPV infections and, consequently, a reduction in the incidence of cervical cancer. | A | A | A |
| Starling-LM-7B alpha | 57. HPV primary screening in low-income countries: HPV primary screening is applicable in low-income countries, especially in regions with a high burden of cervical cancer. The main reasons for its applicability include: - Cost-effectiveness: HPV testing is a cost-effective method for cervical cancer screening, as it can detect precancerous lesions at an early stage, reducing the need for more invasive and expensive diagnostic procedures. - Accessibility: HPV testing is less invasive and more acceptable to women than traditional cytology-based screening methods, making it more accessible in low-resource settings. - Early detection: HPV primary screening allows for the early detection of high-risk HPV types, which are responsible for the majority of cervical cancers, enabling timely intervention and treatment. | A | A | A |
| HuatuoGPT (With prompt) | 57. Is primary HPV screening feasible in low-income countries? If so, why? Primary screening for HPV is feasible in low-income countries, but it may be difficult to implement a mass screening program due to limited resources. However, cervical cancer screening in these countries can help in the early detection and treatment of cervical cancer, thereby reducing mortality. | A | A | A |
| HuatuoGPT | 57. Primary screening for HPV is feasible in low-income countries. This is because primary screening is a relatively low-cost screening method that can help to identify women with pre-cancerous cervical lesions early enough for timely treatment. In addition, primary screening can raise public awareness of cervical cancer and its prevention, laying the foundation for future cervical cancer control programs. | A | A | A |
| BioMedLM 2.7B (With prompt) | 57. Is HPV primary screening applicable in low-income countries? If so, why? The answer to this question is a complex one. The answer depends on the prevalence of HPV infection in the population, the sensitivity and specificity of the HPV test, and the cost of the HPV test. In low-income countries, the prevalence of HPV infection is high, and the sensitivity and specificity of the HPV test are low. The cost of the HPV test is also high. The cost of the HPV test is a major barrier to the implementation of HPV primary screening in low-income countries. | A | A | A |
| BioMedLM 2.7B | 57. Is HPV primary screening applicable in low-income countries? If so, why? ### Response: The answer is that it is applicable in low-income countries. The reason is that the cost of HPV testing is much lower than that of cytology. | A | A | A |
| Microsoft Phi2 (With prompt) | 57. HPV primary screening is not applicable in low-income countries due to the lack of resources and infrastructure required for the screening program. | D | D | D |
| Microsoft Phi2 | 57. HPV primary screening is not applicable in low-income countries due to several reasons. Firstly, the cost of the screening test is high, which makes it unaffordable for most people in low-income countries. Secondly, the infrastructure required for the screening test is not available in most low-income countries. Thirdly, the training required for the healthcare workers to perform the screening test is not available in most low-income countries. | D | D | D |
| QiZhenGPT (With prompt) | 57. Is HPV primary screening applicable in low-income countries? If so, why? * Recommendation: HPV primary screening should be considered in low-income countries where access to advanced cervical cancer screening methods is limited.  * Reason: HPV primary screening can provide early detection of cervical cancer and improve survival rates. | A | A | A |
| QiZhenGPT | 57. HPV primary screening is not applicable in low-income countries because it is expensive and requires significant resources. | D | D | D |
| **58. which cervical cancer screening methods are widely used in low-income countries, particularly in sub-Saharan Africa?** | | | | |
| ChatGPT4.0 turbo (With prompt) | 58. **Cervical Cancer Screening Methods in Low-Income Countries**: In many low-income countries, particularly in sub-Saharan Africa, cervical cancer screening methods commonly include visual inspection with acetic acid (VIA) and HPV DNA testing where feasible. VIA is cost-effective and results are immediately available, making it suitable for settings with limited healthcare infrastructure. HPV testing, although more expensive, is increasingly used where resources allow, due to its higher sensitivity and the potential for longer intervals between screenings. | A | A | A |
| ChatGPT4.0 turbo | 58. Cervical Cancer Screening Methods in LowIncome Countries, Particularly in SubSaharan Africa In lowincome countries, particularly in subSaharan Africa, the most widely used cervical cancer screening methods include:  Visual Inspection with Acetic Acid (VIA): This method involves applying acetic acid to the cervix and visually inspecting it for abnormalities. VIA is inexpensive and results are available immediately, but it requires trained personnel and has variable sensitivity and specificity.  HPV DNA Testing: Where available, HPV testing is becoming more popular due to its higher sensitivity for detecting precancerous lesions. However, the cost and infrastructure requirements can be prohibitive.  Pap Smear: Although effective, the Pap smear is less commonly used in lowresource settings due to the need for laboratory infrastructure and trained cytologists. | A | A | A |
| Claude 2 (With prompt) | 58. Visual inspection with acetic acid (VIA) and careHPV testing when available are currently the predominant cervical cancer screening methods in low-income sub-Saharan African countries. Cytology capacity remains scarce unfortunately. | A | A | A |
| Claude 2 | 58. Visual inspection methods are the mainstay of cervical cancer screening in low-income countries in sub-Saharan Africa. The most commonly used methods are visual inspection with acetic acid (VIA) and visual inspection with Lugol's iodine (VILI). These allow screening and treatment of precancerous lesions in a single visit (screen-and-treat approach). Other methods such as cytology testing and HPV testing require more infrastructure and are less feasible in low-resource settings currently. Efforts are ongoing to evaluate and implement HPV testing and cytology where resources allow. | A | A | A |
| Gemini Pro (With prompt) | 58. Widely Used Cervical Cancer Screening Methods in Low-Income Countries, Particularly in Sub-Saharan Africa   Visual inspection with acetic acid (VIA)  Visual inspection with Lugol's iodine (VILI)  Pap smear cytology (less commonly used due to resource constraints) | A | A | A |
| Gemini Pro | 58. **Cervical cancer screening methods widely used in low-income countries** The most widely used cervical cancer screening methods in low-income countries are: * **Visual inspection with acetic acid (VIA)**: VIA is a simple and inexpensive method that involves applying acetic acid to the cervix and looking for changes in the color of the tissue. * **Visual inspection with Lugol's iodine (VILI)**: VILI is similar to VIA, but it uses Lugol's iodine instead of acetic acid. * **Pap smear:** The Pap smear is a more sensitive method than VIA or VILI, but it is also more expensive and requires more training to perform. | A | A | A |
| Mistral-7B-v0.2 (With prompt) | 58.In low-income countries, particularly in sub-Saharan Africa, cervical cancer screening methods that are widely used include visual inspection after acetic acid application (VIA) and cytology. VIA is a simple and inexpensive method that involves applying acetic acid to the cervix to make abnormal areas appear white. Cytology involves taking a sample of cells from the cervix and examining them under a microscope for signs of cancer or precancer. | A | A | A |
| Mistral-7B-v0.2 | 58. Cervical cancer screening methods used in low-income countries, particularly in Sub-Saharan Africa: Visual Inspection after Acetic Acid (VIA): This screening method involves inspecting the cervix after it has been treated with a diluted acetic acid solution, which can help identify precancerous lesions (called acetowhite lesions). VIA is inexpensive, easy to perform, and does not require specialized equipment, making it preferable for resource-limited settings. Pap testing: Liquid-based or conventional Pap tests are still used to screen for cervical cancer in some low-income countries, despite their limitations and invasive nature. This method tests for abnormal cell growth on the cervix and has a lower sensitivity at an earlier stage compared to HPV testing. | A | A | A |
| Starling-LM-7B alpha (With prompt) | 58. Cervical cancer screening methods widely used in low-income countries, particularly in sub-Saharan Africa, include:   a. Visual Inspection with Acetic Acid (VIA): This method involves the application of acetic acid to the cervix, which causes the transformation of abnormal cells into white or gray lesions that can be easily visualized. VIA is a low-cost, non-invasive, and relatively simple method that can be performed by non-specialist healthcare providers.   b. Visual Inspection with Lugol's iodine (VILI): This method involves the application of Lugol's iodine to the cervix, which selectively stains normal cells while leaving abnormal cells unstained. VILI is another low-cost, non-invasive, and relatively simple method that can be performed by non-specialist healthcare providers.   c. ThinPrep Liquid-Based Cytology (TBLBC): This method involves the collection of cervical cells using a spatula or a cytobrush, which are then suspended in a liquid medium. The liquid medium helps to separate and concentrate the cells, which can then be analyzed under a microscope for the presence of abnormal cells. TBLBC is a more sensitive and specific method than traditional Pap smear tests but requires laboratory equipment and trained personnel for analysis. | A | A | A |
| Starling-LM-7B alpha | 58. Cervical cancer screening methods in low-income countries, particularly in sub-Saharan Africa: In low-income countries, particularly in sub-Saharan Africa, several cervical cancer screening methods are widely used, including: - Visual inspection with acetic acid (VIA): This method involves applying acetic acid to the cervix, which causes precancerous and cancerous lesions to turn white. VIA is a low-cost, non-invasive, and easy-to-use method that can be performed by non-specialist health workers. - Visual inspection with Lugol's iodine (VILI): Similar to VIA, VILI involves the application of Lugol's iodine to the cervix, which stains normal cells but not precancerous or cancerous cells. This method can also be performed by non-specialist health workers and is relatively low-cost. - Self-sampling for HPV testing: This method involves women collecting their own cervical samples using a swab or brush, which are then sent to a laboratory for HPV testing. Self-sampling is a non-invasive, acceptable, and cost-effective method that can be performed in the community setting. | A | A | A |
| HuatuoGPT (With prompt) | 58. What cervical cancer screening methods are widely used in low-income countries, especially in sub-Saharan Africa? A widely used cervical cancer screening method in low-income countries, especially in sub-Saharan Africa, is the smear test. It is a simple and cost-effective screening method for the early detection of CIN, a pre-existing lesion for the development of cervical cancer. | D | C | C |
| HuatuoGPT | 58. Cervical cancer screening methods commonly used in low-income countries, especially in sub-Saharan Africa, include: Visual inspection with acetic acid (VIA): a simple, low-cost and easy-to-perform screening method. Cervical cytology (Pap smear): a widely used cervical cancer screening method, but requires more complex equipment and techniques. | A | A | A |
| BioMedLM 2.7B (With prompt) | 58. which cervical cancer screening methods are widely used in low-income countries, particularly in sub-Saharan Africa? The answer is "cytology." Cytology is the most widely used cervical cancer screening method in low-income countries. In sub-Saharan Africa, cytology is used in most countries, and it is the only screening method used in some countries. | D | D | D |
| BioMedLM 2.7B | 58. which cervical cancer screening methods are widely used in low-income countries, particularly in sub-Saharan Africa? ### Response: The following is a list of cervical cancer screening methods that are widely used in low-income countries, particularly in sub-Saharan Africa.  Visual inspection with acetic acid (VIA)  Visual inspection with Lugol's iodine (VILI)  Visual inspection with acetic acid and Lugol's iodine (VIA/VILI). Visual inspection with Lugol's iodine and colposcopy (VILI/COL) Visual inspection with acetic acid and colposcopy (VIA/COL) Visual inspection with acetic acid and Lugol's iodine and colposcopy (VILI/COL) | A | A | A |
| Microsoft Phi2 (With prompt) | 58. The key cervical cancer screening methods used in low-income countries, particularly in sub-Saharan Africa, include visual inspection with acetic acid (VIA) and liquid-based cytology (LBC). These methods are cost-effective and can be performed by trained health workers in primary care settings. | A | A | A |
| Microsoft Phi2 | 58. The key cervical cancer screening methods widely used in low-income countries, particularly in sub-Saharan Africa, are visual inspection with acetic acid (VIA) and HPV testing. | A | A | A |
| QiZhenGPT (With prompt) | 58. Which cervical cancer screening methods are widely used in low-income countries, particularly in sub-Saharan Africa? * HPV DNA testing: This method is widely used in low-income countries, particularly in sub-Saharan Africa.  * HPV RNA testing: This method is also widely used in low-income countries, particularly in sub-Saharan Africa. | D | D | D |
| QiZhenGPT | 58.Cervical cancer screening methods that are widely used in low-income countries, particularly in sub-Saharan Africa, include visual inspection, acetic acid solution, and Betadine solution. | A | A | A |
| **59. What are the key indicators included in the expert consensus for quality control management of HPV testing?** | | | | |
| ChatGPT4.0 turbo (With prompt) | 59. **Quality Control Management of HPV Testing**: Key indicators in the expert consensus for quality control management of HPV testing include ensuring the accuracy and reliability of test results through regular calibration and maintenance of equipment, proper training and certification of laboratory personnel, and adherence to standardized operating procedures. Additionally, quality control measures should include regular internal checks and participation in external quality assessment (EQA) schemes to benchmark performance against other laboratories. Documentation of all procedures and results is also critical for maintaining high standards of quality control. | B | B | B |
| ChatGPT4.0 turbo | 59. Key Indicators in the Expert Consensus for Quality Control Management of HPV Testing The expert consensus for quality control management of HPV testing typically includes the following key indicators:  Test Sensitivity and Specificity: Ensuring the test accurately identifies HPV infections and does not give false negatives or positives.  Quality Assurance of Laboratory Procedures: Regular monitoring and auditing of the procedures used in the laboratory to ensure consistent standards.  Calibration and Maintenance of Equipment: Regular checks and maintenance of the equipment used for HPV testing.  Training and Proficiency of Personnel: Ensuring that all personnel involved in HPV testing are adequately trained and maintain proficiency through continuous education and evaluation. | A | A | A |
| Claude 2 (With prompt) | 59. Key quality indicators for HPV testing proposed in expert consensus guidance include monitoring sample adequacy, assay reliability, clinician sampling proficiency, laboratory certification/accreditation status, and systems to ensure patient tracking and follow-up care for abnormalities. | A | A | A |
| Claude 2 | 59. Key quality control indicators for HPV testing proposed by an international expert consensus include:  - Sample adequacy rate - HPV positivity rate  - Invalid result rate - Reproducibility (inter- and intra-laboratory agreement) - Genotyping concordance - Clinically relevant HPV persistence rate | A | A | A |
| Gemini Pro (With prompt) | 59. Key Indicators Included in the Expert Consensus for Quality Control Management of HPV Testing   Sensitivity and specificity of the HPV test  Positive predictive value (PPV) and negative predictive value (NPV)  Laboratory proficiency and accreditation  Adequate specimen collection and handling  Regular external quality assurance (EQA) programs | A | A | A |
| Gemini Pro | 59. **Key indicators included in the expert consensus for quality control management of HPV testing** The key indicators included in the expert consensus for quality control management of HPV testing are: * **Analytical sensitivity:** The ability of the test to detect HPV infection. * **Analytical specificity:** The ability of the test to correctly identify people who do not have HPV infection. * **Clinical sensitivity:** The ability of the test to detect precancerous lesions and cervical cancer. * **Clinical specificity:** The ability of the test to correctly identify people who do not have precancerous lesions or cervical cancer. * **Reproducibility:** The ability of the test to produce the same results when it is performed multiple times. | B | B | B |
| Mistral-7B-v0.2 (With prompt) | 59.The key indicators included in the expert consensus for quality control management of HPV testing include:a) Pre-analytical factors: proper sample collection, transport, and storage. b) Analytical factors: accuracy, precision, and reproducibility of the HPV test. c) Post-analytical factors: timely reporting of results, communication of results to the patient, and appropriate follow-up care. | A | A | A |
| Mistral-7B-v0.2 | 59. Key indicators included in the expert consensus for quality control management of HPV testing: Preanalytical: Proper collection, transport, and storage conditions for HPV test samples. Analytical: Laboratory methods and equipment used for performing and interpreting HPV tests, as well as standardization and calibration processes. Post-analytical: Result reporting and implementation of appropriate follow-up procedures based on test results. Total turnaround time: The time that elapses between collecting a sample, performing the HPV test, obtaining the results, and initiating appropriate downstream actions. Personnel training: Ensuring that laboratory staff are adequately trained to perform the HPV tests and interpret the results accurately. | A | A | A |
| Starling-LM-7B alpha (With prompt) | 59. The key indicators included in the expert consensus for quality control management of HPV testing are:   a. Test sensitivity and specificity: The ability of the HPV test to accurately identify high-risk HPV infections and distinguish them from low-risk or non-cancerous infections.   b. Test reproducibility and reliability: The consistency of test results across different laboratories, operators, and testing platforms.   c. Test performance in different populations: The ability of the HPV test to accurately identify high-risk HPV infections in different age groups, ethnicities, and geographic regions.   d. Test cost-effectiveness: The cost of the HPV test in relation to the potential benefits of early detection and treatment of cervical cancer. | A | A | A |
| Starling-LM-7B alpha | 59. Key indicators for quality control management of HPV testing: The expert consensus for quality control management of HPV testing includes several key indicators, such as: - Test sensitivity and specificity: The ability of the HPV test to accurately detect high-risk HPV types and differentiate between positive and negative results. - Test reproducibility: The consistency of test results when the same sample is tested multiple times or by different laboratories. - Test accuracy: The degree to which the HPV test correctly identifies women with and without cervical lesions. - Test performance in different populations: The ability of the HPV test to accurately detect high-risk HPV types in different age groups, ethnicities, and geographic regions. - Test cost-effectiveness: The cost of the HPV test in relation to its ability to detect and prevent cervical cancer. | B | A | A |
| HuatuoGPT (With prompt) | 59. What are the key indicators of quality-controlled managed HPV testing included in the expert consensus? The key indicators of HPV testing for quality control management include sensitivity and specificity. Sensitivity is the ability to correctly identify a true positive (having cancer), while specificity is the ability to correctly identify a true negative (not having cancer). Expert consensus suggests that HPV testing should have a sensitivity of at least 80% and a specificity of 80%. | A | A | A |
| HuatuoGPT | 59. The following key indicators are included in the quality control management of HPV testing: Sensitivity (Sensitivity) and Specificity (Specificity) of the assay: a measure of the accuracy of HPV testing. Positive Predictive Value (Positive Predictive Value) and Negative Predictive Value (Negative Predictive Value): measures the degree of association between HPV test results and cervical cancer. Repeatability and Reproducibility: The requirement that HPV tests have consistent results across laboratories and between operators. | A | A | A |
| BioMedLM 2.7B (With prompt) | 59. what are the key indicators included in the expert consensus for quality control management of HPV testing? The key indicators included in the expert consensus for quality control management of HPV testing are listed in Table [1](#T1){ref-type="table"}. | B | B | B |
| BioMedLM 2.7B | 59. what are the key indicators included in the expert consensus for quality control management of HPV testing? ### Response: The key indicators included in the expert consensus for quality control management of HPV testing are listed in [Table 1](#T1){ref-type="table"}. | D | D | D |
| Microsoft Phi2 (With prompt) | 59. The key indicators included in the expert consensus for quality control management of HPV testing are the sensitivity, specificity, positive predictive value, and negative predictive value of the test. These indicators are important for ensuring the accuracy and reliability of the test results. | B | B | B |
| Microsoft Phi2 | 59. The key indicators included in the expert consensus for quality control management of HPV testing are as follows: - The use of a standardized protocol for sample collection, processing, and storage. - The use of a validated HPV testing method. - The use of a quality control system to monitor the performance of the HPV testing method. - The use of a quality assurance system to ensure that the HPV testing method is being performed correctly. | A | A | A |
| QiZhenGPT (With prompt) | 59. What are the key indicators included in the expert consensus for quality control management of HPV testing? * Key indicators for quality control management of HPV testing include: + Accessibility and convenience of testing + Timeliness of results + Accuracy of results + Timeliness of follow-up care + Availability of support services for patients | B | B | B |
| QiZhenGPT | 59. Key indicators for quality control management of HPV testing include test performance, operator training, and quality control procedures. | B | C | C |
| **60.What is the difference in clinical management between ASC-US and ASC-H?** | | | | |
| ChatGPT4.0 turbo (With prompt) | 60. **Clinical Management Differences between ASC-US and ASC-H**: The clinical management of atypical squamous cells of undetermined significance (ASC-US) and atypical squamous cells – cannot exclude HSIL (ASC-H) differs significantly. For ASC-US, management may include repeat cytology testing in 6 to 12 months, immediate HPV testing, or colposcopy, depending on factors such as age and HPV vaccination status. If HPV testing is negative, routine screening is resumed. In contrast, ASC-H management typically involves immediate referral to colposcopy due to the higher risk of underlying high-grade lesions. The management of ASC-H does not usually depend on HPV test results because of the high-risk nature of this cytological finding. | A | A | A |
| ChatGPT4.0 turbo | 60. Difference in Clinical Management Between ASCUS and ASCH  ASCUS (Atypical Squamous Cells of Undetermined Significance): This is a mild abnormality found on Pap tests. Management may include repeat Pap testing in 6-12 months, immediate HPV testing to determine the necessity of colposcopy, or direct colposcopy depending on age and other risk factors.  ASCH (Atypical Squamous Cells, Cannot Exclude HSIL): This result suggests a higher possibility of highgrade lesions. Management typically involves immediate further evaluation with colposcopy and possibly biopsy, as the risk of underlying highgrade disease is significantly higher compared to ASCUS. These management guidelines can vary based on national or regional clinical protocols and the availability of resources. | A | A | A |
| Claude 2 (With prompt) | 60. ASC-US indicates minor cell abnormalities that may resolve without intervention, while ASC-H indicates more significant abnormalities warranting colposcopy referral given the considerably higher possibility of a concurrent high-grade precancerous lesion. Clinical management differs in that ASC-H abnormalities require more urgent diagnostic evaluation. | A | A | A |
| Claude 2 | 60. The key difference in clinical management between ASC-US (atypical squamous cells of undetermined significance) and ASC-H (atypical squamous cells, cannot exclude HSIL) cytology results is: - ASC-US: Repeat cytology at 12 months and/or HPV reflex testing. Referral to colposcopy if repeat abnormality or positive HPV. - ASC-H: Referral to colposcopy, with consideration of biopsy and treatment as indicated by colposcopy findings. ASC-H indicates a higher probability of a high-grade lesion. | A | A | A |
| Gemini Pro (With prompt) | 60. Difference in Clinical Management Between ASC-US and ASC-H   ASC-US (Atypical Squamous Cells of Undetermined Significance):  Repeat Pap smear in 6-12 months  Consider HPV testing if repeat Pap smear is abnormal  ASC-H (Atypical Squamous Cells, Cannot Exclude High-Grade Squamous Intraepithelial Lesion):  Colposcopy with biopsy  HPV testing may be used to guide management | C | C | C |
| Gemini Pro | 60. **Difference in clinical management between ASC-US and ASC-H** * **ASC-US (atypical squamous cells of undetermined significance):** ASC-US is a cytology result that indicates that the cells on the cervix are abnormal, but it is not clear whether the changes are due to HPV infection or another cause. Women with ASC-US are typically referred for colposcopy to rule out precancerous lesions. * **ASC-H (atypical squamous cells, cannot exclude high-grade squamous intraepithelial lesion):** ASC-H is a cytology result that indicates that the cells on the cervix are abnormal and that there is a high risk of precancerous lesions. Women with ASC-H are typically referred for colposcopy and biopsy to confirm the diagnosis. | C | B | C |
| Mistral-7B-v0.2 (With prompt) | 60.ASC-US (atypical squamous cells of undetermined significance) and ASC-H (atypical squamous cells cannot exclude high-grade squamous intraepithelial lesion or invasive carcinoma) are both abnormal cervical cytology findings. However, the clinical management of these two conditions differs. ASC-US is typically managed with repeat cytology testing or HPV testing. If HPV testing is positive, colposcopy and biopsy may be recommended. ASC-H is typically managed with colposcopy and biopsy, as it is a more severe abnormality that may indicate the presence of precancerous or cancerous lesions. | A | A | A |
| Mistral-7B-v0.2 | 60. Difference in clinical management between ASC-US (Atypical Squamous Cells of Undetermined Significance) and ASC-H (Atypical Squamous Cells that Cannot be Classified): ASC-US refers to abnormal cells that do not demonstrate clear signs of malignancy but may have the potential to develop into cervical cancer, requiring further testing to confirm the diagnosis. Patients with ASC-US are typically recommended for colposcopy or HPV testing to determine the extent and risk of the condition. ASC-H are severely abnormal cells that are difficult to classify. Although not cancerous themselves, they indicate the presence of precancerous lesions or invasive cervical cancer. Women with ASC-H are typically referred for a colposcopy and biopsy to further evaluate the affected areas and determine the appropriate course of treatment, which may include removing the precancerous or invasive tissue. | A | B | A |
| Starling-LM-7B alpha (With prompt) | 60. The difference in clinical management between ASC-US (Atypical Squamous Cells of Undetermined Significance) and ASC-H (Atypical Squamous Cells, Cannot Exclude High-Grade Squamous Intraepithelial Lesion) lies in the level of concern for the presence of high-grade lesions or cancer.   a. ASC-US: This diagnosis indicates the presence of atypical squamous cells, but it is not possible to determine whether these cells represent a low-grade or high-grade lesion. In the case of ASC-US, further testing, such as colposcopy and biopsy, may be recommended to rule out the presence of high-grade lesions or cancer. However, some guidelines may recommend a "watch and wait" approach for low-risk patients with ASC-US, particularly if the patient has a history of low-grade lesions.   b. ASC-H: This diagnosis indicates a higher level of concern for the presence of high-grade lesions or cancer, as the atypical squamous cells cannot be excluded from being a high-grade squamous intraepithelial lesion (HSIL) or worse. In the case of ASC-H, colposcopy and biopsy are typically recommended, and treatment may be initiated if high-grade lesions or cancer are confirmed. | A | A | A |
| Starling-LM-7B alpha | 60. Difference in clinical management between ASC-US and ASC-H: ASC-US (Atypical Squamous Cells of Undetermined Significance) and ASC-H (Atypical Squamous Cells, Cannot Exclude High-Grade Squamous Intraepithelial Lesion) are two categories of cytological findings in cervical cancer screening. The main difference in clinical management between ASC-US and ASC-H is: - ASC-US: Women with ASC-US findings require further evaluation, such as colposcopy and biopsy, to determine if there are high-grade lesions or cancer. If the colposcopy and biopsy results are normal, the woman may be followed up with repeat cytology or HPV testing in 12 months. - ASC-H: Women with ASC-H findings are more likely to have high-grade lesions or cancer, and therefore, they require immediate colposcopy and biopsy. If the colposcopy and biopsy results are abnormal, treatment may be initiated, depending on the severity of the lesion. | C | C | C |
| HuatuoGPT (With prompt) | 60. What is the difference in clinical management between ASC-US and ASC-H? ASC-US is defined as cytologic changes of uncertain significance that require further evaluation. Typically, this involves a repeat smear at 18 months to confirm the persistence or disappearance of the abnormal cells. ASC-H, on the other hand, refers to highly abnormal cells that require immediate invasive diagnostic testing, such as cervical biopsy or colposcopy, to determine the presence or absence of cervical cancer. | A | A | A |
| HuatuoGPT | 60. There are differences in clinical management between ASC-US and ASC-H. ASC-US refers to atypical squamous cells of indeterminate significance and is usually a finding found on routine pap smears. patients with ASC-US usually need to be re-tested for HPV for further evaluation of the need for further treatment. ASC-H, on the other hand, refers to suspicious high-grade squamous cell changes, which implies a potentially more serious risk.Patients with ASC-H usually undergo further testing and treatment, such as cervical biopsy. | B | B | B |
| BioMedLM 2.7B (With prompt) | 60. what is the difference in clinical management between ASC-US and ASC-H? These are two different diagnoses, and the management of each is different. The management of ASC-US is to repeat the Pap smear in 6 months, and if the repeat is negative, the patient can be followed up with a Pap smear in 12 months. The management of ASC-H is to repeat the Pap smear in 3 months, and if the repeat is negative, the patient can be followed up with a Pap smear in 6 months. | D | D | D |
| BioMedLM 2.7B | 60. what is the difference in clinical management between ASC-US and ASC-H? ### Response: The difference in clinical management between ASC-US and ASC-H is that the former is a benign finding and the latter is a possible precursor of cervical cancer. | D | D | D |
| Microsoft Phi2 (With prompt) | 60. The clinical management of ASC-US is different from ASC-H, as ASC-US requires further evaluation and management, while ASC-H does not require further evaluation and management. | C | C | C |
| Microsoft Phi2 | 60. The key difference in clinical management between ASC-US and ASC-H is that ASC-US is a precancerous lesion that requires further evaluation, while ASC-H is a cancerous lesion that requires immediate treatment. ASC-US can be managed with close monitoring and follow-up, while ASC-H requires surgery, radiation therapy, or chemotherapy. | D | D | D |
| QiZhenGPT (With prompt) | 60. What is the difference in clinical management between ASC-US and ASC-H? * ASC-US: + Presence of abnormal cells in the cervical scraping + Low-grade (G1-G2) cancer + Low-risk of progression to advanced cancer + Requires less treatment than high-grade (G3-G4) cancer * ASC-H: + Presence of abnormal cells in the cervical scraping + High-grade (G3-G4) cancer + High-risk of progression to advanced cancer + Requires more treatment than ASC-US Note: These are general guidelines and the specific recommendations may vary depending on the country and context. | C | C | C |
| QiZhenGPT | 60. ASC-US and ASC-H are both methods for cervical cancer screening, but ASC-US is a visual inspection method and ASC-H is a Betadine solution method. The main difference is that ASC-US is more applicable in low-income countries with limited resources, while ASC-H is more applicable in high-income countries with better resources. | D | D | D |
| **61.Is four-quadrant sampling still necessary for patients with no abnormalities on colposcopy?** | | | | |
| ChatGPT4.0 turbo (With prompt) | 61. **Four-Quadrant Sampling in Colposcopy**: Four-quadrant sampling, or directed biopsies of the cervix, is generally not necessary if the colposcopy is completely normal and there are no visible lesions. However, if there is any discrepancy between the cytology results and the colposcopic findings, or if the transformation zone is not fully visible, taking biopsies from the four quadrants may still be recommended to ensure that no high-grade lesions are missed. The decision to perform four-quadrant sampling should be based on the totality of clinical findings and the quality of the colposcopic examination. | A | A | A |
| ChatGPT4.0 turbo | 61. FourQuadrant Sampling in Colposcopy  Fourquadrant sampling, or directed biopsies of the cervix, is traditionally recommended when colposcopic findings are abnormal. However, if no abnormalities are detected on colposcopy, the necessity of fourquadrant sampling can be questioned. Current guidelines suggest that in the absence of any visible lesions and if the colposcopy is deemed adequate (i.e., the transformation zone is fully visible and there are no areas suspicious for highgrade disease), further biopsies may not be necessary. Decisions can depend on the patient's history, risk factors, and the quality of the colposcopy examination. | A | A | A |
| Claude 2 (With prompt) | 61. Four-quadrant sampling is still recommended even in patients with normal colposcopic findings. It allows detection of high-grade lesions that may be missed on initial visual inspection and thus guides treatment decisions. | A | A | A |
| Claude 2 | 61. Four-quadrant cervical biopsies are still recommended for patients with no visible abnormalities on colposcopy. This allows detection of high-grade lesions that may be missed visually. However, some guidelines allow modification to two-quadrant sampling if certain low-risk criteria are met on repeat testing over time. Here are the answers to your additional questions: | D | D | D |
| Gemini Pro (With prompt) | 61. Is four-quadrant sampling still necessary for patients with no abnormalities on colposcopy?  No, four-quadrant sampling is not necessary for patients with no abnormalities on colposcopy. According to the latest guidelines, colposcopy with directed biopsies is sufficient for the evaluation of patients with no abnormalities on colposcopy. | A | A | A |
| Gemini Pro | 61. **Is four-quadrant sampling still necessary for patients with no abnormalities on colposcopy?** No, four-quadrant sampling is not necessary for patients with no abnormalities on colposcopy. A single biopsy from the transformation zone is sufficient to rule out precancerous lesions in these patients. | C | C | C |
| Mistral-7B-v0.2 (With prompt) | 61.No, four-quadrant sampling is not necessary for patients with no abnormalities on colposcopy. Four-quadrant sampling is a method used to obtain cervical cells for cytology testing from all four quadrants of the cervix. It is typically used when there are abnormalities seen on colposcopy or when cytology results are abnormal. | A | A | A |
| Mistral-7B-v0.2 | 61. Four-quadrant sampling and no abnormalities on colposcopy: Generally, four-quadrant sampling is not necessary when there are no abnormalities on colposcopy. Colposcopy is a highly sensitive test used to identify precancerous and abnormal areas on the cervix. If the colposcopy report is normal, the healthcare provider may rely on the negative colposcopy result and forgo a four-quarter sampling. Moreover, since most cases of cervical intraepithelial neoplasia (CIN) associated with HPV do not progress to invasive cancer, the emphasis is on screening and monitoring rather than invasive diagnostic procedures in women with no or minimal abnormalities on colposcopy. | A | A | A |
| Starling-LM-7B alpha (With prompt) | 61. While four-quadrant sampling was previously recommended for all patients, current guidelines suggest that it may not be necessary for patients with no abnormalities on colposcopy. Instead, risk stratification and targeted sampling based on risk factors and colposcopy findings are recommended. | A | A | A |
| Starling-LM-7B alpha | 61. Yes, four-quadrant sampling is still necessary for patients with no abnormalities on colposcopy. It is a standard procedure to ensure comprehensive sampling of the cervix and to detect any potential pre-cancerous or cancerous lesions that may not be visible during the colposcopy examination. | D | D | D |
| HuatuoGPT (With prompt) | 61. Is four-quadrant sampling still necessary for patients with no abnormalities on colposcopy? Four-quadrant sampling is usually not necessary in patients with no abnormalities on colposcopy. Four-quadrant sampling is a pap smear used to detect precancerous lesions of the cervix and cervical cancer. However, if colposcopy does not show an abnormality, patients usually do not need to undergo this procedure. | A | A | A |
| HuatuoGPT | 61. In cervical microscopy, is it still necessary to perform quartile sampling in patients with no abnormalities? Yes, Quartile sampling (QF) is still part of cervical microscopy and is designed to obtain additional cervical tissue to detect areas that may be covered by columnar epithelium. This is to further assess the presence or absence of cervical intraepithelial neoplasia (CIN) and to determine the need for further treatment. | D | D | D |
| BioMedLM 2.7B (With prompt) | 61. is four-quadrant sampling still necessary for patients with no abnormalities on colposcopy? The answer is no. The purpose of colposcopy is to identify the presence of cervical intraepithelial neoplasia (CIN) and to determine the extent of the lesion. The presence of CIN is not a sufficient indication for treatment. The presence of CIN is not a sufficient indication for treatment. The presence of CIN is not a sufficient indication for treatment. The presence of CIN is not a sufficient indication for treatment. | D | D | D |
| BioMedLM 2.7B | 61. is four-quadrant sampling still necessary for patients with no abnormalities on colposcopy? ### Response: Yes. | D | D | D |
| Microsoft Phi2 (With prompt) | 61. No, four-quadrant sampling is not necessary for patients with no abnormalities on colposcopy. | A | A | A |
| Microsoft Phi2 | 61. No, four-quadrant sampling is not necessary for patients with no abnormalities on colposcopy. | A | A | A |
| QiZhenGPT (With prompt) | 61. Is four-quadrant sampling still necessary for patients with no abnormalities on colposcopy? Answer: Yes, four-quadrant sampling is still necessary for patients with no abnormalities on colposcopy. | D | D | D |
| QiZhenGPT | 61. Four-quadrant sampling is not necessary for patients with no abnormalities on colposcopy | A | A | A |
| **62.What are the differences in cervical cancer screening and management strategies for women during pregnancy?** | | | | |
| ChatGPT4.0 turbo (With prompt) | 62. **Cervical Cancer Screening and Management During Pregnancy**: During pregnancy, cervical cancer screening can be performed as per usual screening intervals. However, the management of abnormal findings differs to avoid harm to the developing fetus. Colposcopy is safe during pregnancy, but biopsy is generally reserved for cases where high-grade lesions are suspected. Treatment of cervical dysplasia is usually deferred until after delivery, unless invasive cancer is suspected. The management strategies need to be individualized, balancing the risks and benefits to both mother and fetus. | A | A | A |
| ChatGPT4.0 turbo | 62. Cervical Cancer Screening and Management During Pregnancy Cervical cancer screening and management during pregnancy require careful consideration to balance the risks and benefits of interventions:  Screening: Pap tests can be safely performed during pregnancy. However, HPV testing is also safe but is generally deferred unless needed for followup of an abnormal Pap test result.  Management of Abnormal Results: Colposcopy is safe during pregnancy, but biopsy is generally reserved for cases where highgrade lesions are suspected. Endocervical curettage is avoided due to the risk of complications.  Treatment: Definitive treatment is usually postponed until after delivery unless invasive cancer is diagnosed. Management during pregnancy is typically conservative, focusing on monitoring and delaying treatment until postpartum if possible. | A | A | A |
| Claude 2 (With prompt) | 62. Pregnant patients should not undergo HPV/cytology co-testing. Cytology alone is recommended with colposcopy for ASC-US+. Treatment of CIN is generally deferred until after pregnancy unless invasive cancer is suspected. | B | C | B |
| Claude 2 | 62. The main differences for cervical cancer screening in pregnancy are: - Cytology screening is still recommended during pregnancy as per standard guidelines.  - Colposcopy with biopsy can be performed during pregnancy but is typically deferred until postpartum, especially if invasion is not suspected. - Treatment of precancerous lesions is deferred until after delivery to reduce risk of complications. - Management depends on gestational age and requires a multidisciplinary approach involving obstetrics. | B | B | B |
| Gemini Pro (With prompt) | 62. What are the differences in cervical cancer screening and management strategies for women during pregnancy?   Screening: Pregnant women should undergo cervical cancer screening according to the same guidelines as non-pregnant women.  Management:  Pregnant women with low-grade cervical intraepithelial neoplasia (CIN): Conservative management with close follow-up is recommended.  Pregnant women with high-grade CIN: Treatment should be individualized based on the stage of pregnancy and the severity of the CIN. Options include loop electrosurgical excision procedure (LEEP) or cold knife conization. | B | B | B |
| Gemini Pro | 62. **What are the differences in cervical cancer screening and management strategies for women during pregnancy?** * **Screening:** Cervical cancer screening is not routinely recommended during pregnancy. This is because the hormonal changes of pregnancy can cause changes in the appearance of the cervix, which can make it difficult to interpret Pap smear results. * **Management:** If a woman is diagnosed with cervical cancer during pregnancy, the treatment will depend on the stage of the cancer and the gestational age of the fetus. In general, treatment will be delayed until after the baby is born, unless the cancer is advanced.  Diagnosis | C | B | C |
| Mistral-7B-v0.2 (With prompt) | 62.During pregnancy, cervical cancer screening is typically delayed until after delivery due to the risks associated with radiation exposure from Pap testing. Instead, pregnant women are monitored closely for any signs or symptoms of cervical cancer. If cervical cancer is diagnosed during pregnancy, the treatment options may depend on the stage and location of the cancer, as well as the health of the mother and fetus. | C | C | C |
| Mistral-7B-v0.2 | 62. Cervical cancer screening and management strategies for women during pregnancy: Screening: Current recommendations for cervical cancer screening during pregnancy depend on gestational age: Women with a history of normal Pap test results and/or negative HPV test results prior to pregnancy can delay screenings until after delivery. Women with abnormal Pap test results, history of abnormal Pap tests, abnormal HPV test results, or other risk factors should undergo further evaluation. The preferred approach for these women is colposcopy due to the safety concerns associated with cryosurgery and LEEP during pregnancy. Management: For women diagnosed with cervical cancer during pregnancy, treatment decisions depend on the gestational age, cancer stage, and overall health of the mother and fetus. The options include: Deferral of treatment until after delivery. Treatment prior to delivery if the mother or fetus is at risk or if delaying treatment may worsen the condition. Adjuvant treatment (such as radiation therapy) may be administered postpartum if needed. | C | C | C |
| Starling-LM-7B alpha (With prompt) | 62. Cervical cancer screening and management strategies during pregnancy should be tailored to the individual patient's risk factors and clinical presentation. In general, cervical cytology (Pap smear) and human papillomavirus (HPV) testing should be performed as part of routine prenatal care. Colposcopy and biopsy may be indicated if abnormalities are detected. Treatment options, such as loop electrosurgical excision procedure (LEEP) or cold knife conization, should be carefully considered, taking into account the potential risks to both the mother and the fetus. | B | B | B |
| Starling-LM-7B alpha | 62. Cervical cancer screening and management strategies for pregnant women are similar to those for non-pregnant women, but with some modifications. Screening is typically performed using cytology (Pap smear) and HPV testing. Management strategies include colposcopy, biopsy, and treatment if necessary. However, treatment options during pregnancy are limited due to potential risks to the fetus. Surgery, such as conization or loop electrosurgical excision procedure (LEEP), may be performed if necessary, but radiation therapy and chemotherapy are generally avoided. | B | B | B |
| HuatuoGPT (With prompt) | 62. How does the cervical cancer screening and management strategy for pregnant women differ from that of the general population? Cervical cancer screening and management strategies for pregnant women are different from those for non-pregnant women because there are some limitations and challenges associated with cervical cancer screening and management during pregnancy. Cervical cancer screening should continue during pregnancy, but non-invasive methods such as smear tests or HPV testing should be used. In some cases, invasive diagnostic testing, such as cervical biopsy, may be necessary, but this should be performed in the second trimester of pregnancy. | B | B | B |
| HuatuoGPT | 62. How does the cervical cancer screening and management strategy for pregnant women differ from business as usual? During pregnancy, cervical cancer screening and management strategies are the same as usual because pregnancy does not change the risk or progression of cervical cancer. However, physiologic changes during pregnancy, such as cervical dilation and bleeding, may make it more difficult to perform pap smears or other screening methods. Regular cervical cancer screening during pregnancy is therefore particularly important to ensure early detection and treatment of any abnormalities. | C | C | C |
| BioMedLM 2.7B (With prompt) | 62. what are the differences in cervical cancer screening and management strategies for women during pregnancy? The incidence of cervical cancer is higher in pregnant women than in the general population. The American College of Obstetricisit.^[@bib1]^ The American Cancer Society (ACS) recommends that all pregnant women undergo cervical cancer screening at the first prenatal visit.^[@bib2]^ The ACS also recommends that women with a history of cervical cancer or cervical intraepithelial neoplasia (CIN) undergo cervical cancer screening at the first prenatal visit.^[@bib2]^ ians and Gynecologists (ACOG) recommends that all pregnant women undergo cervical cancer screening at the first prenatal | C | B | C |
| BioMedLM 2.7B | 62. what are the differences in cervical cancer screening and management strategies for women during pregnancy? ### Response: Cervical cancer screening and management strategies for women during pregnancy are similar to those for nonpregnant women. The American College of Obstetricians and Gynecologists (ACOG) recommends that women aged 21 to 65 years be screened for cervical cancer at least once in their lifetime.^[@bib1]^ The ACOG recommends that women aged 21 to 65 years be screened for cervical cancer at least once in their lifetime.^[@bib1]^ The ACOG recommends that women aged 21 to 65 years be screened for cervical cancer at least once in their lifetime.^[@bib1]^ | C | D | D |
| Microsoft Phi2 (With prompt) | 62. Cervical cancer screening and management strategies for women during pregnancy are different from those for non-pregnant women. Pregnant women should be screened for cervical cancer using a combination of Pap smear and HPV testing. If abnormalities are detected, further management may involve a more conservative approach to avoid harm to the fetus. | B | B | B |
| Microsoft Phi2 | 62. Cervical cancer screening and management strategies for women during pregnancy are different from those for non-pregnant women. Pregnant women should be screened for cervical cancer using a combination of HPV testing and cytology. If abnormal results are found, further testing and treatment should be done to ensure the safety of both the mother and the baby. | C | C | C |
| QiZhenGPT (With prompt) | 62. what are the differences in cervical cancer screening and management strategies for women during pregnancy? Answer: Pregnancy can increase the risk of cervical cancer, and women should undergo regular colposcopy and human immunodeficiency virus (HIV) testing during pregnancy and after delivery. The screening and management strategies for women during pregnancy may differ from those for non- pregnant women. | C | C | C |
| QiZhenGPT | 62. Yes | D | D | D |
| **63. What is the preferred clinical diagnosis of cervical cancer?** | | | | |
| ChatGPT4.0 turbo (With prompt) | 63. The preferred clinical diagnosis of cervical cancer typically involves a combination of a thorough history, physical examination (including a pelvic examination), and visualization of the cervix, often aided by colposcopy. A Pap test may raise suspicion, but diagnosis requires further confirmation. Colposcopy-directed biopsy is essential for histological confirmation when lesions are visible or cytology results suggest malignancy. | B | B | B |
| ChatGPT4.0 turbo | 63. Preferred Clinical Diagnosis of Cervical Cancer: The preferred clinical diagnosis of cervical cancer typically begins with a pelvic examination, including a Pap test (Papanicolaou test) and a human papillomavirus (HPV) test. If these tests indicate abnormalities, a colposcopy is often performed, which involves examining the cervix with a magnifying device. If suspicious areas are detected during colposcopy, a biopsy (e.g., punch biopsy, endocervical curettage) is taken to confirm the diagnosis through histological examination. | B | B | B |
| Claude 2 (With prompt) | 63. The preferred initial diagnosis of cervical cancer is made by punch biopsy interpreting cervical histology and human papillomavirus (HPV) testing if available. This guides further management. | B | B | B |
| Claude 2 | 63. The preferred clinical diagnosis of cervical cancer is through a pelvic exam, Pap smear, and colposcopic biopsy of any suspicious lesions. This allows visual and histologic confirmation of cancer. | B | B | B |
| Gemini Pro (With prompt) | 63. Biopsy | B | B | B |
| Gemini Pro | 63. **Preferred clinical diagnosis of cervical cancer**:  - **Colposcopy with biopsy**. | B | B | B |
| Mistral-7B-v0.2 (With prompt) | 63.The preferred clinical diagnosis of cervical cancer is made based on a combination of symptoms, physical examination findings, and diagnostic tests. The most common symptom is abnormal vaginal bleeding, but other symptoms may include pelvic pain, pain during sexual intercourse, and difficulty urinating or having a bowel movement. During a pelvic examination, the healthcare provider may be able to detect abnormalities in the cervix or vaginal area. Diagnostic tests may include a Pap test, HPV testing, and imaging studies such as CT scans or MRI scans. A biopsy may also be necessary to make a definitive diagnosis. | A | B | A |
| Mistral-7B-v0.2 | 63. The preferred clinical diagnosis of cervical cancer is made through a combination of methods including a woman's medical history, physical examination, and diagnostic tests. The most common diagnostic test for cervical cancer is a biopsy, which involves removing a small sample of tissue from the cervix for laboratory examination. Other diagnostic tests may include a Pap test, human papillomavirus (HPV) testing, and colposcopy. | B | B | B |
| Starling-LM-7B alpha (With prompt) | 63. The preferred clinical diagnosis of cervical cancer is based on a combination of clinical, radiological, and pathological findings. The process typically involves:  a) A thorough medical history and physical examination, including a pelvic examination and assessment of the patient's overall health status. b) Imaging studies, such as pelvic ultrasound, computed tomography (CT) scan, or magnetic resonance imaging (MRI), to assess the extent of the disease and identify any metastatic lesions. c) Colposcopy and biopsy of the cervix to confirm the presence of malignant cells and determine the histological subtype and grade of the cancer. | C | C | C |
| Starling-LM-7B alpha | 63. The preferred clinical diagnosis of cervical cancer is through a combination of clinical examination, cytological tests, and histological examination of biopsy specimens. | C | D | D |
| HuatuoGPT (With prompt) | 63. What is the primary clinical diagnosis of cervical cancer? Answer: The primary clinical diagnosis of cervical cancer is cervical biopsy. | B | B | B |
| HuatuoGPT | 63. The most preferred clinical diagnosis for cervical cancer is cervical cancer. This means that no specific clinicopathologic or laboratory findings are prioritized for use in the diagnosis of cervical cancer. Instead, the diagnosis is based on the patient's history, physical examination, and a combination of these findings. | D | D | D |
| BioMedLM 2.7B (With prompt) | 63. What is the preferred clinical diagnosis of cervical cancer? Cervical cancer is a malignant neoplasm of the cervix. The most common type of cervical cancer is squamous cell carcinoma. The most common symptoms of cervical cancer are abnormal vaginal bleeding, postcoital bleeding, and pelvic pain. T | D | D | D |
| BioMedLM 2.7B | 63. What is the preferred clinical diagnosis of cervical cancer? ### Response: Cervical cancer is the most common cancer in women worldwide. It is caused by a sexually transmitted infection, human papillomavirus (HPV). The most common symptoms of cervical cancer are abnormal vaginal bleeding and discharge. | D | C | D |
| Microsoft Phi2 (With prompt) | 63.The preferred clinical diagnosis of cervical cancer is based on the Bethesda System for Reporting Cervical Cytology. | C | C | C |
| Microsoft Phi2 | 63. The preferred clinical diagnosis of cervical cancer is based on the presence of abnormal vaginal bleeding, pelvic pain, and pain during intercourse. | C | C | C |
| QiZhenGPT (With prompt) | 63. What is the preferred clinical diagnosis of cervical cancer? The preferred clinical diagnosis of cervical cancer is stage IA1. | D | D | D |
| QiZhenGPT | 63. The preferred clinical diagnosis of cervical cancer is squamous cell carcinoma (SCC). | D | D | D |
| **64.** **What tests should be conducted to make a pathologic diagnosis of cervical cancer?** | | | | |
| ChatGPT4.0 turbo (With prompt) | 64. To make a pathologic diagnosis of cervical cancer, several tests are conducted:  Colposcopy-directed biopsy**: This is performed when abnormal cells are found during a Pap test or if the cervix appears abnormal during examination. It involves using a colposcope to examine the cervix and direct biopsy of any suspicious areas.  Endocervical curettage**: To sample cells from the cervical canal, which might not be accessible during a simple biopsy.  Cone biopsy (conization)**: This may be used for diagnosis and treatment when deeper layers of cervical cells need to be examined.   HPV DNA testing**: This test can identify the presence of high-risk HPV types that are associated with cervical cancer and may be used in conjunction with other diagnostic procedures. | B | A | A |
| ChatGPT4.0 turbo | 64. Tests for Pathologic Diagnosis: To make a pathologic diagnosis of cervical cancer, the following tests are conducted:   Biopsy: A tissue sample is taken from the cervix during a colposcopy and examined under a microscope to look for cancer cells.  Cone Biopsy (Conization): A larger, coneshaped sample of tissue from the cervix is removed and examined.  LEEP (Loop Electrosurgical Excision Procedure): This procedure uses an electric wire loop to obtain a tissue sample.  Endocervical Curettage (ECC): Scraping of cells from inside the endocervical canal can detect cancer cells higher up in the cervix that may not be visible during colposcopy. | A | A | A |
| Claude 2 (With prompt) | 64. Pathologic diagnosis is made after definitive surgery by microscopic examination of the hysterectomy or cone specimen assessing tumor histology, size, depth of invasion, margins status, lymphovascular invasion and lymph node analysis if lymphadenectomy performed. | A | A | A |
| Claude 2 | 64. Pathologic diagnosis of cervical cancer is made by microscopic examination of a cervical biopsy. This evaluates cancer type, grade, depth of invasion and other prognostic factors. Additional tests include imaging like MRI to see local extent. | B | B | B |
| Gemini Pro (With prompt) | 64. Histopathology and cytology | D | D | D |
| Gemini Pro | 64. **Tests for pathologic diagnosis of cervical cancer**:  - **Biopsy**: Removal of a small sample of tissue from the cervix for examination under a microscope.  - **Cone biopsy**: Removal of a cone-shaped portion of the cervix to obtain a larger sample for more detailed examination.  - **LEEP (loop electrosurgical excision procedure)**: Removal of a thin layer of tissue from the cervix using a wire loop with an electrical current.  - **Punch biopsy**: Removal of a small, round piece of tissue from the cervix using a punch tool. | A | B | A |
| Mistral-7B-v0.2 (With prompt) | 64.To make a pathologic diagnosis of cervical cancer, a biopsy is necessary. This may be performed during a colposcopy, a procedure in which the healthcare provider uses a magnifying instrument to examine the cervix and vaginal area. A sample of tissue is then removed and examined under a microscope for the presence of cancer cells. Other diagnostic tests, such as HPV testing and imaging studies, may also be used to help confirm the diagnosis and determine the stage of the cancer. | A | A | A |
| Mistral-7B-v0.2 | 64. To make a pathologic diagnosis of cervical cancer, the following tests should be conducted: - Colposcopy: A colposcopy is a visual examination of the cervix and vagina using a magnifying instrument. It allows the healthcare provider to closely examine the cervix for abnormalities. - Biopsy: A biopsy is the removal of a small sample of tissue for laboratory examination. A biopsy is the only definitive way to diagnose cervical cancer. - HPV testing: HPV testing can help identify the presence of high-risk HPV strains that are associated with cervical cancer. - Cone biopsy: A cone biopsy is a more extensive procedure that removes a larger sample of tissue from the cervix. It is often used when a smaller biopsy is not able to provide a clear diagnosis. | A | A | A |
| Starling-LM-7B alpha (With prompt) | 64. To make a pathologic diagnosis of cervical cancer, the following tests should be conducted:  a) Colposcopy and biopsy of the cervix to obtain tissue samples for histological examination. This is the gold standard for diagnosing cervical cancer and determining the histological subtype and grade of the tumor. b) Immunohistochemistry (IHC) staining to identify specific markers, such as p16, Ki-67, and HPV, which can help confirm the diagnosis and provide prognostic information. c) Molecular testing, such as polymerase chain reaction (PCR) or fluorescence in situ hybridization (FISH), to detect the presence of high-risk human papillomavirus (HR-HPV) DNA, which is strongly associated with the development of cervical cancer. | A | A | A |
| Starling-LM-7B alpha | 64. To make a pathologic diagnosis of cervical cancer, the following tests should be conducted:  - Colposcopy: A detailed examination of the cervix using a colposcope, which is a specialized magnifying instrument. This helps to visualize abnormal areas of the cervix that may be precancerous or cancerous.  - Biopsy: A small sample of tissue is taken from the cervix and examined under a microscope to confirm the presence of cancerous cells.  - Histopathology: The removed tissue is examined in detail to determine the type and grade of the cancer, which helps in determining the appropriate treatment plan. | A | B | A |
| HuatuoGPT (With prompt) | 64. What tests are needed to diagnose cervical cancer? Ans: The following tests are usually required for the diagnosis of cervical cancer: (a) pap smear, (b) HPV test, (c) cervical biopsy, and (d) imaging tests such as pelvic ultrasound and CT scan. | B | B | B |
| HuatuoGPT | 64. Tests used in the diagnosis of cervical cancer at the pathologic level of anatomy include: (a) pap smear, (b) cervical biopsy, (c) pelvic ultrasound, (d) magnetic resonance imaging (MRI), (e) positron emission tomography (PET-CT) scan, and (f) a number of others, such as lymph node biopsy or laparoscopy. These tests help to determine the stage and extent of spread of cervical cancer and thus guide treatment decisions. | C | C | C |
| BioMedLM 2.7B (With prompt) | 64. What tests should be conducted to make a pathologic diagnosis of cervical cancer?  Pap smear HPV test Colposcopy Biopsy Others (please specify) | B | A | A |
| BioMedLM 2.7B | 64. What tests should be conducted to make a pathologic diagnosis of cervical cancer?  ### Response: Pap smear HPV testColposcopy Biopsy Other (please specify) | C | B | B |
| Microsoft Phi2 (With prompt) | 64.To make a pathologic diagnosis of cervical cancer, a biopsy of the cervix should be taken and examined under a microscope. | A | A | A |
| Microsoft Phi2 | 64. The tests that should be conducted to make a pathologic diagnosis of cervical cancer are a biopsy of the cervix and a histopathologic examination of the biopsy. | A | B | B |
| QiZhenGPT (With prompt) | 64. What tests should be conducted to make a pathologic diagnosis of cervical cancer? Answer: A biopsy of the tissue from the affected area, a routine Papanella test, a test forpv, and a DNA test forpv. | A | B | A |
| QiZhenGPT | 64. To make a pathologic diagnosis of cervical cancer, several tests should be conducted, including:  Visual inspection andacetocarmine staining (VIA) or Bethesda System for reporting cervical cancer  colposcopy, which allows for a more accurate diagnosis and treatment of cervical precancerous lesions  Biopsy, which is used to confirm the diagnosis and determine the type of cancer | C | B | C |
| **65.What tumor markers can be tested for laboratory diagnosis of cervical cancer? What is the significance of each tumor marker detected?** | | | | |
| ChatGPT4.0 turbo (With prompt) | 65. In the context of cervical cancer, tumor markers are not typically used for primary diagnosis but can be useful in monitoring treatment response or detecting recurrence. The following are some of the markers that may be considered in the management of cervical cancer:  Squamous Cell Carcinoma Antigen (SCC-Ag)**: This marker is elevated in many patients with squamous cell carcinoma of the cervix, the most common type of cervical cancer. It can be used to monitor disease progression and response to treatment, as well as to detect recurrence.  Carcinoembryonic Antigen (CEA)**: Though not specific to cervical cancer and more commonly associated with colorectal cancer, CEA can be elevated in cervical cancer and might be used in conjunction with other markers to monitor treatment response.  CA-125**: More commonly associated with ovarian cancer, CA-125 can also be elevated in some cases of cervical cancer, particularly those with advanced disease. It may be useful in monitoring response to therapy in advanced stages or in cases with metastatic spread.  Each of these markers has limitations in sensitivity and specificity; therefore, their use is generally supplementary to primary diagnostic methods such as biopsy and imaging. | A | A | A |
| ChatGPT4.0 turbo | 65. Tumor Markers for Laboratory Diagnosis: While not typically used for the primary diagnosis of cervical cancer, certain tumor markers can be helpful in the management of the disease:   Squamous Cell Carcinoma Antigen (SCCAg): This marker can be elevated in squamous cell carcinoma of the cervix and may be used to monitor treatment response or detect recurrence.  Carcinoembryonic Antigen (CEA): Though not specific, CEA can be elevated in cervical adenocarcinomas and may also serve as a marker for treatment response or recurrence.  CA125: Elevated levels of CA125 are more commonly associated with ovarian cancer but can also be found in advanced cervical cancer and may be useful in monitoring the disease. | A | A | A |
| Claude 2 (With prompt) | 65. Serum tumor markers that can be tested for cervical cancer include SCC antigen and CA-125. Elevated SCC levels suggest squamous histology and can indicate tumor size/burden. CA-125 may be elevated with adenocarcinomas. Neither tumor marker is specific or sensitive enough to diagnose cervical cancer alone. | A | A | A |
| Claude 2 | 65. Tumor markers for cervical cancer diagnosis: - Squamous cell carcinoma antigen (SCC Ag): Elevated levels indicate squamous cell cervical cancer. Helps monitor treatment response. - Cancer antigen 125 (CA-125): Can be elevated in cervical adenocarcinoma. Also monitors treatment response.  - Carcinoembryonic antigen (CEA): Can be elevated in mucinous cervical adenocarcinoma specifically. So these tumor markers complement the histologic diagnosis, help determine cancer type and monitor treatment, but are not sufficient alone to diagnose cervical cancer. | A | A | A |
| Gemini Pro (With prompt) | 65. Tumor markers for laboratory diagnosis of cervical cancer: Squamous cell carcinoma:  SCC antigen  CYFRA 21-1 Adenocarcinoma:  CA125  CEA Significance of each tumor marker detected: Elevated levels of SCC antigen and CYFRA 21-1 are associated with squamous cell carcinoma of the cervix. Elevated levels of CA125 and CEA are associated with adenocarcinoma of the cervix. | A | A | A |
| Gemini Pro | 65. **Tumor markers for laboratory diagnosis of cervical cancer**:  - **Squamous cell carcinoma antigen (SCCA)**: Elevated levels may indicate the presence of squamous cell carcinoma of the cervix.  - **Carcinoembryonic antigen (CEA)**: Elevated levels may indicate the presence of adenocarcinoma of the cervix.  - **Human papillomavirus (HPV)**: The presence of HPV DNA in cervical cells is strongly associated with the development of cervical cancer. | A | C | B |
| Mistral-7B-v0.2 (With prompt) | 65.In the laboratory diagnosis of cervical cancer, several tumor markers can be tested. The most commonly used markers include: a) Human Chorionic Gonadotropin (hCG): This marker is typically not used in the diagnosis of cervical cancer as it is not specific to this type of cancer. b) Carcinoembryonic Antigen (CEA): Elevated levels of CEA can indicate the presence of various types of cancers, including cervical cancer. However, it is not a specific marker for cervical cancer and its levels can be normal in early-stage disease. c) Alpha-Fetoprotein (AFP): AFP is not a specific marker for cervical cancer and its levels are typically normal in this type of cancer. d) CA-125: CA-125 is a marker that can be elevated in various types of cancers, including cervical cancer. However, it is not a specific marker for cervical cancer and its levels can be normal in early-stage disease. e) Squamous Cell Carcinoma Antigen (SCCA): SCCA is a marker that can be elevated in squamous cell carcinomas, including cervical cancer. However, its levels can be normal in early-stage disease and it is not a specific marker for cervical cancer. f) p16 and HPV DNA testing: These markers are not tumor markers per se, but they are important in the diagnosis of cervical cancer. p16 is a protein that is overexpressed in cervical cancer cells and can be detected using immunohistochemistry. HPV DNA testing can detect the presence of high-risk HPV types that are associated with cervical cancer. | A | A | A |
| Mistral-7B-v0.2 | 65. The following tumor markers can be tested for laboratory diagnosis of cervical cancer: - CA-125: CA-125 is a protein that is produced by some ovarian and other cancers, including cervical cancer. Elevated levels of CA-125 in the blood can indicate the presence of cancer, but it is not specific to cervical cancer and can be elevated in other conditions as well. - Squamous cell carcinoma antigen (SCCA): SCCA is a protein that is produced by squamous cell carcinomas, including cervical cancer. Elevated levels of SCCA in the blood can indicate the presence of cervical cancer, but it is not a reliable marker for early-stage disease. - AFP (alpha-fetoprotein): AFP is a protein produced by some cancers, including cervical cancer. Elevated levels of AFP in the blood can indicate the presence of cervical cancer, but it is not a reliable marker for early-stage disease. | A | B | B |
| Starling-LM-7B alpha (With prompt) | 65. Tumor markers that can be tested for laboratory diagnosis of cervical cancer include:  a. Human papillomavirus (HPV) DNA: HPV is the most significant risk factor for cervical cancer. The detection of HPV DNA in cervical samples is crucial for the early diagnosis of cervical cancer. The presence of high-risk HPV types, such as HPV 16 and HPV 18, is strongly associated with the development of cervical cancer.  b. p16: The p16 protein is overexpressed in cervical precancerous lesions and cervical cancer. The p16 test is used as a primary screening tool in conjunction with HPV testing to identify women who require further evaluation, such as colposcopy and biopsy.  c. CA 125: CA 125 is a glycoprotein that is elevated in various gynecologic malignancies, including cervical cancer. While CA 125 levels can be useful in monitoring treatment response and detecting recurrence, its role in the initial diagnosis of cervical cancer is limited due to its low specificity.  d. Squamous cell carcinoma antigen (SCC): SCC is a tumor marker that can be elevated in cervical cancer. However, its use in the diagnosis of cervical cancer is limited due to its low sensitivity and specificity.  The significance of each tumor marker detected:  a. HPV DNA: A positive HPV test result indicates the presence of high-risk HPV types, which are strongly associated with the development of cervical cancer. A negative HPV test result, however, does not rule out the presence of cervical cancer, as some cases may be HPV-negative.  b. p16: A positive p16 test result, in conjunction with a positive HPV test result, indicates a higher likelihood of cervical precancerous lesions or cervical cancer. This combination of test results is used to prioritize women for further evaluation, such as colposcopy and biopsy.  c. CA 125: Elevated CA 125 levels can be useful in monitoring treatment response and detecting recurrence. However, its role in the initial diagnosis of cervical cancer is limited due to its low specificity.  d. SCC: Elevated SCC levels can be suggestive of the presence of cervical cancer, but its use in the diagnosis of cervical cancer is limited due to its low sensitivity and specificity. | A | A | A |
| Starling-LM-7B alpha | 65. Some tumor markers that can be tested for laboratory diagnosis of cervical cancer include:  - Human papillomavirus (HPV): HPV is a group of viruses that can cause changes in the cervical cells, leading to cervical cancer. HPV testing is used to detect the presence of high-risk HPV types that are associated with cervical cancer.  - p16: This is an oncoprotein produced by high-risk HPV types. The p16 test is used as a screening tool for cervical cancer, especially in women with normal HPV test results.  - CA125: This is a protein that is elevated in the blood of women with ovarian cancer, but it can also be elevated in women with cervical cancer. However, CA125 is not specific to cervical cancer and can be elevated in other gynecologic cancers and non-cancerous conditions. | B | B | B |
| HuatuoGPT (With prompt) | 65. What are the tumor markers used in the laboratory for cervical cancer diagnosis? What is the significance of each detected tumor marker symbol? Tumor markers that can be used in the laboratory for the diagnosis of cervical cancer include: Squamous cell carcinoma-associated antigen (SCCA) Carcinoembryonic antigen (CEA) Acid esterase antigen 6 (CA6) Squamous cell carcinoma-associated antigen 3 (SCC3) Squamous cell carcinoma-associated antigen 4 (SCC4) Squamous Cell Carcinoma Antigen 5 (SCC5) All these tumor markers are used to detect the presence of cervical cancer and its stage. Some of these markers are used to determine disease recurrence or remission and others are used to assess response to treatment. | B | B | B |
| HuatuoGPT | 65. Tumor markers used in the laboratory diagnosis of cervical cancer include: (a) carcinoembryonic antigen (CEA), (b) squamous cell carcinoma-associated antigen (SCC-Ag), (c) human papillomavirus (HPV) DNA, (d) the antigen Ki-67, and (e) a number of other markers, such as β-2 microglobulin, CA19-9, CA125 and CA15-3. markers are useful in determining the prognosis and outcome of cervical cancer and in guiding treatment decisions and management. | B | B | B |
| BioMedLM 2.7B (With prompt) | 65. What tumor markers can be tested for laboratory diagnosis of cervical cancer? What is the significance of each tumor marker detected?: Tumor markers are substances that are produced by tumor cells and released into the blood. They are used as indicators of the presence of cancer. The most commonly used tumor markers are squamous cell carcinoma antigen (SCC), carcinoembryonic antigen (CEA), carbohydrate antigen (CA) 125, CA 19-9, CA 15-3, and CA 72-4.[@B1]  SCC SCC is a glycoprotein that is produced by squamous epithelial cells. It is a tumor marker that is used to screen for cervical cancer. The sensitivity and specificity of SCC are 80% and 90%, respectively.  CEA CEA is a glycoprotein that is produced by the gastrointestinal tract, respiratory tract, and reproductive system. It is a tumor marker that is used to screen for colorectal cancer. The sensitivity and specificity of CEA are 60% and 90%, respectively.[@B3]  CA 125 CA 125 is a glycoprotein that is produced by the epithelial cells of the ovary, endometrium, and peritoneum. It is a tumor marker that is used to screen for ovarian cancer. The sensitivity and specificity of CA 125 are 60% and 90%, respectively.[@B4]  CA 19-9 CA 19-9 is a glycoprotein that is produced by the gastrointestinal tract, respiratory tract, and reproductive system. It is a tumor marker that is used to screen for pancreatic cancer. The sensitivity and specificity of CA 19-9 are 60% and 90%, respectively.[@B5]  CA 15-3 CA 15-3 is a glycoprotein that is produced by the epithelial cells of the breast, gastrointestinal tract, and respiratory tract. It is a tumor marker that is used to screen for breast cancer. The sensitivity and specificity of CA 15-3 are 60% and 90%, respectively.[@B6]  CA 72-4 CA 72-4 is a glycoprotein that is produced by the epithelial cells of the gastrointestinal tract, respiratory tract, and reproductive system. It is a tumor marker that is used to screen for gastric cancer. The sensitivity and specificity of CA 72-4 are 60% and 90%, respectively.[@B7] | A | A | A |
| BioMedLM 2.7B | 65. What tumor markers can be tested for laboratory diagnosis of cervical cancer? What is the significance of each tumor marker detected? ### Response: The tumor markers that can be tested for cervical cancer include squamous cell carcinoma antigen (SCC), carcinoembryonic antigen (CEA), carbohydrate antigen (CA) 125, CA19-9, CA15-3, CA72-4, and alpha-fetoprotein (AFP). The significance of each tumor marker detected is as follows: SCC, CEA, CA125, CA19-9, and CA15-3 are used for the diagnosis of cervical cancer, and CA72-4 and AFP are used for the diagnosis of ovarian cancer. | A | A | A |
| Microsoft Phi2 (With prompt) | 65. The tumor markers that can be tested for laboratory diagnosis of cervical cancer are carcinoembryonic antigen (CEA), human epidermal growth factor receptor 2 (HER2), and cytokeratin 18 (CK18). CEA is a tumor marker that is produced by most solid tumors, including cervical cancer. The significance of CEA is that it can be used to monitor the response to treatment and to detect recurrence of the disease. HER2 is a protein that is overexpressed in some types of cervical cancer. The significance of HER2 is that it can be targeted by specific drugs that can improve the prognosis of patients with HER2-positive cervical cancer. CK18 is a protein that is overexpressed in some types of cervical cancer. The significance of CK18 is that it can be used to monitor the response to treatment and to detect recurrence of the disease. | A | A | A |
| Microsoft Phi2 | 65. Tumor markers that can be tested for laboratory diagnosis of cervical cancer include human epidermal growth factor receptor 2 (HER2), carcinoembryonic antigen (CEA), and prostate-specific antigen (PSA). HER2 is a protein that is overexpressed in some types of cervical cancer and can be used to guide treatment decisions. CEA is a protein that is often elevated in patients with cervical cancer and can be used to monitor disease progression. PSA is a protein that is produced by the prostate gland and can be elevated in patients with cervical cancer that has spread to the prostate. | A | A | A |
| QiZhenGPT (With prompt) | 65. tumor markers such as Carcinoembryonic antigen (CEA) and Human Epidermal Growth Factor receptor (HER2) can be tested for laboratory diagnosis of cervical cancer. The significance of each tumor marker detected is to aid in the diagnosis and classification of cervical cancer. | B | B | B |
| QiZhenGPT | 65. There are several tumor markers that can be tested for in the laboratory to diagnose cervical cancer, including: * Squamous cell carcinoma (SCC) markers: Carcinoembryonic antigen (CEA), Mucinous cell adenocarcinoma (MCA), and Squamous cell carcinoma (SCC) antigen (SCCAg) * Endocervical cancer markers: Endocervical cancer antigen (ECA) and Cervical cancer antigen (CCA) *squamous cell carcinoma (SCC) markers: Carcinoembryonic antigen (CEA), Mucinous cell adenocarcinoma (MCA), and Squamous cell carcinoma (SCC) antigen (SCCAg) | B | B | B |
| **66. In the diagnosis of cervical cancer, which imaging method should be preferred in order to evaluate cervical tumors? Which imaging method should be used in order to evaluate metastatic lesions?** | | | | |
| ChatGPT4.0 turbo (With prompt) | 66. For the evaluation of cervical tumors:  Magnetic Resonance Imaging (MRI)**: MRI is the preferred imaging method for evaluating cervical tumors. It provides excellent soft tissue contrast and detailed visualization of the cervix, uterus, and surrounding structures. MRI is particularly useful in assessing the extent of tumor invasion into adjacent tissues, which is crucial for accurate staging and treatment planning.  For the evaluation of metastatic lesions:  Computed Tomography (CT) Scan**: CT scans are commonly used to evaluate for metastatic lesions, especially in the lungs and liver. CT scans offer a quick and effective way to scan the entire body and are valuable in detecting distant metastases.  Positron Emission Tomography (PET) Scan**: A PET scan, often combined with a CT scan (PET/CT), is another effective tool for detecting metastatic disease. It can help identify active tumors based on their metabolic activity, providing valuable information for staging and monitoring response to treatment. | A | A | A |
| ChatGPT4.0 turbo | 66. Imaging Methods for Evaluation:  Evaluation of Cervical Tumors: Magnetic Resonance Imaging (MRI) is the preferred imaging method for evaluating local cervical tumors as it provides detailed soft tissue contrast and can help define the extent of the tumor, including its size and depth of invasion into surrounding tissues.  Evaluation of Metastatic Lesions: For evaluating potential metastatic disease:  Computed Tomography (CT): A CT scan is often used to assess the chest, abdomen, and pelvis for metastatic disease, particularly to the lymph nodes and distant organs.  Positron Emission Tomography (PET)/CT: A PET/CT scan can be helpful in detecting distant metastases and in cases where there is suspicion of cancer spread that is not clearly defined on CT or MRI.  Chest Xray: A basic imaging test to check for metastasis to the lungs. | A | A | A |
| Claude 2 (With prompt) | 66. MRI is the preferred imaging modality for evaluating the primary cervical tumor as it provides excellent soft tissue delineation allowing assessment of tumor size, parametrial invasion, and pelvic sidewall involvement. CT scan of the chest/abdomen/pelvis with contrast is recommended to detect distant metastases and complete disease staging. PET/CT can also detect metastatic lesions. | A | A | A |
| Claude 2 | 66. For evaluating the primary cervical tumor: MRI of the pelvis is the preferred imaging method. It allows detailed assessment of tumor size, local extent, parametrial invasion, pelvic sidewall involvement, etc. This helps determine surgical resectability or need for chemoradiation.  For detecting metastatic lesions: Whole body FDG PET/CT scan is the most sensitive imaging test to screen for nodal or distant metastatic spread. It complements pelvic MRI. CT scan of chest/abdomen/pelvis is also commonly done to look for metastases. | A | A | A |
| Gemini Pro (With prompt) | 66. Imaging methods for diagnosis of cervical cancer:  To evaluate cervical tumors:  Colposcopy  Biopsy  MRI To evaluate metastatic lesions:  CT scan  PET scan | A | A | A |
| Gemini Pro | 66. **Imaging methods for diagnosis of cervical cancer**:  - **Cervical tumors**:  - **Colposcopy**: Magnified examination of the cervix using a colposcope.  - **Biopsy**: Removal of a small sample of tissue from the cervix for examination under a microscope.  - **Cone biopsy**: Removal of a cone-shaped portion of the cervix to obtain a larger sample for more detailed examination.  - **MRI (magnetic resonance imaging)**: Provides detailed images of the cervix and surrounding tissues.  - **Metastatic lesions**:  - **CT (computed tomography)**: Provides cross-sectional images of the body to detect metastatic lesions in the pelvis, abdomen, and chest.  - **PET (positron emission tomography)**: Detects metabolic activity in the body and can be used to identify metastatic lesions. | C | C | C |
| Mistral-7B-v0.2 (With prompt) | 66.In the diagnosis of cervical cancer, magnetic resonance imaging (MRI) is the preferred imaging method to evaluate cervical tumors. MRI can provide detailed information about the size, location, and extent of the tumor, as well as the involvement of nearby structures such as the vagina, bladder, and rectum. To evaluate metastatic lesions, computed tomography (CT) scanning or positron emission tomography (PET) scanning may be used. CT scanning can provide detailed information about the size and location of metastatic lesions, while PET scanning can detect metastatic lesions even when they are small and not visible on CT scanning. However, both CT scanning and PET scanning have limitations in terms of their ability to accurately detect small cervical tumors. Therefore, they are typically used to evaluate metastatic disease rather than primary cervical tumors. | A | A | A |
| Mistral-7B-v0.2 | 66. In the diagnosis of cervical cancer, magnetic resonance imaging (MRI) is the preferred imaging method to evaluate cervical tumors. MRI can provide detailed images of the cervix, uterus, and surrounding structures, allowing healthcare providers to assess the size, location, and extent of the tumor. | A | A | A |
| Starling-LM-7B alpha (With prompt) | 66. For the diagnosis of cervical cancer, the preferred imaging method to evaluate cervical tumors is:  a. Transvaginal ultrasound: Transvaginal ultrasound is the first-line imaging modality for the evaluation of cervical tumors. It provides detailed information about the size, location, and extent of the tumor, as well as the presence of lymph node involvement.  For the evaluation of metastatic lesions, the preferred imaging method is:  b. Computed tomography (CT) scan: CT scan is the most commonly used imaging modality for the detection of metastatic lesions in cervical cancer. It provides detailed information about the size, location, and extent of metastatic disease in the pelvis, abdomen, and lungs.  In addition to CT scan, other imaging modalities such as magnetic resonance imaging (MRI) and positron emission tomography (PET) scan may be used in specific cases to better evaluate metastatic disease or treatment response. | C | C | C |
| Starling-LM-7B alpha | 66. In the diagnosis of cervical cancer, the preferred imaging method to evaluate cervical tumors is:  - Magnetic resonance imaging (MRI): MRI provides detailed images of the cervix, uterus, and surrounding structures, allowing for accurate assessment of the size, location, and extent of the tumor. | A | A | A |
| HuatuoGPT (With prompt) | 66. Which imaging method should be prioritized in the evaluation of cervical cancer? Which imaging method should be used to evaluate metastatic lesions? Ultrasonography should be used preferentially in the evaluation of cervical cancer. Ultrasonography is a reliable method of evaluating cervical tumors, especially in the early stages. It can also be used to identify lymph node involvement. For evaluating metastatic lesions in cervical cancer, CT scan is the imaging method of choice. CT scans provide more detailed images that help determine the size and spread of the tumor. MRI can also be used to evaluate metastatic lesions of cervical cancer, but is not the imaging method of choice in this case, as CT scans are more commonly used and more readily available. | C | C | C |
| HuatuoGPT | 66. The imaging method of choice when evaluating cervical cancer is MRI (Magnetic Resonance Imaging).MRI provides more accurate image detail and assessment of the extent and aggressiveness of the tumor. It can help doctors determine the size, location and depth of infiltration of the tumor, as well as assess the involvement of lymph nodes and surrounding tissues. For evaluating metastatic lesions, a commonly used imaging method is PET-CT (Positron Emission Computed Tomography.) PET-CT can detect the presence and distribution of cancer cells using metabolic activity through the injection of a radiolabeled substance. It can provide more systemic information to help doctors determine the presence of distant metastases and assess their extent and location. Both imaging methods play an important role in the diagnosis and evaluation of cervical cancer, but differ in the assessment of different types of lesions. MRI is indicated for assessing the extent of the primary tumor and local invasion, whereas PET-CT is more appropriate for assessing metastatic lesions and systemic disease. | A | A | A |
| BioMedLM 2.7B (With prompt) | 66. In the diagnosis of cervical cancer, which imaging method should be preferred in order to evaluate cervical tumors? Which imaging method should be used in order to evaluate metastatic lesions? The imaging methods used for the diagnosis of cervical cancer include cervical cytology, colposcopy, and imaging modalities such as magnetic resonance imaging (MRI), computed tomography (CT), and positron emission tomography (PET). The choice of imaging method depends on the stage of the disease, the patient\'s age, and the availability of the equipment. Cervical cytology is the most commonly used method for the diagnosis of cervical cancer. However, it is not sensitive enough to detect early-stage cervical cancer. In addition, it is difficult to perform cervical cytology in patients with a history of cervical cancer or in those who have undergone hysterectomy. Colposcopy is a method that can be used to detect cervical cancer and to determine the extent of the disease. However, it is difficult to perform colposcopy in patients with a history of cervical cancer or in those who have undergone hysterectomy. In addition, colposcopy is not sensitive enough to detect early-stage cervical cancer. Therefore, cervical cytology and colposcopy are not sufficient for the diagnosis of cervical cancer. MRI is the most sensitive imaging method for the diagnosis of cervical cancer. However, it is expensive and not widely available. CT is also used for the diagnosis of cervical cancer. However, it is not sensitive enough to detect early-stage cervical cancer. PET is a method that can be used to detect cervical cancer. However, it is expensive and not widely available. | C | C | C |
| BioMedLM 2.7B | 66. In the diagnosis of cervical cancer, which imaging method should be preferred in order to evaluate cervical tumors? Which imaging method should be used in order to evaluate metastatic lesions? ### Response: A. CT scan. B. MRI. C. PET-CT. | C | C | C |
| Microsoft Phi2 (With prompt) | 66. In the diagnosis of cervical cancer, magnetic resonance imaging (MRI) should be preferred in order to evaluate cervical tumors. MRI is a non-invasive imaging method that can provide detailed information about the size, location, and extent of the tumor. MRI can also be used to evaluate the surrounding tissues and organs for signs of metastasis. In the diagnosis of metastatic lesions, computed tomography (CT) should be used in order to evaluate the extent of the disease. CT is a non-invasive imaging method that can provide detailed information about the size, location, and extent of the metastatic lesions. CT can also be used to evaluate the surrounding tissues and organs for signs of metastasis. | A | A | A |
| Microsoft Phi2 | "66. In the diagnosis of cervical cancer, a pelvic ultrasound should be preferred to evaluate cervical tumors. In the evaluation of metastatic lesions, a computed tomography (CT) scan or magnetic resonance imaging (MRI) should be used. | B | C | C |
| QiZhenGPT (With prompt) | 66. In the diagnosis of cervical cancer, imaging methods such as ultrasound and CT scans should be preferred in order to evaluate cervical tumors. Imaging methods such as MRI and X-rays should be used in order to evaluate metastatic lesions. | B | C | C |
| QiZhenGPT | 66. In the diagnosis of cervical cancer, the following imaging methods should be preferred:  Transvaginal ultrasound (TVU) or Transcervical ultrasound (TCU)  HRCT or MRI  PAP Smear with Blueprint Reading For evaluating cervical tumors, HRCT or MRI are preferred as they can provide a more accurate diagnosis and determine the extent of cancer in the body. For evaluating metastatic lesions, CT or MRI is preferred. | A | A | A |
| **67. what are the recommended diagnostic tools for patients with FIGO stage IA1 cervical cancer? Is it necessary to consider lymphovascular infiltration?** | | | | |
| ChatGPT4.0 turbo (With prompt) | 67. **Diagnostic Tools for FIGO Stage IA1 Cervical Cancer**: For patients diagnosed with FIGO stage IA1 cervical cancer, the primary diagnostic tools include colposcopy and a cone biopsy to assess the depth and extent of invasion and to evaluate for lymphovascular space invasion (LVSI). LVSI is a critical factor in determining the risk of metastasis and can influence the decision regarding further treatment, such as lymph node assessment and the extent of surgery. Therefore, it is necessary to consider LVSI in the management plan. | B | B | B |
| ChatGPT4.0 turbo | 67. Diagnostic Tools for FIGO Stage IA1 Cervical Cancer For patients with FIGO stage IA1 cervical cancer, the primary diagnostic tools include:  Colposcopy and Biopsy: To assess the extent and characteristics of the lesion.  Cone Biopsy or Loop Electrosurgical Excision Procedure (LEEP): These not only serve as diagnostic tools but also potentially as treatment.  Lymphovascular Space Invasion (LVSI): The presence of LVSI is a critical factor in determining the risk of lymph node metastases and recurrence. If LVSI is present, further assessment and possibly more extensive treatment may be necessary. | C | C | C |
| Claude 2 (With prompt) | 67. The recommended diagnostic tools for stage IA1 cervical cancer are cone biopsy with pathological analysis for depth of invasion and lymphovascular space involvement. Lymphovascular infiltration assessment is critical as it guides treatment. | B | B | B |
| Claude 2 | 67. The recommended diagnostic tools for FIGO stage IA1 cervical cancer include: - Examination under anesthesia - Cystoscopy/proctoscopy if bladder/rectal extension suspected  - Cervical conization to determine depth of invasion, margins, and lymphovascular space invasion (LVSI) - Pelvic +/- para-aortic lymph node assessment - Imaging like CT/MRI/PET to rule out nodal disease if LVSI is present | B | B | B |
| Gemini Pro (With prompt) | 67. What are the recommended diagnostic tools for patients with FIGO stage IA1 cervical cancer? Is it necessary to consider lymphovascular infiltration?   Recommended diagnostic tools:  Colposcopy with biopsy  Endocervical curettage  Lymphovascular infiltration: Lymphovascular infiltration is not routinely assessed in FIGO stage IA1 cervical cancer. However, it may be considered in cases where there is a high suspicion of invasion, such as in patients with large tumors or those with a history of pelvic inflammatory disease. | B | B | B |
| Gemini Pro | 67. **What are the recommended diagnostic tools for patients with FIGO stage IA1 cervical cancer? Is it necessary to consider lymphovascular infiltration?** The recommended diagnostic tools for patients with FIGO stage IA1 cervical cancer are: * **Colposcopy:** Colposcopy is a procedure that allows the doctor to visualize the cervix and take biopsies of any abnormal areas. * **Biopsy:** A biopsy is a procedure that removes a small sample of tissue from the cervix for examination under a microscope. Lymphovascular infiltration is a factor that can affect the prognosis of patients with cervical cancer. However, it is not necessary to consider lymphovascular infiltration in the diagnosis of FIGO stage IA1 cervical cancer. | B | B | B |
| Mistral-7B-v0.2 (With prompt) | 67.For patients with FIGO stage IA1 cervical cancer, the recommended diagnostic tools include cone biopsy or loop electrosurgical excision procedure (LEEP). These procedures allow for the removal of the abnormal tissue while preserving as much healthy tissue as possible. Lymphovascular infiltration is not typically considered in the management of FIGO stage IA1 cervical cancer, as it is not a factor in the FIGO staging system for this early stage of disease. | B | B | B |
| Mistral-7B-v0.2 | 67. Recommended diagnostic tools for patients with FIGO stage IA1 cervical cancer: Conization (LLETZ or loop electrosurgical excision procedure): The most common diagnostic tool for early-stage cervical cancer, offering accurate assessment of the extent and depth of the cervical lesion. This method is generally considered safe, but its use during pregnancy may depend on individual circumstances. Cone biopsy: A more invasive diagnostic procedure used to completely remove a section of the cervix for histological examination. Due to its risks, it is typically postponed until after pregnancy. Cold knife conization: Less commonly used due to the associated complications and risks, this surgical procedure involves removing the cervical tissue using a scalpel. Considered safer than cone biopsy, LLETZ is often preferred. Immunohistochemistry (IHC): A molecular diagnostic method used to detect specific proteins related to the progression and aggressiveness of cervical cancer, particularly for lesions with ambiguous features. While it can offer important insights, it is typically used as an adjunct to histopathological evaluation rather than as a primary diagnostic tool. | B | B | B |
| Starling-LM-7B alpha (With prompt) | 67. For patients with FIGO stage IA1 cervical cancer, the recommended diagnostic tools include colposcopy, cervical cytology, and HPV testing. Lymphovascular infiltration is not a separate stage in the FIGO staging system, but it is an important prognostic factor. The presence of lymphovascular space invasion (LVSI) is associated with a higher risk of recurrence and should be considered when determining the appropriate treatment approach. | C | B | B |
| Starling-LM-7B alpha | 67. For patients with FIGO stage IA1 cervical cancer, the recommended diagnostic tools include colposcopy, cytology (Pap smear), HPV testing, and biopsy. Lymphovascular infiltration is an important prognostic factor and should be considered in the diagnostic process. | B | B | B |
| HuatuoGPT (With prompt) | 67. What are the recommended diagnostic tools for patients with FIGO stage IA1 cervical cancer? Is it necessary to consider lymphovascular infiltration? In patients with FIGO stage IA1 cervical cancer, consideration of lymphovascular infiltration (LVS) is usually not necessary.FIGO stage IA1 refers to cancers that are microscopically confined to a stage between intraepithelial (CIS) and invasive carcinoma. Usually, this stage of cancer can be diagnosed by colposcopy and histologic evaluation. However, in some cases, other diagnostic tests, such as a chest x-ray or CT scan, may be necessary to rule out lymphovascular infiltration of regional lymph nodes. | C | C | C |
| HuatuoGPT | 67. What are the recommended diagnostic tools for patients with FIGO stage IA1 cervical cancer? Do you need to consider lymphovascular infiltration? For patients with FIGO stage IA1 cervical cancer, diagnostic tools typically include pap smears, cervical biopsies, and imaging studies. A pap smear is a simple screening tool to detect the presence of cervical intraepithelial neoplasia, while a cervical biopsy provides a more accurate tissue diagnosis. Imaging studies, such as MRI and CT scans, can be used to assess tumor size and lymph node status. It is not necessary to consider lymphovascular infiltration because stage IA1 cancer is confined to the cervix and has not yet spread to the lymphatic system. | B | B | B |
| BioMedLM 2.7B (With prompt) | 67. what are the recommended diagnostic tools for patients with FIGO stage IA1 cervical cancer? Is it necessary to consider lymphovascular infiltration? The recommended diagnostic tools for patients with FIGO stage IA1 cervical cancer are colposcopy, cervical biopsy, and endocervical curettage (ECC). The presence of lymphovascular infiltration (LVSI) is an important prognostic factor in patients with cervical cancer.[@B1] However, the presence of LVSI is not included in the current FIGO staging system.[@B2] The presence of LVSI is not included in the current FIGO staging system. The presence of LVSI is not included in the current FIGO staging system. The presence of LVSI is not included in the current FIGO staging system. | B | B | B |
| BioMedLM 2.7B | 67. what are the recommended diagnostic tools for patients with FIGO stage IA1 cervical cancer? Is it necessary to consider lymphovascular infiltration? ### Response: The answer is yes. Lymphovascular infiltration is a risk factor for recurrence and is an indication for adjuvant therapy. | D | D | D |
| Microsoft Phi2 (With prompt) | 67. The recommended diagnostic tools for patients with FIGO stage IA1 cervical cancer include imaging tools such as ultrasound, MRI, and CT scan. Lymphovascular infiltration is not necessary to consider in FIGO staging. | C | C | C |
| Microsoft Phi2 | 67. The recommended diagnostic tools for patients with FIGO stage IA1 cervical cancer include HPV testing, cytology, and imaging tools such as ultrasound and MRI. Lymphovascular infiltration is not necessary for diagnosis. | C | C | C |
| QiZhenGPT (With prompt) | 67. what are the recommended diagnostic tools for patients with FIGO stage IA1 cervical cancer? Is it necessary to consider lymphovascular infiltration? Answer: The recommended diagnostic tools for patients with FIGO stage IA1 cervical cancer are colposcopy, biopsy, and imaging tools such as CT and MRI. It is not necessary to consider lymphovascular infiltration. | B | B | B |
| QiZhenGPT | 67. No | C | D | D |
| **68. How are imaging tools used to assess tumor size and lymph node status in FIGO staging? What specific imaging tools are recommended?** | | | | |
| ChatGPT4.0 turbo (With prompt) | 68. **Imaging in FIGO Staging**: Imaging tools play a crucial role in assessing tumor size and lymph node status in the FIGO staging of cervical cancer. Magnetic resonance imaging (MRI) is the preferred modality for evaluating the local extent of the disease, particularly in assessing parametrial involvement and the size of the tumor. Computed tomography (CT) and positron emission tomography (PET) scans are commonly used to evaluate for distant metastasis and lymph node involvement. These imaging modalities help in planning the appropriate surgical and therapeutic approaches. | A | A | A |
| ChatGPT4.0 turbo | 68. Imaging in FIGO Staging Imaging tools play a crucial role in assessing tumor size and lymph node status in FIGO staging:  MRI (Magnetic Resonance Imaging): Preferred for its superior soft tissue contrast, which is ideal for evaluating the tumor size and depth of cervical stromal invasion.  CT Scan (Computed Tomography): Useful for detecting lymph node metastases and distant metastases but less effective than MRI for local tumor evaluation.  PET/CT: Recommended for higher stages to assess distant metastases and lymph node status, but not typically used for earlystage disease like IA1 unless there are specific indications. | A | A | A |
| Claude 2 (With prompt) | 68. Cross-sectional imaging like MRI is used to assess primary tumor size and CT or PET/CT to evaluate lymph node involvement in FIGO staging. This guides treatment planning. | A | A | A |
| Claude 2 | 68. Cross-sectional imaging like MRI or CT scan can be used to assess primary tumor size and lymph node status in cervical cancer FIGO staging. MRI is more sensitive than CT for assessing parametrial invasion and nodal disease. PET/CT can also detect nodal and metastatic disease. Expert guidelines specifically recommend pelvic +/- para-aortic MRI for staging tumors >2 cm. | B | B | B |
| Gemini Pro (With prompt) | 68. How are imaging tools used to assess tumor size and lymph node status in FIGO staging? What specific imaging tools are recommended?   Imaging tools used:  Transvaginal ultrasound (TVUS)  Magnetic resonance imaging (MRI)  Recommended imaging tool: TVUS is the preferred imaging tool for assessing tumor size and lymph node status in FIGO staging. | A | A | A |
| Gemini Pro | 68. **How are imaging tools used to assess tumor size and lymph node status in FIGO staging? What specific imaging tools are recommended?** Imaging tools are used to assess tumor size and lymph node status in FIGO staging by providing detailed images of the pelvis and abdomen. The most commonly used imaging tools for FIGO staging are: * **Ultrasound:** Ultrasound uses sound waves to create images of the pelvis and abdomen. It is a non-invasive and relatively inexpensive imaging tool. * **MRI (magnetic resonance imaging):** MRI uses magnetic fields and radio waves to create detailed images of the pelvis and abdomen. It is a more expensive imaging tool than ultrasound, but it can provide more detailed images. * **CT (computed tomography):** CT uses X-rays and computers to create detailed images of the pelvis and abdomen. It is a more expensive imaging tool than ultrasound or MRI, but it can provide more detailed images.  Treatment | A | B | A |
| Mistral-7B-v0.2 (With prompt) | 68.Imaging tools are used to assess tumor size and lymph node status in FIGO staging to help determine the extent of the cancer and guide treatment decisions. The specific imaging tools recommended include magnetic resonance imaging (MRI) and computed tomography (CT) scans. MRI is preferred for assessing tumor size and local extension, while CT scans are used to evaluate lymph node status. | A | A | A |
| Mistral-7B-v0.2 | 68. Imaging tools for assessing tumor size and lymph node status in FIGO staging: Magnetic Resonance Imaging (MRI): While not mandatory for all cases, MRI is a useful imaging modality when evaluating tumor size, especially for detecting the presence of deep stromal invasion and assessing the extent of lymph node involvement. MRI can also help determine the appropriate surgical approach and can provide useful information on the presence of potential para-aortic and iliac lymph node metastases. Computed Tomography (CT) scan: Though not the primary choice for staging, CT scans can detect distant metastasis and may be useful in select cases when MRI is not available or contraindicated. CT scans are able to help evaluate organs that are not easily accessible through MRI, such as the lungs and liver, though they are less effective at assessing lymph node involvement. Positron Emission Tomography/Computed Tomography (PET/CT): PET/CT can be used when considering the most advanced imaging technology for staging and to assess the potential spread of the cervical cancer, even at the early stages. PET/CT is sensitive for detecting metastases, lymph node involvement, and local recurrence. However, given the increased cost and radiation exposure, it is typically reserved for more complicated cases where there is a higher risk of metastatic spread. | A | A | A |
| Starling-LM-7B alpha (With prompt) | 68. Imaging tools used to assess tumor size and lymph node status in FIGO staging include magnetic resonance imaging (MRI) and computed tomography (CT) scans. MRI is generally preferred for its superior soft tissue resolution, which allows for more accurate assessment of tumor size and involvement of the parametria, cervical canal, and lower vagina. CT scans may be used to evaluate the pelvic and para-aortic lymph nodes. | A | A | A |
| Starling-LM-7B alpha | 68. Imaging tools used to assess tumor size and lymph node status in FIGO staging include magnetic resonance imaging (MRI) and computed tomography (CT) scans. These imaging modalities provide detailed information about the size, location, and extent of the tumor, as well as the presence of lymph node involvement. | B | B | B |
| HuatuoGPT (With prompt) | 68. What are the imaging tools used to assess tumor size and regional lymph node status? Which specific imaging tool is recommended? Imaging tools used to assess tumor size and regional lymph node status include pelvic ultrasound, CT scan, and MRI, which can be used to assess the size and extent of the tumor and whether it is invading surrounding structures, as well as to identify lymph node status. In some cases, PET-CT may be necessary to further assess regional lymph node status and to determine the presence of distant metastases. | B | B | B |
| HuatuoGPT | 68. How can imaging tools be used to assess tumor size and lymph node status in FIGO staging? What specific imaging tools are recommended? Tumor size and lymph node status in FIGO staging are usually assessed using imaging tools such as magnetic resonance imaging (MRI) and computed tomography (CT). These tools provide detailed images that help doctors determine the size of the tumor, assess whether it has spread to surrounding tissues, and evaluate the presence of lymph node metastases. The routine use of PET / CT scanning is not recommended because it has limited effectiveness in staging rather than being an affordable imaging tool. | B | B | B |
| BioMedLM 2.7B (With prompt) | 68. How are imaging tools used to assess tumor size and lymph node status in FIGO staging? What specific imaging tools are recommended? The imaging tools used to assess tumor size and lymph node status in FIGO staging are computed tomography (CT), magnetic resonance imaging (MRI), and positron emission tomography (PET). CT and MRI are the most commonly used imaging tools for staging cervical cancer. CT is the most widely used imaging tool for staging cervical cancer because it is readily available, inexpensive, and has a high spatial resolution. MRI is the most sensitive imaging tool for detecting parametrial invasion and lymph node metastasis. PET is not routinely used for staging cervical cancer because of its low sensitivity and specificity. | B | B | B |
| BioMedLM 2.7B | 68. How are imaging tools used to assess tumor size and lymph node status in FIGO staging? What specific imaging tools are recommended? ### Response: The imaging tools used to assess tumor size and lymph node status in FIGO staging are not standardized. The most commonly used imaging tools are computed tomography (CT) and magnetic resonance imaging (MRI). The use of CT and MRI is recommended for the assessment of tumor size and lymph node status. | B | B | B |
| Microsoft Phi2 (With prompt) | 68. Imaging tools such as ultrasound, MRI, and CT scan are used to assess tumor size and lymph node status in FIGO staging. These tools provide detailed information about the extent of the cancer and help determine the appropriate treatment plan. | A | B | B |
| Microsoft Phi2 | 68. Imaging tools such as ultrasound and MRI are used to assess tumor size and lymph node status in FIGO staging. These tools are recommended for patients with FIGO stage IA1 cervical cancer. | C | C | C |
| QiZhenGPT (With prompt) | 68. How are imaging tools used to assess tumor size and lymph node status in FIGO staging? What specific imaging tools are recommended? Answer: Imaging tools such as CT and MRI are used to assess tumor size and lymph node status in FIGO staging. The specific imaging tools recommended may vary depending on the stage and location of the cancer. | B | B | B |
| QiZhenGPT | 68. Imaging tools such as computed tomography (CT) and magnetic resonance imaging (MRI) are used to assess tumor size and lymph node status in stage IA1 cervical cancer. | C | C | C |
| **69. In cervical cancer treatment, if the patient does not wish to preserve fertility, and the stage is ⅠA1 without lymphovascular space invasion, what treatment measures should be taken?** | | | | |
| ChatGPT4.0 turbo (With prompt) | 69. For cervical cancer at stage IA1 without lymphovascular space invasion (LVSI), the standard treatment option is a simple hysterectomy. This involves the removal of the uterus but typically spares the ovaries and fallopian tubes, assuming there is no other indication for their removal. This approach is sufficient as the risk of recurrence is very low when LVSI is absent. | A | A | A |
| ChatGPT4.0 turbo | 69. Stage IA1 without LVSI: The standard treatment for stage IA1 cervical cancer without LVSI is a hysterectomy, which is the surgical removal of the uterus. Since fertility preservation is not a concern, a simple hysterectomy (removal of the uterus but not the surrounding tissues) may be sufficient. | B | A | B |
| Claude 2 (With prompt) | 69. For stage IA1 cervical cancer without lymphovascular invasion in patients who do not desire fertility preservation, recommended treatment is a simple or extrafascial hysterectomy. | A | A | A |
| Claude 2 | 69. For stage IA1 without lymphovascular space invasion, either a simple hysterectomy or cone biopsy is recommended. | A | A | A |
| Gemini Pro (With prompt) | 69. Radical hysterectomy with pelvic lymphadenectomy | A | A | A |
| Gemini Pro | 69. **Stage ⅠA1 without lymphovascular space invasion and no desire to preserve fertility**:  - **Radical hysterectomy** (removal of the uterus, cervix, and upper part of the vagina) with **pelvic lymph node dissection**. | C | C | C |
| Mistral-7B-v0.2 (With prompt) | 69.In cervical cancer treatment, if the patient does not wish to preserve fertility and the stage is ⅠA1 without lymphovascular space invasion, a hysterectomy (removal of the uterus and cervix) is the recommended treatment. Radiation therapy may also be considered as an alternative. | B | B | B |
| Mistral-7B-v0.2 | 69. For cervical cancer patients without lymphovascular space invasion (LVSI) and stage ⅠA1 who do not wish to preserve fertility, the standard treatment is usually radical hysterectomy (simple hysterectomy or radical trachelectomy) with pelvic lymph node dissection. This approach aims to remove the cancer and surrounding tissue, including the uterus and cervix, to prevent recurrence. | C | C | C |
| Starling-LM-7B alpha (With prompt) | 69. In cervical cancer treatment, if the patient does not wish to preserve fertility, and the stage is ⅠA1 without lymphovascular space invasion, what treatment measures should be taken? 2.In cervical cancer treatment, if the patient does not wish to preserve fertility, and the stage is ⅠA1 with lymphovascular space invasion, what treatment measures should be taken? 3.In cervical cancer treatment, if the patient does not wish to preserve fertility, and the stage is ⅠA2, what treatment measures should be taken? 4.In cervical cancer treatment, if the patient does not wish to preserve fertility, and the stage is ⅠB1, ⅡA1, or ⅡB2, what treatment measures should be taken? 5.In cervical cancer treatment, if the patient does not wish to preserve fertility, and the stage is ⅠB3 or ⅡA2, what treatment measures should be taken?<\|end_of_turn\|>GPT4 Correct Assistant: 1. In cervical cancer treatment, if the patient does not wish to preserve fertility and the stage is ⅠA1 without lymphovascular space invasion, the recommended treatment measures are total hysterectomy, including removal of the cervix, uterus, and upper vagina, along with pelvic and para-aortic lymph node dissection. Radiation therapy, either external beam radiation therapy (EBRT) or brachytherapy, can be considered as an alternative to surgery in select cases. | C | C | C |
| Starling-LM-7B alpha | 69. For a patient with stage ⅠA1 cervical cancer who does not wish to preserve fertility, the recommended treatment is a radical hysterectomy, which involves the removal of the uterus, cervix, and surrounding tissues, along with pelvic lymph node dissection. Radiation therapy may also be considered as an alternative treatment option. | D | D | D |
| HuatuoGPT (With prompt) | 69. In the treatment of cervical cancer, if the patient does not wish to preserve fertility and is in stage IA1 with no lymph node vascular invasion, what are the next therapeutic steps that should be taken? Answer: For patients with stage IA1 cervical cancer who do not wish to preserve fertility, surgical treatment, i.e. hysterectomy (including cervix) and lymph node dissection, is usually adopted. | C | C | C |
| HuatuoGPT | 69. If the patient does not wish to preserve fertility and is in stage IA1 with no lymphatic emboli, the conventional treatment is radical hysterectomy and pelvic lymph node dissection. Radical hysterectomy involves removal of the cervix and upper cervical tissues, including the endocervix, uterine body and cervical portion. This ensures complete removal of the lesion and reduces the risk of recurrence. Pelvic lymph node dissection is performed to determine the presence of lymph node metastases to guide subsequent treatment strategies. | C | D | D |
| BioMedLM 2.7B (With prompt) | 69. In cervical cancer treatment, if the patient does not wish to preserve fertility, and the stage is ⅠA1 without lymphovascular space invasion, what treatment measures should be taken? The patient should be treated with radical hysterectomy and pelvic lymphadenectomy. | C | C | C |
| BioMedLM 2.7B | 69. In cervical cancer treatment, if the patient does not wish to preserve fertility, and the stage is ⅠA1 without lymphovascular space invasion, what treatment measures should be taken? ### Response: Surgery + chemotherapy Surgery + radiotherapy Surgery + chemotherapy + radiotherapy. | D | D | D |
| Microsoft Phi2 (With prompt) | 69. In cervical cancer treatment, if the patient does not wish to preserve fertility, and the stage is ⅠA1 without lymphovascular space invasion, the treatment measures that should be taken include radical hysterectomy, bilateral salpingo-oophorectomy, and pelvic lymphadenectomy. | C | C | C |
| Microsoft Phi2 | 69. If the patient does not wish to preserve fertility, and the stage is ⅠA1 without lymphovascular space invasion, the treatment measures that should be taken are radical hysterectomy and pelvic lymphadenectomy. | C | C | C |
| QiZhenGPT (With prompt) | 69. In cervical cancer treatment, if the patient does not wish to preserve fertility, and the stage is IA1 without lymphovascular space invasion, what treatment measures should be taken? The patient should receive a combination of surgery, chemotherapy, and radiation therapy to treat the cancer. | D | D | D |
| QiZhenGPT | 69. In the treatment of cervical cancer, if the patient does not wish to preserve fertility and the stage is stage IA1 with no lymphovascular space invasion, what is the therapeutic measure to be taken? In this case, surgical treatment, possibly total hysterectomy or bilateral adnexectomy, and lymph node dissection is usually indicated. In addition, chemotherapy may be given to kill any remaining cancer cells. The exact treatment plan should be decided by the doctor on a patient-by-patient basis. | D | C | C |
| **70.In cervical cancer treatment, if the patient does not wish to preserve fertility, and the stage is ⅠA1 with lymphovascular space invasion, what treatment measures should be taken?** | | | | |
| ChatGPT4.0 turbo (With prompt) | 70. In cases of stage IA1 cervical cancer with lymphovascular space invasion, the recommended treatment is a radical hysterectomy. This procedure includes the removal of the uterus, the tissue around the cervix (parametrium), and the upper part of the vagina. This is due to the increased risk of spread associated with LVSI. Pelvic lymphadenectomy may also be considered to assess the extent of disease spread and further tailor adjuvant treatments if necessary. | B | B | B |
| ChatGPT4.0 turbo | 70. Stage IA1 with LVSI: For stage IA1 cervical cancer with LVSI, a more radical hysterectomy may be recommended due to the higher risk of cancer spread. A radical hysterectomy involves the removal of the uterus along with some surrounding tissue, the upper part of the vagina, and possibly the lymph nodes. | B | B | B |
| Claude 2 (With prompt) | 70. For IA1 disease with lymphovascular invasion, treatment is a radical hysterectomy and pelvic lymph node dissection given the higher risk of nodal metastases. | A | B | A |
| Claude 2 | 70. For stage IA1 with lymphovascular space invasion, a radical hysterectomy with pelvic lymph node dissection is recommended. | C | C | C |
| Gemini Pro (With prompt) | 70. Radical hysterectomy with pelvic lymphadenectomy and adjuvant therapy | B | B | B |
| Gemini Pro | 70. **Stage ⅠA1 with lymphovascular space invasion and no desire to preserve fertility**:  - **Radical hysterectomy** with **pelvic and para-aortic lymph node dissection**. | C | C | C |
| Mistral-7B-v0.2 (With prompt) | 70.In cervical cancer treatment, if the patient does not wish to preserve fertility and the stage is ⅠA1 with lymphovascular space invasion, a radical hysterectomy (removal of the uterus, cervix, and surrounding tissue) with pelvic lymph node dissection is the recommended treatment. Radiation therapy may also be considered as an alternative. | B | B | B |
| Mistral-7B-v0.2 | 70. For patients with LVSI and stage ⅠA1 who do not wish to preserve fertility, the best treatment option would typically be radical hysterectomy or radical trachelectomy with pelvic and/or para-aortic lymph node dissection. This more extensive surgery aims to address the potential increased risk of metastasis associated with LVSI. | C | C | C |
| Starling-LM-7B alpha (With prompt) | 70. In cervical cancer treatment, if the patient does not wish to preserve fertility and the stage is ⅠA1 with lymphovascular space invasion, the recommended treatment measures are similar to those for stage ⅠA1 without lymphovascular space invasion. However, due to the increased risk of lymph node involvement, adjuvant chemotherapy may be considered in addition to the primary treatment modality. | D | C | C |
| Starling-LM-7B alpha | 70. For a patient with stage ⅠA1 cervical cancer with lymphovascular space invasion who does not wish to preserve fertility, the recommended treatment is the same as for stage ⅠA1 without lymphovascular space invasion, which is a radical hysterectomy and pelvic lymph node dissection. Radiation therapy may also be considered as an alternative treatment option. | C | C | C |
| HuatuoGPT (With prompt) | 70. In the treatment of cervical cancer, if the patient does not want to preserve fertility and is in stage IA1 with lymph node vascular invasion, what are the next therapeutic measures to be taken? Answer: For patients with stage IA1 cervical cancer who do not want to preserve fertility, comprehensive treatment including surgery, radiotherapy and chemotherapy is usually adopted. The specific treatment plan needs to be determined according to the specific conditions of patients. | D | D | D |
| HuatuoGPT | 70. Radical hysterectomy with pelvic lymph node dissection is also an option if the patient does not wish to preserve fertility and is in stage IA1 with lymphatic emboli. Surgical resection can completely remove the lesion, and pelvic lymph node dissection can help to determine whether there are lymph node metastases. | D | D | D |
| BioMedLM 2.7B (With prompt) | 70. In cervical cancer treatment, if the patient does not wish to preserve fertility, and the stage is ⅠA1 with lymphovascular space invasion, what treatment measures should be taken? Radical hysterectomy (RH) + pelvic lymph node dissection (PLND) + para-aortic lymph node dissection (PALND) + para-cervical lymph node dissection (PCLND) + omentectomy + appendectomy + peritoneal cytology + peritoneal biopsy | C | C | C |
| BioMedLM 2.7B | 70. ### Response: Radical hysterectomy (RH) + pelvic lymph node dissection (PLND) + para-aortic lymph node dissection (PALND) + para-cervical lymph node dissection (PCND) + omentectomy + peritoneal cytology (PC) + peritoneal biopsy (PB) + para-aortic lymph node biopsy (PALNB) + para-cervical lymph node biopsy (PCNB) + peritoneal washing cytology (PWC) + peritoneal biopsy (PB) | C | C | C |
| Microsoft Phi2 (With prompt) | 70. In cervical cancer treatment, if the patient does not wish to preserve fertility, and the stage is ⅠA1 with lymphovascular space invasion, the treatment measures that should be taken include radical hysterectomy with bilateral salpingo-oophorectomy, pelvic lymphadenectomy, and radiation therapy. | B | B | B |
| Microsoft Phi2 | 70. If the patient does not wish to preserve fertility, and the stage is ⅠA1 with lymphovascular space invasion, the treatment measures that should be taken are radical hysterectomy, pelvic lymphadenectomy, and pelvic lymphadenectomy with pelvic lymphadenectomy. | C | D | D |
| QiZhenGPT (With prompt) | 70. In cervical cancer treatment, if the patient does not wish to preserve fertility, and the stage is IA1 with lymphovascular space invasion, the treatment measures for this stage should focus on providing supportive care, such as radiation therapy and/or chemotherapy, to manage the symptoms and improve the patient's quality of life. | D | D | D |
| QiZhenGPT | 70. In the treatment of cervical cancer, if the patient does not wish to preserve fertility and has stage IA1 with lymphovascular space invasion, what are the therapeutic measures to be taken? A. For cervical cancer patients with stage IA1 and lymphovascular space invasion, treatment measures usually include surgery, radiotherapy and chemotherapy. Surgery may be extensive resection or sub-extensive resection, radiotherapy may be external irradiation or brachytherapy, and chemotherapy mainly uses paclitaxel, etc. The treatment plan needs to be based on the patient's specific situation. The treatment plan needs to be formulated according to the patient's specific situation and carried out under the supervision of the doctor. | D | D | D |
| **71. In cervical cancer treatment, if the patient does not wish to preserve fertility, and the stage is ⅠA2, what treatment measures should be taken?** | | | | |
| ChatGPT4.0 turbo (With prompt) | 71. For stage IA2 cervical cancer, the treatment recommendation is a radical hysterectomy along with pelvic lymphadenectomy. The increased depth of invasion associated with stage IA2 necessitates a more extensive surgical approach compared to stage IA1, as there is a higher risk of microscopic spread which could involve the lymph nodes. | B | B | B |
| ChatGPT4.0 turbo | 71. Stage IA2: In stage IA2, a radical hysterectomy with or without removal of pelvic lymph nodes is usually recommended. Alternatively, primary radiation therapy may be considered, especially if the patient has other health issues making surgery less safe. | B | B | B |
| Claude 2 (With prompt) | 71. In stage IA2 patients not wishing to preserve fertility, standard treatment is a radical hysterectomy and pelvic lymphadenectomy. Ovarian transposition can be considered to spare ovaries. | B | B | B |
| Claude 2 | 71. For stage IA2, a radical hysterectomy and pelvic lymph node dissection is typically recommended. | B | B | B |
| Gemini Pro (With prompt) | 71. Radical hysterectomy with pelvic lymphadenectomy and adjuvant therapy | B | B | B |
| Gemini Pro | 71. **Stage ⅠA2 and no desire to preserve fertility**:  - **Radical hysterectomy** with **pelvic and para-aortic lymph node dissection**. | B | B | B |
| Mistral-7B-v0.2 (With prompt) | 71.In cervical cancer treatment, if the patient does not wish to preserve fertility and the stage is ⅠA2, a radical hysterectomy with pelvic lymph node dissection is the recommended treatment. Radiation therapy may also be considered as an alternative. | A | A | A |
| Mistral-7B-v0.2 | 71. In the ⅠA2 stage without LVSI, without the desire for fertility preservation, the preferred treatment is usually radical hysterectomy and pelvic lymph node dissection. In some cases, external beam radiation therapy combined with brachytherapy (internal radiation) may also be considered. | A | A | A |
| Starling-LM-7B alpha (With prompt) | 71. In cervical cancer treatment, if the patient does not wish to preserve fertility and the stage is ⅠA2, the recommended treatment measures are radical trachelectomy with pelvic lymph node dissection. This procedure involves the removal of the cervix, upper vagina, and regional lymph nodes while preserving the uterus and ovaries. Radiation therapy, either EBRT or brachytherapy, can be considered as an alternative to surgery in select cases. | A | A | A |
| Starling-LM-7B alpha | 71. For a patient with stage ⅠA2 cervical cancer who does not wish to preserve fertility, the recommended treatment is a radical trachelectomy, which involves the removal of the cervix and upper part of the vagina, along with pelvic lymph node dissection. Radiation therapy may also be considered as an alternative treatment option. | B | B | B |
| HuatuoGPT (With prompt) | 71. In the treatment of cervical cancer, if the patient does not want to preserve fertility and is in stage IA2, what treatment measures should be taken next? Answer: For patients with stage IA2 cervical cancer who do not wish to preserve fertility, a combination of treatments, including surgery, radiotherapy and chemotherapy, is usually adopted. The specific treatment plan needs to be determined according to the specific conditions of patients. | D | D | D |
| HuatuoGPT | 71. Radical hysterectomy and pelvic lymph node dissection may also be considered if the patient does not wish to preserve fertility and is at stage IA2. Stage IA2 indicates that the tumor is above the endocervical os but less than the upper 1/3 of the cervix and that there are no lymphatic vessels with cancerous emboli. Surgical resection allows complete removal of the lesion, and pelvic lymph node dissection helps to determine the presence of lymph node metastases. | B | B | B |
| BioMedLM 2.7B (With prompt) | 71. In cervical cancer treatment, if the patient does not wish to preserve fertility, and the stage is ⅠA2, what treatment measures should be taken?  Radical hysterectomy | C | B | C |
| BioMedLM 2.7B | 71. In cervical cancer treatment, if the patient does not wish to preserve fertility, and the stage is ⅠA2, what treatment measures should be taken? ### Response: 1\. Surgery.2Surgery + chemotherapy.3 Surgery + radiotherapy.4 Surgery + chemotherapy + radiotherapy. | C | C | C |
| Microsoft Phi2 (With prompt) | 71. In cervical cancer treatment, if the patient does not wish to preserve fertility, and the stage is ⅠA2, the treatment measures that should be taken include radical hysterectomy, bilateral salpingo-oophorectomy, pelvic lymphadenectomy, and radiation therapy. | A | A | A |
| Microsoft Phi2 | 71. If the patient does not wish to preserve fertility, and the stage is ⅠA2, the treatment measures that should be taken are radical hysterectomy, pelvic lymphadenectomy, and pelvic lymphadenectomy. | B | B | B |
| QiZhenGPT (With prompt) | 35. In cervical cancer treatment, if the patient does not wish to preserve fertility, and the stage is IA2, what treatment measures should be taken? The patient should be referred to a specialist for further evaluation and treatment planning. The treatment options may include surgery, radiation therapy, and/or chemotherapy. The patient should also be informed about the potential risks and benefits of each treatment option and have a discussion with their healthcare provider about their individual needs and preferences. In addition, the patient may be eligible for a Cervical Cancer Screening Program, which can help reduce the risk of developing cervical cancer by detecting early-stage cases. | D | D | D |
| QiZhenGPT | 71. In the treatment of cervical cancer, if the patient does not want to preserve fertility and the stage is ⅠA2, what is the treatment measure that should be taken? In this case, surgical treatment such as total hysterectomy or bilateral adnexectomy and lymph node dissection may be required. Chemotherapy may also be given to kill any remaining cancer cells. The exact treatment plan should be decided by the doctor based on the patient's specific situation. | A | A | A |
| **72. In cervical cancer treatment, if the patient does not wish to preserve fertility, and the stage is ⅠB1, ⅡA1, or ⅡB2, what treatment measures should be taken?** | | | | |
| ChatGPT4.0 turbo (With prompt) | 72. When treating stages IB1, IIA1, or IIB2 cervical cancer, and fertility preservation is not a concern, the standard of care includes radical hysterectomy with bilateral pelvic lymphadenectomy. Additionally, depending on the size of the lesion and other risk factors, chemoradiation may be recommended, particularly for stages IIB2 where the tumor is larger and may involve nearby tissues more extensively. | A | A | A |
| ChatGPT4.0 turbo | 72. Stage IB1, IIA1, or IIB2: For these stages, the treatment typically involves either a radical hysterectomy with removal of pelvic lymph nodes or radiotherapy (external beam radiation therapy plus brachytherapy) with concurrent chemotherapy. The choice between surgery and radiotherapy may depend on the size of the tumor, the patient's preference, and the presence of any other medical conditions. | A | B | A |
| Claude 2 (With prompt) | For stage IB1, IIA1, or IIB2 cervical cancer when fertility preservation is not desired, the standard treatment is radical hysterectomy with bilateral pelvic lymph node dissection. Chemotherapy and/or radiation may be recommended after surgery depending on pathologic risk factors. | A | A | A |
| Claude 2 | 72. For stages IB1, IIA1, and IIB2, a radical hysterectomy and pelvic lymph node dissection with or without postoperative radiation therapy is usually recommended. | B | B | B |
| Gemini Pro (With prompt) | 72. Radical hysterectomy with pelvic and para-aortic lymphadenectomy and adjuvant therapy | C | C | C |
| Gemini Pro | 72. **Stage ⅠB1, ⅡA1, or ⅡB2 and no desire to preserve fertility**:  - **Concurrent chemoradiation**: External beam radiation therapy (EBRT) to the pelvis and para-aortic lymph node regions, combined with concurrent chemotherapy (usually with cisplatin). | B | B | B |
| Mistral-7B-v0.2 (With prompt) | 72.In cervical cancer treatment, if the patient does not wish to preserve fertility and the stage is ⅠB1, ⅡA1, or ⅡB2, a radical hysterectomy with pelvic lymph node dissection is the recommended treatment. Radiation therapy may also be considered as an alternative, or a combination of both treatments may be used. | B | B | B |
| Mistral-7B-v0.2 | 72. For stage ⅠB1, ⅡA1, and ⅡB2 cervical cancer patients who do not wish to preserve fertility, the standard treatment is typically external beam radiation therapy with intracavitary brachytherapy, combined with chemotherapy. This approach aims to destroy cancer cells using radiation and reduce the risk of recurrence. | B | A | B |
| Starling-LM-7B alpha (With prompt) | 72. In cervical cancer treatment, if the patient does not wish to preserve fertility and the stage is ⅠB1, ⅡA1, or ⅡB2, the recommended treatment measures are radical hysterectomy with pelvic and para-aortic lymph node dissection. Radiation therapy, either EBRT or brachytherapy, can be considered as an alternative to surgery in select cases. In cases where fertility preservation is not a concern, chemotherapy may be added to radiation therapy as adjuvant treatment. | B | B | B |
| Starling-LM-7B alpha | 72. For a patient with stage ⅠB1, ⅡA1, or ⅡB2 cervical cancer who does not wish to preserve fertility, the recommended treatment is a radical hysterectomy, pelvic lymph node dissection, and para-aortic lymph node dissection. Radiation therapy, including external beam radiation therapy (EBRT) and brachytherapy, may also be considered as an alternative treatment option. | B | B | B |
| HuatuoGPT (With prompt) | 72. In the treatment of cervical cancer, if patients do not want to preserve fertility and are in stage ⅠB1, ⅡA1 or ⅡB2, what are the next therapeutic measures to be taken? Answer: For stage ⅠB1, ⅡA | D | D | D |
| HuatuoGPT | 72. If the patient does not wish to preserve fertility and is at stage IB1, IIA1 or IIB2, the treatment remains radical hysterectomy and pelvic lymph node dissection. These stages indicate that the tumor has begun to invade the upper third of the cervix or deep into the uterine wall, but has not yet spread to the pelvic sidewalls or lower third, or has only spread to the lower third of the pelvic sidewalls. surgical excision can completely remove the lesion, and pelvic lymph node dissection can help to determine the presence of lymph node metastases, which guides the subsequent therapeutic strategy. | B | B | B |
| BioMedLM 2.7B (With prompt) | 72. In cervical cancer treatment, if the patient does not wish to preserve fertility, and the stage is ⅠB1, ⅡA1, or ⅡB2, what treatment measures should be taken?  Surgery:  Radical hysterectomy (RH)  Radical trachelectomy (RT)  Radical trachelectomy with pelvic lymphadenectomy (RT+PL)  Radical trachelectomy with para-aortic lymphadenectomy (RT+PAL)  Radical trachelectomy with pelvic and para-aortic lymphadenectomy (RT+PL+PAL) | C | C | C |
| BioMedLM 2.7B | 72. In cervical cancer treatment, if the patient does not wish to preserve fertility, and the stage is ⅠB1, ⅡA1, or ⅡB2, what treatment measures should be taken? ### Response: 1\. Surgery.2. Surgery + chemotherapy.3. Surgery + radiotherapy.4. Surgery + chemotherapy + radiotherapy. | C | C | C |
| Microsoft Phi2 (With prompt) | 72. In cervical cancer treatment, if the patient does not wish to preserve fertility, and the stage is ⅠB1, ⅡA1, or ⅡB2, the treatment measures that should be taken include radical hysterectomy, bilateral salpingo-oophorectomy, pelvic lymphadenectomy, radiation therapy, and chemotherapy. | A | A | A |
| Microsoft Phi2 | 72. If the patient does not wish to preserve fertility, and the stage is ⅠB1, ⅡA1, or ⅡB2, the treatment measures that should be taken are radical hysterectomy, pelvic lymphadenectomy, pelvic lymphadenectomy, and pelvic lymphadenectomy. | B | B | B |
| QiZhenGPT (With prompt) | 72. In cervical cancer treatment, if the patient does not wish to preserve fertility, and the stage is IB1, IIA1, or IIB2, what treatment measures should be taken? The patient should be referred to a specialist for further evaluation and treatment planning. The treatment options may include surgery, radiation therapy, and/or chemotherapy. The patient should also be informed about the potential risks and benefits of each treatment option and have a discussion with their healthcare provider about their individual needs and preferences. In addition, the patient may be eligible for a Cervical Cancer Screening Program, which can help reduce the risk of developing cervical cancer by detecting early-stage cases. | C | C | C |
| QiZhenGPT | 72. In the treatment of cervical cancer, if the patient does not wish to preserve fertility and stage ⅠB1, ⅡA1 or ⅡB2, what are the treatment measures to be taken? In this case, surgical treatment, such as total hysterectomy or bilateral adnexectomy, and lymph node dissection may be required. Chemotherapy may also be given to kill any remaining cancer cells. The exact treatment plan should be decided by the doctor based on the patient's specific situation. | A | A | A |
| **73. In cervical cancer treatment, if the patient does not wish to preserve fertility, and the stage is ⅠB3 or ⅡA2, what treatment measures should be taken?** | | | | |
| ChatGPT4.0 turbo (With prompt) | 73. For stages IB3 or IIA2 cervical cancer, the preferred treatment approach is chemoradiation. This combines chemotherapy with radiation therapy to maximize tumor control, as these stages indicate larger or more invasive tumors that are less likely to be managed by surgery alone. Concurrent chemoradiation has been shown to improve outcomes in terms of both local control and survival for these advanced stages. | A | A | A |
| ChatGPT4.0 turbo | 73. Stage IB3 or IIA2: For stage IB3 or IIA2 cervical cancer, chemoradiation (a combination of chemotherapy and radiation therapy) is generally the preferred treatment. Surgery is not typically the firstline treatment due to the size of the tumor and the increased risk of spread to nearby tissues. | A | A | A |
| Claude 2 (With prompt) | 73. In patients with stage IB3 or IIA2 cervical cancer who do not desire fertility preservation, the standard recommendation is definitive concurrent chemoradiation as for IB2 disease. This includes external pelvic radiation, cisplatin chemotherapy, plus image-guided brachytherapy.  # CONTEXT # Now you are a gynecologist with over 20 years of experience in medicine and you are answering questions about the medical specialty of cervical cancer treatment, diagnosis, and screening # OBJECTIVE # Please answer the following questions correctly and in strict accordance with the latest guidelines for the screening, treatment, and diagnosis of cervical cancer # STYLE # Please follow the style of a gynecologist in the field of medicine # TONE # Formal, academic, professional # RESPONSE # Answer each of the five questions in each group, with the answer number matching the question number. | A | A | A |
| Claude 2 | 73. For stages IB3 and IIA2, primary chemoradiation (radiation therapy with concurrent chemotherapy) is generally the standard treatment. An radical hysterectomy may also be considered after chemoradiation if residual disease is still suspected. | A | A | A |
| Gemini Pro (With prompt) | 73. Concurrent chemoradiotherapy | A | B | B |
| Gemini Pro | 73. **Stage ⅠB3 or ⅡA2 and no desire to preserve fertility**:  - **Neoadjuvant chemotherapy** followed by **radical hysterectomy** with **pelvic and para-aortic lymphadenectomy**. Neoadjuvant chemotherapy aims to shrink the tumor and improve the chances of successful surgical resection. | A | A | A |
| Mistral-7B-v0.2 (With prompt) | 73.In cervical cancer treatment, if the patient does not wish to preserve fertility and the stage is ⅠB3 or ⅡA2, radiation therapy is the recommended primary treatment. Chemotherapy may also be considered as an adjuvant treatment. A hysterectomy may be considered as a treatment option after completion of radiation therapy or chemotherapy. | A | A | A |
| Mistral-7B-v0.2 | 73. In cases of stage ⅠB3 or ⅡA2 cervical cancer patients who do not wish to preserve fertility, advanced radiotherapy techniques, such as intensity-modulated radiation therapy (IMRT) or image-guided intensity-modulated radiation therapy (IG-IMRT), combined with chemotherapy, are the primary options. These advanced radiation techniques aim to more effectively target the cancer while minimizing damage to surrounding healthy tissue. A radical hysterectomy and lymph node dissection may be considered if the cancer is not growing too rapidly. The choice of treatment method depends on the individual circumstances of each patient and should be discussed with the healthcare team. | B | B | B |
| Starling-LM-7B alpha (With prompt) | 73. In cervical cancer treatment, if the patient does not wish to preserve fertility and the stage is ⅠB3 or ⅡA2, the recommended treatment measures are similar to those for stage ⅠB1, ⅡA1, or ⅡB2. However, due to the advanced stage and increased risk of lymph node involvement, adjuvant chemotherapy may be considered in addition to the primary treatment modality.  It is important to note that these recommendations are based on the latest guidelines and may vary depending on the patient's individual circumstances, overall health, and preferences. A multidisciplinary team of specialists, including a gynecologic oncologist, should be involved in the decision-making process to ensure the most appropriate treatment plan is chosen for each patient. | B | B | B |
| Starling-LM-7B alpha | 73. For a patient with stage ⅠB3 or ⅡA2 cervical cancer who does not wish to preserve fertility, the recommended treatment is a combination of chemotherapy and radiation therapy, known as chemoradiation. This treatment involves EBRT and brachytherapy, along with chemotherapy drugs such as cisplatin. Surgery is generally not recommended in these cases, as the tumor has a higher risk of spreading beyond the cervix. | A | A | A |
| HuatuoGPT (With prompt) | 73. Not answered | D | D | D |
| HuatuoGPT | 73. Radical hysterectomy and pelvic lymph node dissection may also be considered if the patient does not wish to preserve fertility and is at stage IB3 or IIA2. These stages indicate that the tumor has invaded the lower 2/3 of the cervix or penetrated deep into the uterine wall, but has not spread to the lateral pelvic wall. Surgical excision can completely remove the lesion, and pelvic lymph node dissection can help determine the presence of lymph node metastases to guide subsequent treatment strategies | B | B | B |
| BioMedLM 2.7B (With prompt) | 73. In cervical cancer treatment, if the patient does not wish to preserve fertility, and the stage is ⅠB3 or ⅡA2, what treatment measures should be taken? Radical hysterectomy (RH) + pelvic lymph node dissection (PLND) + para-aortic lymph node dissection (PALND) RH + PLND + PALND + chemotherapy RH + PLND + PALND + radiotherapy | D | D | D |
| BioMedLM 2.7B | 73. In cervical cancer treatment, if the patient does not wish to preserve fertility, and the stage is ⅠB3 or ⅡA2, what treatment measures should be taken? ### Response: 1\ Surgery.2Chemotherapy.3 Radiotherapy4 Hysterectomy. | B | B | B |
| Microsoft Phi2 (With prompt) | 73. In cervical cancer treatment, if the patient does not wish to preserve fertility, and the stage is ⅠB3 or ⅡA2, the treatment measures that should be taken include radical hysterectomy with bilateral salpingo-oophorectomy, pelvic lymphadenectomy, and radiation therapy. | D | D | D |
| Microsoft Phi2 | 73. If the patient does not wish to preserve fertility, and the stage is ⅠB3 or ⅡA2, the treatment measures that should be taken are radical hysterectomy, pelvic lymphadenectomy, pelvic lymphadenectomy, pelvic lymphadenectomy, and pelvic lymphadenectomy. | C | C | C |
| QiZhenGPT (With prompt) | 73. In cervical cancer treatment, if the patient does not wish to preserve fertility, and the stage is IB3 or IIA2, what treatment measures should be taken? The patient should be referred to a specialist for further evaluation and treatment planning. The treatment options may include surgery, radiation therapy, and/or chemotherapy. The patient should also be informed about the potential risks and benefits of each treatment option and have a discussion with their healthcare provider about their individual needs and preferences. In addition, the patient may be eligible for a Cervical Cancer Screening Program, which can help reduce the risk of developing cervical cancer by detecting early-stage cases. | C | C | C |
| QiZhenGPT | 73. In the treatment of cervical cancer, if the patient does not wish to preserve fertility and stage ⅠB3 or stage ⅡA2, what are the treatment measures that should be taken? For patients with stage ⅠB3 or ⅡA2 of cervical cancer, if the patients do not wish to preserve fertility, the treatment measures usually include surgery, radiotherapy or chemotherapy, and the specific choices depend on the patients' individual conditions. | B | B | B |
| **74. In cervical cancer treatment, if the patient wishes to preserve fertility, and the stage is ⅠA1 without lymphovascular space invasion, what treatment measures should be taken?** | | | | |
| ChatGPT4.0 turbo (With prompt) | 74. For a patient with stage IA1 cervical cancer without lymphovascular space invasion who wishes to preserve fertility, a conservative surgical option such as a cone biopsy or a large loop excision of the transformation zone (LLETZ) can be considered. These procedures aim to remove the cancerous lesion while maintaining the structural integrity of the cervix, thereby preserving the patient’s fertility. | A | A | A |
| ChatGPT4.0 turbo | 74. Stage IA1 without LVSI: For this early stage of cervical cancer without lymphovascular space invasion, a conservative surgical approach such as a cone biopsy or a large loop excision of the transformation zone (LLETZ) may be considered. These procedures remove only the cancerous tissue and a margin of healthy tissue while preserving the uterus and the potential for future pregnancy. | A | A | A |
| Claude 2 (With prompt) | 74. For stage IIIC1 cervical cancer, I would typically recommend a combination of chemotherapy with cisplatin and radiation therapy, followed by brachytherapy. Surgical resection may be considered if the tumor size is amenable. The 5-year survival rate with aggressive treatment is approximately 35-40%. | A | B | A |
| Claude 2 | 74. For stage IA1 without lymphovascular space invasion: Conization (cone biopsy) alone is reasonable to preserve fertility. | C | C | C |
| Gemini Pro (With prompt) | 74. Conization | B | B | B |
| Gemini Pro | 74. **Stage ⅠA1 without lymphovascular space invasion and desire to preserve fertility**:  - **Conization with negative margins**: Removal of a cone-shaped portion of the cervix to remove the cancerous tissue while preserving the uterus. Regular follow-up and monitoring are crucial to detect any recurrence. | A | A | A |
| Mistral-7B-v0.2 (With prompt) | 74.In cervical cancer treatment, if the patient wishes to preserve fertility and the stage is ⅠA1 without lymphovascular space invasion, a cone biopsy (also known as a LEEP or loop electrosurgical excision procedure) is the recommended treatment. This procedure removes the abnormal tissue while preserving as much healthy tissue as possible. | A | A | A |
| Mistral-7B-v0.2 | 74. For cervical cancer patients with stage ⅠA1 and no lymphovascular space invasion who wish to preserve fertility, the recommended treatment is usually a trachelectomy or a radical trachelectomy. These procedures aim to preserve the cervix and the uterus while removing the cancer. However, it's essential to note that the decision to undergo these procedures depends on various factors, including the size, location, and type of the tumor. | B | B | B |
| Starling-LM-7B alpha (With prompt) | 74. For a patient with stage ⅠA1 cervical cancer who wishes to preserve fertility and there is no lymphovascular space invasion, the recommended treatment measures include:  a. Conization: This is a surgical procedure that removes the cancerous part of the cervix while preserving the healthy tissue. This is the preferred treatment option for patients who wish to preserve fertility. b. Radical trachelectomy: This is a more invasive surgical procedure that involves the removal of the cervix, upper vagina, and nearby lymph nodes. This procedure is typically reserved for patients with small, low-risk tumors. c. Close follow-up: Regular follow-up appointments with a gynecologist are essential to monitor for recurrence or new cancers. | C | C | C |
| Starling-LM-7B alpha | 74. For a patient with stage ⅠA1 cervical cancer who wishes to preserve fertility, the recommended treatment measures are:  - Conization: This is a surgical procedure that removes the cancerous part of the cervix while preserving the healthy tissue. This is usually performed under general anesthesia and can be done as an outpatient procedure.  - Radical trachelectomy: This is a more invasive surgical procedure that removes the cervix, upper vagina, and nearby lymph nodes. This procedure is typically reserved for patients with small, early-stage tumors who wish to preserve fertility. | C | B | C |
| HuatuoGPT (With prompt) | 74. If a patient with cervical cancer wants to preserve fertility and is in stage IA1 without lymphovascular invasion, what treatment measures should be taken? For patients with cervical cancer who wish to preserve fertility and are at stage IA1 without lymphovascular invasion, conization or hysterectomy may be considered. These procedures preserve the uterus and remove the cancerous cells from the cervix, thus allowing the patient to preserve fertility. | A | A | A |
| HuatuoGPT | 74. For the treatment of cervical cancer in which the patient wishes to preserve fertility and is in stage IA1 without lymph node space invasion, the following therapeutic measures are usually available. One common treatment is radical hysterectomy. In this procedure, the surgeon removes the patient's uterus, cervix, and nearby lymph nodes. However, in cases where fertility is preserved, the doctor may choose to keep the cervix and remove the tumor. The doctor may then perform an endocervical resection (conization) or a conization to remove the remaining diseased tissue. This will preserve as much of the patient's cervical function as possible and minimize the impact on fertility. Additionally, adjuvant radiotherapy may be an option for the treatment of cervical cancer. Adjuvant radiotherapy is usually used after surgery to kill remaining cancer cells to prevent the cancer from returning. For patients who wish to preserve their fertility, doctors may choose to use a lower dose of radiation therapy to minimize the impact on the ovaries and uterus. | A | A | A |
| BioMedLM 2.7B (With prompt) | 74. In cervical cancer treatment, if the patient wishes to preserve fertility, and the stage is ⅠA1 without lymphovascular space invasion, what treatment measures should be taken? Radical trachelectomy (RT) Radical hysterectomy (RH) Radical hysterectomy with pelvic lymph node dissection (RH+PLND) | D | D | D |
| BioMedLM 2.7B | 74. In cervical cancer treatment, if the patient wishes to preserve fertility, and the stage is ⅠA1 without lymphovascular space invasion, what treatment measures should be taken? ### Response: 1\. Radical trachelectomy (RT) 2\. Radical hysterectomy (RH) 3\. Radical trachelectomy (RT) + pelvic lymphadenectomy (PLND) 4\. Radical hysterectomy (RH) + pelvic lymphadenectomy (PLND) 5\. Radical hysterectomy (RH) + para-aortic lymphadenectomy (PALND) 6\. Radical hysterectomy (RH) + para-aortic lymphadenectomy (PALND) + pelvic lymphadenectomy (PLND) | D | D | D |
| Microsoft Phi2 (With prompt) | 74. In cervical cancer treatment, if the patient wishes to preserve fertility, and the stage is ⅠA1 without lymphovascular space invasion, the treatment measures that should be taken are radical trachelectomy, radical hysterectomy, and pelvic lymphadenectomy. | D | D | D |
| Microsoft Phi2 | 74. If the patient wishes to preserve fertility, and the stage is ⅠA1 without lymphovascular space invasion, the treatment measures that should be taken include surgery to remove the cancerous tissue, followed by radiation therapy to kill any remaining cancer cells. | C | B | C |
| QiZhenGPT (With prompt) | 74. In cervical cancer treatment, if the patient wishes to preserve fertility, and the stage is IA1 without lymphovascular space invasion, what treatment measures should be taken? The patient should be given a combination of surgery and radiation therapy to remove the cancer and its surrounding tissues, while also preserving their fertility. | D | C | C |
| QiZhenGPT | 74. In the treatment of cervical cancer, if the patient wishes to preserve fertility and has stage IA1 with no lymphovascular space invasion, the treatment measures to be adopted are: surgical treatment, radiotherapy and chemotherapy. Depending on the patient's specific situation, the doctor will formulate an individualized treatment plan. | C | C | C |
| **75. In cervical cancer treatment, if the patient wishes to preserve fertility, and the stage is ⅠA1 with lymphovascular space invasion or ⅠA2, what treatment measures should be taken?** | | | | |
| ChatGPT4.0 turbo (With prompt) | 75. In cases of stage IA1 cervical cancer with lymphovascular space invasion or stage IA2, where fertility preservation is desired, a more conservative approach than standard radical surgery is needed, but with careful consideration of the increased risk of recurrence. Options may include a trachelectomy (removal of the cervix while preserving the uterus) with pelvic lymphadenectomy. This allows for the preservation of the uterus for potential future pregnancies while addressing the risk posed by lymphovascular space invasion. | A | A | A |
| ChatGPT4.0 turbo | 75. Stage IA1 with LVSI or IA2: For stage IA1 with LVSI or stage IA2, fertilitysparing options may still be considered but with caution due to the higher risk of spread. A radical trachelectomy, which involves the removal of the cervix and surrounding tissues while preserving the uterus, may be an option for some patients. This procedure often includes a pelvic lymphadenectomy to check for the spread of cancer to the lymph nodes. | A | A | A |
| Claude 2 (With prompt) | 75. For stage IIIC2 cervical cancer, the standard of care is concurrent chemoradiation with cisplatin, followed by brachytherapy. If cancer is limited to the pelvis, pelvic exenteration may be an option for some patients. The prognosis is poorer than IIIC1, with 5-year survival around 15-20%. | A | A | A |
| Claude 2 | 75. For stage IA1 with lymphovascular space invasion or IA2: Radical trachelectomy (removal of cervix while preserving uterus) + pelvic lymphadenectomy is recommended. | A | A | A |
| Gemini Pro (With prompt) | 75. Radical trachelectomy with pelvic lymphadenectomy | A | A | A |
| Gemini Pro | 75. **Stage ⅠA1 with lymphovascular space invasion or ⅠA2 and desire to preserve fertility**:  - **Radical trachelectomy**: Removal of the cervix and upper part of the vagina, while preserving the uterus and fertility. Pelvic lymph node dissection is also performed. | A | A | A |
| Mistral-7B-v0.2 (With prompt) | 75.In cervical cancer treatment, if the patient wishes to preserve fertility and the stage is ⅠA1 with lymphovascular space invasion or ⅠA2, a cone biopsy followed by close monitoring with regular follow-up appointments and cervical cancer screening tests is recommended. If the abnormal cells persist or recur, more aggressive treatment options may be considered. | D | D | D |
| Mistral-7B-v0.2 | 75. For cervical cancer patients with stage ⅠA1 or ⅠA2 and lymphovascular space invasion or those with ⅠB1 disease who wish to preserve fertility, the recommended treatment is usually external beam radiation therapy (EBRT) or brachytherapy (internal radiation) with concurrent chemotherapy. This approach aims to destroy the cancer cells while minimizing damage to the surrounding healthy tissue, including the ovaries and uterus. However, the chances of preserving fertility are lower compared to earlier stages. | D | D | D |
| Starling-LM-7B alpha (With prompt) | 75. For a patient with stage ⅠA1 cervical cancer who wishes to preserve fertility and there is lymphovascular space invasion or stage ⅠA2, the recommended treatment measures include:  a. Radical trachelectomy: This is the preferred treatment option for patients with lymphovascular space invasion or stage ⅠA2 disease who wish to preserve fertility. b. Close follow-up: Regular follow-up appointments with a gynecologist are essential to monitor for recurrence or new cancers. | A | A | A |
| Starling-LM-7B alpha | 75. For a patient with stage ⅠA1 cervical cancer with lymphovascular space invasion or stage ⅠA2 who wishes to preserve fertility, the recommended treatment measures are:  - Radical trachelectomy: This is the preferred treatment option for patients with lymphovascular space invasion or stage ⅠA2 who wish to preserve fertility. This procedure removes the cervix, upper vagina, and nearby lymph nodes. | C | C | C |
| HuatuoGPT (With prompt) | In the treatment of cervical cancer, if patients wish to preserve fertility and are in stage IA1 with lymph node vascular space invasion or stage IA2, what treatment measures should be taken? For patients who wish to preserve fertility and are at stage IA1 or have lymph node vascular space invasion or stage IA2, the standard of care is to perform a radical hysterectomy and pelvic lymph node dissection without cervical resection or radiation therapy. The postoperative pathology report will determine the need for additional treatment, such as radiation or chemotherapy, depending on the specific stage of the cancer and other factors. In some cases, uterus-preserving surgeries such as hysterectomy or cervical cerclage may be considered if the patient wishes to preserve fertility. However, these procedures increase a patient's risk of complications and may not have as favorable an outcome as radical hysterectomy and pelvic lymph node dissection. Therefore, before making a decision, patients should discuss all treatment options and their pros and cons with their doctors. | A | A | A |
| HuatuoGPT | 75. If the patient wishes to preserve fertility and is in stage IA1 with lymphatic vessel emboli or stage IA2, the conventional treatment remains aggressive cancer therapy. This involves removal of the diseased tissue in the cervix by conization or loop electrosurgery (LEEP), followed by regular follow-up surveillance. If the lesion progresses or persists, further surgery or treatment, such as radiation therapy or chemotherapy, may be required to remove the diseased tissue and prevent disease progression. | C | C | C |
| BioMedLM 2.7B (With prompt) | 75. In cervical cancer treatment, if the patient wishes to preserve fertility, and the stage is ⅠA1 with lymphovascular space invasion or ⅠA2, what treatment measures should be taken? Radical trachelectomy (RT) . Radical hysterectomy (RH) . Radical hysterectomy with pelvic lymph node dissection (RH+PLND) . Radical hysterectomy with para-aortic lymph node dissection (RH+PALND) | D | D | D |
| BioMedLM 2.7B | 75. In cervical cancer treatment, if the patient wishes to preserve fertility, and the stage is ⅠA1 with lymphovascular space invasion or ⅠA2, what treatment measures should be taken? ### Response: 1 No treatment. 2 Radical trachelectomy. 3 Radical hysterectomy. 4 Radical trachelectomy with pelvic lymphadenectomy. | D | D | D |
| Microsoft Phi2 (With prompt) | 75. In cervical cancer treatment, if the patient wishes to preserve fertility, and the stage is ⅠA1 with lymphovascular space invasion or ⅠA2, the treatment measures that should be taken are radical trachelectomy, radical hysterectomy, pelvic lymphadenectomy, and pelvic lymphadenectomy. | C | C | C |
| Microsoft Phi2 | 75. If the patient wishes to preserve fertility, and the stage is ⅠA1 with lymphovascular space invasion or ⅠA2, the treatment measures that should be taken include surgery to remove the cancerous tissue, followed by radiation therapy to kill any remaining cancer cells. | C | C | C |
| QiZhenGPT (With prompt) | 75. In cervical cancer treatment, if the patient wishes to preserve fertility, and the stage is IA1 with lymphovascular space invasion or IA2, what treatment measures should be taken? The patient should be treated with a combination of surgery, chemotherapy, and radiation therapy to achieve complete response and preserve fertility. | C | C | C |
| QiZhenGPT | 75. In the treatment of cervical cancer, if the patient wishes to preserve fertility and the staging is IA1 with lymphovascular space invasion or IA2, the treatment measures may include: surgical treatment, radiotherapy and chemotherapy. Depending on the patient's specific situation, the doctor will develop an individualized treatment plan. | C | C | C |
| **76. In cervical cancer treatment, if the patient wishes to preserve fertility, and the stage is ⅠB1, what treatment measures should be taken?** | | | | |
| ChatGPT4.0 turbo (With prompt) | 76. For stage IB1 cervical cancer, fertility-sparing options include radical trachelectomy combined with pelvic lymphadenectomy. This procedure involves the removal of the cervix, part of the vagina, and surrounding tissues but leaves the uterus intact. This approach is suitable for tumors less than 2 cm in size and provides a balance between cancer control and the preservation of fertility. | A | A | A |
| ChatGPT4.0 turbo | 76. Stage IB1: Fertilitysparing treatment for stage IB1 may include radical trachelectomy with pelvic lymphadenectomy. This approach is generally considered for tumors less than 2 cm in size and with limited invasion depth. The eligibility for this procedure should be thoroughly discussed with a gynecologic oncologist. | A | A | A |
| Claude 2 (With prompt) | 76. For stage IVA cervical cancer without lymph node enlargement, recommended treatment is weekly cisplatin and radiation, possibly combined with hysterectomy if the tumor is resectable. The 5-year survival rate is between 15-35% depending on the extent of local vs distant metastasis. | B | B | B |
| Claude 2 | 76. For stage IB1: Radical trachelectomy + pelvic lymphadenectomy may be considered depending on tumor size and other factors. | A | A | A |
| Gemini Pro (With prompt) | 76. Radical trachelectomy with pelvic lymphadenectomy and adjuvant therapy | B | B | B |
| Gemini Pro | 76. **Stage ⅠB1 and desire to preserve fertility**:  - **Radical trachelectomy with pelvic lymph node dissection**. | A | B | A |
| Mistral-7B-v0.2 (With prompt) | 76.In cervical cancer treatment, if the patient wishes to preserve fertility and the stage is ⅠB1, a combination of radiation therapy and chemotherapy may be recommended. This approach can help preserve fertility while also effectively treating the cancer. | C | C | C |
| Mistral-7B-v0.2 | 76.not answered | D | D | D |
| Starling-LM-7B alpha (With prompt) | 76. For a patient with stage ⅠB1 cervical cancer who wishes to preserve fertility, the recommended treatment measures include:  a. Radical trachelectomy: This is the preferred treatment option for patients with stage ⅠB1 disease who wish to preserve fertility. b. Close follow-up: Regular follow-up appointments with a gynecologist are essential to monitor for recurrence or new cancers. | B | B | B |
| Starling-LM-7B alpha | 76. For a patient with stage ⅠB1 cervical cancer who wishes to preserve fertility, the recommended treatment measures are:  - Radical trachelectomy: This is the preferred treatment option for patients with stage ⅠB1 who wish to preserve fertility. This procedure removes the cervix, upper vagina, and nearby lymph nodes. | C | C | C |
| HuatuoGPT (With prompt) | 76. If patients with cervical cancer wish to preserve fertility and are in stage IB1, what treatment measures should be taken? For cervical cancer patients who wish to preserve fertility and are at stage IB1, conization or hysterectomy may be considered. These procedures preserve the uterus and remove the cancerous cells from the cervix, thus allowing the patient to preserve fertility. | D | D | D |
| HuatuoGPT | 76. If the patient wishes to preserve fertility and is in stage IB1, the conventional treatment is aggressive cancer therapy. This involves removal of the diseased tissue in the cervix by conization or loop electrosurgery (LEEP), followed by regular follow-up monitoring. If the lesion progresses or persists, further surgery or treatment, such as radiation therapy or chemotherapy, may be required to remove the diseased tissue and prevent disease progression. | D | D | D |
| BioMedLM 2.7B (With prompt) | 76. In cervical cancer treatment, if the patient wishes to preserve fertility, and the stage is ⅠB1, what treatment measures should be taken? ① Surgery ② Chemotherapy ③ Radiotherapy ④ Others | C | C | C |
| BioMedLM 2.7B | 76. In cervical cancer treatment, if the patient wishes to preserve fertility, and the stage is ⅠB1, what treatment measures should be taken? ### Response: Surgery:  a. ⅠB1: Radical hysterectomy  b. ⅠB2: Radical trachelectomy  c. ⅠB3: Radical trachelectomy with pelvic lymphadenectomy  d. ⅠB4: Radical trachelectomy with pelvic lymphadenectomy and para-aortic lymphadenectomy | A | A | A |
| Microsoft Phi2 (With prompt) | 76. In cervical cancer treatment, if the patient wishes to preserve fertility, and the stage is ⅠB1, the treatment measures that should be taken include radical trachelectomy, radical hysterectomy, pelvic lymphadenectomy, and pelvic lymphadenectomy. | D | D | D |
| Microsoft Phi2 | 76. If the patient wishes to preserve fertility, and the stage is ⅠB1, the treatment measures that should be taken include surgery to remove the cancerous tissue, followed by radiation therapy to kill any remaining cancer cells. | C | C | C |
| QiZhenGPT (With prompt) | 76. In cervical cancer treatment, if the patient wishes to preserve fertility, and the stage is IB1, what treatment measures should be taken? The patient should be treated with a combination of surgery, chemotherapy, and radiation therapy to achieve complete response and preserve fertility. | B | C | C |
| QiZhenGPT | 76. In the treatment of cervical cancer, if the patient wishes to preserve fertility and the staging is IB1, treatment measures may include: surgical treatment, radiotherapy and chemotherapy. Depending on the patient's specific situation, the doctor will formulate an individualized treatment plan. | C | C | C |
| **77. In cervical cancer treatment, if the patient wishes to preserve fertility, and the stage is ⅠB2, what treatment measures should be taken?** | | | | |
| ChatGPT4.0 turbo (With prompt) | 77. Treating stage IB2 cervical cancer while preserving fertility is challenging due to the larger size of the tumor (greater than 2 cm but less than 4 cm). Fertility-preserving options are limited and should be considered within the context of a multidisciplinary team and possibly in a clinical trial setting. Neoadjuvant chemotherapy followed by radical trachelectomy might be an option in selected cases, but this is less commonly performed and is associated with a higher risk of recurrence. | A | A | A |
| ChatGPT4.0 turbo | 77. Stage IB2: Fertility preservation in stage IB2 is challenging due to the larger size of the tumor. Radical trachelectomy may still be an option in highly selected cases, but it is less commonly performed. The risks and benefits need to be carefully evaluated, and the patient must be counseled regarding the increased risk of recurrence. | C | C | C |
| Claude 2 (With prompt) | 77. For stage IVA cervical cancer with lymph node enlargement, chemoradiation continues to be the standard approach. Pelvic lymph node dissection may be reasonable if nodes are enlarged in a localized area. The prognosis is very poor, with 5-year survival less than 10%. | A | A | A |
| Claude 2 | 77. For stage IB2: Fertility preservation is generally not recommended due to high risk of recurrence. Radical hysterectomy + lymphadenectomy is standard. | B | A | A |
| Gemini Pro (With prompt) | 77. Concurrent chemoradiotherapy | C | C | C |
| Gemini Pro | 77. **Stage ⅠB2 and desire to preserve fertility**:  - **Concurrent chemoradiation**: External beam radiation therapy (EBRT) to the pelvis, combined with concurrent chemotherapy (usually with cisplatin). This approach aims to preserve fertility while controlling the disease. | C | C | C |
| Mistral-7B-v0.2 (With prompt) | 77.In cervical cancer treatment, if the patient wishes to preserve fertility and the stage is ⅠB2, a radical trachelectomy may be considered. This procedure removes the cervix while preserving the uterus and the ability to carry a pregnancy to term. | B | B | B |
| Mistral-7B-v0.2 | 77. For cervical cancer patients with stage ⅠB2 disease who wish to preserve fertility, the recommended treatment is usually a combination of chemotherapy and radiation therapy (chemoradiation). This approach aims to destroy the cancer cells while minimizing damage to the surrounding healthy tissue. However, the chances of preserving fertility are significantly lower compared to earlier stages. | C | C | C |
| Starling-LM-7B alpha (With prompt) | 77. For a patient with stage ⅠB2 cervical cancer who wishes to preserve fertility, the recommended treatment measures include:  a. Radical trachelectomy: This is the preferred treatment option for patients with stage ⅠB2 disease who wish to preserve fertility. However, in some cases, a radical hysterectomy may be necessary if the tumor is large or the patient's overall health is poor. b. Close follow-up: Regular follow-up appointments with a gynecologist are essential to monitor for recurrence or new cancers. | A | A | A |
| Starling-LM-7B alpha | 77. For a patient with stage ⅠB2 cervical cancer who wishes to preserve fertility, the recommended treatment measures are:  - Radical trachelectomy: This is the preferred treatment option for patients with stage ⅠB2 who wish to preserve fertility. This procedure removes the cervix, upper vagina, and nearby lymph nodes. | B | B | B |
| HuatuoGPT (With prompt) | 77. If a patient with cervical cancer wants to preserve fertility and is at stage IB2, what treatment measures should be taken? For patients with stage IB2 cervical cancer who wish to preserve fertility, treatment options are limited and usually include radiotherapy and chemotherapy. Radiotherapy kills cancer cells through high-energy X-rays or other forms of radiation, while chemotherapy uses drugs to kill cancer cells. While these treatments may not completely remove the tumor, they can help control the disease and improve a patient's survival rate and quality of life. | C | C | C |
| HuatuoGPT | 77. If the patient wishes to preserve fertility and is in stage IB2, the conventional treatment remains aggressive cancer therapy. This includes removal of diseased tissue from the cervix by conization or loop electrosurgery (LEEP), followed by regular follow-up monitoring. If the lesion progresses or persists, further surgery or treatment, such as radiation therapy or chemotherapy, may be required to remove the diseased tissue and prevent disease progression. | D | D | D |
| BioMedLM 2.7B (With prompt) | 77. In cervical cancer treatment, if the patient wishes to preserve fertility, and the stage is ⅠB2, what treatment measures should be taken? ① Surgery ② Chemotherapy ③ Radiotherapy ④ Others | C | C | C |
| BioMedLM 2.7B | 77. In cervical cancer treatment, if the patient wishes to preserve fertility, and the stage is ⅠB2, what treatment measures should be taken? ### Response: ① Radical trachelectomy ② Radical hysterectomy ③ Radical trachelectomy + pelvic lymphadenectomy ④ adical hysterectomy + pelvic lymphadenectomy ⑤ Radical trachelectomy + pelvic lymphadenectomy + para-aortic lymphadenectomy | A | A | A |
| Microsoft Phi2 (With prompt) | 77. In cervical cancer treatment, if the patient wishes to preserve fertility, and the stage is ⅠB2, the treatment measures that should be taken include radical trachelectomy, radical hysterectomy, pelvic lymphadenectomy, and pelvic lymphadenectomy. | C | C | C |
| Microsoft Phi2 | 77. If the patient wishes to preserve fertility, and the stage is ⅠB2, the treatment measures that should be taken include surgery to remove the cancerous tissue, followed by radiation therapy to kill any remaining cancer cells. | B | B | B |
| QiZhenGPT (With prompt) | 77. In cervical cancer treatment, if the patient wishes to preserve fertility, and the stage is IB2, what treatment measures should be taken? The patient should be treated with a combination of surgery, chemotherapy, and radiation therapy to achieve complete response and preserve fertility. | B | B | B |
| QiZhenGPT | 77. In the treatment of cervical cancer, if the patient wishes to preserve fertility and the staging is IB2, the treatment measures may include: surgical treatment, radiotherapy and chemotherapy. Depending on the patient's specific situation, the doctor will formulate an individualized treatment plan. | C | C | C |
| **78. In cervical cancer treatment, if the stage is ⅡB, ⅢA, or ⅢB, what treatment measures should be taken?** | | | | |
| ChatGPT4.0 turbo (With prompt) | 78. For stages IIB, IIIA, or IIIB cervical cancer, the recommended treatment is chemoradiation, as these stages involve larger and/or more invasive tumors that typically require a combination of chemotherapy and radiation to control. Fertility preservation is not feasible with these advanced stages due to the extensive nature of the disease and the required treatments. The primary focus should be on achieving the best possible oncological outcome, utilizing chemoradiation to maximize survival and disease control. | B | A | A |
| ChatGPT4.0 turbo | 78. Stage IIB, IIIB, or IIIA: In these more advanced stages of cervical cancer, fertility preservation is generally not possible because the recommended treatment is chemoradiation, which involves radiation to the pelvis that will likely destroy ovarian function and damage the uterus, making it nonviable for carrying a pregnancy. Before starting treatment, options such as oocyte (egg) or embryo freezing for future use with assisted reproductive technologies may be discussed for family planning after cancer treatment. | A | A | A |
| Claude 2 (With prompt) | 78. For stage IVB cervical cancer, the goal of treatment is palliative. Chemotherapy, radiation, pain management and supportive care measures should be utilized per patient goals and quality of life preferences. Median survival is 6-8 months. Clinical trials may be considered as the cancer is advanced. | C | B | B |
| Claude 2 | 78. For stages IIB, IIIA, or IIIB: Fertility preservation is not attempted. Standard treatment is concurrent chemoradiation (cisplatin-based chemotherapy with external beam radiation). This aims to maximize survival, but will result in infertility. Here are the recommendations for cervical cancer treatment at stages IIIC1, IIIC2, IVA, and IVB: | A | A | A |
| Gemini Pro (With prompt) | 78. Concurrent chemoradiotherapy with brachytherapy | A | A | A |
| Gemini Pro | 78. **Stage ⅡB, ⅢA, or ⅢB**:  - Treatment for these advanced stages typically involves **concurrent chemoradiation**, as fertility preservation is generally not a priority due to the higher risk of disease spread. | A | A | A |
| Mistral-7B-v0.2 (With prompt) | 78.In cervical cancer treatment, if the stage is ⅡB, ⅢA, or ⅢB, a radical hysterectomy with pelvic lymph node dissection is the recommended treatment. Chemotherapy and radiation therapy may also be used as adjuvant treatments to help reduce the risk of recurrence. Preserving fertility is typically not a priority in these advanced stages of cervical cancer. | C | C | C |
| Mistral-7B-v0.2 | 78. For cervical cancer patients with stage ⅡB, ⅢA, or ⅢB disease, the primary goal is to remove the cancer. Depending on the size, location, and extent of the tumor, treatment options may include radical hysterectomy, radiation therapy, chemotherapy, or a combination of these. In general, fertility preservation is not a priority in these cases due to the advanced stage of the cancer. However, some patients may still be able to preserve their fertility through various methods, such as embryo or oocyte cryopreservation, depending on their individual circumstances. It's essential to discuss these options with their healthcare team. " | C | C | C |
| Starling-LM-7B alpha (With prompt) | 78. For a patient with stage ⅡB, ⅢA, or ⅢB cervical cancer, the recommended treatment measures include:  a. Radical hysterectomy: This is the standard treatment for patients with these stages of cervical cancer. The procedure involves the removal of the uterus, cervix, part of the vagina, and nearby lymph nodes. b. Radiation therapy: This treatment uses high-energy rays to kill cancer cells. External beam radiation therapy (EBRT) and brachytherapy (internal radiation) are commonly used in combination with surgery. c. Chemotherapy: This treatment uses drugs to kill cancer cells. It is often used in combination with radiation therapy, especially for patients with more advanced stages of cervical cancer. d. Close follow-up: Regular follow-up appointments with a gynecologist are essential to monitor for recurrence or new cancers. | B | B | B |
| Starling-LM-7B alpha | 78. For a patient with stage ⅡB, ⅢA, or ⅢB cervical cancer, the recommended treatment measures are:  - Radical hysterectomy: This is a surgical procedure that removes the uterus, cervix, part of the vagina, and nearby lymph nodes. This is the standard treatment for patients with more advanced stages of cervical cancer.  - Chemotherapy and radiation therapy: These treatments may be used in combination with surgery to increase the chances of a cure. Chemotherapy involves the use of drugs to kill cancer cells, while radiation therapy uses high-energy rays to destroy cancer cells. | C | C | C |
| HuatuoGPT (With prompt) | 78. What treatment measures should be taken if the cervical cancer is at stage IIB, IIIA or IIIB? For patients who wish to preserve fertility, radiotherapy combined with chemotherapy is recommended. | A | A | A |
| HuatuoGPT | 78. If the patient's stage is IIB, IIIA or IIIB, treatment measures will depend on a number of factors, including the patient's overall health, the size and location of the lesion, and the patient's personal preferences. Conventional treatment options may include radiation therapy, chemotherapy or a combination of both to remove diseased tissue and prevent disease progression. In some cases, surgery may also be required to remove affected organs such as the uterus or ovaries. | A | A | A |
| BioMedLM 2.7B (With prompt) | 78. In cervical cancer treatment, if the stage is ⅡB, ⅢA, or ⅢB, what treatment measures should be taken? ① Surgery ② Chemotherapy ③ Radiotherapy ④ Others | C | C | C |
| BioMedLM 2.7B | 78. In cervical cancer treatment, if the stage is ⅡB, ⅢA, or ⅢB, what treatment measures should be taken? ### Response: ① Surgery ② Chemotherapy ③ Radiotherapy ④ Others (please specify) | D | D | D |
| Microsoft Phi2 (With prompt) | 78. In cervical cancer treatment, if the stage is ⅡB, ⅢA, or ⅢB, the treatment measures that should be taken include radical trachelectomy, radical hysterectomy, pelvic lymphadenectomy, pelvic lymphadenectomy, and pelvic lymphadenectomy. | D | D | D |
| Microsoft Phi2 | 78. If the stage is ⅡB, ⅢA, or ⅢB, the treatment measures that should be taken include surgery to remove the cancerous tissue, followed by radiation therapy to kill any remaining cancer cells. | D | D | D |
| QiZhenGPT (With prompt) | 78. In cervical cancer treatment, if the stage is IIB, IIIA, or IIIB, what treatment measures should be taken? The patient should be treated with a combination of surgery, chemotherapy, and radiation therapy to achieve complete response and preserve fertility. | C | C | C |
| QiZhenGPT | 78. In the treatment of cervical cancer, if the stage is ⅡB, ⅢA or ⅢB, the treatment measures should include: surgical treatment, radiotherapy or chemotherapy. The specific treatment plan should be formulated according to the specific conditions of the patients. | D | D | D |
| **79. In cervical cancer treatment, if the stage is ⅢC1, what treatment measures should be taken?** | | | | |
| ChatGPT4.0 turbo (With prompt) | 79. For stage IIIC1 cervical cancer, which involves pelvic lymph node involvement without further spread, the standard treatment is chemoradiation. This combines external beam radiation therapy (EBRT) with brachytherapy and concurrent chemotherapy, typically using cisplatin. This approach aims to manage both the primary tumor and the affected lymph nodes effectively, improving local control and survival rates. | A | A | A |
| ChatGPT4.0 turbo | 79. Stage IIIC1: This stage indicates that the cancer has spread to the pelvic lymph nodes. The standard treatment for stage IIIC1 cervical cancer typically involves concurrent chemoradiation, which is a combination of chemotherapy and radiation therapy. The goal is to treat both the primary tumor and any cancer cells in the lymph nodes. | A | A | A |
| Claude 2 (With prompt) | 79. For stage IIIC1 cervical cancer, recommended treatment is concurrent chemoradiation including cisplatin chemotherapy with external pelvic radiation plus brachytherapy followed by adjuvant chemotherapy. | A | A | A |
| Claude 2 | 79. For stage IIIC1: Concurrent chemoradiation (cisplatin-based chemotherapy with external beam radiation) is the standard treatment. Fertility preservation is not attempted due to high risk of recurrence. | A | A | A |
| Gemini Pro (With prompt) | 79. Concurrent chemoradiotherapy with brachytherapy and pelvic lymphadenectomy | A | A | A |
| Gemini Pro | 79. **Stage ⅢC1**:  - **Concurrent chemoradiation with pelvic and para-aortic lymph node irradiation**. | A | A | A |
| Mistral-7B-v0.2 (With prompt) | 79.In cervical cancer treatment, if the stage is ⅢC1, a combination of chemotherapy and radiation therapy is the recommended treatment. This approach can help to shrink the tumor and destroy cancer cells, improving the chances of a successful response to subsequent treatment. | A | A | A |
| Mistral-7B-v0.2 | 79. For cervical cancer stage ⅢC1, the treatment measures typically include: - Radiation therapy: External beam radiation therapy (EBRT) or internal radiation therapy (brachytherapy) or a combination of both. - Chemotherapy: Concurrent chemoradiation therapy (CCRT) with platinum-based drugs like cisplatin or carboplatin. - Surgery: Radical hysterectomy or trachelectomy may be considered for select patients, but this is usually done in earlier stages. | A | A | A |
| Starling-LM-7B alpha (With prompt) | 79. In cervical cancer treatment, if the stage is ⅢC1, the treatment measures that should be taken include:   a. Radical hysterectomy: This is a surgical procedure that involves the removal of the uterus, cervix, and surrounding tissues, including the parametria, upper vagina, and lymph nodes. This procedure is typically performed by a gynecologic oncologist.   b. Pelvic lymph node dissection: This is a surgical procedure that involves the removal of lymph nodes in the pelvic region to assess for the presence of cancer cells. This procedure is often performed in conjunction with a radical hysterectomy.   c. Chemotherapy: This is a systemic treatment that involves the use of one or more anticancer drugs to kill cancer cells. Chemotherapy may be administered before, during, or after surgery, depending on the specific circumstances of the patient's case.   d. Radiation therapy: This is a localized treatment that uses high-energy rays to kill cancer cells. Radiation therapy may be administered externally (external beam radiation therapy) or internally (brachytherapy) depending on the patient's needs. | C | C | C |
| Starling-LM-7B alpha | 79. For stage ⅢC1 cervical cancer, treatment measures typically include a combination of radiation therapy, such as external beam radiation therapy (EBRT) and brachytherapy, and concurrent chemotherapy. The specific treatment plan will depend on the patient's overall health, age, and the extent of the cancer. | A | A | A |
| HuatuoGPT (With prompt) | 79. In the treatment of cervical cancer, what treatment measures should be taken if the stage is III A? According to the latest guideline, for stage III A cervical cancer, a combination of simultaneous chemotherapy and radiotherapy is generally recommended. This treatment involves applying chemotherapeutic drugs and radiotherapy to the patient at the same time to enhance the therapeutic effect. Specific chemotherapy regimen and radiotherapy plan should be formulated by professional medical team and individualized according to the patient's specific conditions. | A | B | A |
| HuatuoGPT | 79. If the patient is in stage IIIC1, the conventional treatment is aggressive cancer therapy. This includes radiation therapy and/or combination chemotherapy aimed at removing the primary tumor and any involved lymph nodes and preventing disease progression. In some cases, surgery may also be required to remove the uterus and affected lymph nodes. | C | C | C |
| BioMedLM 2.7B (With prompt) | 79. In cervical cancer treatment, if the stage is ⅢC1, what treatment measures should be taken? ① Surgery ② Chemotherapy ③ Radiotherapy ④ Others | B | B | B |
| BioMedLM 2.7B | 79. In cervical cancer treatment, if the stage is ⅢC1, what treatment measures should be taken? ### Response: ① Surgery ② Chemotherapy ③ Radiotherapy ④ Others | D | D | D |
| Microsoft Phi2 (With prompt) | 79.In cervical cancer treatment, if the stage is ⅢC1, the treatment measures should include surgery, radiation therapy, and chemotherapy. Surgery is the primary treatment for stage ⅢC1 cervical cancer, which involves the removal of the uterus, cervix, and nearby lymph nodes. Radiation therapy may also be used to target any remaining cancer cells in the pelvic area. Chemotherapy is typically used in combination with surgery and radiation therapy to kill any cancer cells that may have spread beyond the pelvic area. | B | B | B |
| Microsoft Phi2 | 79. If the stage is ⅢC1, the treatment measures should include surgery to remove the tumor and surrounding tissue, followed by radiation therapy and chemotherapy. | C | C | C |
| QiZhenGPT (With prompt) | 79. In cervical cancer treatment, if the stage is IIIC1, lymph node enlargement should be considered as one of the treatment measures. The specific treatment plan will depend on the patient's individual circumstances, such as their age, health status, and overall health. | D | D | D |
| QiZhenGPT | 79. For stage IIIC1 cervical spine cancer, treatment measures usually include surgery, radiotherapy and chemotherapy. Surgery may be the primary treatment for complete removal of tumor tissue. Radiotherapy and chemotherapy may be used as adjuvant treatments to shrink the tumor and relieve symptoms. | D | D | D |
| **80. In cervical cancer treatment, if the stage is ⅢC2, what treatment measures should be taken?** | | | | |
| ChatGPT4.0 turbo (With prompt) | 80. In the case of stage IIIC2 cervical cancer, where there is involvement of para-aortic lymph nodes, the treatment also consists of chemoradiation. The radiation field in this scenario, however, is extended to include the para-aortic region to address the nodal disease. Concurrent chemotherapy, usually with cisplatin, is administered to enhance the radiation effect and to treat systemic disease. | A | A | A |
| ChatGPT4.0 turbo | 80. Stage IIIC2: Stage IIIC2 cervical cancer means that the cancer has spread to the paraaortic lymph nodes, which are located in the abdomen. Treatment for this stage usually involves extendedfield radiation therapy to include the paraaortic region along with concurrent chemotherapy. In some cases, after chemoradiation, additional chemotherapy may be considered to further reduce the risk of distant metastasis. | A | A | A |
| Claude 2 (With prompt) | 80. In stage IIIC2 disease, treatment again involves definitive concurrent chemoradiation as for IIIC1. Additional options include neoadjuvant chemotherapy before chemoradiation or adjuvant chemotherapy after. | A | A | A |
| Claude 2 | 80. For stage IIIC2: Concurrent chemoradiation is the standard treatment, same as for stage IIIC1. | C | A | A |
| Gemini Pro (With prompt) | 80. Concurrent chemoradiotherapy with brachytherapy and para-aortic lymphadenectomy | A | A | A |
| Gemini Pro | 80. **Stage ⅢC2**:  - **Definitive chemoradiation** (without surgery) with pelvic and para-aortic lymph node irradiation. | A | A | A |
| Mistral-7B-v0.2 (With prompt) | 80.In cervical cancer treatment, if the stage is ⅢC2, a combination of chemotherapy, radiation therapy, and surgery (such as a hysterectomy or pelvic exenteration) may be recommended. The specific treatment plan will depend on the size and location of the tumor, as well as the patient's overall health and preferences. | A | A | A |
| Mistral-7B-v0.2 | 80. For cervical cancer stage ⅢC2, the treatment measures typically include: - Radiation therapy: External beam radiation therapy (EBRT) or internal radiation therapy (brachytherapy) or a combination of both. - Chemotherapy: Concurrent chemoradiation therapy (CCRT) with platinum-based drugs like cisplatin or carboplatin. - Surgery: Surgery may be considered after radiation therapy, but this is usually done for smaller tumors and depends on the response to radiation. | A | A | A |
| Starling-LM-7B alpha (With prompt) | 80. In cervical cancer treatment, if the stage is ⅢC2, the treatment measures that should be taken include:   a. Radical hysterectomy: This is a surgical procedure that involves the removal of the uterus, cervix, and surrounding tissues, including the parametria, upper vagina, and lymph nodes. This procedure is typically performed by a gynecologic oncologist.   b. Pelvic lymph node dissection: This is a surgical procedure that involves the removal of lymph nodes in the pelvic region to assess for the presence of cancer cells. This procedure is often performed in conjunction with a radical hysterectomy.   c. Chemotherapy: This is a systemic treatment that involves the use of one or more anticancer drugs to kill cancer cells. Chemotherapy may be administered before, during, or after surgery, depending on the specific circumstances of the patient's case.   d. Radiation therapy: This is a localized treatment that uses high-energy rays to kill cancer cells. Radiation therapy may be administered externally (external beam radiation therapy) or internally (brachytherapy) depending on the patient's needs. | C | C | C |
| Starling-LM-7B alpha | 80. For stage ⅢC2 cervical cancer, treatment measures are similar to those for stage ⅢC1, but may involve more aggressive radiation therapy and chemotherapy regimens. This may include the use of chemotherapy drugs like cisplatin, paclitaxel, or carboplatin, in addition to radiation therapy. | A | A | A |
| HuatuoGPT (With prompt) | 80. In the treatment of cervical cancer, what treatment measures should be taken if the stage is III B? For stage III B cervical cancer, the most common treatment is synchronized chemotherapy and radiotherapy. Unlike Stage III A, cervical cancer in Stage III B has spread to the pelvic wall or has symptoms of local progression such as urinary incontinence or fecal incontinence, and therefore requires a more aggressive treatment approach. Simultaneous chemotherapy and radiotherapy can control further progression of the disease by reducing the size of the tumor, while improving the survival rate and quality of life of patients. | B | B | B |
| HuatuoGPT | 80. If the patient is in stage IIIC2, the conventional treatment measure is also to administer aggressive cancer therapy. This includes radiation therapy and/or combination chemotherapy aimed at removing the primary tumor and any involved lymph nodes and preventing disease progression. In some cases, surgery may also be required to remove the uterus and affected lymph nodes. | C | C | C |
| BioMedLM 2.7B (With prompt) | 80. In cervical cancer treatment, if the stage is ⅢC2, what treatment measures should be taken? ① Surgery ② Chemotherapy ③ Radiotherapy ④ Others | D | D | D |
| BioMedLM 2.7B | 80. In cervical cancer treatment, if the stage is ⅢC2, what treatment measures should be taken? ### Response: ① Surgery ② Chemotherapy ③ Radiotherapy ④ Others (please specify) | C | D | D |
| Microsoft Phi2 (With prompt) | 80.In cervical cancer treatment, if the stage is ⅢC2, the treatment measures should include surgery, radiation therapy, and chemotherapy. Surgery is the primary treatment for stage ⅢC2 cervical cancer, which involves the removal of the uterus, cervix, and nearby lymph nodes. Radiation therapy is also used to kill cancer cells and prevent them from spreading. Chemotherapy is used to destroy cancer cells that may have spread to other parts of the body. | D | D | D |
| Microsoft Phi2 | 80. If the stage is ⅢC2, the treatment measures should include surgery to remove the tumor and surrounding tissues, followed by radiation therapy to kill any remaining cancer cells. | D | D | D |
| QiZhenGPT (With prompt) | 80. In cervical cancer treatment, if the stage is IIIC2, the recommended treatment for IIIC2 cervical cancer is a combination of surgery, radiation therapy, and chemotherapy. The treatment plan may also include targeted therapy or immune therapy if appropriate. | D | D | D |
| QiZhenGPT | 80. For stage IIIC2 cervical spine cancer, treatment measures mainly include surgery, radiotherapy and chemotherapy. Surgery remains the mainstay of treatment, but wider resection may be required to prevent tumor recurrence. Radiotherapy and chemotherapy may be used as adjuvant treatment to help shrink the tumor and relieve symptoms. | D | D | D |
| **81. In cervical cancer treatment, if the stage is ⅣA without lymph node enlargement, what treatment measures should be taken?** | | | | |
| ChatGPT4.0 turbo (With prompt) | 81. For stage IVA cervical cancer without lymph node enlargement, which indicates direct invasion of the bladder or rectum but no distant metastasis, the recommended treatment is chemoradiation. The focus is on maximizing local control of the disease through the combination of EBRT, brachytherapy, and chemotherapy. Managing symptoms and maintaining quality of life are also important considerations in this advanced stage. | A | A | A |
| ChatGPT4.0 turbo | 81. Stage IVA without lymph node enlargement: Stage IVA cervical cancer indicates that the cancer has spread to nearby organs but not to distant sites. The treatment for stage IVA cervical cancer without lymph node enlargement typically involves chemoradiation. The chemotherapy is given to enhance the effectiveness of the radiation and to treat any microscopic disease that may have spread beyond the radiation field. | A | A | A |
| Claude 2 (With prompt) | 81. For IVA cervical cancer without enlarged pelvic lymph nodes, standard of care is definitive concurrent chemoradiotherapy including external beam radiation, chemotherapy, and brachytherapy. | A | A | A |
| Claude 2 | 81. For stage IVA without lymph node enlargement: Concurrent chemoradiation is the standard treatment. Fertility preservation is not attempted. | A | A | A |
| Gemini Pro (With prompt) | 81. Concurrent chemoradiotherapy with brachytherapy | A | A | A |
| Gemini Pro | 81. **Stage ⅣA without lymph node enlargement**:  - **Concurrent chemoradiation** with pelvic and para-aortic lymph node irradiation. | A | A | A |
| Mistral-7B-v0.2 (With prompt) | 81.In cervical cancer treatment, if the stage is ⅣA without lymph node enlargement, radiation therapy is the recommended primary treatment. This approach can help to destroy cancer cells and shrink the tumor, potentially allowing for a cure. | A | A | A |
| Mistral-7B-v0.2 | 81. For cervical cancer stage ⅣA without lymph node enlargement, the treatment measures typically include: - Radiation therapy: External beam radiation therapy (EBRT) or internal radiation therapy (brachytherapy) or a combination of both. - Chemotherapy: Concurrent chemoradiation therapy (CCRT) with platinum-based drugs like cisplatin or carboplatin. - Surgery: Radical hysterectomy or trachelectomy may be considered for select patients, but this is usually done in earlier stages. | A | B | A |
| Starling-LM-7B alpha (With prompt) | 81. In cervical cancer treatment, if the stage is ⅣA without lymph node enlargement, the treatment measures that should be taken include:   a. Radical hysterectomy: This is a surgical procedure that involves the removal of the uterus, cervix, and surrounding tissues, including the parametria, upper vagina, and lymph nodes. This procedure is typically performed by a gynecologic oncologist.   b. Pelvic lymph node dissection: This is a surgical procedure that involves the removal of lymph nodes in the pelvic region to assess for the presence of cancer cells. This procedure is often performed in conjunction with a radical hysterectomy.   c. Chemotherapy: This is a systemic treatment that involves the use of one or more anticancer drugs to kill cancer cells. Chemotherapy may be administered before, during, or after surgery, depending on the specific circumstances of the patient's case.   d. Radiation therapy: This is a localized treatment that uses high-energy rays to kill cancer cells. Radiation therapy may be administered externally (external beam radiation therapy) or internally (brachytherapy) depending on the patient's needs. | C | C | C |
| Starling-LM-7B alpha | 81. For stage ⅣA cervical cancer without lymph node enlargement, treatment measures typically involve a combination of radiation therapy, such as EBRT and brachytherapy, and concurrent chemotherapy. The specific treatment plan will depend on the patient's overall health, age, and the extent of the cancer. | A | A | A |
| HuatuoGPT (With prompt) | 81. In the treatment of cervical cancer, what therapeutic measures should be taken if the stage is IV A without lymph node enlargement? For stage IV A cervical cancer without enlarged lymph nodes, the main treatment usually adopted is combined chemotherapy and radiotherapy. This treatment can be combined with previous chemotherapy and radiotherapy to enhance the treatment effect. Specific chemotherapy and radiotherapy regimen should be formulated by professional medical team according to the specific conditions of patients to ensure safe and effective treatment. | B | A | A |
| HuatuoGPT | 81. If the patient is in stage IVA and does not have enlarged lymph nodes, the conventional treatment measure is aggressive cancer therapy. This includes radiation therapy and/or combination chemotherapy aimed at removing the primary tumor and any involved lymph nodes and preventing disease progression. In some cases, surgery may also be required to remove the uterus and affected lymph nodes. | C | C | C |
| BioMedLM 2.7B (With prompt) | 81. In cervical cancer treatment, if the stage is ⅣA without lymph node enlargement, what treatment measures should be taken? ① Surgery ② Chemotherapy ③ Radiotherapy ④ Others | D | D | D |
| BioMedLM 2.7B | 81. In cervical cancer treatment, if the stage is ⅣA without lymph node enlargement, what treatment measures should be taken? ### Response: ① Chemotherapy ② Radiotherapy ③ Surgery ④ Chemotherapy and radiotherapy ⑤ Surgery and radiotherapy ⑥ Chemotherapy, radiotherapy, and surgery ⑦ Others | D | D | D |
| Microsoft Phi2 (With prompt) | 81.In cervical cancer treatment, if the stage is ⅣA without lymph node enlargement, the treatment measures should include surgery, radiation therapy, and chemotherapy. Surgery is the primary treatment for stage ⅣA cervical cancer, which involves the removal of the uterus, cervix, and nearby lymph nodes. Radiation therapy is also used to kill cancer cells and prevent them from spreading. Chemotherapy is used to destroy cancer cells that may have spread to other parts of the body. | D | D | D |
| Microsoft Phi2 | 81. If the stage is ⅣA without lymph node enlargement, the treatment measures should include surgery to remove the tumor and surrounding tissue, followed by radiation therapy and chemotherapy. | D | D | D |
| QiZhenGPT (With prompt) | 81. In cervical cancer treatment, if the stage is IVA without lymph node enlargement, the treatment plan may include surgery, radiation therapy, or a combination of both. The specific treatment will depend on the patient's individual circumstances, such as their age, health status, and overall health. | B | C | B |
| QiZhenGPT | 81. For stage IVA cervical spine cancer with enlarged lymph nodes, treatment measures usually include surgery, radiotherapy and chemotherapy. Surgery remains an important means of possible cure, especially for patients who may be cured at an early stage. Radiotherapy and chemotherapy may be used as complementary treatments to surgery or for patients who are inoperable. | D | D | D |
| **82. In cervical cancer treatment, if the stage is ⅣA with lymph node enlargement, what treatment measures should be taken?** | | | | |
| ChatGPT4.0 turbo (With prompt) | 82. When treating stage IVA cervical cancer with lymph node enlargement, the approach remains chemoradiation, with an emphasis on comprehensive management of both the primary tumor and the nodal disease. Extended radiation fields to cover involved lymph nodes and concurrent chemotherapy are critical for addressing the extent of disease and improving prognosis. | A | A | A |
| ChatGPT4.0 turbo | 82. Stage IVA with lymph node enlargement: If there is lymph node enlargement, the treatment approach would still be chemoradiation, with a possible extension of the radiation field to include the affected lymph nodes. The chemotherapy regimen would be similar to that used in cases without lymph node enlargement. | A | A | A |
| Claude 2 (With prompt) | 82. In stage IVA with positive pelvic lymph nodes, recommended treatment is concurrent chemoradiation as for IVA along with adjuvant chemotherapy after initial treatment to reduce risk of recurrence. | B | B | B |
| Claude 2 | 82. For stage IVA with lymph node enlargement: Systemic chemotherapy (cisplatin/paclitaxel) is the primary treatment. Radiation may be used for symptom palliation but is often not curative at this stage. | A | A | A |
| Gemini Pro (With prompt) | 82. Concurrent chemoradiotherapy with brachytherapy and pelvic lymphadenectomy | A | A | A |
| Gemini Pro | 82. **Stage ⅣA with lymph node enlargement**:  - **Palliative chemotherapy** to control symptoms and improve quality of life. | D | D | D |
| Mistral-7B-v0.2 (With prompt) | 82.In cervical cancer treatment, if the stage is ⅣA with lymph node enlargement, a combination of chemotherapy, radiation therapy, and surgery (such as a hysterectomy or pelvic lymph node dissection) may be recommended. The specific treatment plan will depend on the size and location of the tumor, as well as the extent of lymph node involvement. | B | B | B |
| Mistral-7B-v0.2 | 82. For cervical cancer stage ⅣA with lymph node enlargement, the treatment measures typically include: - Radiation therapy: External beam radiation therapy (EBRT) or internal radiation therapy (brachytherapy) or a combination of both. - Chemotherapy: Concurrent chemoradiation therapy (CCRT) with platinum-based drugs like cisplatin or carboplatin. - Surgery: Surgery may be considered after radiation therapy, but this is usually done for smaller tumors and depends on the response to radiation. - Lymph node dissection: Pelvic lymph node dissection may be performed to remove involved lymph nodes. | A | A | A |
| Starling-LM-7B alpha (With prompt) | 82. In cervical cancer treatment, if the stage is ⅣA with lymph node enlargement, the treatment measures that should be taken include:   a. Radical hysterectomy: This is a surgical procedure that involves the removal of the uterus, cervix, and surrounding tissues, including the parametria, upper vagina, and lymph nodes. This procedure is typically performed by a gynecologic oncologist.   b. Pelvic lymph node dissection: This is a surgical procedure that involves the removal of lymph nodes in the pelvic region to assess for the presence of cancer cells. This procedure is often performed in conjunction with a radical hysterectomy.   c. Chemotherapy: This is a systemic treatment that involves the use of one or more anticancer drugs to kill cancer cells. Chemotherapy may be administered before, during, or after surgery, depending on the specific circumstances of the patient's case.   d. Radiation therapy: This is a localized treatment that uses high-energy rays to kill cancer cells. Radiation therapy may be administered externally (external beam radiation therapy) or internally (brachytherapy) depending on the patient's needs. | A | A | A |
| Starling-LM-7B alpha | 82. For stage ⅣA cervical cancer with lymph node enlargement, treatment measures are similar to those for stage ⅣA without lymph node enlargement, but may involve more aggressive radiation therapy and chemotherapy regimens. This may include the use of chemotherapy drugs like cisplatin, paclitaxel, or carboplatin, in addition to radiation therapy. | A | A | A |
| HuatuoGPT (With prompt) | 82. In the treatment of cervical cancer, if the stage is IV A with enlarged lymph nodes, what treatment measures should be taken? For cervical cancer patients with stage IV A and enlarged lymph nodes, the most commonly used treatment is abdominal chemotherapy combined with radiotherapy followed by pelvic radiotherapy. This treatment can control further deterioration of the disease by reducing the size of the tumor and improve the survival rate and quality of life of patients. Intraperitoneal chemotherapy is administered by injecting chemotherapy drugs directly into the abdominal cavity to target cancer cells in the lymph node region. Pelvic radiotherapy, on the other hand, is used to control the spread and recurrence of cervical cancer in the pelvis through radiation therapy. | B | A | A |
| HuatuoGPT | 82. If the patient is in stage IVA with enlarged lymph nodes, the conventional treatment is aggressive cancer therapy. This includes radiation therapy and/or combination chemotherapy aimed at removing the primary tumor and affected lymph nodes and preventing disease progression. In some cases, surgery may also be required to remove the uterus and affected lymph nodes. | C | C | C |
| BioMedLM 2.7B (With prompt) | 82. In cervical cancer treatment, if the stage is ⅣA with lymph node enlargement, what treatment measures should be taken? ① Surgery ② Chemotherapy ③ Radiotherapy ④ Others | D | D | D |
| BioMedLM 2.7B | 82. In cervical cancer treatment, if the stage is ⅣA with lymph node enlargement, what treatment measures should be taken? ### Response: ① Chemotherapy ② Radiotherapy ③ Surgery ④ Chemotherapy and radiotherapy ⑤ Surgery and radiotherapy ⑥ Chemotherapy, radiotherapy, and surgery ⑦ Others | D | D | D |
| Microsoft Phi2 (With prompt) | 82.In cervical cancer treatment, if the stage is ⅣA with lymph node enlargement, the treatment measures should include surgery, radiation therapy, and chemotherapy. Surgery is the primary treatment for stage ⅣA cervical cancer, which involves the removal of the uterus, cervix, and nearby lymph nodes. Radiation therapy may also be used to target any remaining cancer cells in the pelvic area. Chemotherapy is typically used in combination with surgery and radiation therapy to kill any cancer cells that may have spread beyond the pelvic area. | B | B | B |
| Microsoft Phi2 | 82. If the stage is ⅣA with lymph node enlargement, the treatment measures should include surgery to remove the tumor and surrounding tissue, followed by radiation therapy and chemotherapy. | D | D | D |
| QiZhenGPT (With prompt) | 82. In cervical cancer treatment, if the stage is IVA with lymph node enlargement, the treatment plan may include surgery, radiation therapy, or a combination of both. The specific treatment will depend on the patient's individual circumstances, such as their age, health status, and overall health. | C | C | C |
| QiZhenGPT | 82. For stage IVA cervical spine cancer with lymph node enlargement, treatment usually includes surgery, radiotherapy, and chemotherapy. Surgery remains the treatment of choice and may require more extensive resection to prevent lymphatic metastasis. Radiotherapy and chemotherapy may be used as adjuvant therapy to help shrink the tumor. | D | D | D |
| **83. In cervical cancer treatment, if the stage is ⅣB, what treatment measures should be taken?** | | | | |
| ChatGPT4.0 turbo (With prompt) | 83. Stage IVB cervical cancer, characterized by distant metastasis, requires a more systemic approach to treatment. The primary modalities include chemotherapy and/or targeted therapy, depending on the specific characteristics of the tumor and the presence of specific molecular targets. Palliative care to manage symptoms and maintain quality of life is also a key component of treatment at this stage. Radiation therapy may be used to palliate symptoms such as pain or bleeding. | A | A | A |
| ChatGPT4.0 turbo | 83. Stage IVB: For stage IVB cervical cancer, where the cancer has spread to distant organs, the primary aim of treatment is usually palliative, to relieve symptoms and improve quality of life rather than to cure the disease. Treatment may involve chemotherapy, targeted therapy, or immunotherapy, depending on the individual's specific situation and the characteristics of the tumor. Participation in clinical trials may also be an option for patients with stage IVB cervical cancer. | B | B | B |
| Claude 2 (With prompt) | 83. For stage IVB metastatic cervical cancer, systemic chemotherapy is the foundation of treatment, most commonly carboplatin/paclitaxel doublets. Additional local therapy such as pelvic radiation may be used for palliative purposes in highly selected cases. | A | A | A |
| Claude 2 | 83. For stage IVB: The cancer has metastasized beyond the pelvis. The goals of treatment are palliative, using chemotherapy, radiation, or both for symptom relief. Fertility is not preserved and survival is limited due to disseminated disease. | A | A | A |
| Gemini Pro (With prompt) | 83. Palliative care | A | A | A |
| Gemini Pro | 83. **Stage ⅣB**:  - **Palliative chemotherapy** or **palliative radiotherapy** to relieve symptoms and improve quality of life. | A | A | A |
| Mistral-7B-v0.2 (With prompt) | 83.In cervical cancer treatment, if the stage is ⅣB, a combination of chemotherapy and radiation therapy is the recommended primary treatment. This approach can help to shrink the tumor and destroy cancer cells, potentially allowing for a response to subsequent treatment. In some cases, surgery may also be considered as a follow-up treatment after chemotherapy and radiation therapy have been completed. | A | A | A |
| Mistral-7B-v0.2 | 83. For cervical cancer stage ⅣB, the treatment measures typically include: - Radiation therapy: External beam radiation therapy (EBRT) or internal radiation therapy (brachytherapy) or a combination of both. - Chemotherapy: Concurrent chemoradiation therapy (CCRT) with platinum-based drugs like cisplatin or carboplatin. - Surgery: Surgery may be considered after radiation therapy, but this is usually done for smaller tumors and depends on the response to radiation. - Lymph node dissection: Pelvic lymph node dissection may be performed to remove involved lymph nodes. - Targeted therapy: For patients with specific biomarkers, targeted therapies like bevacizumab may be considered. - Immunotherapy: For patients with advanced disease, immunotherapy with checkpoint inhibitors like pembrolizumab may be considered. | B | B | B |
| Starling-LM-7B alpha (With prompt) | 83. In cervical cancer treatment, if the stage is ⅣB, the treatment measures that should be taken include:   a. Radical hysterectomy: This is a surgical procedure that involves the removal of the uterus, cervix, and surrounding tissues, including the parametria, upper vagina, and lymph nodes. This procedure is typically performed by a gynecologic oncologist.   b. Pelvic lymph node dissection: This is a surgical procedure that involves the removal of lymph nodes in the pelvic region to assess for the presence of cancer cells. This procedure is often performed in conjunction with a radical hysterectomy.   c. Chemotherapy: This is a systemic treatment that involves the use of one or more anticancer drugs to kill cancer cells. Chemotherapy may be administered before, during, or after surgery, depending on the specific circumstances of the patient's case.   d. Radiation therapy: This is a localized treatment that uses high-energy rays to kill cancer cells. Radiation therapy may be administered externally (external beam radiation therapy) or internally (brachytherapy) depending on the patient's needs.   e. Concurrent chemoradiation: This is a combination treatment that involves the administration of chemotherapy and radiation therapy simultaneously. This approach has been shown to improve outcomes in patients with advanced cervical cancer.  In all cases, the specific treatment plan should be tailored to the individual patient's needs, taking into account factors such as age, overall health, tumor characteristics, and patient preferences. It is essential for patients to discuss their treatment options with their healthcare team, including a gynecologic oncologist, to make informed decisions about their care. | C | C | C |
| Starling-LM-7B alpha | 83. For stage ⅣB cervical cancer, treatment measures typically include a combination of radiation therapy, such as EBRT and brachytherapy, and concurrent chemotherapy. The specific treatment plan will depend on the patient's overall health, age, and the extent of the cancer. In some cases, surgery may also be considered, particularly if the cancer is confined to the cervix and has not spread to other organs. | C | D | C |
| HuatuoGPT (With prompt) | 83. In the treatment of cervical cancer, what treatment measures should be taken if the stage is IV B? For stage IV B cervical cancer, the treatment modality mainly depends on the patient's overall health status and personal preference. In general, combination chemotherapy and radiotherapy remain the mainstay of treatment, aiming to control the progression of the disease and improve the survival rate and quality of life of patients. In addition, some patients may consider participating in clinical trials to try new treatments and drugs for better results. The treatment plan should be formulated by a professional medical team with full communication and joint decision-making with the patient. | A | A | A |
| HuatuoGPT | 83. If the patient is in Stage IVB, the conventional treatment is aggressive cancer therapy. This includes radiation therapy and/or combination chemotherapy aimed at removing the primary tumor and any involved lymph nodes and preventing disease progression. In some cases, surgery may also be required to remove the uterus and affected lymph nodes. | D | D | D |
| BioMedLM 2.7B (With prompt) | 83. In cervical cancer treatment, if the stage is ⅣB, what treatment measures should be taken? ① Surgery ② Chemotherapy ③ Radiotherapy ④ Others | D | D | D |
| BioMedLM 2.7B | 83. In cervical cancer treatment, if the stage is ⅣB, what treatment measures should be taken? ### Response: Chemotherapy Radiotherapy Surgery Others (please specify) | D | D | D |
| Microsoft Phi2 (With prompt) | 83.In cervical cancer treatment, if the stage is ⅣB, the treatment measures should include surgery, radiation therapy, and chemotherapy. Surgery is the primary treatment for stage ⅣB cervical cancer, which involves the removal of the uterus, cervix, and nearby lymph nodes. Radiation therapy may also be used to target any remaining cancer cells in the pelvic area. Chemotherapy is typically used in combination with surgery and radiation therapy to kill any cancer cells that may have spread beyond the pelvic area. | D | D | D |
| Microsoft Phi2 | 83. If the stage is ⅣB, the treatment measures should include surgery to remove the tumor and surrounding tissues, followed by radiation therapy to kill any remaining cancer cells. | D | C | D |
| QiZhenGPT (With prompt) | 83. In cervical cancer treatment, if the stage is IVB, the recommended treatment for IVB cervical cancer is a combination of surgery, radiation therapy, and chemotherapy. The treatment plan may also include targeted therapy or immune therapy if appropriate. | C | C | C |
| QiZhenGPT | 83. For stage IVB cervical spine cancer, treatment measures usually include surgery, radiotherapy and chemotherapy. Surgery may be the treatment of choice, especially for patients who may be cured at an early stage. Radiotherapy and chemotherapy may be used as complementary treatments to surgery or for patients who are inoperable. In some cases, targeted therapy and immunotherapy may also be used to treat cervical spine cancer. | D | D | D |
| **84. After radical surgery for early cervical cancer, if the abdominal aortic lymph nodes are negative but high-risk factors are present, what treatment measures should be taken?** | | | | |
| ChatGPT4.0 turbo (With prompt) | 84. After radical surgery for early-stage cervical cancer where the abdominal aortic lymph nodes are negative but high-risk factors (such as positive surgical margins, deep stromal invasion, or lymphovascular space invasion) are present, adjuvant therapy is recommended. This typically includes chemoradiation, combining external beam radiation therapy with concurrent chemotherapy, usually cisplatin-based. This approach is aimed at reducing the risk of recurrence and improving overall survival by addressing microscopic residual disease. | A | A | A |
| ChatGPT4.0 turbo | 84. Highrisk factors present, negative paraaortic lymph nodes: Highrisk factors typically include large tumor size (>4 cm), deep stromal invasion, or lymphovascular space invasion (LVSI). If any of these highrisk factors are present, the standard of care would usually involve adjuvant concurrent chemoradiation (combination of chemotherapy and radiation therapy). This approach aims to reduce the risk of recurrence by treating both the local area and any potential microscopic disease that may remain. | B | B | B |
| Claude 2 (With prompt) | 84. For high-risk early stage cervical cancer after radical surgery with negative para-aortic lymph nodes, adjuvant chemoradiation or radiation alone is recommended based on pathologic risk factors. | A | A | A |
| Claude 2 | 84. If abdominal aortic lymph nodes are negative but high-risk factors are present (e.g. positive pelvic lymph nodes, parametrial invasion, positive margins): Adjuvant pelvic radiation ± concurrent cisplatin-based chemotherapy is recommended. | A | A | A |
| Gemini Pro (With prompt) | 84. Adjuvant pelvic radiotherapy | A | A | A |
| Gemini Pro | 84. **After radical surgery for early cervical cancer, if the abdominal aortic lymph nodes are negative but high-risk factors are present**:  - **Adjuvant pelvic radiotherapy**. | B | B | B |
| Mistral-7B-v0.2 (With prompt) | 84.After radical surgery for early cervical cancer, if the abdominal aortic lymph nodes are negative but high-risk factors are present, close monitoring with regular follow-up appointments and cervical cancer screening tests is recommended. These tests may include pelvic examinations, Pap tests, HPV testing, and imaging studies such as CT scans or MRI scans. If any abnormalities are detected, further treatment may be necessary. | D | D | D |
| Mistral-7B-v0.2 | 84. If the abdominal aortic lymph nodes are negative but high-risk factors are present after radical surgery for early cervical cancer, additional treatment measures may include: - Radiation therapy: External beam radiation therapy (EBRT) or brachytherapy (internal radiation) may be recommended to help reduce the risk of recurrence. - Chemotherapy: Some doctors may recommend chemotherapy to help kill any remaining cancer cells and reduce the risk of recurrence. | A | A | A |
| Starling-LM-7B alpha (With prompt) | 84. After radical surgery for early cervical cancer, if the abdominal aortic lymph nodes are negative but high-risk factors are present, the treatment measures that should be taken include:  a) Close follow-up with regular pelvic examinations, cervical cytology, and imaging studies to monitor for recurrence or progression of the disease. b) Consideration of adjuvant radiation therapy or chemotherapy, depending on the specific high-risk factors and the patient's overall health status. This decision should be made in consultation with a multidisciplinary team of specialists. c) Patient education regarding lifestyle modifications, such as smoking cessation, maintaining a healthy diet, and regular exercise, which may help reduce the risk of recurrence. | C | C | C |
| Starling-LM-7B alpha | 84. If the abdominal aortic lymph nodes are negative but high-risk factors are present, additional treatment measures may include: - Adjuvant chemotherapy and/or radiation therapy to reduce the risk of recurrence. - Close follow-up with regular imaging and clinical examinations to monitor for any signs of recurrence. - Consideration of clinical trials or experimental treatments if appropriate. | B | B | B |
| HuatuoGPT (With prompt) | 84. If radical surgery is performed for early-stage cervical cancer but the abdominal aortic lymph nodes are negative, but high-risk factors exist, what treatment measures should be taken? Ans: 16. If radical surgery is performed for early-stage cervical cancer but the abdominal aortic lymph nodes are negative but high-risk factors are present, the following therapeutic measures should be taken: (a) hysteroscopic cervical conization (conization), (b) total hysterectomy (hysterectomy), (c) radiotherapy, and (d) chemotherapy. These treatments should be decided on the basis of the patient's specific condition and the doctor's judgment. | B | B | B |
| HuatuoGPT | 84. After radical surgery for early-stage cervical cancer, if the abdominal aortic lymph nodes are negative but high-risk factors are present, conventional treatment measures usually include chemotherapy and/or radiotherapy. This is to reduce the risk of disease recurrence and to improve patient survival. The specific regimen will be based on factors such as the patient's age, overall health and cancer stage. | B | B | B |
| BioMedLM 2.7B (With prompt) | 84. After radical surgery for early cervical cancer, if the abdominal aortic lymph nodes are negative but high-risk factors are present, what treatment measures should be taken? The answer to this question is "yes." The risk factors for recurrence include lymphovascular space invasion (LVSI), positive surgical margins, and parametrial involvement. The risk factors for distant metastasis include LVSI, positive surgical margins, and lymph node metastasis. | C | D | D |
| BioMedLM 2.7B | 84. After radical surgery for early cervical cancer, if the abdominal aortic lymph nodes are negative but high-risk factors are present, what treatment measures should be taken? ### Response: Radiotherapy and chemotherapy. | B | B | B |
| Microsoft Phi2 (With prompt) | 84. If the abdominal aortic lymph nodes are negative but high-risk factors are present, the patient should be treated with adjuvant chemotherapy. | A | A | A |
| Microsoft Phi2 | 84.If the abdominal aortic lymph nodes are negative but high-risk factors are present, the patient should receive adjuvant chemotherapy. | A | B | B |
| QiZhenGPT (With prompt) | 84. After radical surgery for early cervical cancer, if the abdominal aortic lymph nodes are negative but high-risk factors are present, what treatment measures should be taken? Answer: The treatment measures should be considered based on the presence of high-risk factors for recurrence and the stage of the disease. | D | C | C |
| QiZhenGPT | 84. After radical surgery for early cervical cancer, if the abdominal aortic lymph nodes are negative but high-risk factors are present, treatment measures that may be considered include:  Close surveillance and regular follow-up with the cancer team to monitor for recurrence  Participation in clinical trials that focus on the prevention of cervical cancer recurrence  Additional therapies such as chemotherapy, radiation therapy, or brachytherapy may be recommended based on the individual patient's needs and circumstances | A | A | A |
| **85. After radical surgery for early cervical cancer, if the abdominal aortic lymph nodes are negative but intermediate-risk factors are present, what treatment measures should be taken?** | | | | |
| ChatGPT4.0 turbo (With prompt) | 85. If intermediate-risk factors are present after radical surgery for early cervical cancer, and the abdominal aortic lymph nodes are negative, the recommended treatment is usually adjuvant radiation therapy alone. Intermediate-risk factors might include large tumor size, deep cervical stromal invasion, or lymphovascular space invasion. The objective of adjuvant radiation is to reduce the risk of local recurrence by treating potential microscopic residual disease in the pelvis. | B | B | B |
| ChatGPT4.0 turbo | 85. Intermediaterisk factors present, negative paraaortic lymph nodes: Intermediaterisk factors are generally defined by the presence of features such as moderate stromal invasion, lymphovascular space invasion (LVSI), or tumor size between 24 cm. When these factors are present, the patient may benefit from adjuvant radiation therapy to the pelvis to reduce the risk of local recurrence. This is sometimes referred to as pelvic radiotherapy. The use of chemotherapy in this setting is less clear and would depend on the individual patient's risk profile and the treating physician's judgment. | B | C | B |
| Claude 2 (With prompt) | 85. In intermediate-risk disease with negative lymph nodes, observation is reasonable but chemotherapy or radiation can be considered if multiple intermediate risk factors present. | A | A | A |
| Claude 2 | 85. If abdominal aortic lymph nodes are negative and intermediate-risk factors are present (e.g. large tumor size, deep stromal invasion): Adjuvant pelvic radiation may be considered, especially if multiple intermediate risk factors are present. | A | B | B |
| Gemini Pro (With prompt) | 85. Close follow-up | C | C | C |
| Gemini Pro | 85. **After radical surgery for early cervical cancer, if the abdominal aortic lymph nodes are negative but intermediate-risk factors are present**:  - **Close follow-up** with regular pelvic examinations, Pap tests, and imaging studies. Adjuvant therapy may be considered if there are any signs of recurrence or progression. | B | B | B |
| Mistral-7B-v0.2 (With prompt) | 85.After radical surgery for early cervical cancer, if the abdominal aortic lymph nodes are negative but intermediate-risk factors are present, close monitoring with regular follow-up appointments and cervical cancer screening tests is also recommended. The frequency and duration of follow-up appointments may depend on the specific risk factors and individual patient circumstances. | B | C | B |
| Mistral-7B-v0.2 | 85. If the abdominal aortic lymph nodes are negative but intermediate-risk factors are present after radical surgery for early cervical cancer, additional treatment measures may include: - Radiation therapy: EBRT or brachytherapy may be recommended to help reduce the risk of recurrence. - Chemotherapy: Some doctors may recommend chemotherapy to help kill any remaining cancer cells and reduce the risk of recurrence. The decision to use chemotherapy in this situation is often based on the specific intermediate-risk factors present. | A | A | A |
| Starling-LM-7B alpha (With prompt) | 85. After radical surgery for early cervical cancer, if the abdominal aortic lymph nodes are negative but intermediate-risk factors are present, the treatment measures that should be taken include:  a) Close follow-up with regular pelvic examinations, cervical cytology, and imaging studies to monitor for recurrence or progression of the disease. b) Consideration of adjuvant radiation therapy or chemotherapy, depending on the specific intermediate-risk factors and the patient's overall health status. This decision should be made in consultation with a multidisciplinary team of specialists. c) Patient education regarding lifestyle modifications, such as smoking cessation, maintaining a healthy diet, and regular exercise, which may help reduce the risk of recurrence. | A | A | A |
| Starling-LM-7B alpha | 85. If the abdominal aortic lymph nodes are negative but intermediate-risk factors are present, additional treatment measures may include: - Adjuvant chemotherapy and/or radiation therapy to reduce the risk of recurrence. - Close follow-up with regular imaging and clinical examinations to monitor for any signs of recurrence. - Consideration of clinical trials or experimental treatments if appropriate. | B | B | B |
| HuatuoGPT (With prompt) | 85. If radical surgery is performed for early-stage cervical cancer but the abdominal aortic lymph nodes are negative, but intermediate risk factors are present, what treatment measures should be taken? Ans: 17. If radical surgery is performed for early-stage cervical cancer but the abdominal aortic lymph nodes are negative but intermediate risk factors are present, the following therapeutic measures should be taken: (a) hysteroscopic conization of the cervix (conization), (b) subtotal hysterectomy (subtotal uterine resection), (c) radiotherapy, and (d) chemotherapy. These treatments should be decided according to the patient's specific situation and the doctor's judgment. | C | C | C |
| HuatuoGPT | 85. After radical surgery for early-stage cervical cancer, if the abdominal aortic lymph nodes are negative but intermediate risk factors are present, conventional treatment measures usually also include chemotherapy and/or radiotherapy. This is to reduce the risk of disease recurrence and to improve patient survival. The specific regimen will be based on factors such as the patient's age, overall health and cancer stage. | A | B | B |
| BioMedLM 2.7B (With prompt) | 85. After radical surgery for early cervical cancer, if the abdominal aortic lymph nodes are negative but intermediate-risk factors are present, what treatment measures should be taken? The answer is "Yes." The patient has a history of smoking and diabetes. The patient has a history of hypertension and hyperlipidemia. The patient has a history of smoking and diabetes. The patient has a history of hypertension and hyperlipidemia. | D | D | D |
| BioMedLM 2.7B | 85. After radical surgery for early cervical cancer, if the abdominal aortic lymph nodes are negative but intermediate-risk factors are present, what treatment measures should be taken? ### Response: 1\. No further treatment. 2\. Chemotherapy. 3\. Radiotherapy. 4\. Chemotherapy and radiotherapy. 5\. Radiotherapy and chemotherapy. 6\. Chemotherapy and radiotherapy and brachytherapy. | D | D | D |
| Microsoft Phi2 (With prompt) | 85. If the abdominal aortic lymph nodes are negative but intermediate-risk factors are present, the patient should be treated with adjuvant chemotherapy. | B | B | B |
| Microsoft Phi2 | 85.If the abdominal aortic lymph nodes are negative but intermediate-risk factors are present, the patient should receive adjuvant chemotherapy. | B | C | B |
| QiZhenGPT (With prompt) | 85. After radical surgery for early cervical cancer, if the abdominal aortic lymph nodes are negative but intermediate-risk factors are present, what treatment measures should be taken? Answer: The treatment measures should be considered based on the presence of intermediate-risk factors for recurrence and the stage of the disease. | D | D | D |
| QiZhenGPT | 85. After radical surgery for early cervical cancer, if the abdominal aortic lymph nodes are intermediate-risk factors, treatment measures that may be considered include:  Close surveillance and regular follow-up with the cancer team to monitor for recurrence  Participation in clinical trials that focus on the prevention of cervical cancer recurrence  Additional therapies such as chemotherapy, radiation therapy, or brachytherapy may be recommended based on the individual patient's needs and circumstances | B | B | B |
| **86. After radical surgery for early cervical cancer, if the abdominal aortic lymph nodes are positive but there is no distant metastasis, what treatment measures should be taken?** | | | | |
| ChatGPT4.0 turbo (With prompt) | 86. In cases where radical surgery for early cervical cancer results in positive abdominal aortic lymph nodes but no distant metastasis, the recommended treatment is extended-field chemoradiation. This involves radiation therapy that includes the pelvis and para-aortic region, combined with concurrent chemotherapy. This treatment aims to control locoregional disease and address the risk of micrometastatic spread. | A | A | A |
| ChatGPT4.0 turbo | 86. Positive paraaortic lymph nodes, no distant metastasis: The discovery of positive paraaortic lymph nodes indicates a more advanced disease and a higher risk of recurrence. The standard treatment in this case would typically involve extendedfield radiation therapy to include the paraaortic region along with concurrent chemotherapy. This combined modality treatment aims to target both the local and regional areas where cancer has spread. After completing chemoradiation, some patients may also receive additional chemotherapy to further reduce the risk of distant metastasis. | A | A | A |
| Claude 2 (With prompt) | 86. For patients with positive para-aortic lymph nodes after radical surgery without distant metastasis, recommendation is adjuvant extended-field chemoradiation targeting the para-aortic region. | A | A | A |
| Claude 2 | 86. If abdominal aortic lymph nodes are positive but there is no distant metastasis: This indicates regional nodal spread. Recommended treatment is adjuvant extended-field radiation therapy to cover pelvis and para-aortic nodes ± concurrent cisplatin-based chemotherapy. This helps reduce risk of regional recurrence. | A | A | A |
| Gemini Pro (With prompt) | 86. Adjuvant chemoradiotherapy | B | B | B |
| Gemini Pro | 86. **After radical surgery for early cervical cancer, if the abdominal aortic lymph nodes are positive but there is no distant metastasis**:  - **Adjuvant chemoradiation**. | C | C | C |
| Mistral-7B-v0.2 (With prompt) | 86.After radical surgery for early cervical cancer, if the abdominal aortic lymph nodes are positive but there is no distant metastasis, additional treatment such as radiation therapy or chemotherapy may be recommended to reduce the risk of recurrence. The specific treatment plan will depend on the extent of lymph node involvement and other individual factors. | A | A | A |
| Mistral-7B-v0.2 | 86. If the abdominal aortic lymph nodes are positive but there is no distant metastasis after radical surgery for early cervical cancer, additional treatment measures may include: - Radiation therapy: EBRT and/or brachytherapy may be recommended to help reduce the risk of recurrence. - Chemotherapy: Chemotherapy may be recommended to help kill any remaining cancer cells and reduce the risk of recurrence. The specific chemotherapy regimen and duration will depend on the size and number of positive lymph nodes. | A | A | A |
| Starling-LM-7B alpha (With prompt) | 86. After radical surgery for early cervical cancer, if the abdominal aortic lymph nodes are positive but there is no distant metastasis, the treatment measures that should be taken include:  a) Completion of the planned adjuvant radiation therapy or chemotherapy, as determined by the multidisciplinary team of specialists, to target any remaining microscopic disease. b) Close follow-up with regular pelvic examinations, cervical cytology, and imaging studies to monitor for recurrence or progression of the disease. c) Patient education regarding lifestyle modifications, such as smoking cessation, maintaining a healthy diet, and regular exercise, which may help reduce the risk of recurrence. | D | D | D |
| Starling-LM-7B alpha | 86. If the abdominal aortic lymph nodes are positive but there is no distant metastasis, additional treatment measures may include: - Adjuvant chemotherapy and/or radiation therapy to reduce the risk of recurrence. - Close follow-up with regular imaging and clinical examinations to monitor for any signs of recurrence. - Consideration of clinical trials or experimental treatments if appropriate. | B | B | B |
| HuatuoGPT (With prompt) | 86. If radical surgery is performed for early-stage cervical cancer but the abdominal aortic lymph nodes are positive but there are no distant metastases, what treatment measures should be taken? Ans: 18. If radical surgery is performed for early-stage cervical cancer but the abdominal aortic lymph nodes are positive but there is no distant metastasis, the following therapeutic measures should be taken: (a) pelvic lymphadenectomy (pelvic lymph node dissection), (b) radiotherapy, and (c) chemotherapy. These therapeutic measures should be decided on the basis of the patient's specific condition and the physician's judgment. | A | B | A |
| HuatuoGPT | 86. After radical surgery for early-stage cervical cancer, if the abdominal aortic lymph nodes are positive but there are no distant metastases, the conventional treatment measure is usually chemotherapy. This is to reduce the risk of disease recurrence and to improve patient survival. The exact regimen will be based on factors such as the patient's age, overall health and cancer stage. | C | C | C |
| BioMedLM 2.7B (With prompt) | 86. After radical surgery for early cervical cancer, if the abdominal aortic lymph nodes are positive but there is no distant metastasis, what treatment measures should be taken? The answer is "No adjuvant therapy is recommended." | D | D | D |
| BioMedLM 2.7B | 86. After radical surgery for early cervical cancer, if the abdominal aortic lymph nodes are positive but there is no distant metastasis, what treatment measures should be taken? ### Response: 1\. Chemotherapy 2\. Radiotherapy 3\. Chemotherapy and radiotherapy 4\. No treatment | D | D | D |
| Microsoft Phi2 (With prompt) | 86.If the abdominal aortic lymph nodes are positive but there is no distant metastasis, the patient should be treated with adjuvant chemotherapy. | B | B | B |
| Microsoft Phi2 | 86.If the abdominal aortic lymph nodes are positive but there is no distant metastasis, the patient should receive adjuvant chemotherapy. | C | C | C |
| QiZhenGPT (With prompt) | 86. After radical surgery for early cervical cancer, if the abdominal aortic lymph nodes are positive but there is no distant metastasis, what treatment measures should be taken? Answer: The treatment measures should be considered based on the stage of the disease and the presence of distant metastasis. | D | D | D |
| QiZhenGPT | 86. After radical surgery for early cervical cancer, if the abdominal aortic lymph nodes are positive but there is no distant metastasis, treatment measures that may be considered include:  Close surveillance and regular follow-up with the cancer team to monitor for recurrence  Participation in clinical trials that focus on cervical cancer follow-up and treatment  Additional therapies such as chemotherapy, radiation therapy, or brachytherapy may be recommended based on the individual patient's needs and circumstances It is important to note that the specific treatment plan for each individual patient will depend on a variety of factors, including the stage and location of the cancer, the patient's overall health, and their personal preferences. It is recommended that patients discuss their treatment options with their cancer care team to determine the best course of action. | B | C | C |
| **87. What are the surgical treatment options for patients with FIGO stage IA1 cervical cancer? Are they suitable for patients with preserved fertility?** | | | | |
| ChatGPT4.0 turbo (With prompt) | 87. **Surgical Treatment Options for FIGO Stage IA1**: For patients with FIGO stage IA1 cervical cancer, conservative surgical options such as cone biopsy or simple trachelectomy may be sufficient, especially if fertility preservation is a concern. These procedures are typically suitable for patients with tumors less than 3 mm in depth and without LVSI. If fertility is not a concern, a more radical hysterectomy might be considered, depending on the patient's age, desire for fertility preservation, and other individual factors. The choice of surgical method should be carefully discussed with the patient, considering her reproductive plans and the specifics of the tumor | A | A | A |
| ChatGPT4.0 turbo | 87. Surgical Treatment Options for FIGO Stage IA1 Cervical Cancer For FIGO stage IA1 cervical cancer, especially in patients desiring to preserve fertility, the following surgical options are considered:   Conization: Either cold knife conization or LEEP, which can be both diagnostic and therapeutic.  Simple Hysterectomy: For women who do not wish to preserve fertility, although this is often considered overtreatment for IA1 without LVSI.  FertilitySparing Surgery: Such as trachelectomy (removal of the cervix while preserving the uterus), which is suitable for patients who wish to preserve fertility and have tumors that meet specific size and invasion criteria. Each of these options requires careful patient counseling regarding the risks, benefits, and potential impact on future | C | C | C |
| Claude 2 (With prompt) | 87. Standard treatment options for IA1 disease are cone biopsy or extrafascial hysterectomy. Fertility preservation is feasible as lymph node dissection is not indicated for these early small volume cancers without lymphovascular infiltration. | B | B | B |
| Claude 2 | 87. For FIGO IA1 cervical cancer, standard treatment options aimed at preserving fertility include cervical conization (cold knife or LEEP) or simple trachelectomy. Radical trachelectomy may be an option in select cases. Fertility preservation options should be discussed prior to surgery. | A | A | A |
| Gemini Pro (With prompt) | 87. What are the surgical treatment options for patients with FIGO stage IA1 cervical cancer? Are they suitable for patients with preserved fertility?   Surgical treatment options:  Conization: Removal of a cone-shaped piece of tissue from the cervix.  Trachelectomy: Removal of the cervix while preserving the uterus.  Suitability for patients with preserved fertility: Both conization and trachelectomy are suitable for patients with preserved fertility. The choice of procedure depends on the size and location of the tumor, as well as the patient's fertility goals. | A | A | A |
| Gemini Pro | 87. **What are the surgical treatment options for patients with FIGO stage IA1 cervical cancer? Are they suitable for patients with preserved fertility?** The surgical treatment options for patients with FIGO stage IA1 cervical cancer are: * **Conization:** Conization is a procedure that removes a cone-shaped piece of tissue from the cervix. It is the most common surgical treatment for FIGO stage IA1 cervical cancer. Conization is suitable for patients with preserved fertility. * **Simple hysterectomy:** A simple hysterectomy is a procedure that removes the uterus. It is a more radical treatment than conization, but it is also more effective. Simple hysterectomy is not suitable for patients with preserved fertility. * **Radical hysterectomy:** A radical hysterectomy is a procedure that removes the uterus, cervix, and surrounding tissues. It is the most radical treatment for FIGO stage IA1 cervical cancer, but it is also the most effective. Radical hysterectomy is not suitable for patients with preserved fertility. | A | A | A |
| Mistral-7B-v0.2 (With prompt) | 87.The surgical treatment options for patients with FIGO stage IA1 cervical cancer include cone biopsy, LEEP, or simple hysterectomy. These procedures are suitable for patients with preserved fertility, as they allow for the preservation of the uterus and cervix in most cases. However, the specific treatment option recommended will depend on the size and location of the cancer, as well as the patient's individual circumstances and preferences. | A | B | A |
| Mistral-7B-v0.2 | 87. Consideration of lymphovascular infiltration and recommendations for FIGO stage IA1 cervical cancer: Lymphovascular invasion refers to the infiltration of cancer cells into the lymphatic and/or vascular channels. Although the presence of lymphovascular invasion is considered a poor prognostic factor for cervical cancer, the significance during FIGO stage IA1 is still debated, given that the risk of metastasis is low. A few studies suggest that women with lymphovascular invasion may benefit from more aggressive treatment and follow-up, though more research is needed to fully understand its impact on patient outcomes. In general, FIGO stage IA1 cervical cancer cases do not require extensive evaluation for lymph node status, though some experts consider that sentinel lymph node biopsy may be helpful in selected patients. The decision to perform this procedure depends on factors such as the size of the lesion, the depth of invasion, and the risk of occult nodal metastasis. In most cases, women with FIGO stage IA1 cervical cancer are closely monitored through regular follow-up appointments, and the routine inclusion of lymph node evaluation in this early stage is debated. Surgical treatment options for patients with FIGO stage IA1 cervical cancer and fertility preservation: Traditional total abdominal hysterectomy: This involves removing the entire uterus and cervix, often with nearby pelvic lymph nodes. Traditional hysterectomy offers excellent cancer control but permanently ends a woman's reproductive potential. Minimally invasive surgery, such as laparoscopic or robotic-assisted radical hysterectomy: The surgical approach is similar to traditional hysterectomy but uses minimally invasive techniques, minimizing the number of incisions and reducing postoperative recovery time. While minimally invasive surgery may help preserve surrounding structures and potentially improve fertility prospects, not all women are suitable candidates, as criteria for candidacy can be strict due to the importance of complete surgical excision and accurate staging. Tranchectomy: This alternative treatment involves removing the cancerous tissue while preserving the uterine cervix and preserving fertility. It is a promising treatment, but it requires meticulous surgical technique, careful selection of patients, and long-term follow-up to assess efficacy and safety. Current evidence supports its use as a potential alternative to hysterectomy in select cases of FIGO stage IA1 cervical cancers. | C | D | D |
| Starling-LM-7B alpha (With prompt) | 87. Surgical treatment options for patients with FIGO stage IA1 cervical cancer include simple hysterectomy, radical trachelectomy, and laparoscopic or robotic-assisted radical hysterectomy. These procedures are suitable for patients with preserved fertility, as they aim to preserve the ovaries and uterus while removing the cervix and any involved lymph nodes. The choice of procedure depends on the patient's age, desire for future fertility, and the presence of risk factors such as LVSI. | D | D | D |
| Starling-LM-7B alpha | 87. Surgical treatment options for patients with FIGO stage IA1 cervical cancer include simple hysterectomy, radical trachelectomy, and laparoscopic radical hysterectomy. These surgical options are suitable for patients with preserved fertility, as they aim to preserve the uterus while removing the cervix and any involved lymph nodes. | D | D | D |
| HuatuoGPT (With prompt) | 87. What surgical options are available for patients with FIGO stage IA1 cervical cancer? Are these options suitable for patients who maintain fertility? For patients with FIGO stage IA1 cervical cancer, conservative treatment, including surgical resection (conization or hysterectomy) and radiotherapy, is usually used. For patients who wish to maintain their fertility, a hysterectomy followed by vaginal cervical repair may be considered. However, this procedure can have an impact on the patient's fertility and therefore needs to be carefully considered and the associated risks and benefits discussed with the patient. | C | C | C |
| HuatuoGPT | 87. For patients with FIGO-staged IA1 cervical cancer, surgical treatment options usually include cervical conization (conization) and hysterectomy (hysterectomy). For younger patients who still retain their fertility, the option of cervical conization may be more appropriate, as this procedure preserves uterine and ovarian function and has less impact on fertility. However, treatment options should also take into account factors such as the patient's overall health, age and personal wishes. | A | A | A |
| BioMedLM 2.7B (With prompt) | 87. What are the surgical treatment options for patients with FIGO stage IA1 cervical cancer? Are they suitable for patients with preserved fertility? The surgical treatment options for patients with FIGO stage IA1 cervical cancer include simple hysterectomy, radical hysterectomy, and radical trachelectomy. The choice of surgical treatment is based on the patient's age, desire for future fertility, and the extent of disease. The patient's age is the most important factor in determining the surgical treatment. The patient's age should be considered when choosing the surgical treatment. The patient's age should be considered when choosing the surgical treatment. | D | D | D |
| BioMedLM 2.7B | 87. What are the surgical treatment options for patients with FIGO stage IA1 cervical cancer? Are they suitable for patients with preserved fertility? ### Response: FIGO stage IA1 cervical cancer is defined as a tumor confined to the cervix, with a maximum diameter of ≤4 cm. The standard treatment for FIGO stage IA1 cervical cancer is radical hysterectomy. However, in patients who wish to preserve their fertility, radical trachelectomy (RT) is an alternative treatment option. RT is a surgical procedure that involves removing the cervix and upper vagina, and preserving the uterus and ovaries. The procedure is performed by removing the cervix and upper vagina, and preserving the uterus and ovaries. | D | D | D |
| Microsoft Phi2 (With prompt) | 87. The surgical treatment options for patients with FIGO stage IA1 cervical cancer include radical hysterectomy, radical trachelectomy, and radical trachelectomy with pelvic lymphadenectomy. These procedures are suitable for patients with preserved fertility. | D | D | D |
| Microsoft Phi2 | 87. The surgical treatment options for patients with FIGO stage IA1 cervical cancer include radical hysterectomy, radical trachelectomy, and radical trachelectomy with pelvic lymphadenectomy. These treatments are suitable for patients with preserved fertility. | D | D | D |
| QiZhenGPT (With prompt) | 87. What are the surgical treatment options for patients with FIGO stage IA1 cervical cancer? Are they suitable for patients with preserved fertility? Answer: The surgical treatment options for patients with FIGO stage IA1 cervical cancer are colposcopy-guided biopsy, surgery, and radiation therapy. The treatment options are tailored to the individual patient and may include a combination of these approaches. The decision to perform surgery or radiation therapy is based on the presence of any abnormalities on colposcopy and the stage of the disease. The treatment options are suitable for patients with preserved fertility, as long as the patient is willing and able to undergo surgery. | D | D | D |
| QiZhenGPT | 87. The surgical treatment options for patients with stage IA1 cervical cancer include surgery alone or surgery in combination with radiation therapy or chemotherapy. They are suitable for patients with preserved fertility. | C | C | C |
| **88. In patients with FIGO stage IB2 and IIA1, what factors determine the choice between surgery and radiotherapy? What are the differences in outcomes between the two modalities?** | | | | |
| ChatGPT4.0 turbo (With prompt) | 88. **Choice Between Surgery and Radiotherapy for FIGO Stage IB2 and IIA1**: The choice between surgery and radiotherapy in patients with FIGO stage IB2 and IIA1 cervical cancer depends on several factors including the size of the tumor, patient's overall health, fertility desires, and potential side effects of each treatment modality. Surgery (radical hysterectomy with lymphadenectomy) is often preferred for younger patients who may better tolerate surgery and for those desiring to preserve ovarian function, whereas radiotherapy might be chosen for women with larger tumors (>4 cm), poor surgical candidates, or when there is a higher risk of surgical complications. Radiotherapy may be combined with chemotherapy to enhance efficacy (chemoradiation). Outcomes between the two modalities are generally comparable in terms of survival, but they differ in terms of side effects, recovery time, and impact on fertility. | B | B | B |
| ChatGPT4.0 turbo | 88. Factors Determining Choice Between Surgery and Radiotherapy for FIGO Stage IB2 and IIA1 Cervical Cancer The choice between surgery and radiotherapy in patients with FIGO stage IB2 and IIA1 cervical cancer depends on several factors:  Tumor Size and Location: Larger tumors (greater than 4 cm in IB2) or those close to the pelvic wall may be less amenable to surgery alone.  Patient’s Reproductive Desires: Surgery might be preferred if fertility preservation is a priority.  Patient’s General Health and Surgical Risk: Comorbidities that make surgery highrisk might tilt the balance towards radiotherapy.  Availability of Expertise and Resources: Surgical outcomes are better at highvolume centers. Differences in Outcomes:   Surgery can offer excellent local control and the possibility of fertility preservation but comes with surgical risks and potential for significant morbidity.  Radiotherapy, possibly combined with chemotherapy, offers good local control and is less dependent on the patient's surgical risk profile but carries a risk of longterm side effects such as bowel, bladder, and sexual dysfunction. | B | B | B |
| Claude 2 (With prompt) | 88. For stage IB2-IIA1 cervical cancer, surgery and radiotherapy have comparable outcomes. Factors guiding modality choice are primary tumor size, parametrial involvement, lymph node status, desire for ovarian preservation, and surgical candidacy. Radiotherapy may have lower complication rates. | B | B | B |
| Claude 2 | 88. Important factors determining choice between surgery and radiotherapy for FIGO IB2 and IIA1 cervical cancer include desire for future fertility, tumor size, lymphovascular space invasion, and lymph node status. Overall survival is comparable. Surgery allows preservation of ovarian function in premenopausal women. Radiotherapy is associated with higher morbidity and complications like vaginal stenosis. | B | B | B |
| Gemini Pro (With prompt) | 88. In patients with FIGO stage IB2 and IIA1, what factors determine the choice between surgery and radiotherapy? What are the differences in outcomes between the two modalities?   Factors determining the choice of treatment:  Patient age and fertility goals  Tumor size and location  Presence of lymphovascular space invasion (LVSI)  Patient's overall health and comorbidities  Differences in outcomes:  Surgery: Higher rates of local control and overall survival, but may affect fertility.  Radiotherapy: Lower rates of local control and overall survival, but preserves fertility. | B | B | B |
| Gemini Pro | 88. **In patients with FIGO stage IB2 and IIA1, what factors determine the choice between surgery and radiotherapy? What are the differences in outcomes between the two modalities?** The factors that determine the choice between surgery and radiotherapy for patients with FIGO stage IB2 and IIA1 cervical cancer include: * **Patient age:** Younger patients are more likely to be treated with surgery, while older patients are more likely to be treated with radiotherapy. * **Patient fertility:** Patients who wish to preserve their fertility are more likely to be treated with surgery. * **Tumor size:** Smaller tumors are more likely to be treated with surgery, while larger tumors are more likely to be treated with radiotherapy. * **Lymph node involvement:** Patients with lymph node involvement are more likely to be treated with radiotherapy. The outcomes of surgery and radiotherapy for FIGO stage IB2 and IIA1 cervical cancer are similar. However, surgery is associated with a higher risk of complications, such as bleeding, infection, and damage to the bladder or rectum. Radiotherapy is associated with a higher risk of long-term side effects, such as infertility, sexual dysfunction, and bowel problems. | B | B | B |
| Mistral-7B-v0.2 (With prompt) | 88.In patients with FIGO stage IB2 and IIA1 cervical cancer, several factors determine the choice between surgery and radiotherapy. These factors include the patient's age, desire for fertility preservation, tumor size and location, and the presence of lymphovascular space invasion (LVSI) or positive pelvic lymph nodes. Surgery, such as radical hysterectomy, is typically recommended for younger patients who desire fertility preservation and have small, localized tumors without LVSI or positive lymph nodes. Radiotherapy, on the other hand, is recommended for older patients, those with larger tumors, or those with positive lymph nodes. The outcomes of surgery and radiotherapy differ in terms of survival rates, side effects, and fertility preservation. Surgery offers a higher chance of cure, but it may result in more significant side effects such as urinary and sexual dysfunction. Radiotherapy, while less invasive, may result in long-term side effects such as bowel and bladder dysfunction, and it may not offer the same chance of fertility preservation. | B | B | B |
| Mistral-7B-v0.2 | 88. Factors determining the choice between surgery and radiotherapy for patients with FIGO stage IB2 and IIA1 cervical cancer: Patient age, comorbidities, and personal preferences: Patients with age, underlying health conditions, or preferences that make surgery a more challenging option may be better suited for radiotherapy. Tumor location, size, and histology: Tumors that are difficult to remove surgically, have a large size, or present with aggressive histological features are often better managed with radiotherapy. Availability of surgical specialists and resources: The availability of surgical facilities, personnel, and surgeons with expertise in performing complex cervical cancer surgeries plays a significant role in the choice between surgery and radiotherapy. Fertility goals: Women seeking to preserve their fertility are generally recommended radiotherapy, while those who prioritize cure are better candidates for surgery. Location of a cancer center and travel considerations: For patients living far from specialized cancer centers, choosing radiotherapy over surgery may be a more practical option, as it typically requires fewer visits and less time away from home. | B | B | B |
| Starling-LM-7B alpha (With prompt) | 88. In patients with FIGO stage IB2 and IIA1, the choice between surgery and radiotherapy is determined by several factors, including patient age, performance status, tumor size, and histology. Surgery, such as radical hysterectomy or radical trachelectomy, is often considered for younger patients with good performance status and small tumors. Radiotherapy, including external beam radiation therapy (EBRT) and brachytherapy, is often considered for older patients, those with poor performance status, or larger tumors. The outcomes between the two modalities vary, with surgery often resulting in better preservation of quality of life and fertility, while radiotherapy may be more effective in controlling local disease. | B | B | B |
| Starling-LM-7B alpha | 88. In patients with FIGO stage IB2 and IIA1 cervical cancer, the choice between surgery and radiotherapy is determined by several factors, including patient age, performance status, tumor size, histology, and patient preferences. Surgery, such as radical hysterectomy, is often considered for younger patients with good performance status and small tumors, while radiotherapy, including external beam radiation therapy (EBRT) and brachytherapy, is often preferred for older patients, those with poor performance status, or large tumors. The choice of treatment modality may also be influenced by the availability of experienced surgeons and radiation oncologists, as well as the presence of comorbidities. | B | B | B |
| HuatuoGPT (With prompt) | 88.What factors determine the choice between surgery and radiotherapy in patients with FIGO stages IB2 and IIA1? How do outcomes differ between the two modalities? In patients with FIGO stages IB2 and IIA1, the choice between surgery or radiation therapy depends on a variety of factors, including the patient's age, overall health, tumor size, location, and histopathology. Surgery involves removing the entire cervix and the tissues surrounding the cervix, while radiation therapy involves the use of high-energy rays to kill cancer cells. Results may vary between the two modalities, depending on the stage of the disease and the individual patient. In general, radiotherapy is usually used for more advanced stages, while surgery is more suitable for earlier stages. | B | B | B |
| HuatuoGPT | 88. In patients with FIGO-staged IB2 and IIA1 cervical cancer, the choice of surgery or radiotherapy depends on a number of factors, including the size of the tumor, lymph node metastasis, the histological type of the tumor, and the patient's fertility wishes. For early-stage lesions, radiotherapy is usually the treatment of choice, preserving uterine and ovarian function. For advanced lesions, on the other hand, surgery may be a more appropriate choice, as it can provide more complete tumor clearance. The efficacy of the two treatments varies in specific patients and decisions need to be made on an individual basis. | C | C | C |
| BioMedLM 2.7B (With prompt) | 88. In patients with FIGO stage IB2 and IIA1, what factors determine the choice between surgery and radiotherapy? What are the differences in outcomes between the two modalities? The choice of treatment modality in patients with FIGO stage IB2 and IIA1 cervical cancer is based on the patient\'s age, medical comorbidities, and the extent of disease. The National Comprehensive Cancer Network (NCCN) guidelines recommend radical hysterectomy with pelvic lymphadenectomy for patients with stage IB2 disease and chemoradiation for patients with stage IIA1 disease.^[@B1]^ The Gynecologic Oncology Group (GOG) protocol 249 randomized patients with stage IB2 and IIA1 cervical cancer to either radical hysterectomy with pelvic lymphadenectomy or chemoradiation.^[@B2]^ The results of this study showed that chemoradiation was associated with a higher rate of disease-free survival (DFS) and overall survival (OS) compared with radical hysterectomy.^[@B2]^ | D | D | D |
| BioMedLM 2.7B | 88. In patients with FIGO stage IB2 and IIA1, what factors determine the choice between surgery and radiotherapy? What are the differences in outcomes between the two modalities? ### Response: FIGO stage IB2 and IIA1 are defined as tumors that invade the cervical stroma to a depth of 5 mm or less and 6 mm or less, respectively. The depth of invasion is measured from the basement membrane of the cervical epithelium to the deepest point of invasion. The depth of invasion is a major determinant of the choice of treatment. The depth of invasion is a major determinant of the choice of treatment. | D | D | D |
| Microsoft Phi2 (With prompt) | 88. The choice between surgery and radiotherapy in patients with FIGO stage IB2 and IIA1 is based on factors such as the patient's age, overall health, and the extent of the tumor. In some cases, surgery may be preferred if the tumor is small and localized, while radiotherapy may be recommended if the tumor is larger or if the patient is not a suitable candidate for surgery. The outcomes of surgery and radiotherapy are generally similar in terms of local control and overall survival, but surgery may have a higher risk of complications such as infection and bleeding. | B | B | B |
| Microsoft Phi2 | 88. The main factors that determine the choice between surgery and radiotherapy in patients with FIGO stage IB2 and IIA1 cervical cancer are the patient's age, overall health, and the extent of the tumor. In general, surgery is preferred for younger patients with smaller tumors, while radiotherapy is preferred for older patients or those with larger tumors. The differences in outcomes between surgery and radiotherapy for FIGO stage IB2 and IIA1 cervical cancer are not well established. However, some studies have shown that surgery may be associated with a higher risk of complications and a longer recovery time, while radiotherapy may be associated with a higher risk of long-term side effects such as infertility and urinary incontinence. | B | B | B |
| QiZhenGPT (With prompt) | 88. In patients with FIGO stage IB2 and IIA1, what factors determine the choice between surgery and radiotherapy? What are the differences in outcomes between the two modalities? Answer: Factors such as patient age, stage, and overall health should be considered when making a decision between surgery and radiotherapy for patients with FIGO stage IB2 and IIA1 cervical cancer. The outcomes of the two modalities can vary, with surgery generally offering better outcomes in terms of survival and quality of life, while radiotherapy can provide better control over the spread of the cancer. | C | C | C |
| QiZhenGPT | 88. Factors determining the choice between surgery and radiotherapy in patients withFIGO stage IB2 and IIA1 include the patient's general health, the presence or absence of lymph nodes, and the extent of the cancer in the uterus or cervix. The choice between the two modalities is often based on a combination of these factors, as well as the patient's preferences. The outcomes between the two modalities are generally similar, with surgery having a slightly higher five-year survival rate. | C | B | B |
| **89. What is the recommended treatment of choice for FIGO stage IB3 cervical cancer?** | | | | |
| ChatGPT4.0 turbo (With prompt) | 89. **Recommended Treatment for FIGO Stage IB3 Cervical Cancer**: For FIGO stage IB3 cervical cancer, the recommended treatment is chemoradiation. This approach combines radiotherapy with concurrent chemotherapy, typically using cisplatin as the chemotherapeutic agent. This combination has been shown to improve survival rates compared to radiotherapy alone and is effective in controlling both local and regional disease. | A | A | A |
| ChatGPT4.0 turbo | 89. Recommended Treatment for FIGO Stage IB3 Cervical Cancer For FIGO stage IB3 cervical cancer, the recommended treatment is typically chemoradiation. The combination of radiotherapy and concurrent chemotherapy is favored due to the high risk of local and distant failure associated with larger tumor burdens that define this stage. | B | B | B |
| Claude 2 (With prompt) | 89. The recommended primary treatment for stage IB3 is chemoradiation with cisplatin rather than radical hysterectomy given the bulkier disease and higher risk of positive margins with surgery alone. | A | A | A |
| Claude 2 | 89. For FIGO stage IB3 cervical cancer, the standard recommended treatment is chemoradiation (external beam radiation with concurrent cisplatin-based chemotherapy) rather than primary surgery. Primary chemoradiation results in improved overall and disease-free survival compared to surgery in this locally-advanced stage. | A | A | A |
| Gemini Pro (With prompt) | 89. What is the recommended treatment of choice for FIGO stage IB3 cervical cancer?  The recommended treatment of choice for FIGO stage IB3 cervical cancer is concurrent chemoradiation. This involves a combination of external beam radiation therapy (EBRT) and chemotherapy. | B | B | B |
| Gemini Pro | 89. **What is the recommended treatment of choice for FIGO stage IB3 cervical cancer?** The recommended treatment of choice for FIGO stage IB3 cervical cancer is radical hysterectomy with pelvic lymph node dissection. This is because FIGO stage IB3 cervical cancer is more likely to have spread to the lymph nodes than FIGO stage IB2 and IIA1 cervical cancer. | D | D | D |
| Mistral-7B-v0.2 (With prompt) | 89.The recommended treatment of choice for FIGO stage IB3 cervical cancer is radical hysterectomy with pelvic lymph node dissection. This procedure involves the removal of the uterus, cervix, and surrounding tissue, as well as the removal of pelvic lymph nodes. Radiotherapy may also be used as an alternative treatment for patients who are not candidates for surgery due to medical conditions or age. | C | C | C |
| Mistral-7B-v0.2 | 89. Recommended treatment of choice for FIGO stage IB3 cervical cancer: The preferred treatment for FIGO stage IB3 cervical cancer is often external beam radiotherapy (EBRT) combined with brachytherapy, referred to as combined modality radiotherapy (CMRT). This approach allows for a higher dose of radiation to be delivered to the tumor site with minimal damage to surrounding healthy tissue. The goal of this treatment is to achieve complete destruction of the cancerous tissue while preserving the patient's quality of life as much as possible. Surgery is generally not a suitable option for patients with FIGO stage IB3 cervical cancer due to the significant risk of pelvic organ damage and postoperative complications. | B | B | B |
| Starling-LM-7B alpha (With prompt) | 89. The recommended treatment of choice for FIGO stage IB3 cervical cancer is radiotherapy, including EBRT and brachytherapy. This approach provides effective local control and is often combined with chemotherapy to improve systemic control. Surgery, such as radical hysterectomy, may be considered in select cases, but is generally reserved for patients with specific tumor characteristics and good performance status. | B | B | B |
| Starling-LM-7B alpha | 89. The recommended treatment of choice for FIGO stage IB3 cervical cancer is a combination of surgery and radiotherapy. This may include radical hysterectomy followed by EBRT and brachytherapy, or definitive radiotherapy with EBRT and brachytherapy for patients who are not candidates for surgery. | C | C | C |
| HuatuoGPT (With prompt) | 89. What is the treatment of choice for FIGO stage IB3 cervical cancer? The treatment of choice for FIGO stage IB3 cervical cancer is radical hysterectomy, also known as radical hysterectomy. This is a surgical procedure that involves removal of the uterus, cervix, part of the vagina and lymph nodes. It is sometimes accompanied by removal of the ovaries and fallopian tubes. | D | D | D |
| HuatuoGPT | 89. For FIGO stage IB3 cervical cancer, the recommended treatment of choice is external irradiation combined with chemotherapy, i.e. external irradiation radiotherapy combined with chemotherapy. This combination therapy can significantly improve treatment efficacy and reduce the risk of recurrence. In addition, factors such as the patient's age, overall health status, and the patient's acceptance of different treatments need to be considered. | B | A | A |
| BioMedLM 2.7B (With prompt) | 89. What is the recommended treatment of choice for FIGO stage IB3 cervical cancer? The recommended treatment of choice for FIGO stage IB3 cervical cancer is radical hysterectomy with pelvic lymphadenectomy. | D | D | D |
| BioMedLM 2.7B | 89. What is the recommended treatment of choice for FIGO stage IB3 cervical cancer? ### Response: The recommended treatment of choice for FIGO stage IB3 cervical cancer is concurrent chemoradiation therapy (CCRT) with cisplatin. | A | A | A |
| Microsoft Phi2 (With prompt) | 89. The recommended treatment of choice for FIGO stage IB3 cervical cancer is a combination of surgery and radiotherapy. | C | C | C |
| Microsoft Phi2 | 89. What is the recommended treatment of choice for FIGO stage IB3 cervical cancer? Response: The recommended treatment of choice for FIGO stage IB3 cervical cancer is radical hysterectomy with or without pelvic lymphadenectomy. Radical hysterectomy is the surgical removal of the uterus, cervix, and surrounding tissues, while pelvic lymphadenectomy is the removal of the lymph nodes in the pelvis. This treatment aims to achieve complete removal of the tumor and prevent the spread of cancer to other organs. Radical hysterectomy with pelvic lymphadenectomy has a high rate of local control (95%) and survival (90%) compared to other treatments, such as radiotherapy or chemotherapy. | D | D | D |
| QiZhenGPT (With prompt) | 89. What is the recommended treatment of choice for FIGO stage IB3 cervical cancer? Answer: The recommended treatment of choice for FIGO stage IB3 cervical cancer is surgery. Radiotherapy may also be considered in some cases, particularly if the patient has a high risk of recurrence. | C | C | C |
| QiZhenGPT | 89. The recommended treatment of choice for Figure stage IB3 cervical cancer is a combination of surgery and radiotherapy. This approach allows for a more comprehensive treatment of the cancer and can improve outcomes. | C | C | C |
| **90. what is the difference between the different types of radical hysterectomy? For which patients is it indicated?** | | | | |
| ChatGPT4.0 turbo (With prompt) | 90. **Types of Radical Hysterectomy and Indications**: There are different types of radical hysterectomy, classified based on the extent of tissue removal and dissection: Type A (modified radical), Type B (standard radical), and Type C (extended radical). Type B radical hysterectomy is most commonly performed for early-stage cervical cancer (FIGO IA2 to IB1) and involves the removal of the cervix, part of the vagina, and a wide margin of surrounding tissue, along with pelvic lymph nodes. Type C, which includes more extensive dissection possibly involving parts of the bladder, rectum, or exenteration, is indicated for larger or more invasive tumors. Type A may be considered when less extensive dissection is required, typically for smaller tumors with minimal invasion. | B | B | B |
| ChatGPT4.0 turbo | 90. Types of Radical Hysterectomy and Indications Radical hysterectomy involves the removal of the uterus, cervix, part of the vagina, and surrounding tissues, which are categorized into different types based on the extent of tissue removal:  Type A (Modified Radical Hysterectomy): Less extensive, suitable for earlystage cancers where the risk of spread is lower.  Type B (Standard Radical Hysterectomy): Involves more extensive tissue removal, suitable for most cases of stage IA2 to IB1 cervical cancer.  Type C (Extended Radical Hysterectomy): Includes more extensive removal of surrounding tissues and is indicated for larger or more invasive tumors. Radical hysterectomy is typically indicated for patients who have earlystage disease (IA2 to IIA) where the likelihood of metastatic spread is minimal and the tumor size permits complete surgical resection. | C | B | C |
| Claude 2 (With prompt) | 90. The types of radical hysterectomy differ by degree of parametrial and paravaginal tissue resection. Type C has the most extensive resection and is indicated for larger IB2-IIA tumors with suspected parametrial involvement. | B | B | B |
| Claude 2 | 90. The types of radical hysterectomy are:  - Type A - removes uterus + parametria + upper vagina - Type B - removes uterus + parametria + full vagina - Type C - most extensive; removes uterus, parametria, upper vagina + pelvic lymph nodes  Radical hysterectomy is indicated for early stage cervical cancer where fertility preservation is not desired. Type C is preferred for tumors >2cm. | C | C | C |
| Gemini Pro (With prompt) | 90. What is the difference between the different types of radical hysterectomy? For which patients is it indicated?   Types of radical hysterectomy:  Type I: Removal of the uterus, cervix, parametria, and upper vagina.  Type II: Removal of the uterus, cervix, parametria, upper vagina, and pelvic lymph nodes.  Type III: Removal of the uterus, cervix, parametria, upper vagina, pelvic lymph nodes, and a portion of the bladder or rectum.  Indications: Radical hysterectomy is indicated for patients with FIGO stage IB1-IIA2 cervical cancer who are not candidates for radiotherapy or who have recurrent disease after radiotherapy. | B | B | B |
| Gemini Pro | 90. **What is the difference between the different types of radical hysterectomy? For which patients is it indicated?** There are three main types of radical hysterectomy: * **Type I radical hysterectomy:** This type of radical hysterectomy removes the uterus, cervix, and upper vagina. It is indicated for patients with FIGO stage IB2 and IIA1 cervical cancer. * **Type II radical hysterectomy:** This type of radical hysterectomy removes the uterus, cervix, upper vagina, and parametria. It is indicated for patients with FIGO stage IB3 cervical cancer. * **Type III radical hysterectomy:** This type of radical hysterectomy removes the uterus, cervix, upper vagina, parametria, and pelvic lymph nodes. It is indicated for patients with FIGO stage IIIB and IVA cervical cancer. | B | B | B |
| Mistral-7B-v0.2 (With prompt) | 90.There are several types of radical hysterectomy, including simple, modified, and extended radical hysterectomy. The choice of procedure depends on the size and location of the tumor, as well as the presence of LVSI or positive pelvic lymph nodes. Simple radical hysterectomy involves the removal of the uterus and cervix, as well as the parametria and the upper third of the vagina. Modified radical hysterectomy involves the removal of the uterus, cervix, parametria, and the lower third of the vagina. Extended radical hysterectomy involves the removal of the uterus, cervix, parametria, and the pelvic sidewall. Radical hysterectomy is indicated for patients with early-stage cervical cancer who desire fertility preservation and have small, localized tumors without LVSI or positive lymph nodes. | C | C | C |
| Mistral-7B-v0.2 | 90. Differences between types of radical hysterectomy: Total Abdominal Hysterectomy (TAH): In this procedure, the entire uterus, cervix, and surrounding lymph nodes are removed through a large abdominal incision. Laparoscopic Radical Hysterectomy (LRH): The procedure is similar to TAH but performed through multiple small incisions using a laparoscope and laparoscopic instruments. LRH offers several advantages, including smaller incisions, faster recovery, and less postoperative pain. However, not all patients are suitable candidates, as experience and expertise with the procedure are key factors. Robotically-Assisted Radical Hysterectomy (RARH): Similar to LRH, RARH uses a robot to perform the surgery through multiple small incisions. This approach offers the advantage of 3D visualization and precise movements, making it an alternative to traditional LRH for surgeons less experienced with the technique. | D | D | D |
| Starling-LM-7B alpha (With prompt) | 90. The different types of radical hysterectomy include simple, modified, and total radical hysterectomy. These procedures vary in the extent of parametrial and lymph node dissection. Radical hysterectomy is indicated for patients with early-stage cervical cancer, who are candidates for surgery due to factors such as age, performance status, and tumor characteristics. | C | C | C |
| Starling-LM-7B alpha | 90. There are several types of radical hysterectomy, including simple, modified, and total radical hysterectomy. The choice of procedure depends on the extent of disease, patient anatomy, and surgeon experience. Simple radical hysterectomy involves the removal of the uterus and upper vagina, while modified radical hysterectomy includes the removal of the parametria and total radical hysterectomy includes the removal of the parametria and lower vagina. Radical hysterectomy is indicated for patients with early-stage cervical cancer who are candidates for surgery and have no contraindications. | C | C | C |
| HuatuoGPT (With prompt) | 90. What is the difference between the different types of radical hysterectomy? For which patients are they indicated? There are several types of radical hysterectomy including pelvic, radical and modified radical. All of these procedures involve removal of the uterus, cervix, part of the vagina, and lymph nodes. However, each procedure can vary in the extent and number of removals. Pelvic hysterectomy removes only the uterus and cervix, while radical hysterectomy removes the uterus, cervix, part of the vagina and lymph nodes. Modified radical hysterectomy is a variant of pelvic hysterectomy and usually involves removal of the parauterine lymph nodes. | C | C | C |
| HuatuoGPT | 90. Different types of radical hysterectomy have different indications. For FIGO stage IA1 squamous cervical cancer, conization of the cervix is a better choice; for cervical adenocarcinoma of FIGO stage IB1 or above, hysterectomy may be more appropriate; and more extensive surgery such as total hysterectomy or hysterectomy with lymph node dissection may be required for cervical cancers of stage II or higher stages. | C | C | C |
| BioMedLM 2.7B (With prompt) | 90. what is the difference between the different types of radical hysterectomy? For which patients is it indicated? The difference between the different types of radical hysterectomy is that the type II radical hysterectomy is performed for patients with stage IB1 cervical cancer, and the type III radical hysterectomy is performed for patients with stage IB2 cervical cancer | C | C | C |
| BioMedLM 2.7B | 90. what is the difference between the different types of radical hysterectomy? For which patients is it indicated? ### Response: The difference between the different types of radical hysterectomy is that the abdominal approach is used for patients with stage IB1 disease, and the laparoscopic approach is used for patients with stage IB2 disease. | D | D | D |
| Microsoft Phi2 (With prompt) | 90. There are several types of radical hysterectomy, including total hysterectomy, subtotal hysterectomy, and radical hysterectomy with lymphadenectomy. The choice of procedure depends on the extent of the tumor and the patient's overall health. Total hysterectomy involves the removal of the entire uterus, while subtotal hysterectomy involves the removal of part of the uterus. Radical hysterectomy with lymphadenectomy involves the removal of the uterus, cervix, and nearby lymph nodes. This procedure is indicated for patients with a large tumor that has spread to nearby lymph nodes. | C | C | C |
| Microsoft Phi2 | 90. The different types of radical hysterectomy include total hysterectomy, subtotal hysterectomy, and radical hysterectomy with pelvic lymphadenectomy. Total hysterectomy involves the removal of the entire uterus, while subtotal hysterectomy involves the removal of the cervix and part of the uterus. Radical hysterectomy with pelvic lymphadenectomy involves the removal of the uterus, cervix, and surrounding lymph nodes. Radical hysterectomy is indicated for patients with larger tumors or those who are not suitable candidates for radiotherapy. | C | D | C |
| QiZhenGPT (With prompt) | 90. what is the difference between the different types of radical hysterectomy? For which patients is it indicated? Answer: radical hysterectomy refers to a surgical procedure that involves the removal of all the tissue surrounding a specific area of the body, such as the uterus, cervix, and vagina. There are several different types of radical hysterectomy, including total hysterectomy, partial hysterectomy, and vaginal hysterectomy. These procedures are typically indicated for patients with advanced stages of cervical cancer who are not candidates for surgery due to their poor health status. | B | B | B |
| QiZhenGPT | 90. There are three main types of radical hysterectomy: total hysterectomy, subtotal hysterectomy, and radical hysterectomy with lymph node dissection. The choice of which type of radical hysterectomy to perform depends on the patient's specific cancer and the preferences of the surgical team. For example, total hysterectomy may be indicated for patients with large cancer or deep infiltration into the uterus, while subtotal hysterectomy may be indicated for patients with smaller cancer or less infiltration. Radical hysterectomy with lymph node dissection may be indicated for patients with cancer that has spread to the lymph nodes. | B | B | B |
| **91. What are the advantages of IMRT in radiotherapy for cervical cancer?** | | | | |
| ChatGPT4.0 turbo (With prompt) | 91. **Advantages of IMRT in Radiotherapy for Cervical Cancer**: Intensity-Modulated Radiation Therapy (IMRT) offers several advantages in the treatment of cervical cancer. IMRT allows for the radiation dose to be precisely shaped to conform to the geometry of the tumor, minimizing exposure and damage to surrounding healthy tissues and organs. This precision reduces the risk of side effects commonly associated with radiation therapy, such as gastrointestinal and urinary toxicity. Additionally, IMRT can facilitate the delivery of higher radiation doses directly to the tumor or areas at high risk for disease spread, potentially improving treatment efficacy. | A | A | A |
| ChatGPT4.0 turbo | 91. Advantages of IMRT in Radiotherapy for Cervical Cancer  IntensityModulated Radiation Therapy (IMRT) offers several advantages in the treatment of cervical cancer:  Targeted Delivery: IMRT allows for the radiation to be more precisely targeted to the shape of the tumor, sparing surrounding healthy tissue from high doses of radiation.  Reduced Side Effects: By minimizing exposure to healthy tissues, IMRT reduces the risk of side effects associated with radiation therapy, such as gastrointestinal, urological, and sexual dysfunction.  Adaptability: IMRT can be adapted if the tumor changes in size or shape during treatment, allowing for ongoing customization of the radiation dose. | A | B | A |
| Claude 2 (With prompt) | 91. Advantages of IMRT include highly conformal dose distribution allowing dose escalation to target tissues while relatively sparing surrounding organs like bladder and rectum to reduce toxicity. | A | A | A |
| Claude 2 | 91. Advantages of IMRT for cervical cancer radiotherapy include: improved dose conformity to target volumes, decreased dose to bowel/bladder/rectum, reduced acute/late toxicity. This allows dose escalation with potentially better tumor control. | A | A | A |
| Gemini Pro (With prompt) | 91. What are the advantages of IMRT in radiotherapy for cervical cancer?   Advantages of IMRT:  More conformal treatment, reducing the dose to surrounding healthy tissues  Improved local control and overall survival rates  Reduced risk of long-term complications, such as bowel and bladder dysfunction | A | A | A |
| Gemini Pro | 91. **What are the advantages of IMRT in radiotherapy for cervical cancer?** IMRT (intensity-modulated radiotherapy) is a type of radiotherapy that uses computer-controlled technology to deliver precise doses of radiation to the tumor while minimizing the dose to surrounding healthy tissues. The advantages of IMRT in radiotherapy for cervical cancer include: * **Reduced toxicity:** IMRT can reduce the risk of side effects, such as radiation cystitis, proctitis, and vaginal stenosis. * **Improved local control:** IMRT can improve the local control of cervical cancer, which can lead to improved survival rates. | A | A | A |
| Mistral-7B-v0.2 (With prompt) | 91.Intensity-modulated radiation therapy (IMRT) is a type of radiotherapy that offers several advantages over conventional radiotherapy for cervical cancer. IMRT allows for more precise targeting of the tumor, minimizing exposure to healthy tissue and reducing side effects. IMRT also allows for the delivery of higher doses of radiation to the tumor while sparing the surrounding tissue, which may improve outcomes and reduce the risk of recurrence. | A | A | A |
| Mistral-7B-v0.2 | 91. Advantages of Intensity-Modulated Radiation Therapy (IMRT) in radiotherapy for cervical cancer: Conformity to target: IMRT can deliver a high dose of radiation to the tumor while minimizing radiation exposure to adjacent organs, such as the bladder, rectum, and small intestines, which can help reduce side effects. More precise treatment planning: IMRT allows for dose modifications based on an individual patient's anatomy and tumor size, offering personalized cancer treatment. Potential for reduced toxicity and side effects: By tailoring the radiation dose to the individual tumor and surrounding tissues, IMRT has shown the potential for reduced toxicity and fewer side effects compared to 3D-conformal radiotherapy. | B | A | A |
| Starling-LM-7B alpha (With prompt) | 91. Intensity-modulated radiation therapy (IMRT) is an advanced form of radiotherapy that allows for more precise targeting of the tumor while minimizing radiation exposure to surrounding healthy tissues. In cervical cancer, IMRT can reduce the risk of side effects such as bowel, bladder, and sexual dysfunction. Additionally, IMRT may improve local control and overall survival compared to conventional radiotherapy techniques. | A | A | A |
| Starling-LM-7B alpha | 91. Intensity-modulated radiation therapy (IMRT) is an advanced form of radiotherapy that uses computer-generated images to deliver precise doses of radiation to the tumor while minimizing exposure to healthy tissues. The advantages of IMRT in radiotherapy for cervical cancer include improved target coverage, reduced dose to adjacent organs at risk (such as the bladder, rectum, and bowel), and reduced treatment-related side effects. These advantages may lead to improved quality of life and long-term outcomes for patients with cervical cancer. | A | A | A |
| HuatuoGPT (With prompt) | 91. What are the advantages of radiation therapy for cervical cancer? Radiation therapy is one of the treatment options for cervical cancer. Its advantages include: * using high-energy rays to kill cancer cells *Avoiding surgery, which is a convenient option for some patients *Preserves the uterus and reproductive function *can deliver high doses of radiation to a localized area while minimizing radiation to surrounding tissues | A | A | A |
| HuatuoGPT | 91. Intensity-modulated radiotherapy (IMRT) has many advantages in radiotherapy for cervical cancer. Compared with conventional radiotherapy techniques, IMRT can irradiate the tumor area more precisely and reduce damage to healthy tissues, thus reducing the risk of complications.IMRT can also better protect vital organs and tissues, such as the ovaries, rectum, and bladder, by reducing the dose of radiation to which they are exposed. Therefore, IMRT can improve treatment efficacy and reduce the incidence of side effects. | A | A | A |
| BioMedLM 2.7B (With prompt) | 91. What are the advantages of IMRT in radiotherapy for cervical cancer? IMRT has been shown to improve the therapeutic ratio in the treatment of cervical cancer. The use of IMRT has been shown to reduce the dose to the bladder, rectum, and small bowel, and to improve the therapeutic ratio. | A | A | A |
| BioMedLM 2.7B | 91. What are the advantages of IMRT in radiotherapy for cervical cancer? ### Response: IMRT is a technique that allows the delivery of a highly conformal dose to the target volume while sparing the surrounding normal tissues. The advantages of IMRT include the ability to deliver a highly conformal dose to the target volume while sparing the surrounding normal tissues. The advantages of IMRT include the ability to deliver a highly conformal dose to the target volume while sparing the surrounding normal tissues. IMRT is a technique that allows the delivery of a highly conformal dose to the target volume while sparing the surrounding normal tissues. | A | A | A |
| Microsoft Phi2 (With prompt) | 91. IMRT offers several advantages over conventional radiotherapy for cervical cancer. It allows for more precise targeting of the tumor, which can help to minimize damage to surrounding healthy tissue. This can lead to fewer side effects and a better quality of life for the patient. IMRT also allows for the delivery of higher doses of radiation to the tumor, which can improve the chances of achieving local control and preventing recurrence. | A | A | A |
| Microsoft Phi2 | 91. What are the advantages of IMRT in radiotherapy for cervical cancer? Response: IMRT is an advanced technique of radiotherapy that delivers high doses of radiation to the tumor while sparing the surrounding normal tissues. The advantages of IMRT in radiotherapy for cervical cancer are: - It reduces the risk of side effects, such as radiation necrosis, fibrosis, and infertility, by delivering the radiation in a more precise and conformal way. - It improves the quality of life of the patients by minimizing the impact of radiation on the urinary, bowel, and sexual functions. - It increases the survival rate of the patients by enhancing the local control of the tumor and preventing the recurrence of cancer. | B | A | A |
| QiZhenGPT (With prompt) | 91. What are the advantages of IMRT in radiotherapy for cervical cancer? Answer: IMRT is a type of radiation therapy that uses high-energy photons to deliver radiation to the target area. It offers several advantages over traditional radiotherapy, including improved image guidance, reduced side effects, and improved patient comfort. IMRT can be used to treat early-stage cervical cancer and can be effective in reducing the risk of recurrence and improving the overall survival rate. | A | A | A |
| QiZhenGPT | 91. IMRT (Intensity Modulated Radiation Therapy) in radiotherapy for cervical cancer has several advantages, including the ability to deliver a more precise and personalized treatment, the potential for improved outcome, and the ability to reduce side effects. | A | B | B |
| **92. What are the common sites of recurrence in cervical cancer? How is the risk of recurrence monitored?** | | | | |
| ChatGPT4.0 turbo (With prompt) | 92. **Common Sites of Recurrence in Cervical Cancer and Monitoring**: The most common sites of recurrence in cervical cancer include the pelvic region, lymph nodes, and distant sites such as the lungs and liver. Monitoring for recurrence typically involves regular follow-up visits with physical examinations, Pap tests, and imaging studies such as CT scans or MRIs, depending on individual risk factors and initial tumor characteristics. Surveillance strategies are often tailored based on the stage at diagnosis, treatments received, and overall health of the patient. Monitoring is crucial for early detection of recurrence, which can significantly influence the prognosis and options for subsequent treatment. | A | A | A |
| ChatGPT4.0 turbo | 92. Common Sites of Recurrence in Cervical Cancer and Monitoring Risk Common Sites of Recurrence:  Local Recurrence: In the pelvic region including the vaginal cuff, parametrium, and lymph nodes.  Distant Recurrence: Commonly involves the lungs, liver, and bones. Monitoring Risk:  Followup Visits: Regular followup visits including physical exams and Pap tests.  Imaging: Periodic imaging studies such as CT, MRI, or PET scans may be used depending on individual risk factors and symptoms.  Biomarkers: Research into tumor markers is ongoing to help predict and monitor recurrence, though none are currently in standard use for cervical cancer outside of clinical trials. Monitoring strategies are typically tailored based on the initial stage of the cancer, the treatments used, and the overall health of the patient. | A | A | A |
| Claude 2 (With prompt) | 92. Most cervical cancer recurrences occur at local/regional sites first before developing distant metastases. Surveillance includes history and exam every 3-6 months to monitor for symptoms. Imaging as indicated for concerning findings. | A | A | A |
| Claude 2 | 92. Common sites of cervical cancer recurrence include: pelvis, para-aortic nodes, lungs, extrapelvic nodes, bone, and liver. Risk of recurrence is monitored via patient symptoms, physical exam, and imaging like PET/CT. | B | B | B |
| Gemini Pro (With prompt) | 92. What are the common sites of recurrence in cervical cancer? How is the risk of recurrence monitored?   Common sites of recurrence:  Pelvis  Distant metastases (e.g., lungs, liver, bone)  Monitoring the risk of recurrence:  Regular pelvic exams and Pap tests  Imaging studies, such as CT scans or MRI scans | A | B | A |
| Gemini Pro | 92. **What are the common sites of recurrence in cervical cancer? How is the risk of recurrence monitored?** The common sites of recurrence in cervical cancer are: * **Pelvis:** The pelvis is the most common site of recurrence in cervical cancer. * **Abdomen:** The abdomen is the second most common site of recurrence in cervical cancer. * **Lungs:** The lungs are the third most common site of recurrence in cervical cancer. The risk of recurrence in cervical cancer is monitored by: * **Physical examination:** The doctor will perform a physical examination to check for any signs of recurrence. * **Imaging tests:** Imaging tests, such as CT scans and MRI scans, can be used to detect recurrence. * **Blood tests:** Blood tests can be used to check for tumor markers, which are substances that are released into the blood by cancer cells. The frequency of monitoring will depend on the stage of the cancer and the patient's individual risk of recurrence. | A | B | A |
| Mistral-7B-v0.2 (With prompt) | 92.The common sites of recurrence in cervical cancer include the pelvis, para-aortic lymph nodes, and the vagina. The risk of recurrence is monitored through regular follow-up appointments, including pelvic examinations, Pap tests, and imaging studies such as CT scans or MRI scans. Patients may also undergo HPV testing and cervical cancer screening tests to detect recurrence early and improve outcomes. | A | B | A |
| Mistral-7B-v0.2 | 92. Common sites of recurrence in cervical cancer and monitoring risk: Lymph node metastases: Recurrence in the pelvic or para-aortic lymph nodes is a common pattern of recurrence, particularly for patients with FIGO stage IB and IIA cervical cancers. Local recurrences: Local progression of disease occurs in approximately 10% of cases and usually presents within 2-3 years post-treatment. Risk factors include positive surgical or lymph node margins and high-risk tumor characteristics (such as advanced age, large primary tumors, lymphovascular space invasion, or poor differentiation). Distant recurrence: Though less common, distant recurrences can occur in lungs, liver, bone, or brain. Risk factors include advanced stage at diagnosis, large primary tumors, and lymph node metastasis. Regular follow-up: Close monitoring using physical examinations, cervical cancer screenings (Pap tests), and imaging procedures (such as CT scans or MRI) is critical for detecting early signs of recurrence and implementing effective treatment strategies as soon as possible. The American Society of Clinical Oncology (ASCO) recommends follow-up appointments every 3-6 months for 3-5 years, then annually for longer periods, depending on the stage and risk factors. | A | A | A |
| Starling-LM-7B alpha (With prompt) | 92. Common sites of recurrence in cervical cancer include the pelvis, para-aortic lymph nodes, and the vagina. The risk of recurrence is monitored through regular follow-up appointments, which may include physical examinations, imaging studies (such as pelvic and abdominal CT scans), and laboratory tests (such as tumor markers). Patients should be educated about the signs and symptoms of recurrence and encouraged to report any new or worsening symptoms promptly. | A | B | A |
| Starling-LM-7B alpha | 92. Common sites of recurrence in cervical cancer include the pelvis, para-aortic lymph nodes, and distant organs such as the lungs, liver, and bones. The risk of recurrence is monitored through regular follow-up appointments, including physical examinations, imaging studies (such as CT scans and PET scans), and tumor markers (such as CA-125). Early detection of recurrence is crucial for initiating appropriate salvage therapy and improving patient outcomes. | B | B | B |
| HuatuoGPT (With prompt) | 92. What are the common sites of recurrence for cervical cancer? How is the risk of recurrence monitored? Common sites of recurrence for cervical cancer include the pelvis, lungs, bones, liver and lymph nodes. The risk of recurrence depends on the stage of the disease and the effectiveness of treatment. The risk of recurrence is usually monitored over a period of time after treatment, including regular clinical examinations, imaging tests (e.g. CT scan, MRI or PET-CT) and blood tests (e.g. tumor markers) | A | B | A |
| HuatuoGPT | 92. Common sites of recurrence of cervical cancer include the pelvic lymph nodes, tissues surrounding the cervix and proximal organs (e.g. rectum and bladder). The risk of recurrence is related to pathological grading, tumor size and lymph node metastasis. Treatment for recurrent cervical cancer should be tailored to the specific situation and may include surgery, radiotherapy, chemotherapy or combination therapy. Surveillance of recurrence risk is important to guide treatment and prognostic assessment, which can be done through regular clinical examination, imaging examination and tumor marker surveillance. | B | B | B |
| BioMedLM 2.7B (With prompt) | 92. What are the common sites of recurrence in cervical cancer? How is the risk of recurrence monitored? The most common sites of recurrence in cervical cancer are the pelvis and para-aortic lymph nodes. The risk of recurrence is highest in the first 2 years after treatment. The risk of recurrence is highest in patients with positive lymph nodes, positive surgical margins, and large tumors. | A | B | A |
| BioMedLM 2.7B | 92. What are the common sites of recurrence in cervical cancer? How is the risk of recurrence monitored? ### Response: The most common sites of recurrence are the pelvis, para-aortic lymph nodes, and supraclavicular lymph nodes. The risk of recurrence is highest in the first 2 years after treatment. The risk of recurrence is highest in patients with positive lymph nodes, positive parametria, and positive surgical margins. | C | C | C |
| Microsoft Phi2 (With prompt) | 92. The common sites of recurrence in cervical cancer include the pelvis, vagina, and bladder. The risk of recurrence is monitored through regular follow-up appointments, imaging tests, and blood tests. Patients who are at high risk of recurrence may be recommended for additional treatments such as chemotherapy or radiation therapy. | A | B | A |
| Microsoft Phi2 | 92. The common sites of recurrence in cervical cancer include the pelvis, vagina, and bladder. The risk of recurrence is monitored through regular follow-up appointments and imaging tests such as CT scans and MRI scans. | B | B | B |
| QiZhenGPT (With prompt) | 92. What are the common sites of recurrence in cervical cancer? How is the risk of recurrence monitored? Answer: Recurrence can occur at any site in the body, but the most common sites of recurrence in cervical cancer are the lungs, liver, and bones. Risk of recurrence can be monitored by regular follow-up appointments and imaging studies, such as CT or MRI scans, to detect any recurrence early and allow for appropriate treatment. | D | C | C |
| QiZhenGPT | 92. Common sites of recurrence in cervical cancer include the lung, bone, and soft tissue. The risk of recurrence can be monitored through imaging studies such as CT scans and PET scans. | C | C | C |
| **93. What are the recommended treatment strategies for recurrent cervical cancer? Can surgery, radiotherapy, and chemotherapy be combined?** | | | | |
| ChatGPT4.0 turbo (With prompt) | 93. **Treatment Strategies for Recurrent Cervical Cancer**: The treatment of recurrent cervical cancer depends on several factors, including the site and extent of the recurrence, previous treatment modalities, and the patient's overall health. Options may include surgery, radiotherapy, chemotherapy, or a combination of these. If the recurrence is localized and the patient has not previously received radiotherapy, pelvic exenteration may be an option. For patients who have previously had radiotherapy, systemic chemotherapy or targeted therapy may be considered. Combining these treatments is possible, particularly chemoradiation, which may be used if the recurrence is localized and the patient can tolerate it. Clinical trials may also be an option for patients with recurrent disease. | A | A | A |
| ChatGPT4.0 turbo | 93. Treatment Strategies for Recurrent Cervical Cancer Treatment for recurrent cervical cancer depends on the site of recurrence, previous treatments, and overall health of the patient. Options include:  Surgery: Pelvic exenteration may be considered for central pelvic recurrence if there is no evidence of distant metastasis.  Radiotherapy: If not previously used, radiotherapy can be effective, particularly for localized recurrences.  Chemotherapy: Systemic chemotherapy is used for metastatic or recurrent disease not amenable to surgery or radiation. Common regimens include platinumbased therapies.  Combined Modalities: Combining surgery, radiotherapy, and chemotherapy can be considered based on individual patient factors and tumor characteristics. | C | C | C |
| Claude 2 (With prompt) | 93. Treatment of recurrent cervical cancer involves chemotherapy, radiotherapy if the patient has not received prior radiation, and/or pelvic exenteration surgery in highly selected cases. Multimodality therapy is often utilized for resectable disease. | B | B | B |
| Claude 2 | 93. Treatment of recurrent cervical cancer involves chemotherapy, radiotherapy, surgery, or combinations based on prior treatment, disease-free interval, tumor size/site. Chemoradiation can be used if patients were not previously irradiated. Surgery may be an option for isolated central or nodal recurrences. | C | B | C |
| Gemini Pro (With prompt) | 93. What are the recommended treatment strategies for recurrent cervical cancer? Can surgery, radiotherapy, and chemotherapy be combined?   Treatment strategies for recurrent cervical cancer:  Surgery: May be an option for patients with localized recurrence.  Radiotherapy: May be used for patients with locally recurrent or metastatic disease.  Chemotherapy: May be used for patients with metastatic disease.  Combination therapy: Surgery, radiotherapy, and chemotherapy can be combined in some cases to improve outcomes. | B | C | B |
| Gemini Pro | 93. **What are the recommended treatment strategies for recurrent cervical cancer? Can surgery, radiotherapy, and chemotherapy be combined?** The recommended treatment strategies for recurrent cervical cancer depend on the location and extent of the recurrence. Surgery, radiotherapy, and chemotherapy can all be used to treat recurrent cervical cancer, either alone or in combination. If the recurrence is localized to the pelvis, surgery may be an option. However, surgery is often not possible if the recurrence is extensive or if the patient has had previous surgery in the pelvis.  Radiotherapy is another option for treating recurrent cervical cancer. Radiotherapy can be used to treat both local and distant recurrences. Chemotherapy is often used in combination with radiotherapy to treat recurrent cervical cancer. Chemotherapy can help to shrink the tumor and make it more responsive to radiotherapy. | C | C | C |
| Mistral-7B-v0.2 (With prompt) | 93.The recommended treatment strategies for recurrent cervical cancer depend on the location and extent of the recurrence, as well as the patient's individual circumstances. Surgery, radiotherapy, and chemotherapy can be combined in various ways to provide the best possible outcome. For example, surgery may be used to remove local recurrences, followed by radiotherapy or chemotherapy to address any remaining disease. In some cases, chemotherapy may be used to shrink a recurrence prior to surgery. The use of targeted therapies, such as immunotherapy, may also be considered in certain situations. | A | B | A |
| Mistral-7B-v0.2 | 93. Recommended treatment strategies for recurrent cervical cancer: • Localized recurrences: Localized recurrences (involving the pelvic area or vagina) may be treated with salvage radiotherapy, brachytherapy, or surgical re-excision, depending on the size and location of the recurrence and the patient's overall health condition. • Distant recurrences: Distant recurrences (involving other organs, such as the liver, lungs, brain, or bones) are generally treated with systemic targeted therapy or chemotherapy. Chemotherapy, particularly platinum-based agents, is the primary option for these cases due to its ability to penetrate the bloodstream and affect metastatic tumors throughout the body. • Combination therapy: Combining surgery, radiotherapy, and chemotherapy depending on the recurrence site and the patient's individual response and tolerance to each modality can be effective for some patients. This approach allows for multi-pronged treatment, potentially increasing the chances of disease control and survival. | A | A | A |
| Starling-LM-7B alpha (With prompt) | 93. Recommended treatment strategies for recurrent cervical cancer include a combination of surgery, radiotherapy, and chemotherapy, depending on the patient's clinical condition, prior treatment history, and the extent of the recurrence. The treatment plan should be individualized and discussed with a multidisciplinary team, including a gynecologic oncologist. | B | B | B |
| Starling-LM-7B alpha | 93. Recommended treatment strategies for recurrent cervical cancer typically involve a combination of surgery, radiotherapy, and chemotherapy. The choice of treatment depends on the patient's overall health, the extent of the disease, and the patient's preferences. | B | B | B |
| HuatuoGPT (With prompt) | 93. For recurrent cervical cancer, the recommended therapeutic strategy involves a combination of surgery, radiotherapy, and chemotherapy. The choice of treatment depends on the staging of the tumor, the age and overall health status of the patient, and a personalized treatment plan. For early-stage recurrent disease, surgery is a viable option and may include total hysterectomy, pelvic lymph node dissection, or radiotherapy. For more advanced stages of the disease, radiotherapy is typically the primary treatment, with chemotherapy considered as an adjunct. | A | B | A |
| HuatuoGPT | 93. The treatment strategy for recurrent cervical cancer depends on the location of the cancer recurrence, the patient's overall health status and personal preference. Treatment options may include surgery, radiation therapy, chemotherapy, or a combination of them. For locally recurrent cervical cancer, surgery may be a viable option, especially if the lesion can be safely removed. For patients who cannot undergo surgery, radiation therapy may be a better option. Chemotherapy may also be recommended in some cases as a treatment for recurrent cervical cancer, especially if the cancer has spread elsewhere. | A | A | A |
| BioMedLM 2.7B (With prompt) | 93. What are the recommended treatment strategies for recurrent cervical cancer? Can surgery, radiotherapy, and chemotherapy be combined? The treatment of recurrent cervical cancer is a challenge for the gynecologic oncologist. The treatment of recurrent cervical cancer is determined by the site of recurrence, the extent of disease, and the patient's performance status. The treatment options include surgery, radiotherapy, chemotherapy, and a combination of these modalities. The treatment of recurrent cervical cancer is determined by the site of recurrence, the extent of disease, and the patient's performance status. The treatment options include surgery, radiotherapy, chemotherapy, and a combination of these modalities. The treatment of recurrent cervical cancer is determined by the site of recurrence, the extent of disease, and the patient's performance status. | B | B | B |
| BioMedLM 2.7B | 93. What are the recommended treatment strategies for recurrent cervical cancer? Can surgery, radiotherapy, and chemotherapy be combined? ### Response: a\. Surgery b\. Radiotherapy c\. Chemotherapy | B | B | B |
| Microsoft Phi2 (With prompt) | 93. The recommended treatment strategies for recurrent cervical cancer include surgery, radiotherapy, and chemotherapy. These treatments can be combined to provide the best possible outcome for the patient. | B | C | C |
| Microsoft Phi2 | 93. The recommended treatment strategies for recurrent cervical cancer include surgery, radiotherapy, and chemotherapy. These treatments can be combined to provide the best possible outcome for the patient. | B | C | B |
| QiZhenGPT (With prompt) | 93. The recommended treatment strategies for recurrent cervical cancer are surgery, radiotherapy, and chemotherapy. These treatments can be combined to improve the effectiveness and reduce the risk of side effects. | C | B | B |
| QiZhenGPT | 93. The recommended treatment strategies for recurrent cervical cancer are a combination of surgery, radiotherapy, and chemotherapy. This approach is known as a "watch and wait" strategy, where the cancer is monitored and treated as needed, rather than a continuous approach. Surgery may be used to remove the tumor and some surrounding tissue, while radiotherapy and chemotherapy are used to kill any remaining cancer cells. | B | B | B |
| **94. What are the treatment strategies for metastatic cervical cancer? Are there specific treatments at different metastatic sites?** | | | | |
| ChatGPT4.0 turbo (With prompt) | 94. **Treatment Strategies for Metastatic Cervical Cancer**: Treatment for metastatic cervical cancer typically involves systemic chemotherapy as the primary modality, with options including platinum-based regimens like cisplatin or carboplatin combined with paclitaxel. Bevacizumab, an anti-angiogenic agent, has also been shown to improve outcomes when added to chemotherapy in this setting. Treatment may vary based on the location of metastases; for example, palliative radiotherapy might be used for bone metastases to relieve pain and prevent fractures. The choice of treatment is guided by the goal of palliation and the specific symptoms caused by the metastatic sites. | A | B | B |
| ChatGPT4.0 turbo | 94. Treatment Strategies for Metastatic Cervical Cancer Metastatic cervical cancer treatment aims to control symptoms and prolong survival. Strategies include:  Chemotherapy: The mainstay for metastatic cervical cancer, often involving platinumbased chemotherapy combined with agents like paclitaxel or topotecan.  Targeted Therapy: Bevacizumab, a monoclonal antibody against VEGF, has been shown to improve survival when added to chemotherapy.  Palliative Care: Focuses on relieving symptoms and improving quality of life.  Sitespecific Treatments: Radiation therapy may be used for bone metastases to relieve pain, and surgical interventions might be considered for single metastatic lesions in accessible locations. | A | A | A |
| Claude 2 (With prompt) | 94. Systemic chemotherapy with cisplatin doublets (often combined with bevacizumab) is the foundation of treatment for metastatic cervical cancer. Additional site-specific therapies like stereotactic radiosurgery for oligometastases may be considered concurrently. | B | B | B |
| Claude 2 | 94. For metastatic cervical cancer, first-line treatment is systemic chemotherapy (cisplatin doublet). Targeted therapies like bevacizumab can be added. Additional treatments for specific sites include radiation for painful bone metastases, stereotactic radiosurgery for limited brain metastases, and palliative surgery. | A | A | A |
| Gemini Pro (With prompt) | 94. What are the treatment strategies for metastatic cervical cancer? Are there specific treatments at different metastatic sites?   Treatment strategies for metastatic cervical cancer:  Systemic therapy: Chemotherapy is the mainstay of treatment for metastatic cervical cancer.  Targeted therapy: Bevacizumab (Avastin) is a targeted therapy that may be used in combination with chemotherapy.  Immunotherapy: Pembrolizumab (Keytruda) is an immunotherapy drug that may be used in patients with recurrent or metastatic cervical cancer.  Specific treatments at different metastatic sites:  Brain metastases: Surgery or stereotactic radiosurgery may be used to treat brain metastases.  Lung metastases: Surgery or radiotherapy may be used to treat lung metastases.  Liver metastases: Surgery or ablation therapy may be used to treat liver metastases. | B | B | B |
| Gemini Pro | 94. **What are the treatment strategies for metastatic cervical cancer? Are there specific treatments at different metastatic sites?** The treatment strategies for metastatic cervical cancer depend on the location and extent of the metastases. Surgery, radiotherapy, and chemotherapy can all be used to treat metastatic cervical cancer, either alone or in combination. If the metastases are localized to one or two sites, surgery may be an option. However, surgery is often not possible if the metastases are extensive or if the patient has had previous surgery in the area of the metastases. Radiotherapy is another option for treating metastatic cervical cancer. Radiotherapy can be used to treat both local and distant metastases. Chemotherapy is often used in combination with radiotherapy to treat metastatic cervical cancer. Chemotherapy can help to shrink the tumors and make them more responsive to radiotherapy. There are some specific treatments that are used for metastatic cervical cancer at different metastatic sites. For example, if the metastases are in the lungs, chemotherapy may be used in combination with targeted therapy or immunotherapy. | C | C | C |
| Mistral-7B-v0.2 (With prompt) | 94.The treatment strategies for metastatic cervical cancer depend on the location and extent of the metastases. Systemic therapies, such as chemotherapy and targeted therapies, are typically used to treat metastatic disease. For example, chemotherapy may be used to shrink tumors and improve symptoms, while targeted therapies may be used to block specific molecular pathways that drive cancer growth. In some cases, local therapies, such as radiation therapy or surgery, may be used to treat specific metastatic sites. | B | C | B |
| Mistral-7B-v0.2 | 94. Treatment strategies for metastatic cervical cancer: • Chemotherapy: The primary treatment modality for metastatic cervical cancer is chemotherapy, specifically platinum-based agents such as cisplatin or carboplatin, in combination with taxanes like paclitaxel or docetaxel. This approach can help control cancer growth and improve symptoms. • Targeted therapy: For patients whose tumors have specific genetic alterations, targeted therapies such as PARP inhibitors, angiogenesis inhibitors, and anti-EGFR agents can be considered as potential treatment options. • Palliative care: In cases where the cancer has advanced and spread extensively, palliative care is essential for managing symptoms and improving the patient's quality of life. • Radiotherapy: Radiotherapy for metastatic cervical cancer is generally used as a palliative measure for controlling symptoms, such as pain or bleeding, and improving patient comfort. | B | A | A |
| Starling-LM-7B alpha (With prompt) | 94. Treatment strategies for metastatic cervical cancer involve systemic therapy, primarily chemotherapy, with or without targeted therapy, depending on the patient's clinical condition, performance status, and the presence of specific molecular markers. The treatment plan should be individualized and discussed with a multidisciplinary team, including a gynecologic oncologist. There are no specific treatments for different metastatic sites, but the treatment plan should take into account the location of metastases and the patient's overall clinical condition. | B | B | B |
| Starling-LM-7B alpha | 94. Treatment strategies for metastatic cervical cancer are similar to those for recurrent cervical cancer. The primary goal is to control the disease and alleviate symptoms. Treatment options include surgery, radiotherapy, chemotherapy, and targeted therapy. The choice of treatment depends on the location of the metastatic sites, the patient's overall health, and the patient's preferences. | B | B | B |
| HuatuoGPT (With prompt) | 94. The treatment strategy for metastatic cervical cancer varies with the staging and location of the tumor. For locally advanced disease, radiotherapy is a suitable choice and may include brachytherapy, external beam radiation, and pelvic lymph node radiotherapy. For extensively disseminated disease, chemoradiotherapy is the principal treatment, with chemotherapy drugs commonly used in conjunction with radiotherapy to enhance the therapeutic effect on the tumor. In some cases, surgical resection of other metastatic foci may also be considered. | C | C | C |
| HuatuoGPT | 94. The treatment strategy for cervical cancer in the setting of metastasis depends on the site and extent of the metastasis. For metastases limited to localized organs, they may be treated with radiation therapy or chemotherapy. For more extensive metastases, they may be treated with radiation therapy or systemic chemotherapy. In some cases, a combination of both treatments may be used. | C | C | C |
| BioMedLM 2.7B (With prompt) | 94. What are the treatment strategies for metastatic cervical cancer? Are there specific treatments at different metastatic sites? Metastatic cervical cancer is a systemic disease and the treatment strategy is determined by the site of metastasis. The treatment of metastatic cervical cancer is determined by the site of metastasis. | C | D | D |
| BioMedLM 2.7B | 94. What are the treatment strategies for metastatic cervical cancer? Are there specific treatments at different metastatic sites? ### Response: The treatment strategies for metastatic cervical cancer are not well established. The National Comprehensive Cancer Network (NCCN) guidelines recommend that patients with metastatic cervical cancer receive palliative chemotherapy. The NCCN guidelines also recommend that patients with metastatic cervical cancer receive palliative radiation therapy to the symptomatic sites. | B | A | B |
| Microsoft Phi2 (With prompt) | 94. The treatment strategies for metastatic cervical cancer vary depending on the specific metastatic site. For example, patients with liver metastases may receive chemotherapy, while those with bone metastases may undergo radiotherapy. | C | D | D |
| Microsoft Phi2 | 94. The treatment strategies for metastatic cervical cancer vary depending on the specific metastatic site. For example, patients with liver metastases may receive chemotherapy, while those with bone metastases may undergo radiotherapy. | B | B | B |
| QiZhenGPT (With prompt) | 94. The treatment strategies for metastatic cervical cancer include specific treatments at different metastatic sites. | C | D | C |
| QiZhenGPT | 94. The treatment strategies for metastatic cervical cancer are a combination of chemotherapy and radiation therapy. The specific treatments at different metastatic sites may vary depending on the location and size of the metastasis. In some cases, surgery may also be used to remove the metastasis. | C | C | C |
| **95. What are the treatment strategies for patients with cervical cancer during pregnancy? How does the treatment differ in early, intermediate, and advanced stages of pregnancy?** | | | | |
| ChatGPT4.0 turbo (With prompt) | 95. **Cervical Cancer Treatment During Pregnancy**: The treatment of cervical cancer in pregnancy is highly dependent on the stage of the cancer and the trimester of pregnancy. In early pregnancy, management may involve a more immediate treatment approach, including possible termination of the pregnancy if the cancer is at an advanced stage. In the second trimester, conservative management until fetal viability can be considered, followed by a planned preterm delivery and subsequent cancer treatment. In the third trimester, management may involve delaying treatment until after delivery, which can be expedited depending on the gestational age and fetal maturity. The decision-making process involves a multidisciplinary team to balance the best interests of both mother and fetus. | A | B | B |
| ChatGPT4.0 turbo | 95. Treatment Strategies for Cervical Cancer During Pregnancy Treatment varies depending on the stage of the pregnancy and the stage of the cancer:  Early Pregnancy: Management may lean towards termination of the pregnancy if it facilitates more immediate cancer treatment, depending on the patient's wishes and the cancer stage.  Intermediate Stages: If the cancer is diagnosed in the second trimester, the pregnancy can sometimes be continued until fetal maturity is viable, followed by cancer treatment like surgery.  Advanced Stages: Treatment may be delayed until after delivery if diagnosed late in the third trimester, especially if the cancer is at an early stage. | C | C | C |
| Claude 2 (With prompt) | 95. Treatment in early pregnancy may utilize cone biopsy or trachelectomy to preserve pregnancy. In later pregnancy stages, neoadjuvant chemotherapy can enable delaying definitive treatment until fetal maturity. After fetal viability, standard treatment per disease stage is implemented. | B | A | A |
| Claude 2 | 95. For cervical cancer during early pregnancy (<12 weeks), treatment options include immediate radical hysterectomy or delaying treatment until the second trimester. For locally advanced cases, chemotherapy can be given until fetal maturity, followed by definitive treatment. In later pregnancy (>20 weeks), treatment can be deferred until fetal lung maturity, allowing cesarean delivery followed by definitive cancer treatment. | A | A | A |
| Gemini Pro (With prompt) | 95. What are the treatment strategies for patients with cervical cancer during pregnancy? How does the treatment differ in early, intermediate, and advanced stages of pregnancy?   Treatment strategies for cervical cancer during pregnancy:  Early pregnancy (less than 24 weeks): Treatment is typically deferred until after delivery.  Intermediate pregnancy (24-32 weeks): Treatment may include surgery or radiotherapy, depending on the stage of cancer.  Advanced pregnancy (more than 32 weeks): Treatment may include a combination of surgery, radiotherapy, and chemotherapy. | D | C | C |
| Gemini Pro | 95. **What are the treatment strategies for patients with cervical cancer during pregnancy? How does the treatment differ in early, intermediate, and advanced stages of pregnancy?** The treatment strategies for patients with cervical cancer during pregnancy depend on the stage of the pregnancy and the stage of the cervical cancer. In early pregnancy, surgery is the preferred treatment for cervical cancer. However, if the cancer is advanced, radiotherapy may be used instead. In intermediate pregnancy, radiotherapy is the preferred treatment for cervical cancer. However, if the cancer is very advanced, chemotherapy may be used in combination with radiotherapy. In advanced pregnancy, chemotherapy is the preferred treatment for cervical cancer. However, if the cancer is very advanced, palliative care may be the only option. | C | C | C |
| Mistral-7B-v0.2 (With prompt) | 95.The treatment strategies for patients with cervical cancer during pregnancy depend on the stage and location of the cancer, as well as the gestational age. In early pregnancy, the focus is typically on monitoring the cancer and delaying treatment until after delivery. In intermediate and advanced stages of pregnancy, treatment may be necessary to preserve maternal health and prevent progression of the cancer. Radiation therapy and chemotherapy are generally avoided during pregnancy due to the risks to the developing fetus. Surgery may be considered in certain situations, such as when the cancer is localized and the risk to the mother outweighs the risks to the fetus. | C | D | C |
| Mistral-7B-v0.2 | 95. Treatment strategies for patients with cervical cancer during pregnancy: • Early pregnancy: Treatment during the first trimester is often deferred as the risk of adverse effects on the fetus is greatest, but it ultimately depends on the risk to the mother and the stage of the cancer. Options include close monitoring, delayed treatment until the second trimester, and alternative treatment approaches if the cancer poses a significant risk to the mother. • Intermediate pregnancy: In some cases, treatment during the second trimester may be appropriate depending on the gestational age, cancer stage, and risk factors. Treatment options include radiotherapy, chemotherapy, or brachytherapy. Surgery, especially radical hysterectomy, may be considered, but only if the benefits to the mother outweigh the potential risks to the fetus. • Advanced pregnancy: Late-stage cervical cancers during pregnancy require immediate treatment to reduce the risk to the mother. External beam radiotherapy and chemotherapy, using agents with the least potential for adverse effects on the fetus, are typically the primary options. | D | C | D |
| Starling-LM-7B alpha (With prompt) | 95. Treatment strategies for patients with cervical cancer during pregnancy depend on the stage of pregnancy and the clinical stage of the cancer. In early pregnancy, the treatment options are limited due to the risk of teratogenicity. In intermediate and advanced stages of pregnancy, a multidisciplinary approach is essential, including a gynecologic oncologist, an obstetrician, and a radiation oncologist. Treatment options may include surgery, radiation therapy, and chemotherapy, depending on the stage of pregnancy and the clinical stage of the cancer. The treatment plan should be individualized and discussed with a multidisciplinary team. | C | D | D |
| Starling-LM-7B alpha | 95. Treatment strategies for patients with cervical cancer during pregnancy are more complex due to the potential risks to the mother and the fetus. The primary goal is to preserve the pregnancy while effectively treating the cancer. Treatment options include surgery, radiotherapy, and chemotherapy, but the choice of treatment depends on the stage of pregnancy and the extent of the disease. | B | B | B |
| HuatuoGPT (With prompt) | 95. The treatment strategy for cervical cancer during pregnancy varies depending on the trimester, stage of the disease, and individual circumstances. Pregnant women with early-stage disease might consider conservative treatment during the mid-trimester, including surgery or radiotherapy, to postpone treatment until the later stages of pregnancy. Women with mid to late-stage disease may require more aggressive treatments, including surgical resection, radiotherapy, or chemoradiotherapy. Patients with cervical cancer during pregnancy should be co-managed by obstetricians and oncologists to optimize outcomes for both mother and fetus. | C | B | C |
| HuatuoGPT | 95. The treatment strategy for cervical cancer in pregnancy depends on the stage of the cancer and the patient's overall health. In early and mid-trimester pregnancies, conservative treatments, such as surgery or radiation therapy, may be preferred. In advanced pregnancy, a combination of treatments, including surgery and radiotherapy, may be recommended to control the disease and protect the mother's health. | B | B | B |
| BioMedLM 2.7B (With prompt) | 95. What are the treatment strategies for patients with cervical cancer during pregnancy? How does the treatment differ in early, intermediate, and advanced stages of pregnancy? The treatment of cervical cancer during pregnancy is similar to that of nonpregnant patients. The treatment of cervical cancer during pregnancy is based on the stage of the disease. The treatment of early-stage cervical cancer is similar to that of nonpregnant patients. The treatment of intermediate-stage cervical cancer is similar to that of early-stage cervical cancer. The treatment of advanced-stage cervical cancer is similar to that of intermediate-stage cervical cancer. The treatment of cervical cancer during pregnancy is based on the stage of the disease. The treatment of early-stage cervical cancer is similar to that of nonpregnant patients. The treatment of intermediate-stage cervical cancer is similar to that of early-stage cervical cancer. | B | B | B |
| BioMedLM 2.7B | 95. What are the treatment strategies for patients with cervical cancer during pregnancy? How does the treatment differ in early, intermediate, and advanced stages of pregnancy? ### Response: The treatment of cervical cancer during pregnancy is similar to that of nonpregnant patients. The treatment of early-stage disease is similar to that of nonpregnant patients. The treatment of intermediate-stage disease is similar to that of early-stage disease, but the radiation dose is reduced. The treatment of advanced-stage disease is similar to that of intermediate-stage disease, but the radiation dose is increased. | B | B | B |
| Microsoft Phi2 (With prompt) | 95. The treatment strategies for patients with cervical cancer during pregnancy are tailored to the stage of pregnancy. In early pregnancy, surgery may be considered, while in intermediate and advanced stages, chemotherapy is the preferred treatment option. | C | B | B |
| Microsoft Phi2 | 95. The treatment strategies for patients with cervical cancer during pregnancy are tailored to the stage of pregnancy. In early pregnancy, surgery may be considered, while in intermediate and advanced stages, radiotherapy and chemotherapy are typically used. | C | C | C |
| QiZhenGPT (With prompt) | 95. The treatment strategies for patients with cervical cancer during pregnancy vary depending on the stage of pregnancy. In early pregnancy, treatment may be recommended to prevent complications such as labor and delivery. In intermediate and advanced pregnancy stages, treatment may differ depending on the patient's overall health and the potential risks and benefits of the treatment. | C | B | B |
| QiZhenGPT | 95. The treatment strategies for patients with cervical cancer during pregnancy are similar to those used for non-pregnant patients. However, there are some additional considerations, such as the potential effects of radiation on the developing fetus and the need for more frequent monitoring during pregnancy. In early and intermediate stages of pregnancy, surgery may be considered as a first-line treatment, while in advanced stages, chemotherapy and radiation therapy may be more appropriate. | C | C | C |
| **96. Are patients with cervical cancer in pregnancy suitable for surgery? At what stage of pregnancy should surgery be considered?** | | | | |
| ChatGPT4.0 turbo (With prompt) | 96. **Surgical Considerations for Cervical Cancer in Pregnancy**: Surgery is a potential treatment option for cervical cancer during pregnancy, particularly in early-stage disease. In the first and early second trimesters, a cone biopsy or a simple trachelectomy may be considered to preserve the pregnancy if the cancer is diagnosed at a very early stage (IA1-IA2). For more advanced stages, a radical hysterectomy may be necessary, typically after fetal viability has been reached or post-delivery. The timing of surgery is critical and must consider both the cancer's aggressiveness and the gestational age to optimize outcomes for both the mother and the fetus. | A | A | A |
| ChatGPT4.0 turbo | 96. Surgery for Cervical Cancer in Pregnancy Surgery can be an option for cervical cancer in pregnancy, particularly in earlystage disease. The timing of surgery depends on:  Cancer Stage: For earlystage cancer, a conization or a trachelectomy may be possible during pregnancy.  Pregnancy Stage: Surgery is generally safer in the second trimester when the risk of miscarriage is lower and before the pregnancy significantly limits surgical access. | C | B | C |
| Claude 2 (With prompt) | 96. Yes, patients can undergo fertility-sparing surgery in early pregnancy if oncologically appropriate. Extrafascial hysterectomy is also an option in 2nd/3rd trimester. Timing is individualized balancing oncologic risks and pregnancy continuation. | B | C | B |
| Claude 2 | 96. Surgery is generally avoided during pregnancy due to high perioperative risks and complications. However, radical trachelectomy may be considered in very early pregnancy (<12 weeks) for small volume disease, especially if fertility preservation is desired. | B | B | B |
| Gemini Pro (With prompt) | 96. Are patients with cervical cancer in pregnancy suitable for surgery? At what stage of pregnancy should surgery be considered?   Suitability of surgery for cervical cancer in pregnancy: Surgery may be an option for patients with cervical cancer in pregnancy, depending on the stage of cancer and the patient's overall health.  Stage of pregnancy for surgery: Surgery is typically considered in the intermediate stage of pregnancy (24-32 weeks). | C | C | C |
| Gemini Pro | 96. **Are patients with cervical cancer in pregnancy suitable for surgery? At what stage of pregnancy should surgery be considered?** Patients with cervical cancer in pregnancy are suitable for surgery if the cancer is diagnosed in the early stages of pregnancy and the patient is healthy enough to undergo surgery. Surgery is usually performed in the second trimester of pregnancy, after the fetus is viable. | C | C | C |
| Mistral-7B-v0.2 (With prompt) | 96.Patients with cervical cancer in pregnancy may be suitable for surgery, depending on the stage and location of the cancer and the gestational age. Surgery may be considered in the second or third trimester when the risks to the mother outweigh the risks to the fetus. However, the decision to perform surgery during pregnancy should be made on a case-by-case basis, taking into account the specific circumstances of the patient and the potential risks and benefits. | C | C | C |
| Mistral-7B-v0.2 | 96. Suitability of patients with cervical cancer in pregnancy for surgery: • In general, surgery is not typically recommended during pregnancy due to the potential risks to the fetus from anesthesia, surgery, or radiation exposure. However, depending on the stage of the pregnancy, the individual cancer, and the mother's situation, surgery may be deemed necessary in some cases. | C | D | D |
| Starling-LM-7B alpha (With prompt) | 96. Patients with cervical cancer in pregnancy may be suitable for surgery, depending on the stage of pregnancy and the clinical stage of the cancer. Surgery should be considered at a stage of pregnancy where the fetus has the best chance of survival, typically after the first trimester. The decision to perform surgery should be made by a multidisciplinary team, including a gynecologic oncologist, an obstetrician, and a radiation oncologist, taking into account the patient's clinical condition, the stage of pregnancy, and the clinical stage of the cancer. | C | C | C |
| Starling-LM-7B alpha | 96. Patients with cervical cancer in pregnancy may be suitable for surgery, depending on the stage of pregnancy and the extent of the disease. Surgery is generally considered safe in the first trimester, but the risk of complications increases as the pregnancy progresses. The decision to perform surgery should be made on a case-by-case basis, taking into account the patient's overall health and the potential risks to the mother and the fetus. | B | B | B |
| HuatuoGPT (With prompt) | 96. Surgical intervention for cervical cancer during pregnancy is feasible, but its appropriateness depends on the trimester, stage of the disease, and individual circumstances. Surgery is an option for early-stage disease during pregnancy, but for mid to late-stage disease or when significant clinical risk factors are present, radiotherapy or other treatments may be more appropriate. Surgery should be performed early in the pregnancy to minimize adverse effects on the fetus. | B | B | B |
| HuatuoGPT | 96. Surgery may be appropriate for patients with cervical cancer in pregnancy, but the key is to choose the safest treatment option based on the gestational age of the woman and the stage of the cancer. Surgery is usually delayed until after pregnancy. | B | B | B |
| BioMedLM 2.7B (With prompt) | 96. Are patients with cervical cancer in pregnancy suitable for surgery? At what stage of pregnancy should surgery be considered? The majority of patients with cervical cancer are diagnosed in the second or third trimester of pregnancy. The decision to operate on a pregnant patient with cervical cancer is based on the stage of the disease, the gestational age, and the patient\'s desire to continue the pregnancy. The decision to operate on a pregnant patient with cervical cancer is based on the stage of the disease, the gestational age, and the patient\'s desire to continue the pregnancy. The decision to operate on a pregnant patient with cervical cancer is based on the stage of the disease, the gestational age, and the patient\'s desire to continue the pregnancy. | B | B | B |
| BioMedLM 2.7B | 96. Are patients with cervical cancer in pregnancy suitable for surgery? At what stage of pregnancy should surgery be considered? ### Response: Yes, surgery is indicated in the second trimester. | C | C | C |
| Microsoft Phi2 (With prompt) | 96. Patients with cervical cancer in pregnancy can be considered for surgery, but it is important to assess the stage of pregnancy and the potential risks to the mother and fetus. Surgery should be performed at the appropriate stage to minimize any potential complications. | C | D | C |
| Microsoft Phi2 | 96. Patients with cervical cancer in pregnancy can be considered for surgery, but the stage of pregnancy is an important factor to consider. Surgery should be performed at the appropriate stage to ensure the best outcome for both the mother and the baby. | B | B | B |
| QiZhenGPT (With prompt) | 96. For patients with cervical cancer in pregnancy, surgery may be suitable if the cancer is located in the uterus and is not causing any complications. The stage of pregnancy should be considered when surgery is considered. | C | C | C |
| QiZhenGPT | 96. Patients with cervical cancer in pregnancy may be suitable for surgery, depending on the stage of the cancer and the health of the mother and fetus. However, the decision to perform surgery should be made on a case-by-case basis and with the guidance of a multidisciplinary team of healthcare providers. | C | D | C |
| **97. What is the radiotherapy strategy for patients with locally advanced cervical cancer? When is a combination of chemotherapy recommended?** | | | | |
| ChatGPT4.0 turbo (With prompt) | 97. **Radiotherapy Strategy for Locally Advanced Cervical Cancer**: For locally advanced cervical cancer (stages IB2-IVA), the standard treatment is concurrent chemoradiation. The chemotherapy used is typically cisplatin, which is given weekly during the course of external beam radiation therapy followed by brachytherapy. This combination has been shown to improve survival rates significantly compared to radiation therapy alone. The goal of this approach is to maximize local and regional control of the disease while minimizing distant spread. | A | B | A |
| ChatGPT4.0 turbo | 97. Radiotherapy Strategy for Locally Advanced Cervical Cancer For locally advanced cervical cancer (stages IB2 through IVA), the standard treatment involves:  Chemoradiation: The combination of radiation therapy with concurrent chemotherapy (typically cisplatin) is the standard of care. This approach improves local control and survival compared to radiation alone.  External Beam Radiation Therapy (EBRT) followed by brachytherapy: EBRT targets the pelvis and possible metastatic sites, while brachytherapy focuses intensely on the tumor itself, maximizing the dose to the cancer while sparing surrounding tissues. | A | A | A |
| Claude 2 (With prompt) | 97. Locally advanced cervical cancer is treated with concurrent cisplatin-based chemoradiotherapy. Additional adjuvant chemotherapy after primary chemoradiation may be considered in cases with high-risk features like positive nodes. | B | A | A |
| Claude 2 | 97. The standard radiotherapy strategy for locally advanced cervical cancer is external beam radiation with concurrent cisplatin-based chemotherapy (chemoradiation). Chemoradiation is associated with improved overall survival compared to radiation alone and is considered the standard of care for stages IB2-IVA. Addition of adjuvant chemotherapy after primary chemoradiationA can further improve progression-free survival. | A | A | A |
| Gemini Pro (With prompt) | 97. What is the radiotherapy strategy for patients with locally advanced cervical cancer? When is a combination of chemotherapy recommended?   Radiotherapy strategy for locally advanced cervical cancer: External beam radiation therapy (EBRT) is the mainstay of treatment for locally advanced cervical cancer.  Combination of chemotherapy: Chemotherapy is recommended in combination with EBRT for patients with locally advanced cervical cancer who have a high risk of recurrence. | B | B | B |
| Gemini Pro | 97. **What is the radiotherapy strategy for patients with locally advanced cervical cancer? When is a combination of chemotherapy recommended?** The radiotherapy strategy for patients with locally advanced cervical cancer is to deliver a high dose of radiation to the tumor while minimizing the dose to surrounding healthy tissues. This is usually achieved using a combination of external beam radiotherapy and brachytherapy. A combination of chemotherapy and radiotherapy is recommended for patients with locally advanced cervical cancer who have a high risk of recurrence. Chemotherapy can help to shrink the tumor and make it more responsive to radiotherapy. | C | B | C |
| Mistral-7B-v0.2 (With prompt) | 97.The radiotherapy strategy for patients with locally advanced cervical cancer involves external beam radiation therapy (EBRT) and/or brachytherapy. EBRT is used to treat the entire pelvis, while brachytherapy is used to deliver a high dose of radiation directly to the tumor. A combination of chemotherapy and radiotherapy is recommended for patients with locally advanced cervical cancer to improve outcomes and reduce the risk of recurrence. Chemotherapy may be given concurrently with radiotherapy or sequentially after radiotherapy. The specific chemotherapy regimen used depends on the patient's individual circumstances and the stage of the cancer. | B | A | B |
| Mistral-7B-v0.2 | 97. Radiotherapy strategy for patients with locally advanced cervical cancer: • External beam radiotherapy (EBRT) and brachytherapy are the two primary radiotherapy treatment modalities for patients with locally advanced cervical cancer. EBRT targets the tumor from outside the body and is usually combined with brachytherapy to provide high-dose radiation directly to the tumor. • Combination Chemoradiation: In patients with locally advanced cervical cancer, chemotherapy (usually cisplatin or carboplatin) may be given concurrently with radiotherapy to increase the efficacy of treatment and improve survival. This combination therapy has been shown to be effective in several large studies and is the preferred standard treatment for this patient population. | A | B | B |
| Starling-LM-7B alpha (With prompt) | 97. The radiotherapy strategy for patients with locally advanced cervical cancer typically involves external beam radiation therapy (EBRT) combined with brachytherapy. The use of chemotherapy, such as cisplatin, is recommended in combination with radiation therapy to improve treatment outcomes. The decision to use chemoradiation should be made by a multidisciplinary team, including a gynecologic oncologist, an obstetrician, and a radiation oncologist, taking into account the patient's clinical condition, the stage of pregnancy, and the clinical stage of the cancer. | C | B | B |
| Starling-LM-7B alpha | 97. The radiotherapy strategy for patients with locally advanced cervical cancer typically involves external beam radiation therapy (EBRT) combined with brachytherapy. The combination of chemotherapy is recommended for patients with high-risk features, such as large tumor size, lymph node involvement, or positive margins after surgery. The choice of chemotherapy agents depends on the patient's overall health and the patient's preferences. | B | B | B |
| HuatuoGPT (With prompt) | 97. Radiotherapy strategies for patients with locally advanced cervical cancer include brachytherapy, external beam radiation, and pelvic lymph node radiotherapy. For early-stage locally advanced disease, combined radiotherapy and chemotherapy can enhance treatment outcomes. Chemotherapy is typically used in conjunction with radiotherapy to enhance the therapeutic effect on the tumor and reduce the recurrence rate. | B | B | B |
| HuatuoGPT | 97. For patients with locally advanced cervical cancer, radiation therapy is the treatment of choice. In some cases, radiotherapy with chemotherapy may be recommended. Chemotherapy may also be used to treat cervical cancer in some cases, especially if radiotherapy is not effective. Combination therapies are thought to have the potential to improve patient survival, but the pros and cons need to be carefully weighed in consideration of the patient's overall health and risk factors. | C | C | C |
| BioMedLM 2.7B (With prompt) | 97. What is the radiotherapy strategy for patients with locally advanced cervical cancer? When is a combination of chemotherapy recommended? The standard treatment for locally advanced cervical cancer is concurrent chemoradiotherapy (CCRT). The addition of chemotherapy to radiotherapy improves survival and reduces the risk of distant metastasis. The most commonly used chemotherapy regimen is cisplatin-based chemotherapy. The addition of chemotherapy to radiotherapy improves survival and reduces the risk of distant metastasis. | A | A | A |
| BioMedLM 2.7B | 97. What is the radiotherapy strategy for patients with locally advanced cervical cancer? When is a combination of chemotherapy recommended? ### Response: The radiotherapy strategy for patients with locally advanced cervical cancer is determined by the stage of the disease. The standard treatment for patients with stage IB2-IIB cervical cancer is concurrent chemoradiotherapy (CCRT). The standard treatment for patients with stage IIIB cervical cancer is CCRT followed by brachytherapy. The standard treatment for patients with stage IVA cervical cancer is CCRT followed by chemotherapy. | A | A | A |
| Microsoft Phi2 (With prompt) | 97. The radiotherapy strategy for patients with locally advanced cervical cancer involves a combination of chemotherapy and radiotherapy. This approach is recommended to maximize the effectiveness of treatment. | A | A | A |
| Microsoft Phi2 | 97. The radiotherapy strategy for patients with locally advanced cervical cancer involves a combination of chemotherapy and radiotherapy. This approach is recommended to maximize the effectiveness of treatment and improve patient outcomes. | B | B | B |
| QiZhenGPT (With prompt) | 97. For patients with locally advanced cervical cancer, radiotherapy may be recommended as a treatment option. The recommended timing for this treatment will depend on the patient's overall health and the location of the cancer. A combination of chemotherapy and radiotherapy may also be recommended to improve the effectiveness of the treatment. | B | A | B |
| QiZhenGPT | 97. The radiotherapy strategy for patients with locally advanced cervical cancer is typically a combination of external beam radiation therapy and internal radiation therapy, such as a radiation dose-dense schedule. A combination of chemotherapy is often recommended as well. The specific treatment plan will depend on the individual patient and the stage of their cancer. | B | B | B |
| **98. When is image-guided brachytherapy (ICRT) necessary and how is it different from conventional radiotherapy?** | | | | |
| ChatGPT4.0 turbo (With prompt) | 98. **Image-Guided Brachytherapy (IGBT) in Cervical Cancer**: Image-guided brachytherapy (IGBT) is essential in the treatment of cervical cancer, particularly in locally advanced stages (IB2-IVA). IGBT is used after external beam radiotherapy (EBRT) to deliver a high dose of radiation directly to the tumor and adjacent areas at risk while sparing surrounding healthy tissue. This method differs from conventional radiotherapy in that it uses imaging techniques such as MRI or CT scans to guide the placement of radioactive sources directly within or near the tumor. This precision allows for better targeting of the tumor, leading to improved tumor control and reduced side effects compared to conventional brachytherapy, which relies on fixed anatomical landmarks without real-time imaging guidance. | A | A | A |
| ChatGPT4.0 turbo | 98. **Image-guided brachytherapy (IGBT) vs. Conventional Radiotherapy:**  - **Necessity:** Image-guided brachytherapy (IGBT) is necessary in scenarios where high precision treatment is required to maximize the dose to the tumor while minimizing exposure to surrounding healthy tissues. It's particularly important in the treatment of cancers where the tumor is close to critical organs.  - **Difference:** The main difference between IGBT and conventional radiotherapy lies in the use of imaging technology. IGBT utilizes real-time imaging to guide the placement and movement of the radiation source directly within or next to the target tissues. This contrasts with conventional radiotherapy that typically directs radiation from outside the body and relies more on prior imaging to target the radiation. | B | B | B |
| Claude 2 (With prompt) | 98. Image-guided adaptive brachytherapy (IGABT) allows dose escalation to residual tumor while sparing organs at risk like bladder and rectum compared to conventional radiotherapy. It is a critical component of definitive treatment for locally advanced cervical cancer. | B | A | B |
| Claude 2 | 98. Image-guided brachytherapy (IGBT) allows adaptation of brachytherapy dose based on tumor geometry and is recommended for locally advanced cervical cancer (stages IB2-IVA). It enables delivery of very high doses to residual tumor while sparing organs at risk. This results in better local control and survival compared to 2D brachytherapy. | C | D | D |
| Gemini Pro (With prompt) | 98. When is image-guided brachytherapy (ICRT) necessary and how is it different from conventional radiotherapy?   Necessity of ICRT: ICRT is necessary when precise delivery of radiation to the target area is required, such as in cases of:  Cervical cancer involving the parametria or pelvic sidewall  Recurrent cervical cancer after external beam radiation therapy (EBRT)  Difference from conventional radiotherapy: ICRT uses real-time imaging to guide the placement of brachytherapy applicators, ensuring accurate delivery of radiation to the target area. Conventional radiotherapy, on the other hand, relies on external landmarks and pre-treatment planning to deliver radiation. | B | B | B |
| Gemini Pro | 98. **When is image-guided brachytherapy (ICRT) necessary and how is it different from conventional radiotherapy?** Image-guided brachytherapy (ICRT) is a type of brachytherapy that uses imaging technology to guide the placement of the radioactive sources. This allows for more precise delivery of radiation to the tumor while minimizing the dose to surrounding healthy tissues. ICRT is necessary in cases where the tumor is difficult to visualize or is located near critical structures. ICRT is also used in cases where the patient has had previous surgery or radiotherapy, which can make it difficult to place the radioactive sources accurately. ICRT is different from conventional radiotherapy in that it uses imaging technology to guide the placement of the radioactive sources. This allows for more precise delivery of radiation to the tumor while minimizing the dose to surrounding healthy tissues. | B | A | B |
| Mistral-7B-v0.2 (With prompt) | 98.Image-guided brachytherapy (IGRT) is a type of radiation therapy that involves the placement of radioactive sources directly into or near the tumor. IGRT is typically used in the treatment of cervical cancer, particularly in the advanced stages or in cases where the tumor is large or has recurred after initial treatment. IGRT allows for more precise targeting of the tumor and can result in better outcomes, including improved local control and reduced side effects, compared to conventional external beam radiotherapy. IGRT is usually delivered as intracavitary brachytherapy, where the radioactive sources are placed inside the uterine cavity, or interstitial brachytherapy, where the sources are placed directly into the tumor. | A | B | B |
| Mistral-7B-v0.2 | 98. Image-guided brachytherapy (IGRT) and its necessity in treating cervical cancer: Image-guided brachytherapy (IGRT) is a type of interstitial or intracavitary brachytherapy that utilizes imaging techniques to accurately place radioactive sources close to the tumor site. This approach offers several advantages over conventional brachytherapy: • Improved accuracy: Real-time imaging allows for more precise placement of the radioactive sources, delivering the intended dose to the tumor while minimizing exposure to healthy surrounding tissue. • Enhanced conformity to tumor shape: By incorporating image data during radiation planning, IGRT adapts to tumor shape changes during treatment due to patient movement or organ motion. This improves the conformity of the radiation dose to the tumor, which is critical for maximizing treatment efficacy. IGRT is typically necessary for patients with: • Locally advanced cervical cancer: IGRT is used to improve the therapeutic ratio by maximizing the dose to the tumor while minimizing radiation exposure to the adjacent healthy tissue. • Recurring cervical cancer: In cases of recurrence, IGRT is used to localize the tumor accurately, enabling the delivery of a precise radiation dose to the tumor site. | A | A | A |
| Starling-LM-7B alpha (With prompt) | 98. Image-guided brachytherapy (IGRT) is necessary when there is a need for high-dose radiation therapy to be delivered to a specific area, such as the cervix, in order to maximize tumor control while minimizing damage to surrounding healthy tissues. IGRT is different from conventional radiotherapy in that it uses imaging techniques, such as X-rays or ultrasound, to guide the placement of the radiation source, allowing for more precise targeting of the tumor. This results in improved treatment outcomes and reduced side effects for the patient. | B | B | B |
| Starling-LM-7B alpha | 98. Image-guided brachytherapy (IGRT) is necessary when a high degree of precision is required in the delivery of radiation therapy, such as in cases of complex tumor geometries, or when the tumor is in close proximity to critical normal structures. IGRT is different from conventional radiotherapy in that it uses imaging techniques, such as X-rays, CT scans, or MRI, to guide the placement of radiation sources and to monitor the patient's position during treatment. This allows for more accurate targeting of the tumor and minimizes the dose to surrounding healthy tissues, reducing the risk of side effects. | B | B | B |
| HuatuoGPT (With prompt) | 98. When cervical cancer patients present with specific symptoms such as pain, bleeding, or obstruction, image-guided intracavitary radiotherapy (ICRT) is necessary. Unlike traditional radiotherapy, ICRT involves placing high-dose radiation directly on the tumor, using modeling or imaging techniques for precise localization. ICRT provides a higher dosage that is more effective for some tumors, as it allows for more radiation to be concentrated in the tumor area while minimizing exposure to surrounding normal tissues. | C | B | C |
| HuatuoGPT | 98. Image-guided short-course radiotherapy (ICRT) is necessary in the treatment of cervical cancer to help prevent or treat recurrence when residual lesions or microscopic lesions are detected in the tumor or nearby lymph nodes. ICRT involves the use of image guidance to precisely target higher doses of radiation to the tumor or lymph nodes compared to standard radiotherapy. | C | C | C |
| BioMedLM 2.7B (With prompt) | 98. When is image-guided brachytherapy (ICRT) necessary and how is it different from conventional radiotherapy? ICRT is a form of brachytherapy that uses imaging to guide the placement of radioactive sources within the tumor. It is used in the treatment of cervical cancer, endometrial cancer, and other gynecologic malignancies. The use of ICRT has been shown to improve local control and survival in patients with cervical cancer.[@bib1] | B | B | B |
| BioMedLM 2.7B | 98. When is image-guided brachytherapy (ICRT) necessary and how is it different from conventional radiotherapy? ### Response: ICRT is a technique that uses a high-dose-rate (HDR) remote afterloading system to deliver a high dose of radiation to a tumor in a short period of time. The radiation is delivered in a single fraction, and the patient is treated in the outpatient setting. The radiation is delivered to the tumor in a single fraction, and the patient is treated in the outpatient setting. The radiation is delivered to the tumor in a single fraction, and the patient is treated in the outpatient setting. The radiation is delivered to the tumor in a single fraction, and the patient is treated in the outpatient setting. | C | D | D |
| Microsoft Phi2 (With prompt) | 98. Image-guided brachytherapy (ICRT) is necessary when the tumor is located in a difficult-to-reach area or when the tumor is too large for external beam radiation therapy. ICRT involves placing radioactive sources directly into the tumor using imaging techniques such as CT or MRI. This allows for more precise targeting of the tumor and can result in better treatment outcomes. | B | C | B |
| Microsoft Phi2 | 98. Image-guided brachytherapy (ICRT) is necessary when the tumor is located in a difficult-to-reach area or when the tumor is too large for external beam radiation therapy. ICRT involves placing radioactive sources directly into the tumor using imaging guidance. This allows for a higher dose of radiation to be delivered to the tumor while minimizing exposure to surrounding healthy tissue. | B | B | B |
| QiZhenGPT (With prompt) | 98. Image-guided brachytherapy (ICRT) is necessary when traditional radiotherapy is not effective in treating cervical cancer. ICRT uses high-energy radiation sources to deliver precise and targeted radiation to the tumor, while minimizing radiation to surrounding healthy tissue. This approach can improve the effectiveness and efficiency of radiotherapy in treating cervical cancer. However, ICRT is not typically used in routine treatment of cervical cancer, and it is recommended that patients undergo regular imaging studies to monitor their tumor progress. | B | A | B |
| QiZhenGPT | 98. When is image-guided brachytherapy (ICRT) necessary and how is it different from conventional radiotherapy? Image-guided brachytherapy (ICRT) is necessary when the tumor is in a location that makes it difficult to perform conventional radiotherapy, such as in the case of cervical cancer involving theWidget Placeholder for. | D | D | D |
| **99. What are the methods for monitoring recurrence after treatment of cervical cancer? Is routine imaging recommended?** | | | | |
| ChatGPT4.0 turbo (With prompt) | 99. **Monitoring Recurrence After Cervical Cancer Treatment**: Monitoring for recurrence of cervical cancer typically involves a combination of clinical examination, cytology (Pap smear), and patient-reported symptoms. Routine imaging is not universally recommended for all patients but may be tailored based on individual risk factors such as the stage of the disease, the presence of symptoms, or specific findings on physical examination. High-risk patients may undergo more frequent and detailed evaluation, which can include imaging studies like CT, MRI, or PET scans to detect recurrence early, particularly when symptoms or physical findings suggest possible recurrence. | B | B | B |
| ChatGPT4.0 turbo | 99. **Monitoring Recurrence After Cervical Cancer Treatment:**  - Methods include regular pelvic examinations, Pap tests, HPV testing, and, depending on individual risk factors, imaging tests like MRI, CT scans, or PET scans.  - **Routine Imaging:** Routine imaging is not universally recommended for all cervical cancer survivors; it's usually personalized based on factors like the initial cancer stage, treatment response, and specific symptoms that suggest recurrence. | A | A | A |
| Claude 2 (With prompt) | 99. History, physical exam, and cervical/vaginal cytology are used to monitor for cervical cancer recurrence every 3-6 months after treatment. Routine imaging is not recommended without clinical or exam findings concerning for relapse. | B | C | B |
| Claude 2 | 99. Monitoring for cervical cancer recurrence after treatment involves symptom monitoring and physical exams every 3-6 months for 2 years, then every 6-12 months. Imaging like PET/CT, CT scan, or MRI is done only based on concerning symptoms or exam findings suggestive of recurrence. Routine imaging is not recommended due to costs, false positives, and radiation exposure. | B | B | B |
| Gemini Pro (With prompt) | 99. What are the methods for monitoring recurrence after treatment of cervical cancer? Is routine imaging recommended.   Methods for monitoring recurrence:  Pelvic examination: Regular pelvic exams can detect any suspicious changes or masses in the cervix or vagina.  Pap test: Pap tests can detect precancerous or cancerous cells in the cervix.  Colposcopy: A colposcopy allows the doctor to visualize the cervix and vagina under magnification to identify any abnormal areas.  Imaging studies: Imaging studies, such as CT scans or MRI scans, may be used to evaluate for recurrence in the pelvis or distant metastases.  Routine imaging: Routine imaging is not recommended for monitoring recurrence in patients with low-risk cervical cancer. However, imaging may be recommended for patients with high-risk features, such as:  Large tumor size  Lymphovascular space invasion  Positive lymph nodes | A | A | A |
| Gemini Pro | 99. **What are the methods for monitoring recurrence after treatment of cervical cancer? Is routine imaging recommended.** The methods for monitoring recurrence after treatment of cervical cancer include: * **Physical examination:** The doctor will perform a physical examination to check for any signs of recurrence. * **Imaging tests:** Imaging tests, such as CT scans and MRI scans, can be used to detect recurrence. * **Blood tests:** Blood tests can be used to check for tumor markers, which are substances that are released into the blood by cancer cells. Routine imaging is not recommended for all patients after treatment for cervical cancer. However, imaging may be recommended for patients who have a high risk of recurrence, such as patients with locally advanced or metastatic disease. | A | A | A |
| Mistral-7B-v0.2 (With prompt) | 99.The methods for monitoring recurrence after treatment of cervical cancer include regular follow-up appointments, pelvic examinations, Pap tests, and imaging studies such as CT scans, MRI scans, or PET scans. Routine imaging is recommended for patients with high-risk features, such as positive lymph nodes or advanced stage disease, or for those who have experienced a recurrence. Imaging studies can help detect recurrence early, allowing for timely intervention and improved outcomes. | A | A | A |
| Mistral-7B-v0.2 | 99. Methods for monitoring recurrence after cervical cancer treatment: Monitoring for recurrence is crucial for early detection and effective treatment of cervical cancer. The following methods can be used for post-treatment surveillance: • Clinical examinations: Regular pelvic examinations, performed every 3-6 months, are an essential component of follow-up care. These examinations can help detect potential recurrences early based on the presence of physical symptoms. • Cervical cancer screening: Continued cervical cancer screening, such as Pap tests or HPV tests, can help diagnose recurrent disease in cases where clinical examinations are negative. • Imaging: Routine imaging, using techniques like CT, MRI, or PET/CT scans, is essential for follow-up care, especially in high-risk cases or patients with recurring disease. Imaging can help detect recurrences at an early stage, enabling prompt intervention and potentially improving treatment outcomes. However, the frequency and necessity of imaging should be weighted against the risks and costs, as overuse can result in unnecessary treatments or false positives. | A | A | A |
| Starling-LM-7B alpha (With prompt) | 99. Monitoring for recurrence after treatment of cervical cancer is crucial to ensure early detection and intervention. Methods for monitoring include regular follow-up appointments with a gynecologist, pelvic examinations, and imaging studies such as CT scans, MRI scans, and PET scans. Routine imaging is recommended for patients at high risk of recurrence, but the frequency and type of imaging should be individualized based on the patient's specific risk factors and clinical situation. | B | A | A |
| Starling-LM-7B alpha | 99. Monitoring recurrence after treatment of cervical cancer typically involves a combination of clinical examination, imaging studies, and laboratory tests. Routine imaging, such as pelvic exams, ultrasounds, and CT scans, is recommended at regular intervals after treatment to detect any signs of recurrence or new lesions. Additionally, tumor markers, such as CA-125, can be measured in the blood to help monitor the patient's response to treatment and detect any potential recurrence. | C | C | C |
| HuatuoGPT (With prompt) | 99. Common methods for monitoring for recurrence after cervical cancer treatment include routine imaging studies, such as CT scans, MRIs, or PET-CT scans. However, depending on the stage of the disease and individual treatment plans, other monitoring tools such as cervical smears, HPV testing, or PET-CT may also be used. Regular monitoring and follow-up are crucial for detecting recurrent disease early and improving the chances of successful treatment. | A | A | A |
| HuatuoGPT | 99. Methods for monitoring recurrence after treatment for cervical cancer include routine imaging, such as CT scans, MRIs, or PET-CTs.These tests can help doctors determine whether the cancer has recurred and where it has recurred. Other techniques that can be used to monitor recurrence include tumor marker testing and biopsies. | C | C | C |
| BioMedLM 2.7B (With prompt) | 99. What are the methods for monitoring recurrence after treatment of cervical cancer? Is routine imaging recommended. The use of imaging to monitor for recurrence after treatment of cervical cancer is controversial. The National Comprehensive Cancer Network (NCCN) guidelines recommend that imaging be used to monitor for recurrence in patients with stage IB2 or higher disease who have undergone radical hysterectomy and pelvic lymphadenectomy.[@bib1] The American College of Radiology (ACR) recommends that imaging be used to monitor for recurrence in patients with stage IB2 or higher disease who have undergone radical hysterectomy and pelvic lymphadenectomy.[@bib2] The European Society for Medical Oncology (ESMO) guidelines recommend that imaging be used to monitor for recurrence in patients with stage IB2 or higher disease who have undergone radical hysterectomy and pelvic lymphadenectomy.[@bib3] The National Institute for Health and Care Excellence (NICE) guidelines recommend that imaging be used to monitor for recurrence in patients with stage IB2 or higher disease who have undergone radical hysterectomy and pelvic lymphadenectomy.[@bib4] | B | B | B |
| BioMedLM 2.7B | 99. What are the methods for monitoring recurrence after treatment of cervical cancer? Is routine imaging recommended. ### Response: Routine imaging is not recommended. | C | C | C |
| Microsoft Phi2 (With prompt) | 99. The methods for monitoring recurrence after treatment of cervical cancer include regular imaging studies such as CT or MRI, as well as physical examinations and blood tests. Routine imaging is not typically recommended unless there are specific symptoms or risk factors for recurrence. | C | C | C |
| Microsoft Phi2 | 99. The methods for monitoring recurrence after treatment of cervical cancer include regular imaging tests such as CT scans, MRI scans, and PET scans. Routine imaging is not recommended unless there are specific symptoms or signs of recurrence. | B | B | B |
| QiZhenGPT (With prompt) | 99. Methods for monitoring recurrence after treatment of cervical cancer include regular follow-up appointments with the patient and their healthcare provider, as well as imaging studies such as CT, MRI, or ultrasound. Routine imaging is important to ensure that the tumor has not recurrence and to identify any potential complications or side effects of treatment. | C | B | B |
| QiZhenGPT | 99. Monitoring recurrence after treatment of cervical cancer includes regular imaging and blood tests. | C | C | C |
| **100.What are the main goals of palliative care? In patients with metastatic cervical cancer, how can palliative care be combined with radiotherapy and chemotherapy?** | | | | |
| ChatGPT4.0 turbo (With prompt) | 100. **Goals of Palliative Care and Its Combination with Other Therapies in Metastatic Cervical Cancer**: The main goals of palliative care in the context of metastatic cervical cancer are to alleviate symptoms, improve quality of life, and support the patient and family through the illness trajectory. Palliative care can be effectively combined with radiotherapy and chemotherapy to manage symptoms such as pain, bleeding, and other complications caused by the tumor. Radiotherapy can be particularly effective for symptom control, such as palliating pain from bone metastases or reducing bleeding from the tumor itself. Chemotherapy can also help reduce tumor burden and associated symptoms. Palliative care teams work alongside oncology specialists to ensure that treatments are aligned with the patient's goals and preferences, and that side effects are managed proactively. | A | A | A |
| ChatGPT4.0 turbo | 100. **Goals of Palliative Care and Combination with Other Treatments in Metastatic Cervical Cancer:**  - **Main Goals:** The main goals of palliative care include pain management, symptom relief, emotional and psychological support, and improving the quality of life for patients and their families.  - **Combination with Radiotherapy and Chemotherapy:** In patients with metastatic cervical cancer, palliative care can be effectively combined with radiotherapy and chemotherapy to manage symptoms and control pain and tumor growth. The integration of these treatments aims to prolong life, reduce symptoms, and maintain quality of life. | A | A | A |
| Claude 2 (With prompt) | 100. The main goals of palliative care are controlling cancer symptoms, maximizing comfort and quality of life, and supporting the patient holistically including psychosocial needs. Even patients undergoing disease-directed therapy benefit from early palliative care integration to manage side effects and stress of treatment. | A | B | B |
| Claude 2 | 100. The main goals of palliative care are to relieve suffering and improve quality of life. For metastatic cervical cancer, palliative care works with oncology to provide additional support. This can include pain management, counseling, treatment decision support, side effect management during chemotherapy/radiation, and end-of-life care planning. The combination optimizes both length and quality of life. | B | A | B |
| Gemini Pro (With prompt) | 100. What are the main goals of palliative care? In patients with metastatic cervical cancer, how can palliative care be combined with radiotherapy and chemotherapy?   Goals of palliative care:  Relieve symptoms and improve quality of life  Provide emotional and psychological support  Help patients and their families cope with the challenges of cancer  Combination of palliative care with radiotherapy and chemotherapy in metastatic cervical cancer:  Palliative care can be integrated into the treatment plan alongside radiotherapy and chemotherapy to:  Manage symptoms such as pain, nausea, and fatigue  Provide emotional and psychological support to patients and their families  Help patients make informed decisions about their care  Improve overall quality of life | B | A | B |
| Gemini Pro | 100. **What are the main goals of palliative care? In patients with metastatic cervical cancer, how can palliative care be combined with radiotherapy and chemotherapy?** The main goals of palliative care are to relieve symptoms, improve quality of life, and provide support to patients and their families. In patients with metastatic cervical cancer, palliative care can be combined with radiotherapy and chemotherapy to: * **Relieve symptoms:** Palliative care can help to relieve symptoms such as pain, nausea, vomiting, and fatigue. * **Improve quality of life:** Palliative care can help to improve quality of life by providing support to patients and their families. This support can include emotional support, practical help, and spiritual care. * **Provide support to patients and their families:** Palliative care can provide support to patients and their families by helping them to cope with the challenges of living with cancer. This support can include emotional support, practical help, and spiritual care. Palliative care can be combined with radiotherapy and chemotherapy in a number of ways. For example, palliative care can be used to: * **Manage side effects of radiotherapy and chemotherapy:** Palliative care can help to manage side effects of radiotherapy and chemotherapy, such as pain, nausea, vomiting, and fatigue. * **Provide support to patients and their families:** Palliative care can provide support to patients and their families by helping them to cope with the challenges of living with cancer. This support can include emotional support, practical help, and spiritual care. * **Coordinate care:** Palliative care can help to coordinate care between different healthcare providers, such as doctors, nurses, and social workers. This can help to ensure that patients receive the best possible care. | A | A | A |
| Mistral-7B-v0.2 (With prompt) | 100.The main goals of palliative care are to improve quality of life, relieve symptoms, and provide emotional and spiritual support to patients with serious illnesses, including metastatic cervical cancer. Palliative care can be combined with radiotherapy and chemotherapy by providing symptom management and supportive care alongside curative treatments. For example, palliative care can help manage pain, fatigue, and nausea, while also providing emotional support and counseling to patients and their families. Palliative care can be provided in various settings, including hospitals, clinics, and at home, and can be delivered by a multidisciplinary team of healthcare professionals, including doctors, nurses, social workers, and chaplains. | A | A | A |
| Mistral-7B-v0.2 | 100. Main goals of palliative care: The primary goals of palliative care for patients with advanced or metastatic cervical cancer include: • Improving the quality of life: Palliative care focuses on enhancing patients' physical, emotional, social, and spiritual wellbeing. This can involve managing symptoms like pain, fatigue, and shortness of breath, as well as emotional and psychological support for patients and their families. • Enhancing communication: Palliative care practitioners work to facilitate effective communication between patients, families, and healthcare providers to ensure patients have a clear understanding of their diagnosis, treatment options, and prognosis. • Providing symptom management: Palliative care includes the management of symptoms such as pain, shortness of breath, nausea, and fatigue. Effective symptom management can help improve patients' overall comfort and quality of life. Combining palliative care with radiotherapy and chemotherapy: Patients receiving palliative care can still benefit from radiotherapy and chemotherapy treatments, which can help reduce tumor size, alleviate symptoms, and improve quality of life. Palliative care is essential for managing the side effects of these treatments, including ensuring optimal pain control, symptomatic relief, and emotional support for patients and their families. By working together, palliative care and curative therapy can result in better patient outcomes and a higher overall standard of care during the advanced stages of cervical cancer. | A | A | A |
| Starling-LM-7B alpha (With prompt) | 100. The main goals of palliative care are to relieve symptoms, improve quality of life, and provide support for both the patient and their family. In patients with metastatic cervical cancer, palliative care can be combined with radiotherapy and chemotherapy in several ways. This may include the use of pain management strategies, such as medications and physical therapy, as well as addressing other symptoms like fatigue, nausea, and shortness of breath. Additionally, palliative care teams can provide emotional and psychological support, as well as assistance with advance care planning and end-of-life care. The integration of palliative care with radiotherapy and chemotherapy aims to optimize the patient's overall well-being and ensure that their needs are met throughout the course of their treatment. | A | A | A |
| Starling-LM-7B alpha | 100. The main goals of palliative care are to relieve symptoms, improve the patient's quality of life, and support the patient and their family throughout the course of the illness. In patients with metastatic cervical cancer, palliative care can be combined with radiotherapy and chemotherapy in several ways: - Pain management: Palliative care providers can work with the oncology team to optimize pain control, using a combination of medications, physical therapy, and other interventions. - Symptom management: Palliative care can help manage other symptoms, such as fatigue, nausea, and shortness of breath, which may be caused by the cancer or its treatment. - Emotional and psychological support: Palliative care teams can provide counseling, support groups, and other resources to help patients and their families cope with the emotional and psychological challenges of a serious illness. - Coordination of care: Palliative care providers can help coordinate care among the various members of the patient's healthcare team, ensuring that all aspects of the patient's needs are addressed. - Advance care planning: Palliative care can help patients and their families make informed decisions about their care, including discussing treatment options, setting goals of care, and preparing advance directives. By integrating palliative care with radiotherapy and chemotherapy, patients with metastatic cervical cancer can receive comprehensive, patient-centered care that addresses not only their cancer but also their overall quality of life and well-being. | A | A | A |
| HuatuoGPT (With prompt) | 100. The primary goal of palliative care is to provide symptom relief and improve the quality of life for patients with metastatic cervical cancer. This may involve using medications to control pain, alleviate nausea, or improve appetite, or implementing surgical or radiotherapeutic interventions to address specific symptoms. Palliative care can be combined with radiotherapy and chemotherapy to provide more comprehensive care and help manage the symptoms experienced by patients. Chemotherapy is typically used to shrink tumors, radiotherapy to control local disease, and palliative care focuses on symptom management and improving quality of life. | A | A | A |
| HuatuoGPT | 100. The main goal of palliative care is to provide comfort and symptom management for patients with metastatic cervical cancer. This may involve the use of radiation therapy, chemotherapy, or a combination of both to control the growth and spread of the cancer. Palliative care may also include surgery to relieve symptoms such as obstruction or bleeding. Palliative care may be used in combination with radiation therapy and chemotherapy to help control symptoms and improve a patient's quality of life. | A | A | A |
| BioMedLM 2.7B (With prompt) | 100. What are the main goals of palliative care? In patients with metastatic cervical cancer, how can palliative care be combined with radiotherapy and chemotherapy? The main goals of palliative care are to relieve pain and other symptoms, improve quality of life, and enhance the patient's sense of control over their illness.^[@bibr1-2374289519898857]^ The goals of palliative care are not limited to the physical symptoms of cancer, but also include the psychological, social, and spiritual aspects of the disease. | B | B | B |
| BioMedLM 2.7B | 100. What are the main goals of palliative care? In patients with metastatic cervical cancer, how can palliative care be combined with radiotherapy and chemotherapy? ### Response: Palliative care is a multidisciplinary approach to the care of patients with advanced cancer. It is designed to improve the quality of life of patients and their families facing the problems associated with cancer and its treatment. It is not a cure-oriented approach. It is not a treatment for cancer. It is a way to help patients and their families live as actively as possible with the disease. | B | B | B |
| Microsoft Phi2 (With prompt) | 100. The main goals of palliative care are to improve the quality of life for patients with advanced cancer and to manage symptoms such as pain, nausea, and fatigue. In patients with metastatic cervical cancer, palliative care can be combined with radiotherapy and chemotherapy to provide symptom relief and improve overall quality of life. | B | A | A |
| Microsoft Phi2 | 100. The main goals of palliative care are to relieve symptoms and improve quality of life for patients with advanced or metastatic cervical cancer. Palliative care can be combined with radiotherapy and chemotherapy to provide comprehensive care that addresses both the physical and emotional needs of the patient. | B | A | A |
| QiZhenGPT (With prompt) | 100. The main goals of palliative care are to provide relief from symptoms and pain associated with cervical cancer, as well as to improve the quality of life for patients. Palliative care can be combined with radiotherapy and chemotherapy in patients with metastatic cervical cancer by providing targeted | A | A | A |
| QiZhenGPT | 100. The main goals of palliative care are to improve the quality of life for patients with advanced cancer, and to manage symptoms and side effects. Palliative care can be combined with radiotherapy and chemotherapy in patients with metastatic cervical cancer by providing support and management of symptoms, and by helping to ensure that treatment is tailored to the individual needs of the patient. | A | B | A |
